# Seeking *Flavivirus* Cross-Protective Immunity

**DOI:** 10.3389/fimmu.2019.02260

**Published:** 2019-09-20

**Authors:** Lorrany dos Santos Franco, Letícia Tsieme Gushi, Wilson Barros Luiz, Jaime Henrique Amorim

**Affiliations:** ^1^Laboratório de Agentes Infecciosos e Vetores, Programa de Pós-graduação em Patologia Investigativa, Centro das Ciências Biológicas e da Saúde, Universidade Federal do Oeste da Bahia, Bahia, Brazil; ^2^Programa de Pós-graduação em Biologia e Biotecnologia de Microrganismos, Universidade Estadual de Santa Cruz, Bahia, Brazil

**Keywords:** epitopes, flavivirus, serocomplex, cross-protection, immune response

## Abstract

The Flavivirus genus is composed by viral serocomplexes with relevant global epidemiological impact. Many areas of the world present both, vector fauna and geographical conditions compatible with co-circulation, importing, emergence, and epidemics of flaviviruses of different serocomplexes. In this study, we aimed to identify both, immunological determinants and patterns of immune response possibly involved in flavivirus serocomplex cross-protection. We searched B and T cells epitopes which were thoroughly shown to be involved in flavivirus immunological control. Such epitopes were analyzed regarding their conservation, population coverage, and location along flavivirus polyprotein. We found that epitopes capable of eliciting flavivirus cross-protective immunity to a wide range of human populations are concentrated in proteins E, NS3, and NS5. Such identification of both, immunological determinants and patterns of immune response involved in flavivirus cross-protective immunity should be considered in future vaccine development. Moreover, cross-reactive epitopes presented in this work may be involved in dynamics of diseases caused by flaviviruses worldwide.

## Introduction

The Flavivirus genus of the Flaviviridae family is composed by over than 70 viral species with relevant global epidemiological impact. Flaviviruses viral particles have icosahedral capsid, are enveloped and present a single stranded genomic RNA of positive sense (1). The entry of flaviviruses is mainly based on endocytosis mediated by clathrin-coated pits and transport by an endocytic compartment. The low-pH environment within endosomes triggers conformational changes in the envelope glycoprotein (E), leading to membrane fusion of the viral envelope with the endosomal membrane and subsequent release of the nucleocapsid into the cytosol. The genomic RNA is translated into a viral polyprotein, which is cleaved by viral and cellular proteases, to originate flavivirus proteins (1). Three of these proteins are structural components of viral particle, called structural proteins. The E protein is the major antigen of the viral envelope. In addition to E protein, flavivirus particles contains also another envelope protein (M) and the capsid protein (C) (1). The polyprotein also originates non-structural proteins, which are not present on viral particle, but have important roles in viral replication and pathogenesis. There are seven non-structural proteins: NS1, NS2A, NS2B, NS3, NS4A, NS4B e NS5. After protein synthesis, the assembly of viral particles takes place in the endoplasmic reticulum. Immature viral particles are then sent to Golgi complex to be maturated by furin activity. Mature viral particles are then transported through vesicles to the plasma membrane and released by exocytosis (1).

The *Flavivirus* genus is involved in important chapters of both, World and Brazilian epidemiology histories. *Yellow fever virus* (YFV), *Dengue virus* (DENV) and *Zika virus* (ZIKV) were sequentially imported to Brazil and South America. Cases of yellow fever, caused by YFV, were first reported in Brazil in 1685. Thousands of yellow fever cases were noticed until the beginning of the twentieth century, when the *Aedes aegypti* mosquitoe was identified as the main vector of the disease. Vector eradication together with vaccination contributed to elimination of yellow fever from Brazilian urban areas (2). In 1981, the first epidemic caused by DENV was confirmed in Brazil (3). Since then, many epidemics have reached the country, usually related to the introduction of a new serotype or the change of the dominant serotype in a given region (3, 4). Such flavivirus is responsible for approximately 390 million of infections annually by one of its four serotypes (DENV1-4) (5). Thus, DENV is considered as the flavivirus of highest epidemiologic relevance of the world. But recently, ZIKV which was a pathogen previously associated with mild infections in Africa and Asia, was associated to microcephaly and *Guillain-Barré* syndrome in the Americas (6). The first cases of ZIKV infection were reported in early 2015, in the Brazilian Northeast. The virus was very quickly spreaded and 141 suspected cases of microcephaly were reported in Northeastern states, in addition to many cases of spontaneous abortions and stillbirths (6–9). In 2016 yellow fever cases were reported again in Brazil, with a total of 1,127 cases and 331 confirmed deaths until April 2018 (10). There is a number of additional flaviviruses with worldwide epidemiological importance or which are highly pathogenic. Examples are *West Nile virus* (WNV), *Japanese encephalitis virus* (JEV), *Saint Louis encephalitis virus* (SLEV) and *Rocio virus* (ROCV) (11). Such pathogens are vector-borne viruses, which are capable of traveling over long distances, carried by both, the human and/or the arthropod hosts. The risk of importing, emergence and epidemics caused by additional flaviviruses is clear in both, Brazil and other tropical regions of the world.

Humoral immune response to flaviviruses is complex and is involved in both, viral clearance and pathogenesis. An exacerbation of the disease severity mediated by antibodies, known as antibody-dependent enhancement (ADE), is observed in some cases of DENV infection. Immunoglobulins produced in a first infection by a specific serotype cross-react with viral particles present in a second infection caused by a different serotype. Viral particles are targeted to Fc-γ receptors bearing cells by non-neutralizing antibodies, which facilitates penetration and replication of DENV (1, 11). Increased viral replication leads to increased viral loads, exacerbated inflammation, increased release of inflammatory cytokines and vasoactive amines, a phenomenon known as cytokine storm (11, 12). ADE has also been described between DENV and ZIKV *in vitro* (8, 13–15). This phenomenon does not explain by itself the occurrence of severe dengue, but contribute to it in a relevant way. Investigations on ADE between different species of flavivirus have been reported, both *in vitro* (8, 13–16) and *in vivo* (17, 18). Most of the residues exposed at the external surface of flaviviral envelope are not conserved and are specific to each virus. Antibodies directed against envelope proteins potently neutralize the autologous and closely related viruses only (16, 19–21). This criterion of cross-neutralization by polyclonal antibodies has led to the classification of flaviviruses into serocomplexes (22). At present, the literature does not show evidence that antibodies are capable of conferring anti-flavivirus cross-protective immunity by themselves.

On the other hand, cellular immune responses to flaviviruses were shown to be involved in cross-protective immunity (23–28). Non-structural proteins, especially NS3 and NS5, are the main targets of T cells involved in flavivirus cross-protection (27, 29). The use of such proteins as subunit vaccine antigens was well documented in the literature. However, such vaccines were not tested in a serocomplex cross-protection context. Recent reports showed the importance of non-structural proteins in autologous (27, 30–32) or heterologous protection (23), regarding immunization with live attenuated viruses or natural infections. In contrast to structural proteins, it seems there are conserved residues in non-structural proteins which are capable of eliciting flavivirus cross-protective T cell immunity.

The Americas, Africa, Middle East, South East Asia and Europe have at least one *Flavivirus* serocomplex in circulation (23). Many areas of the world present both, vector fauna and geographical conditions compatible with co-circulation, importing, emergence and epidemics of flaviviruses. The encounter with multiple flaviviruses in a lifetime is increasingly likely. However, flavivirus cross-protective immunity is not well understood. In this study we aimed to find both, immunological determinants, and patterns of immune response possibly involved in flavivirus cross-protective immunity. We searched B and T cells epitopes which were thoroughly shown to be involved in flavivirus immunological control. Such epitopes were analyzed regarding their conservation along with different flavivirus serocomplexes. The epitopes were also studied regarding their population coverage, considering HLA allele frequencies of different human populations. Finally, epitopes with highest conservancy and population coverage were studied regarding their position along flavivirus polyprotein. Results presented in this study indicate that epitopes capable of eliciting flavivirus cross-protective immunity to a wide range of human populations are concentrated in proteins E, NS3, and NS5. Such identification of both, immunological determinants and patterns of immune response involved in flavivirus cross-protective immunity should be considered in future vaccine development. In addition, cross-reactive epitopes presented in this work may be involved in dynamics of diseases caused by flaviviruses worldwide.

## Methods

### Study Design

**Figure 1:**
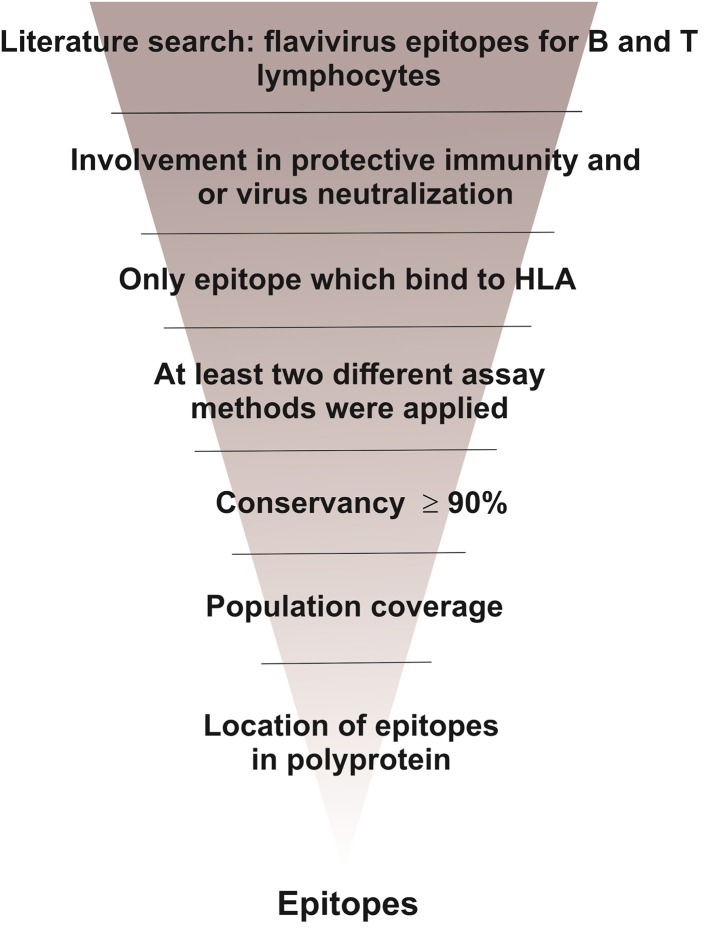
Study design.

In this study we aimed to find both, immunological determinants and patterns of immune response possibly involved in flavivirus cross-protective immunity. As shown in Figure 1, we carried out a literature search of flavivirus epitopes involved in protective immunity. Such epitopes were analyzed with regard to their conservancy, population coverage and location in flavivirus polyprotein.

### Flavivirus Polyprotein Database

A database was built with polyprotein sequences from flaviviruses of different serocomplexes isolated in different continents of the world. Viruses taken in consideration were those of highest epidemiological relevance in Brazil and worldwide: *Dengue virus* (DENV), *Zika virus* (ZIKV), *Yellow fever virus* (YFV), *West Nile virus* (WNV), *Japanese encephalitis virus* (JEV), *Saint Louis encephalitis virus* (SLEV), *Rocio virus* (ROCV), *Cacipocore virus* (CPCV), *Ilhéus virus* (ILHV), and *Iguape virus* (IGUV). Sequences in FASTA format were retrieved from the National Center for Biotechnology Information (NCBI) protein database from September-2017 to September-2018 (http://www.ncbi.nlm.nih.gov/protein/). We aimed to recover sequences from all listed flavivirus species, isolated in all continents, with a limit of three sequences per country of each viral species. We noticed that for some species there were few deposits. In these cases, all available sequences were selected. Additional criteria for selecting sequences were: (i) complete annotation of structural and non-structural proteins within deposited polyprotein sequence and (ii) absence of undefined amino acid into sequence. The database consisted of a total of 325 polyprotein amino acid sequences (Supplemental Material 1).

### Search of T Cell Epitopes Involved in Flavivirus Protective Immunity

We carried out a literature search for reports of experimental characterization of protective epitopes. CD4^+^ and CD8^+^ T cell epitopes which were related to protective immune response to flavivirus were searched in reports available at Pubmed from September-2017 to September-2018 (https://www.ncbi.nlm.nih.gov/pubmed/). Criteria for selecting research articles were: (i) evaluation of epitope binding to HLA; (ii) determination of cytokine production pattern for both, CD4^+^ and CD8^+^ T cells; (iii) use of at least two different methods for determining cytokine production pattern and (iv), clear association with control of flavivirus infection. This last criterion involves validation of epitopes associated with survival under challenge experiments using humanized animals (transgenic mice expressing HLA), control of viral load under challenge experiments using humanized animals, infection of humans without disease and identification of important epitopes in immune responses elicited by well-known protective vaccines. Words used in the search were: CD4^+^ T cell, CD8^+^ T cell, epitopes, protection, immunity, *Dengue virus, Zika virus, Yellow Fever virus, West Nile virus, Japanese encephalitis virus, Saint Louis encephalitis virus, Rocio virus, Cacipocore virus, Ilheus virus*, and *Iguape virus*.

### Search of B Cell Epitopes Targeted by Neutralizing Antibodies

B cell epitopes were searched at Immune Epitope Database and Analysis Resource-IEDB (http://www.iedb.org) (34). The search was carried out until September 30, 2018, using the following conditions: any epitope at the epitope field; flavivirus at the antigen field, humans in the host field; positive assays only, B cells assay, *in vitro* and/or *in vivo* neutralization and 3D structure at the assay field; any MHC restriction at the MHC restriction field and infectious disease at the disease field.

### Conservancy Analysis of Selected Epitopes

The IEDB conservancy analysis tool (http://tools.iedb.org/conservancy) was used to determine the conservancy of the selected epitopes among the flavivirus sequences in the database previously constructed, as previously described (34, 35). Only epitopes at least 90% conserved among all sequences were selected.

### Population Coverage Analyses

Epitopes selected after conservancy analysis were submitted to population coverage analysis, by using the IEDB population coverage calculation tool (http://tools.immuneepitope.org/tools/population/iedb_input), as previously described (35, 36).

### Localization of Selected Epitopes Along Flavivirus Polyprotein

Epitopes with high conservancy were submitted to location analysis along flavivirus polyprotein. Preliminary localization was retrieved from NCBI annotations. Then, 3D protein models of E, NS3 and NS5, deposited at the Protein Data Bank- PDB (https://www.rcsb.org/), were used for fine localization. Pymol (http://www.pymol.org/) was used to highlight selected epitopes along the proteins 3D models, as previously described (33, 35).

## Results

### Selection and Localization of Epitopes Which Are Targets for Neutralizing Antibodies

**Figure 2:**
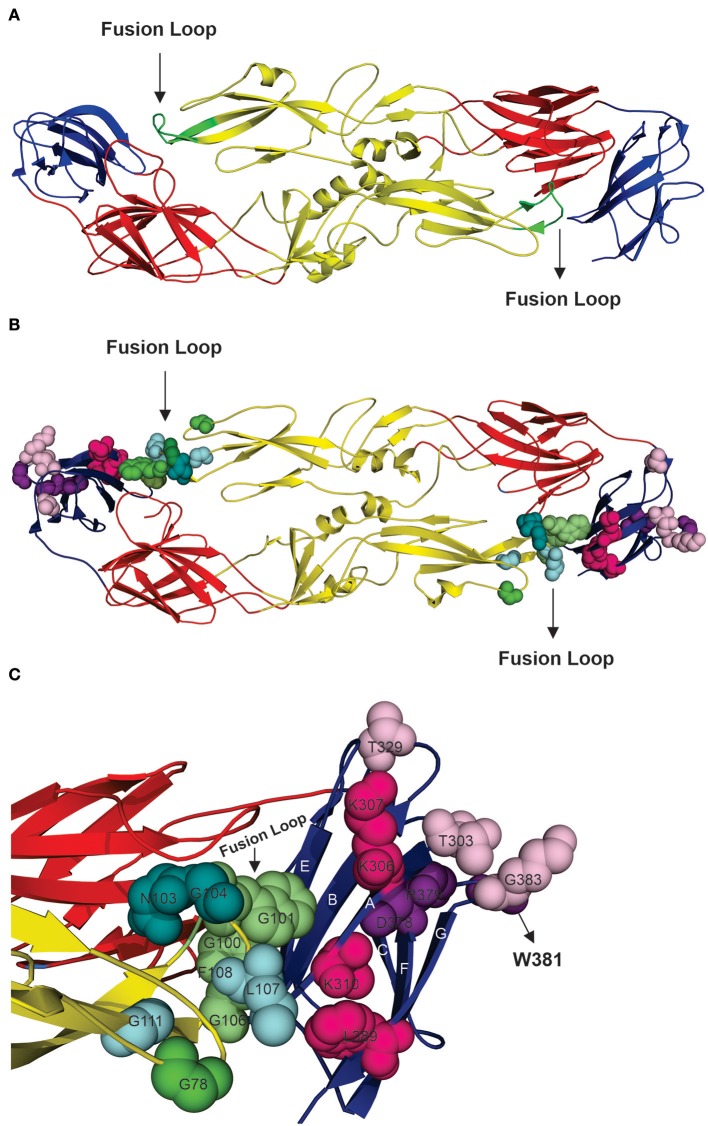
Structural analysis of highly conserved neutralizing epitopes in flaviviruses. **(A)** Model of DENV envelope glycoprotein arranged as a dimer [(33), PDB number 1UZG]. Domain I is shown in red, domain II is shown in yellow, and domain III is shown in blue, and the fusion loop is shown in green. **(B)** Overview of locations of the neutralizing epitopes in E protein. **(C)** Approximate view of locations of epitopes which are targets for neutralizing antibodies. Epitopes shown to be located in domain II are: G78, W101, F108 (shown in green), W101, L107, G111 (shown in cyan), N103, G104, G111 (shown in deepteal cyan) and G100, W101, F108 (shown in lime green). Note that these epitopes are overlapping and that are they concentrated at the fusion loop and share W101 and G111. Domain III presents the epitopes: T303, T329, G383 (shown in light pink), K307, K310, L389 (shown in hot pink) and D378, R379, W381 (shown in violet purple).

From over than 2,000 epitopes retrieved from search carried out as described in materials and methods, 19 were selected after conservancy analyses. One of the epitopes is contained inside a larger epitope as a consensus, in the same location. Thus, 18 epitopes are shown in Table 1. Such epitopes are discontinuous. From these, seven epitopes were shown to be 100% conserved and were selected for structural analyses. All selected epitopes are located in flavivirus envelope glycoprotein, mainly in domains II and III, as shown in Figure 2. Our results indicate that the seven neutralizing epitopes selected are highly conserved among flaviviruses and may be involved in cross-protection and restriction of virus circulation in some regions of the world.

**Table 1:** Discontinuous B lymphocyte epitope selected from IEDB website and, posteriorly, chosen through conservancy analyzes using cut off ≥90%.

**Epitope ID^a^**	**Virus^b^**	**Epitope sequence/location**	**Conservancy (≥90%)**	**100% identity^c^**	**Protein location**
110534	WNV	W101, G104, G106	99.67%	DENV1, DENV2, DENV3, DENV4, ZIKV, YFV, WNV	E protein
161539	DENV	W101, G106, L107, F108	99.34%	DENV1, DENV2, DENV3, DENV4, ZIKV, YFV, WNV	E protein
178102	DENV	W101, L107,G109	99.67%	DENV1, DENV2, DENV3, DENV4, ZIKV, YFV, WNV	E protein
191061	DENV	T76, W101, G106, L107, F108	90.43%	DENV1, DENV2, DENV3, DENV4, ZIKV, WNV	E protein
191066	DENV	W101, G106, F108	99.67%	DENV1, DENV2, DENV3, DENV4, ZIKV, YFV, WNV	E protein
191060	DENV	T76, L107, F108	90.76%	DENV1, DENV2, DENV3, DENV4, ZIKV, WNV	E protein
191068	DENV	W101, L107, F108	99.67%	DENV1, DENV2, DENV3, DENV4, ZIKV, YFV, WNV	E protein
191062	DENV	T76, W101, L107, F108	90.76%	DENV1, DENV2, DENV3, DENV4, ZIKV, WNV	E protein
191057	DENV	G78, W101, L107, F108	99.67%	DENV1, DENV2, DENV3, DENV4, ZIKV, YFV, WNV	E protein
238073	DENV	R99, G102, G106, L107	99.34%	DENV1, DENV2, DENV3, DENV4, ZIKV, YFV, WNV	E protein
504083	DENV	R73, G78, E79	91.42%	DENV1, DENV2, DENV3, DENV4, ZIKV, WNV	E protein
161528	DENV	K307, K310, L389	100.00%	DENV1, DENV2, DENV3, DENV4, ZIKV, YFV, WNV	E protein
161534	DENV	T303, T329, G383	100.00%	DENV1, DENV2, DENV3, DENV4, ZIKV, YFV, WNV	E protein
191056	DENV	G78, W101, F108	100.00%	DENV1, DENV2, DENV3, DENV4, ZIKV, YFV, WNV	E protein
504090	DENV	W101, L107, G111	100.00%	DENV1, DENV2, DENV3, DENV4, ZIKV, YFV, WNV	E protein
504074	DENV	N103, G104, G111	100.00%	DENV1, DENV2, DENV3, DENV4, ZIKV, YFV, WNV	E protein
504080	DENV	G100, W101, F108	100.00%	DENV1, DENV2, DENV3, DENV4, ZIKV, YFV, WNV	E protein
530626	DENV	D378, R379, W381	100.00%	DENV1, DENV2, DENV3, DENV4, ZIKV, YFV, WNV	E protein

a *Number of epitope ID at IEDB website*.

b *Virus in which the epitope was described*.

c *Viruses in which epitopes have 100% identity*.

### Selection of T Cell Epitopes Which May Be Involved in Flavivirus Serocomplex Cross-Protective Immunity

Nineteen research articles were selected reporting 529 epitopes. Such antigen determinants were experimentally characterized and associated with protective immunity. From the 529 epitopes 15 were selected after conservancy analysis. All selected epitopes are class I HLA ligands. Their amino acid sequences, HLA ligands, locations and percent conservations are shown in Table 2. Three epitopes showed conservation higher than 99% among all flavivirus polyproteins analyzed. In addition, virus-specific identity was also analyzed and is shown in Table 3. Epitope identity was shown to be constant in most of cases for each flavivirus. Together, these results indicate that there are fifteen epitopes associated with protective immunity which are highly conserved among flavivirus serocomplexes.

**Table 2:** T cell epitopes selected from literature review as contributing to flavivirus control and showing at least 90% conservation in the polyprotein dataset.

**References^a^**	**Epitope**	**HLA allele^b^**	**Protein^c^**	**Conservancy^d^**
Wen et al. (24)	APTRVVAAEM	B*07:02	NS3 1725–1734	91.08%
Weiskopf et al. (27), Weiskopf et al. (37), Elong Ngono et al. (38), Weiskopf et al. (39)	APTRVVAAEM	B*07:02, B*35:01	NS3 1700–1709	91.08%
Weiskopf et al. (27), Weiskopf et al. (37)	DPASIAARGY	B*35:01	NS3 1768–1777	91.69%
Weiskopf et al. (27)	DISEMGANF	A*26:01	NS3 1887–1895	93.85%
	KAKGSRAIW	B*57:01	NS5 2962–2970	92.00%
	RFLEFEALGF	A*23:01	NS5 2977–2986	100.00%
de Melo et al. (40)	TDTTPFGQQRVFKEK	B*39	NS5 345–359	100.00%
	EFGKAKGSRAIWYMW	B*15	NS5 465–479	99.69%
	AKGSRAIWYMWLGAR	B*15	NS5 469–483	99.69%
Weiskopf et al. (37)	LPVWLAYKVA	B*35:01	NS3 2005–2014	93.85%
Elong Ngono et al. (38)	APTRVVASEM	B*07:02	NS3 1700–1709	96.92%
Weiskopf et al. (39)	SRAIWYMWLGARFLE	DRB1*01:01	NS5 2966–2980	94.15%
Rivino et al. (41)	GEAAGIFMTA	B*40:06	NS3 301–315	92.92%
Duan et al. (42)	WYMWLGARFL	A*24:02	NS5 475–484	94.46%
Turtle et al. (43)	MTTEDMLQVW	B*58:01	NS5 3336–3345	96.00%

a *References of experimental validation of epitopes involved in the protective immune response against flavivirus that presented ≥ 90% of conservation in Flavivirus polyprotein sequences analyzed*.

b *HLA alleles which bind the selected epitopes*.

c *Location of selected epitopes in flavivirus polyprotein. Numbers refer to starting and ending positions of epitopes into flavivirus polyprotein sequences. Proteins in which epitopes are located were also indicated*.

d *Percent conservation of epitopes flavivirus polyprotein dataset. A homology cut off of 90% was applied with regard to flavivirus polyprotein dataset*.

**Table 3:** Percent of identity of selected epitopes into species-specific polyprotein datasets.

**Epitopes sequences**	**DENV1**	**DENV 2**	**DENV3**	**DENV4**	**ZIKV**	**YFV**	**WNV**	**SLEV**	**JEV**	**IGUV**	**ILHV**	**ROCV**
APTRVVAAEM^a^^,^ ^b^	90%	100%	100%	100%	100%	–	100%	–	100%	100%	90%	90%
DPASIAARGY^a^	100%/90%	100%	100%	–	90%	90%	100%	100%	100%	90%	100%	100%
DISEMGANF	100%	100%	100%	100%	100%	100%	100%	100%	100%	100%	100%	100%
KAKGSRAIW	100%	100%	100%	100%	100%	100%	100%	100%	100%	100%	100%	100%
RFLEFEALGF	100%	100%	90%	100%	100%	90–100%	100%	100%	90%	100%	100%	100%
TDTTPFGQQRVFKEK	100%	100%	100%	100%	93.33%	100%	100%	100%	100%	100%	100%	100%
EFGKAKGSRAIWYMW	100%	100%	100%	93.33%	100%	100%	93.33%	100%	93.33%	100%	100%	100%
AKGSRAIWYMWLGAR	100%	100%	100%	100%	100%	100%	93.33%	100%	93.33%	100%	100%	100%
LPVWLAYKVA	90%	90–100%	90%	90%	90%	–	100%	100%	100%	90%	90%	90%
APTRVVASEM	100%	90%	90%	90%	90%	90%	90%	–	90%	90%	–	–
SRAIWYMWLGARFLE	100%	100%	93,33%	100%	100%	93.33–100%	93.33%	100%	–	100%	100%	100%
GEAAGIFMTA	90%	100%	90%	90%	90%	–	90%	90%	90%	–	90%	90%
WYMWLGARFL	100%	100%	100%/90%	100%	100%	100%/90%	90%	100%	–	100%	100%	100%
MTTEDMLQVW	90%	90%	90%	90%	90%	90%	90%	90%	100%	90%	100%	90%

*Only percent identities ≥90% were indicated*.

a *Epitopes experimentally validated by more than one research group. Repeated epitopes were removed from the table*.

b *Epitopes which share amino acid sequences, but are located in different positions of viral polyprotein*.

### Population Coverage Analyses of Conserved T Cell Epitopes

As shown in conservancy analyses, we selected fifteen epitopes with at least 90% identity among all flaviviruses. Such epitopes were shown to present relevant population coverages with regard to the most prevalent HLA alleles and main ethnicities in Brazil and United States, as shown in Table 4. In addition, population coverage for selected epitopes were shown to be also relevant for most of world regions, as shown in Table 5. Our results indicate that those highly conserved epitopes bind to HLA molecules of different human populations worldwide, with high population coverage.

**Table 4:** Population coverage by ethnicity in Brazil and United States of America.

**Epitopes sequences**	**Brazil (62.30%)^a^**				**United States of America (88%)^a^**							
	**Amerindian**	**Caucasoid**	**Mixed**	**Mulatto**	**Amerindian**	**Asian**	**Austronesian**	**Black**	**Caucasoid**	**Hispanic**	**Mestizo**	**Polynesian**
APTRVVAAEM	0.00%	12.18%	19.18%	0.00%	3.46%	5.14%	0.00%	14.27%	23.23%	10.44%	9.67%	2.77%
APTRVVAAEM	0.00%	19.86%	28.09%	0.00%	30.32%	13.15%	0.00%	25.85%	33.59%	21.69%	22.02%	31.55%
DPASIAARGY	0.00%	8.21%	9.94%	0.00%	27.37%	8.23%	0.00%	12.54%	11.87%	11.91%	13.02%	29.22%
DISEMGANF	0.00%	7.26%	4.84%	0.00%	1.65%	7.37%	0.00%	2.91%	5.79%	5.59%	4.72%	23.24%
KAKGSRAIW	0.00%	1.00%	1.40%	0.00%	1.27%	3.72%	0.00%	1.40%	7.21%	2.36%	3.93%	0.00%
RFLEFEALGF	0.00%	10.32%	7.94%	0.00%	1.24%	0.56%	0.00%	20.25%	3.04%	6.90%	4.53%	0.00%
TDTTPFGQQRVFKEK	35.68%	3.17%	9.75%	0.00%	21.89%	3.40%	0.00%	2.71%	3.34%	12.62%	16.68%	14.78%
EFGKAKGSRAIWYMW	22.91%	14.41%	17.55%	0.00%	9.99%	24.90%	0.00%	24.57%	15.22%	14.70%	14.86%	30.06%
AKGSRAIWYMWLGAR	22.91%	14.41%	17.55%	0.00%	9.99%	24.90%	0.00%	24.57%	15.22%	14.70%	14.86%	30.06%
LPVWLAYKVA	0.00%	8.21%	9.94%	0.00%	27.37%	8.23%	0.00%	12.54%	11.87%	11.91%	13.02%	29.22%
APTRVVASEM	0.00%	12.18%	19.18%	0.00%	3.46%	5.14%	0.00%	14.27%	23.23%	10.44%	9.67%	2.77%
SRAIWYMWLGARFLE	0.41%	10.76%	9.43%	11.64%	0.98%	5.50%	0.99%	4.92%	14.81%	7.12%	7.74%	0.00%
GEAAGIFMTA	0.00%	0.00%	0.00%	0.00%	0.00%	6.69%	0.00%	0.08%	0.00%	0.43%	0.40%	11.24%
WYMWLGARFL	14.44%	21.85%	16.16%	0.00%	53.94%	33.22%	0.00%	4.57%	18.26%	23.41%	26.97%	56.79%
MTTEDMLQVW	0.00%	4.15%	4.35%	0.00%	0.59%	11.70%	0.00%	7.01%	1.70%	2.59%	1.78%	0.00%
Total coverage^b^	60.60%	65.91%	69.96%	11.64%	81.09%	74.53%	0.99%	68.51%	71.45%	68.62%	71.52%	92.04%

a *Percent values between parentheses refer to total population coverage of all epitope combined in countries*.

b *Population coverage of the epitope set presented in table computed in combination for each ethnicity*.

**Table 5:** Population coverage of selected epitopes for populations of different regions of the world.

**Epitopes** ** sequences**	**North** ** America**	**Central** ** America**	**South** ** America**	**Europe**	**East** ** Africa**	**West** ** Africa**	**Central** ** Africa**	**North** ** Africa**	**South** ** Africa**	**Northeast** ** Asia**	**South** ** Asia**	**East** ** Asia**	**Southeast** ** Asia**	**Southwest** ** Asia**	**Oceania**
APTRVVAAEM	12.70%	0.00%	4.75%	21.45%	6.15%	7.01%	10.54%	5.57%	0.03%	2.60%	2.74%	9.44%	1.20%	6.02%	3.17%
APTRVVAAEM	25.20%	0.00%	8.43%	30.73%	10.51%	24.53%	20.80%	16.02%	0.05%	6.29%	8.62%	22.01%	3.76%	11.28%	5.37%
DPASIAARGY	13.41%	0.00%	3.78%	10.50%	4.51%	18.20%	10.86%	10.77%	0.02%	3.74%	5.96%	13.23%	2.58%	5.44%	2.24%
DISEMGANF	5.35%	0.00%	1.95%	7.73%	1.91%	5.83%	2.20%	2.45%	0.01%	4.15%	8.55%	12.73%	5.68%	8.61%	2.31%
KAKGSRAIW	3.36%	0.00%	0.98%	6.24%	1.77%	1.65%	0.00%	2.78%	0.02%	1.42%	4.90%	0.37%	1.26%	1.25%	0.60%
RFLEFEALGF	8.21%	0.80%	4.42%	4.32%	14.89%	27.50%	18.14%	19.40%	0.07%	0.97%	1.15%	0.58%	0.11%	5.21%	1.35%
TDTTPFGQQRVFKEK	8.62%	0.00%	22.55%	4.12%	2.99%	2.55%	1.61%	7.22%	0.01%	3.63%	1.53%	7.06%	9.06%	7.36%	6.76%
EFGKAKGSRAIWYMW	19.66%	0.00%	25.68%	12.71%	25.37%	26.83%	17.15%	15.84%	0.15%	48.92%	18.16%	26.01%	31.42%	12.26%	28.69%
AKGSRAIWYMWLGAR	19.66%	0.00%	25.68%	12.71%	25.37%	26.83%	17.15%	15.84%	0.15%	48.92%	18.16%	26.01%	31.42%	12.26%	28.69%
LPVWLAYKVA	13.41%	0.00%	3.78%	10.50%	4.51%	18.20%	10.86%	10.77%	0.02%	3.74%	5.96%	13.23%	2.58%	5.44%	2.24%
APTRVVASEM	12.70%	0.00%	4.75%	21.45%	6.15%	7.01%	10.54%	5.57%	0.03%	2.60%	2.74%	9.44%	1.20%	6.02%	3.17%
SRAIWYMWLGARFLE	13.55%	1.34%	4.05%	15.19%	3.96%	4.47%	2.12%	2.33%	0.00%	2.77%	9.25%	10.21%	0.78%	2.67%	0.81%
GEAAGIFMTA	0.22%	0.00%	0.00%	0.11%	0.00%	0.41%	0.00%	0.72%	0.03%	3.11%	14.14%	8.33%	2.06%	5.32%	0.00%
WYMWLGARFL	22.87%	0.00%	23.76%	17.38%	2.38%	5.04%	2.00%	8.00%	0.04%	24.09%	16.66%	49.65%	40.08%	10.29%	52.33%
MTTEDMLQVW	4.66%	1.40%	1.40%	1.45%	11.14%	8.44%	6.51%	5.93%	0.02%	7.68%	9.08%	3.75%	13.27%	5.13%	2.59%
Total Coverage^a^	74.17%	3.50%	67.96%	69.60%	58.61%	73.78%	55.97%	60.88%	0.30%	74.77%	66.06%	84.70%	74.75%	54.27%	73.66%

a *Population coverage of the epitope set presented in table computed in combination for each region of the world*.

### Localization of Selected T Cell Epitopes Along Flavivirus Polyprotein

**Figure 3:**
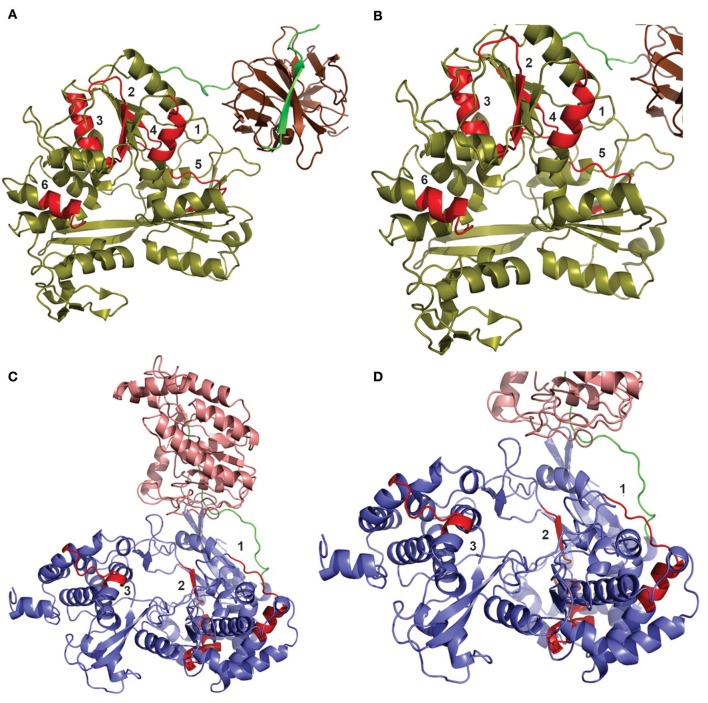
Distribution of T cell epitopes ≥90% conserved among flavivirus polyproteins. The locations of epitopes are shown in two different models: DENV NS3 protein **(A,B)** and ZIKV NS5 protein **(C,D)**. **(A)** The helicase domain of the DENV NS3 protein is shown in olive, while its protease domain is shown in chocolate brown. Six epitopes (shown in red) were shown to be located in NS3, all of them located in the protease domain. **(B)** Zoomed view of NS3 protein to detail epitopes. The numbers refer to the following epitopes, respectively: (1) APTRVVAAEM or APTRVVASEM, (1) APTRVVAAEM, (2) DPASIAARGY, (3) GEAAGIFMTA, (4) DISEMGANF, and (5) LPVWLAYKVA. **(C)** The methyltransferase domain of the NS5 protein is shown in light salmon, while its RNA polymerase domain is shown in slate blue. Eight epitopes (shown in red) were shown to be located in the NS5 protein, all of them in the RNA polymerase domain. **(D)** Approximate view of NS5 protein to detail epitopes. Some epitopes present overlapping. Thus, we show three different regions: Region 1 is composed by one epitope TDTTPFGQQRVFKEK. Region 2 contain six epitopes: KAKGSRAIW, RFLEFEALGF, EFGKAKGSRAIWYMW, AKGSRAIWYMWLGAR, SRAIWYMWLGARFLE, and WYMWLGARFL. Region 3 is composed of only one epitope: MTTEDMLQVW.

All of selected epitopes were concentrated in flavivirus non-structural proteins 3 and 5 (NS3 and NS5, respectively). Thus, we used NS5 and NS3 3D models to study the locations of the epitopes. From fifteen epitopes, six were located in Helicase domain of the NS3 protein (NS3H), and eight were located in the RNA-dependent RNA polymerases domain of the NS5 protein (NS5p) (Figure 3). These results indicate that NS3H and NS5p concentrate most of relevant T cell epitopes in the context of flavivirus cross-protective immunity.

## Discussion

In this study, we aimed to identify both, immunological determinants and patterns of immune response possibly involved in flavivirus serocomplex cross-protection. Our premise was based on epidemiology of flaviviruses: most of them have both, vectoring and geographic conditions to co-circulate all together in the Americas, Africa, Asia and Oceania (44–49). However, it does not happen. In addition, recent studies showed evidences of cross-protective immunity induced by vaccines or sequential infections (23, 24, 26, 50–54). We searched B and T cells epitopes, which were thoroughly shown to be involved in flavivirus infection control. We found a relevant number of epitopes which are capable of eliciting flavivirus protective immunity and are also highly conserved among serocomplexes. In addition, such epitopes cover a wide range of human populations and are concentrated in proteins E, NS3, and NS5. For such epitopes, mechanisms involved in virus control targeting the E protein are all related to antibody-mediated virus neutralization. On the other hand, mechanisms involved in virus control by targeting NS3 and NS5 proteins are mainly related to cytotoxicity mediated by CD8^+^ T limphocytes.

The glycoprotein E is involved in binding of the viral particle to host cell receptors. In addition, such protein is also involved in membrane fusion. The E glycoprotein is arranged in three domains (DI, DII, and DIII). Domains I (DI) is central, surrounded by DII and DIII (55). The hydrophobic fusion loop is located in DII. It is highly conserved in flaviviruses serocomplexes due to its important role in mediating membrane fusion (55). The fusion loop has the same conformation in both protein forms trimer and dimer. In trimer, three amino acids are conserved in all flaviviruses (Trp 101, Leu 107, and Phe 108) and are exposed on molecular surface. Such amino acids have an important role in holding the fusion loop structure (33). Replacement of the Trp101 by an alanine prevents membrane fusion (56). In addition, natural infection by DENV was hypothesized to generate anti-DENV antibodies with cross-neutralizing activity against the fusion loop (57). All of discontinuous epitopes found in our study are located in E protein and most of them present these three conserved amino acids. We hypothesize that B cell epitopes presented in our study remain highly conserved across flavivirus serocomplexes due to their location at fusion loop.

The role of DIII in the flaviviral cycle is still unclear, although frequently related to a receptor binding function (55, 58, 59). It was previously shown that antibodies targeting different regions in DIII are able to strongly neutralize WNV. The regions are the N-terminal linker region (residues 302 to 309) and three strand-connecting loops, namely, BC (residues 330 to 333), DE (residues 365 to 368), and FG (residues 389 to 391) (60). In our study we found three discontinuous epitopes which are located in those regions: T303 T329 G383, K307 K310 L389, and D378 R379 W381. These epitopes revealed 100% conservancy among all 325 sequences analyzed. All of the selected B cell epitopes were show to be important in DENV neutralization (57, 61–63). Of course, a physic validation regarding cross-neutralizing activity remains to be carried out. However, positions highlighted in Figure 2 are located in the same structures of flaviviruses with known envelope glycoprotein structure. Our results indicate that such epitopes are important targets to be considered in flavivirus serocomplex cross-immunity due to their key roles in viral life cycle.

With regard to conservation of T cell-specific immunological determinants among flavivirus serocomplexes, the YVF lost the highest amount of 100% conserved T cell epitopes (see Table 3). This is in accordance with the highest genetic distance of YFV serocomplex with regard to other flavivirus serocomplexes (23). An important explanation for the epidemiology of flaviviruses may arise from this observation. For example, vaccination against YFV in Brazil is not currently related to protection against DENV and ZIKV epidemics. However, DENV and JEV serocomplexes share most of epitopes identified in this work. It seems immunity to DENV precludes viruses of JEV serocomplex of circulating in South America. Recent studies showed evidences of cross-protective immunity between DENV and JEV serocomplexes (23, 54). The same does not occur in Central America, Africa and Asia. Interestingly, Central America and South Africa present a low population coverage of the selected T cell epitopes. Nevertheless, population coverage of class-I HLA specific epitopes presented in this work does not seem to explain the whole scenario. For example, the population of United States is 88% covered by the set of epitopes, but there is co-circulation of DENV and JEV serocomplexes in that country. In addition, most of regions in Africa and Asia, which have also a high population coverage of our set of epitopes, present co-circulation of DENV and JEV serocomplexes. The explanation for this is probably related to class-II HLA restricted epitopes, from which only two were selected according to our criteria. Such epitopes did not present a high conservation among all serocomplexes and probably confer cross-protection in specific combinations of viruses and serocomplexes, as previously described (23). It was recently shown that cross-reactive epitopes can promote recall from a pool of flavivirus serocomplex cross-reactive memory CD4^+^ T cells (23). However, such epitopes are not enough conserved in order to induce cross-protection among all serocomplexes.

Cross-protective immunity into a given serocomplex seems to be more easily achieved (27, 50–53, 64, 65). Such cross-protection depends on recall of serocomplex cross-reactive memory CD4^+^ T cells. There is not a high number of highly conserved class-II HLA restricted epitopes among all serocomplexes. Nevertheless, we identified two peptides capable of binding class-II HLA, involved in anti-YFV protective immune response which are highly conserved among all flavivirus serocomplexes. Thus, it seems achieving multivalent protection among serocomplexes depends on overcoming immunodominance. Highly conserved class-II HLA restricted epitopes do not seem to be immunodominant in order to promote recall of serocomplex cross-reactive memory CD4^+^ T cells in a multivalent way.

With regard to class-I HLA restricted epitopes, we found fifteen highly conserved peptides which were thoroughly shown to be involved in flavivirus infection control. The peptides APTRVVAAEM, DPASIAARGY, DISEMGANF, KAKGSRAIW, and RFLEFEALGF are located in the NS3 or NS5 proteins. They bind to a subset of multifunctional CD8+ T limphocytes capable of producing IFN-γ and TNF-α against DENV infection (24, 27). In addition, the peptide APTRVVAAEM is involved in cross-protection against DENV and ZIKV. The epitopes TDTTPFGQQRVFKEK, EFGKAKGSRAIWYMW, and AKGSRAIWYMWLGAR are concentrated in the NS5 protein and contain multiple HLA-I and -II binding motifs which are involved in elicitation of protective immune memory against YFV (40). The epitope MTTEDMLQVW is involved in protection against JEV (43). The remaining selected epitopes (see Table 2) were also related to protection against DENV. All of the selected epitopes are involved in induction of anti-flavivirus CD8^+^ T cells. Some of the listed studies report generation os memory cells, which could also be recalled in order to favor cross-protective immunity.

Conserved epitopes could be enriched in artificial or natural immunizations. For example, sequential infection or administration of different anti-flavivirus vaccines would recall cross-reactive memory T CD4^+^ and T CD8^+^ lymphocytes. It is important to stress that approved vaccines represent three serocomplexes: DENV, JEV and YFV. However, most of those epitopes which are important for recalling cross-reactive T cells, are concentrated in non-structural proteins. The only anti-DENV vaccine currently approved for use in humans is a chimera between DENV and YFV. Its structural antigens are representative of DENV proteins, while non-structural antigens are representative of YFV proteins. In addition, the JEV vaccine is based on inactivated viruses. Enhancing the recall of cross-protective memory T cells would ideally happen with the use of live attenuated viruses or other vaccine approach capable of providing epitopes of non-structural proteins.

## Data Availability Statement

All datasets generated for this study are included in the manuscript/Supplementary Files.

## Author Contributions

JA conceived the study and wrote the paper. LS, LG, and WL analyzed data and wrote the paper.

### Conflict of Interest Statement

The authors declare that the research was conducted in the absence of any commercial or financial relationships that could be construed as a potential conflict of interest.
