# Supplementary material for: Seeking Flavivirus Cross-Protective Immunity
Source: Front Immunol. 2019 Sep 20;10:2260. doi: 10.3389/fimmu.2019.02260 (PMC6763598; doi:10.3389/fimmu.2019.02260)
Supplement: Supplementary file 1 [file Data_Sheet_1.docx]

>DENV1_AHI43749_Puerto_Rico

MNNQRKKTGRPSFNMLKRARNRVSTGSQLAKRFSKGLLSGQGPMKLVMAFIAFLRFLAIPPTAGILARWSSFKKNGAIKVLRGFKKEISSMLNIMNRRKRSVTMLLMLLPTALAFHLTTRGGEPHMIVSKQERGKSLLFKTSAGVNMCTLIAMDLGELCEDTMTYKCPRIAEAEPDDVDCWCNATDTWVTYGTCSQTGEHRREKRSVALAPHVGLGLETRTETWMSSEGAWKQIQRVETWALRHPGFTVIAFFLAHAIGTSITQKGIIFILLMLVTPSMAMRCVGIGNRDFVEGLSGATWVDVVLEHGSCVTTMAKNKPTLDIELLKTEVTNPAVLRKLCIEAKISNTTTDSRCPTQGEATLVEEQDANFVCRRTFVDRGWGNGCGLFGKGSLLTCAKFKCVTKLEGKIVQYENLKYSVIVTVHTGDQHQVGNETTEHGTIATITPQAPTSEIQLTDYGALTLDCSPRTGLDFNEMVLLTMKEKSWLVHKQWFLDLPLPWTSGASTSQETWNRQDLLVTFKTAHAKKQEVVVLGSQEGAMHTALTGATEIQTSGTTTIFAGHLKCRLKMDKLTLKGMSYVMCTGSFKLEKEVAETQHGTVLVQVKYEGTDAPCKIPFSTQDEKGVTQNGRLITANPIVTDKEKPVNIETEPPFGESYIVVGAGEKALKLSWFKRGSSIGKMFEATARGARRMAILGDTAWDFGSIGGVFTSVGKLVHQIFGTAYGVLFSGVSWTMKIGIGILLTWLGLNSRSTSLSMTCIAVGMVTLYLGVMVQADSGCVINWKGRELKCGSGIFVTNEVHTWTEQYKFQADSPKRLSAAIGKAWEEGVCGIRSATRLENIMWKQISNELNHILLENDMKFTVVVGDANGILAQGKKMIRPQPMEHKYSWKSWGKAKIIGADIQNTTFIIDGPDTPECPDGQRAWNIWEVEDYGFGVFTTNIWLKLRDSYTQMCDHRLMSAAIKDSKAVHADMGYWIESEKNETWKLARASFIEVKTCTWPKSHTLWSNGVLESEMIIPKIYGGPISQHNYRPGYFTQTAGPWHLGKLELDFDLCEGTTVVVDEHCGNRGPSLRTTTVTGKIIHEWCCRSCTLPPLRFRGEDGCWYGMEIRPVKEKEENLVRSMVSAGSGEVDSFSLGILCVSIMIEEVMRSRWSRKMLMTGTLAVFLLLIMGQLTWNDLIRLCIMVGANASDRMGMGTTYLALMATFKMRPMFAVGLLFRRLTSREVLLLTIGLSLVASVELPNSLEELGDGLAMGIMMLKLLTEFQPHQLWTTLLSLTFVKTTLSLDYAWKTTAMALSIVSLFPLCLSTTSQKTTWLPVLLGSFGCKPLTMFLITENKIWGRKSWPLNEGIMAIGIVSILLSSLLKNDVPLAGPLIAGGMLIACYVISGSSADLSLEKAAEVSWEQEAEHSGASHSILVEVQDDGTMKIKDEERDDTLTILLKATLLAVSGVYPMSIPATLFVWYFWQKKKQRSGVLWDTPSPPEVERAVLDNGIYRILQRGLLGRSQVGVGVFQDGVFHTMWHVTRGAVLMYQGKRLEPSWASVKKDLISYGGGWRFQGSWNTGEEVQVIAVEPGKNPKNVQTTPGTFKTPEGEVGAIALDFKPGTSGSPIVNREGKIVGLYGNGVVTTSGTYVSAIAQAKASQEGPLPEIEDEVFKKRNLTIMDLHPGSGKTRRYLPAIVREAIKRKLRTLILAPTRVVASEMAEALKGMPIRYQTTAVKSEHTGREIVDLMCHATFTMRLLSPVRVPNYNMIIMDEAHFTDPASIAARGYISTRVGMGEAAAIFMTATPPGSVEAFPQSNAVIQDEERDIPERSWNSGYDWITDFPGKTVWFVPSIKSGNDIANCLRKNGKRVIQLSRKTFDTEYQKTKNSDWDYVVTTDISEMGANFRADRVIDPRRCLKPVILKDGPERVILAGPMPVTAASAAQRRGRIGRNQNKEGDQYVYMGQPLNNDEDHAHWTEAKMLLDNINTPEGIIPALFEPEREKSAAIDGEYRLRGEARKTFVELMRRGDLPVWLSYKVASEGFQYSDRRWCFDGERNNQVLEENMDVEIWTKEGERKKLRPRWLDARTYSDPLALREFKEFAAGRRSVSGDLILEIGKLPQHLTLRAQNALDNLVMLHNSEQGGKAYRHAMEELPDTIETLMLLALIAVLTGGVTLFFLSGKGLGKTSIGLLCVTASSALLWMASVEPHWIAASIILEFFLMVLLIPEPDRQRTPQDNQLAYVVIGLLFMILTVAANEMGLLETTKKDLGIGYVAAENHQHVTMLDVDLHPASAWTLYAVATTVITPMMRHTIENATANISLTAIANQAAILMGLDKGWPISKMDIGVPLLALGCYSQVNPLTLTAAVLMLVAHYAIIGPGLQAKATREAQKRTAAGIMKNPTVDGIVAIDLDPVVYDAKFEKQLGQIMLLILCTSQILLMRTTWALCESITLATGPLTTLWEGSPGKFWNTTIAVSMANIFRGSYLAGAGLAFSLMKSLGGGRRGTGAQGETLGEKWKRQLNQLSKSEFNTYKRSGIMEVDRSEAKEGLKRGETTKHAVSRGTAKLRWFVERNLVKPEGKVIDLGCGRGGWSYYCAGLKKVTEVKGYTKGGPGHEEPIPMATYGWNLVKLHSGKDVFFMPPEKCDTLLCDIGESSPNPTVEEGRTLRVLKMVEPWLRGNQFCIKILNPYMPSVVETLERMQRKHGGMLVRNPLSRNSTHEMYWVSCGTGNIVSAVNMTSRMLLNRFTMAHRKPTYERDVDLGAGTRHVAVEPEVANLDIIGQRIENIKNEHKSTWHYDEDNPYKTWAYHGSYEVKPSGSASSMVNGVVRLLTKPWDVIPMVTQIAMTDTTPFGQQRVFKEKVDTRTPRAKRGTTQIMEVTAKWLWGFLSRNKKPRICTREEFTRKVRSNAAVGAVFVDENQWNSAKEAVEDERFWDLVHRERELHKQGKCATCVYNMMGKREKKLGEFGKAKGSRAIWYMWLGARFLEFEALGFMNEDHWFSRENSLSGVEGEGLHKLGYILRDISKIPGGNMYADDTAGWDTRVTEDDLQNEAKITDIMEPEHALLATSIFKLTYQNKVVRVQRPAKNGTVMDVISRRDQRGSGQVGTYGLNTFTNMEVQLIRQMESEGIFLPSELETPNLAERALDWLEKHGAERLKRMAISGDDCVVKPIDDRFATALTALNDMGKVRKDIPQWEPSKGWNDWQQVPFCSHHFHQLIMKDGREIVVPCRNQDELVGRARVSQGAGWSLRETACLGKSYAQMWQLMYFHRRDLRLAANAICSAVPVDWVPTSRTTWSIHAHHQWMTTEDMLSVWNRVWIEENPWMENKTHVSSWEEVPYLGKREDQWCGSLIGLTARATWATNIQVAINQVRRLIGNENYLDYMTSMKRFKNESDSEGALW

>DENV1_AHI43750_Puerto_Rico

MNNQRKKTGRPSFNMLKRARNRVSTGSQLAKRFSKGLLSGQGPMKLVMAFIAFLRFLAIPPTAGILARWSSFKKNGAIKVLRGFKKEISSMLNIMNRRKRSVTMLLMLLPTALAFHLTTRGGEPHMIVSKQERGKSLLFKTSAGVNMCTLIAMDLGELCEDTMTYKCPRITEAEPDDVDCWCNATDTWVTYGTCSQTGEHRREKRSVALAPHVGLGLETRTETWMSSEGAWKQIQRVETWALRHPGFTVIAFFLAHAIGTSITQKGIIFILLMLVTPSMAMRCVGIGNRDFVEGLSGATWVDVVLEHGSCVTTMAKNKPTLDIELLKTEVTNPAVLRKLCIEAKISNTTTDSRCPTQGEATLVEEQDANFVCRRTFVDRGWGNGCGLFGKGSLLTCAKFKCVTKLEGKIVQYENLKYSVIVTVHTGDQHQVGNETTEHGTIATITPQAPTSEIQLTDYGALTLDCSPRTGLDFNEMVLLTMKEKSWLVHKQWFLDLPLPWTSGASTSQETWNRQDLLVTFKTAHAKKQEVVVLGSQEGAMHTALTGATEIQTSGTTTIFAGHLKCRLKMDKLTLKGMSYVMCTGSFKLEKEVAETQHGTVLVQVKYEGTDAPCKIPFSTQDEKGVTQNGRLITANPIVTDKEKPVNIETEPPFGESYIVVGAGEKALKLSWFKRGSSIGKMFEATARGARRMAILGDTAWDFGSIGGVFTSVGKLVHQIFGTAYGVLFSGVSWTMKIGIGILLTWLGLNSRSTSLSMTCIVVGMVTLYLGVMVQADSGCVINWKGRELKCGSGIFVTNEVHTWTEQYKFQADSPKRLSAAIGKAWEEGVCGIRSATRLENIMWKQISNELNHILLENDMKFTVVVGDANGILAQGKKMIRPQPMEHKYSWKSWGKAKIIGADIQNTTFIIDGPDTPECPDGQRAWNIWEVEDYGFGVFTTNIWLKLRDSYTQMCDHRLMSAAIKDSKAVHADMGYWIESEKNETWKLARASFIEVKTCTWPKSHTLWSNGVLESEMIIPKIYGGPISQHNYRPGYFTQTAGPWHLGKLELDFDLCEGTTVVVDEHCGNRGPSLRTTTVTGKIIHEWCCRSCTLPPLRFRGEDGCWYGMEIRPVKEKEENLVRSMVSAGSGEVDSFSLGILCVSIMIEEVMRSRWSRKMLMTGTLAVFLLLIMGQLTWNDLIRLCIMVGANASDRMGMGTTYLALMATFKMRPMFAVGLLFRRLTSREVLLLTIGLSLVASVELPNSLEELGDGLAMGIMMLKLLTEFQPHQLWTTLLSLTFVKTTLSLDYAWKTTAMALSIVSLFPLCLSTTSQKTTWLPVLLGSFGCKPLTMFLITENKIWGRKSWPLNEGIMAIGIVSILLSSLLKNDVPLAGPLIAGGMLIACYVISGSSADLSLEKAAEVSWEQEAEHSGASHSILVEVQDDGTMKIKDEERDDTLTILLKATLLAVSGVYPMSIPATLFVWYFWQKKKQRSGVLWDTPSPPEVERAVLDNGIYRILQRGLLGRSQVGVGVFQDGVFHTMWHVTRGAVLMYQGKRLEPSWASVKKDLISYGGGWRFQGSWNTGEEVQVIAVEPGKNPKNVQTTPGTFKTPEGEVGAIALDFKPGTSGSPIVNREGKIVGLYGNGVVTTSGTYVSAIAQAKASQEGPLPEIEDEVFKKRNLTIMDLHPGSGKTRRYLPAIVREAIKRKLRTLILAPTRVVASEMAEALKGMPIRYQTTAVKSEHTGREIVDLMCHATFTMRLLSPVRVPNYNMIIMDEAHFTDPASIAARGYISTRVGMGEAAAIFMTATPPGSVEAFPQSNAVIQDEERDIPERSWNSGYDWITDFPGKTVWFVPSIKSGNDIANCLRKNGKRVIQLSRKTFDTEYQKTKNNDWDYVVTTDISEMGANFRADRVIDPRRCLKPVILKDGPERVILAGPMPVTAASAAQRRGRIGRNQNKEGDQYVYMGQPLNNDEDHAHWTEAKMLLDNINTPEGIIPALFEPEREKSAAIDGEYRLRGEARKTFVELMRRGDLPVWLSYKVASEGFQYSDRRWCFDGERNNQVLEENMDVEIWTKEGERKKLRPRWLDARTYSDPLALREFKEFAAGRRSVSGDLILEIGKLPQHLTLRAQNALDNLVMLHNSEQGGKAYRHAMEELPDTIETLMLLALIAVLTGGVTLFFLSGKGLGKTSIGLLCVTASSALLWMASVEPHWIAASIILEFFLMVLLIPEPDRQRTPQDNQLAYVVIGLLFMILTVAANEMGLLETTKKDLGIGYVAAENHQHATMLDVDLHPASAWTLYAVATTVITPMMRHTIENTTANISLTAIANQAAILMGLDKGWPISKMDIGVPLLALGCYSQVNPLTLTAAVLMLVAHYAIIGPGLQAKATREAQKRTAAGIMKNPTVDGIVAIDLDPVVYDAKFEKQLGQIMLLILCTSQILLMRTTWALCESITLATGPLTTLWEGSPGKFWNTTIAVSMANIFRGSYLAGAGLAFSLMKSLGGGRRGTGAQGETLGEKWKRQLNQLSKSEFNTYKRSGIMEVDRSEAKEGLKRGETTKHAVSRGTAKLRWFVERNLVKPEGKVIDLGCGRGGWSYYCAGLKKVTEVKGYTKGGPGHEEPIPMATYGWNLVKLHSGKDVFFMPPEKCDTLLCDIGESSPNPTIEEGRTLRVLKMVEPWLRGNQFCIKILNPYMPSVVETLERMQRKHGGMLVRNPLSRNSTHEMYWVSCGTGNIVSAVNMTSRMLLNRFTMAHRKPTYERDVDLGAGTRHVAVEPEVANLDIIGQRIENIKNEHKSTWHYDEDNPYKTWAYHGSYEVKPSGSASSMVNGVVRLLTKPWDVIPMVTQIAMTDTTPFGQQRVFKEKVDTRTPRAKRGTTQIMEVTAKWLWGFLSRNKKPRICTREEFTRKVRSNAAIGAVFVDENQWNSAKEAVEDERFWDLVHRERELHKQGKCATCVYNMMGKREKKLGEFGKAKGSRAIWYMWLGARFLEFEALGFMNEDHWFSRENSLSGVEGEGLHKLGYILRDISKIPGGNMYADDTAGWDTRVTEDDLQNEAKITDIMEPEHALLATSIFKLTYQNKVVRVQRPAKNGTVMDVISRRDQRGSGQVGTYGLNTFTNMEVQLIRQMESEGIFLPSELETPNLAERALDWLEKHGAERLKRMAISGDDCVVKPIDDRFATALTALNDMGKVRKDIPQWEPSKGWNDWQQVPFCSHHFHQLIMKDGREIVVPCRNQDELVGRARVSQGAGWSLRETACLGKSYAQMWQLMYFHRRDLRLAANAICSAVPVDWVPTSRTTWSIHAHHQWMTTEDMLSVWNRVWIDENPWMENKTHVSSWEEVPYLGKREDQWCGSLIGLTARATWATNIQVAINQVRRLIGNENYLDYMTSMKRFKNESDSEGALW

>DENV1_AHI43748_Puerto_Rico

MNNQRKKTGRPSFNMLKRARNRVSTGSQLAKRFSKGLLSGQGPMKLVMAFIAFLRFLAIPPTAGILARWSSFKKNGAIKVLRGFKKEISSMLNIMNRRKRSVTMLLMLLPTALAFHLTTRGGEPHMIVSKQERGKSLLFKTSAGVNMCTLIAMDLGELCEDTMTYKCPRITEAEPDDVDCWCNATDTWVTYGTCSQTGEHRREKRSVALAPHVGLGLETRTETWMSSEGAWKQIQRVETWALRHPGFTVIAFFLAHAIGTSITQKGIIFILLMLVTPSMAMRCVGIGNRDFVEGLSGATWVDVVLEHGSCVTTMAKNKPTLDIELLKTEVTNPAVLRKLCIEAKISNTTTDSRCPTQGEATLVEEQDANFVCRRTFVDRGWGNGCGLFGKGSLLTCAKFKCVTKLEGKIVQYENLKYSVIVTVHTGDQHQVGNETTEHGTIATITPQAPTSEIQLTDYGALTLDCSPRTGLDFNEMVLLTMKEKSWLVHKQWFLDLPLPWTSGASTSQETWNRQDLLVTFKTAHAKKQEVVVLGSQEGAMHTALTGATEIQTSGTTTIFAGHLKCRLKMDKLTLKGMSYVMCTGSFKLEKEVAETQHGTVLVQVKYEGTDAPCKIPFSTQDEKGVTQNGRLITANPIVTDKEKPVNIETEPPFGESYIVVGAGEKALKLSWFKRGSSIGKMFEATARGARRMAILGDTAWDFGSIGGVFTSVGKLVHQIFGTAYGVLFSGVSWTMKIGIGILLTWLGLNSRSTSLSMTCIVVGMVTLYLGVMVQADSGCVINWKGRELKCGSGIFVTNEVHTWTEQYKFQADSPKRLSAAIGKAWEEGVCGIRSATRLENIMWKQISNELNHILLENDMKFTVVVGDANGILAQGKKMIRPQPMEHKYSWKSWGKAKIIGADIQNTTFIIDGPDTPECPDGQRAWNIWEVEDYGFGVFTTNIWLKLRDSYTQMCDHRLMSAAIKDSKAVHADMGYWIESEKNETWKLARASFIEVKTCTWPKSHTLWSNGVLESEMIIPKIYGGPISQHNYRPGYFTQTAGPWHLGKLELDFDLCEGTTVVVDEHCGNRGPSLRTTTVTGKIIHEWCCRSCTLPPLRFRGEDGCWYGMEIRPVKEKEENLVRSMVSAGSGEVDSFSLGILCVSIMIEEVMRSRWSRKMLMTGTLAVFLLLIMGQLTWNDLIRLCIMVGANASDRMGMGTTYLALMATFKMRPMFAVGLLFRRLTSREVLLLTIGLSLVASVELPNSLEELGDGLAMGIMMLKLLTEFQPHQLWTTLLSLTFVKTTLSLDYAWKTTAMALSIVSLFPLCLSTTSQKTTWLPVLLGSFGCKPLTMFLITENKIWGRKSWPLNEGIMAIGIVSILLSSLLKNDVPLAGPLIAGGMLIACYVISGSSADLSLEKAAEVSWEQEAEHSGASHSILVEVQDDGTMKIKDEERDDTLTILLKATLLAVSGVYPMSIPATLFVWYFWQKKKQRSGVLWDTPSPPEVERAVLDNGIYRILQRGLLGRSQVGVGVFQDGVFHTMWHVTRGAVLMYQGKRLEPSWASVKKDLISYGGGWRFQGSWNTGEEVQVIAVEPGKNPKNVQTTPGTFKTPEGEVGAIALDFKPGTSGSPIVNREGKIVGLYGNGVVTTSGTYVSAIAQAKASQEGPLPEIEDEVFKKRNLTIMDLHPGSGKTRRYLPAIVREAIKRKLRTLILAPTRVVASEMAEALKGMPIRYQTTAVKSEHTGREIVDLMCHATFTMRLLSPVRVPNYNMIIMDEAHFTDPASIAARGYISTRVGMGEAAAIFMTATPPGSVEAFPQSNAVIQDEERDIPERSWNSGYDWITDFPGKTVWFVPSIKSGNDIANCLRKNGKRVIQLSRKTFDTEYQKTKNNDWDYVVTTDISEMGANFRADRVIDPRRCLKPVILKDGPERVILAGPMPVTAASAAQRRGRIGRNQNKEGDQYVYMGQPLNNDEDHAHWTEAKMLLDNINTPEGIIPALFEPEREKSAAIDGEYRLRGEARKTFVELMRRGDLPVWLSYKVASEGFQYSDRRWCFDGERNNQVLEENMDVEIWTKEGERKKLRPRWLDARTYSDPLALREFKEFAAGRRSVSGDLILEIGKLPQHLTLRAQNALDNLVMLHNSEQGGKAYRHAMEELPDTIETLMLLALIAVLTGGVTLFFLSGKGLGKTSIGLLCVTASSALLWMASVEPHWIAASIILEFFLMVLLIPEPDRQRTPQDNQLAYVVIGLLFMILTVAANEMGLLETTKKDLGIGYVAAENHQHATMLDVDLHPASAWTLYAVATTVITPMMRHTIENTTANISLTAIANQAAILMGLDKGWPISKMDIGVPLLALGCYSQVNPLTLTAAVLMLVAHYAIIGPGLQAKATREAQKRTAAGIMKNPTVDGIVAIDLDPVVYDAKFEKQLGQIMLLILCTSQILLMRTTWALCESITLATGPLTTLWEGSPGKFWNTTIAVSMANIFRGSYLAGAGLAFSLMKSLGGGRRGTGAQGETLGEKWKRQLNQLSKSEFNTYKRSGIMEVDRSEAKEGLKRGETTKHAVSRGTAKLRWFVERNLVKPEGKVIDLGCGRGGWSYYCAGLKKVTEVKGYTKGGPGHEEPIPMATYGWNLVKLHSGKDVFFMPPEKCDTLLCDIGESSPNPTIEEGRTLRVLKMVEPWLRGNQFCIKILNPYMPSVVETLERMQRKHGGMLVRNPLSRNSTHEMYWVSCGTGNIVSAVNMTSRMLLNRFTMAHRKPTYERDVDLGAGTRHVAVEPEVANLDIIGQRIENIKNEHKSTWHYDEDNPYKTWAYHGSYEVKPSGSASSMVNGVVRLLTKPWDVIPMVTQIAMTDTTPFGQQRVFKEKVDTRTPRAKRGTTQIMEVTAKWLWGFLSRNKKPRICTREEFTRKVRSNAAIGAVFVDENQWNSAKEAVEDERFWDLVHRERELHKQGKCATCVYNMMGKREKKLGEFGKAKGSRAIWYMWLGARFLEFEALGFMNEDHWFSRENSLSGVEGEGLHKLGYILRDISKIPGGNMYADDTAGWDTRVTEDDLQNEAKITDIMEPEHALLATSIFKLTYQNKVVRVQRPAKNGTVMDVISRRDQRGSGQVGTYGLNTFTNMEVQLIRQMESEGIFLPSELETPNLAERALDWLEKHGAERLKRMAISGDDCVVKPIDDRFATALTALNDMGKVRKDIPQWEPSKGWNDWQQVPFCSHHFHQLIMKDGREIVVPCRNQDELVGRARVSQGAGWSLRETACLGKSYAQMWQLMYFHRRDLRLAANAICSAVPVDWVPTSRTTWSIHAHHQWMTTEDMLSVWNRVWIDENPWMENKTHVSSWEEVPYLGKREDQWCGSLIGLTARATWATNIQVAINQVRRLIGNENYLDYMTSMKRFKNESDSEGALW

>DENV1_AHI43747_Puerto_Rico

MNNQRKKTGRPSFNMLKRARNRVSTGSQLAKRFSKGLLSGQGPMKLVMAFIAFLRFLAIPPTAGILARWSSFKKNGAIKVLRGFKKEISSMLNIMNRRKRSVTMLLMLLPTALAFHLTTRGGEPHMIVSKQERGKSLLFKTSAGVNMCTLIAMDLGELCEDTMTYKCPRITEAEPDDVDCWCNATDTWVTYGTCSQTGEHRREKRSVALAPHVGLGLETRTETWMSSEGAWKQIQRVETWALRHPGFTVIAFFLAHAIGTSITQKGIIFILLMLVTPSMAMRCVGIGNRDFVEGLSGATWVDVVLEHGSCVTTMAKNKPTLDIELLKTEVTNPAVLRKLCIEAKISNTTTDSRCPTQGEATLVEEQDANFVCRRTFVDRGWGNGCGLFGKGSLLTCAKFKCVTKLEGKIVQYENLKYSVIVTVHTGDQHQVGNETTEHGTIATITPQAPTSEIQLTDYGALTLDCSPRTGLDFNEMVLLTMKEKSWLVHKQWFLDLPLPWTSGASTSQETWNRQDLLVTFKTAHAKKQEVVVLGSQEGAMHTALTGATEIQTSGTTTIFAGHLKCRLKMDKLTLKGMSYVMCTGSFKLEKEVAETQHGTVLVQVKYEGTDAPCKIPFSTQDEKGMTQNGRLITANPIVTDKEKPVNIETEPPFGESYIVVGAGEKALKLSWFKRGSSIGKMFEATARGARRMAILGDTAWDFGSIGGVFTSVGKLVHQIFGTAYGVLFSGVSWTMKIGIGILLTWLGLNSRSTSLSMTCIAVGMVTLYLGVMVQADSGCVINWKGRELKCGSGIFVTNEVHTWTEQYKFQADSPKRLSAAIGKAWEEGVCGIRSATRLENIMWKQISNELNHILLENDMKFTVVVGDANGILAQGKKMIRPQPMEHKYSWKSWGKAKIIGADIQNTTFIIDGPDTPECPDGQRAWNIWEVEDYGFGVFTTNIWLKLRDSYTQMCDHRLMSAAIKDSKAVHADMGYWIESEKNETWKLARASFIEVKTCTWPKSHTLWSNGVLESEMIIPKIYGGPISQHNYRPGYFTQTAGPWHLGKLELDFDLCEGTTVVVDEHCGNRGPSLRTTTVTGKIIHEWCCRSCTLPPLRFRGEDGCWYGMEIRPVKEKEENLVRSMVSAGSGEVDSFSLGILCVSIMIEEVMRSRWSRKMLMTGTLAVFLLLIMGQLTWNDLIRLCIMVGANASDRMGMGTTYLALMATFKMRPMFAVGLLFRRLTSREVLLLTIGLSLVASVELPNSLEELGDGLAMGIMMLKLLTEFQPHQLWTTLLSLTFVRTTLSLDYAWKTTAMALSIVSLFPLCLSTTSQKTTWLPVLLGSFGCKPLTMFLITENKIWGRKSWPLNEGIMAIGIVSILLSSLLKNDVPLAGPLIAGGMLIACYVISGSSADLSLEKAAEVSWEQEAEHSGASHSILVEVQDDGTMKIKDEERDDTLTILLKATLLAVSGVYPMSIPATLFVWYFWQKKKQRSGVLWDTPSPPEVERAVLDNGIYRILQRGLLGRSQVGVGVFQDGVFHTMWHVTRGAVLMYQGKRLEPSWASVKKDLISYGGGWRFQGSWNTGEEVQVIAVEPGKNPKNVQTTPGTFKTPEGEVGAIALDFKPGTSGSPIVNREGKIVGLYGNGVVTTSGTYVSAIAQAKASQEGPLPEIEDEVFKKRNLTIMDLHPGSGKTRRYLPAIVREAIKRKLRTLILAPTRVVASEMAEALKGMPIRYQTTAVKSEHTGREIVDLMCHATFTMRLLSPVRVPNYNMIIMDEAHFTDPASIAARGYISTRVGMGEAAAIFMTATPPGSVEAFPQSNAVIQDEERDIPERSWNSGYDWITDFPGKTVWFVPSIKSGNDIANCLRKNGKRVIQLSRKTFDTEYQKTKNNDWDYVVTTDISEMGANFRADRVIDPRRCLKPVILKDGPERVILAGPMPVTAASAAQRRGRIGRNQNKEGDQYVYMGQPLNNDEDHAHWTEAKMLLDNINTPEGIIPALFEPEREKSAAIDGEYRLRGEARKTFVELMRRGDLPVWLSYKVASEGFQYSDRRWCFDGERNNQVLEENMDVEIWTKEGERKKLRPRWLDARTYSDPLALREFKEFAAGRRSVSGDLILEIGKLPQHLTLRAQNALDNLVMLHNSEQGGKAYRHAMEELPDTIETLMLLALIAVLTGGVTLFFLSGKGLGKTSIGLLCVTASSALLWMASVEPHWIAASIILEFFLMVLLIPEPDRQRTPQDNQLAYVVIGLLFMILTVAANEMGLLETTKKDLGIGYVAAENHQHATMLDVDLHPASAWTLYAVATTVITPMMRHTIENTTANISLTAIANQAAILMGLDKGWPISKMDIGVPLLALGCYSQVNPLTLTAAVLMLVAHYAIIGPGLQAKATREAQKRTAAGIMKNPTVDGIVAIDLDPVVYDAKFEKQLGQIMLLILCTSQILLMRTTWALCESITLATGPLTTLWEGSPGKFWNTTIAVSMANIFRGSYLAGAGLAFSLMKSLGGGRRGTGAQGETLGEKWKRQLNQLSKSEFNTYKRSGIMEVDRSEAKEGLKRGETTKHAVSRGTAKLRWFVERNLVKPEGKVIDLGCGRGGWSYYCAGLKKVTEVKGYTKGGPGHEEPIPMATYGWNLVKLHSGKDVFFMPPEKCDTLLCDIGESSPNPTIEEGRTLRVLKMVEPWLRGNQFCIKILNPYMPSVVETLERMQRKHGGMLVRNPLSRNSTHEMYWVSCGTGNIVSAVNMTSRMLLNRFTMAHRKPTYERDVDLGAGTRHVAVEPEVANLDIIGQRIENIKNEHKSTWHYDEDNPYKTWAYHGSYEVKPSGSASSMVNGVVRLLTKPWDVIPMVTQIAMTDTTPFGQQRVFKEKVDTRTPRAKRGTTQIMEVTAKWLWSFLSRNKKPRICTREEFTRKVRSNAAIGAVFVDENQWNSAKEAVEDERFWDLVHRERELHKQGKCATCVYNMMGKREKKLGEFGKAKGSRAIWYMWLGARFLEFEALGFMNEDHWFSRENSLSGVEGEGLHKLGYILRDISKIPGGNMYADDTAGWDTRVTEDDLQNEAKITDIMEPEHALLATSIFKLTYQNKVVRVQRPAKNGTVMDVISRRDQRGSGQVGTYGLNTFTNMEVQLIRQMESEGIFLPSELETPNLAERALDWLEKHGAERLKRMAISGDDCVVKPIDDRFATALTALNDMGKVRKDIPQWEPSKGWNDWQQVPFCSHHFHQLIMKDGREIVVPCRNQDELVGRARVSQGAGWSLRETACLGKSYAQMWQLMYFHRRDLRLAANAICSAVPVDWVPTSRTTWSIHAHHQWMTTEDMLSVWNRVWIEENPWMENKTHVSSWEEVPYLGKREDQWCGSLIGLTARATWATNIQVAINQVRRLIGNENYLDYMTSMKRFKNESDSEGALW

>DENV1_AHC98446_Nicaragua

MNNQRKKTGRPSFNMLKRARNRVSTGSQLAKRFSKGLLSGQGPMKLVMAFIAFLRFLAIPPTAGILARWSSFKKNGAIKVLRGFKKEISSMLNIMNRRKRSVTMLLMLMPTALAFHLTTRGGEPHMIVSKHERGKALLFKTSTGVNMCTLIAMDLGELCEDTMTYKCPRITEAEPDDVDCWCNATDTWVTYGTCSQTGEHRRDKRSVALAPHVGLGLETRTETWMSSEGAWKQIQRVETWALRHPGFTVIALFLAHAIGTSITQKGIIFILLMLVTPSMAMRCVGIGNRDFVEGLSGATWVDVVLEHGSCVTTMAKNKPTLDIELLKTEVTNPAVLRKLCIEAKISNTTTDSRCPTQGEATLVEEQDANFVCRRTFVDRGWGNGCGLFGKGSLLTCAKFKCVTKLEGKIVQYENLKYSVIVTVHTGDQHQVGNETTEHGTIATITPQAPTSEIQLTDYGALTLDCSPRTGLDFNEMVLLTMKEKSWLVHKQWFLDLPLPWTSGASTSQETWNRQDLLVTFKTAHAKKQEVVVLGSQEGAMHTALTGATEIQTSGTTTIFAGHLKCRLKMDKLTLKGTSYVMCTGSFKLEKEVAETQHGTVLVQVKYEGTDAPCKIPFSTQDEKGVTQNGRLITANPIVTDKEKPVNIETEPPFGESYIVVGAGEKALKLSWFKKGSSIGKMFEATARGARRMAILGDTAWDFGSIGGVFTSVGKLVHQVFGTAYGVLFSGVSWTMKIGIGILLTWLGLNSRSTSLSMTCIAVGMVTLYLGVMVQADSGCVINWKGRELKCGSGIFVTNEVHTWTEQYKFQADSPKRLSAAIGKAWEEGVCGIRSATRLENIMWKQISNELNHILLENGMKFTVVVGDANGILAQGKKMIRPQPMEHKYSWKSWGKAKIIGADIQNTTFIIDGPDTPECPDDQRAWNIWEVEDYGFGIFTTNIWLKLRDSYTQMCDHRLMSAAIKDSKAVHADMGYWIESEKNETWKLARASFIEVKTCTWPKSHTLWSNGVLESEMIIPKIYGGPISQHNYRPGYFTQTAGPWHLGKLELDFDLCEGTTVVVDEHCGNRGPSLRTTTVTGKIIHEWCCRSCTLPPLRFRGEDGCWYGMEIRPVKEKEENLVRSMVSAGSGEVDSFSLGILCVSIMIEEVMRSRWSRKMLMTGTLAVFLLLIMGQLTWNDLIRLCIMVGANASDRMGMGTTYLALMATFKMRPMFAVGILFRRLTSREVLLLTIGLSLVASVELPNSLEELGDGLAMGIMMLKLLTEFQPHQLWTTLLSLTFIKTTLSLDYAWKTTAMALSIVSLFPLCLSTTSQKTTWLPVLLGSFGCKPLTMFLITENKIWGRKSWPINEGIMAIGIVSILLSSLLKNDVPLAGPLIAGGMLIACYVISGSSADLSLEKAAEVSWEQEAEHSGTSHNILVEVQDDGTMRIKDEEKDDTLTILLKATLLAVSGVYPMSIPATLFVWYFWQKKKQRSGVLWDTPSPPEVERAVLDDGIYRILQRGLLGRSQVGVGVFQDGVFHTMWHVTRGAVLMYQGKRLEPSWASVKKDLISYGGGWRFQGSWNTGEEVQVIAVEPGKNPKNVQTTPGTFKTSEGEVGAIALDFKPGTSGSPIVNREGKIVGLYGNGVVTTSGTYVSAIAQAKTSQEGPLPEIEDEVFKKRNLTIMDLHPGSGKTRRYLPAIVREAIKRKLRTLILAPTRVVASEMAEALKGMPIRYQTTAVKSEHTGREIVDLMCHATFTMRLLSPVRVPNYNMIIMDEAHFTDPASIAARGYISTRVGMGEAAAIFMTATPPGSVEAFPQSNAVIQDEERDIPERSWNSGYDWITDFPGKTVWFVPSIKSGNDIANCLRKNGKRVIQLSRKTFDTEYQKTKNNDWDYVVTTDISEMGANFRADRVIDPRRCLKPVILKDGPERVILAGPMPVTVASAAQRRGRIGRNQNKEGDQYVYMGQPLNNDEDHAHWTEAKMLLDNINTPEGIIPALFEPEREKSAAIDGEYRLRGEARKTFVELMRRGDLPVWLSYKVASEGFQYSDRRWCFDGERNNQVLEENMDVEIWTKEGERKKLRPRWLDARTYSDPLALREFKEFAAGRRSVSGDLILEIGKLPQHLTLRAQNALDNLVMLHNSEQGGKAYRHAMEELPDTIETLMLLALIAVLTGGVTLFFLSGKGLGKTSIGLLCVTASSALLWMASVEPHWIAASIILEFFLMVLLIPEPDRQRTPQDNQLAYVVIGLLFMILTVAANEMGLLETTKRDLGIGHVAAENHQHATMLDVDLRPASAWTLYAVATTVITPMMRHTIENTTANISLTAIANQAAILMGLDKGWPISKMDIGVPLLALGCYSQVNPLTLTAAVLMLVAHYAIIGPGLQAKATREAQKRTAAGIMKNPTVDGIVAIDLDPVVYDAKFEKQLGQIMLLILCTSQILLMRTTWALCESITLATGPLTTLWEGSPGKFWNTTIAVSMANIFRGSYLAGAGLAFSLMKSLGGGRRGTGAQGETLGEKWKRQLNQLSKPEFNTYKRSGIMEVDRSEAKEGLKRGETTKHAVSRGTAKLRWFVERNLVKPEGKVIDLGCGRGGWSYYCAGLKKVTEVKGYTKGGPGHEEPIPMATYGWNLVKLHSGKDVFFMPPEKCDTLLCDIGESSPNPTIEEGRTLRVLKMVEPWLRGNQFCIKILNPYMPSVVETLEQMQRKHGGMLVRNPLSRNSTHEMYWVSCGTGNIVSAVNMTSRMLLNRFTMAHRKPTYERDVDLGAGTRHVAVEPEVANLDIIGQRIENIKNEHKSTWHYDEDNPYKTWAYHGSYEVKPSGSASSMVNGVVRLLTKPWDVIPMVTQIAMTDTTPFGQQRVFKEKVDTRTPRAKRGTTQIMEVTAKWLWGFLSRNKKPRICTREEFTRKVRSNAAIGAVFVDENQWNSAKEAVEDERFWDLVHRERELHKQGKCATCVYNMMGKREKKLGEFGKAKGSRAIWYMWLGARFLEFEALGFMNEDHWFSRENSLSGVEGEGLHKLGYILRDISKIPGGNMYADDTAGWDTRITEDDLQNEAKITDIMEPEHALLATSIFKLTYQNKVVRVQRPAKNGTVMDVISRRDQRGSGQVGTYGLNTFTNMEVQLIRQMESEGIFLPSELETPNLAERVLDWLEKHGAERLKRMAISGDDCVVKPIDDRFATALTALNDMGKVRKDIPQWEPSKGWNDWQQVPFCSHHFHQLIMKDGREIVVPCRNQDELVGRARVSQGAGWSLRETACLGKSYAQMWQLMYFHRRDLRLAANAICSAVPVDWVPTSRTTWSIHAHHQWMTTEDMLSVWNRVWIEENPWMEDKTHVSSWEEVPYLGKREDQWCGSLIGLTARATWATNIQVAINQVRRLIGNENYLDYMTSMKRFKNESDPEGALW

>DENV1_AHC98445_Nicaragua

MNNQRKKTGRPSFNMLKRARNRVSTGSQLAKRFSKGLLSGQGPMKLVMAFIAFLRFLAIPPTAGILARWSSFKKNGAIKVLRGFKKEISSMLNIMNRRKRSVTMLLMLLPTALAFHLTTRGGEPHMIVSKHERGKSLLFKTSTGVNMCTLIAMDLGELCEDTMTYKCPRITEAEPDDVDCWCNATDTWVTYGTCSQTGEHRRDKRSVALAPHVGLGLETRTETWMSSEGAWKQIQRVETWALRHPGFTVIALFLAHAIGTSITQKGIIFILLMLVTPSMAMRCVGIGNRDFVEGLSGATWVDVVLEHGSCVTTMAKNKPTLDIELLKTEVTNPAVLRKLCIEAKISNTTTDSRCPTQGEATLVEEQDANFVCRRTLVDRGWGNGCGLFGKGSLLTCAKFKCVTKLEGKIVQYENLKYSVIVTVHTGDQHQVGNETTEHGTIATITPQAPTSEIQLTDYGALALDCSPRTGLDFNEMVLLTMKEKSWLVHKQWFLDLPLPWTSGASTSQETWNRQDLLVTFKTAHAKKQEVVVLGSQEGAMHTALTGATEIQTSGTTTIFAGHLKCRLKMDKLTLKGTSYVMCTGSFKLEKEVAETQHGTVLVQVKYEGTDAPCKIPFSTQDEKGVTQNGRLITANPIVTDKEKPVNIETEPPFGESYIVVGAGEKALKLSWFKKGSSIGKMFEATARGARRMAILGDTAWDFGSIGGVFTSVGKLVHQVFGTAYGVLFSGVSWTMKIGIGILLTWLGLNSRSTSLSMTCIAVGMVTLYLGVMVQADSGCVINWKGRELKCGSGIFVTNEVHTWTEQYKFQADSPKRLSAAIGKAWEEGVCGIRSATRLENIMWKQISNELNHILLENDMKFTVVVGDANGILAQGKKMIRPQPMEHKYSWKSWGKAKIIGADIQNTTFIIDGPDTPECPDDQRAWNIWEVEDYGFGIFTTNIWLKLRDSYTQMCDHRLMSAAIKDSKAVHADMGYWIESEKNETWKLARASFIEVKTCTWPKSHTLWSNGVLESEMIIPKIYGGPISQHNYRPGYFTQTAGPWHLGKLELDFDLCEGTTVVVDEHCGNRGPSLRTTTVTGKIIHEWCCRSCTLPPLRFRGEDGCWYGMEIRPVKEKEENLVRSMVSAGSGEVDSFSLGILCVSIMIEEVMRSRWSRKMLMTGTLAVFLLLIMGQLTWNDLIRLCIMVGANASDRMGMGTTYLALMATFKLRPMFAVGILFRRLTSREVLLLTIGLSLVASVELPNSLEELGDGLAMGIMMLKLLTEFQPHQLWTTLLSLTFIKTTLSLDYAWKTTAMALSIVSLFPLCLSTTSQKTTWLPVLLGSFGCKPLTMYLITENKIWGRKSWPINEGIMAIGIVSILLSSLLKNDVPLAGPLIAGGMLIACYVISGSSADLSLEKAAEVSWEQEAEHSGTSHNILVEVQDDGTMRIKDEEKDDTLTILLKATLLAVSGVYPMSIPATLFVWYFWQKKKQRSGVLWDTPSPPEVERAVLDDGIYRILQRGLLGRSQVGVGVFQDGVFHTMWHVTRGAVLMYQGKRLEPSWASVKKDLISYGGGWRFQGSWNTGEEVQVIAVEPGKNPKNVQTTPGTFKTPEGEVGAIALDFKPGTSGSPIVNREGKIVGLYGNGVVTTSGTYVSAIAQAKASQEGPLPEIEDEVFKKRNLTIMDLHPGSGKTRRYLPAIVREAIKRKLRTLILAPTRVVASEMAEALKGMPVRYQTTAVKSEHTGREIVDLMCHATFTMRLLSPVRVPNYNMIIMDEAHFTDPASIAARGYISTRVGMGEAAAIFMTATPPGSVEAFPQSNAVIQDEERDIPERSWNSGYDWITDFPGKTVWFVPSIKSGNDIANCLRKNGKRVIQLSRKTFDTEYQKTKNNDWDYVVTTDISEMGANFRADRVIDPRRCLKPVILKDGPERVILAGPMPVTVASAAQRRGRIGRNQNKEGDQYVYMGQPLNNDEDHAHWTEAKMLLDNINTPEGIIPALFEPEREKSAAIDGEYRLRGEARKTFVELMRRGDLPVWLSYKVASEGFQYSDRRWCFDGERNNQVLEENMDVEIWTKEGERKKLRPRWLDARTYSDPLALREFKEFAAGRRSVSGDLILEIGKLPQHLTLRAQNALDNLVMLHNSEQGGKAYRHAMEELPDTIETLMLLALIAVLTGGVTLFFLSGKGLGKTSIGLLCVTASSALLWMASVEPHWIAASIILEFFLMVLLIPEPDRQRTPQDNQLAYVVIGLLFMILTVAANEMGLLETTKRDLGIGHVAAENHQHATMLDVDLRPASAWTLYAVATTVITPMMRHTIENTTANISLTAIANQAAILMGLDKGWPISKMDIGVPLLALGCYSQVNPLTLTAAVLMLVAHYAIIGPGLQAKATREAQKRTAAGIMKNPTVDGIVAIDLDPVVYDAKFEKQLGQIMLLILCTSQILLMRTTWALCESITLATGPLTTLWEGSPGKFWNTTIAVSMANIFRGSYLAGAGLAFSLMKSLGGGRRGTGAQGETLGEKWKRQLNQLSKPEFNTYKRSGIMEVDRSEAKEGLKRGETTKHAVSRGTAKLRWFVERNLVKPEGKVIDLGCGRGGWSYYCAGLKKVTEVKGYTKGGPGHEEPIPMATYGWNLVKLHSGKDVFFMPPEKCDTLLCDIGESSPNPTIEEGRTLRVLKMVEPWLRGNQFCIKILNPYMPSVVETLEQMQRKHGGMLVRNPLSRNSTHEMYWVSCGTGNIVSAVNMTSRMLLNRFTMAHRKPTYERDVDLGAGTRHVTVEPEVANLDIIGQRIENIKNEHKSTWHYDEDNPYKTWAYHGSYEVKPSGSASSMVNGVVRLLTKPWDVIPMVTQIAMTDTTPFGQQRVFKEKVDTRTPRAKRGTTQIMEVTAKWLWGFLSRNKKPRICTREEFTRKVRSNAAIGAVFVDENQWNSAKEAVEDERFWDLVHRERELHKQGKCATCVYNMMGKREKKLGEFGKAKGSRAIWYMWLGARFLEFEALGFMNEDHWFSRENSLSGVEGEGLHKLGYILRDISKIPGGNMYADDTAGWDTRITEDDLQNEAKITDIMEPEHALLATSIFKLTYQNKVVRVQRPAKNGTVMDVISRRDQRGSGQVGTYGLNTFTNMEVQLIRQMESEGIFLPSELETPNLAERVLDWLEKHGAERLKRMAISGDDCVVKPIDDRFATALTALNDMGKVRKDIPQWEPSKGWNDWQQVPFCSHHFHQLIMKDGREIVVPCRNQDELVGRARVSQGAGWSLRETACLGKSYAQMWQLMYFHRRDLRLAANAICSAVPVDWVPTSRTTWSIHAHHQWMTTEDMLSVWNRVWIEENPWMEDKTHVSSWEEVPYLGKREDQWCGSLIGLTARATWATNIQVAINQVRRLIGNENYLDYMTSMKRFKNESDPEGALW

>DENV1_AHC98444_Nicaragua

MNNQRKKTGRPSFNMLKRARNRVSTGSQLAKRFSKGLLSGQGPMKLVMAFIAFLRFLAIPPTAGILARWSSFKKNGAIKVLRGFKKEISSMLNIMNRRKRSVTMLLMLMPTALAFHLTTRGGEPHMIVSKHERGKALLFKTSTGVNMCTLIAMDLGELCEDTMTYKCPRITEAEPDDVDCWCNATDTWVTYGTCSQTGEHRRDKRSVALAPHVGLGLETRTETWMSSEGAWKQIQRVETWALRHPGFTVIALFLAHAIGTSITQKGIIFILLMLVTPSMAMRCVGIGNRDFVEGLSGATWVDVVLEHGSCVTTMAKNKPTLDIELLKTEVTNPAVLRKLCIEAKISNTTTDSRCPTQGEATLVEEQDANFVCRRTFVDRGWGNGCGLFGKGSLLTCAKFKCVTKLEGKIVQYENLKYSVIVTVHTGDQHQVGNETTEHGTIATITPQAPTSEIQLTDYGALTLDCSPRTGLDFNEMVLLTMKEKSWLVHKQWFLDLPLPWTSGASTSQETWNRQDLLVTFKTAHAKKQEVVVLGSQEGAMHTALTGATEIQTSGTTTIFAGHLKCRLKMDKLTLKGTSYVMCTGSFKLEKEVAETQHGTVLVQVKYEGTDAPCKIPFSTQDEKGVTQNGRLITANPIVTDKEKPVNIETEPPFGESYIVVGAGEKALKLSWFKKGSSIGKMFEATARGARRMAILGDTAWDFGSIGGVFTSVGKLVHQVFGTAYGVLFSGVSWTMKIGIGILLTWLGLNSRSTSLSMTCIAVGMVTLYLGVMVQADSGCVINWKGRELKCGSGIFVTNEVHTWTEQYKFQADSPKRLSAAIGKAWEEGVCGIRSATRLENIMWKQISNELNHILLENDMKFTVVVGDANGILAQGKKMIRPQPMEHKYSWKSWGKAKIIGADIQNTTFIIDGPDTPECPDDQRAWNIWEVEDYGFGIFTTNIWLKLRDSYTQMCDHRLMSAAIKDSKAVHADMGYWIESEKNETWKLARASFIEVKTCTWPKSHTLWSNGVLESEMIIPKIYGGPISQHNYRPGYFTQTAGPWHLGKLELDFDLCEGTTVVVDEHCGNRGPSLRTTTVTGKIIHEWCCRSCTLPPLRFRGEDGCWYGMEIRPVKEKEENLVRSMVSAGSGEVDSFSLGILCVSIMIEEVMRSRWSRKMLMTGTLAVFLLLIMGQLTWNDLIRLCIMVGANASDRMGMGTTYLALMATFKMRPMFAVGILFRRLTSREVLLLTIGLSLVASVELPNSLEELGDGLAMGIMMLKLLTEFQPHQLWTTLLSLTFIKTTLSLDYAWKTTAMALSIVSLFPLCLSTTSQKTTWLPVLLGSFGCKPLTMFLITENKIWGRKSWPINEGIMAIGIVSILLSSLLKNDVPLAGPLIAGGMLIACYVISGSSADLSLEKAAEVSWEQEAEHSGTSHNILVEVQDDGTMRIKDEEKDDTLTILLKATLLAVSGVYPMSIPATLFVWYFWQKKKQRSGVLWDTPSPPEVERAVLDDGIYRILQRGLLGRSQVGVGVFQDGVFHTMWHVTRGAVLMYQGKRLEPSWASVKKDLISYGGGWRFQGSWNTGEEVQVIAVEPGKNPKNVQTTPGTFKTSEGEVGAIALDFKPGTSGSPIVNREGKIVGLYGNGVVTTSGTYVSAIAQAKTSQEGPLPEIEDEVFKKRNLTIMDLHPGSGKTRRYLPAIVREAIKRKLRTLILAPTRVVASEMAEALKGMPIRYQTTAVKSEHTGREIVDLMCHATFTMRLLSPVRVPNYNMIIMDEAHFTDPASIAARGYISTRVGMGEAAAIFMTATPPGSVEAFPQSNAVIQDEERDIPERSWNSGYDWITDFPGKTVWFVPSIKSGNDIANCLRKNGKRVIQLSRKTFDTEYQKTKNNDWDYVVTTDISEMGANFRADRVIDPRRCLKPVILKDGPERVILAGPMPVTVASAAQRRGRIGRNQNKEGDQYVYMGQPLNNDEDHAHWTEAKMLLDNINTPEGIIPALFEPEREKSAAIDGEYRLRGEARKTFVELMRRGDLPVWLSYKVASEGFQYSDRRWCFDGERNNQVLEENMDVEIWTKEGERKKLRPRWLDARTYSDPLALREFKEFAAGRRSVSGDLILEIGKLPQHLTLRAQNALDNLVMLHNSEQGGKAYRHAMEELPDTIETLMLLALIAVLTGGVTLFFLSGKGLGKTSIGLLCVTASSALLWMASVEPHWIAASIILEFFLMVLLIPEPDRQRTPQDNQLAYVVIGLLFMILTVAANEMGLLETTKRDLGIGHVAAENHQHATMLDVDLRPASAWTLYAVATTVITPMMRHTIENTTANISLTAIANQAAILMGLDKGWPISKMDIGVPLLALGCYSQVNPLTLTAAVLMLVAHYAIIGPGLQAKATREAQKRTAAGIMKNPTVDGIVAIDLDPVVYDAKFEKQLGQIMLLILCTSQILLMRTTWALCESITLATGPLTTLWEGSPGKFWNTTIAVSMANIFRGSYLAGAGLAFSLMKSLGGGRRGTGAQGETLGEKWKRQLNQLSKPEFNTYKRSGIMEVDRSEAKEGLKRGETTKHAVSRGTAKLRWFVERNLVKPEGKVIDLGCGRGGWSYYCAGLKKVTEVKGYTKGGPGHEEPIPMATYGWNLVKLHSGKDVFFMPPEKCDTLLCDIGESSPNPTIEEGRTLRVLKMVEPWLRGNQFCIKILNPYMPSVVETLEQMQRKHGGMLVRNPLSRNSTHEMYWVSCGTGNIVSAVNMTSRMLLNRFTMAHRKPTYERDVDLGAGTRHVAVEPEVANLDIIGQRIENIKNEHKSTWHYDEDNPYKTWAYHGSYEVKPSGSASSMVNGVVRLLTKPWDVIPMVTQIAMTDTTPFGQQRVFKEKVDTRTPRAKRGTTQIMEVTAKWLWGFLSRNKKPRICTREEFTRKVRSNAAIGAVFVDENQWNSAKEAVEDERFWDLVHRERELHKQGKCATCVYNMMGKREKKLGEFGKAKGSRAIWYMWLGARFLEFEALGFMNEDHWFSRENSLSGVEGEGLHKLGYILRDISKIPGGNMYADDTAGWDTRITEDDLQNEAKITDIMEPEHALLATSIFKLTYQNKVVRVQRPAKNGTVMDVISRRDQRGSGQVGTYGLNTFTNMEVQLIRQMESEGIFLPSELETPNLAERVLDWLEKHGAERLKRMAISGDDCVVKPIDDRFATALTALNDMGKVRKDIPQWEPSKGWNDWQQVPFCSHHFHQLIMKDGREIVVPCRNQDELVGRARVSQGAGWSLRETACLGKSYAQMWQLMYFHRRDLRLAANAICSAVPVDWVPTSRTTWSIHAHHQWMTTEDMLSVWNRVWIEENPWMEDKTHVSSWEEVPYLGKREDQWCGSLIGLTARATWATNIQVAINQVRRLIGNENYLDYMTSMKRFKNESDPEGALW

>DENV1_AHC98443_Nicaragua

MNNQRKKTGRPSFNMLKRARNRVSTGSQLAKRFSKGLLSGQGPMKLVMAFIAFLRFLAIPPTAGILARWSSFKKNGAIKVLRGFKKEISSMLNIMNRRKRSVTMLLMLLPTALAFHLTTRGGEPHMIVSKHERGKSLLFKTSTGVNMCTLIAMDLGELCEDTMTYKCPRITEAEPDDVDCWCNATDTWVTYGTCSQTGEHRRDKRSVALAPHVGLGLETRTETWMSSEGAWKQIQRVETWALRHPGFTVIALFLAHAIGTSITQKGIIFILLMLVTPSMAMRCVGIGNRDFVEGLSGATWVDVVLEHGSCVTTMAKNKPTLDIELLKTEVTNPAVLRKLCIEAKISNTTTDSRCPTQGEATLVEEQDANFVCRRTLVDRGWGNGCGLFGKGSLLTCAKFKCVTKLEGKIVQYENLKYSVIVTVHTGDQHQVGNETTEHGTIATITPQAPTSEIQLTDYGALALDCSPRTGLDFNEMVLLTMKEKSWLVHKQWFLDLPLPWTSGASTSQETWNRQDLLVTFKTAHAKKQEVVVLGSQEGAMHTALTGATEIQTSGTTTIFAGHLKCRLKMDKLTLKGTSYVMCTGSFKLEKEVAETQHGTVLVQVKYEGTDAPCKIPFSTQDEKGVTQNGRLITANPIVTDKEKPVNIETEPPFGESYIVVGAGEKALKLSWFKKGSSIGKMFEATARGARRMAILGDTAWDFGSIGGVFTSVGKLVHQVFGTAYGVLFSGVSWTMKIGIGILLTWLGLNSRSTSLSVTCIAVGMVTLYLGVMVQADSGCVINWKGRELKCGSGIFVTNEVHTWTEQYKFQADSPKRLSAAIGKAWEEGVCGIRSATRLENIMWKQISNELNHILLENDMKFTVVVGDANGILAQGKKMIRPQPMEHKYSWKSWGKAKIIGADIQNTTFIIDGPDTPECPDDQRAWNIWEVEDYGFGIFTTNIWLKLRDSYTQMCDHRLMSAAIKDSKAVHADMGYWIESEKNETWKLARASFIEVKTCTWPKSHTLWSNGVLESEMIIPKIYGGPISQHNYRPGYFTQTAGPWHLGKLELDFDLCEGTTVVVDEHCGNRGPSLRTTTVTGKIIHEWCCRSCTLPPLRFRGEDGCWYGMEIRPVKEKEENLVRSMVSAGSGEVDSFSLGILCVSIMIEEVMRSRWSRKMLMTGTLAVFLLLIMGQLTWNDLIRLCIMVGANASDRMGMGTTYLALMATFKLRPMFAVGILFRRLTSREVLLLTIGLSLVASVELPNSLEELGDGLAMGIMMLKLLTEFQPHQLWTTLLSLTFIRTTLSLDYAWKTTAMALSIVSLFPLCLSTTSQKTTWLPVLLGSFGCKPLTMYFITENKIWGRKSWPINEGIMAIGIVSILLSSLLKNDVPLAGPLIAGGMLIACYVISGSSADLSLEKAAEVSWEQEAEHSGTSHNILVEVRDDGTMRIKDEEKDDTLTILLKATLLAVSGVYPMSIPATLFVWYFWQKKKQRSGVLWDTPSPPEVERAVLDDGIYRILQRGLLGRSQVGVGVFQDGVFHTMWHVTRGAVLMYQGKRLEPSWASVKKDLISYGGGWRFQGSWNTGEEVQVIAVEPGKNPKNVQTTPGTFKTPEGEVGAIALDFKPGTSGSPIVNREGKIVGLYGNGVVTTSGTYVSAIAQAKASQEGPLPEIEDEVFKKRNLTIMDLHPGSGKTRRYLPAIVREAIKRKLRTLILAPTRVVASEMAEALKGMPIRYQTTAVKSEHTGREIVDLMCHATFTMRLLSPVRVPNYNMIIMDEAHFTDPASIAARGYISTRVGMGEAAAIFMTATPPGSVEAFPQSNAVIQDEERDIPERSWNSGYDWITDFPGKTVWFVPSIKSGNDIANCLRKNGKRVIQLSRKTFDTEYQKTKNNDWDYVVTTDISEMGANFRADRVIDPRRCLKPVILKDGPERVILAGPMPVTVASAAQRRGRIGRNQNKEGDQYVYMGQPLNNDEDHAHWTEAKMLLDNINTPEGIIPALFEPEREKSAAIDGEYRLRGEARKTFVELMRRGDLPVWLSYKVASEGFQYSDRRWCFDGERNNQVLEENMDVEIWTKEGERKKLRPRWLDARTYSDPLALREFKEFAAGRRSVSGDLILEIGKLPQHLTLRAQNALDNLVMLHNSEQGGKAYRHAMEELPDTIETLMLLALIAVLTGGVTLFFLSGKGLGKTSIGLLCVTASSALLWMASVEPHWIAASIILEFFLMVLLIPEPDRQRTPQDNQLAYVVIGLLFMILTVAANEMGLLETTKRDLGIGHVAAENHQHATMLDVDLRPASAWTLYAVATTVITPMMRHTIENTTANISLTAIANQAAILMGLDKGWPISKMDIGVPLLALGCYSQVNPLTLTAAVLMLVAHYAIIGPGLQAKATREAQKRTAAGIMKNPTVDGIVAIDLDPVVYDAKFEKQLGQIMLLILCTSQILLMRTTWALCESITLATGPLTTLWEGSPGKFWNTTIAVSMANIFRGSYLAGAGLAFSLMKSLGGGRRGTGAQGETLGEKWKRQLNQLSKPEFNTYKRSGIMEVDRSEAKEGLKRGETTKHAVSRGTAKLRWFVERNLVKPEGKVIDLGCGRGGWSYYCAGLKKVTEVKGYTKGGPGHEEPIPMATYGWNLVKLHSGKDVFFMPPEKCDTLLCDIGESSPNPTIEEGRTLRVLKMVEPWLRGNQFCIKILNPYMPSVVETLEQMQRKHGGMLVRNPLSRNSTHEMYWVSCGTGNIVSAVNMTSRMLLNRFTMAHRKPTYERDVDLGAGTRHVAVEPEVANLDIIGQRIENIKNEHKSTWHYDEDNPYKTWAYHGSYEVKPSGSASSMVNGVVRLLTKPWDVIPMVTQIAMTDTTPFGQQRVFKEKVDTRTPRAKRGTTQIMEVTAKWLWGFLSRNKKPRICTREEFTRKVRSNAAIGAVFVDENQWNSAKEAVEDERFWDLVHRERELHKQGKCATCVYNMMGKREKKLGEFGKAKGSRAIWYMWLGARFLEFEALGFMNEDHWFSRENSLSGVEGEGLHKLGYILRDISKIPGGNMYADDTAGWDTRITEDDLQNEAKITDIMEPEHALLATSIFKLTYQNKVVRVQRPAKNGTVMDVISRRDQRGSGQVGTYGLNTFTNMEVQLIRQMESEGIFLPSELETPNLAERVLDWLEKHGAERLKRMAISGDDCVVKPIDDRFATALTALNDMGKVRKDIPQWEPSKGWNDWQQVPFCSHHFHQLIMKDGREIVVPCRNQDELVGRARVSQGAGWSLRETACLGKSYAQMWQLMYFHRRDLRLAANAICSAVPVDWVPTSRTTWSIHAHHQWMTTEDMLSVWNRVWIEENPWMEDKTHVSSWEEVPYLGKREDQWCGSLIGLTARATWATNIQVAINQVRRLIGNESYLDYMTSMKRFKNESDPEGALW

>DENV1_AET43248_El_Salvador

MNNQRKKTGRPSFNMLKRARNRVSTGSQLAKRFSKGLLSGQGPMKLVMAFIAFLRFLAIPPTAGILARWSSFKKNGAIKVLRGFKKEISSMLYIMNRRKRSVTMLLMLLPTALAFHLTTRGGEPHMVVSKQERGKSLLFKTSAGVNMCTLIAMDLGELCEDTMTYKCPRITEAEPDDVDCWCNATDTWVTYGTCSQTGEHRRDKRSVALAPHVGLGLETRTETWMSSEGAWKQIQKVETWALRHPGFTVIALFLAHAIGTSITQKGIIFILLMLVTPSMAMRCVGIGNRDFVEGLSGATWVDVVLEHGSCVTTMAKNKPTLDIELLKTEVTNPAILRKLCIEAKISNTTTDSRCPTQGEATLVEEQDANFVCRRTFVDRGWGNGCGLFGKGSLLTCAKFKCVTKLEGKIVQHENLKYSVIVTVHTGDQHQVGNETTEHGTIATITPQAPTSEIQLTDYGALTLDCSPRTGLDFNEMVLLTMKEKSWLVHKQWFLDLPLPWTSGASTSHETWNRQDLLVTFKTAHAKKQEVVVLGSQEGAMHTALTGATEIQTSGTTTIFAGHLKCRLKMDKLTLKGMSYVMCTGSFKLEKEVAETQHGTVLVQVKYEGTDAPCKIPFSTQDEKGVTQNGRLITANPIVTDKEKPVNIETEPPFGESYIVVGAGEKALKLSWFKKGSSIGKMFEATARGARRMAILGDTAWDFGSIGGVFTSVGKLVHQVFGTAYGVLFSGVSWTMKIGIGILLTWLGLNSRSTSLSMTCIAVGMVTLYLGVMVQADSGCVINWKGRELKCGSGIFVTNEVHTWTEQYKFQADSPKRLSAAIGKAWEEGVCGIRSATRLENIMWKQISNELNHILLENDMKFTVVVGDASGILAQGKKMIRPQPMEHKYSWKSWGKAKIIGADTQNTTFIIDGPDTPECPDDQRAWNIWEVEDYGFGIFTTNIWLKLRDSYTQMCDHRLMSAAIKDSKAVHADMGYWIESEKNETWKLARASFIEVKTCIWPKSHTLWSNGVLESEMIIPKIYGGPISQHNYRPGYFTQTAGPWHLGKLELDFDLCEGTTVVVDEHCGNRGPSLRTTTVTGKIIHEWCCRSCTLPPLRFRGEDGCWYGMEIRPVKEKEENLVRSMVSAGSGEVDSFSLGILCVSIMIEEVMRSRWSRKMLMTGTLAVFLLLIMGQLTWNDLIRLCIMVGANASDRMGMGTTYLALMGTFKMRPMFAVGLLFRRLTSREVLLLTIGLSLVASVELPNSLEELGDGLAMGIMMLKLLTEFQPHQLWTTLLSLTFIKTTLSLDYAWKTMAMVLSIVSLFPLCLSTTSQKTTWLPVLLGSFGCKPLTMFLITENKIWGRKSWPLNEGIMAIGIVSILLSSLLKNDVPLAGPLIAGGMLIACYVISGSSADLSLEKAAEISWEEEAEHSGTSHNILVEVQDDGTMKIKDEERDDTLTILLKATLLAVSGVYPMSIPATLFVWYFWQKKKQRSGVLWDTPSPPEVERAVLDDGIYRILQRGLLGRSQVGVGVFQDGVFHTMWHVTRGAVLMYQGKRLEPSWASVKKDLISYGGGWRFQGSWNTGEEVQVIAVEPGKNPKNVQTTPGTFKTPEGEVGAIALDFKPGTSGSPIVNREGKIVGLYGNGVVTTSGTYVSAIAQAKASQEGPLPEIEDEVFKKRNLTIMDLHPGSGKTRRYLPAIVREAIKRKLRTLILAPTRVVASEMAEALKGMPIRYQTTAVKSEHTGREIVDLMCHATFTMRLLSPVRVPNYNMIIMDEAHFTDPASIAARGYISTRVGMGEAAAIFMTATPPGSVEAFPQSNAVIQDEERDIPERSWNSGYDWITDFPGKTIWFVPSIKSGNDIANCLRKNGKRVIQLSRKTFDTEYQKTKNNDWDYVVTTDISEMGANFRADRVIDPRRCLKPVILKDGPERVILAGPMPVTVASAAQRRGRIGRNQNKEGDQYVYMGQPLNNDEDHAHWTEAKMLLDNINTPEGIIPALFEPEREKSAAIDGEYRLRGEARKTFVELMRRGDLPVWLSYKVASEGFQYSDRRWCFDGERNNQVLEENMDVEIWTKEGERKKLRPRWLDARTYSDPLALREFKEFAAGRRSISGDLILEIGKLPQHLTLRAQNALDNLVMLHNSEQGGKAYRHAMEELPDTIETLMLLALIAVLTGGVTLFFLSGKGLGKTSIGLLCVMASSALLWMANVEPHWIAASIILEFFLMVLLIPEPDRQRTPQDNQLAYVVIGLLFMILTVAANEMGLLETTKKDLGIGHVAAENHHHATMLDVDLRPASAWTLYAVATTVITPMMRHTIENTTANISLTAIANQAAILMGLDKGWPISKMDIGVPLLALGCYSQVNPLTLTAAVLMLVAHYAIIGPGLQAKATREAQKRTAAGIMKNPTVDGIVAIDLDPVVYDAKFEKQLGQIMLLILCTSQILLMRTTWALCESITLATGPLTTLWEGSPGKFWNTTIAVSMANIFRGSYLAGAGLAFSLMKSLGGGRRGTGAQGETLGEKWKRQLNQLSKSEFNTYKRSGIMEVDRSEAKEGLKRGETTKHAVSRGTAKLRWFVERNLVKPEGKVIDLGCGRGGWSYYCAGLKKVTEVKGYTKGGPGHEEPIPMATYGWNLVKLHSGKDVFFTPPEKCDTLLCDIGESSPNPTIEEGRTLRVLKMVEPWLRGNQFCIKILNPYMPSVVETLEQMQRKHGGMLVRNPLSRNSTHEMYWVSCGTGNIVSAVNMTSRMLLNRFTMAHRKPTYEKDVDLGAGTRHVAVEPEVANLDIIGQRIENIKNEHKSTWHYDEDNPYKTWAYHGSYEVKPSGSASSMVNGVVRLLTKPWDVIPMVTQIAMTDTTPFGQQRVFKEKVDTRTPRAKRGTAQIMEVTAKWLWGFLSRNKKPRICTREEFTKKVRSNAAIGAVFVDENQWNSAKEAVEDERFWDLVHRERELHKQGKCATCVYNMMGKREKKLGEFGKAKGSRAIWYMWLGARFLEFEALGFMNEDHWFSRENSLSGVEGEGLHKLGYILRDISKIPGGNMYADDTAGWDTRITEDDLQNEAKITDIMEPEHALLATSIFKLTYQNKVVRVQRPAKNGNVMDVISRRDQRGSGQVGTYGLNTFTNMEVQLIRQMESEGIFLPSELETPNLAERVLDWLEKHGAERLKRMAISGDDCVVKPIDDRFATALIALNDMGKVRKDIPQWEPSKGWNDWQQVPFCSHHFHQLIMKDGREIVVPCRNQDELVGRARVSQGAGWSLRETACLGKSYAQMWQLMYFHRRDLRLAANAICSAVPVDWVPTSRTTWSIHAHHQWMTTEDMLSVWNRVWIEENPWMEDKTHVSSWEEVPYLGKREDQWCGSLIGLTARATWATNIQVAINQVRRLIGNENYLDYMTSMKRFKNESDPEGALW

>DENV1_ALJ53459_Haiti

MNNQRKKTGRPSFNMLKRARNRVSTGSQLAKRFSKGLLSGQGPMKLVMAFIAFLRFLAIPPTAGILARWSSFKKNGAIKVLRGFKKEISSMLNIMNRRKRSVTMLLMLLPTALAFHLTTRGGEPHMIVSKQERGKSLLFKTSAGVNMCTLIAMDLGELCEDTMTYKCPRITEAEPDDVDCWCNATDTWVTYGTCSQTGEHRREKRSVALAPHVGLGLETRTETWMSSEGAWKQIQRVETWALRHPGFTVIAFFLAHAIGTSITQKGIIFILLMLVTPSMAMRCVGIGNRDFVEGLSGATWVDVVLEHGSCVTTMAKNKPTLDIELLKTEVTNPAVLRKLCIEAKISNTTTDSRCPTQGEATLVEEQDANFVCRRTFVDRGWGNGCGLFGKGSLLTCAKFKCVTKLEGKIVQYENLKYSVIVTVHTGDQHQVGNETTEHGTIATITPQAPTSEIQLTDYGALTLDCSPRTGLDFNEMVLLTMKEKSWLVHKQWFLDLPLPWTSGASTSQETWNRQDLLVTFKTAHAKKQEVVVLGSQEGAMHTALTGATEIQTSGTTTIFAGHLKCRLKMDKLTLKGMSYVMCTGSFKLEKEVAETQHGTVLVQVKYEGTDAPCKIPFSTQDEKGVTQNGRLITANPIVTDKEKPVNIETEPPFGESYIVVGAGEKALKLSWFKRGSSIGKMFEATARGARRMAILGDTAWDFGSIGGVFTSVGKLVHQIFGTAYGVLFSGVSWTMKIGIGILLTWLGLNSRSTSLSMTCIAVGMVTLYLGVMVQADSGCVINWKGRELKCGSGIFVTNEVHTWTEQYKFQADSPKRLSAAIGKAWEEGVCGIRSATRLENIMWKQISNELNHILLENDMKFTVVVGDANGILAQGKKMIRPQPMEHKYSWKSWGKAKIIGADIQNTTFIIDGPDTPECPDGQRAWNIWEVEDYGFGVFTTNIWLKLRDSYTQMCDHRLMSAAIKDSKAVHADMGYWIESEKNETWKLARASFIEVKTCTWPKSHTLWSNGVLESEMIIPKIYGGPISQHNYRPGYFTQTAGPWHLGKLELDFDLCEGTTVVVDEHCGNRGPSLRTTTVTGKIIHEWCCRSCTLPPLRFRGEDGCWYGMEIRPVKEKEENLVRSMVSAGSGEVDSFSLGILCVSIMIEEVMRSRWSRKMLMTGTLAVFLLLIMGQLTWNDLIRLCIMVGANASDRMGMGTTYLALMATFKMRPMFAVGLLFRRLTSREVLLLTIGLSLVASVELPNSLEELGDGLAMGIMMLKLLTEFQPHQLWTTLLSLTFVKTTLSLDYAWKTTAMALSIVSLFPLCLSTTSQKTTWLPVLLGSFGCKPLTMFLITENKIWGRKSWPLNEGIMAIGIVSILLSSLLKNDVPLAGPLIAGGMLIACYVISGSSADLSLEKAAEVSWEQEAEHSGASHSILVEVQDDGTMKIKDEERDDTLTILLKATLLAVSGVYPMSIPATLFVWYFWQKKKQRSGVLWDTPSPPEVERAVLDNGIYRILQRGLLGRSQVGVGVFQDGVFHTMWHVTRGAVLMYQGKRLEPSWASVKKDLISYGGGWRFQGSWNTGEEVQVIAVEPGKNPKNVQTTPGTFKTPEGEVGAIALDFKPGTSGSPIVNREGKIVGLYGNGVVTTSGTYVSAIAQAKASQEGPLPEIEDEVFKKRNLTIMDLHPGSGKTRRYLPAIVREAIKRKLRTLILAPTRVVASEMAEALKGMPIRYQTTAVKSEHTGREIVDLMCHATFTMRLLSPVRVPNYNMIIMDEAHFTDPASIAARGYISTRVGMGEAAAIFMTATPPGSVEAFPQSNAVIQDEERDIPERSWNSGYDWITDFPGKTVWFVPSIKSGNDIANCLRKNGKRVIQLSRKTFDTEYQKTKNNDWDYVVTTDISEMGANFRADRVIDPRRCLKPVILKDGPERVILAGPMPVTAASAAQRRGRIGRNQNKEGDQYVYMGQPLNNDEDHAHWTEAKMLLDNINTPEGIIPALFEPEREKSAAIDGEYRLRGEARKTFVELMRRGDLPVWLSYKVASEGFQYSDRRWCFDGERNNQVLEENMDVEIWTKEGERKKLRPRWLDARTYSDPLALREFKEFAAGRRSVSGDLILEIGKLPQHLTLRAQNALDNLVMLHNSEQGGKAYRHAMEELPDTIETLMLLALIAVLTGGVTLFFLSGKGLGKTSIGLLCVTASSALLWMASVEPHWIAASIILEFFLMVLLIPEPDRQRTPQDNQLAYVVIGLLFMILTVAANEMGLLETTKKDLGIGYVAAENHQHATMLDVDLHPASAWTLYAVATTVITPMMRHTIENTTANISLTAIANQAAILMGLDKGWPISKMDIGVPLLALGCYSQVNPLTLTAAVLMLVAHYAIIGPGLQAKATREAQKRTAAGIMKNPTVDGIVAIDLDPVVYDAKFEKQLGQIMLLILCTSQILLMRTTWALCESITLATGPLTTLWEGSPGKFWNTTIAVSMANIFRGSYLAGAGLAFSLMKSLGGGRRGTGAQGETLGEKWKRQLNQLSKSEFNTYKRSGIMEVDRSEAKEGLKRGETTKHAVSRGTAKLRWFVERNLVKPEGKVIDLGCGRGGWSYYCAGLKKVTEVKGYTKGGPGHEEPIPMATYGWNLVKLHSGKDVFFMPPEKCDTLLCDIGESSPNPTIEEGRTLRVLKMVEPWLRGNQFCIKILNPYMPSVVETLERMQRKHGGMLVRNPLSRNSTHEMYWVSCGTGNIVSAVNMTSRMLLNRFTMAHRKPTYERDVDLGAGTRHVAVEPEVANLDIIGQRIENIKNEHKSTWHYDEDNPYKTWAYHGSYEVKPSGSASSMVNGVVRLLTKPWDVIPMVTQIAMTDTTPFGQQRVFKEKVDTRTPRAKRGTTQIMEVTAKWLWGFLSRNKKPRICTREEFTRKVRSNAAIGAVFVDENQWNSAKEAVEDERFWDLVHRERELHKQGKCATCVYNMMGKREKKLGEFGKAKGSRAIWYMWLGARFLEFEALGFMNEDHWFSRENSLSGVEGEGLHKLGYILRDISKIPGGNMYADDTAGWDTRVTEDDLQNEAKITDIMEPEHALLATSIFKLTYQNKVVRVQRPAKNGTVMDVISRRDQRGSGQVGTYGLNTFTNMEVQLIRQMESEGIFLPSELETPNLAERALDWLEKHGAERLKRMAISGDDCVVKPIDDRFATALTALNDMGKVRKDIPQWEPSKGWNDWQQVPFCSHHFHQLIMKDGREIVVPCRNQDELVGRARVSQGAGWSLRETACLGKSYAQMWQLMYFHRRDLRLAANAICSAVPVDWVPTSRTTWSIHAHHQWMTTEDMLSVWNRVWIEENPWMENKTHVSSWEEVPYLGKREDQWCGSLIGLTARATWATNIQVAINQVRRLIGNENYLDYMTSMKRFKNESDSEGALW

>DENV1_AGN94879_Brazil

MNNQRKKTGRPSFNMLKRARNRVSTGSQLAKRFSKGLLSGQGPMKLVMAFIAFLRFLAIPPTAGILARWSSFKKNGAIKVLRGFKKEISSMLNIMNRRKRSVTMLLMLLPTALAFHLTTRGGEPHMIVSKQERGKSLLFKTSAGVNMCTLIAMDLGELCEDTMTYKCPRITEAEPDDVDCWCNATDTWVTYGTCSQTGEHRRDKRSVALAPHVGLGLETRTETWMSSEGAWKQIQKVETWALRHPGFTVIALFLAHAIGTSITQKGIIFILLMLVTPSMAMRCVGIGNRDFVEGLSGATWVDVVLEHGSCVTTMAKNKPTLDIELLKTEVTNPAVLRKLCIEAKISNTTTDSRCPTQGEATLVEEQDANFVCRRTFVDRGWGNGCGLFGKGSLLTCAKFKCVTKLEGKIVQYENLKYSVIVTVHTGDQHQVGNETTEHGTIATITPQAPTSEIQLTDYGALTLDCSPRTGLDFNEMVLLTMKEKSWLVHKQWFLDLPLPWTSGASTSQETWNRQDLLVTFKTAHAKKQEVVVLGSQEGAMHTALTGATEIQTSGTTTIFAGHLKCRLKMDKLTLKGTSYVMCTGSFKLEKEVAETQHGTVLVQVKYEGTDAPCKIPFSTQDEKGVTQNGRLITANPIVTDKEKPVNIETEPPFGESYIVVGAGEKALKLSWFKKGSSIGKMFEATARGARRMAILGDTAWDFGSIGGVFTSVGKLVHQVFGTAYGVLFSGVSWTMKIGIGILLTWLGLNSRSTSLSMTCIAVGMVTLYLGVMVQADSGCVINWKGRELKCGSGIFVTNEVHTWTEQYKFQADSPKRLSAAIGKAWEEGVCGIRSATRLENIMWKQISNELNHILLENDIKFTVVVGNANGILAQGKKMIRPQPMEHKYSWKSWGKAKIIGADIQNTTFIIDGPDTPECPDEQRAWNIWEVEDYGFGIFTTNIWLKLRDSYTQMCDHRLMSAAIKDSKAVHADMGYWIESEKNETWKLARASFIEVKTCIWPKSHTLWSNGVLESEMIIPKMYGGPISQHNYRPGYFTQTAGPWHLGKLELDFDLCEGTTVVVDEHCGSRGPSLRTTTVTGKIIHEWCCRSCTLPPLRFRGEDGCWYGMEIRPVKEKEENLVRSMVSAGSGEVDSFSLGILCVSIMIEEVMRSRWSRKMLMTGTLAVFLLLIMGQLTWNDLIRLCIMVGANASDKMGMGTTYLALMATFKMRPMFAVGLSFRRLTSREVLLLTIGLSLVASVELPNSLEELGDGLAMGIMMLKLLTEFQPHQLWTTLLSLTFIKTTLSLDYAWKTTAMVLSIVSLFPLCLSTTSQKTTWLPVLLGSFGCKPLTMFLITENEIWGRKSWPLNEGIMAIGIVSILLSSLLKNDVPLAGPLIAGGMLIACYVISGSSADLSLEKAAEVSWEEEAEHSGTSHNILVEVQDDGTMKIKDEERDDTLTILLKATLLAVSGVYPMSIPATLFVWYFWQKKKQRSGVLWDTPSPPEVERAVLDDGIYRILQRGLLGRSQVGVGVFQDGVFHTMWHVTRGAVLMYQGKRLEPSWASVKKDLISYGGGWRFQGSWNTGEEVQVIAVEPGKNPKNVQTTPGTFKTPEGEVGAIALDFKPGTSGSPIVNREGKIVGLYGNGVVTTSGTYVSAIAQAKASQEGPLPEIEDEVFRKRNLTIMDLHPGSGKTRRYLPAIVREAIKRKLRTLILAPTRVVASEMAEALKGMPIRYQTTAVKSEHTGREIVDLMCHATFTMRLLSPVRVPNYNMIIMDEAHFTDPASIAARGYISTRVGMGEAAAIFMTATPPGSVEAFPQSNAVIQDEERDIPERSWNSGYDWITDFPGKTVWFVPSIKSGNDIANCLRKNGKRVIQLSRKTFDTEYQKTKNNDWDYVVTTDISEMGANFRADRVIDPRRCLKPVILKDGPERVILAGPMPVTVASAAQRRGRIGRNQNKEGDQYVYMGQPLNNDEDHAHWTEAKMLLDNINTPEGIIPALFEPEREKSAAIDGEYRLRGEARKTFVELMRRGDLPVWLSYKVASEGFQYSDRRWCFDGERNNQVLEENMDVEIWTKEGERKKLRPRWLDARTYSDPLALREFKEFAAGRRSVSGDLILEIGKLPQHLTLRAQNALDNLVMLHNSEQGGKAYRHAMEELPDTIETLMLLALIAVLTGGVTLFFLSGKGLGKTSIGLLCVTASSALLWMASVEPHWIAASIILEFFLMVLLIPEPDRQRTPQDNQLAYVVIGLLFMILTVAANEMGLLETTKKDLGIGHVAAENHQHATMLDVDLHPASAWTLYAVATTVITPMMRHTIENTTANISLTAIANQAAILMGLDKGWPISKMDLGVPLLALGCYSQVNPLTLTAAVLMLVAHYAIIGPGLQAKATREAQKRTAAGIMKNPTVDGIVAIDLDPVVYDAKFEKQLGQIMLLILCTSQILLMRTTWALCESITLATGPLTTLWEGSPGKFWNTTIAVSMANIFRGSYLAGAGLAFSLMKSLGGGRRGTGAQGETLGEKWKRQLNQLSKSEFNTYKRSGIMEVDRSEAKEGLKRGETTKHAVSRGTAKLRWFVERNLVKPEGKVIDLGCGRGGWSYYCAGLKKVTEVKGYTKGGPGHEEPIPMATYGWNLVKLHSGKDVFFMPPEKCDTLLCDIGESSPNPTIEEGRTLRVLKMVEPWLRGNQFCIKILNPYMPSVVETLEQMQRKHGGMLVRNPLSRNSTHEMYWVSCGTGNIVSAVNMTSRMLLNRFTMAHRKPTYERDVDLGAGTRHVAVEPEVANLDIIGQRIENIKNEHKSTWHYDEDNPYKTWAYHGSYEVKPSGSASSMVNGVVRLLTKPWDVIPMVTQIAMTDTTPFGQQRVFKEKVDTRTPRAKRGTAQIMEVTAKWLWGFLSRNKKPRICTREEFTRKVRSNAAIGAVFVDENQWNSAKEAVEDERFWDLVHRERELHKQGKCATCVYNMMGKREKKLGEFGKAKGSRAIWYMWLGARFLEFEALGFMNEDHWFSRENSLSGVEGEGLHKLGYILRDISKIPGGNMYADDTAGWDTRITEDDLQNEAKITDIMEPEHALLATSIFKLTYQNKVVRVQRPAKNGTVMDVISRRDQRGSGQVGTYGLNTFTNMEVQLIRQMESEGIFFPSELESPNLAERVLDWLEKHGAERLKRMAISGDDCVVKPIDDRFATALIALNDMGKVRKDIPQWEPSKGWNDWQQVPFCSHHFHQLIMKDGREIVVPCRNQDELVGRARVSQGAGWSLRETACLGKSYAQMWQLMYFHRRDLRLAANAICSAVPVDWVPTSRTTWSIHAHHQWMTTEDMLSVWNRVWIEENPWMEDKTHVSSWEEVPYLGKREDQWCGSLIGLTARATWATNIQVAINQVRRLIGNENYLDYMTSMKRFKNESDPEGALW

>DENV1_AGN94878_Brazil

MNNQRKKTGRPSFNMLKRARNRVSTGSQLAKRFSKGLLSGQGPMKLVMAFIAFLRFLAIPPTAGILARWSSFKKNGAIKVLRGFKKEISSMLNIMNRRKRSVTMLLMLLPTALAFHLTTRGGEPHMIVSKQERGKSLLFKTSAGVNMCTLIAMDLGELCEDTMTYKCPRITEAEPDDVDCWCNATDTWVTYGTCSQTGEHRRDKRSVALAPHVGLGLETRTETWMSSEGAWKQIQKVETWALRHPGFTVIALFLAHAIGTSITQKGIIFILLMLVTPSMAMRCVGIGNRDFVEGLSGATWVDVVLEHGSCVTTMAKNKPTLDIELLKTEVTNPAVLRKLCIEAKISNTTTDSRCPTQGEATLVEEQDANFVCRRTFVDRGWGNGCGLFGKGSLLTCAKFKCVTKLEGKIVQYENLKYSVIVTVHTGDQHQVGNETTEHGTIATITPQAPTSEIQLTDYGALTLDCSPRTGLDFNEMVLLTMKEKSWLVHKQWFLDLPLPWTSGASTSQETWNRQDLLVTFKTAHAKKQEVVVLGSQEGAMHTALTGATEIQTSGTTTIFAGHLKCRLKMDKLTLKGMSYVMCTGSFKLEKEVAETQHGTVLVQVKYEGTDAPCKIPFSTQDEKGVTQNGRLITANPIVTDKEKPVNIETEPPFGESYIVVGAGEKALKLSWFKKGSSIGKMFEATARGARRMAILGDTAWDFGSIGGVFTSVGKLVHQVFGTAYGVLFSGVSWTMKIGIGILLTWLGLNSRSTSLSMTCIAVGMVTLYLGVMVQADSGCVINWKGRELKCGSGIFVTNEVHTWTEQYKFQADSPKRLSAAIGRAWEEGVCGIRSATRLENIMWKQISNELNHILLENDMKFTVVVGNANGILAQGKKMIKPQPMEHKYSWKSWGKAKIIGADIQNATFIIDGPDTPECPDEQRAWNIWEVEDYGFGIFTTNIWLKLRDSYTQMCDHRLMSAAIKDSKAVHADMGYWIESEKNETWKLARASFIEVKTCIWPKSHTLWSNGVLESEMIIPKMYGGPTSQHNYRPGYFTQTAGPWHLGKLELDFDLCEGTTVVVDEHCGSRGPSLRTTTVTGKIIHEWCCRSCTLPPLRFRGEDGCWYGMEIRPVKEKEENLVRSMVSAGSGEVDSFSLGILCVSIMIEEVMRSRWSRKMLMTGTLAVFLLLIMGQLTWNDLIRLCIMVGANASDKMGMGTTYLALMATFKMRPMFAVGLLFRRLTSREVLLLTIGLSLVASVELPNSLEELGDGLAMGIMMLKLLTEFQPHQLWTTLLSLTFIKTTLSLDYAWKTTATVLSIVSLFPLCLSTTSQKTTWLPVLLGSFGCKPLTMFLITENEIWGRKSWPLNEGIMAIGIVSILLSSLLKNDVPLAGPLIAGGMLIACYVISGSSADLSLEKAAEVSWEEEAEHSGTSHNILVEVQDDGTMKIKDEERDDTLTILLKATLLAISGVYPMSIPATLFVWYFWQKKKQRSGVLWDTPSPPEVERAVLDDGIYRILQRGLLGRSQVGVGVFQDGVFHTMWHVTRGAVLMYQGKRLEPSWASVKKDLISYGGGWRFQGSWNTGEEVQVIAVEPGKNPKNVQTTPGTFKTPEGEVGAIALDFKPGTSGSPIVNREGKIVGLYGNGVVTTSGTYVSAIAQAKASQEGPLPEIEDEVFKKRNLTIMDLHPGSGKTRRYLPAIVREAIKRKLRTLILAPTRVVASEMAEALKGMPIRYQTTAVKSEHTGREIVDLMCHATFTMRLLSPVRVPNYNMIIMDEAHFTDPASIAARGYISTRVGMGEAAAIFMTATPPGSVEAFPQSNAVIQDEERDIPERSWNSGYDWITDFPGKTVWFVPSIKSGNDIANCLRKNGKRVIQLSRKTFDTEYQKTKNNDWDYVVTTDISEMGANFRADRVIDPRRCLKPVILKDGPERVILAGPMPVTVASAAQRRGRIGRNQNKEGDQYVYMGQPLNNDEDHAHWTEAKMLLDNINTPEGIIPALFEPEREKSAAIDGEYRLRGEARKTFVELMRRGDLPVWLSYKVASEGFQYSDRRWCFDGERNNQVLEENMDVEIWTKEGERKKLRPRWLDARTYSDPLALREFKEFAAGRRSVSGDLILEIGKLPQHLTLRAQNALDNLVMLHNSEQGGKAYRHAMEELPDTIETLMLLALIAVLTGGVTLFFLSGKGLGKTSIGLLCVTASSALLWMASVEPHWIAASIILEFFLMVLLIPEPDRQRTPQDNQLAYVVIGLLFMILTVAANEMGLLETTKKDLGIGHVAAENHQHATMLDVDLHPASAWTLYAVATTVITPMMRHTIENTTANISLTAIANQAAILMGLDKGWPISKMDLGVPLLALGCYSQVNPLTLTAAVLMLVAHYAIIGPGLQAKATREAQKRTAAGIMKNPTVDGIVAIDLDPVVYDAKFEKQLGQIMLLILCTSQILLMRTTWALCESITLATGPLTTLWEGSPGKFWNTTIAVSMANIFRGSYLAGAGLAFSLMKSLGGGRRGTGAQGETLGEKWKRQLNQLSKSEFNTYKRSGIMEVDRSEAKEGLKRGETTKHAVSRGTAKLRWFVERNLVKPEGKVIDLGCGRGGWSYYCAGLKKVTEVKGYTKGGPGHEEPIPMATYGWNLVKLHSGKDVFFMPPEKCDTLLCDIGESSPNPTIEEGRTLRVLKMVEPWLRGNQFCIKILNPYMPSVVETLEQMQRKHGGMLVRNPLSRNSTHEMYWVSCGTGNIVSAVNMTSRMLLNRFTMAHRKPTYERDVDLGAGTRHVAVEPEVANLDIIGQRIENIKNEHKSTWHYDEDNPYKTWAYHGSYEVKPSGSASSMVNGVVRLLTKPWDVIPMVTQIAMTDTTPFGQQRVFKEKVDTRTPRAKRGTAQIMEVTAKWLWGFLSRNKKPRICTREEFTRKVRSNAAIGAVFVDENQWNSAKEAVEDERFWDLVHRERELHKQGKCATCVYNMMGKREKKLGEFGKAKGSRAIWYMWLGARFLEFEALGFMNEDHWFSRENSLSGVEGEGLHKLGYILRDISKIPGGNMYADDTAGWDTRITEDDLQNEAKITDIMEPEHALLATSIFKLTYQNKVVRVQRPAKNGTVMDVISRRDQRGSGQVGTYGLNTFTNMEVQLIRQMESEGIFFPSELESPNLAERVLDWLEKHGAERLKRMAISGDDCVVKPIDDRFATALIALNDMGKVRKDIPQWEPSKGWNDWQQVPFCSHHFHQLIMKDGREIVVPCRNQDELVGRARVSQGAGWSLRETACLGKSYAQMWQLMYFHRRDLRLAANAICSAVPVDWVPTSRTTWSIHAHHQWMTTEDMLSVWNRVWIEENPWMEDKTHVSSWEEVPYLGKREDQWCGSLIGLTARATWATNIQVAINQVRRLIGNENYLDYMTSMKRFKNESDPEGALW

>DENV1_AGN94877_Brazil

MNNQRKKTGRPSFNMLKRARNRVSTGSQLAKRFSKGLLSGQGPMKLVMAFIAFLRFLAIPPTAGILARWSSFKKNGAIKVLRGFKKEISSMLNIMNRRKRSVTMLLMLLPTALAFHLTTRGGEPHMIVSKQERGKSLLFKTSAGVNMCTLIAMDLGELCEDTMTYKCPRITEAEPDDVDCWCNATDTWVTYGTCSQTGEHRRDKRSVALAPHVGLGLETRTETWMSSEGAWKQIQKVETWALRHPGFTVIALFLAHAIGTSITQKGIIFILLMLVTPSMAMRCVGIGNRDFVEGLSGATWVDVVLEHGSCVTTMAKNKPTLDIELLKTEVTNPAVLRKLCIEAKISNTTTDSRCPTQGEATLVEEQDANFVCRRTFVDRGWGNGCGLFGKGSLLTCAKFKCVTKLEGKIVQYENLKYSVIVTVHTGDQHQVGNETTEHGTIATITPQAPSSEIQLTDYGALTLDCSPRTGLDFNEMVLLTMKEKSWLVHKQWFLDLPLPWTSGASTSQETWNRQDLLVTFKTAHAKKQEVVVLGSQEGAMHTALTGATEIQTSGTTTIFAGHLKCRLKMDKLTLKGTSYVMCTGSFKLEKEVAETQHGTVLVQVKYEGTDAPCKIPFSTQDEKGVTQNGRLITANPIVTDKEKPVNIETEPPFGESYIVVGAGEKALKLSWFKKGSSIGKMFEATARGARRMAILGDTAWDFGSIGGVFTSVGKLVHQVFGTAYGVLFSGVSWTMKIGIGILLTWLGLNSRSTSLSMTCIAVGMVTLYLGVMVQADSGCVINWKGRELKCGSGIFVTNEVHTWTEQYKFQADSPKRLSAAIGRAWEEGVCGIRSATRLENIMWKQISNELNHILLENDMKFTVVVGNANGILAQGKKMIKPQPMEHKYSWKSWGKAKIIGADIQNATFIIDGPDTPECPDEQRAWNIWEVEDYGFGIFTTNIWLKLRDSYTQMCDHRLMSAAIKDSKAVHADMGYWIESEKNETWKLARASFIEVKTCIWPKSHTLWSNGVLESEMIIPKMYGGPISQHNYRPGYFTQTAGPWHLGKLELDFDLCEGTTVVVDEHCGSRGPSLRTTTVTGKIIHEWCCRSCTLPPLRFRGEDGCWYGMEIRPVKEKEENLVRSMVSAGSGEVDSFSLGILCVSIMIEEVMRSRWSRKMLMTGTLAVFLLLIMGQLTWNDLIRLCIMVGANASDKMGMGTTYLALMATFKMRPMFAVGLLFRRLTSREVLLLTIGLSLVASVELPNSLEELGDGLAMGIMMLKLLTEFQPHQLWTTLLSLTFIKTTLSLDYAWKTTATVLSIVSLFPLCLSTTSQKTTWLPVLLGSFGCKPLTMFLITENKIWGRKSWPLNEGIMAIGIVSILLSSLLKNDVPLAGPLIAGGMLIACYVISGSSADLSLEKAAEVSWEEEAEHSGTSHNILVEVQDDGTMKIKDEERDDTLTILLKATLLAVSGVYPMSIPATLFVWYFWQKKKQRSGVLWDTPSPPEVERAVLDDGIYRILQRGLLGRSQVGVGVFQDGVFHTMWHVTRGAVLMYQGKRLEPSWASVKKDLISYGGGWRFQGSWNTGEEVQVIAVEPGKNPKNVQTTPGTFKTPEGEVGAIALDFKPGTSGSPIVNREGKIVGLYGNGVVTTSGTYVSAIAQAKASQEGPLPEIEDEVFKKRNLTIMDLHPGSGKTRRYLPAIVREAIKRKLRTLILAPTRVVASEMAEALKGMPIRYQTTAVKSEHTGREIVDLMCHATFTMRLLSPVRVPNYNMIIMDEAHFTDPASIAARGYISTRVGMGEAAAIFMTATPPGSVEAFPQSNAVIQDEERDIPERSWNSGYDWITDFPGKTVWFVPSIKSGNDIANCLRKNGKRVIQLSRKTFDTEYQKTKNNDWDYVVTTDISEMGANFRADRVIDPRRCLKPVILKDGPERVILAGPMPVTVASAAQRRGRIGRNQNKEGDQYVYMGQPLNNDEDHAHWTEAKMLLDNINTPEGIIPALFEPEREKSAAIDGEYRLRGEARKTFVELMRRGDLPVWLSYKVASEGFQYSDRRWCFDGERNNQVLEENMDVEIWTKEGERKKLRPRWLDARTYSDPLALREFKEFAAGRRSVSGDLILEIGKLPQHLTLRAQNALDNLVMLHNSEQGGKAYRHAMEELPDTIETLMLLALIAVLTGGVTLFFLSGKGLGKTSIGLLCVTASSALLWMASVEPHWIAASIILEFFLMVLLIPEPDRQRTPQDNQLAYVVIGLLFMILTVAANEMGLLETTKKDLGIGHVAAENHQHATMLDVDLHPASAWTLYAVATTVITPMMRHTIENTTANISLTAIANQAAILMGLDKGWPISKMDLGVPLLALGCYSQVNPLTLTAAVLMLVAHYAIIGPGLQAKATREAQKRTAAGIMKNPTVDGIVAIDLDPVVYDAKFEKQLGQIMLLILCTSQILLMRTTWALCESITLATGPLTTLWEGSPGKFWNTTIAVSMANIFRGSYLAGAGLAFSLMKSLGGGRRGTGAQGETLGEKWKRQLNQLSKSEFNTYKRSGIMEVDRSEAKEGLKRGETTKHAVSRGTAKLRWFVERNLVKPEGKVIDLGCGRGGWSYYCAGLKKVTEVKGYTKGGPGHEEPIPMATYGWNLVKLHSGKDVFFMPPEKCDTLLCDIGESSPNPTIEEGRTLRVLKMVEPWLRGNQFCIKILNPYMPSVVETLEQMQRKHGGMLVRNPLSRNSTHEMYWVSCGTGNIVSAVNMTSRMLLNRFTMAHRKPTYERDVDLGAGTRHVAVEPEVANLDIIGQRIENIKNEHKSTWHYDEDNPYKTWAYHGSYEVKPSGSASSMVNGVVRLLTKPWDVIPMVTQIAMTDTTPFGQQRVFKEKVDTRTPRAKRGTAQIMEVTAKWLWGFLSRNKKPRICTREEFTRKVRSNAAIGAVFVDENQWNSAKEAVEDERFWDLVHRERELHKQGKCATCVYNMMGKREKKLGEFGKAKGSRAIWYMWLGARFLEFEALGFMNEDHWFSRENSLSGVEGEGLHKLGYILRDISKIPGGNMYADDTAGWDTRITEDDLQNEAKITDIMEPEHALLATSIFKLTYQNKVVRVQRPAKNGTVMDVISRRDQRGSGQVGTYGLNTFTNMEVQLIRQMESEGIFFPSELESPNLAERVLDWLEKHGAERLKRMAISGDDCVVKPIDDRFATALIALNDMGKVRKDIPQWEPSKGWNDWQQVPFCSHHFHQLIMKDGREIVVPCRNQDELVGRARVSQGAGWSLRETACLGKSYAQMWQLMYFHRRDLRLAANAICSAVPVDWVPTSRTTWSIHAHHQWMTTEDMLSVWNRVWIEENPWMEDKTHVSSWEEVPYLGKREDQWCGSLIGLTARATWATNIQVAINQVRRLIGNENYLDYMTSMKRFKNESDPEGALW

>DENV1_AET43246_Venezuela

MNNQRKKTGRPSFNMLKRARNRVSTGSQLAKRFSKGLLSGQGPMKLVMAFIAFLRFLAIPPTAGILARWSSFKKNGAIKVLRGFKKEISSMLNIMNRRKRSVTMLLMLLPTALAFHLTTRGGEPHMIVSKQERGKSLLFKTSAGVNMCTLIAMDLGELCEDTMTYKCPRITEAEPDDVDCWCNATDTWVTYGTCSQTGEHRRDKRSVALAPHVGLGLETRTETWMSSEGAWKQIQRVETWALRHPGFTVTALFLAHAIGTSITQKGIIFILLMLVTPSMAMRCVGIGNRDFVEGLSGATWVDVVLEHGSCVTTMAKNKPTLDIELLKTEVTNPAVLRKLCIEAKISNTTTDSRCPTQGEATLVEEQDANFVCRRTFVDRGWGNGCGLFGKGSLLTCAKFKCVTKLEGKIVQYENLKYSVIVTVHTGDQHQVGNETTEHGTIATITPQAPTSEIQLTDYGALTLDCSPRTGLDFNEMVLLTMKEKSWLVHKQWFLDLPLPWTSGASTSQETWNRQDLLVTFKTAHAKKQEVVVLGSQEGAMHTALTGATEIQTSGTTTIFAGHLKCRLKMDKLTLKGTSYVMCTGSFKLEKEVAETQHGTVLVQVKYEGTDAPCKIPFSTQDEKGVTQNGRLITANPIVTDKEKPVNIETEPPFGESYIVVGAGEKALKLSWFKKGSSIGKMFEATARGARRMAILGDTAWDFGSIGGVFTSVGKLVHQVFGTAYGVLFSGVSWTMKIGIGILLTWLGLNSRSTSLSMTCIAVGMVTLYLGVMVQADSGCVINWKGRELKCGSGIFVTNEVHTWTEQYKFQADSPKRLSAAIGKAWEEGVCGIRSATRLENIMWKQISNELNHILLENDMKFTVVVGDANGILAQGKKMIRPQPMEHKYSWKSWGKAKIIGADIQNTTFIIDGPDTPECPDDQRAWNIWEVEDYGFGIFTTNIWLKLRDSYTQMCDHRLMSAAIKDSKAVHADMGYWIESEKNETWKLARASFIEVKTCTWPKSHTLWSNGVLESEMIIPKIYGGPISQHNYRPGYFTQTAGPWHLGKLELDFNLCEGTTVVVDEHCGNRGPSLRTTTVTGKIIHEWCCRSCTLPPLRFRGEDGCWYGMEIRPVKEKEENLVRSMVSAGSGEVDSFSLGILCVSIMIEEVMRSRWSRKMLMTGTLAVFLLLIMGQLTWNDLIRLCIMVGANASDRMGMGTTYLALMATFKMRPMFAVGLLFRRLTSREVLLLTIGLSLVASVELPNSLEELGDGLAMGIMMLKLLTEFQPHQLWTTLLSLTFIKTTLSLDYAWKTTAMALSIVSLFPLCLSTTSQKTTWLPVLLGSFGCKPLTMFLITENKIWGRKSWPLNEGIMAIGIVSILLSSLLKNDVPLAGPLIAGGMLIACYVISGSSADLSLEKAAEVSWEQEAEHSGTSHNILVEVQDDGTMKIKDEEKDDTLTILLKATLLAVSGVYPMSIPATLFVWYFWQKKKQRSGVLWDTPSPPEVERAVLDDGIYRILQRGLLGRSQVGVGVFQDGVFHTMWHVTRGAVLMYQGKRLEPSWASVKKDLISYGGGWRFQGSWNTGEEVQVIAVEPGKNPKNVQTTPGTFKTPEGEVGAIALDFKPGTSGSPIVNREGKIVGLYGNGVVTTSGTYVSAIAQAKASQEGPLPEIEDEVFKKRNLTIMDLHPGSGKTRRYLPAIVREAIKRKLRTLILAPTRVVASEMAEALKGMPIRYQTTAVKSEHTGREIVDLMCHATFTMRLLSPVRVPNYNMIIMDEAHFTDPASIAARGYISTRVGMGEAAAIFMTATPPGSVEAFPQSNAVIQDEERDIPERSWNSGYDWITDFPGKTVWFVPSIKSGNDIANCLRKNGKRVIQLSRKTFDTEYQKTKNNDWDYVVTTDISEMGANFRADRVIDPRRCLKPVILKDGPERVILAGPMPVTVASAAQRRGRIGRNQNKEGDQYVYMGQPLNNDEDHAHWTEAKMLLDNINTPEGIIPALFEPEREKSAAIDGEYRLRGEARKTFVELMRRGDLPVWLSYKVASEGFQYSDRRWCFDGERNNQVLEENMDVEIWTKEGERKKLRPRWLDARTYSDPLALREFKEFAAGRRSVSGDLILEIGKLPQHLTLRAQNALDNLVMLHNSEQGGKAYRHAMEELPDTIETLMLLALIAVLTGGVTLFFLSGKGLGKTSIGLLCVTASSALLWMASVEPHWIAASIILEFFLMVLLIPEPDRQRTPQDNQLAYVVIGLLFMILTVAANEMGLLETTKRDLGIGHVAAENHQHATMLDVDLRPASAWTLYAVATTVITPMMRHTIENTTANISLTAIANQAAILMGLDKGWPISKMDIGVPLLALGCYSQVNPLTLTAAVLMLVAHYAIIGPGLQAKATREAQKRTAAGIMKNPTVDGIVAIDLDPVVYDAKFEKQLGQIMLLILCTSQILLMRTTWALCESITLATGPLTTLWEGSPGKFWNTTIAVSMANIFRGSYLAGAGLAFSLMKSLGGGRRGTGAQGETLGEKWKRQLNQLSKSEFNTYKRSGIMEVDRSEAKEGLKRGETTKHAVSRGTAKLRWFVERNLVKPEGKVIDLGCGRGGWSYYCAGLKKVTEVKGYTKGGPGHEEPIPMATYGWNLVKLHSGKDVFFMPPEKCDTLLCDIGESSPNPTIEEGRTLRVLKMVEPWLRGNQFCIKILNPYMPSVVETLEQMQRKHGGMLVRNPLSRNSTHEMYWVSCGTGNIVSAVNMTSRMLLNRFTMAHRKPTYERDVDLGAGTRHVAVEPEVANLDIIGQRIENIKNEHKSTWHYDEDNPYKTWAYHGSYEVKPSGSASSMVNGVVRLLTKPWDVIPMVTQIAMTDTTPFGQQRVFKEKVDTRTPRAKRGTTQIMEVTAKWLWGFLSRNKKPRICTREEFTRKVRSNAAIGAVFVDENQWNSAKEAVEDERFWDLVHRERELHKQGKCATCVYNMMGKREKKLGEFGKAKGSRAIWYMWLGARFLEFEALGFMNEDHWFSRENSLSGVEGEGLHKLGYILRDISKIPGGNMYADDTAGWDTRITEDDLQNEAKITDIMEPEHALLATSIFKLTYQNKVVRVQRPAKNGTVMDVISRRDQRGSGQVGTYGLNTFTNMEVQLIRQMESEGIFLPSELETPNLAERVLDWLEKHGAERLKRMAISGDDCVVKPIDDRFATALTALNDMGKVRKDIPQWEPSKGWNDWQQVPFCSHHFHQLIMKDGREIVVPCRNQDELVGRARVSQGAGWSLRETACLGKSYAQMWQLMYFHRRDLRLAANAICSAVPVDWVPTSRTTWSIHAHHQWMTTEDMLSVWNRVWIEENPWMEDKTHVSSWEEVPYLGKREDQWCGSLIGLTARATWATNIQVAINQVRRLIGNENYLDYMTSMKRFKNESDPEGALW

>DENV1_AHI43687_Colombia

MNNQRKKTGRPSFNMLKRARNRVSTGSQLAKRFSKGLLSGQGPMKLVMAFIAFLRFLAIPPTAGILARWSSFKKNGAIKVLRGFKKEISSMLNIMNRRKRSVTMLLMLLPTALAFHLTTRGGEPHMIVSKQERGKSLLFKTSAGVNMCTLIAMDLGELCEDTMTYKCPRITEAEPDDVDCWCNATDTWVTYGTCSQTGEHRRDKRSVALAPHVGLGLETRTETWMSSEGAWKQIQRVETWALRHPGFTVIALFLAHVIGTSITQKGIIFILLMLVTPSMAMRCVGIGNRDFVEGLSGATWVDVVLEHGSCVTTMAKNKPTLDIELLKTEVTNPAVLRKLCIEAKISNTTTDSRCPTQGEATLVEEQDANFVCRRTFVDRGWGNGCGLFGKGSLLTCAKFKCVTKLEGKIVQYENLKYSVIVTVHTGDQHQVGNETTEHGTIATITPQAPTSEIQLTDYGALTLDCSPRTGLDFNEMVLLTMKEKSWLVHKQWFLDLPLPWTSGASTSQETWNRQDLLVTFKTAHAKKQEVVVLGSQEGAMHTALTGATEIQTSGTTTIFAGHLKCRLKMDKLTLKGMSYVMCTGSFKLEKEVAETQHGTVLVQVKYEGTDAPCKIPFSTQDEKGVTQNGRLITANPIVTDKEKPVNIETEPPFGESYIVVGAGEKALKLSWFKKGSSIGKMFEATARGARRMAILGDTAWDFGSIGGVFTSVGKLVHQVFGTAYGVLFSGVSWTMKIGIGILLTWLGLNSRSTSLSMTCIAVGMVTLYLGVMVQADSGCVINWKGRELKCGSGIFVTNEVHTWTEQYKFQADSPKRLSAAIGKAWEEGVCGIRSATRLENIMWKQISNELNHILLENDMKFTVVVGDANGILAQGKKMIRPQPMEHKYSWKSWGKAKIIGADTQNTTFIIDGPDTPECPDGQRAWNIWEVEDYGFGIFTTNIWLKLRDSYTQMCDHRLMSAAIKDSKAVHADMGYWIESEKNETWKLARASFIEVKTCTWPKSHTLWSNGVLESEMIIPKIYGGPISQHNYRPGYFTQTAGPWHLGKLELDFDLCEGTTVVVDEHCGNRGPSLRTTTVTGKIIHEWCCRSCTLPPLRFRGEDGCWYGMEIRPVKEKEENLVRSMVSAGSGEVDSFSLGILCVSIMIEEVMRSRWSRKMLMTGTLAVFLLLIMGQLTWNDLTRLCIMVGANASDRMGMGTTYLALMATFKMRPMFAVGLLFRRLTSREVLLLTIGLSLVASVELPNSLEELGDGLAMGIMMLKLLTEFQPHQLWTTLLSLTFVKTTLSLDYAWKTTAMVLSIVSLFPLCLSTTSQKTTWLPVLLGSFGCKPLTMFLITENKIWGRKSWPLNEGIMAIGIVSILLSSLLKNDVPLAGPLIAGGMLIACYVISGSSADLSLEKAAEVSWEQEAEHSGASHNILVEVQDDGTMKIKDEERDDTLTILLKATLLAVSGVYPMSIPATLFVWYFWQKKKQRSGVLWDTPSPPEVERAVLDDGIYRILQRGLLGRSQVGVGVFQDGVFHTMWHVTRGAVLMYQGKRLEPSWASVKKDLISYGGGWRFQGSWNTGEEVQVIAVEPGKNPKNVQTTPGTFKTPEGEVGAIALDFKPGTSGSPIVNREGKIVGLYGNGVVTTSGTYVSAIAQAKASQEGPLPEIEDEVFKKRNLTIMDLHPGSGKTRRYLPAIVREAIKRKLRTLILAPTRVVASEMAEALKGMPIRYQTTAVKSEHTGREIVDLMCHATFTMRLLSPVRVPNYNMIIMDEAHFTDPASIAARGYISTRVGMGEAAAIFMTATPPGSVEAFPQSNAVIQDEERDIPERSWNSGYDWITDFPGKTVWFVPSIKSGNDIANCLRKNGKRVIQLSRKTFDTEYQKTKNNDWDYVVTTDISEMGANFRADRVIDPRRCLKPVILKDGPERVILAGPMPVTVASAAQRRGRIGRNQNKEGDQYVYMGQPLNNDEDHAHWTEAKMLLDNINTPEGIIPALFEPEREKSAAVDGEYRLRGEARKTFVELMRRGDLPVWLSYKVASEGFQYSDRRWCFDGERNNQVLEENMDVEIWTKEGERKKLRPRWLDARTYSDPLALREFKEFAAGRRSVSGDLILEIGKLPQHLTLRAQNALDNLVMLHNSEQGGKAYRHAMEELPDTIETLMLLALIAVLTGGVTLFFLSGKGLGKTSIGLLCVTASSALLWMASVEPHWIAASIILEFFLMVLLIPEPDRQRTPQDNQLAYVVIGLLFMILTVAANEMGLLETTKKDLGIGYVAAENHQHATMLDVDLRPASAWTLYAVATTVITPMMRHTIENTTANISLTAIANQAAILMGLDKGWPISKMDIGVPLLALGCYSQVNPLTLTAAVLMLVAHYAIIGPGLQAKATREAQKRTAAGIMKNPTVDGIVAIDLDPVVYDAKFEKQLGQIMLLILCTSQILLMRTTWALCESITLATGPLTTLWEGSPGKFWNTTIAVSMANIFRGSYLAGAGLAFSLMKSLGGGRRGTGAQGETLGEKWKRQLNQLSKSEFNTYKRSGIMEVDRSEAKEGLKRGETTKHAVSRGTAKLRWFVERNLVKPEGKVIDLGCGRGGWSYYCAGLKKVTEVKGYTKGGPGHEEPIPMATYGWNLVKLHSGKDVFFMPPEKCDTLLCDIGESSPNPTIEEGRTLRVLKMVEPWLRGNQFCIKILNPYMPSVVETLEQMQRKHGGMLVRNPLSRNSTHEMYWVSCGTGNIVSAVNMTSRMLLNRFTMAHRKPTYERDVDLGAGTRHVAVEPEVANLDIIGQRIENIKNEHKSTWHYDEDNPYKTWAYHGSYEVKPSGSASSMVNGVVRLLTKPWDVIPMVTQIAMTDTTPFGQQRVFKEKVDTRTPRAKRGTTQIMEVTAKWLWGFLSRNKKPRICTREEFTRKVRSNAAIGAVFVDENQWNSAKEAVEDERFWDLVHRERELHKQGKCATCVYNMMGKREKKLGEFGKAKGSRAIWYMWLGARFLEFEALGFMNEDHWFSRENSLSGVEGEGLHKLGYILRDISKIPGGNMYADDTAGWDTRITEDDLQNEAKITDIMEPEHALLATSIFKLTYQNKVVRVQRPAKNGTVMDVISRRDQRGSGQVGTYGLNTFTNMEVQLIRQMESEGIFLPSELETPNLAERVLDWLEKHGAERLKRMAISGDDCVVKPIDDRFATALTALNDMGKVRKDIPQWEPSKGWNDWQQVPFCSHHFHQLIMKDGREIVVPCRNQDELVGRARVSQGAGWSLRETACLGKSYAQMWQLMYFHRRDLRLAANAICSAVPVEWVPTSRTTWSIHAHHQWMTTEDMLSVWNRVWIEENPWMEDKTHVSSWEEVPYLGKREDQWCGSLIGLTARATWATNIQVAINQVRRLIGNENYLDYMTSMKRFKNESDPEGALW

>DENV1_AHI43686_Colombia

MNNQRKKTGRPSFNMLKRARNRVSTGSQLAKRFSKGLLSGQGPMKLVMAFIAFLRFLAIPPTAGILARWSSFKKNGAIKVLRGFKKEISSMLNIMNRRKRSVTMLLMLLPTALAFHLTTRGGEPHMIVSKQERGKSLLFKTSAGVNMCTLIAMDLGELCEDTMTYKCPRITEAEPDDVDCWCNATDTWVTYGTCSQTGEHRRDKRSVALAPHVGLGLETRTETWMSSEGAWKQIQRVETWALRHPGFTVIALFLAHVIGTSITQKGIIFILLMLVTPSMAMRCVGIGNRDFVEGLSGATWVDVVLEHGSCVTTMAKNKPTLDIELLKTEVTNPAVLRKLCIEAKISNTTTDSRCPTQGEATLVEEQDANFVCRRTFVDRGWGNGCGLFGKGSLLTCAKFKCVTKLEGKIVQYENLKYSVIVTVHTGDQHQVGNETTEHGTIATITPQAPTSEIQLTDYGALTLDCSPRTGLDFNEMVLLTMKEKSWLVHKQWFLDLPLPWTSGASTSQETWNRQDLLVTFKTAHAKKQEVVVLGSQEGAMHTALTGATEIQTSGTTTIFAGHLKCRLKMDKLTLKGMSYVMCTGSFKLEKEVAETQHGTVLVQVKYEGTDAPCKIPFSTQDEKGVTQNGRLITANPIVTDKEKPVNIETEPPFGESYIVVGAGEKALKLSWFKKGSSIGKMFEATARGARRMAILGDTAWDFGSIGGVFTSVGKLVHQVFGTAYGVLFSGVSWTMKIGIGILLTWLGLNSRSTSLSMTCIAVGMVTLYLGVMVQADSGCVINWKGRELKCGSGIFVTNEVHTWTEQYKFQADSPKRLSAAIGKAWEEGVCGIRSATRLENIMWKQISNELNHILLENDMKFTVVVGDANGILAQGKKMIRPQPMEHKYSWKSWGKAKIIGADTQNTTFIIDGPDTPECPDGQRAWNIWEVEDYGFGIFTTNIWLKLRDSYTQMCDHRLMSAAIKDSKAVHADMGYWIESEKNETWKLARASFIEVKTCTWPKSHTLWSNGVLESEMIIPKIYGGPISQHNYRPGYFTQTAGPWHLGKLELDFDLCEGTTVVVDEHCGNRGPSLRTTTVTGKIIHEWCCRSCTLPPLRFRGEDGCWYGMEIRPVKEKEENLVRSMVSAGSGEVDSFSLGILCVSIMIEEVMRSRWSRKMLMTGTLAVFLLLIMGQLTWNDLTRLCIMVGANASDRMGMGTTYLALMATFKMRPMFAVGLLFRRLTSREVLLLTIGLSLVASVELPNSLEELGDGLAMGIMMLKLLTEFQPHQLWTTLLSLTFVKTTLSLDYAWKTTAMVLSIVSLFPLCLSTTSQKTTWLPVLLGSFGCKPLTMFLITENKIWGRKSWPLNEGIMAIGIVSILLSSLLKNDVPLAGPLIAGGMLIACYVISGSSADLSLEKAAEVSWEQEAEHSGASHNILVEVQDDGTMKIKDEERDDTLTILLKATLLAVSGVYPMSIPATLFVWYFWQKKKQRSGVLWDTPSPPEVERAVLDDGIYRILQRGLLGRSQVGVGVFQDGVFHTMWHVTRGAVLMYQGKRLEPSWASVKKDLISYGGGWRFQGSWNTGEEVQVIAVEPGKNPKNVQTTPGTFKTPEGEVGAIALDFKPGTSGSPIVNREGKIVGLYGNGVVTTSGTYVSAIAQAKASQEGPLPEIEDEVFKKRNLTIMDLHPGSGKTRRYLPAIVREAIKRKLRTLILAPTRVVASEMAEALKGMPIRYQTTAVKSEHTGREIVDLMCHATFTMRLLSPVRVPNYNMIIMDEAHFTDPASIAARGYISTRVGMGEAAAIFMTATPPGSVEAFPQSNAVIQDEERDIPERSWNSGYDWITDFPGKTVWFVPSIKSGNDIANCLRKNGKRVIQLSRKTFDTEYQKTKNNDWDYVVTTDISEMGANFRADRVIDPRRCLKPVILKDGPERVILAGPMPVTVASAAQRRGRIGRNQNKEGDQYVYMGQPLNNDEDHAHWTEAKMLLDNINTPEGIIPALFEPEREKSAAVDGEYRLRGEARKTFVELMRRGDLPVWLSYKVASEGFQYSDRRWCFDGERNNQVLEENMDVEIWTKEGERKKLRPRWLDARTYSDPLALREFKEFAAGRRSVSGDLILEIGKLPQHLTLRAQNALDNLVMLHNSEQGGKAYRHAMEELPDTIETLMLLALIAVLTGGVTLFFLSGKGLGKTSIGLLCVTASSALLWMASVEPHWIAASIILEFFLMVLLIPEPDRQRTPQDNQLAYVVIGLLFMILTVAANEMGLLETTKKDLGIGYVAAENHQHATMLDVDLRPASAWTLYAVATTVITPMMRHTIENTTANISLTAIANQAAILMGLDKGWPISKMDIGVPLLALGCYSQVNPLTLTAAVLMLVAHYAIIGPGLQAKATREAQKRTAAGIMKNPTVDGIVAIDLDPVVYDAKFEKQLGQIMLLILCTSQILLMRTTWALCESITLATGPLTTLWEGSPGKFWNTTIAVSMANIFRGSYLAGAGLAFSLMKSLGGGRRGTGAQGETLGEKWKRQLNQLSKSEFNTYKRSGIMEVDRSEAKEGLKRGETTKHAVSRGTAKLRWFVERNLVKPEGKVIDLGCGRGGWSYYCAGLKKVTEVKGYTKGGPGHEEPIPMATYGWNLVKLHSGKDVFFMPPEKCDTLLCDIGESSPNPTIEEGRTLRVLKMVEPWLRGNQFCIKILNPYMPSVVETLEQMQRKHGGMLVRNPLSRNSTHEMYWVSCGTGNIVSAVNMTSRMLLNRFTMAHRKPTYERDVDLGAGTRHVAVEPEVANLDIIGQRIENIKNEHKSTWHYDEDNPYKTWAYHGSYEVKPSGSASSMVNGVVRLLTKPWDVIPMVTQIAMTDTTPFGQQRVFKEKVDTRTPRAKRGTTQIMEVTAKWLWGFLSRNKKPRICTREEFTRKVRSNAAIGAVFVDENQWNSAKEAVEDERFWDLVHRERELHKQGKCATCVYNMMGKREKKLGEFGKAKGSRAIWYMWLGARFLEFEALGFMNEDHWFSRENSLSGVEGEGLHKLGYILRDISKIPGGNMYADDTAGWDTRITEDDLQNEAKITDIMEPEHALLATSIFKLTYQNKVVRVQRPAKNGTVMDVISRRDQRGSGQVGTYGLNTFTNMEVQLIRQMESEGIFLPSELETPNLAERVLDWLEKHGAERLKRMAISGDDCVVKPIDDRFATALTALNDMGKVRKDIPQWEPSKGWNDWQQVPFCSHHFHQLIMKDGREIVVPCRNQDELVGRARVSQGAGWSLRETACLGKSYAQMWQLMYFHRRDLRLAANAICSAVPVEWVPTSRTTWSIHAHHQWMTTEDMLSVWNRVWIEENPWMEDKTHVSSWEEVPYLGKREDQWCGSLIGLTARATWATNIQVAINQVRRLIGNENYLDYMTSMKRFKNESDPEGALW

>DENV1_AHF50492_Argentina

MNNQRKKTGRPSFNMLKRARNRVSTGSQLAKRFSKGLLSGQGPMKLVMAFIAFLRFLAIPPTAGILARWSSFKKNGAIKVLRGFKKEISSMLNIMNRRKRSVTMLLMLLPTALAFHLTTRGGEPHMIVSKQERGKSLLFKTSAGVNMCTLIAMDLGELCEDTMTYKCPRITEAEPDDVDCWCNATDTWVTYGTCSQTGEHRRDKRSVALAPHVGLGLETRTETWMSSEGAWKQIQRVETWALRHPGFTVIALFLAHAIGTSITQKGIIFILLMLVTPSMAMRCVGIGNRDFVEGLSGATWVDVVLEHGSCVTTMAKNKPTLDIELLKTEVTNPAVLRKLCIEAKISNTTTDSRCPTQGEATLVEEQDANFVCRRTFVDRGWGNGCGLFGKGSLLTCAKFKCVTKLEGKIVQYENLKYSVIVTVHTGDQHQVGNETIEHGTIATITPQAPTSEIQLTDYGALTLDCSPRTGLDFNEMVLLTMKEKSWLVHKQWFLDLPLPWTSGASTPQETWNRQDLLVTFKTAHAKKQEVVVLGSQEGAMHTALTGATEIQTSGTTTIFAGHLKCRLKMDKLTLKGMSYVMCTGSFKLEKEVAETQHGTVLVQVKYEGTDAPCKIPFSTQDEKGVTQNGRLITANPIVTDKEKSVNIETEPPFGESYIVVGVGEKALKLSWFKKGSSIGKMFEATARGARRMAILGDTAWDFGSIGGVFTSVGKLVHQVFGTAYGVLFSGVSWTMKIGIGILLTWLGLNSRSTSLSMTCIAVGMVTLYLGVMVQADSGCVINWKGRELKCGSGIFVTNEVHTWTEQYKFQADSPKRLSAAIGKAWEEGVCGIRSATRLENIMWKQISNELNHILLENDMKFTVVVGDANGILAQGKKMIRPQPMEHKYSWKSWGKAKIIGADIQNTTFIIDGPDTPECPDGQRAWNIWEVEDYGFGIFTTNIWLKLRDSYTQMCDHRLMSAAIKDSKAVHADMGYWIESEKNETWKLARASFIEVKTCTWPKSHTLWSNGVLESEMIIPKIYGGPISQHNYRPGYFTQTAGPWHLGKLELDFDLCEGTTVVVDEHCGNRGPSLRTTTVTGKIIHEWCCRSCTLPPLRFRGEDGCWYGMEIRPVKEKEENLVRSMVSAGSGEVDSFSLGILCVSIMIEEVMRSRWSRKMLMTGTLAVFLLLIMGQLTWNDLIRLCIMVGANASDRMGMGTTYLALMATFKMRPMFAVGLLFRRLTSREVLLLTIGLSLVASVELPNSLEELGDGLAMGIMMLKLLTEFQPHQLWTTLLSLTFVKTTLSLDYAWKTTAMVLSIVSLFPLCLSTTSQKTTWLPVLLGSFGCNPLTMFLITENKIWGRKSWPLNEGIMAIGIVSILLSSLLKNDVPLAGPLIAGGMLIACYVISGSSADLSLEKAAEVSWEQEAEHSGASHNILVEVQDDGTMKIKDEERDDTLTILLKATLLAVSGVYPMSIPATLFVWYFWQKKKQRSGVLWDTPSPPKVERAVLDDGIYRILQRGLLGRSQVGVGVFQDGVFHTMWHVTRGAVLMYQGKRLEPSWASVKKDLISYGGGWRFQGSWNTGEEVQVIAVEPGKNPKNVQTTPGTFKTPEGEVGAIALDFKPGTSGSPIVNREGKIVGLYGNGVVTTSGTYVSAIAQAKASQEGPLPEIEDEVFKKRNLTIMDLHPGSGKTRRYLPAIVREAIKRKLRTLILAPTRVVASEMAEALKGMPIRYQTTAVKSEHTGREIVDLMCHATFTMRLLSPVRVPNYNMIIMDEAHFTDPASIAARGYISTRVGMGEAAAIFMTATPPGSVEAFPQSNAVIQDEERDIPERSWNSGYDWITDFPGKTVWFVPSIKSGNDIANCLRKNGKRVIQLSRKTFDTEYQKTKNNDWDYVVTTDISEMGANFRADRVIDPRRCLKPVILKDGPERVILAGPMPVTVASAAQRRGRIGRNQNKEGDQYVYMGQPLNNDEDHAHWTEAKMLLDNINTPEGIIPALFEPEREKSAAIDGEYRLRGEARKTFVELMRRGDLPVWLSYKVASEGFQYSDRRWCFDGERNNQVLEENMDVEIWTKEGERKKLRPRWLDARTYSDPLALREFKEFAAGRRSVSGDLILEIGKLPQHLTLRAQNALDNLVMLHNSEQGGKAYRHAMEELPDTIETLMLLALIAVLTGGVTLFFLSGKGLGKTSIGLLCVTASSALLWMASVEPHWIAASIILEFFLMVLLIPEPDRQRTPQDNQLAYVVIGLLFMILTVAANEMGLLETTKKDLGIGYVAAENHQHATMLDVDLRPASAWTLYAVATTVITPMMRHTIENTTANISLTAIANQAAILMGLDKGWPISKMDIGVPLLALGCYSQVNPLTLTAAVLMLVAHYAIIGPGLQAKATREAQKRTAAGIMKNPTVDGIVAIDLDPVVYDAKFEKQLGQIMLLILCTSQILLMRTTWALCESITLATGPLTTLWEGSPGKFWNTTIAVSMANIFRGSYLAGAGLAFSLMKSLGGGRRGTGAQGETLGEKWKRQLNQLSKSEFNTYKRSGIMEVDRSEAKEGLKRGETTKHAVSRGTAKLRWFVERNLVKPEGKVIDLGCGRGGWSYYCAGLKKVTEVKGYTKGGPGHEEPIPMATYGWNLVKLHSGKDVFFMPPEKCDTLLCDIGESSPNPTIEEGRTLRVLKMVEPWLRGNQFCIKILNPYMPSVVETLEQMQRKHGGMLVRNPLSRNSTHEMYWVSCGTGNIVSAVNMTSRMLLNRFTMAHRKPTYERDVDLGAGTRHVAVEPEVANLDIIGQRIENIKNEHKSTWHYDEDNPYKTWAYHGSYEVKPSGSASSMVNGVVRLLTKPWDVIPMVTQIAMTDTTPFGQQRVFKEKVDTRTPRAKRGTTQIMEVTAKWLWGFLSRNKKPRICTREEFTRKVRSNAAIGAVFVDENQWNSAKEAVEDERFWDLVHRERELHKQGKCATCVYNMMGKREKKLGEFGKAKGSRAIWYMWLGARFLEFEALGFMNEDHWFSRENSLSGVEGEGLHKLGYILRDISKIPGGNMYADDTAGWDTRITEDDLQNEAKITDIMEPEHALLATSIFKLTYQNKVVRVQRPAKNGTVMDVISRRDQRGSGQVGTYGLNTFTNMEVQLVRQMESEGIFLPSELETPNLAERVLDWLEKHGVERLKRMAISGDDCVVKPIDDRFATALTALNDMGKVRKDIPQWEPSKGWNDWQQVPFCSHHFHQLIMKDGREIVVPCRNQDELVGRARVSQGAGWSLRETACLGKSYAQMWQLMYFHRRDLRLAANAICSAVPVDWVPTSRTTWSIHAHHQWMTTEDMLSVWNRVWIEENPWMEDKTHVSSWEEVPYLGKREDQWCGSLIGLTARATWATNIQVAINQVRRLIGNENYLDYMTSMKRFKNESDPEGALW

>DENV1_AHF50491_Argentina

MNNQRKKTGRPSFNMLKRARNRVSTGSQLAKRFSKGLLSGQGPMKLVMAFIAFLRFLAIPPTAGILARWSSFKKNGAIKVLRGFKKEISSMLNIMNRRKRSVTMLLMLLPTALAFHLTTRGGEPHMIVSKQERGKSLLFKTSAGVNMCTLIAMDLGELCEDTMTYKCPRITEAEPDDVDCWCNATDTWVTYGTCSQTGEHRREKRSVALAPHVGLGLETRTETWMSSEGAWKQIQRVETWALRHPGFTVIAFFLAHAIGTSITQKGIIFILLMLVTPSMAMRCVGIGNRDFVEGLSGATWVDVVLEHGSCVTTMAKNKPTLDIELLKTEVTNPAVLRKLCIEAKISNTTTDSRCPTQGEATLVEEQDANFVCRRTFVDRGWGNGCGLFGKGSLLTCAKFKCVTKLEGKIVQYENLKYSVIVTVHTGDQHQVGNETTEHGTIATITPQAPTSEIQLTDYGALTLDCSPRTGLDFNEMVLLTMKEKSWLVHKQWFLDLPLPWTSGASTSQETWNRQDLLVTFKTAHAKKQEVVVLGSQEGAMHTALTGATEIQTSGTTTIFAGHLKCRLKMDKLTLKGMSYVMCTGSFKLEKEVAETQHGTVLVQVKYEGTDAPCKIPFSTQDEKGVTQNGRLITANPIVTDKEKPVNIETEPPFGESYIVVGAGEKALKLSWFKRGSSIGKMFEATARGARRMAILGDTAWDFGSIGGVFTSVGKLVHQVFGTAYGVLFSGVSWTMKIGIGILLTWLGLNSRSTSLSMTCIAVGMVTLYLGVMVQADSGCVINWKGRELKCGSGIFVTNEVHTWTEQYKFQADSPKRLSAAIGKAWEEGVCGIRSATRLENIMWKQISNELNHILLENDMKFTVVVGDANGILAQGKKMIRPQPMEHKYSWKSWGKAKIIGADIQNTTFIIDGPDTPECPDGQRAWNIWEVEDYGFGIFTTNIWLKLRDSYTQMCDHRLMSAAIKDSKAVHADMGYWIESEKNETWKLARASFIEVKTCTWPKSHTLWSNGVLESEMIIPKIYGGPISQHNYRPGYFTQTAGPWHLGKLELDFDLCEGTTVVVDEHCGNRGPSLRTTTVTGKIIHEWCCRSCTLPPLRFRGEDGCWYGMEIRPVKEKEENLVRSMVSAGSGEVDNFSLGILCVSIMIEEVMRSRWSRKMLMTGTLAVFLLLIMGQLTWNDLIRLCIMVGANASDRMGMGTTYLALMATFKMRPMFAVGLLFRRLTSREVLLLTIGLSLVASLELPNSLEELGDGLAMGIMMLKLLTEFQPHQLWTTLLSLTFVKTTLSLDYAWKTTAMVLSIVSLFPLCLSTTSQKTTWLPVLLGSFGCKPLTMFLITENKIWGRKSWPLNEGIMAIGIVSILLSSLLKNDVPLAGPLIAGGMLIACYVISGSSADLSLEKAAEVSWEQEAEHSGASHNILVEVQDDGTMKIKDEERDDTLTILLKATLLAVSGVYPMSIPATLFVWYFWQKKKQRSGVLWDTPSPPEVERAVLDDGIYRILQRGLLGRSQVGVGVFQDGVFHTMWHVTRGAVLMYQGKRLEPSWACVKKDLISYGGGWRFQGLWNTGEEVQVIAVEPGKNPKNVQTTPGTFKTPEGEVGAIALDFKPGTSGSPIVNREGKIVGLYGNGVVTTSGTYVSAIAQAKASQEGPLPEIEDEVFKKRNLTIMDLHPGSGKTRRYLPAIVREAIKRKLRTLILAPTRVVASEMAEALKGMPIRYQTTAVKSEHTGREIVDLMCHATFTMRLLSPVRVPNYNMIIMDEAHFTDPASIAARGYISTRVGMGEAAAIFMTATPPGSVEAFPQSNAVIQDEERDIPERSWNSGYDWITDFPGKTVWFVPSIKSGNDIANCLRKNGKRVIQLSRKTFDTEYQKTKNNDWDYVVTTDISEMGANFRADRVIDPRRCLKPVILKDGPERVILAGPMPVTVASAAQRRGRIGRNQNKEGDQYVYMGQPLNNDEDHAHWTEAKMLLDNINTPEGIIPALFEPEREKSAAIDGEYRLRGEARKTFVELMRRGDLPVWLSYKVASEGFQYSDRRWCFDGERNNQVLEENMDVEIWTKEGERKKLRPRWLDARTYSDPLALREFKEFAAGRRSVSGDLILEIGKLPQHLTLRAQNALDNLVMLHNSEQGGKAYRHAMEELPDTIETLMLLALIAVLTGGVTLFFLSGKGLGKTSIGLLCVTASSALLWMASVEPHWIAASIILEFFLMVLLIPEPDRQRTPQDNQLAYVVIGLLFMILTVAANEMGLLETTKKDLGIGHVAAENHQHATMLDVDLHPASAWTLYAVATTVITPMMRHTIENTTANISLTAIANQAAILMGLDKGWPISKMDIGVPLLALGCYSQVNPLTLTAAVLMLVAHYAIIGPGLQAKATREAQKRTAAGIMKNPTVDGIVAIDLDPVVYDAKFEKQLGQIMLLILCTSQILLMRTTWALCESITLATGPLTTLWEGSPGKFWNTTIAVSMANIFRGSYLAGAGLAFSLMKSLGGGRRGTGAQGETLGEKWKRQLNQLSKSEFNTYKRSGIMEVDRSEAKEGLKRGETTKHAVSRGTAKLRWFVERNLVKPEGKVIDLGCGRGGWSYYCAGLKKVTEVKGYTKGGPGHEEPIPMATYGWNLVKLHSGKDVFFMPPEKCDTLLCDIGESSPNPTIEEGRTLRVLKMVEPWLRGNQFCIKILNPYMPSVVETLEQMQRKHGGMLVRNPLSRNSTHEMYWVSRGTGNIVSAVNMTSRMLLNRFTMAHRKPTYERDVDLGAGTRHVAVEPEVANLDIIGQRIENIKNEHKSTWHYDEDNPYKTWAYHGSYEVKPSGSASSMVNGVVRLLTKPWDVIPMVTQIAMTDTTPFGQQRVFKEKVDTRTPRAKRGTTQIMEVTAKWLWGFLSRNKKPRICTREEFTRKVRSNAAIGAVFVDEKQWNSAKEAVEDERFWDLVHRERELHKQGKCATCVYNMMGKREKKLGEFGKAKGSRAIWYMWLGARFLEFEALGFMNEDHWFSRENSLSGVEGEGLHKLGYILRDISKIPGGNMYADDTAGWDTRITEDDLQNEAKITDIMEPEHALLATSIFKLTYQNKVVRVQRPAKNGTVMDVISRRDQRGSGQVGTYGLNTFTNMEVQLIRQMESEGIFLPSELETPNLAERVLDWLEKHGAERLKRMAISGDDCVVKPIDDRFATALTALNDMGKVRKDIPQWEPSKGWNDWQQVPFCSHHFHQLIMKDGREIVVPCRNQDELVGRARVSQGAGWSLRETACLGKSYAQMWQLMYFHRRDLRLAANAICSAVPVDWVPTSRTTWSIHAHHQWMTTEDMLSVWNRVWIEENPWMEDKTHVSSWEEVPYLGKREDQWCGSLIGLTARATWATNIQVAINQVRRLIGNENYLDYMTSMKRFKNESDPEGALW

>DENV1_AET43256_Venezuela

MNNQRKKTGRPSFNMLKRARNRVSTGSQLAKRFSKGLLSGQGPMKLVMAFIAFLRFLAIPPTAGILARWSSFKKNGAIKVLRGFKKEISSMLNIMNRRKRSVTMLLMLLPTALAFHLTTRGGEPHMIVSKQERGKSLLFKTSAGVNMCTLIAMDLGELCEDTMTYKCPRITEAEPDDVDCWCNATDTWVTYGTCSQTGEHRREKRSVALAPHVGLGLETRTETWMSSEGAWKQIQRVETWALRHPGFTVIAFFLAHAIGTSITQKGIIFILLMLVTPSMAMRCVGIGNRDFVEGLSGATWVDVVLEHGSCVTTMAKNKPTLDIELLKTEVTNPAVLRKLCIEAKISNTTTDSRCPTQGEATLVEEQDANFVCRRTFVDRGWGNGCGLFGKGSLLTCAKFKCVTKLEGKIVQYENLKYSVIVTVHTGDQHQVGNETTEHGTIATITPQAPTSEIQLTDYGALTLDCSPRTGLDFNEMVLLTMKEKSWLVHKQWFLDLPLPWTSGASTSQETWNRQDLLVTFKTAHAKKQEVVVLGSQEGAMHTALTGATEIQTSGTTTIFAGHLKCRLKMDKLTLKGMSYVMCTGSFKLEKEVAETQHGTVLVQVKYEGTDAPCKIPFSTQDEKGVTQNGRLITANPIVTDKEKPVNIETEPPFGESYIVVGAGEKALKLSWFKRGSSIGKMFEATARGARRMAILGDTAWDFGSIGGVFTSVGKLVHQVFGTAYGVLFSGVSWTMKIGIGILLTWLGLNSRSTSLSMTCIAVGMVTLYLGVMVQADSGCVINWKGRELKCGSGIFVTNEVHTWTEQYKFQADSPKRLSAAIGKAWEEGVCGIRSATRLENIMWKQISNELNHILLENDMKFTVVVGDANGILAQGKKMIRPQPMEHKYSWKSWGKAKIIGADIQNTTFIIDGPDTPECPDGQRAWNIWEVEDYGFGVFTTNIWLKLRDSYTQMCDHRLMSAAIKDSKAVHADMGYWIESEKNETWKLARASFIEVKTCTWPKSHTLWSNGVLESEMIIPKIYGGPISQHNYRPGYFTQTAGPWHLGKLELDFDLCEGTTVVVDEHCGNRGPSLRTTTVTGKIIHEWCCRSCTLPPLRFRGEDGCWYGMEIRPVKEKEENLVRSMVSAGSGEVDSFSLGILCVSIMIEEVMRSRWSRKMLMTGTLAVFLLLIMGQLTWNDLIRLCIMVGANASDRMGMGTTYLALMATFKMRPMFAVGLLFRRLTSREVLLLTIGLSLVASVELPNSLEELGDGLAMGIMMLKLLTEFQPHQLWTTLLSLTFVKTTLSLDYAWKTTAMVLSIVSLFPLCLSTTSQKTTWLPVLLGSFGCKPLTMFLITENKIWGRKSWPLNEGIMAIGIVSILLSSLLKNDVPLAGPLIAGGMLIACYVISGSSADLSLEKAVKISWEQEAEHSGASHNILVEVQDDGTMKIKDEERDDTLTILLKATLLAVSGVYPMSIPATLFVWYFWQKKKQRSGVLWDTPSPPEVERAVLDDGIYRILQRGLLGRSQVGVGVFQDGVFHTMWHVTRGAVLMYQGKRLEPSWASVKKDLISYGGGWRFQGSWNTGEEVQVIAVEPGKNPKNVQTTPGTFKTPEGEVGAIALDFKPGTSGSPIVNREGKIVGLYGNGVVTTSGTYVSAIAQAKASQEGPLPEIEDEVFKKRNLTIMDLHPGSGKTRRYLPAIVREAIKRKLRTLILAPTRVVASEMAEALKGMPIRYQTTAVKSEHTGREIVDLMCHATFTMRLLSPVRVPNYNMIIMDEAHFTDPASIAARGYISTRVGMGEAAAIFMTATPPGSVEAFPQSNAVIQDEERDIPERSWNSGYDWITDFPGKTVWFVPSIKSGNDIANCLRKNGKRVIQLSRKTFDTEYQKTKNNDWDYVVTTDISEMGANFRADRVIDPRRCLKPVILKDGPERVILAGPMPVTVASAAQRRGRIGRNQNKEGDQYVYMGQPLNNDEDHAHWTEAKMLLDNINTPEGIIPALFEPEREKSAAIDGEYRLRGEARKTFVELMRRGDLPVWLSYKVASEGFQYSDRRWCFDGERNNQVLEENMDVEIWTKEGERKKLRPRWLDARTYSDPLALREFKEFAAGRRSVSGDLILEIGKLPQHLTLRAQNALDNLVMLHNSEQGGKAYRHAMEELPDTIETLMLLALIAVLTGGVTLFFLSGKGLGKTSIGLLCVTASSALLWMASVEPHWIAASIILEFFLMVLLIPEPDRQRTPQDNQLAYVVIGLLFMILTVAANEMGLLETTKKDLGIGYVAAENHQHATMLDVDLHPASAWTLYAVATTVITPMMRHTIENTTANISLTAIANQAAILMGLDKGWPISKMDIGVPLLALGCYSQVNPLTLTAAVLMLVAHYAIIGPGLQAKATREAQKRTAAGIMKNPTVDGIVAIDLDPVVYDAKFEKQLGQIMLLILCTSQILLMRTTWALCESITLATGPLTTLWEGSPGKFWNTTIAVSMANIFRGSYLAGAGLAFSLMKSLGGGRRGTGAQGETLGEKWKRQLNQLSKSEFNTYKRSGIMEVDRSEAKEGLKRGETTKHAVSRGTAKLRWFVERNLVKPEGKVIDLGCGRGGWSYYCAGLKKVTEVKGYTKGGPGHEEPIPMATYGWNLVKLHSGKDVFFMPPEKCDTLLCDIGESSPNPTIEEGRTLRVLKMVEPWLRGNQFCIKILNPYMPSVVETLEQMQRKHGGMLVRNPLSRNSTHEMYWVSCGTGNIVSAVNMTSRMLLNRFTMAHRKPTYERDVDLGAGTRHVAVEPEVANLDIIGQRIENIKNEHKSTWHYDEDNPYKTWAYHGSYEVKPSGSASSMVNGVVRLLTKPWDVIPMVTQIAMTDTTPFGQQRVFKEKVDTRTPRAKRGTTQIMEVTAKWLWGFLSRNKKPRICTREEFTRKVRSNAAIGAVFVDENQWNSAKEAVEDERFWDLVHRERELHKQGKCATCVYNMMGKREKKLGEFGKAKGSRAIWYMWLGARFLEFEALGFMNEDHWFSRENSLSGVEGEGLHKLGYILRDISKIPGGNMYADDTAGWDTRITEDDLQNEAKITDIMEPEHALLATSIFKLTYQNKVVRVQRPAKNGTVMDVISRRDQRGSGQVGTYGLNTFTNMEVQLIRQMESEGIFLPSELETPNLAERALDWLEKHGAERLKRMAISGDDCVVKPIDDRFATALTALNDMGKVRKDIPQWEPSKGWNDWQQVPFCSHHFHQLIMKDGREIVVPCRNQDELVGRARVSQGAGWSLRETACLGKSYAQMWQLMYFHRRDLRLAANAICSAVPVDWVPTSRTTWSIHAHHQWMTTEDMLSVWNRVWIEENPWMEDKTHVSSWEEVPYLGKREDQWCGSLIGLTARATWATNIQVAINQVRRLIGNENYLDYMTSMKRFKNESDPEGALW

>DENV1_AFJ91714_USA

MNNQRKKTGRPSFNMLKRARNRVSTGSQLAKRFSKGLLSGQGPMKLVMAFIAFLRFLAIPPTAGILARWSSFKKNGAIKVLRGFKKEISSMLNIMNRRKRSVTMLLMLLPTALAFHLTTRGGEPHMIVSKQERGKSLLFKTSAGVNMCTLIAMDLGELCEDTMTYKCPQITEAEPDDVDCWCNATDTWVTYGTCSQTGEHRRDKRSVALAPHVGLGLETRTETWMSSEGAWKQIQRVETWALRHPGFTVIALFLAHAIGTSITQKGIIFILLMLVTPSMAMRCVGIGNRDFVEGLSGATWVDVVLEHGSCVTTMAKNKPTLDIELLKTEVTNPAILRKLCIEAKISNTTTDSRCPTQGEATLVEEQDANFVCRRTFVDRGWGNGCGLFGKGSLLTCAKFKCVTKLEGKIVQYENLKYSVIVTVHTGDQHQVGNETTEHGTIATITPQAPTSEIQLTDYGALTLDCSPRTGLDFNEMVLLTMKERSWLVHKQWFLDLPLPWTSGASTSQETWNRQDLLVTFKTAHAKKQEVVVLGSQEGAMHTALTGATEIQTSGTTTIFAGHLKCRLKMDKLTLKGTSYVMCTGSFKLEKEVAETQHGTVLVQVKYEGTDAPCKIPFSTQDEKGVTQNGRLITANPIVTDKEKPVNIETEPPFGESYIVVGAGEKALKLSWFKKGSSIGKMFEATARGARRMAILGDTAWDFGSIGGVFTSVGKLVHQVFGTAYGVLFSGVSWTMKIGIGILLTWLGLNSRSTSLSMTCIAVGMVTLYLGVMVQADSGCVINWKGRELKCGSGIFVTNEVHTWTEQYKFQADSPKRLSAAIGKAWEEGVCGIRSATRLENIMWKQISNELNHILLENDMKFTVVVGDANGILAQGKKMIRPQPMEHKYSWKSWGKAKIIGADIQNTTFIIDGPDTPECPDNQRAWNIWEVEDYGFGIFTTNIWLKLRDSYTQMCDHRLMSAAIKDSKAVHADMGYWIESEKNETWKLARASFIEVKTCTWPKSHTLWSNGVLESEMIIPKTYGGPISQHNYRPGYFTQTAGPWHLGKLELDFDLCEGTTVVVDEHCGNRGPSLRTTTVTGKIIHEWCCRSCTLPPLRFRGEDGCWYGMEIRPVKEKEENLVRSMVSAGSGEVDSFSLGILCASIMIEEVMRSRWSRKMLMTGTLAVFLLLIMGQLTWNDLIRLCIMVGANASDRMGMGTTYLALMATFKMRPMFAVGLLFRRLTSREVLLLTIGLSLVASVELPNSLEELGDGLAMGIMMLKLLTEFQPHQLWTTLLSLTFIKTTLSLDYAWKTTAMALSIVSLFPLCLSTTSQKTTWLPVLLGSFGCKPLTMFLITENKIWGRKSWPLNEGIMAIGIVSLLLSSLLKNDVPLAGPLIAGGMLIACYVISGSSADLSLEKAAEVSWEQEAEHSGTSHNILVEVQDDGTMKIKDEERDDTLTILVKATLLAVSGVYPMSIPATLFVWYFWQKKKQRSGVLWDTPSPPEVERAVLDDGIYRILQRGLLGRSQVGVGVFQDGVFHTMWHVTRGAVLMYQGKRLEPSWASVKKDLISYGGGWRFQGSWNTGEEVQVIAVEPRKNPKNVQTTPGTFKTPEGEVGAIALDFKPGTSGSPIVNREGKIVGLYGNGVVTTSGTYVSAIAQAKASQEGPLPEIEDEVFKKRNLTIMDLHPGSGKTRRYLPAIVREAIKRKLRTLILAPTRVVASEMAEALKGMPIRYQTTAVKSEHTGREIVDLMCHATFTMRLLSPVRVPNYNMIIMDEAHFTDPASIAARGYISTRVGMGEAAAIFMTATPPGSVEAFPQSNAVIQDEERDIPERSWNSGYDWITDFPGKTVWFVPSIKSGNDIANCLRKNGKRVIQLSRKTFDTEYQKTKNNDWDYVVTTDISEMGANFRADRVIDPRRCLKPVILKDGPERVILAGPMPVTVASAAQRRGRIGRNQNKEGDQYVYMGQPLNNDEDHAHWTEAKMLLDNINTPEGIIPALFEPEREKSAAIDGEYRLRGEARKTFVELMRRGDLPVWLSYKVASEGFQYSDRRWCFDGERNNQVLEENMDVEIWTKEGERKKLRPRWLDARTYSDPLALREFKEFAAGRRSVSGDLILEIGKLPQHLTLRAQNALDNLVMLHNSEQGGKAYRHAMEELPDTIETLMLLALIAVLTGGVTLFFLSGKGLGKTSIGLLCVTASSALLWMASVEPHWIAASIILEFFLMVLLIPEPDRQRTPQDNQLAYVVIGLLFMILTVAANEMGLLETTKKDLGIGHVAAENHQHATMLDVDLRPASAWTLYAVATTVITPMMRHTIENTTANISLTAIANQAAILMGLDKGWPISKMDIGVPLLALGCYSQVNPLTLTAAVLMLVAHYAIIGPGLQAKATREAQKRTAAGIMKNPTVDGIVAIDLDPVVYDTKFEKQLGQIMLLILCTSQILLMRTTWALCESITLATGPLTTLWEGSPGKFWNTTIAVSMANIFRGSYLAGAGLAFSLMKSVGGGRRGMGAQGETLGEKWKRQLNQLSKSEFNTYKRSGIMEVDRSEAKEGLKRGETTKHAVSRGTAKLRWFVERNLVKPEGKVIDLGCGRGGWSYYCAGLKKVTEVKGYTKGGPGHEEPIPMATYGWNLVKLHSGKDVFFMPPEKCDTLLCDIGESSPNPTIEEGRTLRVLKMVEPWLRGNQFCIKILNPYMPSVVETLEQMQRKHGGMLVRNPLSRNSTHEMYWVSCGTGNIVSAVNMTSRMLLNRFTMAHRKPTYERDVDLGAGTRHVAVEPEVANLDIIGQRIENIKNEHKSTWHYDEDNPYKTWAYHGSYEVKPSGSASSMVNGVVRLLTKPWDVIPMVTQIAMTDTTPFGQQRVFKEKVDTRTPRAKRGTTQIMEVTAKWLWGFLSRNKKPRICTREEFTRKVRSNAAIGAVFVDENQWNSAKEAVEDERFWDLVHRERELHKQGKCATCVYNMMGKREKKLGEFGKAKGSRAIWYMWLGARFLEFEALGFMNEDHWFSRENSLSGVEGEGLHKLGYILRDISKIPGGNMYADDTAGWDTRITEDDLQNEAKITDIMEPEHALLATSIFKLTYQNKVVRVQRPAKNGTVMDVISRRDQRGSGQVGTYGLHTFTNMEVQLIRQMESEGIFLPSELETPNLAERVLDWLEKHGAERLKRMAISGDDCVVKPIDDRFATALTALNDMGKVRKDIPQWEPSKGWNDWQQVPFCSHHFHQLIMKDGREIVVPCRNQDELVGRARVSQGAGWSLRETACLGKSYAQMWQLMYFHRRDLRLAANAICSAVPVDWVPTSRTTWSIHAHHQWMTTEDMLSVWNRVWIEENPWMEDKTHVSSWEEVPYLGKREDQWCGSLIGLTARATWATNIQVAINQVRRLIGNENYLDYMTSMKRFKNESDPEGALW

>DENV1__ALJ53459_EUA

MNNQRKKTGRPSFNMLKRARNRVSTGSQLAKRFSKGLLSGQGPMKLVMAFIAFLRFLAIPPTAGILARWSSFKKNGAIKVLRGFKKEISSMLNIMNRRKRSVTMLLMLLPTALAFHLTTRGGEPHMIVSKQERGKSLLFKTSAGVNMCTLIAMDLGELCEDTMTYKCPRITEAEPDDVDCWCNATDTWVTYGTCSQTGEHRREKRSVALAPHVGLGLETRTETWMSSEGAWKQIQRVETWALRHPGFTVIAFFLAHAIGTSITQKGIIFILLMLVTPSMAMRCVGIGNRDFVEGLSGATWVDVVLEHGSCVTTMAKNKPTLDIELLKTEVTNPAVLRKLCIEAKISNTTTDSRCPTQGEATLVEEQDANFVCRRTFVDRGWGNGCGLFGKGSLLTCAKFKCVTKLEGKIVQYENLKYSVIVTVHTGDQHQVGNETTEHGTIATITPQAPTSEIQLTDYGALTLDCSPRTGLDFNEMVLLTMKEKSWLVHKQWFLDLPLPWTSGASTSQETWNRQDLLVTFKTAHAKKQEVVVLGSQEGAMHTALTGATEIQTSGTTTIFAGHLKCRLKMDKLTLKGMSYVMCTGSFKLEKEVAETQHGTVLVQVKYEGTDAPCKIPFSTQDEKGVTQNGRLITANPIVTDKEKPVNIETEPPFGESYIVVGAGEKALKLSWFKRGSSIGKMFEATARGARRMAILGDTAWDFGSIGGVFTSVGKLVHQIFGTAYGVLFSGVSWTMKIGIGILLTWLGLNSRSTSLSMTCIAVGMVTLYLGVMVQADSGCVINWKGRELKCGSGIFVTNEVHTWTEQYKFQADSPKRLSAAIGKAWEEGVCGIRSATRLENIMWKQISNELNHILLENDMKFTVVVGDANGILAQGKKMIRPQPMEHKYSWKSWGKAKIIGADIQNTTFIIDGPDTPECPDGQRAWNIWEVEDYGFGVFTTNIWLKLRDSYTQMCDHRLMSAAIKDSKAVHADMGYWIESEKNETWKLARASFIEVKTCTWPKSHTLWSNGVLESEMIIPKIYGGPISQHNYRPGYFTQTAGPWHLGKLELDFDLCEGTTVVVDEHCGNRGPSLRTTTVTGKIIHEWCCRSCTLPPLRFRGEDGCWYGMEIRPVKEKEENLVRSMVSAGSGEVDSFSLGILCVSIMIEEVMRSRWSRKMLMTGTLAVFLLLIMGQLTWNDLIRLCIMVGANASDRMGMGTTYLALMATFKMRPMFAVGLLFRRLTSREVLLLTIGLSLVASVELPNSLEELGDGLAMGIMMLKLLTEFQPHQLWTTLLSLTFVKTTLSLDYAWKTTAMALSIVSLFPLCLSTTSQKTTWLPVLLGSFGCKPLTMFLITENKIWGRKSWPLNEGIMAIGIVSILLSSLLKNDVPLAGPLIAGGMLIACYVISGSSADLSLEKAAEVSWEQEAEHSGASHSILVEVQDDGTMKIKDEERDDTLTILLKATLLAVSGVYPMSIPATLFVWYFWQKKKQRSGVLWDTPSPPEVERAVLDNGIYRILQRGLLGRSQVGVGVFQDGVFHTMWHVTRGAVLMYQGKRLEPSWASVKKDLISYGGGWRFQGSWNTGEEVQVIAVEPGKNPKNVQTTPGTFKTPEGEVGAIALDFKPGTSGSPIVNREGKIVGLYGNGVVTTSGTYVSAIAQAKASQEGPLPEIEDEVFKKRNLTIMDLHPGSGKTRRYLPAIVREAIKRKLRTLILAPTRVVASEMAEALKGMPIRYQTTAVKSEHTGREIVDLMCHATFTMRLLSPVRVPNYNMIIMDEAHFTDPASIAARGYISTRVGMGEAAAIFMTATPPGSVEAFPQSNAVIQDEERDIPERSWNSGYDWITDFPGKTVWFVPSIKSGNDIANCLRKNGKRVIQLSRKTFDTEYQKTKNNDWDYVVTTDISEMGANFRADRVIDPRRCLKPVILKDGPERVILAGPMPVTAASAAQRRGRIGRNQNKEGDQYVYMGQPLNNDEDHAHWTEAKMLLDNINTPEGIIPALFEPEREKSAAIDGEYRLRGEARKTFVELMRRGDLPVWLSYKVASEGFQYSDRRWCFDGERNNQVLEENMDVEIWTKEGERKKLRPRWLDARTYSDPLALREFKEFAAGRRSVSGDLILEIGKLPQHLTLRAQNALDNLVMLHNSEQGGKAYRHAMEELPDTIETLMLLALIAVLTGGVTLFFLSGKGLGKTSIGLLCVTASSALLWMASVEPHWIAASIILEFFLMVLLIPEPDRQRTPQDNQLAYVVIGLLFMILTVAANEMGLLETTKKDLGIGYVAAENHQHATMLDVDLHPASAWTLYAVATTVITPMMRHTIENTTANISLTAIANQAAILMGLDKGWPISKMDIGVPLLALGCYSQVNPLTLTAAVLMLVAHYAIIGPGLQAKATREAQKRTAAGIMKNPTVDGIVAIDLDPVVYDAKFEKQLGQIMLLILCTSQILLMRTTWALCESITLATGPLTTLWEGSPGKFWNTTIAVSMANIFRGSYLAGAGLAFSLMKSLGGGRRGTGAQGETLGEKWKRQLNQLSKSEFNTYKRSGIMEVDRSEAKEGLKRGETTKHAVSRGTAKLRWFVERNLVKPEGKVIDLGCGRGGWSYYCAGLKKVTEVKGYTKGGPGHEEPIPMATYGWNLVKLHSGKDVFFMPPEKCDTLLCDIGESSPNPTIEEGRTLRVLKMVEPWLRGNQFCIKILNPYMPSVVETLERMQRKHGGMLVRNPLSRNSTHEMYWVSCGTGNIVSAVNMTSRMLLNRFTMAHRKPTYERDVDLGAGTRHVAVEPEVANLDIIGQRIENIKNEHKSTWHYDEDNPYKTWAYHGSYEVKPSGSASSMVNGVVRLLTKPWDVIPMVTQIAMTDTTPFGQQRVFKEKVDTRTPRAKRGTTQIMEVTAKWLWGFLSRNKKPRICTREEFTRKVRSNAAIGAVFVDENQWNSAKEAVEDERFWDLVHRERELHKQGKCATCVYNMMGKREKKLGEFGKAKGSRAIWYMWLGARFLEFEALGFMNEDHWFSRENSLSGVEGEGLHKLGYILRDISKIPGGNMYADDTAGWDTRVTEDDLQNEAKITDIMEPEHALLATSIFKLTYQNKVVRVQRPAKNGTVMDVISRRDQRGSGQVGTYGLNTFTNMEVQLIRQMESEGIFLPSELETPNLAERALDWLEKHGAERLKRMAISGDDCVVKPIDDRFATALTALNDMGKVRKDIPQWEPSKGWNDWQQVPFCSHHFHQLIMKDGREIVVPCRNQDELVGRARVSQGAGWSLRETACLGKSYAQMWQLMYFHRRDLRLAANAICSAVPVDWVPTSRTTWSIHAHHQWMTTEDMLSVWNRVWIEENPWMENKTHVSSWEEVPYLGKREDQWCGSLIGLTARATWATNIQVAINQVRRLIGNENYLDYMTSMKRFKNESDSEGALW

>DENV1_ACF49259_USA

MNNQRKKTGRPSFNMLKRARNRVSTVSQLAKRFSKGLLSGQGPMKLVMAFMAFLRFLAIPPTAGILARWGSLKKNGAIKVLRGFKKEISNMLSIMNRRKRSVTMLLMLLPTALAFHLTTRGGEPHMIVSKQERGKSLLFKTSAGVNMCTIIAMDLGELCEDTMTYKCPRITKAEPDDVDCWCNATDTWVTYGTCSRTGEHRRDKRSVALAPHVGLGLETRAETWMSSEGAWKQIQKVETWALRHPGFTVIALFLAHAIGTSITQKGIIFILLMLVTPSMAMRCVGIGNRDFVEGLSGATWVDVVLEHGSCVTTMAKDKPTLDIELLKTEVTKPAVLRKLCIEAKISNTTTDSRCPTQGEATLVEEQDANFVCRRTFVDRGWGNGCGLFGKGSLITCAKFKCVTKLEGKIVQYENLKYSVIVTVHTGDQHQVGNETTEHGTIATITPQAPTSEIQLTDYGALTLDCSPRTGLDFNEMVLLTMKEKSWLVHKQWFLDLPLPWTSGASTPQETWNREDLLVTFKTAHAKKQEVVVLGSQEGAMHTALTGATEIQTSGTTKIFAGHLKCRLKMNKLTLKGMSYVMCTGSFKLEKEVAETQHGTVLVQVKYEGTDAPCKIPFSTQDEKGVTQNGRLITANPIVTDKEKPVNIEAEPPFGESYIVVGAGEKALKLSWFKKGSSIGKMLEATARGARRMAILGDTAWDFGSIGGVFTSVGKLVHQIFGTAYGVLFSGVSWTMKIGIGILLTWLGLNSRSASLSMTCIAVGMVTLYLGVMVQADSGCVINWKGRELKCGSGIFVTNEVHTWTEQYKFQADSPKRLSAAIGKAWEEGVCGIRSATRLENIMWKQISNELNHILLENDMKFTVVVGDVSGILTQGRKMIGPQPMEHKYSWKSWGKAKIIGADVQNTTFIIDGPNTPECPDDQRAWNIWEVEDYGFGIFTTNIWLKLRDSYTQVCDPRLMSAAIKDSKAVHADMGYWIESEKNETWKLARASFIEVKTCVWPKSHTLWSNGVLESEMIIPKIYGGPISQHNYRPGYSTQTAGPWHLGKLELDFDLCEGTTVVVDEHCGNRGPSLRTTTVTGKIIHEWCCRSCTLPPLRFKGEDGCWYGMEIRPVKDKEENLVKSLVSAGSGEVDSFSLGLLCISIMIEEVMRSRWSRKMLMTGTLAVFLLLIMGQLTWNDLIRLCIMVGANASDRMGMGTTYLALMATFKMRPMFAVGLLFRRLTSREVLLLTIGLSLVASVELPNSLEELGDGLAMGIMILKLLTDFQSHQLWAALLSLTFIKTTFSLHYAWKTVAMVLSIVSLFPLCLSTTSQKTTWLPVLLGSLGCKPLTMFLIAENKIWGRKSWPLNEGIMAVGIVSILLSSLLKNDVPLAGPLIAGGMLIACYVISGSSADLSLEKAAEISWEEEAEHSGASHNILVEVQDDGTMKIKDEERDDTLTILLKATLLAVSGVYPISIPATLFVWYFWQKKKQRSGVLWDTPSPPEVERAVLDDGIYRILQRGLLGRSQVGVGVFQEGVFHTMWHVTRGAVLMYQGKRLEPSWASVKKDLISYGGGWRFQGSWNTGEEVQVIAVEPGKNPKNVQTAPGTFKTPEGEVGAIALDFKPGTSGSPIVNREGKIVGLYGNGVVTTSGTYVSAIAQAKASQEGPLPEIEDEVFRKRNLTIMDLHPGSGKTRRYLPAIVREAIKRKLRTLILAPTRVVASEMAEALKGMPIRYQTTAVKSEHTGREIVDLMCHATFTMRLLSPVRVPNYNMIIMDEAHFTDPASIAARGYISTRVGMGEAAAIFMTATPPGSVEAFPQSNAVIQDEERDIPERSWNSGYDWITDFPGKTVWFVPSIKSGNDIANCLRKNGKRVIQLSRKTFDTEYQKTKNNDWDYVVTTDISEMGANFRADRVIDPRRCLKPVILKDGPERVILAGPMPVTVASAAQRRGRIGRNQNKEGDQYIYMGQPLNNDEDHAHWTEAKMLLDNINTPEGIIPALFEPERGKSAAIDGEYRLRGEARKTFVELMRRGDLPVWLSYKVASEGFQYSDRKWCFDGERNNQVLEENMDVEIWTKEGERKKLRPRWLDARTYSDPQALREFKEFAAGRRSVSGDLILEIGKLPQHLTQRAQNALDNLVMLHNSEQGGKAYRHAMEELPDTIETLMLLALIAVLTGGVTLFFLSGRGLGKTSIGLLCVMASSVLLWVASVEPHWIAASIILEFFLMVLLIPEPDRQRTPQDNQLAYVVIGLLFMILTVAANEMGLLETTKKDLGIGHVAVENHHHATMLDVDLRPASAWTLYAVATTIITPMMRHTIENTTANISLTAIANQAAILMGLDKGWPISKMDIGVPLLALGCYSQVNPLTLIAAVLMLVAHYAIIGPGLQAKATREAQKRTAAGIMKNPTVDGIVAIDLDPVVYDAKFEKQLGQIMLLILCTSQILLMRTTWALCESITLATGPLTTLWEGSPGKFWNTTIAVSMANIFRGSYLAGAGLAFSLMKSLGGGRRGTGAQGETLGEKWKRQLNQLSKSEFNTYKRSGIMEVDRSEAKEGLKRGETTKHAVSRGTAKLRWFVERNLVKPEGKVIDLGCGRGGWSYYCAGLKKVTEVKGYTKGGPGHEEPIPMATYGWNLVKLHSGKDVFFTPPEKCDTLLCDIGESSPNPTIEEGRTLRVLKMVEPWLRGNQFCIKILNPYMPSVVETLEQMQRKHGGMLVRNPLSRNSTHEMYWVSCGTGNIVSAVNMTSRMLLNRFTMAHRKPTYERDVDLGAGTRHVAVEPEVANLDIIGQRIENIKNEHKSTWHYDEDNPYKTWAYHGSYEVKPSGSASSMVNGVVRLLTKPWDVIPMVTQIAMTDTTPFGQQRVFKEKVDTRTPKAKRGTAQIMEVTARWLWGFLSRNKKPRICTREEFTRKVRSNAAIGAVFVDENQWNSAKEAVEDERFWDLVHRERELHKQGKCATCVYNMMGKREKKLGEFGKAKGSRAIWYMWLGARFLEFEALGFMNEDHWFSRENSLSGVEGEGLHKLGYILRDISKIPGGNMYADDTAGWDTRITEDDLQNEAKITDIMEPEHALLATSIFKLTYQNKVVRVQRPAKNGTVMDVISRRDQRGSGQVGTYGLNTFTNMEVQLIRQMESEGIFSPSELETPNLAERVLDWLEKHGVERLKRMAISGDDCVVKPTDDRFATALTALNDMGKVRKDIPQWEPSKGWNDWQQVPFCSHHFHQLIMKDGREIVVPCRNQDELVGRARVSQGAGWSLRETACLGKSYAQMWQLMYFHRRDLRLAANAICSAVPIDWVPTSRTTWSIHAHHQWMTTEDMLSVWNRVWIEENPWMEDKTHVSSWEDVPYLGKREDQWCGSLIGLTARATWATNIQVAINQVRRLIGNENYLDYMTSMKRFKNESDLEGALW

>DENV1_AEA50936_Venezuela

MNNQRKKTGRPSFNMLKRARNRVSTGSQLAKRFSKGLLSGQGPMKLVMAFIAFLRFLAIPPTAGILARWSSFKKNGAIKVLRGFKKEISSMLNIMNRRKRSVTMLLMLLPTALAFHLTTRGGEPHMIVSKQERGKSLLFKTSAGVNMCTLIAMDLGELCEDTMTYKCPRITEAEPDDVDCWCNATDTWVTYGTCSQTGEHRRDKRSVALAPHVGLGLETRTETWMSSEGAWKQIQRVETWALRHPGFTVIALFLAHAIGTSITQKGIIFILLMLVTPSMAMRCVGIGNRDFVEGLSGATWVDVVLEHGSCVTTMAKNKPTLDIELLKTEVTNPAVLRKLCIEAKISNTTTDSRCPTQGEATLVEEQDANFVCRRTFVDRGWGNGCGLFGKGSLLTCAKFKCVTKLEGKIVQYENLKYSVIVTVHTGDQHQVGNETTEHGTIATITPQAPTSEIQLTDYGALTLDCSPRTGLDFNEMVLLTMKEKSWLVHKQWFLDLPLPWTSGASTSQETWNRQDLLVTFKTAHAKKQEVVVLGSQEGAMHTALTGATEIQTSGTTTIFAGHLKCRLKMDKLTLKGTSYVMCTGSFKLEKEVAETQHGTVLVQVKYEGTDAPCKIPFSTQDEKGVTQNGRLITANPIVTDKEKPVNIETEPPFGESYIVVGAGEKALKLSWFKKGSSIGKMFEATARGARRMAILGDTAWDFGSIGGVFTSVGKLVHQVFGTAYGVLFSGVSWTMKIGIGILLTWLGLNSRSTSLSMTCIAVGMVTLYLGVMVQADSGCVINWKGRELKCGSGIFVTNEVHTWTEQYKFQADSPKRLSAAIGKAWEEGVCGIRSATRLENIMWKQISNELNHILLENDMKFTVVVGDANGILAQGKKMIRPQPMEHKYSWKSWGKAKIIGADIQNTTFIIDGPDTPECPDDQRAWNIWEVEDYGFGIFTTNIWLKLRDSYTQMCDHRLMSAAIKDSKAVHADMGYWIESEKNETWKLARASFIEVKTCTWPKSHTLWSNGVLESEMIIPKIYGGPISQHNYRPGYFTQTAGPWHLGKLELDFDLCEGTTVVVDEHCGNRGPSLRTTTVTGKIIHEWCCRSCTLPPLRFRGEDGCWYGMEIRPVKEKEENLVRSMVSAGSGEVDSFSLGILCVSIMIEEVMRSRWSRKMLMTGTLAVFLLLIMGQLTWNDLIRLCIMVGANASDRMGMGTTYLALMATFKMRPMFAVGLLFRRLTSREVLLLTIGLSLVASVELPNSLEELGDGLAMGIMMLKLLTEFQPHQLWTTLLSLTFIKTTLSLDYAWKTTAMALSIVSLFPLCLSTTSQKTTWLPVLLGSFGCKPLTMFLITENKIWGRKSWPINEGIMAIGIVSILLSSLLKNDVPLAGPLIAGGMLIACYVISGSSADLSLEKAAEVSWEQEAEHSGTSHNILVEVQDDGTMRIKDEEKDDTLTILLKATLLAVSGVYPMSIPATLFVWYFWQKKKQRSGVLWDTPSPPEVERAVLDDGIYRILQRGLLGRSQVGVGVFQDGVFHTMWHVTRGAVLMYQGKRLEPSWASVKKDLISYGGGWRFQGSWNTGEEVQVIAVEPGKNPKNVQTTPGTFKTPEGEVGAIALDFKPGTSGSPIVNREGKIVGLYGNGVVTTSGTYVSAIAQAKASQEGPLPEIEDEVFKKRNLTIMDLHPGSGKTRRYLPAIVREAIKRKLRTLILAPTRVVASEMAEALKGMPIRYQTTAVKSEHTGREIVDLMCHATFTMRLLSPVRVPNYNMIIMDEAHFTDPASIAARGYISTRVGMGEAAAIFMTATPPGSVEAFPQSNAVIQDEERDIPERSWNSGYDWITDFPGKTVWFVPSIKSGNDIANCLRKNGKRVIQLSRKTFDTEYQKTKNNDWDYVVTTDISEMGANFRADRVIDPRRCLKPVILKDGPERVILAGPMPVTVASAAQRRGRIGRNQNKEGDQYVYMGQPLNNDEDHAHWTEAKMLLDNINTPEGIIPALFEPEREKSAAIDGEYRLRGEARKTFVELMRRGDLPVWLSYKVASEGFQYSDRRWCFDGERNNQVLEENMDVEIWTKEGERKKLRPRWLDARTYSDPLALREFKEFAAGRRSVSGDLILEIGKLPQHLTLRAQNALDNLVMLHNSEQGGKAYRHAMEELPDTIETLMLLALIAVLTGGVTLFFLSGKGLGKTSIGLLCVTASSVLLWMASVEPHWIAASIILEFFLMVLLIPEPDRQRTPQDNQLAYVVIGLLFMILTVAANEMGLLETTKRDLGIGHVAAENHQHATMLDVDLRPASAWTLYAVATTVITPMMRHTIENTTANISLTAIANQAAILMGLDKGWPISKMDIGVPLLALGCYSQVNPLTLTAAVLMLVAHYAIIGPGLQAKATREAQKRTAAGIMKNPTVDGIVAIDLDPVVYDAKFEKQLGQIMLLILCASQVLLMRTTWALCESITLATGPLTTLWEGSPGKFWNTTIAVSMANIFRGSYLAGAGLAFSLMKSLGGGRRGTGAQGETLGEKWKRQLNQLSKSEFNTYKRSGIMEVDRSEAKEGLKRGETTKHAVSRGTAKLRWFVERNLVKPEGKVIDLGCGRGGWSYYCAGLKKVTEVKGYTKGGPGHEEPIPMATYGWNLVKLHSGKDVFFMPPEKCDTLLCDIGESSPNPTIEEGRTLRVLKMVEPWLRGNQFCIKILNPYMPSVVETLEQMQRKHGGMLVRNPLSRNSTHEMYWVSCGTGNIVSAVNMTSRMLLNRFTMAHRKPTYERDVDLGAGTRHVAVEPEVANLDIIGQRIENIKNEHKSTWHYDEDNPYKTWAYHGSYEVKPSGSASSMVNGVVRLLTKPWDVIPMVTQIAMTDTTPFGQQRVFKEKVDTRTPRAKRGTTQIMEVTAKWLWGFLSRNKKPRICTREEFTRKVRSNAAVGAVFVDENQWNSAKEAVEDERFWDLVHRERELHKQGKCATCVYNMMGKREKKLGEFGKAKGSRAIWYMWLGARFLEFEALGFMNEDHWFSRENSLSGVEGEGLHKLGYILRDISKIPGGNMYADDTAGWDTRITEDDLQNEAKITDIMEPEHALLATSIFKLTYQNKVVRVQRPAKNGTVMDVISRRDQRGSGQVGTYGLNTFTNMEVQLIRQMESEGIFLPSELETPNLAERVLDWLEKHGAERLKRMAISGDDCVVKPIDDRFATALTALNDMGKVRKDIPQWEPSKGWNDWQQVPFCSHHFHQLIMKDGREIVVPCRNQDELVGRARVSQGAGWSLRETACLGKSYAQMWQLMYFHRRDLRLAANAICSAVPVDWVPTSRTTWSIHAHHQWMTTEDMLSVWNRVWIEENPWMEDKTHVSSWEEVPYLGKREDQWCGSLIGLTARATWATNIQVAINQVRRLIGNENYLDYMTSMKRFKNESDPEGALW

>DENV1_AHI43752_Mexico

MNNQRKKTGRPSFNMLKRARNRVSTGSQLAKRFSKGLLSGQGPMKLVMAFIAFLRFLAIPPTAGILARWSSFKKNGAIKVLRGFKKEISSMLNIMNRRKRSVTMLLMLLPTALAFHLTTRGGEPHMIVSKQERGKSLLFKTSAGVNMCTLIAMDLGELCEDTMTYKCPQITEAEPDDVDCWCNATDTWVTYGTCSQTGEHRRDKRSVALAPHVGLGLETRTETWMSSEGAWKQIQRVETWALRHPGFTVIALFLAHAIGTSITQKGIIFILLMLVTPSMAMRCVGIGNRDFVEGLSGATWVDVVLEHGSCVTTMAKNKPTLDIELLKTEVTNPAILRKLCIEAKISNTTTDSRCPTQGEATLVEEQDANFVCRRTFVDRGWGNGCGLFGKGSLLTCAKFKCVTKLEGKIVQYENLKYSVIVTVHTGDQHQVGNETTEHGTIATITPQAPTSEIQLTDYGALTLDCSPRTGLDFNEMVLLTMKEKSWLVHKQWFLDLPLPWTSGASTSQETWNRQDLLVTFKTAHAKKQEVVVLGSQEGAMHTALTGATEIQTSGTTTIFAGHLKCRLKMDKLTLKGTSYVMCTGSFKLEKEVAETQHGTVLVQVKYEGTDAPCKIPFSTQDEKGVTQNGRLITANPIVTDKEKPVNIETEPPFGESYIVVGAGEKALKLSWFKKGSSIGKMFEATARGARRMAILGDTAWDFGSIGGVFTSVGKLVHQVFGTAYGVLFSGVSWTMKIGIGILLTWLGLNSRSTSLSMTCIAVGMVTLYLGVMVQADSGCVINWKGRELKCGSGIFVTNEVHTWTEQYKFQADSPKRLSAAIGKAWEEGVCGIRSATRLENIMWKQISNELNHILLENDMKFTVVVGDANGILAQGKKMIRPQPMEHKYSWKSWGKAKIIGADIQNTTFIIDGPDTPECPDNQRAWNIWEVEDYGFGIFTTNIWLKLRDSYTQMCDHRLMSAAIKDSKAVHADMGYWIESEKNETWKLARASFIEVKTCTWPKSHTLWSNGVLESEMIIPKIYGGPISQHNYRPGYFTQTAGPWHLGKLELDFDLCEGTTVVVDEHCGNRGPSLRTTTVTGKIIHEWCCRSCTLPPLRFRGEDGCWYGMEIRPVKEKEENLVRSMVSAGSGEVDSFSLGILCVSIMIEEVMRSRWSRKMLMTGTLAVFLLLVMGQLTWNDLIRLCIMVGANASDRMGMGTTYLALMATFKMRPMFAVGLLFRRLTSREVLLLTIGLSLVASVELPNSLEELGDGLAMGIMMLKLLTEFQPHQLWTTLLSLTFIKTTLSLDYAWKTTAMALSIVSLFPLCLSTTSQKTTWLPVLLGSFGCKPLTMFLITENKIWGRKSWPLNEGIMAIGIVSILLSSLLKNDVPLAGPLIAGGMLIACYVISGSSADLSLEKAAEVSWEQEAEHSGTSHNILVEVQDDGTMKIKDEERDDTLTILLKATLLAVSGVYPMSIPATLFVWYFWQKKKQRSGVLWDTPSPPEVERAVLDDGIYRILQRGLLGRSQVGVGVFQDGVFHTMWHVTRGAVLMYQGKRLEPSWASVKKDLISYGGGWRFQGSWNTGEEVQVIAVEPGKNPKNVQTTPGTFKTPEGEVGAIALDFKPGTSGSPIVNREGKIVGLYGNGVVTTSGTYVSAIAQAKASQEGPLPEIEDEVFKKRNLTIMDLHPGSGKTRRYLPAIVREAIKRKLRTLILAPTRVVASEMAEALKGMPIRYQTTAVKSEHTGREIVDLMCHATFTMRLLSPVRVPNYNMIIMDEAHFTDPASIAARGYISTRVGMGEAAAIFMTATPPGSVEAFPQSNAVIQDEERDIPERSWNSGYDWITDFPGKTVWFVPSIKSGNDIANCLRKNGKRVIQLSRKTFDTEYQKTKNNDWDYVVTTDISEMGANFRADRVIDPRRCLKPVILKDGPERVILAGPMPVTVASAAQRRGRIGRNQNKEGDQYVYMGQPLNNDEDHAHWTEAKMLLDNINTPEGIIPALFEPEREKNAAIDGEYRLRGEARKTFVELMRRGDLPVWLSYKVASEGFQYSDRRWCFDGERNNQVLEENMDVEIWTKEGERKKLRPRWLDARTYSDPLALREFKEFAAGRRSVSGDLILEIGKLPQHLTLRAQNALDNLVMLHNSEQGGKAYRHAMEELPDTIETLMLLALIAVLTGGVTLFFLSGKGLGKTSIGLLCVTASSALLWMASVEPHWIAASIILEFFLMVLLIPEPDRQRTPQDNQLAYVVIGLLFMILTVAANEMGLLETTKKDLGIGHVAAENHQHATMLDVDLRPASAWTLYAVATTVITPMMRHTIENTTANISLTAIANQAAILMGLDKGWPISKMDIGVPLLALGCYSQVNPLTLTAAVLMLVAHYAIIGPGLQAKATREAQKRTAAGIMKNPTVDGIVAIDLDPVVYDAKFEKQLGQIMLLILCTSQILLMRTTWALCESITLATGPLTTLWEGSPGKFWNTTIAVSMANIFRGSYLAGAGLAFSLMKSLGGGRRGTGAQGETLGEKWKRQLNQLSKSEFNTYKRSGIMEVDRSEAKEGLKRGETTKHAVSRGTAKLRWFVERNLVKPEGKVIDLGCGRGGWSYYCAGLKKVTEVKGYTKGGPGHEEPIPMATYGWNLVKLHSGKDVFFMPPEKCDTLLCDIGESSPNPTIEEGRTLRVLKMVEPWLRGNQFCIKILNPYMPSVVETLEQMQRKHGGMLVRNPLSRNSTHEMYWVSCGTGNIVSAVNMTSRMLLNRFTMAHRKPTYERDVDLGAGTRHVAVEPEVANLDIIGQRIENIKNEHKSTWHYDEDNPYKTWAYHGSYEVKPSGSASSMVNGVVRLLTKPWDVIPMVTQIAMTDTTPFGQQRVFKEKVDTRTPRAKRGTTQIMEVTAKWLWGFLSRNKKPRICTREEFTRKVRSNAAIGAVFVDENQWNSAKEAVEDERFWDLVHRERELHKQGKCATCVYNMMGKREKKLGEFGKAKGSRAIWYMWLGARFLEFEALGFMNEDHWFSRENSLSGVEGEGLHKLGYILRDISKIPGGNMYADDTAGWDTRITEDDLQNEAKITEIMEPEHALLATSIFKLTYQNKVVRVQRPAKNGTVMDVISRRDQRGSGQVGTYGLNTFTNMEVQLIRQMESEGIFLPSELETPNLAERVLDWLEKHGAERLKRMAISGDDCVVKPIDDRFATALTALNDMGKVRKDIPQWEPSKGWNDWQQVPFCSHHFHQLIMKDGREIVVPCRNQDELVGRARVSQGAGWSLRETACLGKSYAQMWQLMYFHRRDLRLAANAICSAVPVDWVPTSRTTWSIHAHHQWMTTEDMLSVWNRVWIEENPWMEDKTHVSSWEEVPYLGKREDQWCGSLIGLTARATWATNIQVAINQVRRLIGNENYLDYMTSMKRFKNESDPEGALW

>DENV1_AHI43751_Mexico

MNNQRKKTGRPSFNMLKRARNRVSTGSQLAKRFSKGLLSGQGPMKLVMAFIAFLRFLAIPPTAGILARWSSFKKNGAIKVLRGFKKEISSMLNIMNRRKRSVTMLLMLLPTALAFHLTTRGGEPHMIVSKQERGKSLLFKTSAGVNMCTLIAMDLGELCEDTMTYKCPQITEAEPDDVDCWCNATDTWVTYGTCSQNGEHRRDKRSVALAPHVGLGLETRTETWMSSEGAWKQIQRVETWALRHPGFTVIALFLAHAIGTSITQKGIIFILLMLVTPSMAMRCVGIGNRDFVEGLSGATWVDVVLEHGSCVTTMAKNKPTLDIELLKTEVTNPAILRKLCIEAKISNTTTDSRCPTQGEATLVEEQDANFVCRRTFVDRGWGNGCGLFGKGSLLTCAKFKCVTKLEGKIVQYENLKYSVIVTVHTGDQHQVGNETTEHGTIATITPQAPTSEIQLTDYGALTLDCSPRTGLDFNEMVLLTMKEKSWLVHKQWFLDLPLPWTSGASTSQETWNRQDLLVTFKTAHAKKQEVVVLGSQEGAMHTALTGATEIQTSGTTTIFAGHLKCRLKMDKLTLKGTSYVMCTGPFKLEKEVAETQHGTVLVQVKYEGTDAPCKIPFSTQDEKGVTQNGRLITANPIVTDKEKPVNIETEPPFGESYIVVGAGEKALKLSWFKKGSSIGKMFEATARGARRMAILGDTAWDFGSIGGVFTSVGKLVHQVFGTAYGVLFSGVSWTMKIGIGILLTWLGLNSRSTSLSMTCIAVGMVTLYLGVMVQADSGCVINWKGRELKCGSGIFVTNEVHTWTEQYKFQADSPKRLSAAIGKAWEEGVCGIRSATRLENIMWKQISNELNHILLENDMKFTVVVGDANGILAQGKKMIRPQPMEHKYSWKSWGKAKIIGADIQNTTFIIDGPDTPECPDNQRAWNIWEVEDYGFGIFTTNIWLKLRDSYTQMCDHRLMSAAIKDSKAVHADMGYWIESEKNETWKLARASFIEVKTCTWPKSHTLWSNGVLESEMIIPKIYGGPISQHNYRPGYFTQTAGPWHLGKLELDFDLCEGTTVVVDEHCGNRGPSLRTTTVTGKIIHEWCCRSCTLPPLRFRGEDGCWYGMEIRPVKEKEENLVRSMVSAGSGEVDSFSLGILCVSIMIEEVMRSRWSRKMLMTGTLAVFLLLVMGQLTWNDLIRLCIMVGANASDRMGMGTTYLALMATFKMRPMFAVGLLFRRLTSREVLLLTIGLSLVASVELPNSLEELGDGLAMGIMMLKLLTEFQPHQLWTTLLSLTFIKTTLSLDYAWKTTAMALSIVSLFPLCLSTTSQKTTWLPVLLGSFGCKPLTMFLITENKIWGRKSWPLNEGIMAIGIVSILLSSLLKNDVPLAGPLIAGGMLIACYVISGSSADLSLEKAAEVSWEQEAEHSGTSHNILVEVQDDGTMKIKDEERDDTLTILLKATLLAVSGVYPMSIPATLFVWYFWQKKKQRSGVLWDTPSPPEVERAVLDDGIYRILQRGLLGRSQVGVGVFQDGVFHTMWHVTRGAVLMYQGKRLEPSWASVKKDLISYGGGWRFQGSWNTGEEVQVIAVEPGKNPKNVQTTPGTFKTPEGEVGAIALDFKPGTSGSPIVNREGKIVGLYGNGVVTTSGTYVSAIAQAKASQEGPLPEIEDEVFKKRNLTIMDLHPGSGKTRRYLPAIVREAIKRKLRTLILAPTRVVASEMAEALKGMPIRYQTTAVKSEHTGREIVDLMCHATFTMRLLSPVRVPNYNMIIMDEAHFTDPASIAARGYISTRVGMGEAAAIFMTATPPGSVEAFPQSNAVIQDEERDIPERSWNSGYDWITDFPGKTVWFVPSIKSGNDIANCLRKNGKRVIQLSRKTFDTEYQKTKNNDWDYVVTTDISEMGANFRADRVIDPRRCLKPVILKDGPERVILAGPMPVTVASAAQRRGRIGRNQNKEGDQYVYMGQPLNNDEDHAHWTEAKMLLDNINTPEGIIPALFEPEREKSAAIDGEYRLRGEARKTFVELMRRGDLPVWLSYKVASEGFQYSDRRWCFDGERNNQVLEENMDVEIWTKEGERKKLRPRWLDARTYSDPLALREFKEFAAGRRSISGDLILEIGKLPQHLTLRAQNALDNLVMLHNSEQGGKAYRHAMEELPDTIETLMLLALIAVLTGGVTLFFLSGKGLGKTSIGLLCVTASSALLWMASVEPHWIAASIILEFFLMVLLIPEPDRQRTPQDNQLAYVVIGLLFMILTVAANEMGLLETTKKDLGIGHVAAENHQHATMLDVDLRPASAWTLYAVATTVITPMMRHTIENTTANISLTAIANQAAILMGLDKGWPISKMDIGVPLLALGCYSQVNPLTLTAAVLMLVAHYAIIGPGLQAKATREAQKRTAAGIMKNPTVDGIVAIDLDPVVYDAKFEKQLGQIMLLILCTSQILLMRTTWALCESITLATGPLTTLWEGSPGKFWNTTIAVSMANIFRGSYLAGAGLAFSLMKSLGGGRRGTGAQGETLGEKWKRQLNQLSKSEFNTYKRSGIMEVDRSEAKEGLKRGETTKHAVSRGTAKLRWFVERNLVKPEGKVIDLGCGRGGWSYYCAGLKKVTEVKGYTKGGPGHEEPIPMATYGWNLVKLHSGKDVFFMPPEKCDTLLCDIGESSPNPTIEEGRTLRVLKMVEPWLRGNQFCIKILNPYMPSVVETLEQMQRKHGGMLVRNPLSRNSTHEMYWVSCGTGNIVSAVNMTSRMLLNRFTMAHRKPTYERDVDLGAGTRHVAVEPEVANLDIIGQRIENIKNEHKSTWHYDEDNPYKTWAYHGSYEVKPSGSASSMVNGVVRLLTKPWDVIPMVTQIAMTDTTPFGQQRVFKEKVDTRTPRAKRGTTQIMEVTAKWLWGFLSRNKKPRICTREEFTRKVRSNAAIGAVFVDENQWNSAKEAVEDERFWDLVHRERELHKQGKCATCVYNMMGKREKKLGEFGKAKGSRAIWYMWLGARFLEFEALGFMNEDHWFSRENSLSGVEGEGLHKLGYILRDISKIPGGNMYADDTAGWDTRITEDDLQNEAKITEFMEPEHALLATSIFKLTYQNKVVRVQRPAKNGTVMDVISRRDQRGSGQVGTYGLNTFTNMEVQLIRQMESEGIFLPSELETPNLAERVFDWLEKHGAERLKRMAISGDDCVVKPIDDRFATALTALNDMGKVRKDIPQWEPSKGWNDWQQVPFCSHHFHQLIMKDGREIVVPCRNQDELVGRARVSQGAGWSLRETACLGKSYAQMWQLMYFHRRDLRLAANAICSAVPVDWVPTSRTTWSIHAHHQWMTTEDMLSVWNRVWIEENPWMEDKTHVSSWEEVPYLGKREDQWCGSLIGLTARATWATNIQVAINQVRRLIGNENYLDYMTSMKRFKNESDPEGALW

>DENV1_AHG23208_Mexico

MNNQRKKTGQPSFNMLKRARNRVSTGSQLAKRFSKGLLSGQGPMKLVMAFIAFLRFLAIPPTAGILARWSSFKKNGAIKVLRGFKKEISSMLNIMNRRKRSVTMLLMLLPTALAFHLTTRGGEPHMIVSKQERGKSLLFKTSAGVNMCTLIAMDLGELCEDTMTYKCPQITEAEPDDVDCWCNATDTWVTYGTCSQTGEHRRDKRSVALAPHVGLGLETRTETWMSSEGAWKQIQRVETWALRHPGFTVIALFLAHAIGTSITQKGIIFILLMLVTPSMAMRCVGIGNRDFVEGLSGATWVDVVLEHGSCVTTMAKNKPTLDIELLKTEVTNPAILRKLCIEAKISNTTTDSRCPTQGEATLVEEQDANFVCRRTFVDRGWGNGCGLFGKGSLLTCAKFKCVTKLEGKIVQYENLKYSVIVTVHTGDQHQVGNETTEHGTIATITPQAPTSEIQLTDYGALTLDCSPRTGLDFNEMVLLTMKEKSWLVHKQWFLDLPLPWTSGASTSQETWNRQDLLVTFKTAHAKKQEVVVLGSQEGAMHTALTGATEIQTSGTTTIFAGHLKCRLKMDKLTLKGTSYVMCTGSFKLEKEVAETQHGTVLVQVKYEGTDAPCKIPFSTQDEKGVTQNGRLITANPIVTDKEKPVNIETEPPFGESYIVVGAGEKALKLSWFKKGSSIGKMFEATARGARRMAILGDTAWDFGSIGGVFTSVGKLVHQVFGTAYGVLFSGVSWTMKIGIGILLTWLGLNSRSTSLSMTCIAVGMVTLYLGVMVQADSGCVINWKGRELKCGSGIFVTNEVHTWTEQYKFQADSPKRLSAAIGKAWEEGVCGIRSATRLENIMWKQISNELNHILLENDMKFTVVVGDANGILAQGKKMIRPQPMEHKYSWKSWGKAKIIGADIQNTTFIIDGPDTPECPDNQRAWNIWEVEDYGFGIFTTNIWLKLRDSYTQMCDHRLMSAAIKDSKAVHADMGYWIESEKNETWKLARASFIEVKTCTWPKSHTLWSNGVLESEMIIPKIYGGPISQHNYRPGYFTQTAGPWHLGKLELDFDLCDGTTVVVDEHCGNRGPSLRTTTVTGKIIHEWCCRSCTLPPLRFRGEDGCWYGMEIRPVKEKEENLVRSMVSAGSGEVDSFSLGILCASIMIEEVMRSRWSRKMLMTGTLAVFLLLIMGQLTWNDLIRLCIMVGANASDRMGMGTTYLALMATFKMRPMFAVGLLFRRLTSREVLLLTIGLSLVASVELPNSLEELGDGLAMGIMMLKLLTEFQPHQLWTTLLSLTFIKTTLSLDYAWKTTAMALSIVSLFPLCLSTTSQKTTWLPVLLGSFGCKPLTMFLITENKIWGRKSWPLNEGIMAIGIVSILLSSLLKNDVPLAGPLIAGGMLIACYVISGSSADLSLEKAAEVSWEQEAEHSGTSHNILVEVQDDGTMKIKDEERDDTLTILLKATLLAVSGVYPMSIPATLFVWYFWQKKKQRSGVLWDTPSPPEVERAVLDDGIYRILQRGLLGRSQVGVGVFQDGVFHTMWHVTRGAVLMYQGKRLEPSWASVKKDLISYGGGWRFQGSWNTGEEVQVIAVEPGKNPKNVQTTPGTFKTPEGEVGAIALDFKPGTSGSPIVNREGKIVGLYGNGVVTTSGTYVSAIAQAKASQEGPLPEIEDEVFKKRNLTIMDLHPGSGKTRRYLPAIVREAIKRKLRTLILAPTRVVASEMAEALKGMPIRYQTTAVKSEHTGREIVDLMCHATFTMRLLSPVRVPNYNMIIMDEAHFTDPASIAARGYISTRVGMGEAAAIFMTATPPGSVEAFPQSNAVIQDEERDIPERSWNSGYDWITDFPGKTVWFVPSIKSGNDIANCLRKNGKRVIQLSRKTFDTEYQKTKNNDWDYVVTTDISEMGANFRADRVIDPRRCLKPVILKDGPERVILAGPMPVTVASAAQRRGRIGRNQNKEGDQYVYMGQPLNNDEDHAHWTEAKMLLDNINTPEGIIPALFEPEREKSAAIDGEYRLRGEARKTFVELMRRGDLPVWLSYKVASEGFQYSDRRWCFDGERNNQVLEENMDVEIWTKEGERKKLRPRWLDARTYSDPLALREFKEFAAGRRSVSGDLILEIGKLPQHLTLRAQNALDNLVMLHNSEQGGKAYRHAMEELPDTIETLMLLALIAVLTGGVTLFFLSGKGLGKTSIGLLCVTASSALLWMASVEPHWIAASIILEFFLMVLLIPEPDRQRTPQDNQLAYVVIGLLFMILTVAANEMGLLETTKKDLGIGHVAAENHQHATMLDVDLRPASAWTLYAVATTVITPMMRHTIENTTANISLTAIANQAAILMGLDKGWPISKMDIGVPLLALGCYSQVNPLTLTAAVLMLVAHYAIIGPGLQAKATREAQKRTAAGIMKNPTVDGIVAIDLDPVVYDAKFEKQLGQIMLLILCTSQILLMRTTWALCESITLATGPLTTLWEGSPGKFWNTTIAVSMANIFRGSYLAGAGLAFSLMKSLGGGRRGTGAQGETLGEKWKRQLNQLSKSEFNTYKRSGIMEVDRSEAKEGLKRGETTKHAVSRGTAKLRWFVERNLVKPEGKVIDLGCGRGGWSYYCAGLKKVTEVKGYTKGGPGHEEPIPMATYGWNLVKLHSGKDVFFMPPEKCDTLLCDIGESSPNPTIEEGRTLRVLKMVEPWLRGNQFCIKILNPYMPSVVETLEQMQRKHGGMLVRNPLSRNSTHEMYWVSCGTGNIVSAVNMTSRMLLNRFTMAHRKPTFERDVDLGAGTRHVAVEPEVANLDIIGQRIENIKNEHKSTWHYDEDNPYKTWAYHGSYEVKPSGSASSMVNGVVRLLTKPWDVIPMVTQIAMTDTTPFGQQRVFKEKVDTRTPRAKRGTTQIMEVTAKWLWGFLSRNKKPRICTREEFTRKVRSNAAIGAVFVDENQWNSAKEAVEDERFWDLVHRERELHKQGKCATCVYNMMGKREKKLGEFGKAKGSRAIWYMWLGARFLEFEALGFMNEDHWFSRENSLSGVEGEGLHKLGYILRDISKIPGGNMYADDTAGWDTRITEDDLQNEAKITDIMEPEHALLATSIFKLTYQNKVVRVQRPAKNGTVMDVISRRDQRGSGQVGTYGLNTFTNMEVQLIRQMESEGIFLPSELETPNLAERVLDWLEKHGAERLKRMAISGDDCVVKPIDDRFATALTALNDMGKVRKDIPQWEPSKGWNDWQQVPFCSHHFHQLIMKDGREIVVPCRNQDELVGRARVSQGAGWSLRETACLGKSYAQMWQLMYFHRRDLRLAANAICSAVPVDWVPTSRTTWSIHAHHQWMTTEDMLSVWNRVWIEENPWMEDKTHVSSWEEVPYLGKREDQWCGSLIGLTARATWATNIQVAINQVRRLIGNENYLDYMTSMKRFKNESDPEGALW

>DENV1__AHG23209_Mexico

MNNQRKKTGQPSFNMLKRARNRVSTGSQLAKRFSKGLLSGQGPMKLVMAFIAFLRFLAIPPTAGILARWSSFKKNGAIKVLRGFKKEISSMLNIMNRRKRSVTMLLMLLPTALAFHLTTRGGEPHMIVSKQERGKSLLFKTSAGVNMCTLIAMDLGELCEDTMTYKCPQITEAEPDDVDCWCNATDTWVTYGTCSQTGEHRRDKRSVALAPHVGLGLETRTETWMSSEGAWKQIQRVETWALRHPGFTVIALFLAHAIGTSITQKGIIFILLMLVTPSMAMRCVGIGNRDFVEGLSGATWVDVVLEHGSCVTTMAKNKPTLDIELLKTEVTNPAILRKLCIEAKISNTTTDSRCPTQGEATLVEEQDANFVCRRTFVDRGWGNGCGLFGKGSLLTCAKFKCVTKLEGKIVQYENLKYSVIVTVHTGDQHQVGNETTEHGTIATITPQAPTSEIQLTDYGALTLDCSPRTGLDFNEMVLLTMKEKSWLVHKQWFLDLPLPWTSGASTSQETWNRQDLLVTFKTAHAKKQEVVVLGSQEGAMHTALTGATEIQMSGTTTIFAGHLKCRLKMDKLTLKGTSYVMCTGSFKLEKEVAETQHGTVLVQVKYEGTDAPCKIPFSTQDEKGVTQNGRLITANPIVTDKEKPVNIETEPPFGESYIVVGAGEKALKLSWFKKGSSIGKMFEATARGARRMAILGDTAWDFGSIGGVFTSVGKLVHQVFGTAYGVLFSGVSWTMKIGIGILLTWLGLNSRSTSLSMTCIAVGMVTLYLGVMVQADSGCVINWKGRELKCGSGIFVTNEVHTWTEQYKFQADSPKRLSAAIGKAWEEGVCGIRSVTRLENIMWKQISNELNHILLENDMKFTVVVGDANGILAQGKKMIRPQPMEHKYSWKSWGKAKIIGADIQNTTFIIDGPDTPECPDNQRAWNIWEVEDYGFGIFTTNIWLKLRDSYTQMCDHRLMSAAIKDSKAVHADMGYWIESEKNETWKLARASFIEVKTCTWPKSHTLWSNGVLESEMIIPKIYGGPISQHNYRPGYFTQTAGPWHLGKLELDFDLCEGTTVVVDEHCGNRGPSLRTTTVTGKIIHEWCCRSCTLPPLRFRGEDGCWYGMEIRPVKEKEENLVRSMVSAGSGEVDSFSLGILCASIMIEEVMRSRWSRKMLMTGTLAVFLLLIMGQLTWNDLIRLCIMVGANASDRMGMGTTYLALMATFKMRPMFAVGLLFRRLTSREVLLLTIGLSLVASVELPNSLEELGDGLAMGIMMLKLLTEFQPHQLWTTLLSLTFIKTTLSLDYAWKTTAMALSIVSLFPLCLSTTSQKTTWLPVLLGSFGCKPLTMFLITENKIWGRKSWPLNEGIMAIGIVSILLSSLLKNDVPLAGPLIAGGMLIACYVISGSSADLSLEKAAEVSWEQEAEHSGTSHNILVEVQDDGTMKIKDEERDDTLTILLKATLLAVSGVYPMSIPATLFLWYFWQKKKQRSGVLWDTPSPPEVERAVLDDGIYRILQRGLLGRSQVGVGVFQDGVFHTMWHVTRGAVLMYQGKRLEPSWASVKKDLISYGGGWRFQGSWNTGEEVQVIAVEPGKNPKNVQTTPGTFKTPEGEVGAIALDFKPGTSGSPIVNREGKIVGLYGNGVVTTSGTYVSAIAQAKASQEGPLPEIEDEVFKKRNLTIMDLHPGSGKTRRYLPAIVREAIKRKLRTLILAPTRVVASEMAEALKGMPIRYQTTAVKSEHTGREIVDLMCHATFTMRLLSPVRVPNYNMIIMDEAHFTDPASIAARGYISTRVGMGEAAAIFMTATPPGSVEAFPQSNAVIQDEERDIPERSWNSGYDWITDFPGKTVWFVPSIKSGNDIANCLRKNGKRVIQLSRKTFDTEYQKTKNNDWDYVVTTDISEMGANFRADRVIDPRRCLKPVILKDGPERVILAGPMPVTVASAAQRRGRIGRNQNKEGDQYVYMGQPLNNDEDHAHWTEAKMLLDNINTPEGIIPALFEPEREKSAAIDGEYRLRGEARKTFVELMRRGDLPVWLSYKVASEGFQYSDRRWCFDGERNNQVLEENMDVEIWTKEGERKKLRPRWLDARTYSDPLALREFKEFAAGRRSVSGDLILEIGKLPQHLTLRAQNALDNLVMLHNSEQGGKAYRHAMEELPDTIETLMLLALIAVLTGGVTLFFLSGKGLGKTSIGLLCVTASSALLWMASVEPHWIAASIILEFFLMVLLIPEPDRQRTPQDNQLAYVVIGLLFMILTVAANEMGLLETTKKDLGIGHVAAENHQHATMLDVDLRPASAWTLYAVATTVITPMMRHTIENTTANISLTAIANQAAILMGLDKGWPISKMDIGVPLLALGCYSQVNPLTLTAAVLMLVAHYAIIGPGLQAKATREAQKRTAAGIMKNPTVDGIVAIDLDPVVYDAKFEKQLGQIMLLILCTSQILLMRTTWALCESITLATGPLTTLWEGSPGKFWNTTIAVSMANIFRGSYLAGAGLAFSLMKSLGGGRRGTGAQGETLGEKWKRQLNQLSKSEFNTYKRSGIMEVDRSEAKEGLKRGETTKHAVSRGTAKLRWFVERNLVKPEGKVIDLGCGRGGWSYYCAGLKKVTEVKGYTKGGPGHEEPIPMATYGWTLVKLHSGKDVFFMPPEKCDTLLCDIGESSPNPTIEEGRTLRVLKMVEPWLRGNQFCIKILNPYMPSVVETLEQMQRKHGGMLVRNPLSRNSTHEMYWVSCGTGNIVSAVNMTSRMLLNRFTMAHRKPTFERDVDLGAGTRHVAVEPEVANLDIIGQRIENIKNEHKSTWHYDEDNPYKTWAYHGSYEVKPSGSASSMVNGVVRLLTKPWDVIPMVTQIAMTDTTPFGQQRVFKEKVDTRTPRAKRGTTQIMEVTAKWLWGFLSRNKKPRICTREEFTRKVRSNAAIGAVFVDENQWNSAKEAVEDERFWDLVHRERELHKQGKCATCVYNMMGKREKKLGEFGKAKGSRAIWYMWLGARFLEFEALGFMNEDHWFSRENSLSGVEGEGLHKLGYILRDISKIPGGNMYADDTAGWDTRITEDDLQNEAKITDIMEPEHALLATSIFKLTYQNKVVRVQRPAKNGTVMDVISRRDQRGSGQVGTYGLNTFTNMEVQLIRQMESEGIFLPSELETPNLAERVLDWLEKHGAERLKRMAISGDDCVVKPIDDRFATALTALNDMGKVRKDIPQWEPSKGWNDWQQVPFCSHHFHQLIMKDGREIVVPCRNQDELVGRARVSQGAGWSLRETACLGKSYAQMWQLMYFHRRDLRLAANAICSAVPVDWVPTSRTTWSIHAHHQWMTTEDMLSVWNRVWIEENPWMEDKTHVSSWEEVPYLGKREDQWCGSLIGLTARATWATNIQVAINQVRRLIGNENYLDYMTSMKRFKNESDPEGALW

>DENV1_AHG23193_Mexico

MNNQRKKTGRPSFNMLKRARNRVSTGSQLAKRFSKGLLSGQGPMKLVMAFIAFLRFLAIPPTAGILARWSSFKKNGAIKVLRGFKKEISSMLNIMNRRKRSVTMLLMLLPTALAFHLTTRGGEPHMIVSKQERGKSLLFKTSAGVNMCTLIAMDLGELCEDTMTYKCPQITEAEPDDVDCWCNATDTWVTYGTCSQTGEHRRDKRSVALAPHVGLGLETRTETWMSSEGAWKQIQRVETWALRHPGFTVIALFLAHAIGTSITQKGIIFILLMLVTPSMAMRCVGIGNRDFVEGLSGATWVDVVLEHGSCVTTMAKNKPTLDIELLKTEVTNPAILRKLCIEAKISNTTTDSRCPTQGEATLVEEQDANFVCRRTFVDRGWGNGCGLFGKGSLLTCAKFKCVTKLEGKIVQYENLKYSVIVTVHTGDQHQVGNETTEHGTIATITPQAPTSEIQLTDYGALTLDCSPRTGLDFNEMVLLTMKEKSWLVHKQWFLDLPLPWTSGASTSQETWNRQDLLVTFKTAHAKKQEVVVLGSQEGAMHTAWTGATEIQTSGTTTIFAGHLKWRLKMDKLTLKGTSYVMCTGSFKLEKEVAETQHGTGLVQVKYEGTDAPCKIPFSAQDEKGVTQNGRLITANPIVTDKEKPVNIETEPPFGESYIVVGAGEKALKLSWFKKGSSIGKMFEATARGARRMAILGDTAWDFGSIGGVFTSVGKLVHQVFGTAYGVLFSGVSWTMKIGIGILLTWLGLNSRSTSLSMTCIAVGMVTLYLGVMVQADSGCVINWKGRELKCGSGIFVTNEVHTWTEQYKFQADSPKRLSAAIGKAWEEGVCGIRSATRLENIMWKQISNELNHILLENDMKFTVVVGDANGILAQGKKMIRPQPMEHKYSWKSWGKAKIIGADIQNTTFIIDGPDTPECPDNQRAWNIWEVEDYGFGIFTTNIWLKLRDSYTQMCDHRLMSAAIKDSKAVHADMGYWIESEKNETWKLARASFIEVKTCTWPKSHTLWSNGVLESEMIIPKIYGGPISQHNYRPGYFTQTAGPWHLGKLELDFDLCEGTTVVVDEHCGNRGPSLRTTTVTGKIIHEWCCRSCTLPPLRFRGEDGCWYGMEIRPVKEKEENLVRSMVSAGSGEVDSFSLGILCASIMIEEVMRSRWSRKMLMTGTLAVFLLLVMGQLTWNDLIRLCIMVGANASDRMGMGTTYLALMATFKMRPMFAVGLLFRRLTSREVLLLTIGLSLVASVELPNSLEELGDGLAMGIMMLKLLTEFQPHQLWTTLLSLTFIKTTLSLDYAWKTTAMALSIVSLFPLCLSTTSQKTTWLPVLLGSFGCKPLTMFLITENKIWGRKSWPLNEGIMAIGIVSILLSSLLKNDVPLPDPLIAGGILIACYVISGSSADLSLEKAAEMSWEQEAEHSGTSHNILVEVQDDGPMKIKDEERDDTLTILLKATLLAVSGVYPMSIPGTLFVWYFWQKKKQRSGVLWDTPSPPKVERAVLDDGIYRILQRGLLGRSQVGVGVFQDGVFHTMWHVTRGAVLMYQGKRLEPSWASVKKDLISYGGGWRFQGSWNTGEEVQVIAVEPGKNPKNVQTTPGTFKTPEGEVGAIALDFKPGTSGSPIVNREGKIVGLYGNGVVTTSGTYVSAIAQAKASQEGPLPEIEDEVFKKRNLTIMDLHPGSGKTRRYLPAIVREAIKRKLRTLILAPTRVVASEMAEALKGMPIRYQTTAVKSEHTGREIVDLMCHATFTMRLLSPVRVPNYNMIIMDEAHFTDPASIAARGYISTRVGMGEAAAIFMTATPPGSVEAFPQSNAVIQDEERDIPERSWNSGYDWITDFPGKTVWFVPSIKSGNDIANCLRKNGKRVIQLSRKTFDTEYQKTKNNDWDYVVTTDISEMGANFRADRVIDPRRCLKPVILKDGPERVILAGPMPVTVASAAQRRGRIGRNQNKEGDQYVYMGQPLNNDEDHAHWTEAKMLLDNINTPEGIIPALFEPEREKSAAIDGEYRLRGEARKTFVELMRRGDLPVWLSYKVASEGFQYSDRRWCFDGERNNQVLEENMDVEIWTKEGERKKLRPRWLDARTYSDPLALREFKEFAAGRRSISGDLILEIGKLPQHLTLRAQNALDNLVMLHNSEQGGKAYRHAMEELPDTIETLMLLALIAVLTGGVTLFFLSGKGLGKTSIGLLCVTASSALLWMASVEPHWIAASIILEFFLMVLLIPEPDRQRTPQDNQLAYVVIGLLFMILTVAANEMGLLETTKKDLGIGHVAAENHQHATMLDVDLRPASAWTLYAVATTVITPMMRHTIENTTANISLTAIANQAAILMGLDKGWPISKMDIGVPLLALGCYSQVNPLTLTAAVLMLVAHYAIIGPGLQAKATREAQKRTAAGIMKNPTVDGIVAIDLDPVVYDAKFEKQLGQIMLLILCTSQILLMRTTWALCESITLATGPLTTLWEGSPGKFWNTTIAVSMANIFRGSYLAGAGLAFSLMKSLGGGRRGTGAQGETLGEKWKRQLNQLSKSEFNTYKRSGIMEVDRSEAKEGLKRGETTKHAVSRGTAKLRWFVERNLVKPEGKVIDLGCGRGGWSYYCAGLKKVTEVKGYTKGGPGHEEPIPMATYGWNLVKLHSGKDVFFMPPEKCDTLLCDIGESSPNPTIEEGRTLRVLKMVEPWLRGNQFCIKILNPYMPSVVETLEQMQRKHGGMLVRNPLSRNSTHEMYWVSCGTGNIVSAVNMTSRMLLNRFTMAHRKPTYERDVDLGAGTRHVAVEPEVANLDIIGQRIENIKNEHKSTWHYDEDNPYKTWAYHGSYEVKPSGSASSMVNGVVRLLTKPWDVIPMVTQIAMTDTTPFGQQRVFKEKVDTRTPRAKRGTTQIMEVTAKWLWGFLSRNKKPRICTREEFTRKVRSNAAIGAVFVDENQWNSAKEAVEDERFWDLVHRERELHKQGKCATCVYNMMGKREKKLGEFGKAKGSRAIWYMWLGARFLEFEALGFMNEDHWFSRENSLSGVEGEGLHKLGYILRDISKIPGGNMYADDTAGWDTRITEDDLQNEAKITEIMEPEHALLATSIFKLTYQNKVVRVQRPAKNGTVMDVISRRDQRGSGQVGTYGLNTFTNMEVQLIRQMESEGIFLPSELETPNLAERVLDWLEKHGAERLKRMAISGDDCVVKPIDDRFATALTALNDMGKVRKDIPQWEPSKGWNDWQQVPFCSHHFHQLIMKDGREIVVPCRNQDELVGRARVSQGAGWSLRETACLGKSYAQMWQLMYFHRRDLRLAANAICSAVPVDWVPTSRTTWSIHAHHQWMTTEDMLSVWNRVWIEENPWMEDKTHVSSWEEVPYLGKREDQWCGSLIGLTARATWATNIQVAINQVRRLIGNENYLDYMTSMKRFKNESDPEGALW

>DENV1_AHI43732_Mexico

MNNQRKKTGRPSFNMLKRARNRVSTGSQLAKRFSKGLLSGQGPMKLVMAFIAFLRFLAIPPTAGILARWSSFKKNGAIKVLRGFKREISSMLNIMNRRKRSVTMLLMLLPTALAFHLTTRGGEPHMIVSKHERGKSLLFKTSAGVNMCTLIAMDLGELCEDTMTYKCPRITEAEPDDVDCWCNATDTWVTYGTCSQTGEHRRDKRSVALAPHVGLGLETRTETWMSSEGAWKQIQRVETWALRHPGFTVIALFLAHAIGTSITQKGIIFILLMLVTPSMAMRCVGIGNRDFVEGLSGATWVDVVLEHGSCVTTMAKNKPTLDIELLKTEVTNPAVLRKLCIEAKISNTTTDSRCPTQGEATLVEEQDANFVCRRTFVDRGWGNGCGLFGKGSLLTCAKFKCVTKLEGKIVQYENLKYSVIVTVHTGDQHQVGNETTEHGTIATITPQAPTSEIQLTDYGALTLDCSPRTGLDFNEMVLLTMKEKSWLVHKQWFLDLPLPWTSGASTSQETWNRQDLLVTFKTAHAKKQEVVVLGSQEGAMHTALTGATEIQTSGTTTIFAGHLKCRLKMDKLTLKGTSYVMCTGSFKLEKEVAETQHGTVLVQVKYEGTDAPCKIPFSTQDEKGVTQNGRLITANPIVTDKEKPVNIETEPPFGESYIVVGAGEKALKLSWFKKGSSIGKMFEATARGARRMAILGDTAWDFGSIGGVFTSVGKLVHQVFGTAYGVLFSGVSWTMKIGIGILLTWLGLNSRSTSFSMTCIAVGMVTLYLGVMVQADSGCVINWKGRELKCGSGIFVTNEVHTWTEQYKFQADSPKRLSAAIGKAWEEGVCGIRSATRLENIMWKQISNELNHILLENDMKFTVVVGDANGILAQGKKMIRPQPMEHKYSWKSWGKAKIIGADIQNTTFIIDGPDTPECPDDQRAWNIWEVEDYGFGIFTTNIWLKLRDSYTQMCDHRLMSAAIKDSKAVHADMGYWIESEKNETWKLARASFIEVKTCTWPKSHTLWSNGVLESEMIIPKIYGGPISQHNYRPGYFTQTAGPWHLGKLELDFDLCEGTTVVVDEHCGNRGPSLRTTTVTGKIIHEWCCRSCTLPPLRFRGEDGCWYGMEIRPVKEKEENLVRSMVSAGSGEVDSFSLGILCVSIMIEEVMRSRWSRKMLMTGTLAVFLLLIMGQLTWNDLIRLCIMVGANASDRMGMGTTYLALMATFKMRPMFAVGILFRRLTSREVLLLTIGLSLVASVELPNSLEELGDGLAMGIMMLKLLTEFQPHQLWTTLLSLTFIKTTLSLDYAWKTTAMALSIVSLFPLCLSTTSQKTTWLPVLLGSFGCKPLTMFLITENKIWGRKSWPINEGIMAIGIVSILLSSLLKNDVPLAGPLIAGGMLIACYVISGSSADLSLEKAAEVSWEQEAEHSGTSHNILVEVQDDGTMRIKDEEKDDTLTILLKATLLAVSGVYPMSIPATLFVWYFWQKKKQRSGVLWDTPSPPEVERAVLDDGIYRILQRGLLGRSQVGVGVFQDGVFHTMWHVTRGAVLMYQGKRLEPSWASVKKDLISYGGGWRFQGSWNTGEEVQVIAVEPGKNPKNVQTTPGTFKTPEGEVGAIALDFKPGTSGSPIVNREGKIVGLYGNGVVTTSGTYVSAIAQAKASQEGPLPEIEDEVFKKRNLTIMDLHPGSGKTRRYLPAIVREAIKRKLRTLILAPTRVVASEMAEALKGMPIRYQTTAVKSEHTGREIVDLMCHATFTMRLLSPVRVPNYNMIIMDEAHFTDPASIAARGYISTRVGMGEAAAIFMTATPPGSVEAFPQSNAVIQDEERDIPERSWNSGYDWITDFPGKTVWFVPSIKSGNDIANCLRKNGKRVIQLSRKTFDTEYQKTKNNDWDYVVTTDISEMGANFRADRVIDPRRCLKPVILKDGPERVILAGPMPVTVASAAQRRGRIGRNQNKEGDQYVYMGQPLNNDEDHAHWTEAKMLLDNINTPEGIIPALFEPEREKSAAIDGEYRLRGEARKTFVELMRRGDLPVWLSYKVASEGFQYSDRRWCFDGERNNQVLEENMDVEIWTKEGERKKLRPRWLDARTYSDPLALREFKEFAAGRRSVSGDLILEIGKLPQHLTLRAQNALDNLVMLHNSEQGGKAYRHAMEELPDTIETLMLLALIAVLTGGVTLFFLSGKGLGKTSIGLLCVTASSALLWMASVEPHWIAASIILEFFLMVLLIPEPDRQRTPQDNQLAYVVIGLLFMILTVAANEMGLLETTKRDLGIGHVAAENHQHATMLDVDLRPASAWTLYAVATTVITPMMRHTIENTTANISLTAIANQAAILMGLDKGWPISKMDIGVPLLALGCYSQVNPLTLTAAVLMLVAHYAIIGPGLQAKATREAQKRTAAGIMKNPTVDGIVAIDLDPVVYDAKFEKQLGQIMLLILCTSQILLMRTTWALCESITLATGPLTTLWEGSPGKFWNTTIAVSMANIFRGSYLAGAGLAFSLMKSLGGGRRGTGAQGETLGEKWKRQLNQLSKPEFNTYKRSGIMEVDRSEAKEGLKRGETTKHAVSRGTAKLRWFVERNLVKPEGKVIDLGCGRGGWSYYCAGLKKVTEVKGYTKGGPGHEEPIPMATYGWNLVKLHSGKDVFFMPPEKCDTLLCDIGESSPNPTIEEGRTLRVLKMVEPWLRGNQFCIKILNPYMPSVVETLEQMQRKHGGMLVRNPLSRNSTHEMYWVSCGTGNIVSAVNMTSRMLLNRFTMAHRKPTYERDVDLGAGTRHVAVEPEVANLDIIGQRIENIKNEHKSTWHYDEDNPYKTWAYHGSYEVKPSGSASSMVNGVVRLLTKPWDVIPMVTQIAMTDTTPFGQQRVFKEKVDTRTPRAKRGTTQIMEVTAKWLWGFLSRNKKPRICTREEFTRKVRSNAAIGAVFVDENQWNSAKEAVEDERFWDLVHRERELHKQGKCATCVYNMMGKREKKLGEFGKAKGSRAIWYMWLGARFLEFEALGFMNEDHWFSRENSLSGVEGEGLHKLGYILRDISKIPGGNMYADDTAGWDTRITEDDLQNEAKITDIMEPEHALLATSIFKLTYQNKVVRVQRPAKNGTVMDVISRRDQRGSGQVGTYGLNTFTNMEVQLIRQMESEGIFLPSELETPNLAERVLNWLEKHGAERLKRMAISGDDCVVKPIDDRFATALTALNDMGKVRKDIPQWEPSKGWNDWQQVPFCSHHFHQLIMKDGREIVVPCRNQDELVGRARVSQGAGWSLRETACLGKSYAQMWQLMYFHRRDLRLAANAICSAVPVDWVPTSRTTWSIHAHHQWMTTEDMLSVWNRVWIEENPWMEDKTHVSSWEEVPYLGKREDQWCGSLIGLTARATWATNIQVAINQVRRLIGNENYLDYMTSMKRFKNESDPEGALW

>DENV1__AHI43729_Mexico

MNNQRKKTGRPSFNMLKRARNRVSTGSQLAKRFSKGLLSGQGPMKLVMAFIAFLRFLAIPPTAGILARWSSFKKNGAIKVLRGFKKEISSMLNIMNRRKRSVTMLLMLLPTALAFHLTTRGGEPHMIVSKQERGKSLLFKTSAGVNMCTLIAMDLGELCEDTMTYKCPQITEAEPDDVDCWCNATDTWVTYGTCSQTGEHRRDKRSVALAPHVGLGLETRTETWMSSEGAWKQIQRVETWALRHPGFTVIALFLAHAIGTSITQKGIIFILLMLVTPSMAMRCVGIGNRDFVEGLSGATWVDVVLEHGSCVTTMAKNKPTLDIELLKTEVTNPAILRKLCIEAKISNTTTDSRCPTQGEATLVEEQDANFVCRRTFVDRGWGNGCGLFGKGSLLTCAKFKCVTKLEGKIVQYENLKYSVIVTVHTGDQHQVGNETTEHGTIATITPQAPTSEIQLTDYGALTLDCSPRTGLDFNEMVLLTMKEKSWLVHKQWFLDLPLPWTSGASTSQETWNRQDLLVTFKTAHAKKQEVVVLGSQEGAMHTALTGATEIQMSGTTTIFAGHLKCRLKMDKLTLKGTSYVMCTGSFKLEKEVAETQHGTVLVQVKYEGTDAPCKIPFSTQDEKGVTQNGRLITANPIVTDKEKPVNIETEPPFGESYIVVGAGEKALKLSWFKKGSSIGKMFEATARGARRMAILGDTAWDFGSIGGVFTSVGKLVHQVFGTAYGVLFSGVSWTMKIGIGILLTWLGLNSRSTSLSMTCIAVGMVTLYLGVMVQADSGCVINWKGRELKCGSGIFVTNEVHTWTEQYKFQADSPKRLSAAIGKAWEEGVCGIRSATRLENIMWKQISNELNHILLENDMKFTVVVGDANGILAQGKKMIRPQPMEHKYSWKSWGKAKIIGADIQNTTFIIDGPDTPECPDNQRAWNIWEVEDYGFGIFTTNIWLKLRDSYTQMCDHRLMSAAIKDSKAVHADMGYWIESEKNETWKLARASFIEVKTCTWPKSHTLWSNGVMESEMIIPKIYGGPISQHNYRPGYFTQTAGPWHLGKLELDFDLCEGTTVVVDEHCGNRGPSLRTTTVTGKIIHEWCCRSCTLPPLRFRGEDGCWYGMEIRPVKEKEENLVRSMVSAGSGEVDSFSLGILCASIMIEEVMRSRWSRKMLMTGTLAVFLLLVMGQLTWNDLIRLCIMVGANASDRMGMGTTYLALMATFKMRPMFAVGLLFRRLTSREVLLLTIGLSLVASVELPNSLEELGDGLAMGIMMLKLLTEFQPHQLWTTLLSLTFIKTTLSLDYAWKTTAMALSIVSLFPLCLSTTSQKTTWLPVLLGSFGCKPLTMFLITENKIWGRKSWPLNEGIMAIGIVSILLSSLLKNDVPLAGPLIAGGMLIACYVISGSSADLSLEKAAEVSWEQEAEHSGTSHNILVEVQDDGTMKIKDEERDDTLTILLKATLLAVSGVYPMSIPATLFLWYFWQKKKQRSGVLWDTPSPPEVERAVLDDGIYRILQRGLLGRSQVGVGVFQDGVFHTMWHVTRGAVLMYQGKRLEPSWASVKKDLISYGGGWRFQGSWNTGEEVQVIAVEPGKNPKNVQTTPGTFKTPEGEVGAIALDFKPGTSGSPIVNREGKIVGLYGNGVVTTSGTYVSAIAQAKASQEGPLPEIEDEVFKKRNLTIMDLHPGSGKTRRYLPAIVREAIKRKLRTLILAPTRVVASEMAEALKGMPIRYQTTAVKSEHTGREIVDLMCHATFTMRLLSPVRVPNYNMIIMDEAHFTDPASIAARGYISTRVGMGEAAAIFMTATPPGSVEAFPQSNAVIQDEERDIPERSWNSGYDWITDFPGKTVWFVPSIKSGNDIANCLRKNGKRVIQLSRKTFDTEYQKTKNNDWDYVVTTDISEMGANFRADRVIDPRRCLKPVILKDGPERVILAGPMPVTVASAAQRRGRIGRNQNKEGDQYVYMGQPLNNDEDHAHWTEAKMLLDNINTPEGIIPALFEPEREKSAAIDGEYRLRGEARKTFVELMRRGDLPVWLSYKVASEGFQYSDRRWCFDGERNNQVLEENMDVEIWTKEGERKKLRPRWLDARTYSDPLALREFKEFAAGRRSVSGDLILEIGKLPQHLTSRAQNALDNLVMLHNSEQGGKAYRHAMEELPDTIETLMLLALIAVLTGGVTLFFLSGKGLGKTSIGLLCVTASSALLWMASVEPHWIAASIILEFFLMVLLIPEPDRQRTPQDNQLAYVVIGLLFMILTVAANEMGLLETTKKDLGIGHVAAENHQHATMLDVDLRPASAWTLYAVATTVITPMMRHTIENTTANISLTAIANQAAILMGLDKGWPISKMDIGVPLLALGCYSQVNPLTLTAAVLMLVAHYAIIGPGLQAKATREAQKRTAAGIMKNPTVDGIVAIDLDPVVYDAKFEKQLGQIMLLILCTSQILLMRTTWALCESITLATGPLTTLWEGSPGKFWNTTIAVSMANIFRGSYLAGAGLAFSLMKSLGGGRRGTGAQGETLGEKWKRQLNQLSKSEFNTYKRSGIMEVDRSEAKEGLKRGETTKHAVSRGTAKLRWFVERNLVKPEGKVIDLGCGRGGWSYYCAGLKKVTEVKGYTKGGPGHEEPIPMATYGWNLAKLHSGKDVFFMPPEKCDTLLCDIGESSPNPTIEEGRTLRVLKMVEPWLRGNQFCIKILNPYMPSVVETLEQMQRKHGGMLVRNPLSRNSTHEMYWVSCGTGNIVSAVNMTSRMLLNRFTTAHRKPTYERDVDLGAGTRHVAVEPEVANLDIIGQRIENIKNEHKSTWHYDEDNPYKTWAYHGSYEVKPSGSASSMVNGVVRLLTKPWDVIPMVTQIAMTDTTPFGQQRVFKEKVDTRTPRAKRGTTQIMEVTAKWLWGFLSRNKKPRICTREEFTRKVRSNAAIGAVFVDENQWNSAKEAVEDERFWDLVHRERELHKQGKCATCVYNMMGKREKKLGEFGKAKGSRAIWYMWLGARFLEFEALGFMNEDHWFSRENSLSGVEGEGLHKLGYILRDISKIPGGNMYADDTAGWDTRITEDDLQNEAKITEIMEPEHALLATSIFKLTYQNKVVRVQRPAKNGTVMDVISRRDQRGSGQVGTYGLNTFTNMEVQLIRQMESEGIFLPSELETPNLAERVLDWLEKHGAERLKRMAISGDDCVVKPIDDRFATALTALNDMGKVRKDILQWEPSKGWNDWQQVPFCSHHFHQLIMKDGREIVVPCRNQDELVGRARVSQGAGWSLRETACLGKSYAQMWQLMYFHRRDLRLAANAICSAVPVDWVPTSRTTWSIHAHHQWMTTEDMLSVWNRVWIEENPWMEDKTHVSSWEEVPYLGKREDQWCGSLIGLTARATWATNIQVAINQVRRLIGNENYLDYMTSMKRFKNESDPEGALW

>DENV1_AMN88557_Australia

MNNQRKKTARPSFNMLKRARNRVSTVSQLAKRFSKGLLSGQGPMKLVMAFIAFLRFLAIPPTAGILARWGSFKKNGAIKVLRGFKKEISNMLNIMNRRKRSVTMLLMLMPTALAFHLTTRGGEPHMIVSKQERGKSLLFKTSAGVNMCTLIAMDLGELCEDTLTYKCPRITEAEPDDVDCWCNATDTWVTYGTCSQTGEHRRDKRSVALAPHVGLGLETRTETWMSSEGAWKQIQRVETWALRHPGFTVIALFLAHAIGTSITQKGIIFILLMLVTPSMAMRCVGIGSRDFVEGLSGATWVDVVLEHGSCVTTMAKDKPTLDIELLKTEVTNPAVLRKLCIEAKISNTTTDSRCPTQGEATLVEEQDANFVCRRTFVDRGWGNGCGLFGKGSLITCAKFKCVTKLEGKIVQYENLKYSVIVTVHTGDQHQVGNESTEHGTTATITPQAPTTEIQLTDYGALTLDCSPRTGLNFNEMVLLTMKEKSWLVHKQWFLDLPLPWTSGASTTQETWNRQDLLVTFKTAHAKKQEVVVLGSQEGAMHTALTGATEIQTSGTTTIFAGHLKCRLKMDKLTLKGMSYVMCTGSFKLEKEVAETQHGTVLVQIKYEGTDAPCKIPFSTQDEKGVTQNGRLITANPIVTDKEKPVNIEAEPPFGESYIVIGAGEKALKLSWFKKGSSIGKMFEATARGARRMAILGDTAWDFGSIGGVFTSVGKLVHQIFGTAYGVLFSGVSWTMKIGIGVLLTWLGLNSRSTSLSMTCIAVGLVTLYLGVMVQADSGCVINWKGRELKCGSGIFVTNEVHTWTEQYKFQADSPRRLSAAIGKAWEEGVCGIRSATRLENIMWKQISNELNHILLENDMKFTVVVGDVAGILAQGKKMIRPQPMEHKYSWKSWGKAKIIGADVQNTTFIIDGPNTPECPDDQRAWNIWEVEDYGFGIFTTNIWLKLRDSYTQVCDHRLMSAAIKDSKAVHADMGYWIESEKNETWKLARASFIEVKTCIWPKSHTLWSNGVLESEMIIPKIYGGPISQHNYRPGYFTQTAGPWHLGKLELDFDLCEGTTVVVDEHCGNRGPSLRTTTVTGKIIHEWCCRSCTLPPLRFRGEDGCWYGMEIRPVKEKEENLVKSMVSAGSGEVDSFSLGLLCISIMIEEVMRSRWSRKMLITGTLAVFFLLIMGQVTWNDLIRLCIMVGANASDRMGMGTTYLALMATFKMRPMFAVGLLFRRLTSREVLLLTIGLSLVASVELPNSLEELGDGLAMGIMILKLLTDFQPHQLWATLLSLTFIKTTFSLHYAWKTMAMVLSIVSLFPLCLSTTSQKTTWLPVLLGSLGCKPLTMFLIAENKIWGRKSWPLNEGIMAVGIVSILLSSLLKNDVPLAGPLIAGGMLIACYVISGSSADLSLEKAAEVSWEEEAEHSGASHNILVEVQDDGTMKIKDEERDDTLTILLKATLLAVSGVYPLSIPATLFVWYFWQKKKQRSGVLWDTPSPPEVERAVLDDGIYRIMQRGLLGRSQVGVGVFQENVFHTMWHVTRGAVLMYQGKRLEPSWASVKKDLISYGGGWRLQGSWNTGEEVQVIAVEPGKNPKNVQTAPGTFKTPEGEVGAIALDFKPGTSGSPIVNREGKIVGLYGNGVVTTSGTYVSAIAQAKASQEGPLPEIEDEVFRKRNLTIMDLHPGSGKTRRYLPAIVREAIKRKLRTLILAPTRVVASEMAEALKGMPIRYQTTAVKSEHTGKEIVDLMCHATFTMRLLSPVRVPNYNMIIMDEAHFTDPSSIAARGYISTRVGMGEAAAIFMTATPPGSVEAFPQSNAVIQDEERDIPERSWNSGYEWITDFPGKTVWFVPSIKSGNDIANCLRKNGKRVIQLSRKTFDTEYQKTKNNDWDYVVTTDISEMGANFRADRVIDPRRCLKPVILKDGPERVILAGPMPVTVASAAQRRGRIGRNHNKEGDQYIYMGQPLNNDEDHAHWTEAKMLLDNINTPEGIIPALFEPEREKSAAIDGEYRLRGEARKTFVELMRRGDLPVWLSYKVASEGFQYSDRRWCFDGERNNQVLEENMDVEIWTKEGERKKLRPRWLDARTYSDPLALREFKEFAAGRRSVSGDLILEIGKLPQHLTQRAQNALDNLVMLHNSEQGGRAYRHAMEELPDTIETLMLLALIAVLTGGVTLFFLSGRGLGKTSIGLLCVMASSVLLWMASVEPHWIAASIILEFFLMVLLIPEPDRQRTPQDNQLAYVVIGLLFMILTVAANEMGLLETTKKDLGIGHVAVENHHHAAMLDVDLHPASAWTLYAVATTIITPMMRHTIENTTANISLTAIANQAAILMGLDKGWPISKMDIGVPLLALGCYSQVNPLTLTAAVLMLVAHYAIIGPGLQAKATREAQKRTAAGIMKNPTVDGIVAIDLDPVVYDAKFEKQLGQIMLLILCTSQILLMRTTWALCESITLATGPLTTLWEGSPGKFWNTTIAVSMANIFRGSYLAGAGLAFSLMKSLGGGRRGTGAQGETLGEKWKRQLNQLSKSEFNTYKRSGIMEVDRSEAKEGLKRGETTKHAVSRGTAKLRWFVERNLVKPEGKVIDLGCGRGGWSYYCAGLKKVTEVKGYTKGGPGHEEPIPMATYGWNLVKLHSGKDVFFIPPEKCDTLLCDIGESSPNPTIEEGRTLRVLKMVEPWLRGNQFCIKILNPYMPSVVETLEQMQRKHGGMLVRNPLSRNSTHEMYWVSCGTGNIVSAVNMTSRMLLNRFTMAHRKPTYERDVDLGAGTRHVAVEPEVANLDIIGQRIENIKHEHKSTWHYDEDNPYKTWAYHGSYEVKPSGSASSMVNGVVKLLTKPWDVIPMVTQIAMTDTTPFGQQRVFKEKVDTRTPKAKRGTAQIMEVTARWLWGFLSRNKKPRICTREEFTRKVRSNAAIGAVFVDENQWNSAKEAVEDERFWDLVHRERELHKQGKCATCVYNMMGKREKKLGEFGKAKGSRAIWYMWLGARFLEFEALGFMNEDHWFSRENSLSGVEGEGLHKLGYILRDISKIPGGNMYADDTAGWDTRITEDDLQNEAKITDIMEPEHALLAKAIFKLTYQNKVVRVQRPAKNGTVMDVISRRDQRGSGQVGTYGLNTFTNMEAQLIRQMESEGIFSPSELETPNLAERVLYWLEKYGVERLKRMAISGDDCVVKPIDDRFATALTALNDMGKVRKDIPQWEPSKGWNDWQQVPFCSHHFHQLIMKDGREIVVPCRNQDELVGRARVSQGAGWSLRETACLGKSYAQMWQLMYFHRRDLRLAANAICSAVPVDWVPTSRTTWSIHAHHQWMTTEDMLSVWNRVWIEENPWMEDKTHISSWEDVPYLGKREDQWCGSLIGLTARATWATNIQVAINQVRRLIGNENYLDYMTSMKRFKNESDPEGALW

>DENV1_AIE17470_Sri_Lanka

MNNQRKKTARPSFNMLKRARNRVSTVSQLAKRFSKGLLSGQGPMKLVMAFIAFLRFLAIPPTAGILARWGSFKKNGAIKVLRGFKKEISNMLNIMNRRRKSVTMLLMLMPTALAFHLTTRGGEPHMIVSKQERGKSLLFKTSAGVNMCTLIAMDLGELCEDTMTYKCPRITEAEPDDVDCWCNATDTWVTYGTCSQTGEHRRDKRSVALAPHVGLGLETRTETWMSSEGAWKQIQRVETWALRHPGFTVIALFLAHAIGTSITQKGIIFILLMLVTPSMAMRCVGIGSRDFVEGLSGATWVDVVLEHGSCVTTMAKDKPTLDIELLKTEVTNPAVLRKLCIEAKISNTTTDSRCPTQGEATLVEEQDANFVCRRTFVDRGWGNGCGLFGKGSLLTCAKFKCVTKLEGKIVQYENLKYSVIVTVHTGDQHQVGNESTEHGTTATITPQAPTTEIQLTDYGALTLDCSPRTGLDFNEMVLLTMKEKSWLVHKQWFLDLPLPWTSGASTSQETWNRQDLLVTFKTAHAKKQEVVVLGSQEGAMHTALTGATEIQMSGTTTIFAGHLKCRLKMDKLTLKGMSYVMCTGSFKLEKEVAETQHGTVLVQIKYEGTDAPCKIPFSTQDEKGVTQNGRLITANPIVTDKEKPVNIEAEPPFGESYIVIGAGEKALKLSWFKKGSSIGKMFEATARGARRMAILGDTAWDFGSIGGVFTSVGKLVHQIFGTAYGVLFSGVSWTMKIGIGVLLTWLGLNSRSTSLSMTCIVVGLVTLYLGVMVQADSGCVINWKGRELKCGSGIFVTNEVHTWTEQYKFQADSPKRLSAAIGKAWEEGVCGIRSATRLENIMWKQISNELNHILLENDMKFTVVVGDVAGILAQGKKMIRPQPMEHKYSWKNWGKAKIIGADVQNSTFIIDGPNTPECSDDQRAWNIWEVEDYGFGIFTTNIWLKLRDSYTQVCDHRLMSAAIKDSKAVHADMGYWIESEKNDTWKLARASFIEVKTCIWPKSHTLWSNGVLESEMIIPKIYGGPISQHNYRPGYFTQTAGPWHLGKLELDFDLCEGTTVVVDEHCGNRGPSLRTTTVTGKIIHEWCCRSCTLPPLRFRGEDGCWYGMEIRPVKEKEENLVKSMVSAGSGEVDSFSLGLLCISIMIEEVMRSRWSRKMLMTGTLAVFFLLIMGQLTWNDLIRLCIMVGANASDRMGMGTTYLALMATFKMRPMFAVGLLFRRLTSREVLLLTIGLSLVASVELPNSLEELGDGLAMGIMILKLLTDFQSHQLWATLLSMTFIKTTFSLHYAWKTMAMVLSIVSLFPLCLSTTSQKTTWLPVLLGSLGCKPLTMFLIAENKIWGRKSWPLNEGIMAVGIVSILLSSLLKNDVPLAGPLIAGGMLIACYVISGSSADLSLEKAAEVSWEEEAEHSGASHNILVEVQDDGTMKIKDEERDDTLTILLKATLLAVSGVYPLSIPATLFVWYFWQKKKQRSGVLWDTPSPPKVEKAVLDDGIYRIMQRGLLGRSQVGVGVFQENVFHTMWHVTRGAVLMYQGKRLEPSWASVKKDLISYGGGWRLQGSWNTGEEVQVIAVEPGKNPKNVQTAPGTFKTPEGEVGAIALDFKPGTSGSPIVNREGKIVGLYGNGVVTTSGTYVSAIAQAKASQEGPLPEIEDEVFRKRNLTIMDLHPGSGKTRRYLPAIVREAIKRKMRTLILAPTRVVASEMAEALKGMPIRYQTTAVKSEHTGKEIVDLMCHATFTMRLLSPVRVPNYNMIIMDEAHFTDPSSIAARGYISTRVGMGEAAAIFMTATPPGSVEAFPQSNAVIQDEERDIPERSWNSGYEWITDFPGKTVWFVPSIKAGNDIANCLRKNGKRVIQLSRKTFDTEYQKTKNNDWDYVVTTDISEMGANFRADRVIDPRRCLKPVILKDGPERVILAGPMPVTVASAAQRRGRIGRNHNKEGDQYIYMGQPLNNDEDHAHWTEAKMLLDNINTPEGIIPALFEPEREKSAAIDGEYRLRGEARKTFVELMRRGDLPVWLSYKVASEGFQYSDRRWCFDGERNNQVLEENMDVEIWTKEGERKKLRPRWLDARTYSDPLALREFKEFAAGRRSVSGDLILEIGKLPQHLTQRAQNALDNLVMLHNSEQGGRAYRHAMEELPDTIETLMLLALIAVLTGGVTLFFLSGKGLGKTSIGLLCVMASSTLLWMASVEPHWIAASIILEFFLMVLLIPEPDRQRTPQDNQLAYVVIGLLFMILTVAANEMGLLETTKKDLGIGHVAVENHHHAAMLDIDLHPASAWTLYAVATTIITPMMRHTIENTTANISLTAIANQAAILMGLDKGWPISKMDIGVPLLALGCYSQVNPLTLTAAVLMLVAHYAIIGPGLQAKATREAQKRTAAGIMKNPTIDGIVAIDLDPVVYDAKFEKQLGQIMLLILCTSQILLMRTTWALCESITLATGPLTTLWEGSPGKFWNTTIAVSMANIFRGSYLAGAGLAFSLMKSLGGGRRGTGAQGETLGEKWKRQLNQLSKSEFNTYKRSGIMEVDRSEAKEGLKRGETTKHAVSRGTAKLRWFVERNLVKPEGKVIDLGCGRGGWSYYCAGLKKVTEVKGYTKGGPGHEEPIPMATYGWNLVKLHSGKDVFFIPPEKCDTLLCDIGESSPNPTIEEGRTLRVLKMVEPWLRGNQFCIKILNPYMPSVVETLEQMQRKHGGMLVRNPLSRNSTHEMYWVSCGTGNIVSAVNMTSRMLLNRFTMAHRKPTYERDVDLGAGTRHVAVEPEVANLDIIGQRIENIKHEHKSTWHYDEDNPYKTWAYHGSYEVKPSGSASSMVNGVVKLLTKPWDVIPMVTQIAMTDTTPFGQQRVFKEKVDTRTPKAKRGTAQIMEVTARWLWSFLSRNKKPRICTKEEFTRKVRSNAAIGAVFVDENQWNSAKEAVEDDRFWDLVHRERELHKQGKCATCVYNMMGKREKKLGEFGKAKGSRAIWYMWLGARFLEFEALGFMNEDHWFSRENSLSGVEGEGLHKLGYILRDISKIPGGNMYADDTAGWDTRITEDDLQNEAKITDIMEPEHALLAKSIFKLTYQNKVVRVQRPAKNGTVMDVISRRDQRGSGQVGTYGLNTFTNMEAQLIRQMESEEIFSPNELETPNLAERVLDWLEKYGVERLKRMAISGDDCVVKPIDDRFATALTALNDMGKVRKDIPQWEPSKGWNDWQQVPFCSHHFHQLIMKDGREIVVPCRNQDELVGRARVSQGAGWSLRETACLGKSYAQMWQLMYFHRRDLRLAANAICSAVPVDWVPTSRTTWSIHAHHQWMTTEDMLSVWNRVWIEENPWMEDKTRVSSWEDVPYLGKREDQWCGSLIGLTARATWATNIQVAINQVRRLIGNENYSDYMTSMKRFKNESEPEGALW

>DENV1_AKC32653_Sri_Lanka

MNNQRKKTARPSFNMLKRARNRVSTVSQLAKRFSKGLLSGQGPMKLVMAFIAFLRFLAIPPTAGILARWGSFKKNGAIKVLRGFKKEISNMLNIMNRRRKSVTMLLMLMPTALAFHLTTRGGEPHMIVSKQERGKSLLFKTSAGVNMCTLIAMDLGELCEDTMTYKCPRITEAEPDDVDCWCNATDTWVTYGTCSQTGEHRRDKRSVALAPHVGLGLETRTETWMSSEGAWKQIQRVETWALRHPGFTVIALFLAHAIGTSITQKGIIFILLMLVTPSMAMRCVGIGSRDFVEGLSGATWVDVVLEHGSCVTTMAKDKPTLDIELLKTEVTSPAVLRKLCIEAKISNTTTDSRCPTQGEATLVEEQDANFVCRRTFVDRGWGNGCGLFGKGSLLTCAKFKCVTKLEGKIVQYENLKYSVIVTVHTGDQHQVGNESTEHGTTATITPQAPTTEIQLTDYGALTLDCSPRTGLDFNEMVLLTMKEKSWLVHKQWFLDLPLPWTSGASTSQETWNRQDLLVTFKTAHAKKQEVVVLGSQEGAMHTALTGATEIQMSGTTTIFAGHLKCRLKMDKLTLKGMSYVMCTGSFKLEKEVAETQHGTVLVQIKYEGTDAPCKIPFSTQDEKGVTQNGRLITANPIVTDKEKPVNIEAEPPFGESYIVIGAGEKALKLSWFKKGSSIGKMFEATARGARRMAILGDTAWDFGSIGGVFTSVGKLVHQIFGTAYGVLFSGVSWTMKIGIGVLLTWLGLNSRSTSLSMTCIAVGLVTLYLGVMVQADSGCVINWKGRELKCGSGIFVTNEVHTWTEQYKFQADSPKRLSAAIGKAWEEGVCGIRSATRLENIMWKQISNELNHILLENDMKFTVVVGDVAGILAQGKKMIRPQPMEHKYSWKNWGKAKIIGADVQNSTFIIDGPNTPECSDDQRAWNIWEVEDYGFGIFTTNIWLKLRDSYTQVCDHRLMSAAIKDSKAVHADMGYWIESEKNDTWKLARASFIEVKTCIWPKSHTLWSNGVLESEMIIPKIYGGPISQHNYRPGYFTQTAGPWHLGKLELDFDLCEGTTVVVDEHCGNRGPSLRTTTVTGKIIHEWCCRSCTLPPLRFRGEDGCWYGMEIRPVKEKEENLVKSMVSAGPGEVDSFSLGLLCISIMIEEVMRSRWSRKMLMTGTLAVFFLLIMGQLTWNDLIRLCIMVGANASDRMGMGTTYLALMATFKMRPMFAVGLLFRRLTSREVLLLTIGLSLVASVELPNSLEELGDGLAMGIMILKLLTDFQSHQLWATLLSMTFIKTTLSLHYAWKTMAMVLSIVSLFPLCLSTTSQKTTWLPVLLGSLGCKPLTMFLIAENKIWGRKSWPLNEGIMAVGIVSILLSSLLKNDVPLAGPLIAGGMLIACYVISGSSADLSLEKAAEVSWEEEAEHSGASHNILVEVQDDGTMKIKDEERDDTLTILLKATLLAVSGVYPLSIPATLFVWYFWQKKKQRSGVLWDTPSPPKVEKAVLDDGIYRIMQRGLLGRSQVGVGVFQENVFHTMWHVTRGAVLMYQGKRLEPSWASVKKDLISYGGGWRLQGSWNTGEEVQVIAVEPGKNPKNVQTAPGTFKTPEGEVGAIALDFKPGTSGSPIVNREGKIVGLYGNGVVTTSGTYVSAIAQAKASQEGPLPEIEDEVFRKRNLTIMDLHPGSGKTRRYLPAIVREAIKRKMRTLILAPTRVVASEMAEALKGMPIRYQTTAVKSEHTGKEIVDLMCHATFTMRLLSPVRVPNYNMIIMDEAHFTDPSSIAARGYISTRVGMGEAAAIFMTATPPGSVEAFPQSNAVIQDEERDIPERSWNSGYEWITDFPGKTVWFVPSIKAGNDIANCLRKNGKRVIQLSRKTFDTEYQKTKNNDWDYVVTTDISEMGANFRADRVIDPRRCLKPVILKDGPERVILAGPMSVTVASAAQRRGRIGRNHNKEGDQYIYMGQPLNNDEDHAHWTEAKMLLDNINTPEGIIPALFEPEREKSAAIDGEYRLRGEARKTFVELMRRGDLPVWLSYKVASEGFQYSDRRWCFDGERNNQVLEENMDVEIWTKEGERKKLRPRWLDARTYSDPLALREFKEFAAGRRSVSGDLILEIGKLPQHLTQRAQNALDNLVMLHNSEQGGRAYRHAMEELPDTIETLMLLALIAVLTGGVTLFFLSGKGLGKTSIGLLCVMASSALLWMASVEPHWIAASIILEFFLMVLLIPEPDRQRTPQDNQLAYVVIGLLFMILTVAANEMGLLETTKKDLGIGHVAVENHHHAAMLDIDLHPASAWTLYAVATTIITPMMRHTIENTTANISLTAIANQAAILMGLDKGWPISKMDIGVPLLALGCYSQVNPLTLTAAVLMLVAHYAIIGPGLQAKATREAQKRTAAGIMKNPTIDGIVAIDLDPVVYDAKFEKQLGQIMLLILCTSQILLMRTTWALCESITLATGPLTTLWEGSPGKFWNTTIAVSMANIFRGSYLAGAGLAFSLMKSLGGGRRGTGAQGETLGEKWKRQLNQLSKSEFNTYKRSGIMEVDRSEAKEGLKRGETTKHAVSRGTAKLRWFVERNLVKPEGKVIDLGCGRGGWSYYCAGLKKVTEVKGYTKGGPGHEEPIPMATYGWNLVKLHSGKDVFFTPPEKCDTLLCDIGESSPNPTIEEGRTLRVLKMVEPWLRGNQFCIKILNPYMPSVVETLEQMQRKHGGMLVRNPLSRNSTHEMYWVSCGTGNIVSAVNMTSRMLLNRFTMAHRKPTYERDVDLGAGTRHVAVEPEVANLDIIGQRIENIKHEHKSTWHYDEDNPYKTWAYHGSYEVKPSGSASSMVNGVVKLLTKPWDVIPMVTQIAMTDTTPFGQQRVFKEKVDTRTPKAKRGTAQIMEVTARWLWSFLSRNKKPRICTREEFTRKVRSNAAIGAVFVDENQWNSAKEAVEDDRFWDLVHRERELHKQGKCATCVYNMMGKREKKLGEFGKAKGSRAIWYMWLGARFLEFEALGFMNEDHWFSRENSLSGVEGEGLHKLGYILRDISKIPGGNMYADDTAGWDTRITEDDLQNEAKITDIMEPEHALLAKSIFKLTYQNKVVRVQRPAKNGTVMDVISRRDQRGSGQVGTYGLNTFTNMEAQLIRQMESEEIFSPNELETPNLAERVLDWLEKYGVERLKRMAISGDDCVVKPIDDRFATALTALNDMGKVRKDIPQWEPSKGWNDWQQVPFCSHHFHQLIMKDGREIVVPCRNQDELVGRARVSQGAGWSLRETACLGKSYAQMWQLMYFHRRDLRLAANAICSAVPVDWVPTSRTTWSIHAHHQWMTTEDMLSVWNRVWIEENPWMEDKTRVSSWEDVPYLGKREDQWCGSLIGLTARATWATNIQVAINQVRRLIGNENYSDYMTSMKRFKNESEPEGALW

>DENV1_AHG23212_Vietnam

MNNQRKKTARPSFNMLKRARNRVSTVSQLAKRFSKGLLSGQGPMKLVMAFIAFLRFLAIPPTAGILARWGSFKKSGAIKVLRGFKKEISNMLNIMNRRKRSVTMFLMLMPTALAFHLTTRGGEPHMIVSKQERGKSLLFKTSAGVNMCTLIAMDLGELCEDTMTYKCPRITETEPDDVDCWCNATDTWVTYGTCSQTGEHRRDKRSVALAPHVGLGLETRTETWMSSEGAWKQIQRVETWALRHPGFTVIALFLAHAIGTSITQKGIIFILLMLVTPSMAMRCVGIGSRDFVEGLSGATWVDVVLEHGSCVTTMAKDKPTLDIELLKTEVTNPAVLRKLCIEAKISNTTTDSRCPTQGEATLVEEQDANFVCRRTFVDRGWGNGCGLFGKGSLITCAKFKCVTKLEGKIVQYENLKYSVIVTVHTGDQHQVGNESTEHGTTATITPQAPTTEIQLTDYGALTLDCSPRTGLDFNEMVLLTMKEKSWLVHKQWFLDLPLPWTSGASTSQETWNRQDLLVTFKTAHAKKQEVVVLGSQEGAMHTALTGATEIQTSGTTTIFAGHLKCRLKMDKLTLKGMSYVMCTGSFKLEKELAETQHGTVLVQIKYEGTDAPCKIPFSTQDEKGVTQNGRLITANPIVTDKEKPVNIEAEPPFGESYIVIGAGEKALKLSWFKKGSSIGKMFEATARGARRMAILGDTAWDFGSIGGVFTSVGKLVHQIFGTAYGVLFSGVSWTMKIGIGVLLTWLGLNSRSTSLSMTCIAVGLVTLYLGVMVQADSGCVINWKGRELKCGSGIFVTNEVHTWTEQYKFQADSPKRLSAAIGKAWEEGVCGIRSATRLENIMWKQISNELNHILLENDMKFTVVVGDVAGILAQGKKMIRPQPMEYKYSWKSWGKAKIIGADVQNTTFIIDGPNTPECPDDQRAWNIWEVEDYGFGIFTTNIWLKLRDSYTQVCDHRLMSAAIKDSKAVHADMGYWIESEKNETWKLARASFIEVKTCIWPKSHTLWSNGVLESEMIIPKIYGGPISQHNYRPGYFTQTAGPWHLGKLELDFDLCEGTTVVVDEHCGNRGPSLRTTTVTGKIIHEWCCRSCTLPPLRFRGEDGCWYGMEIRPVKEKEENLVKSMVSAGSGEVDSFSLGLLCISIMIEEVMRSRWSRKMLMTGTLAVFFLLIMGQLTWNDLIRLCIMVGANASDRMGMGTTYLALMATFKMRPMFAVGLLFRRLTSREVLLLTIGLSLVASVELPNSLEELGDGLAMGIMILKLLTDFQSHQLWATLLSLTFIKTTFSLHYAWKTMAMVLSIVSLFPLCLSTTSQKTTWLPVLLGSLGCKPLTMFLIAENKIWGRRSWPLNEGIMAVGIVSILLSSLLKNDVPLAGPLIAGGMLIACYVISGSSADLSLEKAAEVSWEEEAEHSGASHNILVEVQDDGTMKIKDEERDDTLTILLKATLLAVSGVYPLSIPATLFVWYFWQKKKQRSGVLWDTPSPPEVERAVLDDGIYRIMQRGLLGRSQVGVGVFQENVFHTMWHVTRGAVLMYQGKRLEPSWASVKKDLISYGGGWRLQGSWNTGEEVQVIAVEPGKNPKNVQTAPGTFKTPEGEVGAIALDFKPGTSGSPIVNREGKIVGLYGNGVVTTSGTYVSAIAQARASQEGPLPEIEAEVFRKRNLTIMDLHPGSGKTRRYLPAIVREAIKRKLRTLILAPTRVGASEMAEALKGMPIRYQTTAVKSEHTGKEIVDLMGHATFTMGLLAPGRVPNYNMVIMDEAHFTDPSRIAARGYISTRVGMGEAAAIFMTATPPGSVEAFPQSNAVIQDEERDIPERSWNSGYEWITDFPGKTVWFVPSIKSGNDIANCLRKNGKRVIQLSRKTFDTEYQKTKNNDWDYVVTTDISEMGANFRADRVIDPRRCLNPVILKDGPERVILAGPMPVTVASAAQRRGRIGRNHNKEGDQYIYMGQPLNNDEDHAPWTEAKMLLDNINTPEGIIPALFEPEREKSAAIDGESRLRGEARKTFVELMRRGDLPVWLSYKVASEGFQYSDRRWCFDGERTNQVLEENMAVEIWPKEGERKKLRPRCLDARTYSDPLALREFKEFPPGRRSVSGDLILEIGKLPHPLTQRPQNALATLVMSHTSEQGGRAYRHAMEELPATIETLMLLALIAVLTGGVTLFFLSGRGLGKTSIGLLCVMASSVLLWMASVEPHWIAASIILEFFLMVLLIPEPDSNATRQDNQLAYVVIGLLFMILTVAANEMGLLETTKKDLGIGHVAVENHHHAAMLDVDLHPASAWTLYAVATTIITPMMRHTIENTTANISLTAIANQAAILMGLAKGWPISKMDIGVPLLALGCYSQVNPLTLTAPVLMLVPHYPIIGPGLQAKATREAQKRTAAGIMKNPTVDGIVAIDLDPVVYDAKFEKQLGQIMLLILCTSQILLMRTTWALCESITLATGPLTTLWEGSPGKFWNTTIAVSMANIFRGSYLAGAGLAFSLMKSLGGGRRGTGAQGETLGEKWKRQLNQLTKSEFNTYKRSGIMEVDRSEAKEGLKRGETTKHPVSRGTAKLRWFVERTLVKPEGKVIDLGCGRGGWSYYCAGLKKVTEVKGYTKGGPGHEDPIPMGTYGWNLVKLRSGKDVFFIPPEKCDTLLCDIGESSPNPTIEEGRTLRVLKMVEPWLRGNQFCIKILNPYMASVVETLEQMQRKHGGMLVRNPLSRNSTHEMYWVSWGTGNIVSAVNMTSRMLLNRFTMAHRKPTYERDVDLGAGTRHVAVEPEVANLDIIGQRIENIKHEHKSTWHYDEDNPYKTWAYHGSYEGKPSGSASSMVNGGVKLLTKPWDGIPMVTQIAMTDTTPFGQQRVFKEKVDTRTPKAKRGTAQIMEVTAKWLWGFLSRNKKPRICTREEFTRKVRSNAAIGAVFVDENQWNSAKEAVEDERFWDLVHRERELHKQGKCATCVYNMMGKREKKLGEFGKAKGSRAIWYMWLGARFLEFEALGFMNEDHWFSRENSLSGVEGEGLHKLGYILRDISKIPGGNMYADDTAGWDTRITEDDLQNEARITDIMEPEHALLAKSIFKLTYQNKVVRVQRPAKNGTVMDVISRRDQRGSGQVGTYGLNTFTNMEAQLIRQMESGGIFSPRELETPNLAERVLDWVEKYGVERLERMAISGDDCLGEPIYDRFATALTALNDMGKVRKDIPQWEPSKGWNDWQQGPFCSHHFHQLIMKDGREIVVPCRNQDELVGRARVSQGAGWSLRETACLGKSYAQMWQLMDFHRRDLRLAANAIWSAGPGDWIPTRRTTWSIHAHHQWMTTEDMLSVWNRVWIEENPWMEDKTHISSWGDVPYLGKREDQWCGSLIGLTARATWATNIQVAINQVRRLIGNENYLDYMASMKRFKNESDPEGALW

>DENV1_AGT63075_Laos

MNNQRKKTARPSFNMLKRARNRVSTVSQLAKRFSKGLLSGQGPMKLVMAFIAFLRFLAIPPTAGILARWGSFKKNGAIKVLRGFKKEISNMLNIMNRRKRSVTMLLMLMPTALAFHLTTRGGEPHMIVSKQERGKSLLFKTSAGVNMCTLIAMDLGELCEDTMTYKCPRITEAEPDDVDCWCNATDTWVTYGTCSQTGEHRRDKRSVALAPHVGLGLETRTETWMSSEGAWKQIQRVETWALRHPGFTVIALFLAHAIGTSITQKGIIFILLMLVTPSMAMRCVGIGSRDFVEGLSGATWVDVVLEHGSCVTTMAKDKPTLDIELLKTEVTNPAVLRKLCIEAKISNTTTDSRCPTQGEATLVEEQDANFVCRRTFVDRGWGNGCGLFGKGSLITCAKFKCVTKLEGKIVQYENLKYSVIVTVHTGDQHQVGNESTEHGTTATITPQAPTTEIQLTDYGALTLDCSPRTGLDFNEMVLLTMKEKSWLVHKQWFLDLPLPWTSGASTSQETWNRQDLLVTFKTAHAKKQEVVVLGSQEGAMHTALTGATEIQTSGTTTIFAGHLKCRLKMDKLTLKGMSYVMCTGSFKLEKEVAETQHGTVLVQIKYEGTDAPCKIPFSTQDEKGVTQNGRLITANPIVTDKEKPVNIEAEPPFGESYIVIGAGEKALKLSWFKKGSSIGKMFEATARGARRMAILGDTAWDFGSIGGVFTSVGKLVHQIFGTAYGVLFSGVSWTMKIGIGVLLTWLGLNSRSTSLSMTCIAVGLVTLYLGVMVQADSGCVVNWKGRELKCGSGIFVTNEVHTWTEQYKFQADSPKRLSAAIGKAWEEGVCGIRSATRLENVMWKQISNELNHILLENDMKFTVVVGDVAGILAQGKKMIRPQPMEYKYSWKSWGKAKIIGADVQNTTFIIDGPNTPECPDDQRAWNIWEVEDYGFGIFTTNIWLRLRDSYTQVCDHRLMSAAIKDSKAVHADMGYWIESEKNETWKLARASFIEVKTCIWPKSHTLWSNGVLESEMIIPKIYGGPISQHNYRPGYFTQTAGPWHLGKLELDFDLCEGTTVVVDEHCGNRGPSLRTTTVTGKTIHEWCCRSCTLPPLRFRGEDGCWYGMEIRPVKEKEENLVKSMVSAGSGEVDSFSLGLLCISIMIEEVMRSRWSRKMLMTGTLAVFFLLIIGQLTWNDLIRLSIMVGANASDKMGMGTTYLALMATFKMRPMFAVGLLFRRLTSREVLLLTIGLSLVASVELPNSLEELGDGLAMGIMILKLLTDFQPHQLWATLLSLTFIKTTFSLHYAWKTMAMVLSIVSLFPLCLSTTSQKTTWLPVLLGSLGCKPLTMFLITENKIWGRKSWPLNEGIMAVGIVSILLSSLLKNDVPLAGPLIAGGMLIACYVISGSSADLSLEKAAEVSWEEEAEHSGASHNILVEVQDDGTMKIKDEERDDTLTILLKATLLAVSGVYPLSIPATLFVWYFWQKKKQRSGVLWDTPSPPEVERAALDDGIYRIMQRGLLGRSQVGVGVFQENVFHTMWHVTRGAVLMYQGKRLEPSWASVKKDLISYGGGWRLQGSWNTGEEVQVIAVEPGKNPKNVQTAPGTFKTPEGEVGAIALDFKPGTSGSPIVNREGKIVGLYGNGVVTTSGTYVSAIAQAKASQEGPLPEIEDEVFRKRNLTIMDLHPGSGKTRRYLPAIVREAIKRKLRTLILAPTRVVASEMAEALKGMPIRYQTTAVKSEHTGKEIVDLMCHATFTMRLLSPVRVPNYNMIIMDEAHFTDPSSIAARGYISTRVGMGEAAAIFMTATPPGSVEAFPQSNAVIQDEERDIPERSWNSGYEWITDFPGKTVWFVPSIKSGNDIANCLRKNGKRVIQLSRKTFDTEYQKTKNNDWDYVVTTDISEMGANFRADRVIDPRRCLKPVILKDGPERVILAGPMPVTVASAAQRRGRIGRNHNKEGDQYIYMGQPLNNDEDHAHWTEAKMLLDNINTPEGIIPALFEPEREKSAAIDGEYRLRGEARKTFVELMRRGDLPVWLSYKVASEGFQYSDRRWCFDGERNNQVLEENMDVEIWTKEGERKKLRPRWLDARTYSDPLALREFKEFAAGRRSVSGDLILEIGKLPQHLTQRAQNALDNLVMLHNSEQGGRAYRHAMEELPDTIETLMLLALIAVLTGGVTLFFLSGRGLGKTSIGLLCVMASSVLLWMASVEPHWIAASIILEFFLMVLLIPEPDRQRTPQDNQLAYVVIGLLFMILTVAANEMGLLETTKKDLGIGHVADENHHHAAMLDVDLHPASAWTLYAVATTIITPMMRHTIENTTANISLTAIANQAAILMGLDKGWPISKMDIGVPLLALGCYSQVNPLTLTAAVLMLVAHYAIIGPGLQAKATREAQKRTAAGIMKNPTVDGIVAIDLDPVVYDAKFEKQLGQIMLLILCTSQILLMRTTWALCESITLATGPLTTLWEGSPGKFWNTTIAVSMANIFRGSYLAGAGLAFSLMKSLGGGRRGTGAQGETLGEKWKRQLNQLSKSEFNTYKRSGIMEVDRSEAKEGLKRGETTKHAVSRGTAKLRWFVERNLVKPEGKVIDLGCGRGGWSYYCAGLKKVTEVKGYTKGGPGHEEPIPMATYGWNLVKLHSGKDVFFIPPEKCDTLLCDIGESSPNPTIEEGRTLRVLKMVEPWLRGNQFCIKILNPYMPSVVETLEQMQRKHGGMLVRNPLSRNSTHEMYWVSCGTGNIVSAVNMTSRMLLNRFTMVHRKPTYERDVDLGAGTRHVAVEPEVANLDIIGQRIENIKHEHKSTWHYDEDNPYKTWAYHGSYEVKPSGSASSMVNGVVKLLTKPWDVIPMVTQIAMTDTTPFGQQRVFKEKVDTRTPKAKRGTAQIMEVTARWLWGFLSRNKKPRICTREEFTRKVRSNAAIGAVFIDENQWNSAKEAVEDERFWDLVHRERELHKQGKCATCVYNMMGKREKKLGEFGKAKGSRAIWYMWLGARFLEFEALGFMNEDHWFSRENSLSGVEGEGLHKLGYILRDISKIPGGNMYADDTAGWDTRITEDDLQNEAKITDIMESEHALLAKSIFKLTYQNKVVRVQRPAKNGTVMDVISRRDQRGSGQVGTYGLNTFTNMEAQLIRQMESEGIFSPSELETPNLAERALDWLEKHGVERLKRMAISGDDCVVKPIDDRFATALTALNDMGKVRKDIPQWEPSKGWNDWQQVPFCSHHFHQLIMKDGREIVVPCRNQDELVGRARVSQGAGWSLRETACLGKSYAQMWQLMYFHRRDLRLAANAICSAVPVDWVPTSRTTWSIHAHHQWMTTEDMLSVWNRVWIEENPWMEDKTHISSWEDVPYLGKREDQWCGSLIGLTARATWATNIQVAISQVRRLIGNENYLDYMTSMKRFKNESDPEGALW

>DENV1_AGT63074_Laos

MNNQRKKTARPSFNMLKRARNRVSTVSQLAKRFSKGLLSGQGPMKLVMAFIAFLRFLAIPPTAGILARWGSFKKNGAIKVLRGFKKEISNMLNIMNRRKRSVTMLLMLMPTALAFHLTTRGGEPHMIVSKQERGKSLLFKTSAGVNMCTLIAMDLGELCEDTMTYKCPRITEAEPDDVDCWCNATDTWVTYGTCSQAGEHRRDKRSVALAPHVGLGLETRTETWMSSEGAWKQIQRVETWALRHPGFTVIALFLAHAIGTSITQKGIIFILLMLVTPSMAMRCVGIGSRDFVEGLSGATWVDVVLEHGSCVTTMAKDKPTLDIELLKTEVTNPAVLRKLCIEAKISNTTTDSRCPTQGEATLVEEQDANFVCRRTFVDRGWGNGCGLFGKGSLLTCAKFKCVTKLEGKIVQYENLKYSVIVTVHTGDQHQVGNESTEHGTTATITPQAPTTEIQLTDYGALTLDCSPRTGLDFNEMVLLTMKEKSWLVHKQWFLDLPLPWTSGASTSQETWNRQDLLVTFKTAHAKKQEVVVLGSQEGAMHTALTGATEIQTSGTTTIFAGHLKCRLKMDKLTLKGMSYVMCTGSFKLEKEVAETQHGTVLVQIKYEGTDAPCKIPFSTQDEKGVTQNGRLITANPIVTDKEKPVNIEAEPPFGESYIVIGAGEKALKLSWFKKGSSIGKMFEATARGARRMAILGDTAWDFGSIGGVFTSVGKLVHQIFGTAYGVLFSGVSWTMKIGIGVLLTWLGLNSRSTSLSMTCIAVGLVTLYLGVMVQADSGCVINWKGRELKCGSGIFVTNEVHTWTEQYKFQADSPKRLSAAIGKAWEEGVCGIRSATRLENIMWKQISNELNHILLENDMKFTVVVGDVAGILAQGKKMIRPQPMEYKYSWKSWGKAKIIGADVQNSTFIIDGPNTPECPDDQRAWNIWEVEDYGFGIFTTNIWLKLRDSYTQVCDHRLMSAAIKDSKAVHADMGYWIESEKNETWKLARASFIEVKTCIWPKSHTLWSNGVLESEMIIPKIYGGPISQHNYRPGYFTQTAGPWHLGKLELDFDLCEGTTVVVDEHCGNRGPSLRTTTVTGKIIHEWCCRSCTLPPLRFRGEDGCWYGMEIRPVKEKEENLVKSMVSAGSGEVDSFSLGLLCISIMIEEVMRSRWSRKMLMTGTLAVFFLLIMGQLTWNDLIRLCIMVGANASDRMGMGTTYLALMATFKMRPMFAVGLLFRRLTSREVLLLTIGLSLVASVELPNSLEELGDGLAMGIMILKLLTDFQSHQLWATLLSLTFIKTTFSLHYAWKTMAMVLSIVSLFPLCLSTTSQKTTWLPVLLGSLGCKPLTMFLIAENKIWGRKSWPLNEGIMAVGIVSILLSSLLKNDVPLAGPLIAGGMLIACYVISGSSADLSLEKAAEVSWEEEAEHSGASHNILVEVQDDGTMKIKDEERDDTLTILLKATLLAVSGVYPLSIPATLFVWYFWQKKKQRSGVLWDTPSPPEVERAVLDDGIYRIMQRGLLGRSQVGVGVFQENVFHTMWHVTRGAVLMYQGKRLEPSWASVKKDLISYGGGWRFQGSWNTGEEVQVIAVEPGKNPKNVQTAPGTFKTPEGEVGAIALDFKPGTSGSPIVNREGKIVGLYGNGVVTTSGTYVSAIAQAKASQEGPLPEIEDEVFRKRNLTIMDLHPGSGKTRRYLPAIVREAIKRKLRTLILAPTRVVASEMAEALKGMPIRYQTTAVKSEHTGKEIVDLMCHATFTMRLLSPVRVPNYNMIIMDEAHFTDPSSIAARGYISTRVGMGEAAAIFMTATPPGSMEAFPQSNAVIQDEERDIPERSWNLGHEWITDFPGKTVWFVPSIKAGNDIANCLRKNGKRVIQLSRKTFDTEYQKTKNNDWDYVVTTDISEMGANFRADRVIDPRRCLKPVILKDGPERVILAGPMPVTVASAAQRRGRIGRNHNKEGDQYIYMGQPLNNDEDHAHWTEAKMLLDNINTPEGIIPALFEPEREKSAAIDGEYRLRGEARKTFVELMRRGDLPVWLSYKVASEGFQYSDRRWCFDGERNNQVLEENMDVEIWTKEGERKKLRPRWLDARTYSDPLALREFKEFAAGRRSVSGDLILEIGKLPQHLTQRAQNALDNLVILHNSEQGGRAYRHAMEELPDTIETLMLLALIAVLTGGVTLFFLSGKGLGKTSIGLLCVMASSVLLWMASVEPHWIAASIILEFFLMVLLIPEPDRQRTPQDNQLAYVVIGLLFMILTVAANEMGLLETTKKDLGIGHVAVENHHHAAMLDVDLHPASAWTLYAVATTIITPMMRHTIENTTANISLTAIANQAAILMGLDKGWPISKMDIGVPLLALGCYSQVNPLTLTAAVLMLVAHYAIIGPGLQAKATREAQKRTAAGIMKNPTIDGIVAIDLDPVVYDAKFEKQLGQIMLLILCTSQILLMRTTWALCESITLATGPLTTLWEGSPGKFWNTTIAVSMANIFRGSYLAGAGLAFSLMKSLGGGRRGTGAQGETLGEKWKRQLNQLSKSEFNTYKRSGIMEVDRSEAKEGLKRGETTKHAVSRGTAKLRWFVERNLVKPEGKVIDLGCGRGGWSYYCAGLKKVTEVKGYTKGGPGHEEPIPMATYGWNLVKLHSGKDVFFVPPEKCDTLLCDIGESSPNPTIEEGRTLRVLKMVEPWLRGNQFCIKILNPYMPSVVETLEQMQRKHGGMLVRNPLSRNSTHEMYWVSCGTGNIVSAVNMTSRMLLNRFTMAHRKPTYERDVDLGAGTRHVAVEPEVANLDIIGQRIENIKHEHKSTWHYDEDNPYKTWAYHGSYEVKPSGSASSMVNGVVKLLTKPWDVIPMVTQIAMTDTTPFGQQRVFKEKVDTRTPKAKRGTAQIMEVTARWLWGFLSRNKKPRICTREEFTRKVRSNAAIGAVFVDENQWNSAKEAVEDDRFWDLVHRERELHKQGKCATCVYNMMGKREKKLGEFGKAKGSRAIWYMWLGARFLEFEALGFMNEDHWFSRENSLSGVEGEGLHKLGYILRDISKIPGGNMYADDTAGWDTRITEDDLQNEAKITDIMEPEHALLAKSIFKLTYQNKVVRVQRPAKNGTVMDVISRRDQRGSGQVGTYGLNTFTNMEAQLIRQMESEGIFSPNELETPNLAERVLDWLEKYGVERLKRMAISGDDCVVKPIDDRFATALTALNDMGKVRKDIPQWEPSKGWNDWQQVPFCSHHFHQLIMKDGREIVVPCRNQDELVGRARVSQGAGWGLRETACLGKSYAQMWQLMYFHRRDLRLAANAICSAVPVDWVPTSRTTWSIHAHHQWMTTEDMLSVWNRVWIEENPWMEDKTRISSWEDVPYLGKREDQWCGSLIGLTARATWATNIQVAINQVRRLIGNENYSDYMTSMKRFKNESEPEGALW

>DENV1_ACO06148_Thailand

MNNQRKKTARPSFNMLKRARNRVSTVSQLAKRFSKGLLSGQGPMKLVMAFIAFLRFLAIPPTAGILARWGSFKKNGAIKVLRGFKKEVSNMLNIMNRRKRSVTMLLMLMPTALAFHLTTRGGEPHMIVSKQERGKSLLFKTSAGVNMCTLIAMDLGELCEDTMTYKCPRITEAEPDDVDCWCNATDTWVTYGTCSQTGEHRRDKRSVALAPHVGLGLETRTETWMSSEGAWKQIQRVETWALRHPGFTVIALFLAHAIGTSITQKGIIFILLMLVTPSMAMRCVGIGSRDFVEGLSGATWVDVVLEHGSCVTTMAKDKPTLDIELLKTEVTNPAVLRKLCIEAKISNTTTDSRCPTQGEATLVEEQDANFVCRRTFVDRGWGNGCGLFGKGSLLTCAKFKCVTKLEGKIVQYENLKYSVIVTVHTGDQHQVGNESTEHGTTATITPQAPTTEIQLTDYGALTLDCSPRTGLDFNEMVLLTMKEKSWLVHKQWFLDLPLPWTSGASTSQETWNRQDLLVTFKTAHAKKQEVVVLGSQEGAMHTALTGATEIQTSGTTTIFAGHLKCRLKMDKLTLKGMSYVMCTGSFKLEKEVAETQHGTVLVQIKYEGTDAPCKIPFSTQDEKGVTQNGRLITANPIVTDKEKPVNIEAEPPFGESYIVIGAGEKALKLSWFKKGSSIGKMFEATARGARRMAILGDTAWDFGSIGGVFTSVGKLVHQIFGTAYGVLFSGVSWTMKIGIGVLLTWLGLNSRSTSLSMTCIAVGLVTLYLGVMVQADSGCVINWKGRELKCGSGIFVTNEVHTWTEQYKFQADSPKRLSAAIGKAWEEGVCGIRSATRLENIMWKQISNELNHILLENDMKFTVVVGDVAGILAQGKKMIRPQPMEYKYSWKSWGKAKIIGADVQNSTFIIDGPNTPECPDDQRAWNIWEVEDYGFGIFTTNIWLKLRDSYTQVCDHRLMSAAIKDSKAVHADMGYWIESEKNETWKLAGASFIEVKTCIWPKSHTLWSNGVLESEMIIPKIYGGPISQHNYRPGYFTQTAGPWHLGKLELDFDLCEGTTVVVDEHCGNRGPSLRTTTVTGKIIHEWCCRSCTLPPLRFRGEDGCWYGMEIRPVKEKEENLVKSMVSAGSGEVDSFSLGLLCISIMIEEVMRSRWSRKMLMTGTLAVFFLLIMGQLTWNDLIRLCIMVGANASDRMGMGTTYLALMATFKMRPMFAVGLLFRRLTSREVLLLTIGLSLVASVELPNSLEELGDGLAMGIMILKLLTDFQSHQLWATLLSLTFIKTTFSLHYAWKTMAMVLSIVSLFPLCLSTTSQKTTWLPVLLGSLGCKPLTMFLIAENKIWGRKSWPLNEGIMAVGIVSILLSSLLKNDVPLAGPLIAGGMLIACYVISGSSADLSLEKAAEVSWEEEAEHSGASHNILVEVQDDGTMKIKDEERDDTLTILLKATLLAVSGVYPLSIPATLFVWYFWQKKKQRSGVLWDTPSPPEVERAVLDDGIYRIMQRGLLGRSQVGVGVFQENVFHTMWHVTRGAVLMYQGKRLEPSWASVKKDLISYGGGWRLQGSWNTGEEVQVIAVEPGKNPKNVQTAPGTFKTPEGEVGAIALDFKPGTSGSPIVNREGKIVGLYGNGVVTTSGTYVSAIAQAKASQEGPLPEIEDEVFRKRNLTIMDLHPGSGKTRRYLPAIVREAIKRKLRTLILAPTRVVASEMAEALKGMPIRYQTTAVKSEHTGKEIVDLMCHATFTMRLLSPVRVPNYNMIIMDEAHFTDPSSIAARGYISTRVGMGEAAAIFMTATPPGSVEAFPQSNAVIQDEERDIPERSWNSGYEWITDFPGKTVWFVPSIKAGNDIANCLRKNGKRVIQLSRKTFDTEYQKTKNNDWDYVVTTDISEMGANFRADRVIDPRRCLKPVILKDGPERVILAGPMPVTVASAAQRRGRIGRNHNKEGDQYIYMGQPLNNDEDHAHWTEAKMLLDNINTPEGIIPALFEPEREKSAAIDGEYRLRGEARKTFVELMRRGDLPVWLSYKVASEGFQYSDRRWCFDGERNNQVLEENMDVEIWTKEGERKKLRPRWLDARTYSDPLALREFKEFAAGRRSVSGDLILEIGKLPQHLTQRAQNALDNLVMLHNSEQGGRAYRHAMEELPDTIETLMLLALIAVLTGGVTLFFLSGKGLGKTSIGLLCVMASSVLLWMASVEPHWIAASIILEFFLMVLLIPEPDRQRTPQDNQLAYVVIGLLFMILTVAANEMGLLETTKKDLGIGHVAVENHHHAAMLDVDLHPASAWTLYAVATTIITPMMRHTIENTTANISLTAIANQAAILMGLDKGWPISKMDIGVPLLALGCYSQVNPLTLTAAVLMLVAHYAIIGPGLQAKATREAQKRTAAGIMKNPTIDGIVAIDLDPVVYDAKFEKQLGQIMLLILCTSQILLMRTTWALCESITLATGPLTTLWEGSPGKFWNTTIAVSMANIFRGSYLAGAGLAFSLMKSLGGGRRGTGAQGETLGEKWKRQLNQLSKSEFNTYKRSGIMEVDRSEAKEGLKRGETTKHAVSRGTAKLRWFVERNLVKPEGKVIDLGCGRGGWSYYCAGLKKVTEVKGYTKGGPGHEEPIPMATYGWNLVKLHSGKDVFFIPPEKCDTLLCDIGESSPNPTIEEGRTLRVLKMVEPWLRGNQFCIKILNPYMPSVVETLEQMQRKHGGMLVRNPLSRNSTHEMYWVSCGTGNIVSAVNMTSRMLLNRFTMAHRKPTYERDVDLGAGTRHVAVEPEVANLDIIGQRIENIKHEHKSTWHYDEDNPYKTWAYHGSYEVKPSGSASSMVNGVVKLLTKPWDVIPMVTQIAMTDTTPFGQQRVFKEKVDTRTPKAKRGTAQIMEVTARWLWGFLSRNKKPRICTREEFTRKVRSNAAIGAVFVDENQWNSAKEAVEDDRFWDLVHRERELHKQGKCATCVYNMMGKREKKLGEFGKAKGSRAIWYMWLGARFLEFEALGFMNEDHWFSRENSLSGVEGEGLHKLGYILRDISKIPGGNMYADDTAGWDTRITEDDLQNEAKITDIMEPEHALLAKSIFKLTYQNKVVRVQRPAKNGTVMDVISRRDQRGSGQVGTYGLNTFTNMEAQLIRQMESEGIFSPNELETPNLAERVLDWLEKYGVERLKRMAISGDDCVVKPIDDRFATALTALNDMGKVRKDIPQWEPSKGWNDWQQVPFCSHHFHQLIMKDGREIVVPCRNQDELVGRARVSQGAGWSLRETACLGKSYAQMWQLMYFHRRDLRLAANAICSAVPVDWVPTSRTTWSIHAHHQWMTTEDMLSVWNRVWIEENPWMEDKTRISSWEDVPYLGKREDQWCGSLIGLTARATWATNIQVAINQVRRLIGNENYSDYMTSMKRFKNESEPEGALW

>DENV1_AMN88556_Brunei

MNNQRKKTGRPSFNMLKRARNRVSTVSQLAKRFSKGLLTGQGPMKLVMAFIAFLRFLAIPPTAGILARWGSFKKNGAIKVLRGFKKEISNMLNIMNRRRRTVVALLMLLPMVFSFHLTTRGGEPHMIVNKQERGKSLLFKTSGGINMCTLIAMDLGDLCEDTMTYKCPRITEAEPDDVDCWCNATDTWVTYGICSPTGEHRREKRSVALAPHVGLGLETRAETWMSSEGAWKQIQRVETWALRHPGFTVIALFLAHIIGTSITQKGVIFILLMLVTPSMAMRCVGVGNRDFVEGLSGATWVDVVLEHGSCVTTMAKNKPTLDIELLKTEVSNPAVLRKLCIEARISNTTTDSRCPTQGEASLSEEQDTNFVCRRTLVDRGWGNGCGLFGKGSLITCAKFECLTKMEGKIVQYENLKYSVIVTVHTGDQHQVGNETTGHGTTATITPQAPTSEIQLTDYGALTLDCSPRTGLDFNEMVLLTMKEKSWLVHKQWFLDLPLPWASGASSPQETWNRQDLLVTFKTAHAKKQEVVVLGSQEGAMHTALTGATEIQTSGTTTIFAGHLKCRLKMDKLTLKGMSYVMCTGSFKLKKEVAETQHGTVLVQVEYEGTDAPCKIPFTTQDEKGVIQNGRLITANPIVTDKEKPVNIEAEPPFGESYMVIGVGDKALKLSWFKKGSSLGKMFEATARGARRMAILGDTAWDFGSVGGVFTSMGKLVHQIFGTAYGVLFSGVSWTMKIGIGLLLTWLGLNSRSTSLSMTCIAVGIITLYLGVMVQADTGCVVNWKGRELKCGSGIFVTNEVHTWTEQYKFQADSPKRLSAAIGKAWEDGVCGIRSATRLENIMWKQIANELNYILLENDMKFTVVVGDVKGILTQGKKGIRPQPMEHKYSWKSWGKAKIIGADVQNTTFIIDGPDTPECPDSQRAWNIWEVEDYGFGIFTTNIWLKLRDSYTQVCDHRLMSAAIKDSRAVHADMGYWIESEKNETWKLSRASFIEVKTCTWPKSHTLWSNGVLESEMIIPKIYGGPISQHNYRPGYFTQTAGPWHLGKLELDFDLCEGTTVVVDEQCGNRGPSLRTTTVTGKIIHEWCCRSCTLPPLRFKGEDGCWYGMEIRPVKEKEENLVKSMVSAGSGKVDNFSLGLLCISIMVEEVMRSRWSGKMLMTGTMAVFLLLVMGQLTWNDLIRVCIMVGAHALDSMGMGTTYLALMATFKIRPMLAVGLLFRKLTSREILLLTIGLSLITPVELPNSIEELGDGLAMGIMMLKLLTDFQPYQLWATLLSLTFVKTTLSLHYAWKTAAMVLSIVSLLPLCLSTTSQKTTWLPILLGSLGCKPLTMFLISENKIWGRRSWPINEGIMAVGIVSILMSSLLKNDVPLAGPLIAGGMLVACYVISGSSADLTLEKTAEVSWEEEAEHSGTAHNILVEVQDDGTMKIKDEEREDTLTILLKAALLALSGVYPMSIPATLFVWYFWQKKKQRSGVLWDIPSPPEVEKAVLDDGIYRILQRGVFGRSQVGVGVFQEGVFHTMWHVTRGAVLTYQGKRLEPSWASVKKDLISYGGGWRLQGSWNVGEEVQVIAVEPGKNPKNVQTVPGTFKTHEGEVGAIALDFKPGTSGSPIVNRDGKVVGLYGNGVVTTSGTYVSAIAQSKVSQEGPLPEIEDEVFKKKNLTIMDLHPGSGKTRRYLPAIVRESIKRRLRTLILAPTRVVASEMAEALKGLPIRYQTTAVRNEHTGREIVDLMCHATFTMRLLSPVRVPNYNMIIMDEAHFTDPASIAARGYISTRVGMGEAAAIFMTATPPGSAEAFPQSNSVIYDEEKDIPERSWNSGYDWITDFQGKTVWFVPSIKTGNDIANCLRKNGKRVIQLSRKTFDTEYQKTKNSDWDFVVTTDISEMGANFRADRVIDPRRCLKPVILRDGPERVILAGPMPVTVASAAQRRGRIGRNQSKEGDQYIYMGQPLNNDEDHAHWTEAKMLLDNINTPEGIIPALFEPEREKSAAIDGEYRLRGEARKTFVELMRRGDLPVWLSYKVASEGIQYADRKWCFDGERNNQILEENMDVEIWTKEGERKKLRPRWLDARTYSDPLALREFKEFAAGRRSVSGDLILEIGKLPHHLTQRAQNALDNLVMLHNSEQGGRAYRHAMEELPDTIETLMLLAMIATLTGGVALFFLSGKGLGKTSIGLLCVMASSTLLWMASVEPHWIAASIVLEFFLMVLLIPEPDRQRTPQDNQLAYVVIGLLVLILTVAANEMGLLETTKKDLGIGSAAAEIQHHTTILDVDLHPASAWTLYAVATTVITPMLRHTIENTTANMSLTAIANQAAILMGLDKGWPISKMDIGVPLLALGCYSQVNPMTLTAAVLMLVAHYAIIGPGLQAKATRDAQKRTAAGIMKNPTVDGIVAIDLDPVVYDTKFEKQLGQIMLLILCTSQILLMRTTWAFCESITLATGPLTTLWEGSPGKFWNTTIAVSMANIFRGSYLAGAGLAFSLMKSLGGNRRGTGAQGETLGEKWKRQLNQLSKLEFNTYKRSGIMEVDRTEAKEGLKRGETTRHAVSRGTAKLRWFVERNLVKPEGKVIDLGCGRGGWSYYCAGLKKVTEVKGYTKGGPGHEEPIPMATYGWNLVKLHSGKDVFFIPPEKCDTLLCDIGESSPNPTIEEGRTLRVLKMVEPWLRGNQFCIKVLNPYMPSVVETLERMQRKHGGMLVRNPLSRNSTHEMYWVSCGTGNIVSAVNMTSRMLLNRFTMTHRKPTYEKDVDLGAGTRHVAVEPEVANLDIIGQRIENIKNEHKSTWHYDEDNPYKTWAYHGSYEVKPSGSASSMVNGVVRLLTKPWDVIPMVTQIAMTDTTPFGQQRVFKEKVDTRTPKAKRGTAQVMEITAGWLWKFLSRKKKPRICTREEFTRKVRSNAAIGAVFVDENQWNSAKEAVDDERFWDLVNRERELHKQGKCATCVYNMMGKREKKLGEFGKAKGSRAIWYMWLGARFLEFEALGFMNEDHWFSRDNSLSGVEGEGLHKLGYILRDIARIPGGNMYADDTAGWDTRITEEDLQNEARVTELMEPDHAQLATSIFRLTYQNKVVRVQRPGKNGTVMDVISRRDQRGSGQVGTYGLNTFTNMEAQLIRQMESEGVFSPSELETPNLANRVLDWLEKYGVERLKRMAISGDDCVVKPIDDRFATALTALNDMGKVRKDIPQWEPSRGWNDWQQVPFCSHHFHQLIMKDGREIVVPCRNQDELVGRARVSQGAGWSLRETACLGKSYAQMWQLMYFHRRDLRLAANAICSAVPVDWVPTSRTTWSIHAHHQWMTTEDMLSVWNRVWIEENPWMEDKTHVTSWEDVPYLGKREDQWCGSLIGLSARATWAINIQVAINQVRKLIGNEHYQDYMTSMRRFKNESEPEGALW

>DENV1_AET43254_Cambodia

MNNQRKKTARPSFNMLKRARNRVSTVSQLAKRFSKGLLSGQGPMKLVMAFIAFLRFLAIPPTAGILARWGSFKKNGAIKVLRGFKKEISNMLNIMNRRKRSVTMLLMLMPTALAFHLTTRGGEPHMIVSKQERGKSLLFKTSAGVNMCTLIAMDLGELCEDTLTYKCPRITEVEPDDVDCWCNTTDTWVTYGTCSQTGEHRRDKRSVALAPHVGLGLETRAETWMSSEGAWKQIQRVETWALRHPGFTVIALFLAHAIGTSITQKGIIFILLMLVTPSMAMRCVGIGSRDFVEGLSGATWVDVVLEHGSCVTTMAKDKPTLDIELLKTEVTNPAVLRKLCIEAKISNTTTDSRCPTQGEATLVEEQDANFVCRRTFVDRGWGNGCGLFGKGSLITCAKFKCVTKLEGKIVQYENLKYSVIVTVHTGDQHQVGNESTEHGTTATITPQAPTSEIQLTDYGALTLDCSPRTGLDFNEMVLLTMKEKSWLVHKQWFLDLPLPWTSGASTSQETWNRQDLLVTFKTAHAKKQEVVVLGSQEGAMHTALTGATEIQTSGTTTIFAGHLKCRLKMDKLTLKGMSYVMCTGSFKLEKEVAETQHGTVLVQIKYEGTDAPCKIPFSTQDGKGVTQNGRLITANPIVTDKEKPVNIEAEPPFGESYIVIGAGEKALKLSWFKKGSSIGKMFEATARGARRMAILGDTAWDFGSVGGVFTSVGKLVHQIFGTAYGVLFSGVSWTMKIGIGVLLTWLGLNSRSTSLSMTCIAVGLVTLYLGVMVQADSGCVINWKGRELKCGSGIFVTNEVHTWTEQYKFQADSPKRLSAAIGKAWEEGVCGIRSATRLENVMWRQISNELNHILLENDMKFTVVVGDVVGILAQGKKMIRPQPMEHKYSWKSWGKAKIIGADVQNTTFIIDGPNTSECPDDQRAWNIWEVEDYGFGIFTTNIWLKLRDSYTQVCDHRLMSAAIKDSRAVHADMGYWIESEKNETWKLARASFIEVKTCIWPKSHTLWSNGVLESEMIIPKIYGGPISQHNYRPGYFTQTAGPWHLGKLELDFDLCEGTTVVVDEHCGNRGPSLRTTTVTGKIIHEWCCRSCTLPPLRFKGEDGCWYGMEIRPVKEKEENLVKSMVSAGSGEVDSFSLGLLCISIMIEEVMRSRWSRKMLMTGTLAVFFLLIMGQLTWNDLIRLCIMVGANASDRMGMGTTYLALMATFKMRPMFAVGLLFRRLTSREVLLLTIGLSLVASVELPNSLEELGDGLAMGIMILKLLTDFQSYQLWATLLSLTFIKTTFSLHYAWKTMAMVLSIVSLLPLCLSTTSQKTTWLPVLLGSLGCKPLTMFLIAENKIWGRKSWPLNEGIMAVGIVSILLSSLLKNDVPLAGPLIAGGMLIACYVISGSSADLSLEKAAEVSWEEEAEHSGASHNILVEVQDDGTMKIKDEERDDTLTILLKATLLAVSGVYPLSIPATLFVWYFWQKKKQRSGVLWDTPSPPEVERAVLDDGIYRIMQRGLLGRSQVGVGVFQENVFHTMWHVTRGAVLMYQGKRLEPSWASVKKDLISYGGGWRLQGSWNTGEEVQVIAVEPGKNPKNVQTAPGTFKTPEGEVGAIALDFKPGTSGSPIVNREGKIVGLYGNGVVTTSGTYVSAIAQAKASQEGPLPEIEDEVFRKRNLTIMDLHPGSGKTRRYLPAIVREAIKRKLRTLILAPTRVVASEMAEALKGMPIRYQTTAVKSEHTGREIVDLMCHATFTMRLLSPVRVPNYNMIIMDEAHFTDPSSIAARGYISTRVGMGEAAAIFMTATPPGSVEAFPQSNAVIQDEERDIPERSWNSGYEWITDFPGKTVWFVPSIKSGNDIANCLRKNGKRVIQLSRKTFDTEYQKTKNNDWDYVVTTDISEMGANFRADRVIDPRRCLKPVILKDGPERVILAGPMPVTVASAAQRRGRIGRNHNKEGDQYIYMGQPLNNDEDHAHWTEAKMLLDNINTPEGIIPALFEPEREKSAAIDGEYRLRGEARKTFVELMRRGDLPVWLSYKVASEGFQYSDRRWCFDGERNNQVLEENMDVEIWTKEGERKKLRPRWLDARTYSDPLALREFKEFAAGRRSVSGDLILEIGKLPQHLTQRAQNALDNLVMLHNSEQGGRAYRHAMEELPETIETLMLLALIAVLTGGVTLFFLSGRGLGKTSIGLLCVMASSALLWMASVEPHWIAASIILEFFLMVLLIPEPDRQRTPQDNQLAYVVIGLLFMILTVAANEMGLLETTKKDLGIGHVAVENHHHATMLDVDLHPASAWTLYAVATTIITPMMRHTIENTTANISLTAIANQAAILMGLDKGWPISKMDIGVPLLALGCYSQVNPLTLTAAVLMLVAHYAIIGPGLQAKATREAQKRTAAGIMKNPTVDGIVAIDLDPVVYDAKFEKQLGQIMLLILCTSQILLMRTTWALCESITLATGPLTTLWEGSPGKFWNTTIAVSMANIFRGSYLAGAGLAFSLMKSLGGGRRGTGAQGETLGEKWKRQLNQLSKSEFNTYKRSGIMEVDRSEAKEGLRRGETTKHAVSRGTAKLRWFVERNLVKPEGKVIDLGCGRGGWSYYCAGLKKVTEVKGYTKGGPGHEEPIPMATYGWNLVKLHSGKDVFFIPPEKCDTLLCDIGESSPNPTIEEGRTLRVLKMVEPWLRGNQFCIKILNPYMPSVVETLEQMQRKHGGMLVRNPLSRNSTHEMYWVSCGTGNIVSAVNMTSRMLLNRFTMAHRKPTYERDVDLGAGTRHVAVEPEVANLDIIGQRIENIKHEHKSTWHYDEDNPYKTWAYHGSYEVKPSGSASSMVNGVVKLLTKPWDVIPMVTQIAMTDTTPFGQQRVFKEKVDTRTPKAKRGTAQIMEVTARWLWGFLSRNKKPRICTREEFTRKVRSNAAIGAVFVDENQWNSAKEAVEDERFWDLVHRERELHKQGKCATCVYNMMGKREKKLGEFGKAKGSRAIWYMWLGARFLEFEALGFMNEDHWFSRENSLSGVEGEGLHKLGYILRDISKIPGGNMYADDTAGWDTRITEDDLQNEAKITDIMEPEHALLATSIFKLTYQNKVVRVQRPAKNGTVMDVISRRDQRGSGQVGTYGLNTFTNMEAQLIRQMESEGIFSPSELETPNLAERVLDWLEKYGVERLKRMAISGDDCVVKPIDDRFATALTALNDMGKVRKDIPQWEPSKGWNDWQQVPFCSHHFHQLIMKDGREIVVPCRNQDELVGRARVSQGAGWSLRETACLGKSYAQMWQLMYFHRRDLRLAANAICSAVPVDWVPTSRTTWSIHAHQQWMTTEDMLSVWNRVWIEENPWMEDKTHISSWGDVPYLGKREDQWCGSLIGLTARATWATNIQVAINQVRRLIGNENYLDYMTSMKRFKNESDPEGALW

>DENV1_ARO84721_Malaysia

MNNQRKKTARPSFNMLKRARNRVSTVSQLAKRFSKGLLSGQGPMKLVMAFIAFLRFLAIPPTAGILARWGSFKKNGAIKVLRGFKKEISNMLNIMNRRKRSVTMLLMLMPTALAFHLTTRGGEPHMIVSKQERGKSLLFKTSAGVNMCTLIAMDLGELCEDTMTYKCPRITEAEPDDVDCWCNATDTWVTYGTCSQTGEHRRDKRSVALAPHVGLGLETRTETWMSSEGAWKQIQRVETWALRHPGFTVIALFLAHAIGTSITQKGIIFILLMLVTPSMAMRCVGIGSRDFVEGLSGATWVDVVLEHGSCVTTMAKDKPTLDIELLKTEVTNPAVLRKLCIEAKISNTTTDSRCPTQGEATLVEEQDANFVCRRTFVDRGWGNGCGLFGKGSLITCAKFKCVTKLEGKIVQYENLKYSVIVTVHTGDQHQVGNESTEHGTTATITPQAPTTEIQLTDYGALTLDCSPRTGLDFNEMVLLTMKEKSWLVHKQWFLDLPLPWTSGASTSQETWNRQDLLVTFKTAHAKKQEVVVLGSQEGAMHTALTGATEIQTSGTTTIFAGHLKCRLKMDKLTLKGMSYVMCTGSFKLEKEVAETQHGTVLVQIKYEGTDAPCKIPFSTQDEKGVTQNGRLITANPIVTDKEKPVNIEAEPPFGESYIVIGAGEKALKLSWFKKGSSIGKMFEATARGARRMAILGDTAWDFGSIGGVFTSVGKLVHQIFGTAYGVLFSGVSWTMKIGIGVLLTWLGLNSRSTSLSMTCIAVGLVTLYLGVMVQADSGCVINWKGRELKCGSGIFVTNEVHTWTEQYKFQADSPKRLSAAIGKAWEEGVCGIRSATRLENIMWKQISNELNHILLENDMKFTVVVGDVAGILAQGKKMIRPQPMEHKYSWKSWGKAKIIGADVQNTTFIIDGPNTPECPDDQRAWNIWEVEDYGFGIFTTNIWLKLRDSYTQVCDHRLMSAAIKDSKAVHADMGYWIESEKNETWKLARASFIEVKTCIWPKSHTLWSNGVLEGEMIIPKIYGGPISQHNYRPGYFTQTAGPWHLGKLELDFDLCEGTTVVVDEHCGNRGPSLRTTTVTGKIIHEWCCRSCTLPPLRYRGEDGCWYGMEIRPVKEKEENLVKSMVSAGSGEVDSFSLGLLCISIMIEEVMRSRWSRKMLMTGTLAVFFLLIMGQLTWNDLIRLCIMVGANASDRMGMGTTYLALMATFKMRPMFAVGLLFRRLTSREVLLLTIGLSLVASVELPNSLEELGDGLAMGIMILKLLTDFQSHQLWATLLSLTFIKTTFSLHYAWKTIAMVLSIVSLFPLCLSTTSQKTTWLPVLLGSLGCKPLTMFLIAENKIWGRKSWPLNEGIMAVGIVSILLSSFLKNDVPLAGPLIAGGMLIACYVISGSSADLSLEKAAEVSWEEEAEHSGASHNILVEVQDDGTMKIKDEERDDTLTILLKATLLAVSGVYPLSIPATLFVWYFWQKKKQRSGVLWDTPSPPEVERAVLDDGIYRIMQRGLLGRSQVGVGVFQENVFHTMWHVTRGAVLMYQGKRLEPSWASVKKDLISYGGGWRLQGSWNTGEEVQVIAVEPGKNPKNVQTAPGTFKTPEGEVGAIALDFKPGTSGSPIVNREGKIVGLYGNGVVTTSGTYVSAIAQAKASQEGPLPEIEDEVFRKRNLTIMDLHPGSGKTRRYLPAIVREAIKRKLRTLILAPTRVVASEMAEALKGMPIRYQTTAVKSEHTGKEIVDLMCHATFTMRLLSPVRVPNYNMIIMDEAHFTDPSSIAARGYISTRVGMGEAAAIFMTATPPGSVEAFPQSNAVIQDEERDIPERSWNSGYEWITDFPGKTVWFVPSIKSGNDIANCLRKNGKRVIQLSRKTFDTEYQKTKNNDWDYVVTTDISEMGANFRADRVIDPRRCLKPVILKDGPERVILAGPMPVTVASAAQRRGRIGRNHNKEGDQYIYMGQPLNNDEDHAHWTEAKMLLDNINTPEGIIPALFEPEREKSAAIDGEYRLRGEARKTFVELMRRGDLPVWLSYKVASEGFQYSDRRWCFDGERNNQVLEENMDVEIWTKEGERKKLRPRWLDARTYSDPLALREFKEFAAGRRSVSGDLILEIGKLPQHLTQRAQNALDNLVMLHNSEQGGRAYRHAMEELPDTIETLMLLALIAVLTGGVTLFFLSGKGLGKTSIGLLCVMASSVLLWMASVEPHWIAASIILEFFLMVLLIPEPDRQRTPQDNQLAYVVIGLLFMILTVAANEMGLLETTKKDLGIGHVAVENHHHAAMLDVDLHPASAWTLYAVATTIITPMMRHTIENTTANISLTAIANQAAILMGLDKGWPISKMDIGVPLLALGCYSQVNPLTLTAAVLMLVAHYAIIGPGLQAKATREAQKRTAAGIMKNPTVDGIVAIDLDPVVYDAKFEKQLGQIMLLILCTSQILLMRTTWALCESITLATGPLTTLWEGSPGKFWNTTIAVSMANIFRGSYLAGAGLAFSLMKSLGGGRRGTGAQGETLGEKWKRQLNQLSKSEFNTYKRSGIMEVDRSEAKEGLKRGETTKHAVSRGTAKLRWFVERNLVKPEGKVIDLGCGRGGWSYYCAGLKKVTEVKGYTKGGPGHEEPVPMATYGWNLVKLHSGKDVFFIPPEKCDTLLCDIGESSPNPTIEEGRTLRVLKMVEPWLRGNQFCIKILNPYMPSVVETLEQMQRKHGGMLVRNPLSRNSTHEMYWVSCGTGNIVSAINMTSRMLLNRFTMAHRKPTYERDVDLGAGTRHVAVEPEVANLDIIGQRIENIKCEHKSTWHYDEDNPYKTWAYHGSYEVKPSGSASSMVNGVVKLLTKPWDVIPMVTQIAMTDTTPFGQQRVFKEKVDTRTPKAKRGTAQIMEVTARWLWGFLSRNKKPRICTREEFTRKVRSNAAIGAVFVDENQWNSAKEAVEDERFWDLVHRERELHKQGKCATCVYNMMGKREKKLGEFGKAKGSRAIWYMWLGARFLEFEALGFMNEDHWFSRENSLSGVEGEGLHKLGYILRDISKISGGNMYADDTAGWDTRITEDDLQNEAKITEIMEPEHAQLAKAIFKLTYQNKVVRVQRPAKNGTVMDVISRRDQRGSGQVGTYGLNTFTNMEAQLIRQMESEGIFSPSELETPNLAERVLDWLEKYGVERLKRMAISGDDCVVKPIDDRFATALTALNDMGKVRKDIPQWEPSKGWNDWQQVPFCSHHFHQLIMKDGREIVVPCRNQDELVGRARVSQGAGWSLRETACLGKSYAQMWQLMYFHRRDLRLAANAICSAVPVDWVPTSRTTWSIHAHHQWMTTEDMLSVWNRVWIEENPWMKDKTHISSWEDVPYLGKREDQWCGSLIGLTARATWATNIQVAINQVRRLIGNENYLDYMTSMKRFKNESDPEGALW

>DENV1__AUA17938__Taiwan

MNNQRKKTARPSFNMLKRARNRVSTVSQLAKRFSKGLLSGQGPMKLVMAFIAFLRFLAIPPTAGILARWGSFKKNGAIKVLRGFKKEISNMLNIMNRRKRSVTMLLMLMPTALAFHLTTRGGEPHMIVSKQERGKSLLFKTSAGVNMCTLIAMDLGELCEDTMTYKCPRITEAEPDDVDCWCNATDTWVTYGTCSQTGEHRRDKRSVALAPHVGLGLETRTETWMSSEGAWKQIQRVETWALRHPGFTVIALFLAHAIGTSITQKGIIFILLMLVTPSMAMRCVGIGSRDFVEGLSGATWVDVVLEHGSCVTTMAKDKPTLDIELLKTEVTNPAILRKLCIEAKISNTTTDSRCPTQGEATLVEEQDANFVCRRTFVDRGWGNGCGLFGKGSLITCAKFKCVTKLEGKIVQYENLKYSVIVTVHTGDQHQVGNESTEHGTTATITPQAPTTEIQLTDYGALTLDCSPRTGLDFNEMVLLTMKEKSWLVHKQWFLDLPLPWTSGASTSQETWNRQDLLVTFKTAHAKKQEVVVLGSQEGAMHTALTGATEIQTSGTTTIFAGHLKCRLKMDKLTLKGMSYVMCTGSFKLEKEVAETQHGTVLVQIKYEGTDAPCKIPFSTQDEKGVTQNGRLITANPIVTDKEKPVNIEAEPPFGESYIVIGAGEKALKLSWFKKGSSIGKMFEATARGARRMAILGDTAWDFGSIGGVFTSVGKLVHQIFGTAYGVLFSGVSWTMKIGIGVLLTWLGLNSRSTSLSMTCIAVGLVTLYLGVMVQADSGCVINWKGRELKCGSGIFVTNEVHTWTEQYKFQADSPKRLSAAIGKAWEEGVCGIRSATRLENIMWKQISNELNHILLENDMKFTVVVGDVAGILAQGKKMIRPQPMEYKYSWKSWGKAKIIGADVQNTTFIIDGPNTPECPDDQRAWNIWEVEDYGFGIFTTNIWLKLRDSYTQVCDHRLMSAAIKDSKAVHADMGYWIESEKNETWKLARASFIEVKTCIWPKSHTLWSNGVLESEMIIPKIYGGPISQHNYRPGYFTQTAGPWHLGKLELDFDLCEGTTVVVDEHCGNRGPSLRTTTVTGKIIHEWCCRSCTLPPLRFRGEDGCWYGMEIRPVKEKEENLVKSMVSAGSGEVDSFSLGLLCISIMIEEVMRSRWSRKMLMTGTLAVFFLLIMGQLTWNDLIRLCIMVGANASDRMGMGTTYLALMATFKMRPMFAVGLLFRRLTSREVLLLTIGLSLVASVELPNSLEELGDGLAMGIMILKLLTDFQSHQLWATLLSLTFIKTTFSLHYAWKTMAMVLSIVSLFPLCLSTTSQKTTWLPVLLGSLGCKPLTMFLIAENKIWGRKSWPLNEGIMAVGIVSILLSSLLKNDVPLAGPLIAGGMLIACYVISGSSADLSLEKAAEVSWEEEAEHSGASHNILVEVQDDGTMKIKDEERDDTLTILLKATLLAVSGVYPLSIPATLFLWYFWQKKKQRSGVLWDTPSPPEVERAVLDDGIYRIMQRGLLGRSQVGVGVFQENVFHTMWHVTRGAVLMYQGKRLEPSWASVKKDLISYGGGWRLQGSWNTGEEVQVIAVEPGKNPKNVQTAPGTFKTPEGEVGAIALDFKPGTSGSPIVNREGKIVGLYGNGVVTTSGTYVSAIAQAKASQEGPLPEIEDEVFRKRNLTIMDLHPGSGKTRRYLPAIVREAIKRKLRTLILAPTRVVASEMAEALKGMPIRYQTTAVKSEHTGKEIVDLMCHATFTMRLLSPVRVPNYNMIIMDEAHFTDPSSIAARGYISTRVGMGEAAAIFMTATPPGSVEAFPQSNAVIQDEERDIPERSWNSGYEWITDFPGKTVWFVPSIKSGNDIANCLRKNGKRVIQLSRKTFDTEYQKTKNNDWDYVVTTDISEMGANFRADRVIDPRRCLKPVILKDGPERVILAGPMPVTVASAAQRRGRIGRNHNKEGDQYIYMGQPLNNDEDHAHWTEAKMLLDNINTPEGIIPALFEPEREKSAAIDGEYRLRGEARKTFVELMRRGDLPVWLSYKVASEGFQYSDRRWCFDGERNNQVLEENMDVEIWTKEGERKKLRPRWLDARTYSDPLALREFKEFAAGRRSVSGDLILEIGKLPQHLTQRAQNALDNLVMLHNSEQGGRAYRHAMEELPDTIETLMLLALIAVLTGGVTLFFLSGRGLGKTSIGLLCVMASSVLLWMASVEPHWIAASIILEFFLMVLLIPEPDRQRTPQDNQLAYVVIGLLFMILTVAANEMGLLETTKKDLGIGHVAVENHHHAAMLDVDLHPASAWTLYAVATTIITPMMRHTIENTTANISLTAIANQAAILMGLDKGWPISKMDIGVPLLALGCYSQVNPLTLTAAVLMLVAHYAIIGPGLQAKATREAQKRTAAGIMKNPTVDGIVAIDLDPVVYDAKFEKQLGQIMLLILCTSQILLMRTTWALCESITLATGPLTTLWEGSPGKFWNTTIAVSMANIFRGSYLAGAGLAFSLMKSLGGGRRGTGAQGETLGEKWKRQLNQLSKSEFNTYKRSGIMEVDRSEAKEGLKRGETTKHAVSRGTAKLRWFVERNLVKPEGKVIDLGCGRGGWSYYCAGLKKVTEVKGYTKGGPGHEEPIPMATYGWNLVKLHSGKDVFFIPPEKCDTLLCDIGESSPNPTIEEGRTLRVLKMVEPWLRGNQFCIKILNPYMPSVVETLEQMQRKHGGMLVRNPLSRNSTHEMYWVSCGTGNIVSAVNMTSRMLLNRFTMAHRKPTYERDVDLGAGTRHVAVEPEVANLDIIGQRMKNIKHEHKSTWHYDEDNPYKTWAYHGSYEVKPSGSASSMVNGVVKLLTKPWDVIPMVTQIAMTDTTPFGQQRVFKEKVDTRTPKAKRGTAQIMEVTARWLWGFLSRNKKPRICTREEFTRKVRSNAAIGAVFVDENQWNSAKEAVEDERFWDLVHRERELHKQGKCATCVYNMMGKREKKLGEFGKAKGSRAIWYMWLGARFLEFEALGFMNEDHWFSRENSLSGVEGEGLHKLGYILRDISKIPGGNMYADDTAGWDTRITEDDLQNEAKITDIMEPEHALLANSIFKLTYQNKVVRVQRPAKNGTVMDVISRRDQRGSGQVGTYGLNTFTNMEAQLIRQMESEGIFLPSELETPNLAERVLDWLEKYGVERLKRMAISGDDCVVKPIDDRFATALTALNDMGKVRKDIPQWEPSKGWNDWQQVPFCSHHFHQLIMKDGREIVVPCRNQDELVGRARVSQGAGWSLRETACLGKSYAQMWQLMYFHRRDLRLAANAICSAVPVDWVPTSRTTWSIHAHHQWMTTEDMLSVWNRVWIEENPWMEDKTHISSWGDVPYLGKREDQWCGSLIGLTARATWATNIQVAINQVRRLIGNENYLDYMTSMKRFKNESDPEGALW

>DENV1_AHL19966_China

MNNQRKKTARPSFNMLKRARNRVSTVSQLAKRFSKGLLSGQGPMKLVMAFIAFLRFLAIPPTAGILARWGSFKKNGAIKVLRGFKKEISNMLNIMNRRKRSVTMLLMLMPTALAFHLTTRGGEPHMMVSKQERGKSLLFKTSAGVNMCTLIAMDLGELCEDTMTYKCPRITEAEPDDVDCWCNATDTWVTYGTCSQTGEHRRDKRSVALAPHVGLGLETRTETWMSSEGAWKQIQRVETWALRHPGFTVIALFLAHAIGTSITQKGIIFILLMLVTPSMAMRCVGIGSRDFVEGLSGATWVDVVLEHGSCVTTMAKDKPTLDIELLKTEVTNPAVLRKLCIEAKISNTTTDSRCPTQGEATLVEEQDANFVCRRTFVDRGWGNGCGLFGKGSLITCAKFKCVTKLEGKIVQYENLKYSVIVTVHTGDQHQVGNESTEHGTTATITPQAPTTEIQLTDYGALTLDCSPRTGLDFNEMVLLTMKEKSWLVHKQWFLDLPLPWTSGASTSQETWNRQDLLVTFKTAHAKKQEVVVLGSQEGAMHTALTGATEIQTSGTTTIFAGHLKCRLKMDKLTLKGMSYVMCTGSFKLEKEVAETQHGTVLVQIKYEGTDAPCKIPFSTQDEKGVTQNGRLITANPIVTDKEKPVNIEAEPPFGESYIVIGAGEKALKLSWFKKGSSIGKMFEATARGARRMAILGDTAWDFGSIGGVFTSVGKLVHQIFGTAYGVLFSGVSWTMKIGIGVLLTWLGLNSRSTSLSMTCIAVGLVTLYLGVMVQADSGCVINWKGRELKCGSGIFVTNEVHTWTEQYKFQADSPKRLSAAIGKAWEEGVCGIRSATRLENIMWKQISNELNHILLENDMKFTVVVGDVAGILAQGKKMIRPQPMEYKYSWKSWGKAKIIGADVQNTTFIIDGPNTPECPDDQRAWNIWEVEDYGFGIFTTNIWLKLRDSYTQVCDHRLMSAAIKDSKAVHADMGYWIESEKNDTWKLARASFIEVKTCIWPKSHTLWSNGVLESEMIIPKIYGGPISQHNYRPGYFTQTAGPWHLGKLELDFDLCEGTTVVVDEHCGNRGPSLRTTTVTGKIIHEWCCRSCTLPPLRFRGEDGCWYGMEIRPVKEKEENLVKSMVSAGSGEVDSFSLGLLCISIMIEEVMRSRWSRKMLMIGTLAVFFLLIMGQLTWNDLIRLCIMVGANASDRMGMGTTYLALMATFKMRPMFAVGLLFRRLTSREVLLLTIGLSLVASAELPNSLEELGDGLAMGIMILKLLTDFQSHQLWATLLSLTFIKTTFSLHYAWKTMAMVLSIVSLFPLCLSTTSQKTTWLPVLLGSLGCKPLTMFLIAENKIWGRKSWPLNEGIMAVGIVSILLSSLLKNDVPLAGPLIAGGMLIACYVISGSSADLSLEKAAEVSWEEEAEHSGASHNILVEVQDDGTMKIKDEERDDTLTILLKATLLAVSGVFPLSIPATLFVWYFWQKKKQRSGVLWDTPSPPEVERAVLDDGIYRIMQRGLLGRSQVGVGVFQENVFHTMWHVTRGAVLMYQGKRLEPSWASVKKDLISYGGGWRLQGSWNTGEEVQVIAVEPGKNPKNVQTAPGTFKTPEGEVGAIALDFKPGTSGSPIVNREGKIVGLYGNGVVTTSGTYVSAIAQAKASQEGPLPEIEDEVFRKRNLTIMDLHPGSGKTRRYLPAIVREAIKRKLRTLILAPTRVVASEMAEALKGMPIRYQTTAVKSEHTGKEIVDLMCHATFTMRLLSPVRVPNYNMIIMDEAHFTDPSSIAARGYISTRVGMGEAAAIFMTATPPGSVEAFPQSNAVIQDEERDIPERSWNSGYEWITDFPGKTVWFVPSIKSGNDIANCLRKNGKRVIQLSRKTFDTEYQKTKNNDWDYVVTTDISEMGANFRADRVIDPRRCLKPVILKDGPERVILAGPMPVTVASAAQRRGRIGRNHNKEGDQYIYMGQPLNNDEDHAHWTEAKMLLDNINTPEGIIPALFEPEREKSAAIDGEYRLRGEARKTFVELMRRGDLPVWLSYKVASEGFQYSDRRWCFDGERNNQVLEENMDVEIWTKEGERKKLRPRWLGARTYSDPLALREFKEFAAGRRSVSGDLILEIGKLPQHLTQRAQNALDNLVMLHNSEQGGRAYRHAMEELPDTIETLMLLALIAVLTGGVTLFFLSGRGLGKTSIGLLCVMASSVLLWMASVEPHWIAASIILEFFLMVLLIPEPDRQRTPQDNQLAYVVIGLLFMILTVAANEMGLLETTKKDLGIGHVAVENHHHAAMLDVDLHPASAWTLYAVATTIITPMMRHTIENTTANISLTAIANQAAILMGLDKGWPISKMDIGVPLLALGCYSQVNPLTLTAAVLMLVAHYAIIGPGLQAKATREAQKRTAAGIMKNPTVDGIVAIDLDPVVYDAKFEKQLGQIMLLILCTSQILLMRTTWALCESITLATGPLTTLWEGSPGKFWNTTIAVSMANIFRGSYLAGAGLAFSLMKSLGGGRRGTGAQGETLGEKWKRRLNQLSKSEFNTYKRSGIMEVDRSEAKEGLKRGETTKHAVSRGTAKLRWFVERNLVKPEGKVIDLGCGRGGWSYYCAGLKKVTEVKGYTKGGPGHEEPIPMATYGWNLVKLHSGKDVFFIPPEKCDTLLCDIGESSPNPTIEEGRTLRVLKMVEPWLRGNQFCIKILNPYMPSVVETLEQMQRKHGGMLVRNPLSRNSTHEMYWVSCGTGNIVSAVNMTSRMLLNRFTMAHRKPTYERDVDLGAGTRHVAVEPEVANLDIIGQRIENIKHEHKSTWHYDEDNPYKTWAYHGSYEVKPSGSASSMVNGVVKLLTKPWDVIPMVTQIAMTDTTPFGQQRVFKEKVDTRTPKAKRGTAQIMEVTARWLWGFLSRNKKPRICTREEFTRKVRSNAAIGAVFIDENQWNSAKEAVEDERFWDLVHRERELHKQGKCATCVYNMMGKREKKLGEFGKAKGSRAIWYMWLGARFLEFEALGFMNEDHWFSRENSLSGVEGEGLHKLGYILRDISKIPGGNMYADDTAGWDTRITEDDLQNEAKITDIMEPEHALLAKAIFKLTYQNKVVRVQRPAKNGTVMDVISRRDQRGSGQVGTYGLNTFTNMEAQLIRQMESEGIFSPSELETPNLAERVLDWLGKYGVERLKRMAISGDDCVVKPIDDRFATALTALNDMGKVRKDIPQWEPSKGWNDWQQVPFCSHHFHQLIMKDGREIVVPCRNQDELVGRARVSQGAGWSLRETACLGKSYAQMWQLMYFHRRDLRLAANAICSAVPVDWVPTSRTTWSIHAHHQWMTTEDMLSVWNRVWIEENPWMEDKTHISNWEDVPYLGKREDQWCGSLIGLTARATWATNIQVAINQVRRLIGNENYLDYMTSMKRFKNESDPEGALW

>DENV1_ATJ00092_Ecuador

MNNQRKKTGRPSFNMLKRARNRVSTGSQLAKRFSKGLLSGQGPMKLVMAFIAFLRFLAIPPTAGILARWSSFKKNGAIKVLRGFKKEISSMLNIMNRRKRSVTMLLMLLPTALAFHLTTRGGEPHMIVSKQERGKSLLFKTSAGVNMCTLIAMDLGELCEDTMTYKCPRITEAEPDDVDCWCNATDTWVTYGTCSQTGEHRRDKRSVALAPHVGLGLETRTETWMSSEGAWKQIQRVETWALRHPGFTVIALFLAHAIGTSITQKGIIFILLMLVTPSMAMRCVGIGNRDFVEGLSGATWVDVVLEHGSCVTTMAKNKPTLDIELLKTEVTNPAVLRKLCIEAKISNTTTDSRCPTQGEATLVEEQDANFVCRRTFVDRGWGNGCGLFGKGSLLTCAKFKCVTKLEGKIVQYENLKYSVIVTVHTGDQHQVGNETTEHGTIATITPQAPTSEIQLTDYGALTLDCSPRTGLDFNEMVLLTMKEKSWLVHKQWFLDLPLPWTSGATTSQETWNRQDLLVTFKTAHAKKQEVVVLGSQEGAMHTALTGATEIQTSGTTTIFAGHLKCRLKMDKLTLKGMSYVMCTGSFKLEKEVAETQHGTVLVQVKYEGTDAPCKIPFLTQDEKGVTQNGRLITANPIVTDKEKPVNIETEPPFGESYIVVGAGEKALKLSWFKKGSSIGKMFEATARGARRMAILGDTAWDFGSIGGVFTSVGKLVHQVFGTAYGVLFSGVSWTMKIGIGILLTWLGLNSRSTSLSMTCIAVGMVTLYLGVMVQADSGCVINWKGRELKCGSGIFVTNEVHTWTEQYKFQADSPKRLSAAIGKAWEEGVCGIRSVTRLENIMWKQISNELNHILLENDMKLTVVVGDANGILAQGKKMIRPQPMEHKYSWKSWGKAKIIGADTQNTTFIIDGPDTPECPDGQRAWNIWEVEDYGFGIFTTNIWLKLRDSYTQMCDHRLMSAAIKDSKAVHADMGYWIESEKNETWKLARASFIEVKTCTWPKSHTLWSNGVLESEMIIPKIYGGPISQHNYRPGYFTQTAGPWHLGKLELDFDLCEGTTVVVDEHCGNRGPSLRTTTVTGKIIHEWCCRSCTLPPLRFRGEDGCWYGMEIRPVKEKEENLVRSMVSAGSGEVDSFSLGILCVSIMIEEVMRSRWSRKMLMTGTLAVFLLLIMGQLTWNDLIRLCIMVGANASDRMGMGTTYLALMATFKMRPMFAVGLLFRRLTSREVLLLTIGLSLVASVELPNSLEELGDGLAMGIMMLKLLTEFQPHQLWTTLLSLTFVKTTLSLDYAWKTTAMVLSIVSLFPLCLSTTSQKTTWLPVLLGSFGCKPLTMFLITENKIWGRKSWPLNEGIMAIGIVSILLSSLLKNDVPLAGPLIAGGMLIACYVISGSSADLSLEKAAEVSWEQEAEHSGASHNILVEVQDDGTMKIKDEERDDTLTILLKATLLAVSGVYPMSIPATLFVWYFWQKKKQRSGVLWDTPSPPEVEKAVLDDGIYRILQRGLLGRSQVGVGVFQDGVFHTMWHVTRGAVLMYQGKRLEPSWASVKKDLISYGGGWRFQGSWNTGEEVQVIAVEPGKNPKNVQTTPGTFKTPEGEVGAIALDFKPGTSGSPIVNREGKIVGLYGNGVVTTSGTYVSAIAQAKASQEGPLPEIEDEVFKKRNLTIMDLHPGSGKTRRYLPAIVREAIKRKLRTLILAPTRVVASEMAEALKGMPIRYQTTAVKSEHTGREIVDLMCHATFTMRLLSPVRVPNYNMIIMDEAHFTDPASIAARGYISTRVGMGEAAAIFMTATPPGSVEAFPQSNAVIQDEERDIPERSWNSGYDWITDFPGKTVWFVPSIKSGNDIANCLRKNGKRVIQLSRKTFDTEYQKTKNNDWDYVVTTDISEMGANFRADRVIDPRRCLKPVILKDGPERVILAGPMPVTVASAAQRRGRIGRNQNKEGDQYVYMGQPLNNDEDHAHWTEAKMLLDNINTPEGIIPALFEPEREKSAAIDGEYRLRGEARKTFVELMRRGDLPVWLSYKVASEGFQYSDRRWCFDGERNNQVLEENMDVEIWTKEGERKKLRPRWLDARTYSDPLALREFKEFAAGRRSVSGDLILEIGKLPQHLTLRAQNALDNLVMLHNSEQGGKAYRHAMEELPDTIETLMLLALIAVLTGGVTLFFLSGKGLGKTSIGLLCVTASSALLWMAGVEPHWIAASIILEFFLMVLLIPEPDRQRTPQDNQLAYVVIGLLFMILTVAANEMGLLETTKKDLGIGYVATENHQHATMLDVDLRPASAWTLYAVATTVITPMMRHTIENTTANISLTAIANQAAILMGLDKGWPISKMDIGVPLLALGCYSQVNPLTLTAAVLMLVAHYAIIGPGLQAKATREAQKRTAAGIMKNPTVDGIVAIDLDPVVYDAKFEKQLGQIMLLILCTSQILLMRTTWALCESITLATGPLTTLWEGSPGKFWNTTIAVSMANIFRGSYLAGAGLAFSLMKSLGGGRRGTGAQGETLGEKWKRQLNQLSKSEFNIYKRSGIMEVDRSEAKEGLKRGETTKHAVSRGTAKLRWFVERNLVKPEGKVIDLGCGRGGWSYYCAGLKKVTEVKGYTKGGPGHEEPIPMATYGWNLVKLHSGKDVFFMPPEKCDTLLCDIGESSPNPTIEEGRTLRVLKMVEPWLRGNQFCIKILNPYMPSVVETLEQMQRKHGGMLVRNPLSRNSTHEMYWVSCGTGNIVSAVNMTSRMLLNRFTMAHRKPTYERDVDLGAGTRHVAVEPEVANLDIIGQRIENIKNEHKSTWHYDEDNPYKTWAYHGSYEVKPSGSASSMVNGVVRLLTKPWDVIPMVTQIAMTDTTPFGQQRVFKEKVDTRTPRAKRGTTQIMEVTAKWLWGFLSRNKKPRICTREEFTRKVRSNAAIGAVFVDENQWNSAKEAVEDERFWDLVHRERELHKQGKCATCVYNMMGKREKKLGEFGKAKGSRAIWYMWLGARFLEFEALGFMNEDHWFSRENSLSGVEGEGLHKLGYILRDISKIPGGNMYADDTAGWDTRITEDDLQNEAKITDIMEPEHALLATSIFKLTYQNKVVRVQRPAKNGTVMDVISRRDQRGSGQVGTYGLNTFTNMEVQLIRQMESEGIFLPSELETPNLAERVLDWLEKHGAERLKRMAISGDDCVVKPIDDRFAAALTALNDMGKVRKDIPQWEPSKGWNDWQQVPFCSHHFHQLIMKDGREIVVPCRNQDELVGRARVSQGAGWSLRETACLGKSYAQMWQLMYFHRRDLRLAANAICSAVPVDWVPTSRTTWSIHAHHQWMTTEDMLSVWNRVWIEENPWMEDKTHVSSWEEVPYLGKREDQWCGSLIGLTARATWATNIQVAINQVRRLIGNENYLDYMTSMKRFKNESDPEGALW

>DENV1_ARQ80406__Saint_Barthelemy

MNNQRKKTGRPSFNMLKRARNRVSTGSQLAKRFSKGLLSGQGPMKLVMAFIAFLRFLAIPPTAGILARWSSFKKNGAIKVLRGFKKEISSMLNIMNRRKRSVTMLLMLLPTALAFHLTTRGGEPHMIVSKQERGKSLLFKTSAGVNMCTLIAMDLGELCEDTMTYKCPQITEAEPDDVDCWCNATDTWVTYGTCSQTGEHRRDKRSVALAPHVGLGLETRTETWMSSEGAWKQIQRVETWALRHPGFTVIALFLAHAIGTSITQKGIIFILLMLVTPSMAMRCVGIGNRDFVEGLSGATWVDVVLEHGSCVTTMAKNKPTLDIELLKTEVTNPAILRKLCIEAKISNTTTDSRCPTQGEATLVEEQDANFVCRRTFVDRGWGNGCGLFGKGSLLTCAKFKCVTKLEGKIVQYENLKYSVIVTVHTGDQHQVGNETTEHGTIATITPQAPTSEIQLTDYGALTLDCSPRTGLDFNEMVLLTMKEKSWLVHKQWFLDLPLPWTSGASTSQETWNRQDLLVTFKTAHAKKQEVVVLGSQEGAMHTALTGATEIQTSGTTTIFAGHLKCRLKMDKLTLKGTSYVMCTGSFKLEKEVAETQHGTVLVQVKYEGTDAPCKIPFSTQDEKGVTQNGRLITANPIVTDKEKPVNIETEPPFGESYIVVGAGEKALKLSWFKKGSSIGKMFEATARGARRMAILGDTAWDFGSIGGVFTSVGKLVHQVFGTAYGVLFSGVSWTMKIGIGILLTWLGLNSRSTSLSMTCIAVGMVTLYLGVMVQADSGCVINWKGRELKCGSGIFVTNEVHTWTEQYKFQADSPKRLSAAIGKAWEEGVCGIRSATRLENIMWKQISNELNHILLENDMKFTVVVGDATGILAQGKKMIRPQPMEHKYSWKSWGKAKIIGADIQNTTFIIDGPDTPECPDNQRAWNIWEVEDYGFGIFTTNIWLKLRDSYTQMCDHRLMSAAIKDSKAVHADMGYWIESEKNETWKLARASFIEVKTCTWPKSHTLWSNGVLESEMIIPKIYGGPISQHNYRPGYFTQTAGPWHLGKLELDFDLCEGTTVVVDEHCGNRGPSLRTTTVTGKIIHEWCCRSCTLPPLRFRGEDGCWYGMEIRPVKEKEENLVRSMVSAGSGEVDSFSLGILCASIMIEEVMRSRWSRKMLMTGTLAVFLLLIMGQLTWNDLIRLCIMVGANASDRMGMGTTYLALMATFKMRPMFAVGLLFRRLTSREVLLLTIGLSLVASVELPNSLEELGDGLAMGIMMLKLLTEFQPHQLWTTLLSLTFIKTTLSLDYAWKTTAMALSIVSLFPLCLSTTSQKTTWLPVLLGSFGCKPLTMFLITENKIWGRKSWPLNEGIMAIGIVSILLSSLLKNDVPLAGPLIAGGMLIACYVISGSSADLSLEKAAEVSWEQEAEHSGTSHNILVEVQDDGTMKIKDEERDDTLTILLKATLLAVSGVYPMSIPATLFVWYFWQKKKQRSGVLWDTPSPPEVERAVLDDGIYRILQRGLLGRSQVGVGVFQDGVFHTMWHVTRGAVLMYQGKRLEPSWASIKKDLISYGGGWRFQGSWNTGEEVQVIAIEPGKNPKNVQTTPGTFKTPEGEVGAIALDFKPGTSGSPIVNREGKIVGLYGNGVVTTSGTYVSAIAQAKASQEGPLPEIEDEVFKKRNLTIMDLHPGSGKTRRYLPAIVREAIKRKLRTLILAPTRVVASEMAEALKGMPIRYQTTAVKSEHTGREIVDLMCHATFTMRLLSPVRVPNYNMIIMDEAHFTDPASIAARGYISTRVGMGEAAAIFMTATPPGSVEAFPQSNAVIQDEERDIPERSWNSGYDWITDFPGKTVWFVPSIKSGNDIANCLRKNGKRVIQLSRKTFDTEYQKTKNNDWDYVVTTDISEMGANFRADRVIDPRRCLKPVILKDGPERVILAGPMPVTVASAAQRRGRIGRNQNKEGDQYVYMGQPLNNDEDHAHWTEAKMLLDNINTPEGIIPALFEPEREKSAAIDGEYRLRGEARKTFVELMRRGDLPVWLSYKVASEGFQYSDRRWCFDGERNNQVLEENMDVEIWTKEGERKKLRPRWLDARTYSDPLALREFKEFAAGRRSVSGDLILEIGKLPQHLTLRAQNALDNLVMLHNSEQGGKAYRHAMEELPDTIETLMLLALIAVLTGGVTLFFLSGKGLGKTSIGLLCVTASSALLWMASVEPHWIAASIILEFFLMVLLIPEPDRQRTPQDNQLAYVVIGLLFMILTVAANEMGLLETTKKDLGIGHVAAENHQHATMLDVDLRPASAWTLYAVATTVITPMMRHTIENTTANISLTAIANQAAILMGLDKGWPISKMDIGVPLLALGCYSQVNPLTLTAAVLMLVAHYAIIGPGLQAKATREAQKRTAAGIMKNPTVDGIVAIDLDPVVYDTKFEKQLGQIMLLILCTSQILLMRTTWALCESITLATGPLTTLWEGSPGKFWNTTIAVSMANIFRGSYLAGAGLAFSLMKSLGGGRRGTGAQGETLGEKWKRQLNQLSKSEFNIYKRSGIMEVDRSEAKEGLKRGETTKHAVSRGTAKLRWFVERNLVKPEGKVIDLGCGRGGWSYYCAGLKKVTEVKGYTKGGPGHEEPIPMATYGWNLVKLHSGKDVFFMPPEKCDTLLCDIGESSPNPTIEEGRTLRVLKMVEPWLRGNQFCIKILNPYMPSVVETLEQMQRKYGGMLVRNPLSRNSTHEMYWVSCGTGNIVSAVNMTSRMLLNRFTMAHRKPTYERDVDLGAGTRHVAVEPEVANLDIIGQRIENIKNEHKSTWHYDEDNPYKTWAYHGSYEVKPSGSASSMVNGVVRLLTKPWDVISMVTQIAMTDTTPFGQQRVFKEKVDTRTPRAKRGTTQIMEVTAKWLWGFLSRNKKPRICTREEFTRKVRSNAAIGAVFVDENQWNSAKEAVEDERFWDLVHRERELHKQGKCATCVYNMMGKREKKLGEFGKAKGSRAIWYMWLGARFLEFEALGFMNEDHWFSRENSLSGVEGEGLHKLGYILRDISKIPGGNMYADDTAGWDTRITEDDLQNEAKITDIMEPEHALLATSIFKLTYQNKVVRVQRPAKNGTVMDVISRRDQRGSGQVGTYGLNTFTNMEVQLIRQMESEGIFLPSELETPNLAERVLDWLEKHGAERLKRMAISGDDCVVKPIDDRFATALTALNDMGKVRKDIPQWEPSKGWNDWQQVPFCSHHFHQLIMKDGREIVVPCRNQDELVGRARVSQGAGWSLRETACLGKSYAQMWQLMYFHRRDLRLAANAICSAVPVDWVPTSRTTWSIHAHHQWMTTEDMLSVWNRVWIEENPWMEDKTHVSSWEEVPYLGKREDQWCGSLIGLTARATWATNIQVAINQVRRLIGNENYLDYMTSMKRFKNESDPEGALW

>DENV1_ARM59244_Singapore

MNNQRKKTGRPSFNMLKRARNRVSTGSQLAKRFSKGLLSGQGPMKMVMAFIAFLRFLAIPPTAGILARWSSFKKNGAIKVLRGFKKEISSMLNIMNRRKRSVTMLLMLLPTALAFHLTTRGGEPHMIVSKQERGKSLLFKTSAGVNMCTLIAMDLGELCEDTMTYKCPRITEAEPDDVDCWCNATDTWVTYGTCSQTGEHRRDKRSVALAPHVGLGLETRTETWMSSEGAWRQIQKVETWALRHPGFTVIALFLAHAIGTSITQKGIIFILLMLVTPSMAMRCVGIGNRDFVEGLSGATWVDVVLEHGSCVTTMAKNKPTLDIELLKTEVTNPAVLRKLCIEAKISNTTTDSRCPTQGEATLVEEQDANFVCRRTFVDRGWGNGCGLFGKGSLLTCAKFKCVTKLEGKIVQYENLKYSVIVTVHTGDQHQVGNETTEHGTIATITPQAPTSEIQLTDYGALTLDCSPRTGLDFNEMVLLTMKEKSWLVHKQWFLDLPLPWTSGASTSQETWNRQDLLVTFKTAHAKKQEVVVLGSQEGAMHTALTGATEIQTSGTTTIFAGHLKCRLKMDKLTLKGVSYVMCTGSFKLEKEVAETQHGTVLVQVKYEGTDAPCKIPISTQDEKGVTQNGRLITANPIVTDKEKPVNIETEPPFGESYIVIGAGEKALKLSWFKKGSSIGKMFEATARGARRMAILGDTAWDFGSIGGVFTSVGKLVHQVFGTAYGVLFSGVSWTMKIGIGILLTWLGLNSRSTSLSMTCIAVGMVTLYLGVMVQADSGCVINWKGRELKCGSGIFVTNEVHTWTEQYKFQADSPKRLSAAIGKAWEEGVCGIRSATRLENIMWKQISNELNHILLENDMKFTVVVGDANGILTQGKKMIRPQPMEHKYSWKSWGKAKIIGADTQNTTFIIDGPDTPECPDDQRAWNIWEVEDYGFGVFTTNIWLKLRDSYTQMCDHRLMSAAIKDSKAVHADMGYWIESEKNETWKLARASFIEVKTCIWPRSHTLWSNGVLESEMIIPKIYGGPISQHNYRPGYFTQTAGPWHLGKLELDFNLCEGTTVVVDEHCGNRGPSLRTTTVTGKIIHEWCCRSCTLPPLRFRGEDGCWYGMEIRPVKEKEENLVRSMVSAGSGEVDSFSLGLLCVSIMIEEVMRSRWSRKMLMTGTLAVFFLLIMGQLTWNDLIRLCIMVGANVSDRMGMGTTYLALMATFKMRPMFAVGLLFRRLTSREVLLLTIGLSLVASVELPNSLEELGDGLAMGIMMLKLLTDFQSHQLWTTLLSLTFIKTTLSLDYAWKTMAMVLSIVSLFPLCLSTTSQKTTWLPVLLGSFGCKPLTMFLITENKIWGRKSWPLNEGIMAVGIVSILLSSLLKNDVPLAGPLIAGGMLIACYVISGSSADLSLERAAEVSWEEEAEHSGASHNILVEVQDDGTMKIKDEERDDTLTILLKATLLAVSGVYPMSIPATLFVWYFWQKKKQRSGVLWDTPSPPEVERAVLDDGIYRILQRGLLGRSQVGVGVFQDGVFHTMWHVTRGAVLMYQGKRLEPSWASVKKDLISYGGGWRFQGSWNTGEEVQVIAVEPGKNPKNVQTTPGTFKTPEGEVGAIALDFKPGTSGSPIVNREGKIVGLYGNGVVTTSGTYVSAIAQAKASQEGPLPEIEDEVFKKRNLTIMDLHPGSGKTRRYLPAIVREAIKRKLRTLILAPTRVVASEMAEALKGMPIRYQTTAVKSEHTGKEIVDLMCHATFTMRLLSPVRVPNYNMIIMDEAHFTDPASIAARGYISTRVGMGEAAAIFMTATPPGSVESFPQSNAVIQDEERDIPERSWNSGYDWITDFPGKTVWFVPSIKSGNDIANCLRKNGKRVIQLSRKTFDTEYQKTKNNDWDYVVTTDISEMGANFRADRVIDPRRCLKPVILKDGPERVILAGPMPVTAASAAQRRGRIGRNQNKEGDQYIYMGQPLNNDEDHAHWTEAKMLLDNINTPEGIIPALFEPEREKSAAIDGEYRLRGEARKTFVELMRRGDLPVWLSYKVASEGFQYSDRRWCFDGERNNQVLEENMDVEIWTKEGERKKLRPRWLDARTYSDPLALREFKEFAAGRRSVSGDLILEIGKLPQHLTLRAQNALDNLVMLHNSEQGGKAYRHAMEELPDTIETLMLLALIAVLTGGVTLFFLSGKGLGKTSIGLLCVMASSVLLWMASVEPHWIAASIILEFFLMVLLIPEPDRQRTPQDNQLAYVVIGLLFMILTVAANEMGLLETTKKDLGIGHVVAENHHHATMLDIDLHPASAWTLYAVATTIITPMMRHTIENTTANISLTAIANQAAILMGLDKGWPISKMDIGVPLLALGCYSQVNPLTLTAAVLMLVAHYAIIGPGLQAKATREAQKRTAAGIMKNPTVDGIVAIDLDPVVYDAKFEKQLGQIMLLILCTSQILLMRTTWALCESITLATGPLTTLWEGSPGKFWNTTIAVSMANIFRGSYLAGAGLAFSLMKSLGGGRRGTGAQGETLGEKWKRQLNQLSKSEFNTYKRSGIIEVDRSEAKEGLKRGETTKHAVSRGTAKLRWFVERNLVKPEGKVIDLGCGRGGWSYYCAGLKKVTEVKGYTKGGPGHEEPIPMATYGWNIVKLHSGRDVFFTPPEKCDTLLCDIGESSPNPTIEEGRTLRVLKMVEPWLRGNQFCIKILNPYMPSVVETLEQMQRKHGGMLVRNPLSRNSTHEMYWVSCGTGNIVSAVNMTSRMLLNRFTMAHRKPTYERDVDLGAGTRHVAVEPEIANLDIIGQRIENIKNEHKSTWHYDEDNPYKTWAYHGSYEVKPSGSASSMVNGVVRLLTKPWDVIPMVTQIAMTDTTPFGQQRVFKEKVDTRTPRPKRGTAQIMEVTAKWLWGFLSRNKKPRICTREEFTRKVRSNAAIGAVFVDENQWNSAKEAVEDERFWDLVHRERELHKQGKCATCVYNMMGKREKKLGEFGKAKGSRAIWYMWLGARFLEFEALGFMNEDHWFSRENSLSGVEGEGLHKLGYILRDISKIPGGNMYADDTAGWDTRITEDDLQNEAKITDIMEPEHALLATSIFKLTYQNKVVRVQRPAKSGTVMDVISRRDQRGSGQVGTYGLNTFTNMEVQLIRQMESEGIFSPSELENSNLAERVLDWLEKHGVERLKRMAISGDDCVVKPTDDRFATALTALNDMGKVRKDIPQWEPSKGWNDWQQVPFCSHHFHQLIMKDGREIVVPCRNQDELVGRARVSQGAGWSLRETACLGKSYAQMWQLMYFHRRDLRLAANAICSAVPVNWVPTSRTTWSIHAHHQWMTTEDMLSVWNRVWIEENPWMEDKTHVSSWEDVPYLGKREDQWCGSLIGLTARATWATNIQVAINQVRRLIGNENYLDYMTSMKRFKNESDPEGALW

>DENV1_BAQ08293_Japan

MNNQRKKTARPSFNMLKRARNRVSTVSQLAKRFSKGLLSGQGPMKLVMAFIAFLRFLAIPPTAGILARWGSFKKNGAIKVLRGFKKEISNMLNIMNRRKRSVTMLLMLMPTALAFHLTTRGGEPHMIVSKQERGKSLLFKTSAGVNMCTLIAMDLGELCEDTMTYKCPRITEAEPDDVDCWCNATDTWVTYGTCSQTGEHRRDKRSVALAPHVGLGLETRTETWMSSEGAWKQIQRVETWALRHPGFTVIALFLAHAIGTSITQKGIIFILLMLVTPSMAMRCVGIGSRDFVEGLSGATWVDVVLEHGSCVTTMAKDKPTLDIELLKTEVTNPAVLRKLCIEAKISNTTTDSRCPTQGEATLVEEQDANFVCRRTFVDRGWGNGCGLFGKGSLITCAKFKCVTKLEGKIVQYENLKYSVIVTVHTGDQHQVGNESTEHGTTATITPQAPTTEIQLTDYGALTLDCSPRTGLDFNEMVLLTMKEKSWLVHKQWFLDLPLPWTSGASTSQETWNRQDLLVTFKTAHAKKQEVVVLGSQEGAMHTALTGATEIQTSGTTTIFAGHLKCRLKMDKLTLKGMSYVMCTGSFKLEKEVAETQHGTVLVQIKYEGTDAPCKIPFSTQDEKGVTQNGRLITANPIVTDKEKPVNIEAEPPFGESYIVIGAGEKALKLSWFKKGSSIGKMFEATARGARRMAILGDTAWDFGSIGGVFTSVGKLVHQIFGTAYGVLFSGVSWTMKIGIGVLLTWLGLNSRSTSLSMTCIAVGLVTLYLGVMVQADSGCVINWKGRELKCGSGIFVTNEVHTWTEQYKFQADSPKRLSAAIGKAWEEGVCGIRSATRLENIMWKQISNELNHILLENDMKFTVVVGDVAGILAQGKKMIRPQPMEHKYSWKSWGKAKIIGADVQNTTFIIDGPNTPECPDDQRAWNIWEVEDYGFGIFTTNIWLKLRDSYTQVCDHRLMSAAIKDSKAVHADMGYWIESEKNETWKLARASFIEVKTCIWPKSHTLWSNGVLESEMIIPKIYGGPISQHNYRPGYFTQTAGPWHLGKLELDFDLCEGTTVVVDEHCGNRGPSLRTTTVTGKIIHEWCCRSCTLPPLRYRGEDGCWYGMEIRPVKEKEENLVKSMVSAGSGEVDSFSLGLLCISIMIEEVMRSRWSRKMLMTGTLAVFFLLIMGQLTWNDLIRLCIMVGANASDRMGMGTTYLALMATFKMRPMFAVGLLFRRLTSREVLLLTIGLSLVASVELPNSLEELGDGLAMGIMILKLLTDFQSHQLWATLLSLTFIKTTFSLHYAWKTIAMVLSIVSLFPLCLSTTSQKTTWLPVLLGSLGCKPLTMFLIAENKIWGRKSWPLNEGIMAVGIVSILLSSLLKNDVPLAGPLIAGGMLIACYVISGSSADLSLEKAAEVSWEEEAEHSGASHNILVEVQDDGTMKIKDEERDDTLTILLKATLLAVSGVYPLSIPATLFVWYFWQKKKQRSGVLWDTPSPPEVERAVLDDGIYRIMQRGLLGRSQVGVGVFQENVFHTMWHVTRGAVLMYQGKRLEPSWASVKKDLISYGGGWRLQGSWNTGEEVQVIAVEPGKNPKNVQTAPGTFKTPEGEVGAIALDFKPGTSGSPIVNREGKIVGLYGNGVVTTSGTYVSAIAQAKASQEGPLPEIEDEVFRKRNLTIMDLHPGSGKTRRYLPAIVREAIKRKLRTLILAPTRVVASEMAEALKGMPIRYQTTAVKSEHTGKEIVDLMCHATFTMRLLSPVRVPNYNMIIMDEAHFTDPSSIAARGYISTRVGMGEAAAIFMTATPPGSVEAFPQSNAVIQDEERDIPERSWNSGYEWITDFPGKTVWFVPSIKSGNDIANCLRKNGKRVIQLSRKTFDTEYQKTKNNDWDYVVTTDISEMGANFRADRVIDPRRCLKPVILKDGPERVILAGPMPVTVASAAQRRGRIGRNHNKEGDQYIYMGQPLNNDEDHAHWTEAKMLLDNINTPEGIIPALFEPEREKSAAIDGEYRLRGEARKTFVELMRRGDLPVWLSYKVASEGFQYSDRRWCFDGERNNQVLEENMDVEIWTKEGERKKLRPRWLDARTYSDPLALREFKEFAAGRRSVSGDLILEIGKLPQHLTQRAQNALDNLVMLHNSEQGGRAYRHAMEELPDTIETLMLLALIAVLTGGVTLFFLSGKGLGKTSIGLLCVMASSVLLWMASVEPHWIAASIILEFFLMVLLIPEPDRQRTPQDNQLAYVVIGLLFMILTVAANEMGLLETTKKDLGIGHVAVENHHHAAMLDVDLHPASAWTLYAVATTIITPMMRHTIENTTANISLTAIANQAAILMGLDKGWPISKMDIGVPLLALGCYSQVNPLTLTAAVLMLVAHYAIIGPGLQAKATREAQKRTAAGIMKNPTVDGIVAIDLDPVVYDAKFEKQLGQIMLLILCTSQILLMRTTWALCESITLATGPLTTLWEGSPGKFWNTTIAVSMANIFRGSYLAGAGLAFSLMKSLGGGRRGTGAQGETLGEKWKRQLNQLSKSEFNTYKRSGIMEVDRSEAKEGLKRGETTKHAVSRGTAKLRWFVERNLVKPEGKVIDLGCGRGGWSYYCAGLKKVTEVKGYTKGGPGHEEPVPMATYGWNLVKLHSGKDVFFIPPEKCDTLLCDIGESSPNPTIEEGRTLRVLKMVEPWLRGNQFCIKILNPYMPSVVETLEQMQRKHGGMLVRNPLSRNSTHEMYWVSCGTGNIVSAINMTSRMLLNRFTMAHRKPTYERDVDLGAGTRHVAVEPEVANLDIIGQRIENIKCEHKSTWHYDEDNPYKTWAYHGSYEVKPSGSASSMVNGVVKLLTKPWDVIPMVTQIAMTDTTPFGQQRVFKEKVDTRTPKAKRGTAQIMEVTARWLWGFLSRNKKPRICTREEFTRKVRSNAAIGAVFVDENQWNSAKEAVEDERFWDLVHRERELHKQGKCATCVYNMMGKREKKLGEFGKAKGSRAIWYMWLGARFLEFEALGFMNEDHWFSRENSLSGVEGEGLHKLGYILRDISKISGGNMYADDTAGWDTRITEDDLQNEAKITEIMEPEHAQLAKAIFKLTYQNKVVRVQRPAKNGTVMDVISRRDQRGSGQVGTYGLNTFTNMEAQLIRQMESEGIFSPSELETPNLAERVLDWLEKYGVERLKRMAISGDDCVVKPIDDRFATALTALNDMGKVRKDIPQWEPSKGWNDWQQVPFCSHHFHQLIMKDGREIVVPCRNQDELVGRARVSQGAGWSLRETACLGKSYAQMWQLMYFHRRDLRLAANAICSAVPVDWVPTSRTTWSIHAHHQWMTTEDMLSVWNRVWIEENPWMKDKTHISSWEDVPYLGKREDQWCGSLIGLTARATWATNIQVAINQVRRLIGNENYLDYMTSMKRFKNESDPEGALW

>DENV1_AJP08954_India

MNNQRKKTARPSFNMLKRARNRVSTVSQLAKRFSKGLLSGQGPMKLVMAFIAFLRFLAIPPTAGILARWGSFKKNGAIKVLRGFKKEISNMLNIMNRRRKSVTMLLMLMPTALAFHLTTRGGEPHMIVSKQERGKSLLFKTSAGVNMCTLIAMDLGELCEDTMTYKCPRITEAEPDDVDCWCNATDTWVTYGTCSQTGEHRRDKRSVALAPHVGLGLETRAETWMSSEGAWKQIQRVETWALRHPGFTVIALFLAHAIGTSITQKGIIFILLMLVTPSMAMRCVGIGSRDFVEGLSGATWVDVELEHGSCVTTMAKDKPTLDIELLKTEVTNPAVLRKLCIEAKISNTTTDSRCPTQGEATLVEEQDANFVCRRTFVDRGWGNGCELFGKGSLLTCAKFKCVTKLEGKIVQYENLKYSVIVTVHIAAQHQVGNESTEHGTTAIITPQAPTTEIQLTDYGALTLDCSPRTGLDFNEMVLLTMKEKSWLVHKQWFLDLPLPWTSGASTSQETWNRLFLLVTFKTAHAKKQEVVVLGSQEGAMHTALTGATALQTFGTTTIFAGHLNIRLKMDKLTLKGMSYVMCTGSFKLEKEVAETQHGTILVQIKYEGTDAPCKIPFSTQDEKGVTQNGRLITANPIVTDKEKPVNIEAEPPFGESYIVIGAGEKALKLSWFKKGSSIGKMFEATARGARRMAILGDTAWDFGSIGGVFTSVGKLVHQIFGTAYGVLFSGVSWTMKIGIGVLLTWLGLNSRSTSLSMTCIAVGLVTLYLGVMVQADSGCVINWKGRELKCGSGIFVTNEVHTWTEQYKFQADSPKRLSAAIGKAWEEGVCGIRSATRLENIMWKQISNELNHILLENDLKFTVVVGDVAGILAQGKKMIRPQPMEHKYSWKNWGKAKIIGADVQNSTFIIDGPNTPECSDDQRAWNIWEVEDYGFGIFTTNIWLKLRDSYTQVCDHRLMSAAIKDSKAVHADMGYWIESEKNETWKLARASFIEVKTCTWPKSHTLWSNGVLESEMIIPKIYGGPISQHNYSPGYFTQTAGPWHLGKLELDFDLCEGTTVVVDEHCGNRGPSLRTTTVTGKIIHEWCCRSCTLPPLRFRGEDGCWYGMEIRPVKEKEENLVKSMVSAGSGEVVSLSLGLLCISIMIEEVMRSRWSRKMLMTGTLAVFFRKIRGQLTWNDLIRLCIMVGANASDRMGTRTTYLALMATFKMRPMFSVGLLFRRLTSREVLLLTIGLSLVASVELPNSLEELGVLLAMGIMILTLLTDFQSHQLWATLLSMTFIKTTFSLHYTWKTMAMVLSIVSLFPLCLSTTSQKTTWLPVLLGSLGCKPLTMFLIAENKIWGRKSWPLNEGIMAVGIVSILLSSLLKNDVPLAGPLIAGGMLIACYVISGSSADLSLEKAAEVSWEEEAEHSGASHNILVEVQDDGTMKIKDEERDDTLTILLKATLLAVSGVYPLSIPATLFVWYFWQKKKQRSGVLWDTPSPPKVEKAVLDDGIYRIMQRGLLGRSQVGVGVFQENVFHTMWHVTRGAVLMYQGKRLEPSWGRVKKDLISYGGGWGLKGSWNTGEEVQVIAVEPGKNPKNVQTAPGTFKTPEGEVGAIALDFKPGTSGSPIVNREGKIVGLYGNGVVTTSGTYVSAIAHAKASQEGPLPETEVEAFRKRNLTIMDLHPGSGKTRRYLPAIVREAIKRKMRTLILAPTRVVASEMAEALKGMPIRYQTTAVKSEHTGKEIVDLMCHATFTMRLLSPVRVPNYNMIIMDEAHFTDPSSLAARGYISTRVGMGEAAAIFMTATPPGSVEAFPQSNAVIQDEERDIPERSWNSGYEWITDFPGKTVWFVPSIKAGNDIANCLRKNGKRVIQLSRKTFDTEYQKTKNNDWDYVVTTDISEMGANFPADRVIDPRRCLNPVILKDGPERVILAGPMPVTVASANQRRGRIGRNHNKEGDQYIYMGQPLNNDEDHAHWTEAKMLLDNINTPEGIIPALFEPEREKSAAIDGEYRLRGEARKTFVELMRRGDLPVWLSYKVASEGFQYSDRRWCFDGERNNQVLEENMDVEIWTKEGERKKLRPRWLDARTYSDPLALREKKEFAAGRRSVSGDLILEIGKLPQHLTQRAQNALDNLVMLHNSEQGGRAYRHAMEELPDTIETLMLLALIAVLTGGVTLFFLSGKGLGKAGNGLLCVMASSALLWMANVEPHWIAASIILEFFLMVLLIPEPDRQRTPQDNQLAYVVIRLLFMILTVAANEMGLLETTKKDLGIGHAAVENHHHAAMLDIDLHPASAWTLYAVATTIITPMMRHTIENTTANISLTAIANQAAILMGLDKGWPISKMDIGVPLLALGFLSQVNPLTLTAAVLMLVAHYAIIGPGLQAKATREAQKRTAAGIMKNPTIDGIVAIDLDPVVYDAKFEKQLGQIMLLILCTSQILLMRTTWALCESITLATGPLSPLWEGSPGKSWNTTIAVSMANIFRGSYLAGAGLAFSLMKSLGGGRRGTGAQGETLGEKWKRQLNQLSKTEFNTYKRSGIMEVDRSEAKEGLKRGETTKHAVSRGTAKLRWFVERNLVKPEGKVIDLGCGRGGWSYYCAGLKKVSEVRGYTKGGPGHEEPIPMATYGWDLLKRCSGKEFFFIPPEKCDSFLGDIGESSPNPTIEEGRTVRVLKMVEPWLRGNQFCIKILNPYMPSVVETLEQMQRKHGGMLVRNPLSRNSTHEMYWVSCGTGNIVSAVNMTSRMLLNRFTMAHRKPTYERDVDLGAGTRHVAVEPEVANLDIIGQRIENIKHEHKSTWHYDEDNPYKTWAYHGSYEVKPSGSASSMVNGVVKLLTKPWDVIPMVTQIAMTDTTPFGQQRVFKEKVDTRTPKAKRGTAQIMEVTARWLWSFLSRNKKPRICTREEFTRKVRSNAAIGAVFVDENQWNSAKEAVEDDRFWDLVHRERELHKQGKCATCVYNMMGKREKKLGEFGKAKGSRAIWYMWLGARFLEFEALGFMNEDHWFSRENSLSGVEGEGLHKLGYILRDISKIPGGNMYADDTAGWDTRITEDDLQNEAKITDIMEPEHALLAKSIFKLTYQNKVVRVQRPAKNGTVMDVISRRDQRGSGQVGTYGLNTFTNMEAQLIRQMESEEIFSPNELETPNLAERVLDWLEKYGVERLKRMAISGDDCVVKPIDDRFATALTALNDMGKVRKDIPQWEPSKGWNDWQQVPFCSHHFHQLVMKDGREIVVPCRNQDELVGRARVSQGAGWSLRETACLGKSYAQMWQLMYFHRRDLRLAANAICSAVPVDWVPTSRTTWSIHAHHQWMTTEDMLSVWNRVWIEENPWMEDKTRVSSWEDVPYLGKREDQWCGSLIGLTARATWATNIQVAINQVRRLIGNENYSDYMTSMKRFKNESEPEGALW

>DENV1_BAU61357_Philippines

MNNQRKKTGRPSFNMLKRARNRVSTVSQLAKRFSKGLLSGQGPMKLVMAFIAFLRFLAIPPTAGILARWSSFKKNGAIKVLRGFKKEISNMLNIMNRRRRSVTMLLMLLPTALAFHLTTRGGEPHMIVSKQERGKSLLFKTSAGVNMCTLIAMDLGELCEDTMTYKCPRITETEPDDVDCWCNATETWVTYGTCSQTGEHRRDKRSVALAPHVGLGLETRTETWMSSEGAWRQIQKVETWALRHPGFTVIALFLAHAIGTSITQKGIIFILLMLVTPSMAMRCVGIGNRDFVEGLSGATWVDVVLEHGSCVTTMAKDKPTLDIELLKTEVTNPAVLRKLCIEAKISNTTTDSRCPTQGEATLVEEQDTNFVCRRTFVDRGWGNGCGLFGKGSLITCAKFKCVTKLEGKIVQYENLKYSVIVTVHTGDQHQVGNETTEHGTTATITPQAPTSEIQLTDYGALTLDCSPRTGLDFNEMVLLTMKEKSWLVHKQWFLDLPLPWTSGASTSQETWNRQDLLVTFKTAHAKKQEVVVLGSQEGAMHTALTGATEIQTSGTTTIFAGHLKCRLKMDKLTLKGVSYVMCTGSFKLEKEVAETQHGTVLVQVKYEGTDAPCKIPFSSQDEKGVTQNGRLITANPIVTDKEKPVNIEAEPPFGESYLVVGAGEKALKLSWFKKGSSIGKMFEATARGARRMAILGDTAWDFGSIGGVFTSVGKLIHQIFGTAYGVLFSGVSWTMKIGIGILLTWLGLNSRSTSLSMTCIAVGMVTLYLGVMVQADSGCVINWKGRELKCGSGIFVTNEVHTWTEQYKFQADSPKRLSAAIGKAWEEGVCGIRSATRLENIMWKQISNELNHILLENDMKFTVVVGDVNGILAQGKKMIRPQPMEHKYSWKSWGKAKVIGADVQNTTFIIDGPNTPECPDDQRAWNIWEVEDYGFGIFTTNIWLKLRDSYTQVCDHRLMSAAIKDSKAVHADMGYWIESEKNETWKLTRASFIEVKTCIWPKSHTLWSNGVLESEMIIPKTYGGPISQHNYRPGYFTQTAGPWHLGKLELDFGLCEGTTVVVDEHCGNRGPSLRTTTVTGKIIHEWCCRSCTLPPLRFKGEDGCWYGMEIRPVKEKEENLVRSMVSAGSGEVDSFSLGLLCISIMIEEVMRSRWSRKMLMTGTLAVFLLLIMGQLTWNDLIRLCIMVGANASDKMGMGTTYLALMATFRMRPMFAVGLLFRRLTSREVLLLTVGLSLVASVELPNSLEELGDGLAMGIMMLKLLTDFQSHQLWAALLSLTFVKTTFSLHYAWKTMAMILSIISLFPLCMSTTSQKTTWLPVLLGSLGCKPLTMFLITENKIWGRKSWPLNEGIMAVGIVSILLSSLLKNDVPLAGPLIAGGMLIACYVISGSSADLSLEKAAEVSWEEEAEHSGASHNILVEVQDDGTMKIKDEERDDTLTILLKATLLAISGVYPMSIPATLFVWYFWQKKKQRSGVLWDTPSPPEVERAVLDDGIYRILQRGLLGKSQVGVGVFQEGVFHTMWHVTRGAVLMYQGKRLEPSWASVKKDLISYGGGWRFQGSWNTGEEVQVIAVEPGKNPKNVQTAPGTFKTPEGEVGAIALDFKPGTSGSPIVNREGKIVGLYGNGVVTTSGTYVSAIAQAKASQEGPLPEIEDEVFRKRNLTIMDLHPGSGKTRRYLPAIVREAIKRKLRTLVLAPTRVVASEMAEALKGMPIRYQTTAVKSEHTGKEIVDLMCHATFTMRLLSPVRVPNYNMIIMDEAHFTDPASIAARGYISTRVGMGEAAAIFMTATPPGSMEAFPQSNAAIQDEERDIPERSWNSGYDWITDFPGKTVWFVPSIKSGNDIANCLRKNGKRVVQLSRKTFDTEYQKTKNNDWDYVVTTDISEMGANFRADRVIDPRRCLKPVILKDGPERVILAGPMPVTVASAAQRRGRIGRNQNKEGDQYIYMGQPLNNDEDHAHWTEAKMLLDNINTPEGIIPALFEPEREKSAAIDGEYRLRGEARKTFVELMRRGDLPVWLSYKVASEGFQYSDRRWCFDGERNNQVLEENMDVEIWTKEGERKKLRPRWLDARTYSDPLALREFKEFAAGRRSVSGDLILEIGKLPQHLTQRAQNALDNLVMLHNSEQGGKAYRHAMEELPDTIETLMLLALIAVLTGGVTLFFLSGRGLGKTSIGLLCVMASSALLWMASVEPHWIAASIILEFFLMVLLIPEPDRQRTPQDNQLAYVVIGLLFMILTVAANEMGLLETTKKDLGIGHVAAESHHHAAMLDVDLHPASAWTLYAVATTIITPMMRHTIENTTANISLTAIANQAAILMGLDKGWPISKMDIGVPLLALGCYSQVNPLTLTAAVLMLVAHYAIIGPGLQAKATREAQKRTAAGIMKNPTVDGIVAIDLDPVVYDAKFEKQLGQIMLLILCTSQILLMRTTWALCESITLATGPLTTLWEGSPGKFWNTTIAVSMANIFRGSYLAGAGLAFSLMKSLGGGRRGTGAQGETLGEKWKRQLNQLSKSEFNTYKRSGIMEVDRSEAKEGLKRGETTKHAVSRGTAKLRWFVERNLVKPEGKVIDLGCGRGGWSYYCAGLKKVTEVKGYTKGGPGHEEPIPMATYGWNLVKLYSGKDVFFTPPEKCDTLLCDIGESSPNPTIEEGRTLRVLKMVEPWLRGNQFCIKVLNPYMPSVVETLEQMQRKHGGMLVRNPLSRNSTHEMYWVSCGTGNIVSAVNMTSRMLLNRFTMTHRKPTYEKDVDLGAGTRHVAVEPEVANLDIIGQRIENIKNEHKSTWHYDEDNPYKTWAYHGSYEVKPSGSASSMVNGVVRLLTKPWDVIPMVTQIAMTDTTPFGQQRVFKEKVDTRTPKAKRGTAQIMEVTARWLWGFLSRNKKPRICTREEFTRKVRSNAAIGAVFVDENQWNSAKEAVEDERFWDLVHRERELHKQGKCATCVYNMMGKREKKLGEFGKAKGSRAIWYMWLGARFLEFEALGFMNEDHWFSRENSLSGVEGEGLHKLGYILRDISKIPGGNMYADDTAGWDTRITEDDLQNEAKITDIMEPEHALLATSIFKLTYQNKVVRVQRPAKNGTVMDVISRRDQRGSGQVGTYGLNTFTNMEAQLIRQMESEGIFLPSELETPNLAERVLDWLEKHGTERLKRMAISGDDCVVKPIDDRFATALTALNDMGKVRKDIPQWEPSKGWNDWQQVPFCSHHFHQLIMKDGREIVVPCRNQDELVGRARVSQGAGWSLRETACLGKSYAQMWQLMYFHRRDLRLAANAICSAVPVDWVPTSRTTWSIHAHHQWMTTEDMLSVWNRVWIEENPWMEDKTHVSSWEDVPYLGKREDQWCGSLIGLTARATWATNIQVAINQVRRLIGNENYLDYMTSMKRFKNESDPEGALW

>DENV1_ALI16134_South_Korea

MNNQRKKTARPSFNMLKRARNRVSTVSQLAKRFSKGLLSGQGPMKLVMAFIAFLRFLAIPPTAGILARWGSFKKNGAIKVLRGFKKEISNMLNIMNRRKRSVTMLLMLMPTALAFHLTTRGGEPHMIVSKQERGKSLLFKTSAGINMCTLIAMDLGELCEDTLTYKCPRITEAEPDDVDCWCNATDTWVTYGTCSQTGEHRRDKRSVALAPHVGLGLETRTETWMSSEGAWKQIQRVETWALRHPGFTVIALFLAHAIGTSITQKGIIFILLMLVTPSMAMRCVGIGSRDFVEGLSGATWVDVVLEHGSCVTTMAKDKPTLDIELLKTEVTNPAVLRKLCIEAKISNTTTDSRCPTQGEATLVEEQDANFVCRRTFVDRGWGNGCGLFGKGSLITCAKFKCVTKLEGKIVQYENLKYSVIVTVHTGDQHQVGNESTEHGTTATITPQAPTTEIQLTDYGALTLDCSPRTGLDFNEMVLLTMKEKSWLVHKQWFLDLPLPWTSGASTSQEIWNRQDLLVTFKTAHAKKQEVVVLGSQEGAMHTALTGATEIQTSGTTTIFAGHLKCRLKMDKLTLKGMSYVMCTGSFKLEKEVAETQHGTVLVQIKYEGTDAPCKIPFSTQDEKGVTQNGRLITANPIVTDKEKPVNIEAEPPFGESYIVIGAGEKALKLSWFKKGSSIGKMFEATARGARRMAILGDTAWDFGSIGGVFTSVGKLVHQIFGTAYGVLFSGVSWTMKIGIGVLLTWLGLNSRSTSLSMTCIAVGLVTLYLGVMVQADSGCVINWKGRELKCGSGIFVTNEVHTWTEQYKFQADSPKRLSAAIGKAWEEGVCGIRSATRLENIMWKQISNELNHILLENDMKFTVVVGDVAGILAQGKKMIRPQPMEHKYSWKSWGKAKIIGADVQNTTFIIDGPNTPECPDDQRAWNIWEVEDYGFGIFTTNIWLKLRDSYTQVCDHRLMSAAIKDSKAVHADMGYWIESEKNETWKLARASFIEVKTCIWPKSHTLWSNGVLESEMIIPKIYGGPISQHNYRPGYFTQTAGPWHLGKLELDFDLCEGTTVVVDEHCGNRGPSLRTTTVTGKIIHEWCCRSCTLPPLRFRGEDGCWYGMEIRPVKEKEENLVKSMVSAGSGEVDSFSLGLLCISIMIEEVMRSRWSRKMLMTGTLAVFFLLIMGQLTWNDLIRLCIMVGANASDRMGMGTTYLALMATFKMRPMFAVGLLFRRLTSREVLLLTIGLSLVASVELPNSLEELGDGLAMGIMILKLLTDFQSHQLWATLLSLTFIKTTFSLHYAWKTMAMVLSIVSLFPLCLSTTSQKTTWLPVLLGSLGCKPLTMFLIAENKIWGRKSWPLNEGIMAVGIVSILLSSLLKNDVPLAGPLIAGGMLIACYVISGSSADLSLEKAAEVSWEEEAEHSGASHNILVEVQDDGTMKIKDEERDDTLTILLKATLLAVSGVYPLSIPATLFVWYFWQKKKQRSGVLWDTPSPPEVERAVLDDGIYRIMQRGLLGRSQVGVGVFQENVFHTMWHVTRGAVLMYQGKRLEPSWASVKKDLISYGGGWRLQGSWNTGEEVQVIAVEPGKNPKNVQTAPGTFKTPEGEVGAIALDFKPGTSGSPIVNREGKIVGLYGNGVVTTSGTYVSAIAQAKASQEGPLPEIEDEVFRKRNLTIMDLHPGSGKTRRYLPAIVREAIKRKLRTLILAPTRVVASEMAEALKGMPIRYQTTAVKSEHTGKEIVDLMCHATFTMRLLSPVRVPNYNMIIMDEAHFTDPSSIAARGYISTRVGMGEAAAIFMTATPPGSVEAFPQSNAVIQDEERDIPERSWNSGYEWITDFPGKTVWFVPSIKSGNDIANCLRKNGKRVIQLSRKTFDTEYQKTKNNDWDYVVTTDISEMGANFRADRVIDPRRCLKPVILKDGPERVILAGPMPVTVASAAQRRGRVGRNHNKEGDQYIYMGQPLNNDEDHAHWTEAKMLLDNINTPEGIIPALFEPEREKSAAIDGEYRLRGEARKTFVELMRRGDLPVWLSYKVASEGFQYSDRRWCFDGERNNQVLEENMDVEIWTKEGERKKLRPRWLDARTYSDPLALREFKEFAAGRRSVSGDLILEIGKLPQHLTQRAQNALDNLVMLHNSEQGGRAYRHAMEELPDTIETLMLLALIAVLTGGVTLFFLSGRGLGKTSIGLLCVMASSVLLWMASVEPHWIAASIILEFFLMVLLIPEPDRQRTPQDNQLAYVVIGLLFMILTVAANEMGLLETTKKDLGIGHVAVENHHHAAMLDVDLHPASAWTLYAVATTIITPMMRHTIENTTANISLTAIANQAAILMGLDKGWPISKMDIGVPLLALGCYSQVNPLTLTAAVLMLVAHYAIIGPGLQAKATREAQKRTAAGIMKNPTVDGIVAIDLDPVVYDAKFEKQLGQIMLLILCTSQILLMRTTWALCESITLATGPLTTLWEGSPGKFWNTTIAVSMANIFRGSYLAGAGLAFSLMKSLGGGRRGTGAQGETLGEKWKRQLNQLSKSEFNTYKRSGIMEVDRSEAKEGLKRGETTKHAVSRGTAKLRWFVERNLVKPEGKVIDLGCGRGGWSYYCAGLKKVTEVKGYTKGGPGHEEPIPMATYGWNLVKLHSGKDVFFIPPEKCDTLLCDIGESSPNPTIEEGRTLRVLKMVEPWLRGNQFCIKILNPYMPSVVETLEQMQRKHGGMLVRNPLSRNSTHEMYWVSCGTGNIVSAVNMTSRMLLNRFTMAHRKPTYERDVDLGAGTRHVAVEPEVANLDIIGQRIENIKHEHKSTWHYDEDNPYKTWAYHGSYEVKPSGSASSMVNGVVKLLTKPWDVIPMVTQIAMTDTTPFGQQRVFKEKVDTRTPKAKRGTTQIMEVTARWLWGFLSRNKKPRICTREEFTRKVRSNAAIGAVFVDENQWNSAKEAVEDERFWDLVHRERELHKQGKCATCVYNMMGKREKKLGEFGKAKGSRAIWYMWLGARFLEFEALGFMNEDHWFSRENSLSGVEGEGLHKLGYILRDISKIPGGNMYADDTAGWDTRITEDDLQNEAKITDIMEPEHALLAKAIFKLTYQNKVVRVQRPAKNGTVMDVISRRDQRGSGQVGTYGLNTFTNMEAQLIRQMESEGIFSPSELETPNLAERVLDWLEKYGVERLKRMAISGDDCVVKPIDDRFATALTALNDMGKVRKDIPQWEPSKGWNDWQQVPFCSHHFHQLIMKDGREIVVPCRNQDELVGRARVSQGAGWSLRETACLGKSYAQMWQLMYFHRRDLRLAANAICSAVPVDWVPTSRTTWSIHAHHQWMTTEDMLSVWNRVWIEENPWMEDKTHISSWEDVPYLGKREDQWCGSLIGLTARATWATNIQVAINQVRRLIGNENYLDYMTSMKRFKNESDPEGALW

>DENV1_AIG59667_Saudi_Arabia

MNNQRKKTGRPSFNMLKRARNRVSTVSQLAKRFSKGLLSGQGPMKLVMAFIAFLRFLAIPPTAGILARWGSFKKNGAIKVLRGFKKEISNMLNIMNRRKRSVTMLLMLLPTVLAFHLTTRGGEPHMIVTKQERGKSLLFKTSTGVNMCTLIAMDLGELCEDTMTYKCPRITEAEPDDIDCWCNATDTWVTYGTCSQTGEHRRDKRSVALAPHVGLGLETRTETWMSSEGAWKQIQRVETWALRHPGFTVTALFLAHAIGTSITQKGIIFILLMLVTPSMAMRCVGIGNRDFVEGLSGATWVDVVLEHGSCVTTMAKDKPTLDIELLKTEVTNPAVLRKLCIEAKISNTTTDSRCPTQGEATLVEEQDANFVCRRTFVDRGWGNGCGLFGKGSLITCAKFKCVTKLEGKIVQYENLKYSVIVTVHTGDQHQVGNESTEHGTTATITPQAPTSEIQLTDYGALTLDCSPRTGLDFNEMVLLTMKEKSWLVHKQWFLDLPLPWTSGATTSQETWNRQDLLVTFKTAHAKKQEVVVLGSQEGAMHTALTGATEIQTSGTTTIFAGHLKCRLKMDKLTLKGMSYVMCTGSFKLEKEVVETQHGTVLVQVKYEGTDAPCKIPFLTQDEKGVTQNGRVITANPIVTDKEKPVNIEAEPPFGESYIVVGAGEKALKLSWFKKGSTIGKMFEATARGARRMAILGDTAWDFGSIGGVFTSVGKLVHQIFGTAYGVLFSGVSWTMKIGIGVLLTWLGLNSRSTSLSMTCIAVGLVTLYLGVMVQADSGCVINWKGRELKCGSGIFVTNEVHTWTEQYKFQADSPKRLSAAIGKAWEEGVCGIRSATRLENIMWKQISNELNHILLENDMKFTVVVGDVSGILAQGKKMIRPQPMEHKYSWKSWGKAKIIGADVQNSTFIIDGPNTPECPDDQRAWNIWEVEDYGFGIFTTNIWLKLRDSYTQVCDHRLMSAAIKDSKAVHADMGYWIESEKNETWKLARASFIEVKTCVWPKSHTLWSNGVLESEMIIPKIYGGPISQHNYRPGYFTQTAGPWHLGKLELDFDLCEGTTVVVDEHCGNRGPSLRTTTVTGKIIHEWCCRSCTLPPLRFKGEDGCWYGMEIRPVKEKEENLVKSMVSAGSGEVDSFSLGLLCISIMIEEVMRSRWSRKMLMTGTLAVFLLLIMGQLTWNDLIRLCIMVGANASDRMGMGTTYLALMATFKMRPMFAVGLLFRRLTSREVLLLTIGLSLVASVELPNSLEELGDGLAMGIMILKLLTDFQSHQLWAALLSLTFIKTTCSLHYAWKTMAMVLSIVSLFPLCMSTTSQKTTWLPVLLGSLGCKPLTMFLIAENKIWGRKSWPLNEGIMAVGIVSILLSSLLKNDVPLAGPLVAGGMLIACYVISGSSADLSLEKAAEVSWEEEAEHSGASHNILVEVQDDGTMKIKDEERDDTLTILLKATLLAVSGVYPLSIPATLFVWYFWQKKKQRSGVLWDTPSPPEVERAVLDDGIYRIMQRGLLGRSQVGVGVFQENVFHTMWHVTRGAVLMYKGKRLEPTWASVKKDLISYGGGWRLQGSWNTGEEVQVIAVEPGKNPKNVQTAPGTFKTPEGEVGAIALDFKPGTSGSPIVNREGKIVGLYGNGVVTTSGTYVSAIAQAKASQEGPLPEIEDEVFRKRNLTIMDLHPGSGKTRRYLPAIVREAIKRKLRTLILAPTRVVASEMAEALKGMPIRYQTTAVKSEHTGKEIVDLMCHATFTMRLLSPVRVPNYNMIIMDEAHFTDPSSIAARGYISTRVGMGEAAAIFMTATPPGSVEAFPQSNAVIQDEERDIPERSWNSGYDWITDFPGKTVWFVPSIKSGNDIANCLRKNGKRVIQLSRKTFDTEYQKTKNNDWDYVVTTDISEMGANFRADRVIDPRRCLKPVILKDGPERVILAGPMPVTVASAAQRRGRIGRNQNKEGDQYIYMGQPLNNDEDHAHWTEAKMLLDNINTPEGIIPALFEPEREKSAAIDGEYRLRGEARKTFVELMRRGDLPVWLSYKVASEGFQYSDRRWCFDGERNNQVLEENMDVEIWTKEGERKKLRPRWLDARTYSDPLALREFKEFAAGRRSVSGDLILEIGKLPQHLTQRAQSALDNLVMLHNSEQGGRAYRHAMEELPDTIETLMLLALIAVLTGGVTLFFLSGKGLGKTSIGLLCVMASSMLLWMASVEPHWIAASIILEFFLMVLLIPEPDRQRTPQDNQLAYVVIGLLFMILTVAANEMGLLETTKKDLGIGHVAVENHHHATMLDVDLHPASAWTLYAVATTIITPMMRHTIENTTANISLTAIANQAAILMGLDKGWPISKMDIGVPLLALGCYSQVNPMTLTAAVLMLVAHYTIIGPGLQAKATREAQKRTAAGIMKNPTVDGIVAIDLDPVVYDAKFEKQLGQIMLLILCTSQILLMRTTWALCESITLATGPLTTLWEGSPGKFWNTTIAVSMANIFRGSYLAGAGLAFSLMKSLGGGRRGTGAQGETLGEKWKRQLNQLSKSEFNTYKRSGIIEVDRSEAKEGLKRGETTKHAVSRGTAKLRWFVERNLVKPEGKVIDLGCGRGGWSYYCAGLKKVTEVKGYTKGGAGHEEPIPMATYGWNLVKLHSGKDVFFIPPEKCDTLLCDIGESSPNPTIEEGRTLRVLKMVEPWLRGNQFCIKILNPYMPSVVETLEQMQRKHGGMLVRNPLSRNSTHEMYWVSCGTGNIVSAVNMTSRMLLNRFTMAHRKPTYERDVDLGAGTRHVAVEPEVANLDIIGQRIENIKHEHKSTWHYDEDNPYKTWAYHGSYEVKPSGSASSMVNGVVRLLTKPWDVIPMVTQIAMTDTTPFGQQRVFKEKVDTRTPKAKRGTAQVMEVTARWLWGFLSRNKKPRICTREEFTRKVRSNAAIGAVFVDENQWNSAKEAVEDERFWDLVHRERELHKQGKCATCVYNMMGKREKKLGEFGKAKGSRAIWYMWLGARFLEFEALGFMNEDHWFSRENSLSGVEGEGLHRLGYILRDISKIPGGNMYADDTAGWDTRITEDDLQNEAKITDIMEPEHALLATSIFKLTYQNKVVRVQRPAKNGTVMDVISRRDQRGSGQVGTYGLNTFTNMEAQLIRQMESEGLFSPSELETPNLAERVLNWLGKYGVERLKRMAISGDDCVVKPIDDRFATALTALNDMGKVRKDIPQWEPSKGWNDWQQVPFCSHHFHQLIMKDGREIVVPCRNQDELVGRARVSQGAGWSLRETACLGKSYAQMWQLMYFHRRDLRLAANAICSAVPVDWVPTSRTTWSIHAHHQWMTTENMLSVWNRVWIEENPWMEDKTHVSSWEDVPYLGKREDQWCGSLIGLTARATWATNIQVAINQVRKLIGNENYLDYMTSMKRFKNDSDPEGALW

>DENV1_AFY10029_New_Caledonia

MNNQRKKTGRPSFNMLKRARNRVSTVSQLAKRFSKGLLSGQGPMKLVMAFIAFLRFLAIPPTAGILARWGSFKKNGAIKVLRGFKKEISNMLNIMNRRKRSVTMLLMLLPTVLTFHLTTRGGEPHMIVSKQERGKSLLFKTSAGVNMCTLIAMDLGELCDDTMTYKCPRITETEPDDVDCWCNATETWVTYGTCSQTGEHRRDKRSVALAPHVGLGLETRTETWMSSEGAWRQIQKVETWALRHPGFTVIALFLAHAIGTSITQKGIIFILLMLVTPSMAMRCVGIGNRDFVEGLSGATWVDVVLEHGSCVTTMAKDKPTLDIELLKTEVTNPAVLRKLCIEAKISNTTTDSRCPTQGEATLVEEQDTNFVCRRTFVDRGWGNGCGLFGKGSLITCAKFKCVTKLEGKIVQYENLKYSVIVTVHTGDQHQVGNETTEHGTIATITPQAPTSEIQLTDYGALTLDCSPRTGLDFNEMVLLTMKEKSWLVHKQWFLDLPLPWTTGASTSQETWNRQDLLVTFKTAHAKKQEVVVLGSQEGAMHTALTGATEIQTSGTTTIFAGHLKCRLKMDKLTLKGISYVMCTGSFKLEKEVAETQHGTVLVQVKYEGTDAPCKIPFSSQDEKGVTQNGRLVTANPIVTDKERPVNIEAEPPFGESYIVVGAGEKALKLSWFKKGSSIGKMFEATARGARRMAILGDTAWDFGSIGGVFTSVGKLVHQIFGTAYGVLFSGVSWTMKIGIGILLTWLGLNSRSTSLSMTCIAVGMVTLYLGVMVQADSGCVINWKGRELKCGSGIFVTNEVHTWTEQYKFQADSPKRLSAAIGKAWEEGVCGIRSATRLENIMWKQISNELNHILLENDMKFTVVVGDVSGILAQGKKTIRPQPMEHKYSWKSWGKAKIIGADVQNTTFIIDGPNTPECPDDQRAWNIWEVEDYGFGIFTTNIWLKLRDSYTQVCDHRLMSAAIKDSKAVHADMGYWIESEKNETWKLARASFIEVKTCVWPKSHTLWSNGVLESEMVIPKIYGGPISQHNYRPGYFTQTAGPWHLGKLELDFDLCEGTTVVVDEHCGNRGPSLRTTTVTGKIIHEWCCRSCTLPPLRFKGEDGCWYGMEIRPVKEKEENLVKSMVSAGSGEVDSFSLGLLCISIMIEEVMRSRWSRKMLMTGTLVVFLLLTLGQLTWNDLIRLCIMVGANASDNMGMGTTYLALMATFRMRPMFAVGLLFRRLTSREVLLLTVGLSLVASVELPNSLDELGDGLAMGIMILKLLTDFQSHQLWATLLSLTFVKTTLSLHYAWKTMAMILSIVSLLPLCLSTTSQKTTWLPVLLGSLGCKPLTMFLITENKIWGRKSWPLNEGIMAVGIVSILLSSLLKNDVPLAGPLIAGGMLIACYVISGSSADLSLEKAAKVSWEEEAEHSGASHNILVEIQDDGTMKIKDEERDDTITILLKATLLAISGVYPMSIPATLFVWHFWQKKKQRSGVLWDTPSPPEVEKAVLDDGIYRILQRGLLGRSQVGVGVFQEGVFHTMWHVTRGAVLMYQGKRLEPSWASVKKDLISYGGGWRFQGSWNMGEEVQVIAVEPGKNPKNVQTAPGTFKTSEGEVGAIALDFKPGTSGSPIVNREGKIVGLYGNGVVTTSGTYVSAIAQAKASQEGPLPEIEDEVFRKRNLTIMDLHPGSGKTRRYLPAIVREAIKRKLRTLVLAPTRVVASEMAEALKGMPIRYQTTAVKSEHTGKEIVDLMCHATFTMRLLSPVRVPNYNMIIMDEAHFTDPASIAARGYISTRVGMGEAAAIFMTATPPGSVEAFPQSNAVIQDEEKDIPERSWNSGYDWITDFPGKTVWFVPSIKSGNDIANCLRKNGKRVIQLSRKTFDTEYQKTKNNDWDYVVTTDISEMGANFRADRVIDPRRCLKPVILKDGPERVILAGPMPVTVASAAQRRGRIGRNQNKEGDQYIYMGQPLNNDEDHAHWTEAKMLLDNINTPEGIIPALFEPEREKSAAIDGEYRLRGEARKTFVELMRRGDLPVWLSYKVASEGFQYSDRRWCFDGERNNQVLEENMDVEIWTKEGERKKLRPRWLDARTYSDPLALREFKEFAAGRRSVSGDLILEIGKLPQHLTQRAQNALDNLVMLHNSEQGGKAYRHAMEELPDTIETIMLLALIAVLTGGVTLFFLSGRGLGKTSIGLLCVTSSSALLWMASVEPHWIAASIILEFFLMVLLIPEPDRQRTPQDNQLAYVVIGLLFIILTVAANEMGLLETTKKDLGIGHTAAENHHHAAMLDVDLHPASAWTLYAVATTIITPMMRHTIENTTANISLTAIANQAAILMGLDKGWPISKMDIGVPLLALGCYSQVNPLTLTAAVLMLVAHYAIIGPGLQAKATREAQKRTAAGIMKNPTVDGIVAIDLDPVVYDAKFEKQLGQIMLLILCTSQILLMRTTWALCESITLATGPLTTLWEGSPGKFWNTTIAVSMANIFRGSYLAGAGLAFSLMKSLGGGRRGTGAQGETLGEKWKRQLNQLSKSQFNTYKRSGIIEVDRSEAKEGLKRGETTKHAVSRGTAKLRWFVERNLVKPEGKVIDLGCGRGGWSYYCAGLKKVTEVKGYTKGGPGHEEPIPMATYGWNLVKLHSGKDVFFIPPEKCDTLLCDIGESSPNPTIEEGRTLRVLKMVEPWLRGNQFCIKILNPYMPSVVETLEQMQRKHGGMLVRNPLSRNSTHEMYWVSCGTGNIVSAVNMTSRMLLNRFTMAHRKPTYERDVDLGAGTRHVAVEPEVANLDIIGQRIENIKNEHKSTWHYDEDNPYKTWAYHGSYEVKPSGSASSMVNGVVRLLTKPWDVIPMVTQIAMTDTTPFGQQRVFKEKVDTRTPKAKRGTAQIMEVTAKWLWGFLSRNKKPRICTREEFTRKVRSNAAIGAVFVDENQWNSAKEAVEDERFWDLVRRERELHKQGKCATCVYNMMGKREKKLGEFGKAKGSRAIWYMWLGARFLEFEALGFMNEDHWFSRENSLSGVEGEGLHKLGYILRDISKIPGGNMYADDTAGWDTRITEEDLQNEAKITDIMEPEHALLATSIFKLTYQNKVVRVQRPAKNGTVMDVISRRDQRGSGQVGTYGLNTFTNMEAQLIRQMESEGIFSPSELETPNLAGRVLDWLEKHGIERLRRMAISGDDCVVKPIDDRFATALTALNDMGKVRKDIPQWEPSKGWNDWQQVPFCSHHFHQLIMKDGREIVVPCRNQDELVGRARVSQGAGWSLRETACLGKSYAQMWQLMYFHRRDLRLAANAICSAVPVDWVPTSRTTWSIHAHHQWMTTEDMLSVWNRVWIEENPWMEDKTHVSSWEDVPYLGKREDQWCGSLIGLTARATWATNIQVAINQVRRLIGNENYLDYMTSMKRFKNESDPEGALW

>DENV1_ABG75766_Hawaii

MNNQRKKTGRPSFNMLKRARNRVSTVSQLAKRFSKGLLSGQGPMKLVMAFIAFLRFLAIPPTAGILARWSSFKKNGAIKVLRGFKKEISNMLNIMNRRKRSVTMLLMLLPTALAFHLTTRGGEPHMIVSKQERGKSLLFKTSAGVNMCTLIAMDLGELCEDTMTYKCPRITETEPDDVDCWCNATETWVTYGTCSQTGEHRRDKRSVALAPHVGLGLETRTETWMSSEGAWRQIQKVETWALRHPGFTVIAFFLAHAIGTSITQKGIIFILLMLVTPSMAMRCVGIGNRDFVEGLSGATWVDVVLEHGSCVTTMAKDKPTLDIELLKTEVTNPAVLRKLCIEARISNTTTDSRCPTQGEATLVEEQDTNFVCRRTFVDRGWGNGCGLFGKGSLITCAKFKCVTKLEGKIVQYENLKYSVIVTVHTGDQHQVGNETTEHGTTATITPQAPTSEIQLTDYGALTLDCSPRTGLDFNEMVLLTMKEKSWLVHKQWFLDLPLPWTSGASTSQETWNRQDLLVTFKTAHAKKQEVVVLGSQEGAMHTALTGATEIQTSGTTTIFAGHLKCRLKMDKLTLKGVSYVMCTGSFKLEKEVAETQHGTVLVQVKYEGTDAPCKIPFSSQDERGVTQNGRLITANPIVTDKEKPVNIEAEPPFGESYLVVGAGEKALKLSWFKKGSSIGKMFEATARGARRMAILGDTAWDFGSIGGVFTSVGKLIHQIFGTAYGVLFSGVSWTMKIGIGILLTWLGLNSRSTSLSMTCIAVGMVTLYLGVMVQADSGCVINWKGRELKCGSGIFVTNEVHTWTEQYKFQADSPKRLSAAIGKAWEEGVCGIRSATRLENIMWKQISNELNHILLENDMKFTVVVGDVNGVLAQGKKMIRPQPMEHKYSWKSWGKAKVIGADVQNITFIIDGPNTPECPDDQRAWNIWEVEDYGFGIFTTNIWLKLRDSYTQVCDHRLMSAAIKDSKAVHADMGYWIESEKNETWKLTRASFIEVKTCIWPKSHTLWSNGVLESEMIIPKIYGGPISQHNYRPGYFTQTAGPWHLGKLELDFGLCEGTTVVVDEHCGNRGPSLRTTTVTGKIIHEWCCRSCTLPPLRFKGEDGCWYGMEIRPVKEKEENLVKSMVSAGSGEVDSFSLGLLCISIMIEEVMRSRWSRKMLMTGTLAVFLLLIMGQLTWNDLIRLCIMVGANASDKMGMGTTYLALMATFRMRPMFAVGLLFRRLTSREVLLLTVGLSLVASVELPNSLEELGDGLAMGIMMLKLLTDFQSHQLWAALLSLTFVKTTFSLHYAWKTMAMILSIVSLFPLCLSTTSQKTTWLPVLLGSLGCKPLTMFLITENKIWGRKSWPLNEGIMAVGIVSILLSSLLKNDVPLAGPLIAGGMLIACYVISGSSADLSLEKAAEVSWEVEAEHSGASHNILVEVQDDGTMKIKDEERDDTLTILLKATLLAISGVYPMSIPATLFVWYFWQKKKQRSGVLWDTPSPPEVERAVLDDGIYRILQRGLLGKSQVGVGVFQEGVFHTMWHVTRGAVLMYQGKRLEPSWASVKKDLISYGGGWRFQGSWNMGEEVQVIAVEPGKNPKNVQTAPGTFKTPEGEVGAIALDFKPGTSGSPIVNREGKIVGLYGNGVVTTSGTYVSAIAQAKASQEGPLPEIEDEVFRKRNLTIMDLHPGSGKTRRYLPAIVREAIKRKLRTLVLAPTRVVASEMAEALKGMPIRYQTTAVKSEHTGKEIVDLMCHATFTMRLLSPVRVPNYNMIIMDEAHFTDPASIAARGYISTRVGMGEAAAIFMTATPPGSMEAFPQSNAAIQDEERDIPERSWNSGYDWITDFPGKTVWFVPSIKSGNDIANCLRKNGKRVVQLSRKTFDTEYQKTKNNDWDYVVTTDISEMGANFRADRVIDPRRCLKPVILKDGPERVILAGPMPVTVASAAQRRGRIGRNQNKEGDQYIYMGQPLNNDEDHAHWTEAKMLLDNINTPEGIIPALFEPEREKSAAIDGEYRLRGEARKTFVELMRRGDLPVWLSYKVASEGFQYSDRRWCFDGERNNQVLEENMDVEIWTKEGERKKLRPRWLDARTYSDPLALREFKEFAAGRRSVSGDLILEIGKLPQHLTQRAQNALDNLVMLHNSEQGGKAYRHAMEELPDTIETLMLLALIAVLTGGVTLFFLSGRGLGKTSIGLLCVMASSALLWMASVEPHWIAASIILEFFLMVLLIPEPDRQRTPQDNQLAYVVIGLLFMILTVAANEMGLLETTKKDLGIGHVAAESHHHAAMLDVDLHPASAWTLYAVATTIITPMMRHTIENTTANISLTAIANQAAILMGLDKGWPISKMDIGVPLLALGCYSQVNPLTLTAAVLMLVAHYAIIGPGLQAKATREAQKRTAAGIMKNPTVDGIVAIDLDPVVYDAKFEKQLGQIMLLILCTSQILLMRTTWALCESITLATGPLTTLWEGSPGKFWNTTIAVSMANIFRGSYLAGAGLAFSLMKSLGGGRRGTGAQGETLGEKWKRQLNQLSKSEFNTYKRSGIIEVDRSEAKEGLKRGETTKHAVSRGTAKLRWFVERNLVKPEGKVIDLGCGRGGWSYYCAGLKKVTEVKGYTKGGPGHEEPIPMATYGWNLVKLYSGKDVFFTPPEKCDTLLCDIGESSPNPTIEEGRTLRVLKMVEPWLRGNQFCIKVLNPYMPSVVETLEQMQRKHGGMLVRNPLSRNSTHEMYWVSCGTGNIVSAVNMTSRMLLNRFTMAHRKPTYERDVDLGAGTRHVAVEPEVANLDIIGQRIENIKNEHKSTWHYDEDNPYKTWAYHGSYEVKPSGSASSMVNGVVRLLTKPWDVISMVTQIAMTDTTPFGQQRVFKEKVDTRTPKAKRGTAQIMEVTARWLWGFLSRNKKPRICTREEFTRKVRSNAAIGAVFVDENQWNSAKEAVEDERFWDLVHRERELHKQGKCATCVYNMMGKREKKLGEFGKAKGSRAIWYMWLGARFLEFEALGFMNEDHWFSRENSLSGVEGEGLHKLGYILRDISKIPGGNMYADDTAGWDTRITEDDLQNEAKITDIMEPEHALLATSIFKLTYQNKVVRVQRPAKNGTVMDVISRRDQRGSGQVGTYGLNTFTNMEAQLIRQMESEGIFLPSELETPNLAERVLDWLEKHGIERLKRMAISGDDCVVKPIDDRFATALTALNDMGKVRKDIPQWEPSKGWNDWQQVPFCSHHFHQLIMKDGREIVVPCRNQDELVGRARVSQGAGWSLRETACLGKSYAQMWQLMYFHRRDLRLAANAICSAIPVDWVPTSRTTWSIHAHHQWMTTEDMLSVWNRVWIEENPWMEDKTHVSSWEDVPYLGKREDQWCGSLIGLTARATWATNIQVAINQVRRLIGNENYLDYMTSMKRFKNESDPEGALW

>DENV1_AAK60418_Djibouti

MNNQRKKTGRPSFNMLKRARNRVSTVSQLAKRFSKGLLSGQGPMKLVMAFIAFLTFLAIPPTAGILARWGSFKKNGAIKVLRGFKKEISNMLNIMNRRKRSVTMLLMLLPTVLAFHLTTRGGEPHMIVTKQERGKSLLFKTSTGVNMCTLIAMDLGELCEDTMTYKCPRITEAEPDDVDCWCNATDTWVTYGTCSQTGEHRRDKRSVALAPHVGLGLETRTETWMSSEGAWKQIQRVETWALRHPGFTVTALFLAHAIGTSITQKGIIFILLMLVTPSMAMRCVGIGNRDFVEGLSGATWVDVVLEHGSCVTTMAKDKPTLDIELLKTEVTNPAVLRKLCIEAKISNTTTDSRCPTQGEATLVEEQDANFVCRRTFVDRGWGNGCGLFGKGSLITCAKFKCVTKLEGKIVQYENLKYSVIVTVHTGDQHQVGNESTEHGTTATITPQAPTSEIQLTDYGALTLDCSPRTGLDFNEMVLLTMKEKSWLVHKQWFLDLPLPWTSGATTSQETWNRQDLLVTFKTAHAKKQEVVVLGSQEGAMHTALTGATEIQTSGTTTIFAGHLKCRLKMDKLTLKGMSYVMCTGSFKLEKEVAETQHGTVLVQVKYEGTDAPCKIPFSTQDEKGVTQNGRVITANPIVTDKEKPVNIEAEPPFGESYIVVGAGEKALKLSWFKKGSTIGKMFEATARGARRMAILGDTAWDFGSIGGVFTSVGKLVHQIFGTAYGVLFSGVSWTMKIGIGVLLTWLGLNSRSTSLSMTCIAVGLVTLYLGVMVQADSGCVINWKGRELKCGSGIFVTNEVHTWTEQYKFQADSPKRLSAAIGKAWEEGVCGIRSATRVENIMWKQISNELNHILFENDMKFTVVVGDVSGILAQGKKMIRPQPMEHKYSWKSWGKAKIIGADVQNSTFIIDGPNTPECPDDQRAWNIWEVEDYGFGIFTTNIWLKLRDSYTQVCDHRLMSAAIKDSKAVHADMGYWIESEKNETWKLARASFIEVKTCVWPKSHTLWSNGVLESEMIIPKIYGGPISQHNYRPGYFTQTAGPWHLGKLELDFDLCEGTTVVVDEHCGNRGPSLRTTTVTGKIIHEWCCRSCTLPPLRFKGEDGCWYGMEIRPVKEKEENLVKSMVSAGLGEVDSFSLGLLCISIMIEEVMRSRWSRKMLMTGTLAVFLLLIMGQLTWNDLIRLCIMIGANASDRMGMGTTYLALMATFKMRPMFAVGLLFRRLTSREVLLLTIGLSLVASVELPNSLEELGDGLAMGIMILKLLTDFQSHQLWAELLSLTFIKTTCSLHYAWKTMAMVLSIVSLFPLCMSTTSQKTTWLPVLLGSLGCKPLTMFLIAENKIWGRKSWPLNEGIMAVGIVSILLSSLLKNDVPLAGPLIAGGMLIACYVISGSSADLSLEKAAEVSWEEEAEHSGASHNILVEVQDDGTMKIKDEERDDTLTILLKATLLAVSGVYPLSIPATLFVWYFWQKKKQRSGVLWDTPSPPEVERAVLDDGIYRIMQRGLLGRSQVGVGVFQENVFHTMWHVTRGAVLMYQGKRLEPSWASVKKDLISYGGGWRLQGSWNTGEEVQVIAVEPGKNPKNVQTAPGTFKTPEGEVGAIALDFKPGTSGSPIVNREGKIVGLYGNGVVTTSGTYVSAIAQAKASQEGPLPEIEDEVFRKRNLTIMDLHPGSGKTRRYLPAIVREAIKRKLRTLILAPTRVVASEMAEALKGMPIRYQTTAVKSEHTGKEIVDLMCHATFTMRLLSPVRVPNYNMIIMDEAHFTDPSSIAARGYISTRVGMGEAAAIFMTATPPGSVEAFPQSNAVIQDEERDIPERSWNSGYDWITDFPGKTVWFVPSIKSGNDIANCLRKNGKRVIQLSRKTFDTEYQKTKNNDWDYVVTTDISEMGANFRADRVIDPRRCLKPVILKDGPERVILAGPMPVTVASAAQRRGRIGRNQNKEGDQYIYMGQPLNNDEDHAHWTEAKMLLDNINTPEGIIPALFEPEREKSAAIDGEYRLRGEARKTFVELMRRGDLPVWLSYKVASEGFQYSDRRWCFDGERNNQVLEENMDVEIWTKEGERKKLRPRWLDARTYSDPLALREFKEFAAGRRSVSGDLILEIGKLPQHLTQRAQNALDNLVMLHNSEQGGRAYRHAMEELPDTIETLMLLALIAVLTGGVTLFFLSGRGLGKTSIGLLCVMASSVLLWMASVEPHWIAASIILEFFLMVLLIPEPDRQRTPQDNQLAYVVIGLLFMILTVAANEMGLLETTKKDLGIGHVAVENHHHATMLDVDLHPASAWTLYAVATTIITPMMRHTIENTTANISLTAIANQAAILMGLDKGWPISKMDIGVPLLALGCYSQVNPLTLTAAVLMLVAHYAIIGPGLQAKATREAQKRTAAGIMKNPTVDGIVAIDLDPVVYDAKFEKQLGQIMLLILCTSQILLMRTTWALCESITLATGPLTTLWEGSPGKFWNTTIAVSMANIFRGSYLAGAGLAFSLMKSLGGGRRGTGAQGETLGEKWKRQLNQLSKSEFNTYKRSGIIEVDRSEAKEGLKRGETTKHAVSRGTAKLRWFVERNLVKPEGKVIDLGCGRGGWSYYCAGLKKVTEVKGYTKGGAGHEEPIPMATYGWNLVKLHSGKDVFFTPPEKCDTLLCDIGESSPNPTIEEGRTLRVLKMVEPWLRGNQFCIKILNPYMPSVVETLEQMQRKHGGMLVRNPLSRNSTHEMYWVSCGTGNIVSAVNMTSRMLLNRFTMAHRKPTYERDVDLGAGTRHVTVEPEVANLDIIGQRIENIKHEHKSTWHYDEDNPYKTWAYHGSYEVKPSGSASSMVNGVVRLLTKPWDVIPMVTQIAMTDTTPFGQQRVFKEKVDTRTPKAKRGTAQVMEVTARWLWGFLSRNKKPRICTREEFTRKVRSNAAIGAVFVDENQWNSAKEAVEDERFWDLVHRERELHKQGKCARCVYNMMGKREKKLGEFGKAKGSRAIWYMWLGARFLEFEALGFMNEDHWFSRENSLSGVEGEGLHRLGYILRGISKIPGGNMYADDTAGWDTRISEDDLQNEAKITDIMEPEHALLATSIFKLTYQNKVVRVQRPAKNGTVMDVISRRDQRGSGQVGTYGLNTFTNMEAQLIRQMESEGIFSPSELETPNLAQRVLNWLEKYGVERLKRMAISGDDCVVKPIDDRFATALTALNDMGKVRKDIPQWEPSKGWNDWQQVPFCSHHFHQLIMKDGREIVVPCRNQDELVGRARVSQGAEWSLRETACLGKSYAQMWQLMYFHRRDLRLAANAICSAVPVDWVPTSRTTWSIHAHHQWMTTENMLSVWNRVWIEENPWMEDKTHVSSWEDVPYLGKREDQWCGSLIGLTARATWATNIQVAINQVRRLIGNENYLDYMTSMKRFKNDSDPEGALW

>DENV1__AAB70696_Nauru_Island

MNNQRKKTGRPSFNMLKRARNRVSTVSQLAKRFSKGLLSGQGPMKLVMAFIAFLRFLAIPPTAGILARWGSFKKNGAIKVLRGFKKEISNMLNIMNRRKRSVTMLLMLLPTALAFHLTTRGGEPHMIVSKQERGKSLLFKTSAGVNMCTLIAMDLGELCEDTMTYKCPRITETEPDDVDCWCNATETWVTYGTCSQTGEHRRDKRSVALAPHVGLGLETRTETWMSSEGAWKQIQKVETWALRHPGFTVIALFLAHAIGTSITQKGIIFILLMLVTPSMAMRCVGIGNRDFVEGLSGATWVDVVLEHGSCVTTMAKDKPTLDIKLLKTEVTNPAVLRKLCIEAKISNTTTDSRCPTQGEATLVEEQDTNFVCRRTFVDRGWGNGCGLFGKGSLITCAKFKCVTKLEGKIVQYENLKYSVIVTVHTGDQHQVGNETIEHGTTATITPQAPTSEIQLTDYGALTLDCSPRTGLDFNEMVLLTMKKKSWLVHKQWFLDLPLPWTSGASTSQETWNRQDLLVTFKTAHAKKQEVVVLGSQEGAMHTASTGATEIQTSGTTTIFAGHLKCRLKMDKLILKGMSYVMCTGSFKLEKEVAETQHGTVLVQVKYEGTDAPCKIPFSSQDEKGVTQNGRLITANPIVTDKEKPVDIEAEPPFGESYIVVGAGEKALKLSWFKKGSSIGKMFEATARGARRMAILGDTAWDFGSIGGVFTSVGKLIHQIFGTAYGVLFSGVSWTMKIGIGILLTWLGLNSRSTSFSMTCIAVGMVTLYLGVMVQADSGCVINWKGRELKCGSGIFVTNEVHTWTEQYKFQADSPKRLSAAIGKAWEEGVCGIRSATRLENIMWKQISNELNHILLENDMKFTVVVGDVSGILAQGKKMIRPQPMEHKYSWKSWGKAKIIGADVQNTTFIIDGPNTPECPDNQRAWNIWEVEDYGFGIFTTNIWLKLRDSYTQVCDHRLMSAAIKDSKAVHADMGYWIESEKNETWKLARASFIEVKTCIWPKSHTLWSNGVLESEMIIPKIYGGPISQHNYRPGYFTQTAGPWHLGKLELDFDLCEGTTVVVDEHCGNRGPSLRTTTVTGKTIHEWCCRSCTLPPLRFKGEDGCWYGMEIRPAKEKEENLVKSMVSAGSGEVDSFSLGLLCISIMIEEVMRSRWSRKMLMIGTLAVFLLLTMGQLTWNDLIRLCIMVGANASDKMGMGTTYLALMATFRMRPMFAVGLLFRRLTSREVLLLTVGLSLVASVELPNSLEELGDGLAMGIMMLKLLTDFQSHQLWATLLSLTFVKTTFSLHYAWKTMAMILSIVSLFPLCLSTTSQKTTWLPVLLGSLGCKPLTMFLITENKIWGRKSWPLNEGIMAVGIVSILLSSLLKNDVPLAGPLIAGGMLIACYVISGSSADLSLEKAAEVSWEEEAEHSGASHNILVEVQDDGTMKIKDEERDDTLTILLKATLLAISGVYPMSIPATLFVWYFWQKKKQRSGVLWDTPSPPEVERAVLDDGIYRILQRGLLGRSQVGVGVFQEGVFHTMWHVTRGAVLMYQGKRLEPSWASVKKDLISYGGGWRFQGSWNAGEEVQVIAVEPGKNPKNVQTAPGTFKTPEGEVGAIALDFKPGTSGSPIVNREGKIVGLYGNGVVTTSGTYVSAIAQAKASQEGPLPEIEDEVFRKRKLTIMDLHPGSGKTRRYLPAIVREAIKRKLRTLVLAPTRVVASEMAEALKGMPIRYQTTAVKSEHTGKEIVDLMCHATFTMRLLSPVRVPNYNMIIMDEAHFTDPASIAARGYISTRVGMGEAAAIFMTATPPGSVEAFPQSNAVIQDEERDIPERSWNSGYDWITGFPGKTVWFVPSIKSGNDIANCLRKNGKRVVQLSRKTFDTEYQKTKNNDWDYVVTTDISEMGANFRADRVIDPRRCLKPVILKDGPERVILAGPMPVTVASAAQRRGRIGRNQNKEGDQYIYMGQPLNNDEDHAHWTEAKMLLDNINTPEGIIPALFEPEREKSAAIDGEYRLRGEARKTFVELMRRGDLPVWLSYKVASEGFQYSDRRWCFDGERNNQVLEENMDVEIWTKEGERKKLRPRWLDARTYSDPLALREFKEFAAGRRSVSGDLILEIGKLPQHLTQRAQNALDNLVMLHNSEQGGKAYRHAMEELPDTIETLMLLALIAVLTGGVTLFFLSGRGLGKTSIGLLCVIASSALLWMASVEPHWIAASIILEFFLMVLLIPEPDRQRTPQDNQLAYVVIGLLFMILTVAANEMGLLETTKKDLGIGHAAAENHHHAAMLDVDLHPASAWTLYAVATTIITPMMRHTIENTTANISLTAIANQAAILMGLDKGWPISKMDIGVPLLALGCYSQVNPLTLTAAVLMLVAHYAIIGPGLQAKATREAQKRTAAGIMKNPTVDGIVAIDLDPVVYDAKFEKQLGQIMLLILCTSQILLMRTTWALCESITLATGPLTTLWEGSPGKFWNTTIAVSMANIFRGSYLAGAGLAFSLMKSLGGGRRGTGAQGETLGEKWKRQLNQLSKSEFNTYKRSGIIEVDRSEAKEGLKRGETTKHAVSRGTAKLRWFVERNLVKPEGKVIDLGCGRGGWSYYCAGLKKVTEVKGYTKGGPGHEEPIPMATYGWNLVKLYSGKDVFFTPPEKRDTLLCDIGESSPNPTIEEGRTLRVLKMVEPWLRGNQFCIKILNPYMPSVVETLEQMQRKHGGMLVRNPLSRNSTHEMYWVSCGTGNIVSAVNMTSRMLLNRFTMAHRKPTYERDVDLGAGTRHVAVEPEVANLDIIGQRIENIKNEHKSTWHYDEDNPYKTWAYHGSYEVKPSGSASSMVNGVVRLLTKPWDVIPMVTQIAMTDTTPFGQQRVFKEKVDTRTPKAKRGTAQIMEVTARWLWGFLSRNKKPRICTREEFTRKVRSNAAIGAVFVDENQWNSAKEAVEDERFWDLVHRERELHKQGKCATCVYNMMGKREKKLGEFGKAKGSRAIWYVWLGARFLEFEALGFMNEDHWFSRENSLSGVEGEGLHKLGYILRDISKIPGGNMYADDTAGWDTRITEDDLQNEAKITDIMEPEHALLATSIFKLTYQNKVVRVQRPAKNGTVMDVISRRDQRGSGQVGTYGLNTFTNMEAQLIRQMESEGIFSPSELETPNLAERVLDWLKKHGTERLKRMAISGDDCVVKPIDDRFATALTALNDMGKVRKDIPQWEPSKGWNDWQQVPFCSHHFHQLIMKDGREIVVPCRNQDELVGRARVSQGAGWSLRETACLGKSYAQMWQLMYFHRRDLRLAANAICSAVPVDWVPTSRTTWSIHAHHQWMTTEDMLSVWNRVWIEENPWMEDKTHVSSWEDVPYLGKREDQWCGSLIGLTARATWATNIQVAINQVRRLIGNENYLDFMTSMKRFKNESDPEGALW

>DENV1__ABC07334_Comoros

MNNQRKKTGRPSFNMLKRARNRVSTGSQLAKRFSKGLLSGQGPMKLVMAFIAFLRFLAIPPTAGILARWSSFKKNGAIKVLRGFKKEISSMLNIMNRRKRSVTMLLMLLPTALAFHLTTRGGEPHMIVSKQERGKSLLFKTSAGVNMCTLIAMDLGELCEDTMTYKCPRITEAEPDDVDCWCNATDTWVTYGTCSQTGEHRRDKRSVALAPHVGLGLETRTETWMSSEGAWRQIQKVETWALRHPGFTVIALFLAHTIGTSITQKGIIFILLMLVTPSMAMRCVGIGNRDFVEGLSGATWVDVVLEHGSCVTTMAKNKPTLDIELLKTEVTNPAVLRKLCIEAKISNTTTDSRCPTQGEATLVEEQDANFVCRRTFVDRGWGNGCGLFGKGSLLTCAKFKCVTKLEGKIVQYENLKYSVIVTVHTGDQHQVGNETTEHGTIATITPQAPTSEIQLTDYGALTLDCSPRTGLDFNEMVLLTMKEKSWLVHKQWFLDLPLPWTSGASTSQETWNRQDLLVTFKTAHAKKQEVVVLGSQEGAMHTALTGATEIQTSGTTTIFAGHLKCRLKMDKLTLKGVSYVMCTGSFKLEKEVAETQHGTVLVQVKYEGTDAPCKIPFSTQDEKGVTQNGRLITANPIVTDKEKPVNIETEPPFGESYIVIGAGEKALKLSWFKKGSSIGKMFEATARGARRMAILGDTAWDFGSIGGVFTSVGKLVHQVFGTAYGVLFSGVSWTMKIGIGILLTWLGLNSRSTSLSMTCIVVGMVTLYLGVMVQADSGCVINWKGRELKCGSGIFVTNEVHTWTEQYKFQADSPKRLSAAIGKAWEEGVCGIRSATRLENIMWKQISNELNHILLENDMKFTVVVGDANGILTQGKKMIRPQPMEHKYSWKSWGKAKIIGADIQNTTFIIDGPDTPECPDDQRAWNIWEVEDYGFGIFTTNIWLKLRDSYTQMCDHRLMSAAIKDSKAVHADMGYWIESEKNETWKLARASFIEVKTCIWPRSHTLWSNGVLESEMIIPKIYGGPISQHNYRPGYFTQTAGPWHLGKLELDFDLCEGTTVVVDEHCGNRGPSLRTTTVTGKIIHEWCCRSCTLPPLRFKGEDGCWYGMEIRPVKEKEENLVRSMVSAGSGEVDSFSLGLLCVSIMVEEVMRSRWSRKMLMTGTLAVFFLLIMGQLTWNDLIRLCIMVGANVSDRMGMGTTYLALMATFKMRPMFAVGLLFRRLTSREVLLLTIGLSLVASVELPNSLEELGDGLAMGIMMLKLLTDFQSHQLWTTLLSLTFIKTTLSLDYAWKTIAMVLSIVSLFPLCLSTTSQKTTWLPVLLGSFGCKPLTMFLITENKIWGRKSWPLNEGIMAVGIVSILLSSLLKNDVPLAGPLIAGGMLITCYVISGSSADLSLEKAAEVSWEEEAEHSGASHNILVEVQDDGTMKIKDEERDDTLTILLKATLLAVSGVYPMSIPATLFVWYFWQKKKQRSGVLWDTPSPPEVERAVLDDGIYRILQRGLLGRSQVGVGVFQDGVFHTMWHVTRGAVLMYQGKRLEPSWASVKKDLISYGGGWRFQGSWNTGEEVQVIAVEPGKNPKNVQTTPGTFKTPEGEVGAIALDFKPGTSGSPIVNREGKIVGLYGNGVVTTSGTYVSAIAQAKASQEGPLPEIEDEVFKKRNLTIMDLHPGSGKTRRYLPAIVREAIKRKLRTLILAPTRVVASEMAEALKGMPIRYQTTAVKSEHTGREIVDLMCHATFTMRLLSPVRVPNYNMIIMDEAHFTDPASIAARGYISTRVGMGEAAAIFMTATPPGSVEAFPQSNAVIQDEERDIPERSWNSGYDWITDFPGKTVWFVPSIKSGNDIANCLRKNGKRVIQLSRKTFDTEYQKTKNNDWDYVVTTDISEMGANFRADRVIDPRRCLKPVILKDGPERVILAGPMPVTVASAAQRRGRIGRNQNKEGDQYIYMGQPLNNDEDHAHWTEAKMLLDNINTPEGIIPALFEPEREKSAAIDGEYRLRGEARKTFVELMRRGDLPVWLSYKVASEGFQYSDRRWCFDGERNNQVLEENMDVEIWTKEGERKKLRPRWLDARTYSDPLALREFKEFAAGRRSVSGDLILEIGKLPQHLTLRAQNALDNLVMLHNSEQGGKAYRHAMEELPDTIETLMLLALIAVLTGGVTLFFLSGKGLGKTSIGLLCVMASSVLLWMASVEPHWIAASIILEFFLMVLLIPEPDRQRTPQDNQLAYVVIGLLFMILTVAANEMGLLETTKKDLGIGHAVAENHHHATMLDIDLHPASAWTLYAVATTIITPMMRHTIENTTANISLTAIANQAAILMGLDKGWPISKMDIGVPLLALGCYSQVNPLTLTAAVLMLVAHYAIIGPGLQAKATREAQKRTAAGIMKNPTVDGIVAIDLDPVVYDAKFEKQLGQIMLLILCTSQILLMRTTWALCESITLATGPLTTLWEGSPGKFWNTTIAVSMANIFRGSYLAGAGLAFSLMKSLGGGRRGTGAQGETLGEKWKRQLNQLSKSEFNTYKRSGIMEVDRSEAKEGLKRGETTKHAVSRGTAKLRWFVERNLVKPEGKVIDLGCGRGGWSYYCAGLKKVTEVKGYTKGGPGHEEPIPMATYGWNLVKLHSGKDVFFTPPEKCDTLLCDIGESSPNPTIEEGRTLRVLKMVEPWLRGNQFCIKILNPYMPSVVETLEQMQRKHGGMLVRNPLSRNSTHEMYWVSCGTGNIVSAVNMTSRMLLNRFTMAHRKPTYERDVDLGAGTRHVAVEPEIANLDIIGQRIENIKNEHKSTWHYDEDNPYKTWAYHGSYEVKPSGSASSMVNGVVRLLTKPWDVIPMVTQIAMTDTTPFGQQRVFKEKVDTRTPRPKRGTAQIMEVTAKWLWGFLSRNKKPRICTREEFTRKVRSNAAIGAVFVDENQWNSAKEAVEDERFWDLVHRERELHKQGKCATCVYNMMGKREKKLGEFGKAKGSRAIWYMWLGARFLEFEALGFMNEDHWFSRENSLSGVEGEGLHKLGYILRDISKIPGGNVYADDTAGWDTRITEDDLQNEAKITDIMEPEHALLATSIFKLTYQNKVVRVQRPAKSGTVMDIISRRDQRGSGQVGTYGLNTFTNMEVQLIRQMESEGIFSPSELENSNLAERVLDWLEKHGVERLKRMAISGDDCVVKPTDDRFATALTALNDMGKVRKDIPQWEPSKGWNDWQQVPFCSHHFHQLIMKDGREIVVPCRNQDELVGRARVSQGAGWSLRETACLGKSYAQMWQLMYFHRRDLRLAANAICSAVPVDWVPTSRTTWSIHAHHQWMTTEDMLSVWNRVWIEENPWMEDKTHVSSWEDVPYLGKREDQWCGSLIGLTARATWATNIQVAIKQVRRLIGNENYLDYMTSMKRFKNESDPEGALW

>DENV1_AAW28114_Myanmar

MNNQRKKTGRPSFNMLKRARNRVSTGSQLAKRFSKGLLSGQGPMKLVMAFIAFLRFLAIPPTAGILARWSSFKKNGAIKVLRGFKKEISSMLNIMNRRKRSVTMLLMLLPTALAFHLTTRGGEPHMVVSKQERGKSLLFKTSAGVNMCTLIAMDLGELCEDTMTYKCPRITEAEPDDVDCWCNATDTWVTYGTCSQTGGHRRDKRSVALAPHVGLGLETRTETWMSSEGAWKQIQKVETWALRHPGFTVIALFLAHAIGTSITQKGIIFILLMLVTPSMAMRCVGIGNRDFVEGLSGATWVDVVLEHGSCVTTMAKNKPTLDIELLKTEVTNPAVLRKLCIEAKISNTTTDSRCPTQGEATLVEEQDANFVCRRTFVDRGWGNGCGLFGKGSLLTCAKFKCVTKLEGKIVQYENLKYTVIVTVHTGDQHQVGNETTEHGTIATITPQAPTSEIQLTDYGALTLDCSPRTGLDFNEMVLLTMKEKSWLVHKQWFLDLPLPWTSGASTSQETWNRQDLLVTFKTAHAKKQEVVVLGSQEGAMHTALTGATEIQTSGTTTIFAGHLKCRLKMDKLTLKGMSYVMCTGSFKLEKEVAETQHGTVLVQVKYEGADAPCKIPFSTQDEKGVTQNGRLITANPIVTDKEKPVNIETEPPFGESYIVIGAGEKALKLSWFKKGSSIGKMFEATARGARRMAILGDTAWDFGSIGGVFTSVGKLVHQVFGAAYGVLFSGVSWTMKIGIGILLTWLGINSRSTSLSMTCIAVGMVTLYLGVTVQADSGCVINWKGRELKCGSGIFVTNEVHTWTEQYKFQADSPKRLSAAIGKAWEEGVCGIRSATRLENIMWKQISNELNHILLENDMKFTVVVGDANGILTQGKKMIRPQPMEHKYSWKSWGKAKIIGADIQNTTFIIDGPDTPECPDDQRAWNIWEVEDYGFGIFTTNIWLKLRDSYTQMCDHRLMSAAIKDSKAVHADMGYWIESEKNETWKLARASFIEVKTCIWPRSHTLWSNGVLESEMIIPKIYGGPTSQHNYRPGYFTQTAGPWHLGKLELDFDLCEGTTVVVDEHCGNRGPSLRTTTVTGKIIHEWCCRSCTLPPLRFKGEDGCWYGMEIRPVKEKEENLVRSMVSAGSGEVDSFSLGLLCVSIMIEEVMRSRWSRKMLMTGTLAVFFLLIMGQLTWNDLIRVCIMVGANASDRMGMGTTYLALMATFKMRPMFAVGLLFRRLTSREVLLLTIGLSLVASVELPNSLEELGDGLAMGIMMLKLLTDFQSHQLWTTLLSLTFIKTTLSLDYAWKTMAMVLSIISLFPLCLSTTSQKTTWLPVLLGSFGCKPLTMFLITENKIWGRKSWPLNEGIMAVGIVSILLSSLLKNDVPLAGPLIAGGMLIACYVISGSSADLSLEKAAEVSWEEEAEHSGASHNILVEVQDDGTMKIKDEERDDTLTILLKATLLAVSGVYPMSIPATLFVWYFWQKKKQRSGVLWDTPSPPEVERAVLDDGIYRILQRGLLGRSQVGVGVFQDGVFHTMWHVTRGAVLMYQGKRLEPSWASVKKDLISYGGGWRFQGSWNTGEEVQVIAVEPGKNPKNVQTTPGTFKTPEGEVGAIALDFKPGTSGSPIVNREGKIVGLYGNGVVTTSGTYVSAIAQAKASQEGPLPEIEDEVFRKRNLTIMDLHPGSGKTRRYLPAIVREAIKRKLRTLILAPTRVVASEMAEALKGMPIRYQTTAVKSEHTGREIVDLMCHATFTMRLLSPVRVPNYNMIIMDEAHFTDPASIAARGYISTRVGMGEAAAIFMTAPPPGSVEAFPQSNAVIQDEERDIPERSWNSGYDWITDFPGKTVWFVPSIKSGNDIANCLRKNGKRVIQLSRKTFDTEYQKTKNNDWDYVVTTDISEMGANFRADRVIDPRRCLKPVILKDGPERVILAGPMPVTVASAAQRRGRIGRNQNKEGDQYVYMGQPLNNDEDHAHWTEAKMLLDNINTPEGIIPALFEPEREKSAAIDGEYRLRGEARKTFVELMRRGDLPVWLSYKVASEGFQYSDRRWCFDGERNNQVLEENMDVEIWTKEGERKKLRPRWLDARTYSDPLALREFKEFAAGRRSVSGDLILEIGKLPQHLTLKAQNALDNLVMLHNSEQGGKAYRHAMEELPDTIETLMLLALIAVLTGGVTLFFLSGKGLGKTSIGLLCVMASSVLLWMASVEPHWIAASIILEFFLMVLLIPEPDRQRTPQDNQLAYVVIGLLFMILTVAANEMGLLETTKKDLGIGHVVAENHHHATMLDIDLHPASAWTLYAVATTIITPMMRHTIENTTANISLTAIANQAAILMGLDKGWPISKMDIGVPLLALGCYSQVNPLTLTAAVLMLVAHYAIIGPGLQAKATREAQKRTAAGIMKNPTVDGIVAIDLDPVVYDAKFEKQLGQIMLLILCTSQILLMRTTWALCESITLATGPLTTLWEGSPGKFWNTTIAVSMANIFRGSYLAGAGLAFSLMKSLGGGRRGTGAQGETLGEKWKRQLNQLSKSEFNTYKRSGIMEVDRSEAKEGLKRGETTKHAVSRGTAKLRWFVERNLVKPEGKVIDLGCGRGGWSYYCAGLKKVTEVKGYTKGGPGHEEPIPMATYGWNLVKLHSGKDVFFIPPEKCDTLLCDIGESSPNPTIEEGRTLRVLKMVEPWLRGNQFCIKILNPYMPSVVETLEQMQRKHGGMLVRNPLSRNSTHEMYWVSCGTGNIVSAVNMTSRMLLNRFTMAHRKPTYEKDVDLGAGTRHVTMEPEVANLDIIGQRIENIKNEHKSTWHYDEDNPYKTWAYHGSYEVKPSGSASSMVNGVVRLLTKPWDVIPMVTQIAMTDTTPFGQQRVFKEKVDTRTPRAKRGTAQIMEVTAKWLWGFLSRNKKPRICTREEFTRKVRSNAAIGAVFVDENQWNSAKEAVEDEKFWDLVHRERELHKQGKCATCVYNMMGKREKKLGEFGKAKGSRAIWYMWLGARFLEFEALGFMNEDHWFSRENSLSGVEGEGLHKLGYILRDISKIPGGNMYADDTAGWDTRITEDDLQNEAKITDIMEPEHALLATSIFKLTYQNKVVRVQRPAKSGTVMDVISRRDQRGSGQVGTYGLNTFTNMEVQLIRQMESEGIFSPSELETPNLAERVLDWLEKHGVERLKRMAISGDDCVVKPTDDRFATALTALNDMGKVRKDIPQWEPSKGWNDWQQVPFCSHHFHQLIMKDGREIVVPCRNQDELVGRARVSQGAGWSLRETACLGKSYAQMWQLMYFHRRDLRLAANAICSAVPVDWVPTSRTTWSIHAHHQWMTTEDMLSVWNRVWIEENPWMEDKTHVPSWEDVPYLGKREDQWCGSLIGLTARATWATNIQVAINQVRRLIGNENYLDYMTSMKRFKNESDPEGALW

>DENV1__ACI48993_Chile_Easter_Island

MNNQRKKTGRPSFNMLKRARNRVSTVSQLAKRFSKGLLSGQGPMKLVMAFIAFLRFLAIPPTAGILARWGSFKKNGAIKVLRGFKKEISNMLNIMNRRKRSVTMLLMLLPTVLTFHLTTRGGEPHMIVSKQERGKSLLFKTSAGVNMCTLIAMDLGELCDDTMTYKCPRITETEPDDVDCWCNATETWVTYGTCSQTGEHRRDKRSVALAPHVGLGLETRTETWMSSEGAWRQIQKVETWALRHPGFTVIALFLAHAIGTSITQKGIIFILLMLVTPSMAMRCVGIGNRDFVEGLSGATWVDVVLEHGSCVTTMAKDKPTLDIELLKTEVTNPAVLRKLCIEAKISNTTTDSRCPTQGEATLVEEQDTNFVCRRTFVDRGWGNGCGLFGKGSLITCAKFKCVTKLEGKIVQYENLKYSVIVTVHTGDQHQVGNETTEHGTIATITPQAPTSEIQLTDYGALTLDCSPRTGLDFNEMVLLTMKEKSWLVHKQWFLDLPLPWTSGASTSQETWNRQDLLVTFKTAHAKKQEVVVLGSQEGAMHTALTGATEIQTSGTTTIFAGHLKCRLKMDKLTLKGISYVMCTGLFKLEKEVAETQHGTVLVQVKYEGTDAPCKIPFSSQDEKGATQNGRLVTANPIVTDKEKPVNIEAEPPFGESYIVVGAGEKALKLSWFKKGSSIGKMFEATARGARRMAILGDTAWDFGSIGGVFTSVGKLVHQIFGTAYGVLFSGVSWTMKIGIGILLTWLGLNSRSTSLSMTCIAVGMVTLYLGVMVQADSGCVINWKGRELKCGSGIFVTNEVHTWTEQYKFQADSPKRLSAAIGKAWEEGVCGIRSATRLENIMWKQISNELNHILLENDMKFTVVVGDVSGILAQGKKTIRPQPMEHKYSWKSWGKAKIIGADVQNTTFIIDGPNTPECPDDQRAWNIWEVEDYGFGIFTTNIWLKLRDSYTQVCDHRLMSAAIKDSKAVHADMGYWIESEKNETWKLARASFIEVKTCVWPKSHTLWSHGVLESEMIIPKIYGGPISQHNYRPGYFTQTAGPWHLGKLELDFDLCEGTTVVVDEHCGNRGPSLRTTTVTGKIIHEWCCRSCTLPPLRFKGEDGCWYGMEIRPVKEKEENLVKSMVSAGSGEVDSFSLGLLCISIMIEEVMRSRWSRKMLMTGTLVVFLLLTLGQLTWNDLIRLCIMVGANASDNMGMGTTYLALMATFRMRPMFAVGLLFRRLTSREVLLLTVGLSLVASVELPNSLDELGDGLAMGIMILKLLTDFQSHQLWATLLSLTFVKTTLSLHYAWKTMAMILSIVSLLPLCLSTTSQKTTWLPVLLGSLGCKPLTMFLITENKIWGRKSWPLNEGIMAVGIVSILLSSLLKNDVPLAGPLIAGGMLIACYVISGSSADLSLEKAAKVSWEEEAEHSGASHNILVEIQDDGTMKIKDEERDDTITILLKATLLAISGVYPMSIPATLFVWHFWQKKKQRSGVLWDTPSPPEVEKAVLDDGIYRILQRGLLGRSQVGVGVFQEGVFHTMWHVTRGAVLMYQGKRLEPSWASVKKDLISYGGGWRFQGSWNTGEEVQVIAVEPGKNPKNVQTAPGTFKTSEGEVGAIALDFKPGTSGSPIVNREGKIVGLYGNGVVTTSGTYVSAIAQAKASQEGPLPEIEDEVFRKRNLTIMDLHPGSGKTRRYLPAIVREAIKRKLRTLVLAPTRVVASEMAEALKGMPIRYQTTAVKSEHTGKEIVDLMCHATFTMRLLSPVRVPNYNMIIMDEAHFTDPASIAARGYISTRVGMGEAAAIFMTATPPGSVEAFPQSNAVIQDEEKDIPERSWNSGYDWITDFPGKTVWFVPSIKSGNDIANCLRKNGKRVIQLSRKTFDTEYQKTKNNDWDYVVTTDISEMGANFRADRVIDPRRCLKPVILKDGPERVILAGPMPVTVASAAQRRGRIGRNQNKEGDQYIYMGQPLNNDEDHAHWTEAKMLLDNINTPEGIIPALFEPEREKSAAIDGEYRLRGEARKTFVELMRRGDLPVWLSYKVASEGFQYSDRRWCFDGERNNQVLEENMDVEIWTKEGERKKLRPRWLDARTYSDPLALREFKEFAAGRRSVSGDLILEIGKLPQHLTQRAQKALDNLVMLHNSEQGGKAYRHAMEELPDTIETIMLLALIAVLTGGVTLFFLSGRGLGKTSIGLLCVTSSSALLWMASVEPHWIAASIILEFFLMVLLIPEPDRQRTPQDNQLAYVVIGLLFMILTVAATEMGLLETTKKDLGIGHAAAENHHHAAMLDVDLHPASAWTLYAVATTIITPMMRHTIENTTANISLTAIANQAAILMGLDKGWPISKMDIGVPLLALGCYSQVNPLTLTAAVLMLVAHYAIIGPGLQAKATREAQKRTAAGIMKNPTVDGIVAIDLDPVVYDAKFEKQLGQIMLLILCTSQILLMRTTWALCESITLATGPLTTLWEGSPGKFWNTTIAVSMANIFRGSYLAGAGLAFSLMKSLGGGRRGTGAQGETLGEKWKRQLNQLSKSEFKTYKRSGIIEVDRSEAKEGLKRGETTKHAVSRGTAKLRWFVERNLVKPEGKVIDLGCGRGGWSYYCAGLKKVTEVKGSTKGGPGHEEPIPMATYGWNLVKLHSGKDVFFIPPEKCDTLLCDIGESSPNPTIEEGRTLRVLKMVEPWLRGNQFCIKILNPYMPSVVETLEQMQRKHGGMLVRNPLSRNSTHEMYWVSCGTGNIVSAVNMTSRMLLNRFTMAHRKPTYERDVDLGAGTRHVAVEPEVANLDIIGQRIENIKNEHKSTWHYDEDNPYKTWAYHGSYEVKPSGSASSMVNGVVRLLTKPWDVIPMVTQIAMTDTTPFGQQRVFKEKVDTRTPKAKRGTAQIMEVTAKWLWGFLSRNKKPRICTREEFTRKVRSNAAIGAVFVDENQWNSPKEAVEDERFWDLVHRERELHKQGKCATCVYNMMGKREKKLGEFGKAKGSRAIWYMWLGARFLEFEALGFMNEDHWFSRENSLSGVEGEGLHKLGYILRDISKIPGGNMYADDTAGWDTRITEDDLQNEAKITDIMEPEHALLATSIFKLTYQNKVVRVQRPAKNGTVMDVISRRDQRGSGQVGTYGLNTFTNMEAQLIRQMESEGIFLPSELEPPNLAGRVPDWLEKHGTERLRRMAISGDDCVVKPIDDRFATALTALNDMGKVRKDIPQWEPSKGWNDWQQVPFCSHHFHQLIMKDGREIVVPCRNQDELVGRARVSQGAGWSLRETACLGKSYAQMWQLMYFHRRDLRLAANAICSAVPVDWVPTSRTTWSIHAHHQWMTTEDMLSVWNRVWIEENPWMEDKTHVSSWEDVPYLGKREDQWCGSLIGLTARATWATNIQVAINQVRRLIGNENYLDYMTSMKRFKNESDPEGALW

>DENV1_ABO38807_French_Guiana

MNNQRKKTGRPSFNMLKRARNRVSTGSQLAKRFSKGLLSGQGPMKLVMAFIAFLRFLAIPPTAGILARWSSFKKNGAIKVLRGFKKEISSMLNIMNRRKRSVTMLLMLLPTVLAFHLTTRGGEPHMIVSKQERGKSLLFKTSAGVNMCTLIAMDLGELCEDTMTYKCPRITEAEPDDVDCWCNATDTWVTYGTCSQTGEHRRDKRSVALAPHVGLGLETRTETWMSSEGAWKQIQKVETWALRHPGFTVIALFLAHAIGTSITQKGIIFILLMLVTPSMAMRCVGIGNRDFVEGLSGATWVDVVLEHGSCVTTMAKNKPTLDIELLKTEVTNPAVLRKLCIEAKISNTTTDSRCPTQGEATLVEEQDANFVCRRTVVDRGWGNGCGLFGKGSLLTCAKFKCVTKLEGKIVQYENLKYSVIVTVHTGDQHQVGNETTEHGTIATITPQAPTSEIQLTDYGTLTLDCSPRTGLDFNEVVLLTMKEKSWLVHKQWFLDLPLPWTSGASTSQETWNRQDLLVTFKTAHAKKQEVVVLGSQEGAMHTALTGATEIQTSGTPTIFAGHLKCRLKMDKLTLKGMSYVMCTGSFKLEKEVAETQHGTVLVQVKYEGTDAPCKIPFSTQDEKGVTQNGRLITANPIVTDKEKPVNIETEPPFGESYIIVGAGEKALKLSWFKKGSSIGKMFEATARGARRMAILGDTAWDFGSIGGVFTSVGKLVHQVFGTAYGVLFSGVSWTMKIGIGILLTWLGLNSRSASLSMTCIAVGMVTLYLGVMVQADSGCVINWKGRELKCGSGIFVTNEVHTWTEQYKFQADSPKRLSAAIGKAWEEGVCGIRSATRLENIMWKQISNELNHILLENDMKFTVVVGDANGILAQGKKMIRPQPMEHKYSWKSWGKAKIIGADTQNTTFIIDGPDTPECPDDQRAWNIWEVEDYGFGIFTTNIWLKLRDSYTQMCDHRLMSAAIKDSKAVHADMGYWIESEKNETWKLARASFIEVKTCIWPKSHTLWSNGVLESEMIIPKIYGGPISQHNYRPGYFTQTAGPWHLGKLELDFDLCEGTTVVVDEHCGNRGPSLRTTTVTGKIIHEWCCRSCTLPPLRFRGEDGCWYGMEIRPVKEKEENLVRSMVSAGSGEVDSFSLGILCVSIMIEEVMRSRWSRKMLMTGTLAVFLLLIMGQLTWNDLIRLCIMVGANASDRMGMGTTYLALMATFKMRPMFAVGLLFRRLTSREVLLLTIGLSLVASVELPNSLEELGDGLAMGIMMLKLLTEFQPHQLWTTLLSLTFIKTTLSLDYAWKTMAMVLSIVSLFPLCLSTTSQKTTWLPVLLGSFGCKPLTMFLITENKIWGRKSWPLNEGIMAIGIVSILLSSLLKNDVPLAGPLIAGGMLIACYVISGSSADLSLEKAAEVSWEEEAEHSGTSHNILVEVQDDGTMKIKDEERDDTLTILLKATLLAVSGVYPMSIPATLFVWYFWQKKKQRSGVLWDTPSPPEVERAVLDDGIYRILQRGLLGRSQVGVGVFQDGVFHTMWHVTRGAVLMYQGKRLEPSWASVKKDLISYGGGWRFQGSWNTGEEVQVIAVEPGKNPKNVQTTPGTFKTPEGEVGAIALDFKPGTSGSPIVNREGKIVGLYGNGVVTTSGTYVSAIAQAKASQEGPLPEIEDEVFKKRNLTIMDLHPGSGKTRRYLPAIVREAIKRKLRTLILAPTRVVASEMAEALKGMPIRYQTTAVKSEHTGREIVDLMCHATFTMRLLSPVRVPNYNMIIMDEAHFTDPASIAARGYISTRVGMGEAAAIFMTATPPGSVEAFPQSNAVIQDEERDIPERSWNSGYDWITDFPGKTVWFVPSIKSGNDIANCLRKNGKRVIQLSRKTFDTEYQKTKNNDWDYVVTTDISEMGANFRADRVIDPRRCLKPVISKDGPERVILAGPMPVTVASAAQRRGRIGRNQNKEGDQYVYMGQPLNNDEDHAHWTEAKMLLDNINTPEGIIPALFEPEREKSAAIDGEYRLRGEARKTFVELMRRGDLPVWLSYKVASEGFQYSDRRWCFDGERNNQVLEENMDVEIWTKEGERKKLRPRWLDARTYSDPLALREFKEFAAGRRSVSGDLILEIGKLPQHLTLRAQNALDNLVMLHNSEQGGKAYRHAMEELPDTIETLMLLALIAVLTGGVMLFFLSGKGLGKTSIGLLCVMASSALLWMASVEPHWIAASIILEFFLMVLLIPEPDRQRTPQDNQLAYVVIGLLFMILTVAANEMGLLETTKKDLGIGHVAAENHHHVTMLDVDLRPASAWTLYAVATTVITPMMRHTIENTTANISLTAIANQAAILMGLDKGWPISKMDIGVPLLALGCYSQVNPLTLTAAVLMLVAHYAIIGPGLQAKATREAQKRTAAGIMKNPTVDGIVAIDLDPVVYDAKFEKQLGQIMLLILCTSQILLMRTTWALCESITLATGPLTTLWEGSPGKFWNTTIAVSMANIFRGSYLAGAGLAFSLMKSLGGGRRGTGAQGETLGEKWKRQLNQLSKSEFNTYKRSGIMEVDRSEAKEGLKRGETTKHAVSRGTAKLRWFVERNLVKPEGKVIDLGCGRGGWSYYCAGLKKVTEVKGYTKGGPGHEEPIPMATYGWNLVKLHSGKDVFFIPPEKCDTLLCDIGESSPNPTIEEGRTLRVLKMVEPWLRGNQFCIKILNPYMPSVVETLEQMQRKHGGMLVRNPLSRNSTHEMYWVSCGTGNIVSAVNMTSRMLLNRFTMAHRKPTYERDVDLGAGTRHVAVEPEVANLDIIGQRIENIKNEHKSTWHYDEDNPYKTWAYHGSYEVKPSGSASSMVNGVVRLLTKPWDVIPMVTQIAMTDTTPFGQQRVFKEKVDTRTPRAKRGTAQIMEMTAKWLWGFLSRNKKPRICTREEFIRKVRSNAAIGAVFVDENQWNSAKEAVEDERFWDLVHRERELHKQGKCATCVYNMMGKREKKLGEFGKAKGSRAIWYMWLGARFLEFEALGFMNEDHWFSRENSLSGVEGEGLHKLGYILRDISKIPGGNMYADDTAGWDTRITEDDLQNEAKITDIMEPEHALLATSIFKLTYQNKVVRVQRPAKNGTVMDVISRRDQRGSGQVGTYGLNTFTNMEVQLIRQMESEGIFLPSELETPNLAERVLDWLEKHGAERLKRMAISGDDCVVKPIDDRFATALIALNDMGKVRKDIPQWEPSKGWNDWQQVPFCSHHFHQLIMKDGREIVVPCRNQDELVGRARVSQGAGWSLRETACLGKSYAQMWQLMYFHRRDLRLAANAICSAVPVDWVPTSRTTWSIHAHHQWMTTEDMLSVWNRVWIEENPWMEDKTHVSSWEEVPYLGKREDQWCGSLIGLTARATWATNIQVAINQVRRLIGNENYLDYMTSMKRFKNESDPEGALW

>DENV1__BAD42414_Indonesia

MNNQRKKTGRPSFNMLKRARNRVSTVSQLAKRFSKGLLSGQGPMKLVMAFIAFLRFLAIPPTAGILARWGSFKKNGAIKVLRGFKKEISNMLNIMNRRKRSVTMLFMLLPTALAFHLTTRGGEPHMIVSKQERGKSLLFKTSAGVNMCTLIAMDLGELCEDTMTYKCPRITETEPDDVDCWCNATETWVTYGTCSQTGEHRRDKRSVALAPHVGLGLETRTETWMSSEGAWRQIQKVETWALRHPGFTVMALFLAHAIGTSITQKGIIFILLMLVTPSMAMRCVGIGNRDFVEGLSGATWVDVVLEHGSCVTTMAKDKPTLDIELLKTEVTNPAVLRKLCIEAKISNTTTDSRCPTQGEATLVEEQDTNFVCRRTFVDRGWGNGCGLFGKGSLITCAKFKCVTKLEGKIVQYENLKYSVIVTVHTGDQHQVGNETTEHGTTAIITPQAPTSEIQLTDYGALTLDCSPRTGLDFNEMVLLTMKEKSWLVHKQWFLDLPLPWTSGASTSQETWNRQDLLVTFKTAHAKKQEVVVLGSQEGAMHTALTGATEIQTSGTTTIFAGHLKCRLKMDKLTLKGVSYVMCTGSFKLEKEVAETQHGTVLVQVKYEGTDAPCKIPFSSQDEKGVIQNGRLITANPIVTDKEKPVNIEAEPPFGESYIVVGAGEKALKLSWFKKGSSIGKMFEATARGARRMAILGDTAWDFGSIGGVFTSVGKLVHQIFGTAYGVLFSGVSWTMKIGIGILLTWLGLNSRSTSLSMTCIAVGMVTLYLGVMVQADSGCVINWKGRELKCGSGIFVTNEVHTWTEQYKFQADSPKRLSAAIGKAWEEGVCGIRSATRLENIMWKQISNELNHILLENDMKFTVVVGDVSGILAQGKKMIRPQPMEHKYSWKSWGKAKIIGADVQNITFIIDGPNTSECPDNQRAWNIWEVEDYGFGIFTTNIWLKLRDSYTQVCDHRLMSAAIKDSKAVHADMGYWIESEKNETWKLARASFIEVKTCIWPKSHTLWSNGVLESEMIIPKIYGGPVSQHNYRPGYFTQTAGPWHLGKLELDFDLCEGTTVVVDENCGNRGPSLRTTTVTGKTIHEWCCRSCTLPPLRFKGEDGCWYGMEIRPVKEKEENLVKSMVSAGSGEVDSFSLGLLCISIMIEEVMRSRWSRKMLMTGTLAVFLLLTMGQLTWNDLIRLCIMVGANASDMMGMGTTYLALMATFRMRPMFAVGLLFRRLTSREVLLLTVGLSLVASVELPNSLEELGDGLAMGIMMLKLLTDFQPHQLWATLLSLTFVKTTFSLHYAWKTMAMILSIVSLLPLCLSTTSQKTTWLPVLLGSLGCKPLTMLLITENKIWGRRSWPLNEGIMAIGIVSILLSSLLKNDVPLAGPLIAGGMLIACYVISGSSADLSLEKAAEVSWEDEAEHSGASHNILVEVQDDGTMKIKDEERDDTLTILLKATLLAISGVYPMSIPATLFVWYFWQKKKQRSGVLWDTPSPPEVERAVLDNGIYRILQRGLLGRSQVGVGVFQEGVFHTMWHVTRGAVLMYQGKRLEPSWASVKRDLISYGGGWRFQGSWNTGEEVQVIAVEPGKNPKNVQTTPGTFKTPEGEVGAIALDFKPGTSGSPIVNREGKIVGLYGNGVVTTSGTYVSAIAQAKASQEGPLPEIEDEVFRKRNLTIMDLHPGSGKTRRYLPAIVREAIKRKLRTLVLAPTRVVASEMAEALKGMPIRYQTTAVKSEHTGKEIVDLMCHATFTMRLLSPVRVPNYNMIIMDEAHFTDPASIAARGYISTRVGMGEAAAIFMTATPPGSVEAFPQSNAVIQDEERDIPERSWNSGYEWITDFPGKTVWFVPSIKSGNDIANCLRKNGKRVVQLSRKTFDTEYQKTKNNDWDYVVTTDISEMGANFRADRVIDPRRCLKPVILKDGPERVILAGPMPVTVASAAQRRGRIGRNPNKEGDQYVYMGQPLNNDEDHAHWTEAKMLLDNINTPEGIIPALFEPEREKSAAIDGEYRLRGEARKTFVELMRRGDLPVWLSYKVASEGFQYSDRRWCFDGERNNQVLEENMDVEIWTKEGERKKLRPRWLDARTYSDPLALREFKEFAAGRRSVSGDLILEIGKLPQHLTQKAQNALDNLVMLHNSEQGGKAYRHAMEELPDTIETLMLLALIAVLTGGVTLFFLSGRGLGKTSIGLLCVMASSALLWMASVEPHWIAASIILEFFLMVLLIPEPDRQRTPQDNQLAYVVIGLLFMILTVAANEMGLLETTKKDLGIGHAAAENHHHAAMLDVDLHPASAWTLYAVATTIITPMMRHTIENTTANISLTAIANQAAILMGLDKGWPISKMDIGVPLLALGCYSQVNPLTLTAAVLMLVAHYAIIGPGLQAKATREAQKRTAAGIMKNPTVDGIVAIDLDPVVYDAKFEKQLGQIMLLILCTSQILLMRTTWALCESITLATGPLTTLWEGSPGKFWNTTIAVSMANIFRGSYLAGAGLAFSLMKSLGGGRRGTGAQGETLGEKWKRQLNQLSKSEFNIYKRSGIIEVDRSEAKEGLKRGETTKHAVSRGTAKLRWFVERNLVKPEGKVIDLGCGRGGWSYYCAGLKKVTEVKGYTKGGPGHEEPIPMATYGWNLVKLHSGKDVFFTPPEKCDTLLCDIGESSPNPTIEEGRTLRVLKMVEPWLRGNQFCIKILNPYMPSVVETLEKMQRKHGGMLVRNPLSRNSTHEMYWVSCGTGNIVSAVNMTSRMLLNRFTMAHRKPTYERDVDLGAGTRHVAVEPEVANLDIIGQRIENIKNEHKSTWHYDEDNPYKTWAYHGSYEVKPSGSASSMVNGVVRLLTKPWDVIPMVTQIAMTDTTPFGQQRVFKEKVDTRTPKAKRGTAQIMEVTARWLWGFLSRNKKPRICTREEFTRKVRSNAAIGAVFVDENQWNSAKEAVEDERFWDLVHRERELHKQGKCATCVYNMMGKREKKLGEFGKAKGSRAIWYMWLGARFLEFEALGFMNEDHWFSRENSLSGVEGEGLHKLGYILRDISKIPGGNMYADDTAGWDTRITEDDLQNEAKITDIMEPEHALLATSIFKLTYQNKVVRVQRPAKNGTVMDVISRRDQRGSGQVGTYGLNTFTNMEAQLIRQMESEGIFSPSELETPNLAERVFDWLEKHGIERLKRMAISGDDCVVKPIDDRFATALTALNDMGKVRKDIPQWEPSKGWNDWQQVPFCSHHFHQLIMKDGREIVVPCRNQDELVGRARVSQGAGWSLRETACLGKSYAQMWQLMYFHRRDLRLAANAICSAVPVDWVPTSRTTWSIHAHHQWMTTEDMLSVWNRVWIEENPWMEDKTHVSSWEDVPYLGKREDQWCGSLIGLTARATWATNIQVAINQVRRLIGNENYLDYMTSMKRFKNESDPEGALW

>DENV1_AIN75463_Germany

MNNQRKKTARPSFNMLKRARNRVSTVSQLAKRFSKGLLSGQGPMKLVMAFIAFLRFLAIPPTAGILARWGSFKKNGAIKVLRGFKKEISNMLNIMNRRRKSVTMLLMLMPTALAFHLTTRGGEPHMIVSKQERGKSLLFKTSAGVNMCTLIAMDLGELCEDTITYKCPRITEAEPDDVDCWCNATDTWVTYGTCSQTGEHRRDKRSVALAPHVGLGLETRTETWMSSEGAWKQIQRVETWALRHPGFTVIALFLAHAIGTSITQKGIIFILLMLVTPSMAMRCVGIGSRDFVEGLSGATWVDVVLEHGSCVTTMAKDKPTLDIELLKTEVTNPAVLRKLCIEAKISNTTTDSRCPTQGEATLVEEQDANFVCRRTFVDRGWGNGCGLFGKGSLLTCAKFKCVTKLEGKIVQNENLKYSVIVTVHTGDQHQVGNESTEHGTTATITPQAPTTEIQLTDYGALTLDCSPRTGLDFNEMVLLTMKEKSWLVHKQWFLDLPLPWTSGASTSQETWNRQDLLVTFKTAHAKKQEVVVLGSQEGAMHTALTGATEIQMSGTTTIFAGHLKCRLKMDKLTLKGMSYVMCTGSFKLEKEVPETQHGTVLVQIKYEGTDAPCKIPFSTQDEKGVTQNGRLITANPIVTDKEKPVNIEAEPPFGESYIVIGAGEKALKLSWFKKGSSIGKMFEATARGARRMAILGDTAWDFGSIGGVFTSVGKLVHQIFGTAYGVLFSGVSWTMKIGIGVLLTWLGLNSRSTSLSMTCIAVGLVTLYLGVMVQADSGCVINWKGRELKCGSGIFVTNEVHTWTEQYKFQADSPKRLSAAIGKAWEEGVCGIRSATRLENIMWKQISNELNHILLENDMKFTVVVGDVAGILAQGKKMIRPQPMEHKYSWKNWGKAKIIGADVQNTTFIIDGPNTPECPDDQRAWNIWEVEDYGFGIFTTNIWLKLRDSYTQVCDHRLMSAAIKDSKAVHADMGYWIESEKNDTWKLARASFIEVKTCIWPKSHTLWSNGVLESEMIIPKIYGGPISQHNYRPGYFTQTAGPWHLGKLELDFDLCEGTTVVVDEHCGNRGPSLRTTTVTGKIIHEWCCRSCTLPPLRFRGEDGCWYGMEIRPVKEKEENLVKSMVSAGSGEVDSFSLGLLCISIMIEEVMRSRWSRKMLMTGTLAVFFLLIMGQLTWNDLIRLCTMVGANASDRMGMGTTYLALMATFKMRPMFAVGLLFRRLTSREVLLLTIGLSLVASVELPNSLEELGDGLAMGIMILKLLTDFQSHQLWATLLSMTFIKTTFSLHYAWKTMAMVLSIVSLFPLCLSTTSQKTTWLPVLLGSLGCKPLTMFLIAENKIWGRKSWPLNEGIMAVGIVSILLSSLLKNDVPLAGPLIAGGMLIACYVISGSSADLSLEKAAEVSWEEEAEHSGASHNILVEVQDDGTMKIKDEERDDTLTILLKATLLAVSGVYPLSIPATLFVWYFWQKKKQRSGVLWDTPSPPKVERAVLDDGIYRIMQRGLLGRSQVGVGVFQENVFHTMWHVTRGSVLMYQGKRLEPSWASVKKDLISYGGGWRLQGSWNTGEEVQVIAVEPGKNPKNVQTAPGTFKTPEGEVGAIALDFKPGTSGSPIVNREGKIVGLYGNGVVTTSGTYVSAIAQAKASQEGPLPEIEDEVFRKRNLTIMDLHPGSGKTRRYLPAIVREAIKRKMRTLILAPTRVVASEMAEALKGMPIRYQTTAVKSEHTGKEIVDLMCHATFTMRLLSPVRVPNYNMIIMDEAHFTDPSSIAARGYISTRVGMGEAAAIFMTATPPGSVEAFPQSNAVIQDEERDIPERSWNSGYEWITDFPGKTVWFVPSIKAGNDIANCLRKNGKRVIQLSRKTFDTEYQKTKNNDWDYVVTTDISEMGANFRADRVIDPRRCLKPVILKDGPERVILAGPMPVTVASAAQRRGRIGRNHNKEGDQYIYMGQPLNNDEDHAHWTEAKMLLDNINTPEGIIPALFEPEREKSAAIDGEYRLRGEARKTFVELMRRGDLPVWLSYKVASEGFQYSDRRWCFDGERKNQVLEENMDVEIWTKEGERKKLRPRWLDARTYSDPLAMREFKEFAAGRRSVSGDLILEIGKLPQRLTQRAQNALDNLVMLHNSEQGGRAYRHAMEELPDTIETLMLLALIAVLTGGVTLFFLSGKGLGKTSIGLLCVMASSALLWMASVEPHWIAASIILEFFLMVLLIPEPDRQRTPQDNQLAYVVIGLLFMILTVAANEMGLLETTKKDLGIGHVAVENHHHAAMLDIDLHPASAWTLYAVATTIITPMMRHTIENTTANISLTAIANQAAILMGLDKGWPISKMDIGVPLLALGCYSQVNPLTLTAAVLMLVAHYAIIGPGLQAKATREAQKRTAAGIMKNPTIDGIVAIDLDPVVYDAKFEKQLGQIMLLILCTSQILLMRTTWALCESITLATGPLTTLWEGSPGKFWNTTIAVSMANIFRGSYLAGAGLAFSLMKSLGGGRRGTGAQGETLGEKWKRQLNQLSKSEFNTYKRSGIMEVDRSEAKEGLKRGETTKHAVSRGTAKLRWFVERNLVKPEGKVIDLGCGRGGWSYYCAGLKKVTEVKGYTKGGPGHEEPIPMATYGWNLVKLHSGKDVFFIPPEKCDTLLCDIGESSPNPTIEEGRTLRVLKMVEPWLRGNQFCIKILNPYMPSVVETLEQMQRKHGGMLVRNPLSRNSTHEMYWVSCGTGNIVSAVNMTSRMLLNRFTMAHRKPTYERDVDLGAGTRHVAVEPEVANLDIIGQRIENIKHEHKSTWHYDEDNPYKTWAYHGSYEVKPSGSASSMVNGVVKLLTKPWDVIPMVTQIAMTDTTPFGQQRVFKEKVDTRTPKAKRGTAQIMEVTARWLWSFLSRNKKPRICTREEFTRKVRSNAAIGAVFVDENQWNSAKEAVEDDRFWDLVHRERELHKQGKCATCVYNMMGKREKKLGEFGKAKGSRAIWYMWLGARFLEFEALGFMNEDHWFSRENSLSGVEGEGLHKLGYILRDISKIPGGNMYADDTAGWDTRITEDDLQNEAKITDIMEPEHALLAKSIFKLTYQNKVVRVQRPAKNGTVMDVISRRDQRGSGQVGTYGLNTFTNMEAQLIRQMESEEIFSPNELETPNLAERVLDWLEKYGVERLKRMAISGDDCVVKPIDDRFATALTALNDMGKVRKDIPQWEPSKGWNDWQQVPFCSHHFHQLIMKDGREIVVPCRNQDELVGRARVSQGAGWSLRETACLGKSYAQMWQLMYFHRRDLRLAANAICSAVPVDWVPTSRTTWSIHAHHQWMTTEDMLSVWNRVWIEENPWMEDKTRVSRWEDVPYLGKREDQWCGSLIGLTARATWATNIQVAINQVRRLIGNENYSDYMTSMKRFKNESEPEGALW

>DENV2_BAX09288_Thailand

MNNQRKKAKNTPFNMLKRERNRVSTVQQLTKRFSLGMLQGRGPLKLFMALVAFLRFLTIPPTAGILKRWGTIKKSKAINVLRGFRKEIGRMLNILNRRRRSAGMIIMLIPTVMAFHLTTRNGEPHMIVGIQEKGKSLLFKTEDGVNMCTLMAMDLGELCEDTITYKCPLLRQNEPEDIDCWCNSTSTWVTYGTCTTTGEHRREKRSVALVPHVGMGLETRTETWMSSEGAWKHAQRIETWILRHPGFTIMAAILAYTIGTTHFQRVLIFILLTAVAPSMTMRCIGISNRDFVEGVSGGSWVDIVLEHGSCVTTMAKNKPTLDFELIKTEAKQPATLRKYCIEAKLTNTTTESRCPTQGEPSLKEEQDKRFVCKHSMVDRGWGNGCGLFGKGGIVTCAMFTCKKNMEGKIVQPENLEYTIVVTPHSGEEHAVGNDTGKHGKEIKVTPQSSITEAELTGYGTVTMECSPRTGLDFNEMVLLQMENKAWLVHRQWFLDLPLPWLPGADKQESNWIQKETLVTFKNPHAKKQDVVVLGSQEGAMHTALTGATEIQMSSGNLLFTGHLKCRLRMDKLQLKGMSYSMCTGKFKVVKEIAETQHGTIVIRVQYEGDGSPCKIPFEIMDLEKRYVLGRLITVNPIVTEKDSPVNIEAEPPFGDSYIIIGVEPGQLKLNWFKKGSSIGQMFETTMRGAKRMAILGDTAWDFGSLGGVFTSIGKALHQVFGAIYGAAFSGVSWTMKILIGVIITWIGMNSRSTSLSVSLVLVGIVTLYLGVMVEADSGCVVSWKNKELKCGSGIFITDNVHTWTEQYKFQPEAPSKLASAIQKAQEEGICGIRSVTRLENLMWKQITPELNHILAENEVKLTIMTGDIKGIMQAGKRSLRPQPTELKYSWKTWGKAKMLSTESYNQTFLIDGPETAECPNTNRAWNSLEVEDYGFGVFTTNIWLKLKEKQDAFCDSKLMSAAIKDNRAVHADMGYWIESALNDTWKIEKASFIEVKNCHWPKSHTLWSNGVLESEMIIPKNLAGPVSQHNYRPGYHTQIAGPWHLGKLEMDFDFCDGTTVIVTEDCGNRGPSLRTTTASGKLITEWCCRSCTLPPLRYRGEDGCWYGMEIRPLKEKEENLVNSLVTAGHGQVDNFSLGVLGMALFLEEMLRTRVGTKHAILLVAVSFVTLITGNMSFKDLGRVVVMVGATMADDIGMGVTYLALLAAFKVRPTFAAGLLLRKLTSKELMMTTIGIVLLSQSTIPETVLELTDALALGMMVLKIVRNMEKYQLAVTIMAILCVPNAVILQNAWKVSCTILAVVSVSPLLLTSSQQKTDWIPLALTIKGLNPTAIFLTTLSRTNKKRSWPLNEAIMAVGMVSILASSLLKNDIPMTGPLVAGGLLTVCYVLTGRSADLELERATDVKWEDQAKISGSSPILSITISEDGSMSIKNEEEEQTLTILIRTGLLVISGLFPISIPITAAAWYLWEVKKQRAGVLWDVPSPPPMGKAELEDGAYRIKQKGILGYSQIGAGVYKEGTFHTMWHVTRGAVLMHKGKRIEPSWADVKKDLISYGGGWKLEGEWKEGEEVQVLALEPGKNPRAVQTKPGLFKTNTGTIGAVSLDFSPGTSGSPIIDKKGKVVGLYGNGVVTRSGAYVSAIAQTEKSIEDNPEIEDDIFRKRRLTIMDLHPGAGKTKRYLPAIVREAIKRGLRTLILAPTRVVAAEMEEALRGLPIRYQTPAIRAEHTGREIVDLMCHATFTMRLLSPVRVPNYNLIIMDEAHFTDPASIAARGYISTRVEMGEAAGIFMTATPPGSRDPFPQSNAPIIDEEREIPERSWNSGHEWVTDFKGKTVWFVPSIKAGNDIAACLRKNGKKVIQLSRKTFDSEYVKTRTNDWDFVVTTDISEMGANFKAERVIDPRRCMKPVILTDGEERVILAGPMPVTHSSAAQRRGRIGRNPKNENDQYIYMGEPLENDEDCAHWKEAKMLLDNINTPEGIIPSMFEPEREKVDAIDGEYRLRGEARKTFVDLMRRGDLPVWLAYKVAAEGINYADRRWCFDGIKNNQILEENVEVEIWTKEGERKKLKPRWLDARIYSDPLALKEFKEFAAGRKSLTLNLITEMGRLPTFMTQKTRDALDNLAVLHTAEAGGRAYNHALSELPETLETLLLLTLLATVTGGIFLFLMSGRGIGKMTLGMCCIITASILLWYAQIQPHWIAASIILEFFLIVLLIPEPEKQRTPQDNQLTYVVIAILTVVAATMANEMGFLEKTKKDLGLVSIATQQPESNILDIDLRPASAWTLYAVATTFVTPMLRHSIENSSVNVSLTAIANQATVLMGLGKGWPLSKMDIGVPLLAIGCYSQVNPITLTAALFLLVAHYAIIGPGLQAKATREAQKRAAAGIMKNPTVDGITVIDLDPIPYDPKFEKQLGQVMLLILCVTQVLMMRTTWALCEALTLATGPISTLWEGNPGRFWNTTIAVSMANIFRGSYLAGAGLLFSIMKNTTNTRRGTGNIGETLGEKWKSRLNALGKSEFQIYKKSGIQEVDRTLAKEGIKRGETDHHAVSRGSAKLRWFVERNMVTPEGKVVDLGCGRGGWSYYCGGLKNVREVKGLTKGGPGHEEPIPMSTYGWNLVRLQSGVDVFFIPPEKCDTLLCDIGESSPNPTVEAGRTLRVLNLVENWLNNNTQFCIKVLNPYMPSVIEKMEALQRKYGGALVRNPLSRNSTHEMYWVSNASGNIVSSVNMISRMLINRFTMRHKKATYEPDVDLGSGTRNIGIESEIPNLDIIGKRIEKIKQEHETSWHYDQDHPYKTWAYHGSYETKQTGSASSMVNGVVRLLTKPWDVLPTVTQMAMTDTTPFGQQRVFKEKVDTRTQEPKEGTKKLMKITAEWLWKELGKKKTPRMCTREEFTRKVRSNAALGAIFTDENKWKSAREAVEDSRFWELVDKERNLHLEGKCETCVYNMMGKREKKLGEFGKAKGSRAIWYMWLGARFLEFEALGFLNEDHWFSRENSLSGVEGEGLHKLGYILRDVSKKEGGAMYADDTAGWDTRITLEDLKNEEMVTNHMEGEHKKLAEAIFKLTYQNKVVRVQRPTPRGTVMDIISRRDQRGSGQVGTYGLNTFTNMEAQLIRQMEGEGVFKNIQHLTVTEEVAVQNWLARVGRERLSRMAISGDDCVVKPLDDRFANALTALNDMGKIRKDIQQWEPSKGWNDWTQVPFCSHHFHELIMKDGRVLVVPCRNQDELIGRARISQGAGWSLRETACLGKSYAQMWSLMYFHRRDLRLAANAICSAVPSHWVPTSRTTWSIHAKHEWMTTEDMLTVWNRVWIRENPWMEDKTPVESWEEIPYLGKREDQWCGSLIGLTSRATWAKNIQVAINQVRSLIGNEEYTDYMPSMKRFRREEEEAGVLW

>DENV2_BAD36760_Dominican_Republic

MNNQRKKARSTPFNMLKRERNRVSTVQQLTKRFSLGMLQGRGPLKLFMALVAFLRFLTIPPTAGILKRWGTIKKSKAINVLRGFRKEIGRMLNILNRRRRTAGVIVMLIPTAMAFHLTTRNGEPHMIVGRQEKGKSLLFKTEDGVNMCTLMAIDLGELCEDTITYKCPLLRQNEPEDIDCWCNSTSTWVTYGTCTTTGEHRREKRSVALVPHVGMGLETRTETWMSSEGAWKHVQRIETWILRHPGFTIMAAILAYTIGTTHFQRALIFILLTAVAPSMTMRCIGISNRDFVEGVSGGSWVDIVLEHGSCVTTMAKNKPTLDFELIKTEAKQPATLRKYCIEAKLTNTTTESRCPTQGEPSLNEEQDKRFICKHSMVDRGWGNGCGLFGKGGIVTCAMFTCKKNMEGKIVQPENLEYTIVITPHSGEEHAVGNDTGKHGKEIKITPQSSTTEAELTGYGTVTMECSPRTGLDFNEMVLLQMEDKAWLVHRQWFLDLPLPWLPGADTQGSNWIQKETLVTFKNPHAKKQDVVVLGSQEGAMHTALTGATEIQMSSGNLLFTGHLKCRLRMDKLQLKGMSYSMCTGKFKIVKEIAETQHGTIVIRVQYEGDGSPCKIPFEITDLEKRHVLGRLITVNPIVTEKDSPVNIEAEPPFGDSYIIVGVEPGQLKLNWFKKGSSIGQMFETTMRGAKRMAILGDTAWDFGSLGGVFTSIGKALHQVFGAIYGAAFSGVSWTMKILIGVIITWIGMNSRSTSLSVSLVLVGVVTLYLGAMVQADSGCIVSWKNKELKCGSGIFITDNVHTWTEQYKFQPESPSKLASAIQKAHEEGICGIRSVTRLENLMWKQITPELNHILSENEVKLTIMTGDIKGIMQAGKRSLRPQPTELKYSWKTWGKAKMLSTESHNQTFLIDGPETAECPNTNRAWNSLEVEDYGFGVFTTNIWLKLREKQDVFCDSKLMSAAIKDNRAVHADMGYWIESALNDTWKMEKASFIEVKSCHWPKSHTLWSNGVLESEMIIPKNFAGPVSQHNYRPGYHTQTAGPWHLGKLEMDFDFCEGTTVVVTEDCGNRGPSLRTTTASGKLITEWCCRSCTLPPLRYRGEDGCWYGMEIRPLKEKEENLVNSLVTAGHGQIDNFSLGVLGMALFLEEMLRTRVGTKHAILLVALSFVTLITGNMSFRDLGRVMVMVGATMTDDIGMGVTYLALLAAFKVRPTFAAGLLLRKLTSKELMMATIGIALLSQSTLPETILELTDALALGMMVLKIVRNMEKYQLAVTIMAISCVPNAVILQNAWKVSCTILAAVSVSPLLLTSSQQKADWIPLALTIKGLNPTAIFLTTLSRTSKKRSWPLNEAIMAVGMVSILASSLLKNDIPMTGPLVAGGLLTVCYVLTGRSADLELERAADVKWEDQAEISGSSPILSITISEDGSMSIKNEEEEQTLTILIRTGLLVISGVFPVSIPITAAAWYLWEVKKQRAGVLWDVPSPPPVGKAELEDGAYRIKQRGILGYSQIGAGVYKEGTFHTMWHVTRGAVLMHRGKRIEPSWADVKKDLISYGGGWKLEGEWKEGEEVQVLALEPGKNPRAVQTKPGIFKTNTGTIGAVSLDFSPGTSGSPIVDRKGKVVGLYGNGVVTRSGAYVSAIAQTEKSIEDNPEIEDDIFRKKRLTIMDLHPGAGKTKRYLPAIVREAIKRGLRTLILAPTRVVAAEMEEALRGLPIRYQTPAIRAEHTGREIVDLMCHATFTMRLLSPVRVPNYNLIIMDEAHFTDPASIAARGYISTRVEMGEAAGIFMTATPPGSRDPFPQSNAPIMDEEREIPERSWNSGHEWVTDFKGKTVWFVPSIKAGNDIAACLRKNGKKVIQLSRKTFDSEYVKTRANDWDFVVTTDISEMGANFKAERVIDPRRCMKPVILTDGEERVILAGPMPVTHSSAAQRRGRIGRNPKNENDQYIYMGEPLENDEDCAHWKEAKMLLDNINTPEGIIPSMFEPEREKVDAIDGEYRLRGEARKTFVDLMRRGDLPVWLAYKVAAEGINYADRKWCFDGIKNNQILEENVEVEIWTKEGERKKLKPRWLDARIYSDPLALKEFKEFAAGRKSLTLNLITEMGRLPTFMTQKARNALDNLAVLHTAEVGGRAYNHALSELPETLETLLLLTLLATVTGGIFLFLMSGKGIGKMTLGMCCIITASILLWYAQIQPHWIAASIILEFFLIVLLIPEPEKQRTPQDNQLTYVVIAILTVVAATMANEMGFLEKTKKDLGFGSITTQESESNILDIDLRPASAWTLYAVATTFVTPMLRHSIENSSVNVSLTAIANQATVLMGLGKGWPLSKMDIGVPLLAIGCYSQVNPITLTAALLLLVAHYAIIGPGLQAKATREAQKRAAAGIMKNPTVDGITVIDLEPIPYDPKFEKQLGQVMLLILCVTQVLMMRTTWALCEALTLATGPISTLWEGNPGRFWNTTIAVSMANIFRGSYLAGAGLLFSIMKNTTNTRRGTGNVGETLGEKWKSRLNALGKSEFQIYKKSGIQEVDRTLAKEGIKRGETDHHAVSRGSAKLRWFVERNMVTPEGKVVDLGCGRGGWSYYCGGLKNVREVKGLTKGGPGHEEPIPMSTYGWNLVRLQSGVDVFFTPPEKCDTLLCDIGESSPNPTIEAGRTLRVLNLVENWLNNNTQFCIKVLNPYMPSVIEKMETLQRKYGGALVRNPLSRNSTHEMYWVSNATGNIVSSVNMISRMLINRFTMKHKKATYEPDVDLGSGTRNIGIESEIPNLDIIGKRIEKIKQEHETSWHYDQDHPYKTWAYHGSYETKQTGSASSMVNGVVRLLTKPWDVVPMVTQMAMTDTTPFGQQRVFKEKVDTRTQEPKEGTKKLMKITAEWLWKELGKEKTPRMCTREEFTRKVRSNAALGAIFTDENKWKSAREAVEDGRFWELVDRERNLHLEGKCETCVYNMMGKREKKLGEFGKAKGSRAIWYMWLGARFLEFEALGFLNEDHWFSRGNSLSGVEGEGLHRLGYILREVGKKEGGAMYADDTAGWDTRITLEDLKNEEMVTNHMKGEHKKLAEAIFKLTYQNKVVRVQRPTPRGTVMDIISRKDQRGSGQVGTYGLNTFTNMEAQLIRQMEGEGIFKSIQHLTATEEIAVQNWLARVGRERLSRMAISGDDCVVKPIDDRFASALTALNDMGKVRKDIQQWEPSRGWNDWTQVPFCSHHFHELVMKDGRVLVVPCRNQDELIGRARISQGAGWSLKETACLGKSYAQMWTLMYFHRRDLRLAANAICSAVPSHWVPTSRTTWSIHAKHEWMTTEDMLAVWNRVWIQENPWMEDKTPVESWEEVPYLGKREDQWCGSLIGLTSRATWAKNIQTAINQVRSLIGNEEYTDYMPSMKRFRREEEEAGVLW

>DENV2_BAD36759_Dominican_Republic

MNNQRKKARSTPFNMLKRERNRVSTVQQLTKRFSLGMLQGRGPLKLFMALVAFLRFLTIPPTAGILKRWGTIKKSKAINVLRGFRKEIGRMLNILNRRRRTAGVIVMLIPTAMAFHLTTRNGEPHMIVGRQEKGKSLLFKTEDGVNMCTLMAIDLGELCEDTITYKCPLLRQNEPEDIDCWCNSTSTWVTYGTCTTTGEHRREKRSVALVPHVGMGLETRTETWMSSEGAWKHVQRIETWILRHPGFTIMAAILAYTIGTTHFQRALIFILLTAVAPSMTMRCIGISNRDFVEGVSGGSWVDIVLEHGSCVTTMAKNKPTLDFELIKTEAKQPATLRKYCIEAKLTNTTTESRCPTQGEPSLNEEQDKRFICKHSMVDRGWGNGCGLFGKGGIVTCAMFTCKKNMEGKIVQPENLEYTIVITPHSGEEHAVGNDTGKHGKEIKITPQSSTTEAELTGYGTVTMECSPRTGLDFNEMVLLQMEDKAWLVHRQWFLDLPLPWLPGADTQGSNWIQKETLVTFKNPHAKKQDVVVLGSQEGAMHTALTGATEIQMSSGNLLFTGHLKCRLRMDKLQLKGMSYSMCTGKFKIVKEIAETQHGTIVIRVQYEGDGSPCKIPFEITDLEKRHVLGRLITVNPIVTEKDSPVNIEAEPPFGDSYIIVGVEPGQLKLNWFKKGSSIGQMFETTMRGAKRMAILGDTAWDFGSLGGVFTSIGKALHQVFGAIYGAAFSGVSWTMKILIGVIITWIGMNSRSTSLSVSLVLVGVVTLYLGAMVQADSGCIVSWKNKELKCGSGIFITDNVHTWTEQYKFQPESPSKLASAIQKAHEEGICGIRSVTRLENLMWKQITPELNHILSENEVKLTIMTGDIKGIMQAGKRSLRPQPTELKYSWKTWGKAKMLSTESHNQTFLIDGPETAECPNTNRAWNSLEVEDYGFGVFTTNIWLKLREKQDVFCDSKLMSAAIKDNRAVHADMGYWIESALNDTWKMEKASFIEVKSCHWPKSHTLWSNGVLESEMIIPKNFAGPVSQHNYRPGYHTQTAGPWHLGKLEMDFDFCEGTTVVVTEDCGNRGPSLRTTTASGKLITEWCCRSCTLPPLRYRGEDGCWYGMEIRPLKEKEENLVNSLVTAGHGQIDNFSLGVLGMALFLEEMLRTRVGTKHAILLVALSFVTLITGNMSFRDLGRVMVMVGATMTDDIGMGVTYLALLAAFKVRPTFAAGLLLRKLTSKELMMATIGIALLSQSTLPETILELTDALALGMMVLKIVRNMEKYQLAVTIMAISCVPNAVILQNAWKVSCTILAAVSVSPLLLTSSQQKADWIPLALTIKGLNPTAIFLTTLSRTSKKRSWPLNEAIMAVGMVSILASSLLKNDIPMTGPLVAGGLLTVCYVLTGRSADLELERAADVKWEDQAEISGSSPILSITISEDGSMSIKNEEEEQTLTILIRTGLLVISGVFPVSIPITAAAWYLWEVKKQRAGVLWDVPSPPPVGKAELEDGAYRIKQRGILGYSQIGAGVYKEGTFHTMWHVTRGAVLMHRGKRIEPSWADVKKDLISYGGGWKLEGEWKEGEEVQVLALEPGKNPRAVQTKPGIFKTNTGTIGAVSLDFSPGTSGSPIVDRKGKVVGLYGNGVVTRSGAYVSAIAQTEKSIEDNPEIEDDIFRKKRLTIMDLHPGAGKTKRYLPAIVREAIKRGLRTLILAPTRVVAAEMEEALRGLPIRYQTPAIRAEHTGREIVDLMCHATFTMRLLSPVRVPNYNLIIMDEAHFTDPASIAARGYISTRVEMGEAAGIFMTATPPGSRDPFPQSNAPIMDEEREIPERSWNSGHEWVTDFKGKTVWFVPSIKAGNDIAACLRKNGKKVIQLSRKTFDSEYVKTRANDWDFVVTTDISEMGANFKAERVIDPRRCMKPVILTDGEERVILAGPMPVTHSSAAQRRGRIGRNPKNENDQYIYMGEPLENDEDCAHWKEAKMLLDNINTPEGIIPSMFEPEREKVDAIDGEYRLRGEARKTFVDLMRRGDLPVWLAYKVAAEGINYADRKWCFDGIKNNQILEENVEVEIWTKEGERKKLKPRWLDARIYSDPLALKEFKEFAAGRKSLTLNLITEMGRLPTFMTQKARNALDNLAVLHTAEVGGRAYNHALSELPETLETLLLLTLLATVTGGIFLFLMSGKGIGKMTLGMCCIITASILLWYAQIQPHWIAASIILEFFLIVLLIPEPEKQRTPQDNQLTYVVIAILTVVAATMANEMGFLEKTKKDLGFGSITTQESESNILDIDLRPASAWTLYAVATTFVTPMLRHSIENSSVNVSLTAIANQATVLMGLGKGWPLSKMDIGVPLLAIGCYSQVNPITLTAALLLLVAHYAIIGPGLQAKATREAQKRAAAGIMKNPTVDGITVIDLEPIPYDPKFEKQLGQVMLLILCVTQVLMMRTTWALCEALTLATGPISTLWEGNPGRFWNTTIAVSMANIFRGSYLAGAGLLFSIMKNTTNTRRGTGNVGETLGEKWKSRLNALGKSEFQIYKKSGIQEVDRTLAKEGIKRGETDHHAVSRGSAKLRWFVERNMVTPEGKVVDLGCGRGGWSYYCGGLKNVREVKGLTKGGPGHEEPIPMSTYGWNLVRLQSGVDVFFTPPEKCDTLLCDIGESSPNPTIEAGRTLRVLNLVENWLNNNTQFCIKVLNPYMPSVIEKMETLQRKYGGALVRNPLSRNSTHEMYWVSNATGNIVSSVNMISRMLINRFTMKHKKATYEPDVDLGSGTRNIGIESEIPNLDIIGKRIEKIKQEHETSWHYDQDHPYKTWAYHGSYETKQTGSASSMVNGVVRLLTKPWDVVPMVTQMAMTDTTPFGQQRVFKEKVDTRTQEPKEGTKKLMKITAEWLWKELGKEKTPRMCTREEFTRKVRSNAALGAIFTDENKWKSAREAVEDGRFWELVDRERNLHLEGKCETCVYNMMGKREKKLGEFGKAKGSRAIWYMWLGARFLEFEALGFLNEDHWFSRGNSLSGVEGEGLHRLGYILREVGKKEGGAMYADDTAGWDTRITLEDLKNEEMVTNHMKGEHKKLAEAIFKLTYQNKVVRVQRPTPRGTVMDIISRKDQRGSGQVGTYGLNTFTNMEAQLIRQMEGEGIFKSIQHLTATEEIAVQNWLARVGRERLSRMAISGDDCVVKPIDDRFASALTALNDMGKVRKDIQQWEPSRGWNDWTQVPFCSHHFHELVMKDGRVLVVPCRNQDELIGRARISQGAGWSLKETACLGKSYAQMWTLMYFHRRDLRLAANAICSAVPSHWVPTSRTTWSIHAKHEWMTTEDMLAVWNRVWIQENPWMEDKTPVESWEEVPYLGKREDQWCGSLIGLTSRATWAKNIQTAINQVRSLIGNEEYTDYMPSMKRFRREEEEAGVLW

>DENV2_ARO84705_Malaysia

MNNQRKKARNTPFNMLKRERNRVSTVQQLTKRFSLGMLQGRGPLKLFMALVAFLRFLTIPPTAGILKRWGTIKKSKAINVLRGFRKEIGRMLNILNRRRRTAGIIIMMIPTVMAFHLTTRNGEPHMIVSRQEKGKSLLFKTENGVNMCTLMAMDLGELCEDTITYNCPLLRQNEPEDIDCWCNSTSTWVTYGTCTATGEHRREKRSVALVPHVGMGLETRTETWMSSEGAWKHAQRIETWVLRHPGFTIMAAILAYTIGTTYFQRVLIFILLTAVAPSMTMRCIGISNRDFVEGVSGGSWVDIVLEHGSCVTTMAKNKPTLDFELIKTEAKHPATLRKYCIEAKLTNTTTASRCPTQGEPSLNEEQDKRFVCKHSMVDRGWGNGCGLFGKGGIVTCAMFTCKKNMEGKIVQPENLEYTIVITPHSGEENAVGNDTGKHGKEIKVTPQSSITEAELTGYGTVTMECSPRTGLDFNEMVLLQMENKAWLVHRQWFLDLPLPWLPGADTQGSNWIQKETLVTFKNPHAKKQDVVVLGSQEGAMHTALTGATEIQMSSGNLLFTGHLKCRLRMDKLQLKGMSYSMCTGKFKVVKEIAETQHGTIVIRVQYEGDGSPCKIPFEIMDLEKRHVLGRLITVNPIVTEKDSPVNIEAEPPFGDSYIIIGVEPGQLKLSWFKKGSSIGQMFETTMRGAKRMAILGDTAWDFGSLGGVFTSIGKALHQVFGAIYGAAFSGVSWTMKILIGVVITWIGMNSRSTSLSVSLVLVGVVTLYLGVMVQADSGCVVSWKNKELKCGSGIFITDNVHTWTEQYKFQPESPSKLASAIQKAHEEGICGIRSVTRLENLMWKQITPELNHILTENEVKLTIMTGDIKGIMQAGKRSLRPQPTELKYSWKAWGKAKMLSTELHNHTFLIDGPETAECPNTNRAWNSLEVEDYGFGVFTTNIWLKLKERQDVSCDSKLMSAAIKDNRAVHADMGYWIESALNDTWKIEKASFIEVKSCHWPKSHTLWSNGVLESEMIIPKNFAGPVSQHNYRPGYHTQTAGPWHLGRLEMDFDFCEGTTVVVTENCGNRGPSLRTTTASGKLITEWCCRSCTLPPLRYRGEDGCWYGMEIRPLKEKEENLVNSLVTAGHGQIDNFSLGVLGMALFLEEMLRTRVGTKHAILLVAVSFVTLITGNMSFRDLGRVMVMVGATMTDDIGMGVTYLALLAAFKVRPTFAAGLLLRKLTSKELMMTTIGIVLLSQSTIPETILELTDALALGMMVLKIVRNMEKYQLAVTIMAILCVPNAVILQNAWKVSCTALAVVSVSPLLLTSSQQKTDWIPLALTIKGLNPTAIFLTTLSRTSKKRSWPLNEAIMAVGMVSILASSFLKNDIPMTGPLVAGGLLTVCYVLTGRSADLELERAADIRWEEQAEISGSSPILSITISEDGSMSIKNEEEEQTLTILIRTGLLVISGLFPVSIPITAAAWYLWEVKKQRAGVLWDVPSPPPVGRAELEDGAYRIKQKGILGYSQIGAGVYKEGTFHTMWHVTRGAVLMHKGKRIEPSWADVKKDLISYGGGWKLEGEWKEGEEVQVLALEPGKNPRAVQTKPGLFKTNTGTIGAVSLDFSPGTSGSPIVDKKGKVVGLYGNGVVTRSGTYVSAIAQTEKSIEDNPEIEDDIFRKKRLTIMDLHPGAGKTKRYLPAIVREAIKRGLRTLILAPTRVVAAEMEEALRGLPIRYQTPAIRAEHTGREIVDLMCHATFTMRLLSPIRVPNYNLIIMDEAHFTDPASIAARGYISTRVEMGEAAGIFMTATPPGSRDPFPQSNAPIMDEEREIPERSWNSGHEWVTDFKGKTVWFVPSIKAGNDIAACLRKNGKKVIQLSRKTFDSEYIKTRTNDWDFVVTTDISEMGANFKAERVIDPRRCMKPVILTDGEERVILAGPMPVTHSSAAQRRGRVGRNPKNENDQYIYMGEPLENDEDCAHWKEAKMLLDNINTPEGIIPSMFEPEREKVDAIDGEYRLRGEARKTFVDLMRRGDLPVWLAYRVAAEGINYADRRWCFDGVKNNQILEENVEVEIWTKEGERKKLKPRWLDARIYSDPLALKEFKEFAAGRKSLTLNLITEMGRLPTFMTQKARNALDNLAVLHTAEAGGRAYNHALSELPETLETLLLLTLLATVTGGIFLFLMSGKGIGKMTLGMCCIVTASILLWYAQIQPHWIAASIILEFFLIVLLIPEPEKQRTPQDNQLTYVVIAILTVVAATMANEMGFLEKTKKDFGLGSITTQQPESNILDIDLRPASAWTLYAVATTFITPMLRHSIENSSVNVSLTAIANQATVLMGLGKGWPLSKMDIGVPLLAIGCYSQVNPITLTAALLLLVAHYAIIGPGLQAKATREAQKRAAAGIMKNPTVDGITVIDLDPIPYDPKFEKQLGQVMLLVLCVTQVLMMRTTWALCEALTLATGPISTLWEGNPGRFWNTTIAVSMANIFRGSYLAGAGLLFSIMKNTANTRRGTGNTGETLGEKWKNRLNALGKSEFQIYKKSGIQEVDRTLAKEGIKRGETDHHAVSRGSAKLRWFVERNLVTPEGKVVDLGCGRGGWSYYCGGLKNVKEVKGLTKGGPGHEEPIPMSTYGWNLVRLQSGVDVFFTPPEKCDTLLCDIGESSPNPTVEAGRTLRVLNLVENWLNNNTQFCIKVLNPYMPSVIEKMEALQRKYGGALVRNPLSRNSTHEMYWVSNASGNIVSSVNMISRMLINRFTMRHKKATYEPDVDLGSGTRNIGIESEVPNLDIIGKRIEKIKQEHETSWHYDQDHPYKTWAYHGSYETKQTGSASSMVNGVVRLLTKPWDVIPMVTQMAMTDTTPFGQQRVFKEKVDTRTQEPKEGTKKLMKITAEWLWKELGKKKTPRMCTREEFTRKVRSNAALGAIFTDENKWKSAREAVEDSGFWELVDKERNLHLEGKCETCVYNMMGKREKKLGEFGKAKGSRAIWYMWLGARFLEFEALGFLNEDHWFSRENSLSGVEGEGLHKLGYILRDVSKKEGGAMYADDTAGWDTRITLEDLKNEEMVTNHMEGEHKKLAEAIFRLTYQNKVVRVQRPTPRGTVMDIISRRDQRGSGQVVTYGLNTFTNMEAQLIRQMEGEGVFKSIQHLTATEEIAVKNWLVKVGRERLSRMAISGDDCVVKPLDDRFANALTALNDMGKVRKDIQQWEPSRGWNDWTQVPFCSHHFHELIMKDGRVLVVPCRNQDELIGRARISQGAGWSLRETACLGKSYAQMWSLMYFHRRDLRLAANAICSAVPSHWVPTSRTTWSIHATHEWMTTEDMLTVWNRVWIQENPWMEDKTPVESWEEIPYLGKREDQWCGSLIGLTSRATWAKNIQTAINQVRSLIGNEEYTDYMPSMKRFRREEEEAGVLW

>DENV2_ARO84704_Malaysia

MNNQRKKARNTPFNMLKRERNRVSTVQQLTKRFSLGMLQGRGPLKLFMALVAFLRFLTIPPTAGILKRWGTIKKSKAINVLRGFRKEIGRMLNILNRRRRTAGIIIMMIPTVMAFHLTTRNGEPHMIVSRQEKGKSLLFKTENGVNMCTLMAMDLGELCEDTITYNCPLLRQNEPEDIDCWCNSTSTWVTYGTCTATGEHRREKRSVALVPHVGMGLETRTETWMSSEGAWKHAQRIETWVLRHPGFTIMAAILAYTIGTTYFQRVLIFILLTAVAPSMTMRCIGISNRDFVEGVSGGSWVDIVLEHGSCVTTMAKNKPTLDFELIKTEAKHPATLRKYCIEAKLTNTTTASRCPTQGEPSLNEEQDKRFVCKHSMVDRGWGNGCGLFGKGGIVTCAMFTCKKNMEGKIVQPENLEYTIVITPHSGEENAVGNDTGKHGKEIKVTPQSSITEAELTGYGTVTMECSPRTGLDFNEMVLLQMENKAWLVHRQWFLDLPLPWLPGADTQGSNWIQKETLVTFKNPHAKKQDVVVLGSQEGAMHTALTGATEIQMSSGNLLFTGHLKCRLRMDKLQLKGMSYSMCTGKFKVVKEIAETQHGTIVIRVQYEGDGSPCKIPFEIMDLEKRHVLGRLITVNPIVTEKDSPVNIEAEPPFGDSYIIIGVEPGQLKLSWFKKGSSIGQMFETTMRGAKRMAILGDTAWDFGSLGGVFTSIGKALHQVFGAIYGAAFSGVSWTMKILIGVVITWIGMNSRSTSLSVSLVLVGVVTLYLGVMVQADSGCVVSWKNKELKCGSGIFITDNVHTWTEQYKFQPESPSKLASAIQKAHEEGICGIRSVTRLENLMWKQITPELNHILTENEVKLTIMTGDIKGIMQAGKRSLRPQPTELKYSWKAWGKAKMLSTELHNHTFLIDGPETAECPNTNRAWNSLEVEDYGFGVFTTNIWLKLKERQDVSCDSKLMSAAIKDNRAVHADMGYWIESALNDTWKIEKASFIEVKSCHWPKSHTLWSNGVLESEMIIPKNFAGPVSQHNYRPGYHTQTAGPWHLGRLEMDFDFCEGTTVVVTEDCGNRGPSLRTTTASGKLITEWCCRSCTLPPLRYRGEDGCWYGMEIRPLKEKEENLVNSLVTAGHGQIDNFSLGVLGMALFLEEMLRTRVGTKHAILLVAVSFVTLITGNMSFRDLGRVMVMVGATMTDDIGMGVTYLALLAAFKVRPTFAAGLLLRKLTSKELMMTTIGIVLLSQSTIPETILELTDALALGMMVLKIVRNMEKYQLAVTIMAILCVPNAVILQNAWKVSCTALAVVSVSPLLLTSSQQKTDWIPLALTIKGLNPTAIFLTTLSRTSKKRSWPLNEAIMAVGMVSILASSFLKNDIPMTGPLVAGGLLTVCYVLTGRSADLELERAADIRWEEQAEISGSSPILSITISEDGSMSIKNEEEEQTLTILIRTGLLVISGLFPVSIPITAAAWYLWEVKKQRAGVLWDVPSPPPVGRAELEDGAYRIKQKGILGYSQIGAGVYKEGTFHTMWHVTRGAVLMHKGKRIEPSWADVKKDLISYGGGWKLEGEWKEGEEVQVLALEPGKNPRAVQTKPGLFKTNTGTIGAVSLDFSPGTSGSPIVDKKGKVVGLYGNGVVTRSGTYVSAIAQTEKSIEDNPEIEDDIFRKKRLTIMDLHPGAGKTKRYLPAIVREAIKRGLRTLILAPTRVVAAEMEEALRGLPIRYQTPAIRAEHTGREIVDLMCHATFTMRLLSPIRVPNYNLIIMDEAHFTDPASIAARGYISTRVEMGEAAGIFMTATPPGSRDPFPQSNAPIMDEEREIPERSWNSGHEWVTDFKGKTVWFVPSIKAGNDIAACLRKNGKKVIQLSRKTFDSEYIKTRTNDWDFVVTTDISEMGANFKAERVIDPRRCMKPVILTDGEERVILAGPMPVTHSSAAQRRGRVGRNPKNENDQYIYMGEPLENDEDCAHWKEAKMLLDNINTPEGIIPSMFEPEREKVDAIDGEYRLRGEARKTFVDLMRRGDLPVWLAYRVAAEGINYADRRWCFDGVKNNQILEENVEVEIWTKEGERKKLKPRWLDARIYSDPLALKEFKEFAAGRKSLTLNLITEMGRLPTFMTQKARNALDNLAVLHTAEAGGRAYNHALSELPETLETLLLLTLLATVTGGIFLFLMSGKGIGKMTLGMCCIVTASILLWYAQIQPHWIAASIILEFFLIVLLIPEPEKQRTPQDNQLTYVVIAILTVVAATMANEMGFLEKTKKDLGLGSITTQQPESNILDIDLRPASAWTLYAVATTFITPMLRHSIENSSVNVSLTAIANQATVLMGLGKGWPLSKMDIGVPLLAIGCYSQVNPITLTAALLLLVAHYAIIGPGLQAKATREAQKRAAAGIMKNPTVDGITVIDLDPIPYDPKFEKQLGQVMLLVLCVTQVLMMRTTWALCEALTLATGPISTLWEGNPGRFWNTTIAVSMANIFRGSYLAGAGLLFSIMKNTANTRRGTGNTGETLGEKWKNRLNALGKSEFQIYKKSGIQEVDRTLAKEGIKRGETDHHAVSRGSAKLRWFVERNLVTPEGKVVDLGCGRGGWSYYCGGLKNVKEVKGLTKGGPGHEEPIPMSTYGWNLVRLQSGVDVFFTPPEKCDTLLCDIGESSPNPTVEAGRTLRVLNLVENWLNNNTQFCIKVLNPYMPSVIEKMEALQRKYGGALVRNPLSRNSTHEMYWVSNASGNIVSSVNMISRMLINRFTMRHKKATYEPDVDLGSGTRNIGIESEVPNLDIIGKRIEKIKQEHETSWHYDQDHPYKTWAYHGSYETKQTGSASSMVNGVVRLLTKPWDVIPMVTQMAMTDTTPFGQQRVFKEKVDTRTQEPKEGTKKLMKITAEWLWKELGKKKTPRMCTREEFTRKVRSNAALGAIFTDENKWKSAREAVEDSGFWELVDKERNLHLEGKCETCVYNMMGKREKKLGEFGKAKGSRAIWYMWLGARFLEFEALGFLNEDHWFSRENSLSGVEGEGLHKLGYILRDVSKKEGGAMYADDTAGWDTRITLEDLKNEEMVTNHMEGEHKKLAEAIFRLTYQNKVVRVQRPTSRGTVMDIISRRDQRGSGQVVTYSLNTFTNMEAQLIRQMEGEGVFKSIQHLTATEEIAVKNWLVRVGRERLSRMAISGDDCVVKPLDDRFANALTALNDMGKVRKDIQQWEPSRGWNDWTQVPFCSHHFHELIMKDGRVLVVPCRNQDELIGRARISQGAGWSLRETACLGKSYAQMWSLMYFHRRDLRLAANAICSAVPSHWVPTSRTTWSIHATHEWMTTEDMLTVWNRVWIQENPWMEDKTPVESWEEIPYLGKREDQWCGSLIGLTSRATWAKNIQTAINQVRSLIGNEEYTDYMPSMKRFRREEEEAGVLW

>DENV2_APW84878_Haiti

MNNQRKKARSTPFNMLKRERNRVSTVQQLTKRFSLGMLQGRGPLKLFMALVAFLRFLTIPPTAGILKRWGTIKKSKAINVLRGFRKEIGRMLNILNRRRRTAGVIVMLIPTAMAFHLTTRNGEPHMIVGRQEKGKSLLFKTEDGVNMCTLMAIDLGELCEDTITYKCPLLRQNEPEDIDCWCNSTSTWVTYGTCTTTGEHRREKRSVALVPHVGMGLETRTETWMSSEGAWKHVQRIETWILRHPGFTIMAAILAYTIGTTHFQRALILILLTAVAPSMTMRCIGISNRDFVEGVSGGSWVDIVLEHGSCVTTMAKNKPTLDFELIKTEAKQPATLRKYCIEAKLTNTTTESRCPTQGEPSLNEEQDKRFICKHSMVDRGWGNGCGLFGKGGIVTCAMFTCKKNMEGKIVQPENLEYTIVITPHSGEEHAVGNDTGKHGKEIKITPQSSTTEAELTGYGTVTMECSPRTGLDFNEMVLLQMEDKAWLVHRQWFLDLPLPWLPGADTQGSNWIQKETLVTFKNPHAKKQDVVVLGSQEGAMHTALTGATEIQMSSGNLLFTGHLKCRLRMDKLQLKGMSYSMCTGKFKIVKEIAETQHGTIVIRVQYEGDGSPCKIPFEITDLEKRHVLGRLITVNPIVTEKDSPVNIEAEPPFGDSYIIVGVEPGQLKLNWFKKGSSIGQMFETTMRGAKRMAILGDTAWDFGSLGGVFTSIGKALHQVFGAIYGAAFSGVSWTMKILIGVIITWIGMNSRSTSLSVSLVLVGVVTLYLGAMVQADSGCIVSWKNKELKCGSGIFITDNVHTWTEQYKFQPESPSKLASAIQKAHEEGICGIRSVTRLENLMWKQITPELNHILSENEVKLTIMTGDIKGIMQAGKRSLRPQPTELKYSWKTWGKAKMLSTESHNQTFLIDGPETAECPNTNRAWNSLEVEDYGFGVFTTNIWLKLREKQDVFCDSKLMSAAIKDNRAVHADMGYWIESALNDTWKMEKASFIEVKSCHWPKSHTLWSNGVLESEMIIPKNFAGPVSQHNYRPGYHTQTAGPWHLGKLEMDFDFCEGTTVVVTEDCEKRGPSLRTTTASGKLITEWCCRSCTLPPLRYRGEDGCWYGMEIRPLKEKEENLVNSLVTAGHGQIDNFSLGVLGMALFLEEMLRTRVGTKHAILLVALSFVTLITGNMSFRDLGRVMVMVGATMTDDIGMGVTYLALLAAFKVRPTFAAGLLLRKLTSKELMMATIGIALLSQSTLPETILELTDALALGMMALKIVRNMEKYQLAVTIMAISCVPNAVILQNAWKVSCTILAAVSVSPLLLTSSQQKADWIPLALTIKGLNPTAIFLTTLSRTSKKRSWPLNEAIMAVGMVSILASSLLKNDIPMTGPLVAGGLLTVCYVLTGRSADLELERAADVKWEDQAEISGSSPILSITISEDGSMSIKNEEEEQTLTILIRTGLLVISGVFPVSIPITAAAWYLWEVKKQRAGVLWDVPSPPPVGKAELEDGAYRIKQRGILGYSQIGAGVYKEGTFHTMWHVTRGAVLMHRGKRIEPSWADVKKDLISYGGGWKLEGEWKEGEEVQVLALEPGKNPRAVQTKPGIFKTNTGTIGAVSLDFSPGTSGSPIVDRKGKVVGLYGNGVVTRSGAYVSAIAQTEKSIEDNPEIEDDIFRKKRLTIMDLHPGAGKTKRYLPAIVREAIKRGLRTLILAPTRVVAAEMEEALRGLPIRYQTPAIRAEHTGREIVDLMCHATFTMRLLSPVRVPNYNLIIMDEAHFTDPASIAARGYISTRVEMGEAAGIFMTATPPGSRDPFPQSNAPIMDEEREIPERSWNSGHEWVTDFKGKTVWFVPSIKAGNDIAACLRKNGKKVIQLSRKTFDSEYVKTRANDWDFVVTTDISEMGANFKAERVIDPRRCMKPVILTDGEERVILAGPMPVTHSSAAQRRGRIGRNPKNENDQYIYMGEPLENDEDCAHWKEAKMLLDNINTPEGIIPSMFEPEREKVDAIDGEYRLRGEARKTFVDLMRRGDLPVWLAYKVAAEGINYADRKWCFDGIKNNQILEENVEVEIWTKEGERKKLKPRWLDARIYSDPLALKEFKEFAAGRKSLTLNLITEMGRLPTFMTQKARNALDNLAVLHTAEVGGKAYTHALSELPETLETLLLLTLLAAVTGGIFLFLMSGKGIGKMTLGMCCIITASILLWYAQIQPHWIAASIILEFFLIVLLIPEPEKQRTPQDNQLTYVVIAILTVVAATMANEMGFLEKTKKDLGFGSITTQESESNILDIDLRPASAWTLYAVATTFVTPMLRHSIENSSVNVSLTAIANQATVLMGLGKGWPLSKMDIGVPLLAIGCYSQVNPITLTAALLLLVAHYAIIGPGLQAKATREAQKRAAAGIMKNPTVDGITVIDLEPIPHDPKFEKQLGQVMLLILCVTQVLMMRTTWALCEALTLATGPISTLWEGNPGRFWNTTIAVSMANIFRGSYLAGAGLLFSIMKNTTNTRRGTGNVGETLGEKWKSRLNALGKSEFQIYKKSGIQEVDRTLAKEGIKRGETDHHAVSRGSAKLRWFVERNMVTPEGKVVDLGCGRGGWSYYCGGLKNVREVKGLTKGGPGHEEPIPMSTYGWNLVRLQSGVDVFFTPPEKCDTLLCDIGESSPNPTIEAGRTLRVLNLVENWLNNNTQFCIKVLNPYMPSVIEKMETLQRKYGGALVRNPLSRNSTHEMYWVSNATGNIVSSVNMISRMLINRFTMKHKKATYEPDVDLGSGTRNIGIESETPNLDIIGKRIEKIKQEHETSWHYDQDHPYKTWAYHGSYETKQTGSASSMVNGVVRLLTKPWDVVPMVTQMAMTDTTPFGQQRVFKEKVDTRTQEPKEGTKKLMKITAEWLWKELGKEKTPRMCTREEFTRKVRSNAALGAVFTDENKWKSAREAVEDGRFWELVDRERNLHLEGKCETCVYNMMGKREKKLGEFGKAKGSRAIWYMWLGARFLEFEALGFLNEDHWFSRGNSLSGVEGEGLHRLGYILREVGKKEGGAMYADDTAGWDTRITLEDLKNEEMVTNHMKGEHKKLAEAIFKLTYQNKVVRVQRPTPRGTVMDIISRKDQRGSGQVGTYGLNTFTNMEAQLIRQMEGEGIFKSIQHLTATEETAVQNWLARVGRERLSRMAISGDDCVVKPIDDRFASALTALNDMGKVRKDIQQWEPSRGWNDWTQVPFCSHHFHELVMKDGRVLVVPCRNQDELIGRARISQGAGWSLKETACLGKSYAQMWTLMYFHRRDLRLAANAICSAVPSHWVPTSRTTWSIHAKHEWMTTEDMLAVWNRVWIQENPWMEDKTPVESWEEVPYLGKREDQWCGSLIGLTSRATWAKNIQTAINQVRSLIGNEGYTDYMPSMKRFRREEEEAGVLW

>DENV2_AOE23002_Haiti

MNNQRKKARSTPFNMLKRERNRVSTVQQLTKRFSLGMLQGRGPLKLFMALVAFLRFLTIPPTAGILKRWGTIKKSKAINVLRGFRKEIGRMLNILNRRRRTAGVIVMLIPTAMAFHLTTRNGEPHMIVGRQEKGKSLLFKTEDGVNMCTLMAIDLGELCEDTITYKCPLLRQNEPEDIDCWCNSTSTWVTYGTCTTTGEHRREKRSVALVPHVGMGLETRTETWMSSEGAWKHVQRIETWILRHPGFTIMAAILAYTIGTTHFQRALILILLTAVAPSMTMRCIGISNRDFVEGVSGGSWVDIVLEHGSCVTTMAKNKPTLDFELIKTEAKQPATLRKYCIEAKLTNTTTESRCPTQGEPSLNEEQDKRFICKHSMVDRGWGNGCGLFGKGGIVTCAMFTCKKNMEGKIVQPENLEYTIVITPHSGEEHAVGNDTGKHGKEIKITPQSSTTEAELTGYGTVTMECSPRTGLDFNEMVLLQMEDKAWLVHRQWFLDLPLPWLPGADTQGSNWIQKETLVTFKNPHAKKQDVVVLGSQEGAMHTALTGATEIQMSSGNLLFTGHLKCRLRMDKLQLKGMSYSMCTGKFKIVKEIAETQHGTIVIRVQYEGDGSPCKIPFEITDLEKRHVLGRLITVNPIVTEKDSPVNIEAEPPFGDSYIIVGVEPGQLKLNWFKKGSSIGQMFETTMRGAKRMAILGDTAWDFGSLGGVFTSIGKALHQVFGAIYGAAFSGVSWTMKILIGVIITWIGMNSRSTSLSVSLVLVGVVTLYLGAMVQADSGCIVSWKNKELKCGSGIFITDNVHTWTEQYKFQPESPSKLASAIQKAHEEGICGIRSVTRLENLMWKQITPELNHILSENEVKLTIMTGDIKGIMQAGKRSLRPQPTELKYSWKTWGKAKMLSTESHNQTFLIDGPETAECPNTNRAWNSLEVEDYGFGVFTTNIWLKLREKQDVFCDSKLMSAAIKDNRAVHADMGYWIESALNDTWKMEKASFIEVKSCHWPKSHTLWSNGVLESEMIIPKNFAGPVSQHNYRPGYHTQTAGPWHLGKLEMDFDFCEGTTVVVTEDCEKRGPSLRTTTASGKLITEWCCRSCTLPPLRYRGEDGCWYGMEIRPLKEKEENLVNSLVTAGHGQIDNFSLGVLGMALFLEEMLRTRVGTKHAILLVALSFVTLITGNMSFRDLGRVMVMVGATMTDDIGMGVTYLALLAAFKVRPTFAAGLLLRKLTSKELMMATIGIALLSQSTLPETILELTDALALGMMALKIVRNMEKYQLAVTIMAISCVPNAVILQNAWKVSCTILAAVSVSPLLLTSSQQKADWIPLALTIKGLNPTAIFLTTLSRTSKKRSWPLNEAIMAVGMVSILASSLLKNDIPMTGPLVAGGLLTVCYVLTGRSADLELERAADVKWEDQAEISGSSPILSITISEDGSMSIKNEEEEQTLTILIRTGLLVISGVFPVSIPITAAAWYLWEVKKQRAGVLWDVPSPPPVGKAELEDGAYRIKQRGILGYSQIGAGVYKEGTFHTMWHVTRGAVLMHRGKRIEPSWADVKKDLISYGGGWKLEGEWKEGEEVQVLALEPGKNPRAVQTKPGIFKTNTGTIGAVSLDFSPGTSGSPIVDRKGKVVGLYGNGVVTRSGAYVSAIAQTEKSIEDNPEIEDDIFRKKRLTIMDLHPGAGKTKRYLPAIVREAIKRGLRTLILAPTRVVAAEMEEALRGLPIRYQTPAIRAEHTGREIVDLMCHATFTMRLLSPVRVPNYNLIIMDEAHFTDPASIAARGYISTRVEMGEAAGIFMTATPPGSRDPFPQSNAPIMDEEREIPERSWNSGHEWVTDFKGKTVWFVPSIKAGNDIAACLRKNGKKVIQLSRKTFDSEYVKTRANDWDFVVTTDISEMGANFKAERVIDPRRCMKPVILTDGEERVILAGPMPVTHSSAAQRRGRIGRNPKNENDQYIYMGEPLENDEDCAHWKEAKMLLDNINTPEGIIPSMFEPEREKVDAIDGEYRLRGEARKTFVDLMRRGDLPVWLAYKVAAEGINYADRKWCFDGIKNNQILEENVEVEIWTKEGERKKLKPRWLDARIYSDPLALKEFKEFAAGRKSLTLNLITEMGRLPTFMTQKARNALDNLAVLHTAEVGGKAYTHALSELPETLETLLLLTLLAAVTGGIFLFLMSGKGIGKMTLGMCCIITASILLWYAQIQPHWIAASIILEFFLIVLLIPEPEKQRTPQDNQLTYVVIAILTVVAATMANEMGFLEKTKKDLGFGSITTQESESNILDIDLRPASAWTLYAVATTFVTPMLRHSIENSSVNVSLTAIANQATVLMGLGKGWPLSKMDIGVPLLAIGCYSQVNPITLTAALLLLVAHYAIIGPGLQAKATREAQKRAAAGIMKNPTVDGITVIDLEPIPHDPKFEKQLGQVMLLILCVTQVLMMRTTWALCEALTLATGPISTLWEGNPGRFWNTTIAVSMANIFRGSYLAGAGLLFSIMKNTTNTRRGTGNVGETLGEKWKSRLNALGKSEFQIYKKSGIQEVDRTLAKEGIKRGETDHHAVSRGSAKLRWFVERNMVTPEGKVVDLGCGRGGWSYYCGGLKNVREVKGLTKGGPGHEEPIPMSTYGWNLVRLQSGVDVFFTPPEKCDTLLCDIGESSPNPTIEAGRTLRVLNLVENWLNNNTQFCIKVLNPYMPSVIEKMETLQRKYGGALVRNPLSRNSTHEMYWVSNATGNIVSSVNMISRMLINRFTMKHKKATYEPDVDLGSGTRNIGIESETPNLDIIGKRIEKIKQEHETSWHYDQDHPYKTWAYHGSYETKQTGSASSMVNGVVRLLTKPWDVVPMVTQMAMTDTTPFGQQRVFKEKVDTRTQEPKEGTKKLMKITAEWLWKELGKEKTPRMCTREEFTRKVRSNAALGAVFTDENKWKSAREAVEDGRFWELVDRERNLHLEGKCETCVYNMMGKREKKLGEFGKAKGSRAIWYMWLGARFLEFEALGFLNEDHWFSRGNSLSGVEGEGLHRLGYILREVGKKEGGAMYADDTAGWDTRITLEDLKNEEMVTNHMKGEHKKLAEAIFKLTYQNKVVRVQRPTPRGTVMDIISRKDQRGSGQVGTYGLNTFTNMEAQLIRQMEGEGIFKSIQHLTATEETAVQNWLARVGRERLSRMAISGDDCVVKPIDDRFASALTALNDMGKVRKDIQQWEPSRGWNDWTQVPFCSHHFHELVMKDGRVLVVPCRNQDELIGRARISQGAGWSLKETACLGKSYAQMWTLMYFHRRDLRLAANAICSAVPSHWVPTSRTTWSIHAKHEWMTTEDMLAVWNRVWIQENPWMEDKTPVESWEEVPYLGKREDQWCGSLIGLTSRATWAKNIQTAINQVRSLIGNEGYTDYMPSMKRFRREEEEAGVLW

>DENV2_AII99332_India

MNNQRKKARNTPFNMLKRERNRVSTVQQLTKRFSLGMLQGRGPLKLFMALVAFLRFLTIPPTAGILKRWGTIKKSKAINVLRGFRKEIGRMLNILNRRRRTAGMIIMLIPTVMAFHLTTRNGEPHMIVSRQEKGKSLLFKTKDGTNMCTLMAMDLGELCEDTITYKCPFLRQNEPEDIDCWCNSTSTWVTYGTCTTTGEHRREKRSVALVPHVGMGLETRTETWMSSEGAWKHAQRIEIWILRHPGFTIMAAILAYTIGTTHFQRVLIFILLTAIAPSMTMRCIGISNRDFVEGVSGGSWVDIVLEHGSCVTTMAKNKPTLDFELIKTEAKQPATLRKYCIEAKLTNTTTDSRCPTQGEPTLNEEQDKRFVCKHSMVDRGWGNGCGLFGKGGIVTCAMFTCKKIMKGKIVQPENLEYTVVITPHSGEEHAVGNDTGKHGKEVKITPQSSITEAELTGYGTVTMECSPRTGLDFNEMVLLQMEDKAWLVHRQWFLDLPLPWLPGADTQGSNWIQKETLVTFKNPHAKKQDVVVLGSQEGAMHTALTGATEIQMSSGNLLFTGHLKCRLRMDKLQLKGMSYSMCTGKFKVVKEIAETQHGTIVIRVQYEGDGSPCKIPFEIMDLEKRHVLGRLITVNPIVTEKDSPVNIEAEPPFGDSYIIIGVEPGQLKLNWFKKGSSIGQMLETTMRGAKRMAILGDTAWDFGSLGGVFTSIGKALHQVFGAIYGAAFSGVSWTMKILTGVIITWIGMNSRSTSLSVSLVLVGIVTLYLGVMVQADSGCVVSWKNKELKCGSGIFVTDNVHTWTEQYKFQPESPSKLASAIQKAHEEGICGIRSVTRLENLMWKQITPELNHILSENEVKLTIMTGDIKGIMQVGKRSLRPQPTELRYSWKTWGKAKMLSTELHNQTFLIDGPETAECPNTNRAWNSLEVEDYGFGVFTTNIWLRLREKQDVFCDSKLMSAAIKDNRAVHADMGYWIESALNDTWKIEKASFIEVKSCHWPKSHTLWSDGVLESEMVIPKNFAGPVSQHNNRPGYHTQTAGPWHLGKLEMDFDFCEGTTVVVTEDCGNRGPSLRTTTASGKLITEWCCRSCTLPPLRYRGEDGCWYGMEIRPLKEKEENLVSSLVTAGHGQIDNFSLGILGMALFLEEMLRTRVGTKHAILLVAVSFVTLITGNMSFRDLGRVMVMVGATMTDDIGMGVTYLALLAAFKVRPTFAAGLLLRKLTSKELMMTTIGIVLLSQSSIPETILELTDALALGMMVLKMVRNMEKYQLAVTIMAILCVPNAVILQNAWKVSCTILALVSVSPLFLTSSQQKADWIPLALTIKGLNPTAIFLTTLSRTSKKRSWPLNEAIMAVGMVSILASSLLKNDIPMTGPLVAGGLLTVCYVLTGRSADLELERAADVKWDDQAEISGSSPILSITISEDGSMSIKNEEEEQTLTILIRTGLLVISGLFPVSIPITAAAWYLWEVKKQRAGVLWDVPSPPPVGKAELEDGAYRIKQKGILGYSQIGAGVYKEGTFHTMWHVTRGAVLMHKGKRIEPSWADVKKDLISYGGGWKLEGEWKEGEEVQVLALEPGKNPRAVQTKPGLFKTNTGTIGAVSLDFSPGTSGSPIVDKKGKVVGLYGNGVVTRSGAYVSAIAQTEKSIEDNPEIEDDIFRKRRLTIMDLHPGAGKTKRYLPAIVREAIKRGLRTLILAPTRVVAAEMEEALRGLPIRYQTPAIRAEHTGREIVDLMCHATFTMRLLSPIRVPNYNLIIMDEAHFTDPASIAARGYISTRVEMGEAAGIFMTATPPGSRDPFPQSNAPIMDEEREIPERSWNSGHEWVTDFKGKTVWFVPSIKTGNDIAACLRKNGKRVIQLSRKTFDSEYVKTRTNDWDFVVTTDISEMGANFKAERVIDPRRCMKPVILTDGEERVILAGPMPVTHSSAAQRRGRIGRNPKNENDQYIYMGEPLENDEDCAHWKEAKMLLDNINTPEGIIPSMFEPEREKVDAIDGEYRLRGEARKTFVDLMRRGDLPVWLAYKVAAEGINYADRRWCFDGTRNNQILEENVEVEIWTKEGERKKLKPRWLDARIYSDPLALKEFKEFAAGRKSLTLNLITEMGRLPTFMTQKARDALDNLAVLHTAEAGGKAYNHALSELPETLETLLLLTLLATVTGGIFLFLMSGRGIGKMTLGMCCIITASILLWCAQIQPHWIAASIILEFFLIVLLIPEPEKQRTPQDNQLTYVIIAILTVVAATMANEMGFLEKTKKDLGLGNIVTQQPESNILDIDLRPASAWTLYAVATTFITPMLRHSIENSSVNMSLTAIANQATVLMGLGKGWPLSKMDIGVPLLAIGCCSQVNPITLTAALFLLVAHYAIIGPGLQAKATREAQKRAAAGIMKNPTVDGITVIDLDPIPYDPKFEKQLGQVMLLVLCVTQVLIMRTTWALCEALTLATGPVSTLWEGNPGRFWNTTIAVSMANIFRGSYLAGAGLLFCIMNNTTRLRRGTGHLRKTLGDKCKSRLNTLGKSEFQIYKKSGIQEVDRTLAKEGIKRGETDHHAVSRGSAKLRWFVERNLVTPEGKVVDLGCGRGGWSYYCGGLKNVREVKGLTKGGPGHEEPIPMSTYGWNLVRLQSGVDVFFIPPEKCDTLLCDIGESSPNPTVEAGRTLRVLNLVENWLNNNTQFCIKVLNPYMPSVIEKMETLQRKYGGALVRNPLSRNSTHEMYWVSNASGNIVSSVNMISRMLINRFTMRHKKATYEPDVDLGSGTRNIGIESETPNLDIIGKRIEKIKQEHETSWHYDQDHPYKTWAYHGSYETKQTGSASSMVNGVVRLLTKPWDVVPMVTQMAMTDTTPFGQQRVFKEKVDTRTQEPKEGTKKLMKITAEWLWKELGKKKTPRMCTREEFTKKVRSNAALGAIFTDENKWKSAREAVEDGRFWELVDKERNLHLEGKCETCVYNMMGKREKKLGEFGKAKGSRAIWYMWLGARFLEFEALGFLNEDHWFSRENSLSGVEGEGLHKLGYILREVSKKEGGAMYADDTAGWDTRITIEDLKNEEMITNHMAGEHKKLAEAIFKLTYQNKVVRVQRPTPRGTVMDIISRRDQRGSGQVGTYGLNTFTNMEAQLIRQMEGEGIFKSIQHLTATEEIAVQDWLARVGRERLSRMAISGDDCVVKPLDDRFARALTALNDMGKVRKDIQQWEPSRGWNDWTQVPFCSHHFHELIMKDGRTLVVPCRNQDELIGRARISQGAGWSLRETACLGKSYAQMWSLMYFHRRDLRLAANAICSAVPSHWVPTSRTTWSIHASHEWMTTEDMLTVWNRVWIQENPWMEDKTPVESWEEIPYLGKREDQWCGSLIGLTSRATWAKNIQTAINQVRSLIGNEEYTDYMPSMKRFRREEEEAGVLW

>DENV2_AHI43753_Mexico

MNNQRKKARSTPFNMLKRERNRVSTVQQLTKRFSLGMLQGRGPLKLFMALVAFLRFLTIPPTAGILKRWGTIKKSKAINVLRGFRKEIGRMLNILNRRRRTAGVIVMLIPTAMAFHLTTRNGEPHMIVSRQEKGKSLLFKTEDGVNMCTLMAIDLGELCEDTITYKCPLLRQNEPEDIDCWCNSTSTWVTYGTCTTTGEHRREKRSVALVPHVGMGLETRTETWMSSEGAWKHVQRIETWILRHPGFTIMAAILAYTIGTTHFQRALIFILLTAVAPSMTMRCIGISNRDFVEGVSGGSWVDIVLEHGSCVTTMAKNKPTLDFELIKTEAKQPATLRKYCIEAKLTNTTTESRCPTQGEPSLNEEQDKRFICKHSMVDRGWGNGCGLFGKGGIVTCAMFTCKKNMEGKVVQPENLEYTIVITPHSGEEHAVGNDTGKHGKEIKITPQSSITEAELTGYGTVTMECSPRTGLDFNEMVLLQMEDKAWLVHRQWFLDLPLPWLPGADTQGSNWIQKETLVTFKNPHAKKQDVVVLGSQEGAMHTALTGATEIQMSSGNLLFTGHLKCRLRMDKLQLKGMSYSMCTGKFKIVKEIAETQHGTIVIRVQYEGDGSPCKIPFEITDLEKRHVLGRLITVNPIVTEKDSPVNIEAEPPFGDSYIIIGVEPGQLKLNWFKKGSSIGQMFETTMRGAKRMAILGDTAWDFGSLGGVFTSIGKALHQVFGAIYGAAFSGVSWTMKILIGVIITWIGMNSRSTSLSVSLVLVGVVTLYLGAMVQADSGCVVSWKNKELKCGSGIFITDNVHTWTEQYKFQPESPSKLASAIQKAHEEGICGIRSVTRLENLMWKQITPELNHILSENEVKLTIMTGDIKGIMQAGKRSLRPQPTELKYSWKTWGKAKMLSTESHNQTFLIDGPETAECPNTNRAWNSLEVEDYGFGVFTTNIWLKLKEKQDVFCDSKLMSAAIKDNRAVHADMGYWIESALNDTWKMEKASFIEVKSCHWPKSHTLWSNGVLESEMIIPKNFAGPVSQHNYRPGYHTQTAGPWHLGKLEMDFDLCEGTTVVVTEDCGNRGPSLRTTTASGKLITEWCCRSCTLPPLRYRGEDGCWYGMEIRPLKEKEENLVNSLVTAGHGQIDNFSLGVLGMALFLEEMLRTRIGTKHAILLVAVSFVTLITGNMSFRDLGRVMVMVGATMTDDIGMGVTYLALLAAFKVRPTFAAGLLLRKLTSKELMMATIGIALLSQSTIPETILELTDALALGMMVLKVVRNMEKYQLAVTIMAISCVPNAVILQNAWKVSCTILAAVSVSPLLLTSSQQKADWIPLALTIKGLNPTAIFLTTLSRTSKKRSWPLNEAIMAVGMVSILASSLLKNDIPMTGPLVAGGLLTVCYVLTGRSADLELERAADVKWEDQAEISGSSPILSITISEDGSMSIKNEEEEQTLTILIRTGLLVISGVFPVSIPITAAAWYLWEVKKQRAGVLWDVPSPPPVEKAELEDGAYRIKQRGILGYSQIGAGVYKEGTFHTMWHVTRGAVLMHRGKRIEPSWADVKKDLISYGGGWKLEGEWKEGEEVQVLALEPGKNPRAVQTKPGIFKTNTGTIGAVSLDFSPGTSGSPIVDRKGKVVGLYGNGVVTRSGAYVSAIAQTEKSIEDNPEIEDDIFRKKRLTIMDLHPGAGKTKRYLPAIVREAIKRGLRTLILAPTRVVAAEMEEALRGLPIRYQTPAIKAEYTGREIVDLMCHATFTMRLLSPVRVPNYNLIIMDEAHFTDPASIAARGYISTRVEMGEAAGIFMTATPPGSRDPFPQSNAPIMDEEREIPERSWNSGHEWVTDFKGKTVWFVPSIKAGNDIAACLRKNGKKVIQLSRKTFDSEYVKTRANDWDFVVTTDISEMGANFKAERVIDPRRCMKPVILTDGEERVILAGPMPVTHSSAAQRRGRIGRNPKNENDQYIYMGEPLENDEDCAHWKEAKMLLDNINTPEGIIPSMFEPEREKVDAIDGEYRLRGEARKTFVDLMRRGDLPVWLAYKVAAEGINYADRKWCFDGIKNNQILEENMEVEIWTKEGERKKLKPRWLDARIYSDPLALKEFKEFAAGRKSLTLNLITEMGRLPTFMTQKARNALDNLAVLHTAEAGGRAYNHALSELPETLETLLLLTLLATVTGGIFLFLMSGKGIGKMTLGMCCIITASILLWYAQIQPHWIAASIILEFFLIVLLIPEPEKQRTPQDNQLTYVVIAILTVVAATMANEMGFLEKTKKDLGLGSITTQESESNILDIDLRPASAWTLYAVATTFLTPMLRHSIENSSVNVSLTAIANQATVLMGLGKGWPLSKMDIGVPLLAIGCYSQVNPITLTAALLLLVAHYAIIGPGLQAKATREAQKRAAAGIMKNPTVDGITVIDLDPIPYDPKFEKQLGQVMLLILCVTQVLMMRTTWALCEALTLATGPISTLWEGNPGRFWNTTIAVSMANIFRGSYLAGAGLLFSIMKNTTSTRRGTGNIGETLGEKWKSRLNALGKSEFQIYKKSGIQEVDRTLAKEGIKRGETDHHAVSRGSAKLRWFVERNMVTPEGKVVDLGCGRGGWSYYCGGLKNVREVKGLTKGGPGHEEPIPMSTYGWNLVRLQSGVDVFFTPPEKCDTLLCDIGESSPNPTIEAGRTLRVLNLVENWLNNNTQFCIKVLNPYMPSVIEKMEALQRKYGGALVRNPLSRNSTHEMYWVSNATGNIVSSVNMISRMLINRFTMKHKKATYEPDVDLGSGTRNIGIESEVPNLDIIGKRIEKIKQEHETSWHYDQDHPYKTWAYHGSYETKQTGSASSMVNGVVRLLTKPWDVVPMVTQMAMTDTTPFGQQRVFKEKVDTRTQEPKEGTKKLMKITAEWLWKELGKKKTPRMCTREEFTRKVRSNAALGAIFTDENKWKSAREAVEDGRFWELVDRERNLHLEGKCETCVYNMMGKREKKLGEFGKAKGSRAIWYMWLGARFLEFEALGFLNEDHWFSRGNSLSGVEGEGLHRLGYILREVGKKEGGAMYADDTAGWDTRITLEDLKNEEMVTNHMKGEHKKLAEAIFKLTYQNKVVRVQRPTPRGTVMDIISRRDQRGSGQVGTYGLNTFTNMEAQLIRQMEGEGIFKSIQHLTATEEIAVQNWLVRVGRERLSRMAISGDDCVVKPIDDRFASALTALNDMGKVRKDIQQWEPSRGWNDWTQVPFCSHHFHELVMKDGRVLVVPCRNQDELIGRARISQGAGWSLKETACLGKSYAQMWTLMYFHRRDLRLAANAICSAVPSHWVPTSRTTWSIHAKHEWMTTEDMLAVWNRVWIQENPWMEDKTPVESWEEVPYLGKREDQWCGSLIGLTSRATWAKNIQTAINQVRSLIGNEEYTDYMPSMKRFRREEEEAGVLW

>DENV2_AHI43694_Mexico

MNNQRKKARSTPFNMLKRERNRVSTVQQLTKRFSLGMLQGRGPLKLFMAFVAFLRFLTIPPTAGILKRWGTIKKSKAINVLRGFRKEIGRMLNILNRRRRTAGVIVMLIPTVMAFHLTTRNGEPHMIVGRQEKGKSLLFKTEDGVNMCTLMAIDLGELCEDTITYKCPLLRQNEPEDIDCWCNSTSTWITYGTCSTTGEHRREKRSVALVPHVGMGLETRTETWMSSEGAWKHVQRIETWILRHPGFTIMAAILAYTIGTTHFQRALIFILLTAVAPSMTMRCIGISNRDFVEGVSGGSWVDIVLEHGSCVTTMAKNKPTLDFELIKTEAKQPATLRKYCIEAKLTNTTTESRCPTQGEPSLNEEQDKRFICKHSMVDRGWGNGCGLFGKGGIVTCAMFTCKKNMEGKVVQPENLEYTIVITPHSGEEHAVGNDTGKHGKEIKITPQSSITEAELTGYGTVTMECSPRTGLDFNEMVLLQMEDKAWLVHRQWFLDLPLPWLPGADTQGSNWIQKETLVTFKNPHAKKQDVVVLGSQEGAMHTALTGATEIQMSSGNLLFTGHLKCRLRMDKLQLKGMSYSMCTGKFKIVKEIAETQHGTIVIRVQYEGDGSPCKIPFEITDLEKRHVLGRLITVNPIVTEKDSPVNIEAEPPFGDSYIIIGVEPGQLKLNWFKKGSSIGQMFETTMRGAKRMAILGDTAWDFGSLGGVFTSIGKALHQVFGAIYGAAFSGVSWTMKILIGVIITWIGMNSRSTSLSVSLVLVGVVILYLGAMVQADSGCVVSWKNKELKCGSGIFITDNVHTWTEQYKFQPESPSKLASAIQKAHEEGICGIRSVTRLENLMWKQITPELNHILSENEVKLTIMTGDIKGIMQAGKRSLRPQPTELKYSWKTWGKAKMLSTESHNQTFLIDGPETAECPNTNRAWNSLEVEDYGFGVFTTNIWLKLREKQDVFCDSKLMSAAIKDNRAVHADMGYWIESALNDTWKMEKASFIEVKSCHWPKSHTLWSNGVLESEMIIPKNFAGPVSQHNYRPGYHTQTAGPWHLGKLEMDFDLCEGTTVVVTEDCGNRGPSLRTTTASGKLITEWCCRSCTLPPLRYRGEDGCWYGMEIRPLKEKEENLVNSLVTAGHGQIDNFSLGVLGMALFLEEMLRTRIGTKHAILLVAASFVTLITGNMSFRDLGRVMVMVGATMTDDIGMGVTYLALLAAFKVRPTFAAGLLLRKLTSKELMMATIGIALLSQSTIPESILELTDALALGMMVLKIVRNMEKYQLAVTIMAISCVPNAVILQNAWKVSCTILAAVSVSPLLLTSSQQKADWIPLALTIKGLNPTAIFLTTLSRTSKKRSWPLNEAVMAVGMVSILASSLLKNDIPMTGPLVAGGLLTVCYVLTGRSADLELERAADVKWEDQAEISGSSPILSITISEDGSMSIKNEEEEQTLTILIRTGLLVISGVFPVSIPITAAAWYLWEVKKQRAGVLWDVPSPPPVEKAELEDGAYRIKQRGILGYSQIGAGVYKEGTFHTMWHVTRGAVLMHRGKRIEPSWADVKKDLISYGGGWKLEGEWKEGEEVQVLALEPGKNPRAVQTKPGIFKTNTGTIGAVSLDFSPGTSGSPIVDRKGKVVGLYGNGVVTRSGAYVSAIAQTEKSIEDNLEIEDDIFRKKRLTIMDLHPGAGKTKRYLPAIVREAIKRGLRTLILAPTRVVAAEMEEALRGLPIRYQTPAIKTEHTGREIVDLMCHATFTMRLLSPVRVPNYNLIIMDEAHFTDPASIAARGYISTRVEMGEAAGIFMTATPPGSRDPFPQSNAPIMDEEREIPERSWNSGHEWVTDFKGKTVWFVPSIKAGNDIAACLRKNGKKVIQLSRKTFDSEYVKTRANDWDFVVTTDISEMGANFKAERVIDPRRCMKPVILTDGEERVILAGPMPVTHSSAAQRRGRIGRNPKNENDQYIYMGEPLENDEDCAHWKEAKMLLDNINTPEGIIPSMFEPEREKVDAIDGEYRLRGEARKTFVDLMRRGDLPVWLAYKVAAEGINYADRKWCFDGIKNNQILEENMEVEIWTKEGERKKLKPRWLDARIYSDPLALKEFKEFAAGRKSLTLNLITEMGRLPTFMTQKARNALDNLAVLHTAEAGGRAYNHALSELPETLETLLLLTLLATVTGGIFLFLMSGKGIGKMTLGMCCIITASILLWYAQIQPHWIAASIILEFFLIVLLIPEPEKQRTPQDNQLTYVVIAILTVVAATMANEMGFLEKTKKDLGLGSITTQESESNILDIDLRPASAWTLYAVATTFVTPMLRHSIENSSVNVSLTAIANQATVLMGLGKGWPLSKMDIGVPLLAIGCYSQVNPITLTAALLLLVAHYAIIGPGLQAKATREAQKRAAAGIMKNPTVDGITVIDLEPIPYDPKFEKQLGQVMLLILCVTQVLMMRTTWALCEALTLATGPISTLWEGNPGRFWNTTIAVSMANIFRGSYLAGAGLLFSIMKNTTNTRRGTGNIGETLGEKWKSRLNALGKSEFQIYKKSGIQEVDRTLAKEGIKRGETDHHAVSRGSAKLRWFVERNMVTPEGKVVDLGCGRGGWSYYCGGLKDVREVKGLTKGGPGHEEPIPMSTYGWNLVRLQSGIDVFFTPPEKCDTLLCDIGESSPNPTIEAGRTLRVLNLVENWLNNNTQFCIKVLNPYMPSVIEKMETLQRKYGGALVRNPLSRNSTHEMYWVSNATGNIVSSVNMISRMLINRFTMKHKKATYEPDVDLGSGTRNIGIESEIPNLDIIGKRIEKIKQEHETSWHYDQDHPYKTWAYHGSYETKQTGSASSMVNGVVRLLTKPWDVVPMVTQMAMTDTTPFGQQRVFKEKVDTRTQEPKEGTKKLMKITAEWLWKELGKKKTPRMCTREEFTRKVRSNAALGAIFTDENKWKSAREAVEDGRFWELVDRERSLHLEGKCETCVYNMMGKREKKLGEFGKAKGSRAIWYMWLGARFLEFEALGFLNEDHWFSRGNSLSGVEGEGLHRLGYILRDVGKKEGGAMYADDTAGWDTRITLEDLKNEEMVTNHMKGEHKKLAEAIFKLTYQNKVVRVQRPTPRGTVMDIISRRDQRGSGQVGTYGLNTFTNMEAQLIRQMEGEGIFKNIQHLTATEEIAVQNWLARVGRERLSRMAISGDDCVVKPIDDRFASALTALNDMGKVRKDIQQWEPSRGWNDWTQVPFCSHHFHELVMKDGRVLVVPCRNQDELIGRARISQGAGWSLKETACLGKSYAQMWTLMYFHRRDLRLAANAICSAVPSHWVPTSRTTWSIHAKHEWMTTEDMLAVWNRVWIQENPWMEDKTPVESWEEVPYLGKREDQWCGSLIGLTSRATWAKNIQTAINQVRSLIGNEEYTDYMPSMKRFRKEEEEAGVLW

>DENV2_AHI43693_Mexico

MNNQRKKARSTPFNMLKRERNRVSTVQQLTKRFSLGMLQGRGPLKLFMAFVAFLRFLTIPPTAGILKRWGTIKKSKAINVLRGFRKEIGRMLNILNRRRRTAGVIVMLIPTVMAFHLTTRNGEPHMIVGRQEKGKSLLFKTEDGVNMCTLMAIDLGELCEDTITYKCPLLRQNEPEDIDCWCNSTSTWITYGTCSTTGEHRREKRSVALVPHVGMGLETRTETWMSSEGAWKHVQRIETWILRHPGFTIMAAILAYTIGTTHFQRALIFILLTAVAPSMTMRCIGISNRDFVEGVSGGSWVDIVLEHGSCVTTMAKNKPTLDFELIKTEAKQPATLRKYCIEAKLTNTTTESRCPTQGEPSLNEEQDKRFICKHSMVDRGWGNGCGLFGKGGIVTCAMFTCKKNMEGKVVQPENLEYTIVITPHSGEEHAVGNDTGKHGKEIKITPQSSITEAELTGYGTVTMECSPRTGLDFNEMVLLQMEDKAWLVHRQWFLDLPLPWLPGADTQGSNWIQKETLVTFKNPHAKKQDVVVLGSQEGAMHTALTGATEIQMSSGNLLFTGHLKCRLRMDKLQLKGMSYSMCTGKFKIVKEIAETQHGTIVIRVQYEGDGSPCKIPFEITDLEKRHVLGRLITVNPIVTEKDSPVNIEAEPPFGDSYIIIGVEPGQLKLNWFKKGSSIGQMFETTMRGAKRMAILGDTAWDFGSLGGVFTSIGKALHQVFGAIYGAAFSGVSWTMKILIGVIITWIGMNSRSTSLSVSLVLVGVVILYLGAMVQADSGCVVSWKNKELKCGSGIFITDNVHTWTEQYKFQPESPSKLASAIQKAHEEGICGIRSVTRLENLMWKQITPELNHILSENEVKLTIMTGDIKGIMQAGKRSLRPQPTELKYSWKTWGKAKMLSTESHNQTFLIDGPETAECPNTNRAWNSLEVEDYGFGVFTTNIWLKLREKQDVFCDSKLMSAAIKDNRAVHADMGYWIESALNDTWKMEKASFIEVKSCHWPKSHTLWSNGVLESEMIIPKNFAGPVSQHNYRPGYHTQTAGPWHLGKLEMDFDLCEGTTVVVTEDCGNRGPSLRTTTASGKLITEWCCRSCTLPPLRYRGEDGCWYGMEIRPLKEKEENLVNSLVTAGHGQIDNFSLGVLGMALFLEEMLRTRIGTKHAILLVAASFVTLITGNMSFRDLGRVMVMVGATMTDDIGMGVTYLALLAAFKVRPTFAAGLLLRKLTSKELMMATIGIALLSQSTIPESILELTDALALGMMVLKIVRNMEKYQLAVTIMAISCVPNAVILQNAWKVSCTILAAVSVSPLLLTSSQQKADWIPLALTIKGLNPTAIFLTTLSRTSKKRSWPLNEAVMAVGMVSILASSLLKNDIPMTGPLVAGGLLTVCYVLTGRSADLELERAADVKWEDQAEISGSSPILSITISEDGSMSIKNEEEEQTLTILIRTGLLVISGVFPVSIPITAAAWYLWEVKKQRAGVLWDVPSPPPVEKAELEDGAYRIKQRGILGYSQIGAGVYKEGTFHTMWHVTRGAVLMHRGKRIEPSWADVKKDLISYGGGWKLEGEWKEGEEVQVLALEPGKNPRAVQTKPGIFKTNTGTIGAVSLDFSPGTSGSPIVDRKGKVVGLYGNGVVTRSGAYVSAIAQTEKSIEDNPEIEDDIFRKKRLTIMDLHPGAGKTKRYLPAIVREAIKRGLRTLILAPTRVVAAEMEEALRGLPIRYQTPAIKTEHTGREIVDLMCHATFTMRLLSPVRVPNYNLIIMDEAHFTDPASIAARGYISTRVEMGEAAGIFMTATPPGSRDPFPQSNAPIMDEEREIPERSWNSGHEWVTDFKGKTVWFVPSIKAGNDIAACLRKNGKKVIQLSRKTFDSEYVKTRANDWDFVVTTDISEMGANFKAERVIDPRRCMKPVILTDGEERVILAGPMPVTHSSAAQRRGRIGRNPKNENDQYIYMGEPLENDEDCAHWKEAKMLLDNINTPEGIIPSMFEPEREKVDAIDGEYRLRGEARKTFVDLMRRGDLPVWLAYKVAAEGINYADRKWCFDGIKNNQILEENMEVEIWTKEGERKKLKPRWLDARIYSDPLALKEFKEFAAGRKSLTLNLITEMGRLPTFMTQKARNALDNLAVLHTAEAGGRAYNHALSELPETLETLLLLTLLATVTGGIFLFLMSGKGIGKMTLGMCCIITASILLWYAQIQPHWIAASIILEFFLIVLLIPEPEKQRTPQDNQLTYVVIAILTVVAATMANEMGFLEKTKKDLGLGSITTQESESNILDIDLRPASAWTLYAVATTFVTPMLRHSIENSSVNVSLTAIANQATVLMGLGKGWPLSKMDIGVPLLAIGCYSQVNPITLTAALLLLVAHYAIIGPGLQAKATREAQKRAAAGIMKNPTVDGITVIDLEPIPYDPKFEKQLGQVMLLILCVTQVLMMRTTWALCEALTLATGPISTLWEGNPGRFWNTTIAVSMANIFRGSYLAGAGLLFSIMKNTTNTRRGTGNIGETLGEKWKSRLNALGKSEFQIYKKSGIQEVDRTLAKEGIKRGETDHHAVSRGSAKLRWFVERNMVTPEGKVVDLGCGRGGWSYYCGGLKDVREVKGLTKGGPGHEEPIPMSTYGWNLVRLQSGIDVFFTPPEKCDTLLCDIGESSPNPTIEAGRTLRVLNLVENWLNNNTQFCIKVLNPYMPSVIEKMETLQRKYGGALVRNPLSRNSTHEMYWVSNATGNIVSSVNMISRMLINRFTMKHKKATYEPDVDLGSGTRNIGIESEIPNLDIIGKRIEKIKQEHETSWHYDQDHPYKTWAYHGSYETKQTGSASSMVNGVVRLLTKPWDVVPMVTQMAMTDTTPFGQQRVFKEKVDTRTQEPKEGTKKLMKITAEWLWKELGKKKTPRMCTREEFTRKVRSNAALGAIFTDENKWKSAREAVEDGRFWELVDRERNLHLEGKCETCVYNMMGKREKKLGEFGKAKGSRAIWYMWLGARFLEFEALGFLNEDHWFSRGNSLSGVEGEGLHRLGYILRDVGKKEGGAMYADDTAGWDTRITLEDLKNEEMVTNHMKGEHKKLAEAIFKLTYQNKVVRVQRPTPRGTVMDIISRRDQRGSGQVGTYGLNTFTNMEAQLIRQMEGEGIFKNIQHLTATEEIAVQNWLARVGRERLSRMAISGDDCVVKPIDDRFASALTALNDMGKVRKDIQQWEPSRGWNDWTQVPFCSHHFHELVMKDGRVLVVPCRNQDELIGRARISQGAGWSLKETACLGKSYAQMWTLMYFHRRDLRLAANAICSAVPSHWVPTSRTTWSIHAKHEWMTTEDMLAVWNRVWIQENPWMEDKTPVESWEEVPYLGKREDQWCGSLIGLTSRATWAKNIQTAINQVRSLIGNEEYTDYMPSMKRFRKEEEEAGVLW

>DENV2_AHI43692_Mexico

MNNQRKKARSTPFNMLKRERNRVSTVQQLTKRFSLGMLQGRGPLKLFMALVAFLRFLTIPPTAGILKRWGTIKKSKAINVLRGFRKEIGRMLNILNRRRRTAGVIVMLIPTAMAFHLTTRNGEPHMIVGRQEKGKSLLFKTEDGVNMCTLMAIDLGELCEDTITYKCPLLRQNEPEDIDCWCNSTSTWVTYGTCTTTGEHRREKRSVALVPHVGMGLETRTETWMSSEGAWKHVQRIETWILRHPGFTIMAAILAYTIGTTHFQKALIFILLTAVAPSMTMRCIGISNRDFVEGVSGGSWVDIVLEHGSCVTTMAKNKPTLDFELIKTEAKQPATLRKYCIEAKLTNTTTESRCPTQGEPSLNEEQDKRFICKHSMVDRGWGNGCGLFGKGGIVTCAMFTCKKNMEGKVVQPENLEYTIVITPHSGEEHAVGNDTGKHGKEIKITPQSSITEAELTGYGTVTMECSPRTGLDFNEMVLLQMEDKAWLVHRQWFLDLPLPWLPGADTQGSNWIQKETLVTFKNPHAKKQDVVVLGSQEGAMHTALTGATEIQMSSGNLLFTGHLKCRLRMDKLQLKGMSYSMCTGKFKIVKEIAETQHGTIVIRVQYEGDGSPCKIPFEITDLEKRHVLGRLITVNPIVTEKDSPVNIEAEPPFGDSYIIIGVEPGQLKLNWFKKGSSIGQMFETTMRGAKRMAILGDTAWDFGSLGGVFTSIGKALHQVFGAIYGAAFSGVSWTMKILIGVIITWIGMNSRSTSLSVSLVLVGVVTLYLGAMVQADSGCVVSWKNKELKCGSGIFITDNVHTWTEQYKFQPESPSKLASAIQKAHEEGICGIRSVTRLENLMWKQITPELNHILSENEVKLTIMTGDIKGIMQAGKRSLRPQPTELKYSWKTWGKAKMLSTEPHNQTFLIDGPETAECPNTNRAWNSLEVEDYGFGVFTTNIWLKLREKQDVFCDSKLMSAAIKDNRAVHADMGYWIESALNDTWKMEKASFIEVKSCHWPKSHTLWSNGVLESEMIIPKNFAGPVSQHNYRPGYHTQTAGPWHLGKLEMDFDLCEGTTVVVTEDCGNRGPSLRTTTASGKLITEWCCRSCTLPPLRYRGEDGCWYGMEIRPLKEKEENLVNSLVTAGHGQIDNFSLGVLGMALFLEEMLRTRIGTKHAILLVAVSFVTLITGNMSFRDLGRVMVMVGATMTDDIGMGVTYLALLAAFKVRPTFAAGLLLRKLTSKELMMATIGIALLSQSTIPETILELTDALALGMMVLKIVRSMEKYQLAVTIMAISCVPNAVILQNAWKVSCTILAAVSVSPLLLTSSQQKADWIPLALTIKGLNPTAIFLTTLSRTSKKRSWPLNEAIMAVGMVSILASSLLKNDIPMTGPLVAGGLLTVCYVLTGRSADLELERAADVKWEDQAEISGSSPILSITISEDGSMSIKNEEEEQTLTILIRTGLLVISGVFPVSIPITAAAWYLWEVKKQRAGVLWDVPSPPPVEKAELEDGAYRIKQRGILGYSQIGAGVYKEGTFHTMWHVTRGAVLMHRGKRIEPSWADVKKDLISYGGGWKLEGEWKEGEEVQVLALEPGKNPRAVQTKPGIFKTNTGTIGAVSLDFSPGTSGSPIVDRKGKVVGLYGNGVVTRSGAYVSAIAQTEKSIEDNPEIEDDIFRKKRLTIMDLHPGAGKTKRYLPAIVREAIKRGLRTLILAPTRVVAAEMEEALRGLPIRYQTPAIKTQHTGREIVDLMCHATFTMRLLSPVRVPNYNLIIMDEAHFTDPASIAARGYISTRVEMGEAAGIFMTATPPGSRDPFPQSNAPIMDEEREIPERSWNSGHEWVTDFKGKTVWFVPSIKAGNDIAACLRKNGKKVIQLSRKTFDSEYVKTRANDWDFVVTTDISEMGANFKAERVIDPRRCMKPVILTDGEERVILAGPMPVTHSSAAQRRGRIGRNPKNENDQYIYMGEPLENDEDCAHWKEAKMLLDNINTPEGIIPSMFEPEREKVDAIDGEYRLRGEARKTFVDLMRRGDLPVWLAYKVAAEGINYADRKWCFDGIKNNQILEENIEVEIWTKEGERKKLKPRWLDARIYSDPLALKEFKEFAAGRKSLTLNLITEMGRLPTFMTQKARNALDNLAVLHTAEAGGRAYNHALSELPETLETLLLLTLLATVTGGIFLFLMSGKGVGKMTLGMCCIITASILLWYAQIQPHWIAASIILEFFLIVLLIPEPEKQRTPQDNQLTYVVIAILTVVAATMANEMGFLEKTKKDLGLGSITTQESESNILDIDLRPASAWTLYAVATTFVTPMLRHSIENSSVNVSLTAIANQATVLMGLGKGWPLSKMDIGVPLLAIGCYSQVNPITLTAALLLLVAHYAIIGPGLQAKATREAQKRAAAGIMKNPTVDGITVIDLEPIPYDPKFEKQLGQVMLLILCVTQVLMMRTTWALCEALTLATGPISTLWEGNPGRFWNTTIAVSMANIFRGSYLAGAGLLFSIMKNTTNTRRGTGNIGETLGEKWKSRLNALGKSEFQVYKKSGIQEVDRTLAKEGIKRGETDHHAVSRGSAKLRWFVERNMVTPEGKVVDLGCGRGGWSYYCGGLKNVREVKGLTKGGPGHEEPIPMSTYGWNLVRLQSGVDVFFTPPEKCDTLLCDIGESSPNPTIEAGRTLRVLNLVENWLNNNTQFCIKVLNPYMPSVIEKMEALQRKYGGALVRNPLSRNSTHEMYWVSNATGNIVSSVNMISRMLINRFTMKHKKATYEPDVDLGSGTRNIGIESEIPNLDIIGKRIEKIKQEHETSWHYDQDHPYKTWAYHGSYETKQTGSASSMVNGVVRLLTKPWDVVPMVTQMAMTDTTPFGQQRVFKEKVDTRTQEPKEGTKKLMKITAEWLWKELGKKKTPRMCTREEFTRKVRSNAALGAIFTDENKWKSAREAVEDGRFWELVDRERNLHLEGKCETCVYNMMGKREKKLGEFGKAKGSRAIWYMWLGARFLEFEALGFLNEDHWFSRGNSLSGVEGEGLHRLGYILRDVGKKEGGAMYADDTAGWDTRITLEDLKNEEMVTNHMKGEHKKLAEAIFKLTYQNKVVRVQRPTPRGTVMDIISRRDQRGSGQVGTYGLNTFTNMEAQLIRQMEGEGIFKNIQHLTATEEIAVQNWLARVGRERLSRMAISGDDCVVKPIDDRFASALTALNDMGKVRKDIQQWEPSRGWNDWTQVPFCSHHFHELVMKDGRVLVVPCRNQDELIGRARISQGAGWSLKETACLGKSYAQMWTLMYFHRRDLRLAANAICSAVPPHWVPTSRTTWSIHAKHEWMTTEDMLAVWNRVWIQENPWMEDKTPVESWEEVPYLGKREDQWCGSLIGLTSRATWAKNIQTAINQVRSLIGNEEYTDYMPSMKRFRREEEEAGVLW

>DENV2_AHI43691_Mexico

MNNQRKKARSTPFNMLKRERNRVSTVQQLTKRFSLGMLQGRGPLKLFMALVAFLRFLTIPPTAGILKRWGTIKKSKAINVLRGFRKEIGRMLNILNRRRRTAGVIVMLIPTAMAFHLTTRNGEPHMIVGRQEKGKSLLFKTEDGVNMCTLMAIDLGELCEDTITYKCPLLRQNEPEDIDCWCNSTSTWVTYGTCTTTGEHRREKRSVALVPHVGMGLETRTETWMSSEGAWKHVQRIETWILRHPGFTIMAAILAYTIGTTHFQKALIFILLTAVAPSMTMRCIGISNRDFVEGVSGGSWVDIVLEHGSCVTTMAKNKPTLDFELIKTEAKQPATLRKYCIEAKLTNTTTESRCPTQGEPSLNEEQDKRFICKHSMVDRGWGNGCGLFGKGGIVTCAMFTCKKNMEGKVVQPENLEYTIVITPHSGEEHAVGNDTGKHGKEIKITPQSSITEAELTGYGTVTMECSPRTGLDFNEMVLLQMEDKAWLVHRQWFLDLPLPWLPGADTQGSNWIQKETLVTFKNPHAKKQDVVVLGSQEGAMHTALTGATEIQMSSGNLLFTGHLKCRLRMDKLQLKGMSYSMCTGKFKIVKEIAETQHGTIVIRVQYEGDGSPCKIPFEITDLEKRHVLGRLITVNPIVTEKDSPVNIEAEPPFGDSYIIIGVEPGQLKLNWFKKGSSIGQMFETTMRGAKRMAILGDTAWDFGSLGGVFTSIGKALHQVFGAIYGAAFSGVSWTMKILIGVIITWIGMNSRSTSLSVSLVLVGVVTLYLGAMVQADSGCVVSWKNKELKCGSGIFITDNVHTWTEQYKFQPESPSKLASAIQKAHEEGICGIRSVTRLENLMWKQITPELNHILSENEVKLTIMTGDIKGIMQAGKRSLRPQPTELKYSWKTWGKAKMLSTESQNQTFLIDGPETAECPNTNRAWNSLEVEDYGFGVFTTNIWLKLREKQDVFCDSKLMSAAIKDNRAVHADMGYWIESALNDTWKMEKASFIEVKSCHWPKSHTLWSNGVLESEMIIPKNFAGPVSQHNYRPGYHTQTAGPWHLGKLEMDFDLCEGTTVVVTEDCGNRGPSLRTTTASGKLITEWCCRSCTLPPLRYRGEDGCWYGMEIRPLKEKEENLVNSLVTAGHGQIDNFSLGVLGMALFLEEMLRTRIGTKHAILLVAVSFVTLITGNMSFRDLGRVMVMVGAAMTDDIGMGVTYLALLAAFKVRPTFAAGLLLRKLTSKELMMATIGIALLSQSTIPETILELTDALALGMMVLKIVRNMEKYQLAVTIMAISCVPNAVILQNAWKVSCTILAAVSVSPLLLTSSQQKADWIPLALTIKGLNPTAIFLTTLSRTSKKRSWPLNEAIMAVGMVSILASSLLKNDIPMTGPLVAGGLLTVCYVLTGRSADLELERAADVKWEDQAEISGSSPILSITISEDGSMSIKNEEEEQTLTILIRTGLLVISGVFPVSIPITAAAWYLWEVKKQRAGVLWDVPSPPPVEKAELEDGAYRIKQRGILGYSQIGAGVYKEGTFHTMWHVTRGAVLMHRGKRIEPSWADVKKDLISYGGGWKLEGEWKEGEEVQVLALEPGKNPRAVQTKPGIFKTNTGTIGAVSLDFSPGTSGSPIVDRKGKVVGLYGNGVVTRSGAYVSAIAQTEKSIEDNPEIEDDIFRKKRLTIMDLHPGAGKTKRYLPAIVREAIKRGLRTLILAPTRVVAAEMEEALRGLPIRYQTPAIKTEHTGREIVDLMCHATFTMRLLSPVRVPNYNLIIMDEAHFTDPASIAARGYISTRVEMGEAAGIFMTATPPGSRDPFPQSNAPIMDEEREIPERSWNSGHEWVTDFKGKTVWFVPSIKAGNDIAACLRKNGKKVIQLSRKTFDSEYVKTRANDWDFVVTTDISEMGANFKAERVIDPRRCMKPVILTDGEERVILAGPMPVTHSSAAQRRGRIGRNPKNENDQYIYMGEPLENDEDCAHWKEAKMLLDNINTPEGIIPSMFEPEREKVDAIDGEYRLRGEARKTFVDLMRRGDLPVWLAYKVAAEGINYADRKWCFDGIKNNQILEENIEVEIWTKEGERKKLKPRWLDARVYSDPLALKEFKEFAAGRKSLTLNLITEMGRLPTFMTQKARNALDNLAVLHTAEAGGRAYNHALSELPETLETLLLLTLLATVTGGIFLFLMSGKGIGKMTLGMCCIITASILLWYAQIQPHWIAASIILEFFLIVLLIPEPEKQRTPQDNQLTYVVIAILTVVAATMANEMGFLEKTKKDLGLGSITTQESESNILDIDLRPASAWTLYAVATTFVTPMLRHSIENSSVNVSLTAIANQATVLMGLGKGWPLSKMDIGVPLLAIGCYSQVNPITLTAALLLLVAHYAIIGPGLQAKATREAQKRAAAGIMKNPTVDGITVIDLEPIPYDPKFEKQLGQVMLLILCVTQVLMMRTTWALCEALTLATGPISTLWEGNPGRFWNTTIAVSMANIFRGSYLAGAGLLFSIMKNTTNTRRGTGNIGETLGEKWKSRLNALGKSEFQVYKKSGIQEVDRTLAKEGIKRGETDHHAVSRGSAKLRWFVERNMVTPEGKVVDLGCGRGGWSYYCGGLKNVREVKGLTKGGPGHEEPIPMSTYGWNLVRLQSGVDVFFTPPEKCDTLLCDIGESSPNPTIEAGRTLRVLNLVENWLNNNTQFCIKVLNPYMPSVIEKMEALQRKYGGALVRNPLSRNSTHEMYWVSNATGNIVSSVNMISRMLINRFTMKHKKATYEPDVDLGSGTRNIGIESEIPNLDIIGKRIEKIKQEHETSWHYDQDHPYKTWAYHGSYETKQTGSASSMVNGVVRLLTKPWDVVPMVTQMAMTDTTPFGQQRVFKEKVDTRTQEPKEGTKKLMKITAEWLWKELGKKKTPRMCTRDEFTRKVRSNAALGAIFTDENKWKSAREAVEDGRFWELVDRERNLHLEGKCETCVYNMMGKREKKLGEFGKAKGSRAIWYMWLGARFLEFEALGFLNEDHWFSRGNSLSGVEGEGLHRLGYILRDVGKKEGGAMYADDTAGWDTRITLEDLKNEEMVTNHMKGEHKKLAEAIFKLTYQNKVVRVQRPTPRGTVMDIISRRDQRGSGQVGTYGLNTFTNMEAQLIRQMEGEGIFKSIQHLTATEEIAVQNWLARVGRERLSRMAISGDDCVVKPIDDRFASALTALNDMGKVRKDIQQWEPSRGWNDWTQVPFCSHHFHELVMKDGRVLVVPCRNQDELIGRARISQGAGWSLKETACLGKSYAQMWTLMYFHRRDLRLAANAICSAVPPHWVPTSRTTWSIHAKHEWMTTEDMLAVWNRVWIQENPWMEDKTPVESWEEVPYLGKREDQWCGSLIGLTSRATWAKNIQTAINQVRSLIGNEEYTDYMPSMKRFRREEEEAGVLW

>DENV2_AHG25313_Philippines

MNNQRKKARNTPFNMLKRERNRVSTVQQLTKRFSLGMLQGRGPLKLFMALVAFLRFLTIPPTVGILKRWGTIKKSKAINVLRGFRKEIGRMLNILNRRRRTAGMIIMLIPTVMAFHLTTRNGEPHMIVSRQEKGKSLLFKTEDGVNMCTLMAMDLGELCEDTITYKCPLLRQNEPEDIDCWCNSTSTWVTYGTCTTTGEHRREKRSVALVPHVGMGLETRTETWMSSEGAWKHAQRIETWILRHPGFTLMAAILAYTIGTTNFQRALIFILLTAVAPSMTMRCIGISNRDFVEGVSGGSWVDIVLEHGSCVTTMAKNKPTLDFELIKTEAKHPATLRKYCIEAKLTNTTTASRCPTQGEPSLNEEQDKRFVCKHSMVDRGWGNGCGLFGKGGIVTCAMFTCKKNMEGKIVQPENLEYTIVITPHSGEEHAVGNDTGKHGKEIKITPQSSITEAELTGYGTVTMECSPRTGLGFNEMVLLQMEDKAWLVHRQWFLDLPLPWLPGADTQGSNWIQKETLVTFKNPHAKKQDVVVLGSQEGAMHTALTGATEIQMSSGNLLFTGHLKCRLRMDKLQLKGMSYSMCTGKFKVVKEIAETQHGTIVVRVQYEGDGSPCKIPFEIMDLEKRHVLGRLITVNPIVTEKDSPVNIEAEPPFGDSYVIIGVEPGQLKLSWFKKGSSIGQMFETTMRGAKRMAILGDTAWDFGSLGGVFTSIGKALHQVFGAIYGAAFSGVSWTMKILIGVIITWIGMNSRSTSLSVSLVLVGVVTLYLGVVVQADSGCVVSWRNKELKCGSGIFITDNVHTWTEQYKFQPESPSKLASAIQKAHQEGICGIRSVTRLENLMWKQITPELNHILSENEVKLTIMTGDIKGIMQAGKRSLRPQPTELRYSWKTWGKAKILSTESHNQTFLIDGPETAECPNTNRAWNSLEVEDYGFGVFTTNIWLRLREKQDAFCDSKLMSAAIKDNRAVHADMGYWIESALNDTWKIEKASFIEVKSCHWPKSHTLWSNGVLESEMIIPKNFAGPVSQHNYRPGYHTQTAGPWHLGKLEMDFDFCKGTTVVVTEDCGNRGPSLRTTTASGKLITEWCCRSCTLPPLRYRGEDGCWYGMEIRPLKEKEENLVNSLVTAGHGQIDNFSLGVLGMALFLEEMLRTRVGTKHAILLVAVSFVTLITGNMSFRDLGRVMVMVGATMTDDIGMGVTYLALLAAFKVRPTFAAGLLLRKLTSKELMMTTIGIVLLSQSTIPETILELTDALALGMMVLKIVRNMEKYQLAVTIMAILCVPNAVILQNAWKVSCTILAVVSVSPLLLTSSQQKADWIPLALTIKGLNPTAIFLTTLSRTSKKRSWPLNEAIMAVGMVSILASSLLKNDIPMTGPLVAGGLLTVCYVLTGRSADLELEKAADVRWEDQAEISGSSPILSIIISEDGSMSIKNEEEEQTLTILIRTGLLVISGLFPVSIPITAAAWYLWEVKKQRAGVLWDVPSPPPVGKAELEDGAYRIKQKGILGYSQIGAGVYKEGTFHTMWHVTRGAVLMHKGKRIEPSWADVKKDLISYGGGWKLEGEWKEGEEVQVLALEPGKNPRAVQTKPGLFKTNAGTIGAVSLDFSPGTSGSPIIDKKGKVVGLYGNGVVTRNGSYVSAIAQTEKSIEDNPEIEDDIFRKKRLTIMDLHPGAGKTKRYLPAIVREAIKRGLRTLILAPTRVVAAEMEEALRGLPIRYQTPAIRAEHTGREIVDLMCHATFTMRLLSPIRVPNYNLIIMDEAHFTDPASIAARGYISTRVEMGEAAGIFMTATPPGSRDPFPQSNAPIMDEEREIPERSWNSGHEWVTDFKGKTVWFVPSIKAGNDIAACLRKNGKKVIQLSRKTFDSEYVKTRTNDWDFVVTTDISEMGANFKAERVIDPRRCMKPVILTDGEERVILAGPMPVTHSSAAQRRGRIGRNPKNENDQYIYMGEPLENDEDCAHWKEAKMLLDNINTPEGIIPSMFEPEREKVDAIDGEYRLRGEARKTFVDLMRRGDLPVWLAYKVAAEGINYADRRWCFDGIRNNQILEENVEVEIWTKEGERKKLKPRWLDARIYSDPLALKEFKEFAAGRKSLTLNLITEMGRLPTFMTQKARNALDNLAVLHTAEAGGRAYNHALSELPETLETLLLLTLLATVTGGIFLFLMSGKGIGKMTLGMCCIITASVLLWYAQIQPHWIAASIILEFFLIVLLIPEPEKQRTPQDNQLTYVVIAILTVVAATMANEMGFLEKTKKDLGLGSITTQQPESNILDIDLRPASAWTLYAVATTFITPMLRHSIENSSVNVSLTAIANQATVLMGLGKGWPLSKMDIGVPLLAIGCYSQVNPITLTAALLLLVAHYAIIGPGLQAKATREAQKRAAAGIMKNPTVDGITVIDLDPIPYDPKFEKQLGQVMLLVLCVTQVLMMRTTWALCEALTLATGPISTLWEGNPGRFWNTTIAVSMANIFRGSYLAGAGLLFSIMKNTTNTRRGTGNIGETLGEKWKSRLNALGKSEFQIYKKSGIQEVDRTLAKEGIKRGETDHHAVSRGSAKLRWFVERNMITPEGKVVDLGCGRGGWSYYCGGLKNVREVKGLTKGGPGHEEPIPMSTYGWNLVRLQSGVDVFFTPPEKCDTLLCDIGESSPNPTVEAGRTLRVLNLVENWLNNNTQFCIKVLNPYMPSVIEKMEALQRKYGGALVRNPLSRNSTHEMYWVSNASGNIVSSVNMISRMLINRFTMKHKKATYEPDVDLGSGTRNIGIESEVPNLDIIGKRIEKIKQEHETSWHYDQDHPYKTWAYHGSYETKQTGSASSMVNGVVRLLTKPWDVIPMVTQMAMTDTTPFGQQRVFKEKVDTRTQEPKEGTKKLMKITAEWLWKELGKKKTPRMCTREEFTRKVRSNAALGAIFTDENKWKSAREAVEDSRFWELVDKERNLHLEGKCETCVYNMMGKREKKLGEFGKAKGSRAIWYMWLGARFLEFEALGFLNEDHWFSRENSLSGVEGEGLHKLGYILRDVSKKEGGAMYADDTAGWDTRITLEDLKNEEMVTNHMEGEHKKLAEAIFKLTYQNKVVRVQRPTPRGTVMDIISRRDQRGSGQVGTYGLNTFTNMEAQLIRQMEGEGVFKSIQHLTATEEIAVQNWLARVGRERLSRMAISGDDCVVKPLDDRFASALTALNDMGKVRKDIQQWEPSKGWNDWTQVPFCSHHFHELIMKDGRVLVVPCRNQDELIGRARISQGAGWSLRETACLGKSYAQMWSLMYFHRRDLRLAANAICSAVPSHWVPTSRTTWSIHAKHEWMTTEDMLTVWNRVWIQENPWMEDKTPVESWEEIPYLGKREDQWCGSLIGLTSRATWAKNIQTAINQVRSLIGNEEYTDYMPSMKRFRREEEEAGVLW

>DENV2_AHG23170_Cambodia

MNNQRKKAKNTPFNMLKRERNRVSTVQQLTKRFSLGMLQGRGPLKLFMALVAFLRFLTIPPTAGILKRWGTIKKSKAINVLRGFRKEIGRMLNILNRRRRSAGMIIMLIPTVMAFHLTTRNGEPHMIVGIQEKGKSLLFKTEDGVNMCTLMAMDLGELCEDTITYKCPLLRQNEPEDIDCWCNSTSTWVTYGTCTTTGEHRREKRSVALVPHVGMGLETRTETWMSSEGAWKHAQRIETWILRHPGFTIMAAILAYTIGTTHFQRVLIFILLTAVAPSMTMRCIGISNRVFVEGVSGGSWVDIVLEHGSCVTTMAKNKPTLDFELIKTEAKQPATLRKYCIEAKLTNTTTESRCPTQGEPSLKEEQDKRFVCKHSMVDRGWGNGCGLFGKGGIVTCAMFTCKKNMEGKIVQPENLEYTIVVTPHSGEEHAVGNDTGKHGMEIKVTPQSSITEAELTGYGTVTMECSPRTGLDFNEMVLLQMENKAWLVHRQWFLDLPLPWLPGADKQESNWIQKETLVTFKNPHAKKQDVVVLGSQEGAMHTALTGATEIQMSSGNLLFTGHLKCRLRMDKLQLKGMSYSMCTGKFKVVKEIAETQHGTIVIRVQYEGDGSPCKIPFEIMDLEKRYVLGRLITVNPIVTEKDSPVNIEAEPPFGDSYIIIGVEPGQLKLNWFKKGSSIGQMFETTMRGAKRMAILGDTAWDFGSLGGVFTSIGKALHQVFGAIYGVAFSGVSWTMKILIGVIITWIGMNSRSTSLSVSLVLVGIVTLYLGVMVQADSGCVVSWKNKELKCGSGIFITDNVHTWTEQYKFQPESPSKLASAIQKAQEEGICGIRSVTRLENLMWKQITPELNHILAENEVKLTIMTGDIKGIMQAGKRSLRPQPTELKYSWKTWGKAKMLSTESYNQTFLIDGPETAECPNTNRAWNSLEVEDYGFGVFTTNIWLKLKEKQDAFCDSKLMSAAIKDNRAVHADMGYWIESALNDTWKIEKASFIEVKNCHWPKSHTLWSNGVLESEMIIPKNLAGPVSQHNYRPGYHTQIAGPWHLGKLEMDFDFCDGTTVIVTEDCGNRGPSLRTTTASGKLITEWCCRSCTLPPLRYRGEDGCWYGMEIRPLKEKEENLVNSLVTAGHGQVDNFSLGVLGMALFLEEMLRTRVGTKHAILLVAVSFVTLITGNMSFKDLGRVVVMVGATMADDIGMGVTYLALLAAFKVRPTFAAGLLLRKLTSKELMMTTIGIVLLSQSTIPETVLELTDALALGMMVLKIVRNMEKYQLAVTIMAILCVPNAVILQNAWKVSCTILAVVSVSPLLLTSSQQKTDWIPLALTIKGLNPTAIFLTTLSRTNKKRSWPLNEAIMAVGMVSILASSLLKNDIPMTGPLVAGGLLTVCYVLTGRSADLELERATDVKWEDQAEISGSSPILSITISEDGSMSIKNEEEEQTLTILIRTGLLVISGLFPISIPITAAAWYLWEVKKQRAGVLWDVPSPPPMGKAELEDGAYRIKQKGILGYSQIGAGVYKEGTFHTMWHVTRGAVLMHKGKRIEPSWADVKKDLISYGGGWKLEGEWKEGEEVQVLALEPGKNPRAVQTKPGLFKTNTGTIGAVSLDFSPGTSGSPIIDKKGKVVGLYGNGVVTRSGAYVSAIAQTEKSIEDNPEIEDDIFRKRRLTIMDLHPGAGKTKRYLPAIVREAIKRGLRTLILAPTRVVAAEMEEALRGLPIRYQTPAIRAEHTGREIVDLMCHATFTMRLLSPVRVPNYNLIIMDEAHFTDPASIAARGYISTRVEMGEAAGIFMTATPPGSRDPFPQSNAPIIDEEREIPERSWNSGHEWVTDFKGKTVWFVPSIKAGNDIAACLRKNGKKVIQLSRKTFDSEYVKTRTNDWDFVVTTDISEMGANFKAERVIDPRRCMKPVILTDGEERVILAGPMPVTHSSAAQRRGRIGRNPKNENDQYIYMGEPLENDEDCAHWKEAKMLLDNINTPEGIIPSMFEPEREKVDAIDGEYRLRGEARKTFVDLMRRGDLPVWLAYKVAAEGINYADRRWCFDGIKNNQILEENVEVEIWTKEGERKKLKPRWLDARIYSDPLALKEFKEFAAGRKSLTLNLITEMGRLPTFMTQKTRDALDNLAVLHTAEAGGRAYNHALSELPETLETLLLLTLLATVTGGIFLFLMSGRGIGKMTLGMCCIITASILLWYAQIQPHWIAASIILEFFLIVLLIPEPEKQRTPQDNQLTYVVIAILTVVAATMANEMGFLEKTKKDLGLGSIATQQPESNILDIDLRPASAWTLYAVATTFVTPMLRHSIENSSVNVSLTAIANQATVLMGLGKGWPLSKMDIGVPLLAIGCYSQVNPITLTAALLLLVAHYAIIGPGLQAKATREAQKRAAAGIMKNPTVDGITVIDLDPIPYDPKFEKQLGQVMLLILCVTQVLMMRTTWALCEALTLATGPISTLWEGNPGRFWNTTIAVSMANIFRGSYLAGAGLLFSIMKNTTNTRRGTGNIGETLGEKWKSRLNALGKSEFQIYKKSGIQEVDRTLAKEGIKRGETDHHAVSRGSAKLRWFVERNMVTPEGKVVDLGCGRGGWSYYCGGLKNVREVKGLTKGGPGHEEPIPMSTYGWNLVRLQSGVDVFFIPPEKCDTLLCDIGESSPNPTVEAGRTLRVLNLVENWLNNNTQFCIKVLNPYMPSVIEKMEALQRKYGGALVRNPLSRNSTHEMYWVSNASGNIVSSVNMISRMLINRFTMRHKKATYEPDVDLGSGTRNIGIESETPNLDIIGKRIEKIKEEHETSWHYDQDHPYKTWAYHGSYETKQTGSASSMVNGVVRLLTKPWDVLPTVTQMAMTDTTPFGQQRVFKEKVDTRTQEPKEGTKKLMKITAEWLWKELGKKKTPRMCTREEFTRKVRSNAALGAIFTDENKWKSAREAVEDSRFWELVDKERNLHLEGKCETCVYNMMGKREKKLGEFGKAKGSRAIWYMWLGARFLEFEALGFLNEDHWFSRENSLSGVEGEGLHKLGYILRDVSKKEGGAMYADDTAGWDTRITLEDLKNEEMVTNHMEGEHKKLAEAIFKLTYQNKVVRVQRPTPRGTVMDIISRRDQRGSGQVGTYGLNTFTNMEAQLIRQMEGEGVFKNIQHLTVTEEAAVQNWLARVGRERLSRMAISGDDCVVKPLDDRFANALTALNDMGKIRKDIQQWEPSKGWNDWTQVPFCSHHFHELIMKDGRVLVVPCRNQDELIGRARISQGAGWSLRETACLGKSYAQMWSLMYFHRRDLRLAANAICSAVPSHWVPTSRTTWSIHAKHEWMTTEDMLTVWNRVWIRENPWMEDKTPVESWEEIPYLGKREDQWCGSLIGLTSRATWAKNIQAAINQVRSLIGNEEYTDYMPSMKRFRREEEEAGVLW

>DENV2_AHG23169_Cambodia

MNNQRKKAKNTPFNMLKRERNRVSTVQQLTKRFSLGMLQGRGPLKLFMALVAFLRFLTIPPTAGILKRWGTIKKSKAINVLRGFRKEIGRMLNILNRRRRSAGMIIMLIPTVMAFHLTTRNGEPHMIVGIQEKGKSLLFKTEDGVNMCTLMAMDLGELCEDTITYKCPLLRQNEPEDIDCWCNSTSTWVTYGTCTTTGEHRREKRSVALVPHVGMGLETRTETWMSSEGAWKHAQRIETWILRHPGFTIMAAILAYTIGTTHFQRVLIFILLTAVAPSMTMRCIGISNRDFVEGVSGGSWVDIVLEHGSCVTTMAKNKPTLDFELIKTEAKQPATLRKYCIEAKLTNTTTESRCPTQGEPSLKEEQDKRFVCKHSMVDRGWGNGCGLFGKGGIVTCAMFTCKKNMEGKIVQPENLEYTIVVTPHSGEEHAVGNDTGKHGMEIKVTPQSSITEAELTGYGTVTMECSPRTGLDFNEMVLLQMENKAWLVHRQWFLDLPLPWLPGADKQESNWIQKETLVTFKNPHAKKQDVVVLGSQEGAMHTALTGATEIQMSSGNLLFTGHLKCRLRMDKLQLKGMSYSMCTGKFKVVKEIAETQHGTIVIRVQYEGDGSPCKIPFEIMDLEKRYVLGRLITVNPIVTEKDSPVNIEAEPPFGDSYIIIGVEPGQLKLNWFKKGSSIGQMFETTMRGAKRMAILGDTAWDFGSLGGVFTSIGKALHQVFGAIYGVAFSGVSWTMKILIGVIITWIGMNSRSTSLSVSLVLVGIVTLYLGVMVQADSGCVVSWKNKELKCGSGIFITDNVHTWTEQYKFQPESPSKLASAIQKAQEEGICGIRSVTRLENLMWKQITPELNHILAENEVKLTIMTGDIKGIMQAGKRSLRPQPTELKYSWKTWGKAKMLFTESYNQTFLIDGPETAECPNTNRAWNSLEVEDYGFGVFTTNIWLKLKEKQDAFCDSKLMSAAIKDNRAVHADMGYWIESALNDTWKIEKASFIEVKNCHWPKSHTLWSNGVLESEMIIPKNLAGPVSQHNYRPGYHTQIAGPWHLGKLEMDFDFCDGTTVIVTEDCGNRGPSLRTTTASGKLITEWCCRSCTLPPLRYRGEDGCWYGMEIRPLKEKEENLVNSLVTAGHGQVDNFSLGVLGMALFLEEMLRTRVGTKHAILLVAVSFVTLITGNMSFKDLGRVVVMVGATMADDIGMGVTYLALLAAFKVRPTFAAGLLLRKLTSKELMMTTIGIVLLSQSTIPETVLELTDALALGMMVLKIVRNMEKYQLAVTIMAILCVPNAVILQNAWKVSCTILAVVSVSPLLLTSSQQKTDWIPLALTIKGLNPTAIFLTTLSRTNKKRSWPLNEAIMAVGMVSILASSLLKNDIPMTGPLVAGGLLTVCYVLTGRSADLELERATDVKWEDQAEISGSSPILSITISEDGSMSIKNEEEEQTLTILIRTGLLVISGLFPISIPITAAAWYLWEVKKQRAGVLWDVPSPPPMGKAELEDGAYRIKQKGILGYSQIGAGVYKEGTFHTMWHVTRGAVLMHKGKRIEPSWADVKKDLISYGGGWKLEGEWKEGEEVQVLALEPGKNPRAVQTKPGLFKTNTGTIGAVSLDFSPGTSGSPIIDKKGKVVGLYGNGVVTRSGAYVSAIAQTEKSIEDNPEIEDDIFRKRRLTIMDLHPGAGKTKRYLPAIVREAIKRGLRTLILAPTRVVAAEMEEALRGLPIRYQTPAIRAEHTGREIVDLMCHATFTMRLLSPVRVPNYNLIIMDEAHFTDPASIAARGYISTRVEMGEAAGIFMTATPPGSRDPFPQSNAPIIDEEREIPERSWNSGHEWVTDFKGKTVWFVPSIKAGNDIAACLRKNGKKVIQLSRKTFDSEYVKTRTNDWDFVVTTDISEMGANFKAERVIDPRRCMKPVILTDGEERVILAGPMPVTHSSAAQRRGRIGRNPKNENDQYIYMGEPLENDEDCAHWKEAKMLLDNINTPEGIIPSMFEPEREKVDAIDGEYRLRGEARKTFVDLMRRGDLPVWLAYKVAAEGINYADRRWCFDGIKNNQILEENVEVEIWTKEGERKKLKPRWLDARIYSDPLALKEFKEFAAGRKSLTLNLITEMGRLPTFMTQKTRDALDNLAVLHTAEAGGRAYNHALSELPETLETLLLLTLLATVTGGIFLFLMSGRGIGKMTLGMCCIITASILLWYAQIQPHWIAASIILEFFLIVLLIPEPEKQRTPQDNQLTYVVIAILTVVAATMANEMGFLEKTKKDLGLGSIATQQPESNILDIDLRPASAWTLYAVATTFVTPMLRHSIENSSVNVSLTAIANQATVLMGLGKGWPLSKMDIGVPLLAIGCYSQVNPITLTAALLLLVAHYAIIGPGLQAKATREAQKRAAAGIMKNPTVDGITVIDLDPIPYDPKFEKQLGQVMLLILCVTQVLMMRTTWALCEALTLATGPISTLWEGNPGRFWNTTIAVSMANIFRGSYLAGAGLLFSIMKNTTNTRRGTGNIGETLGEKWKSRLNALGKSEFQIYKKSGIQEVDRTLAKEGIKRGETDHHAVSRGSAKLRWFVERNMVTPEGKVVDLGCGRGGWSYYCGGLKNVREVKGLTKGGPGHEEPIPMSTYGWNLVRLQSGVDVFFIPPEKCDTLLCDIGESSPNPTVEAGRTLRVLNLVENWLNNNTQFCIKVLNPYMPSVIEKMEALQRKYGGALVRNPLSRNSTHEMYWVSNASGNIVSSVNMISRMLINRFTMRHKKATYEPDVDLGSGTRNIGIESETPNLDIIGKRIEKIKEEHETSWHYDQDHPYKTWAYHGSYETKQTGSASSMVNGVVRLLTKPWDVLPTVTQMAMTDTTPFGQQRVFKEKVDTRTQEPKEGTKKLMKITAEWLWKELGKKKTPRMCTREEFTRKVRSNAALGAIFTDENKWKSAREAVEDSRFWELVDKERNLHLEGKCETCVYNMMGKREKKLGEFGKAKGSRAIWYMWLGARFLEFEALGFLNEDHWFSRENSLSGVEGEGLHKLGYILRDVSKKEGGAMYADDTAGWDTRITLEDLKNEEMVTNHMEGEHKKLAEAIFKLTYQNKVVRVQRPTPRGTVMDIISRRDQRGSGQVGTYGLNTFTNMEAQLIRQMEGEGVFKNIQHLTVTEEAAVQNWLARVGRERLSRMAISGDDCVVKPLDDRFANALTALNDMGKIRKDIQQWEPSKGWNDWTQVPFCSHHFHELIMKDGRVLVVPCRNQDELIGRARISQGAGWSLRETACLGKSYAQMWSLMYFHRRDLRLAANAICSAVPSHWVPTSRTTWSIHAKHEWMTTEDMLTVWNRVWIRENPWMEDKTPVESWEEIPYLGKREDQWCGSLIGLTSRATWAKNIQAAINQVRSLIGNEEYTDYMPSMKRFRREEEEAGVLW

>DENV2_AHG23138_Nicaragua

MNNQRKKARSTPFNMLKRERNRVSTVQQLTKRFSLGMLQGRGPLKLFMALVAFLRFLTIPPTAGILKRWGTIKKSKAINVLRGFRKEIGRMLNILNRRRRTAGVIVMLIPTAMAFHLTTRNGEPHMIVGRQEKGKSLLFKTEDGVNMCTLMAIDLGELCEDTITYKCPLLRQNEPEDIDCWCNSTSTWVTYGTCTTTGEHRREKRSVALVPHVGMGLETRTETWMSSEGAWKHVQRIETWILRHPGFTIMAAILAYTIGTTHFQRALIFILLTTVAPSMTMRCIGISNRDFVEGVSGGSWVDIVLEHGSCVTTMAKNKPTLDFELIKTEAKQPATLRKYCIEAKLTNTTTESRCPTQGEPSLNEEQDKRFICKHSMVDRGWGNGCGLFGKGGIVTCAMFTCKKNMEGKVVQPENLEYTIVITPHSGEEHAVGNDTGKHGKEIKITPQSSITEAELTGYGTVTMECSPRTGLDFNEMVLLQMEDKAWLVHRQWFLDLPLPWLPGADTQGSNWIQKETLVTFKNPHAKKQDVVVLGSQEGAMHTALTGATEIQMSSGNLLFTGHLKCRLRMDKLQLKGMSYSMCTGKFKIVKEIAETQHGTIVIRVQYEGDGSPCKIPFEITDLEKRHVLGRLITVNPIVTEKDSPVNIEAEPPFGDSYIIIGVEPGQLKLNWFKKGSSIGQMFETTMRGAKRMAILGDTAWDFGSLGGVFTSIGKALHQVFGAIYGAAFSGVSWTMKILIGVIITWIGMNSRSTSLSVSLVLVGVVTLYLGAVVQADSGCVVSWKNKELKCGSGIFITDNVHTWTEQYKFQPESPSKLASAIQKAHEEGICGIRSVTRLENLMWKQITPELNHILSENEVKLTIMTGDIKGIMQAGKRSLRPQPTELKYSWKTWGKAKMLSTESHNQTFLIDGPETAECPNTNRAWNSLEVEDYGFGVFTTNIWLKLREKQDVFCDSKLMSAAIKDNRAVHADMGYWIESALNDTWKMEKASFIEVKSCHWPKSHTLWSNGVLESEMIIPKNFAGPVSQHNYRPGYHTQTAGPWHLGKLEMDFDFCEGTTVVVTEDCGNRGPSLRTTTASGKLITEWCCRSCTLPPLRYRGEDGCWYGMEIRPLKEKEENLVNSLVTAGHGQIDNFSLGVLGMALFLEEMLRTRIGTKHAILLVAVSFVTLITGNMSFRDLGRVMVMVGATMTDDIGMGVTYLALLAAFKVRPTFAAGLLLRKLTSKELMMATIGIALLSQSTIPETILELTDALALGMMVLKIVRNMEKYQLAVTIMAISCVPNAVILQNAWKVSCTILAAVSVSPLLLTSSQQKADWIPLALTIKGLNPTAIFLTTLSRTSKKRSWPLNEAIMAVGMVSILASSLLKNDIPMTGPLVAGGLLTVCYVLTGRSADLELERAADVKWEDQAEISGSSPILSITISEDGSMSIKNEEEEQTLTILIRTGLLVISGVFPVSIPITAAAWYLWEVKKQRAGVLWDVPSPPPVEKAELEDGAYRIKQRGILGYSQIGAGVYKEGTFHTMWHVTRGAVLMHRGKRIEPSWADVKKDLISYGGGWKLEGEWKEGEEVQVLALEPGKNPRAVQTKPGIFKTNTGTIGAVSLDFSPGTSGSPIVDRKGKVVGLYGNGVVTRSGAYVSAIAQTEKSIEDNPEIEDDIFRKKRLTIMDLHPGAGKTKRYLPAIVREAIKRGLRTLILAPTRVVAAEMEEALRGLPIRYQTPAIKTEHTGREIVDLMCHATFTMRLLSPVRVPNYNLIIMDEAHFTDPASIAARGYISTRVEMGEAAGIFMTATPPGSRDPFPQSNAPIMDEEREIPERSWNSGHEWVTDFKGKTVWFVPSIKAGNDIAACLRKNGKKVIQLSRKTFDSEYVKTRANDWDFVVTTDISEMGANFKAERVIDPRRCMKPVILTDGEERVILAGPMPVTHSSAAQRRGRIGRNPKNENDQYIYMGEPLENDEDCAHWKEAKMLLDNINTPEGIIPSMFEPEREKVDAIDGEYRLRGEARKTFVDLMRRGDLPVWLAYKVAAEGINYADRKWCFDGIKNNQILEENMEVEIWTKEGERKKLKPRWLDARIYSDPLALKEFKEFAAGRKSLTLNLITEMGRLPTFMTQKARNALDNLAVLHTAEAGGRAYNHALSELPETLETLLLLTLLATVTGGIFLFLMSGKGIGKMTLGMCCIITASILLWYAQIQPHWIAASIILEFFLIVLLIPEPEKQRTPQDNQLTYVVIAILTVVAATMANEMGFLEKTKKDLGLGSITTQESESNILDIDLRPASAWTLYAVATTFVTPMLRHSIENSSVNVSLTAIANQATVLMGLGKGWPLSKMDIGVPLLAIGCYSQVNPITLTAALLLLVAHYAIIGPGLQAKATREAQKRAAAGIMKNPTVDGITVIDLEPIPYDPKFEKQLGQVMLLILCVTQVLMMRTTWALCEALTLATGPISTLWEGNPGRFWNTTIAVSMANIFRGSYLAGAGLLFSIMKNTTSTRRGTGNIGETLGEKWKSRLNALGKSEFQIYKKSGIQEVDRTLAKEGIKRGETDHHAVSRGSAKLRWFVERNMVTPEGKVVDLGCGRGGWSYYCGGLKNVREVKGLTKGGPGHEEPIPMSTYGWNLVRLQSGVDVFFTPPEKCDTLLCDIGESSPNPTIEAGRTLRVLNLVENWLNNNTQFCIKVLNPYMPSVIEKMEALQRQYGGALVRNPLSRNSTHEMYWVSNATGNIVSSVNMISRMLINRFTMKHKKATYEPDVDLGSGTRNIGIESEIPNLDIIGKRIEKIKQEHEISWHYDQDHPYKTWAYHGSYETKQTGSASSMVNGVVRLLTKPWDVVPMVTQMAMTDTTPFGQQRVFKEKVDTRTQEPKEGTKKLMKITAEWLWKELGKKKTPRMCTREEFTKKVRSNAALGAIFTDENKWKSAREAVEDGRFWELVDRERNLHLEGKCETCVYNMMGKREKKLGEFGKAKGSRAIWYMWLGARFLEFEALGFLNEDHWFSRGNSLSGVEGEGLHRLGYILRDVGKKEGGAMYADDTAGWDTRITLEDLKNEEMVTNHMKGEHKKLAEAIFKLTYQNKVVRVQRPTPRGTVMDIISRRDQRGSGQVGTYGLNTFTNMEAQLIRQMEGEGIFKSIQHLTATEEIAVQNWLARVGRERLSRMAISGDDCVVKPIDDRFASALTALNDMGKVRKDIQQWEPSRGWNDWTQVPFCSHHFHELVMKDGRVLVVPCRNQDELIGRARISQGAGWSLKETACLGKSYAQMWTLMYFHRRDLRLAANAICSAVPSHWVPTSRTTWSIHAKHEWMTTEDMLAVWNRVWIQENPWMEDKTPVESWEEVPYLGKREDQWCGSLIGLTSRATWAKNIQTAINQVRSLIGNEEYTDYMPSMKRFRREEEEAGVLW

>DENV2_AHG23135_Nicaragua

MNNQRKKARSTPFNMLKRERNRVSTVQQLTKRFSLGMLQGRGPLKLFMALVAFLRFLTIPPTVGILKRWGTIKKSKAINVLRGFRKEIGRMLNILNRRRRTAGVIVMLIPTAMAFHLTTRNGEPHMIVGRQEKGKSLLFKTEDGVNMCTLMAIDLGELCEDTITYKCPLLRQNEPEDIDCWCNSTSTWVTYGTCTTTGEHRREKRSVALVPHVGMGLETRTETWMSSEGAWKHVQRIETWILRHPGFTIMAAILAYTIGTTHFQRALIFILLTAVAPSMTMRCIGISNRDFVEGVSGGSWVDIVLEHGSCVTTMAKNKPTLDFELIKTEAKQPATLRKYCIEAKLTNTTTESRCPTQGEPSLNEEQDKRFICKHSMVDRGWGNGCGLFGKGGIVTCAMFTCKKNMEGKVVQPENLEYTIVITPHSGEEHAVGNDTGKHGKEIKITPQSSITEAELTGYGTVTMECSPRTGLDFNEMVLLQMEDKAWLVHRQWFLDLPLPWLPGADTQGSNWIQKETLVTFKNPHAKKQDVVVLGSQEGAMHTALTGATEIQMSSGNLLFTGHLKCRLRMDKLQLKGMSYSMCTGKFKIVKEIAETQHGTIVIRVQYEGDGSPCKIPFEITDLEKRHVLGRLITVNPIVTEKDSPVNIEAEPPFGDSYIIIGVEPGQLKLNWFKKGSSIGQMFETTMRGAKRMAILGDTAWDFGSLGGVFTSIGKALHQVFGAIYGAAFSGVSWTMKILIGVIITWIGMNSRSTSLSVSLVLVGVVTLYLGAVVQADSGCVVSWKNKELKCGSGIFITDNVHTWTEQYKFQPESPSKLASAIQKAHEEGICGIRSVTRLENLMWKQITPELNHILSENEVKLTIMTGDIKGIMQAGKRSLRPQPTELKYSWKTWGKAKMLSTESHNQTFLIDGPETAECPNTNRAWNSLEVEDYGFGVFTTNIWLKLREKQDVFCDSKLMSAAIKDNRAVHADMGYWIESALNDTWKMEKASFIEVKSCHWPKSHTLWSNGVLESEMIIPKNFAGPVSQHNYRPGYHTQTAGPWHLGKLEMDFDFCEGTTVVVTEDCGNRGPSLRTTTASGKLITEWCCRSCTLPPLRYRGEDGCWYGMEIRPLKEKEENLVNSLVTAGHGQIDNFSLGVLGMALFLEEMLRTRIGTKHAILLVAVSFVTLITGNMSFRDLGRVMVMVGATMTDDIGMGVTYLALLAAFKVRPTFAAGLLLRKLTSKELMMATIGIALLSQSTIPETILELTDALALGMMVLKIVRNMEKYQLAVTIMAISCVPNAVILQNAWKVSCTILAAVSVSPLLLTSSQQKADWIPLALTIKGLNPTAIFLTTLSRTSKKRSWPLNEAIMAVGMVSILASSLLKNDIPMTGPLVAGGLLTVCYVLTGRSADLELERAADVKWEDQAEISGSSPILSITISEDGSMSIKNEEEEQTLTILIRTGLLVISGVFPVSIPITAAAWYLWEVKKQRAGVLWDVPSPPPVEKAELEDGAYRIKQRGILGYSQIGAGVYKEGTFHTMWHVTRGAVLMHRGKRIEPSWADVKKDLISYGGGWKLEGEWKEGEEVQVLALEPGKNPRAVQTKPGIFKTNTGTIGAVSLDFSPGTSGSPIVDRKGKVVGLYGNGVVTRSGAYVSAIAQTEKSIEDNPEIEDDIFRKKRLTIMDLHPGAGKTKRYLPAIVREAIKRGLRTLILAPTRVVAAEMEEALRGLPIRYQTPAIKTEHTGREIVDLMCHATFTMRLLSPVRVPNYNLIIMDEAHFTDPASIAARGYISTRVEMGEAAGIFMTATSPGSRDPFPQSNAPIMDEEREIPERSWNSGHEWVTDFKGKTVWFVPSIKAGNDIAACLRKNGKKVIQLSRKTFDSEYVKTRANDWDFVVTTDISEMGANFKAERVIDPRRCMKPVILTDGEERVILAGPMPVTHSSAAQRRGRIGRNPKNENDQYIYMGEPLENDEDCAHWKEAKMLLDNINTPEGIIPSMFEPEREKVDAIDGEYRLRGEARKTFVDLMRRGDLPVWLAYKVAAEGINYADRKWCFDGIKNNQILEENMEVEIWTKEGERKKLKPRWLDARIYSDPLALKEFKEFAAGRKSLTLNLITEMGRLPTFMTQKARNALDNLAVLHTAEAGGRAYNHALSELPETLETLLLLTLLATVTGGIFLFLMSGKGIGKMTLGMCCIITASILLWYAQIQPHWIAASIILEFFLIVLLIPEPEKQRTPQDNQLTYVVIAILTVVAATMANEMGFLEKTKKDLGLGSITTQESESNILDIDLRPASAWTLYAVATTFVTPMLRHSIENSSVNVSLTAIANQATVLMGLGKGWPLSKMDIGVPLLAIGCYSQVNPITLTAALLLLVAHYAIIGPGLQAKATREAQKRAAAGIMKNPTVDGITVIDLEPIPYDPKFEKQLGQVMLLILCVTQVLMMRTTWALCEALTLATGPISTLWEGNPGRFWNTTIAVSMANIFRGSYLAGAGLLFSIMKNTTSTRRGTGNIGETLGEKWKSRLNALGKSEFQIYKKSGIQEVDRTLAKEGIKRGETDHHAVSRGSAKLRWFVERNMVTPEGKVVDLGCGRGGWSYYCGGLKNVREVKGLTKGGPGHEEPIPMSTYGWNLVRLQSGVDVFFTPPEKCDTLLCDIGESSPNPTIEAGRTLRVLNLVENWLNNNTQFCIKVLNPYMPSVIEKMEALQRQYGGALVRNPLSRNSTHEMYWVSNATGNIVSSVNMISRMLINRFTMKHKKATYEPDVDLGSGTRNIGIESEIPNLDIIGKRIEKIKQEHEISWHYDQDHPYKTWAYHGSYETKQTGSASSMVNGVVRLLTKPWDVVPMVTQMAMTDTTPFGQQRVFKEKVDTRTQEPKEGTKKLMKITAEWLWKELGKKKTPRMCTREEFTKKVRSNAALGAIFTDENKWKSAREAVEDGRFWELVDRERNLHLEGKCETCVYNMMGKREKKLGEFGKAKGSRAIWYMWLGARFLEFEALGFLNEDHWFSRGNSLSGVEGEGLHRLGYILRDVGKKEGGAMYADDTAGWDTRITLEDLKNEEMVTNHMKGEHKKLAEAIFKLTYQNKVVRVQRPTPRGTVMDIISRRDQRGSGQVGTYGLNTFTNMEAQLIRQMEGEGIFKSIQHLTATEEIAVQNWLARVGRERLSRMAISGDDCVVKPIDDRFASALTALNDMGKVRKDIQQWEPSRGWNDWTQVPFCSHHFHELVMKDGRVLVVPCRNQDELIGRARISQGAGWSLKETACLGKSYAQMWTLMYFHRRDLRLAANAICSAVPSHWVPTSRTTWSIHAKHEWMTTEDMLAVWNRVWIQENPWMEDKTPVESWEEVPYLGKREDQWCGSLIGLTSRATWAKNIQTAINQVRSLIGNEEYTDYMPSMKRFRREEEEAGVLW

>DENV2_AHG23133_Puerto_Rico

MNNQRKKARSTPFNMLKRERNRVSTVQQLTKRFSLGMLQGRGPLKLFMALVAFLRFLTIPPTAGILKRWGTIKKSKAINVLRGFRKEIGRMLNILNRRRRTAGVIIMLIPTAMAFHLTTRNGEPHMIVGRQEKGKSLLFKTEDGVNMCTLMAIDLGELCEDTITYKCPLLRQNEPEDIDCWCNSTSTWVTYGTCTTTGEHRREKRSVALVPHVGMGLETRTETWMSSEGAWKHVQRIETWILRHPGFTIMAAILAYTIGTTHFQRALIFILLTAVAPSMTMRCIGISNRDFVEGVSGGSWVDIVLEHGSCVTTMAKNKPTLDFELIKTEAKQPATLRKYCIEAKLTNTTTESRCPTQGEPSLNEEQDKRFICKHSMVDRGWGNGCGLFGKGGIVTCAMFTCKKNMEGKVVLPENLEYTIVITPHSGEEHAVGNDTGKHGKEIKITPQSSITEAELTGYGTVTMECSPRTGLDFNEMVLLQMEDKAWLVHRQWFLDLPLPWLPGADTQGSNWIQKETLVTFKNPHAKKQDVVVLGSQEGAMHTALTGATEIQMSSGNLLFTGHLKCRLRMDKLQLKGMSYSMCTGKFKIVKEIAETQHGTIVIRVQYEGDGSPCKIPFEIMDLEKRHVLGRLITVNPIVTEKDSPVNIEAEPPFGDSYIIIGVEPGQLKLNWFKKGSSIGQMFETTMRGAKRMAILGDTAWDFGSLGGVFTSIGKALHQVFGAIYGAAFSGVSWTMKILIGVIITWIGMNSRSTSLSVSLVLVGVVTLYLGVMVQADTGCVVSWKNKELKCGSGIFITDNVHTWTEQYKFQPESPSKLASAIQKAHEEGICGIRSVTRLENLMWKQITPELNHILSENGVKLTIMTGDIKGIMQAGKRSLRPQPTELKYSWKTWGKAKMLSTESHNQTFLIDGPETAECPNTNRAWNSLEVEDYGFGVFSTNIWLKLREKQDVLCDSKLMSAAIKDNRAVHADMGYWIESALNDTWKMEKASFIEIKSCHWPKSHTLWSNGVLESEMIIPKNFAGPVSQHNYRPGYHTQTAGPWHLGKLEMDFDFCEGTTVVVTEDCGNRGPSLRTTTASGKLITEWCCRSCTLPPLRYRGEDGCWYGMEIRPLKEKEENLVNSLVTAGHGQIDNFSLGVLGMALFLEEMLRTRVGTKYAILLVAVSFVTLITGNMSFRDLGRVMVMVGATMTDDIGMGVTYLALLAAFKVRPTFAAGLLLRKLTSKELMMATIGIALLSQSTIPETILELTDALALGMMVLKIVRNMEKYQLAVTIMAISCVPNAVILQNAWKVSCTILAAVSVSPLLLTSSQQKADWIPLALTIKGLNPTAIFLTTLSRTSKKRSWPLNEAVMAVGMVSILASSLLKNDIPMTGPLVAGGLLTVCYVLTGRSADLELERAADVKWEDQAEISGSSPILSITISEDGSMSIKNEEEEHTLTILIRTGLLVISGVFPVSIPITAAAWYLWEVKKQRAGVLWDVPSPPPVGKAELEDGAYRIKQRGIFGYSQIGAGVYKEGTFHTMWHVTRGAVLMHRGKRIEPSWADVKKDLISYGGGWKLEGEWKEGEEVQVLALEPGKNPRAVQTKPGLFKTNTGTIGAVSLDFSPGTSGSPIVDRKGKVVGLYGNGVVTRSGAYVSAIAQTEKSIEDNPEIEDDIFRKKRLTIMDLHPGAGKTKRYLPAIVREAIKRGLRTLILAPTRVVAAEMEEALRGLPIRYQTPAIRAEHTGREIVDLMCHATFTMRLLSPVRVPNYNLIIMDEAHFTDPASIAARGYISTRVEMGEAAGIFMTATPPGSRDPFPQSNAPIMDEEREIPERSWNSGHEWVTDFKGKTVWFVPSIKAGNDIAACLRKNGKKVIQLSRKTFDSEYVKTRANDWDFVVTTDISEMGANFRAERVIDPRRCMKPVILTDGEERVILAGPMPVTHSSAAQRRGRIGRNPKNENDQYIYMGEPLENDEDCAHWKEAKMLLDNINTPEGIIPSMFEPEREKVDAIDGEYRLRGEARKTFVDLMRRGDLPVWLAYRVAAEGINYADRRWCFDGIKNNQILEENVEVEIWTKEGERKKLKPRWLDARIYSDPLALKEFKEFAAGRKSLTLNLITEMGRLPTFMTQKARDALDNLAVLHTAEVGGRAYNHALSELPETLETLLLLTLLATVTGGIFLFLMSGKGIGKMTLGMCCIITASILLWYAQIQPHWIAASIILEFFLIVLLIPEPEKQRTPQDNQLTYVVIAILTVVAATMANEMGFLEKTKKDLGLGSITTQESESNILDIDLRPASAWTLYAVATTFVTPMLRHSIENSSVNVSLTAIANQATVLMGLGRGWPLSKMDIGVPLLAIGCYSQVNPITLTAALLLLVAHYAIIGPGLQAKATREAQKRTAAGIMKNPTVDGITVIDLEPIPYDPKFEKQLGQVMLLVLCVTQVLMMRTTWALCEALTLATGPISTLWEGNPGRFWNTTIAVSMANIFRGSYLAGAGLLFSIMKNTTNTRRGTGNIGETLGEKWKSRLNALGKSEFQIYKKSGIQEVDRTLAKEGIKRGETDHHAVSRGSAKLRWFVERNMVTPEGKVVDLGCGRGGWSYYCGGLKNVREVKGLTKGGPGHEEPIPMSTYGWNLVRLQSGVDVFFTPPEKCDTLLCDIGESSPNPTIEAGRTLRVLNLVENWLNNNTQFCVKVLNPYMPSVIEKMETLQRKYGGALVRNPLSRNSTHEMYWVSNATGNIVSSVNMISRMLINRFTMKHKKATYEPDVDLGSGTRNIGIENEIPNLDIIGKRIEKIKQEHETSWHYDQDHPYKTWAYHGSYETKQTGSASSMVNGVVKLLTKPWDVVPMVTQMAMTDTTPFGQQRVFKEKVDTRTQEPKEGTKKLMRITAEWLWKELGKKKTPRMCTREEFTRKVRSNAALGAIFTDENKWKSAREAVEDSRFWELVDRERNLHLEGKCETCVYNMMGKREKKLGEFGKAKGSRAIWYMWLGARFLEFEALGFLNEDHWFSRGNSLSGVEGEGLHKLGYILRDVSKKEGGAMYADDTAGWDTRITLEDLKNEEMVTNHMKGEHKKLAEAIFKLTYQNKVVRVQRPTPRGTVMDIISRKDQRGSGQVGTYGLNTFTNMEAQLIRQMEGEGIFKSIQRLTVTEEIAVQNWLARVGRERLSRMAISGDDCVVKPLDDRFASALTALNDMGKVRKDIQQWEPSRGWNDWTQVPFCSHHFHELVMKDGRVLVVPCRNQDELIGRARISQGAGWSLKETACLGKSYAQMWTLMYFHRRDLRLAANAICSAVPSHWVPTSRTTWSIHAKHEWMTTEDMLAVWNRVWIQENPWMEDKTPVESWEEVPYLGKREDQWCGSLIGLTSRATWAKNIQTAINQVRSLIGNEEYTDYMPSMKRFRKEEEEAGVLW

>DENV2_AGN94892_Brazil

MNNQRKKARSTPFNMLKRERNRVSTVQQLTKRFSLGMLQGRGPLKLFMALVAFLRFLTIPPTAGILKRWGTIKKSKAINVLRGFRKEIGRMLNILNRRRRTAGVIIMLIPTAMAFHLTTRNGEPHMIVGRQEKGKSLLFKTEDGVNMCTLMAIDLGELCEDTITYKCPLLRQNEPEDIDCWCNSTSTWVTYGTCTTTGEHRREKRSVALVPHVGMGLETRTETWMSSEGAWKHVQRIETWILRHPGFAIMAAILAYTIGTTHFQRALIFILLTAVAPSMTMRCIGISNRDFVEGVSGGSWVDIVLEHGSCVTTMAKNKPTLDFELIKTEAKQPATLRKYCIEAKLTNTTTESRCPTQGEPSLNEEQDKRFICKHSMVDRGWGNGCGLFGKGGIVTCAMFTCKKNMEGKVVLPENLEYTIVITPHSGEEHAVGNDTGKHGKEIKITPQSSITEAELTGYGTVTMECSPRTGLDFNEMVLLQMEEKAWLVHRQWFLDLPLPWLPGADTQGSNWIQKETLVTFKNPHAKKQDVVVLGSQEGAMHTALTGATEIQMSSGNLLFTGHLKCRLRMDKLQLKGMSYSMCTGKFKIVKEIAETQHGTIVIRVQYEGDGSPCKIPFEIMDLEKRHVLGRLITVNPIVTEKDSPVNIEAEPPFGDSYIIIGVEPGQLKLNWFKKGSSIGQMFETTMRGAKRMAILGDTAWDFGSLGGVFTSIGKALHQVFGAIYGAAFSGVSWTMKILIGVIITWIGMNSRSTSLSVSLVLVGVVTLYLGAMVQADSGCVVSWKNKELKCGSGIFITDNVHTWTEQYKFQPESPSKLASAIQKAHEEGICGIRSVTRLENLMWKQITPELNHILSENEVKLTIMTGDIKGIMQAGKRSLRPQPTELKYSWKTWGKAKMLSTESHNQTFLIDGPETAECPNTNRAWNSLEVEDYGFGVFTTNIWLKLREKQDVFCDSKLMSAAIKDNRAVHADMGYWIESALNDTWKMEKASFIEVKSCHWPKSHTLWSNGVLESEMIIPKNFAGPVSQHNYRPGYHTQTAGPWHLGKLEMDFDFCEGTTVVVTEDCGNRGPSLRTTTASGKLITEWCCRSCTLPPLRYRGEDGCWYGMEIRPLKEKEENLVNSLVTAGHGQIDNFSLGVLGMALFLEEMLRTRVGTKHAILLVAASFVTLITGNMSFRDLGRVMVMVGATMTDDIGMGVTYLALLAAFKVRPTFAAGLLLRKLTSKELMMATIGIALLSQSTIPETILELTDALALGMMVLRIVRNMEKYQLAVTIMAISCVPNAVILLNAWKVSCTILAAVSVSPLLLTSSQQKTDWIPLALTIKGLNPTAIFLTTLSRTSKKRSWPLNEAVMAVGMVSILASSLLKNDIPMTGPLVAGGLLTVCYVLTGRSADLELERAADVKWEDQAEISGSSPILSITISEDGSMSIKNEEEEQTLTILIRTGLLVISGVFPVSIPITAAAWYLWEVKKQRAGVLWDVPSPPPVEKAELEDGAYRIKQRGILGYSQIGAGVYKEGTFHTMWHVTRGAVLMHRGKRIEPSWADVKKDLISYGGGWKLEGEWKEGEEVQVLALEPGKNPRAVQTKPGLFKTNTGTIGAISLDFSPGTSGSPIVDRKGKVVGLYGNGVVTRSGAYVSAIAQTEKSIEDNPEIEDDIFRKKRLTIMDLHPGAGKTKRYLPAIVREAIKRGLRTLILAPTRVVAAEMEEALRGLPIRYQTPAIRAEHTGREIVDLMCHATFTMRLLSPVRVPNYNLIIMDEAHFTDPASIAARGYISTRVEMGEAAGIFMTATPPGSRDPFPQSNAPIMDEEREIPERSWNSGHEWVTDFKGKTVWFVPSIKAGNDIAACLRKNGKKVIQLSRKTFDSEYVKTRANDWDFVVTTDISEMGANFRAERVIDPRRCMKPVILTDGEERVILAGPMPVTHSSAAQRRGRIGRNPKNENDQYIYMGEPLENDEDCAHWKEAKMLLDNINTPEGIIPSMFEPEREKVDAIDGEYRLRGEARKTFVDLMRRGDLPVWLAYKVAAEGINYADRRWCFDGIKNNQILEENVEVEIWTKEGERKKLKPRWLDARIYSDPLALKEFKEFAAGRKSLTLNLITEMGRLPTFMTQKARDALDNLAVLHTAEAGGRAYKHALSELPETLETLLLLTLLATVTGGIFLFLMSGKGIGKMTLGMCCIITASILLWYAQIQPHWIAASIILEFFLIVLLIPEPEKQRTPQDNQLTYVVIAILTVVAATMANEMGFLEKTKKDLGLGSITTQESESNILDIDLRPASAWTLYAVATTFVTPMLRHSIENSSVNVSLTAIANQATVLMGLGKGWPLSKMDIGVPLLAIGCYSQVNPITLTAALLLLVAHYAIIGPGLQAKATREAQKRAAAGIMKNPTVDGITVIDLEPIPYDPKFEKQLGQVMLLILCVTQVLMMRTTWALCEALTLATGPISTLWEGNPGRFWNTTIAVSMANIFRGSYLAGAGLLFSIMKNTANTRRGTGNIGETLGEKWKSRLNALGKSEFQIYKKSGIQEVDRTLAKEGIKRGETDHHAVSRGSAKLRWFVERNMVTPEGKVVDLGCGRGGWSYYCGGLKNVREVKGLTKGGPGHEEPIPMSTYGWNLVRLQSGVDVFFTPPEKCDTLLCDIGESSPNPTIEAGRTLRVLNLAENWLNNNTQFCIKVLNPYMPSVIEKMETLQRKYGGALVRNPLSRNSTHEMYWVSNATGNIVSSVNMISRMLINRFTMKHKKATYEPDVDLGSGTRNIGIESEIPNLDIIGKRIEKIKQEHETSWHYDQDHPYKTWAYHGSYETKQTGSASSMVNGVVRLLTKPWDVVPMVTQMAMTDTTPFGQQRVFKEKVDTRTQEPKEGTKKLMRITAEWLWKELGKKKTPRMCTREEFTRKVRSNAALGAIFTDENKWKSAREAVEDSRFWELVDRERNLHLEGKCETCVYNMMGKREKKLGEFGKAKGSRAIWYMWLGARFLEFEALGFLNEDHWFSRGNSLSGVEGEGLHRLGYILRDVSKKEGGAMYADDTAGWDTRITLEDLKNEEMVTNHMEGEHKKLAEAIFKLTYQNKVVRVQRPTPRGTVMDIISRKDQRGSGQVGTYGLNTFTNMEAQLIRQMEGEGIFKSIQHLTVTEEIAVQNWLARVGRERLSRMAISGDDCVVKPLDDRFASALTALNDMGKVRKDIQQWEPSRGWNDWTQVPFCSHHFHELVMKDGRVLVVPCRNQDELIGRARISQGAGWSLKETACLGKSYAQMWTLMYFHRRDLRLAANAICSAVPSHWVPTSRTTWSIHAKHEWMTTEDMLAVWNRVWIQENPWMEDKTPVESWEEVPYLGKREDQWCGSLIGLTSRATWAKNIQTAINQVRSLIGNEEYTDYMPSMKRFRREEEEAGVLW

>DENV2_AGN94891_Brazil

MNNQRKKARSTPFNMLKRERNRVSTVQQLTKRFSLGMLQGRGPLKLFMALVAFLRFLTIPPTAGILKRWGTIKKSKAINVLRGFRKEIGRMLNILNRRRRTAGVIIMLIPTAMAFHLTTRNGEPHMIVGRQEKGKSLLFKTEDGVNMCTLMAIDLGELCEDTITYKCPLLRQNEPEDIDCWCNSTSTWVTYGTCTTTGEHRREKRSVALVPHVGMGLETRTETWMSSEGAWKHVQRIETWILRHPGFAIMAAILAYTIGTTHFQRALIFILLTAVAPSMTMRCIGISNRDFVEGVSGGSWVDIVLEHGSCVTTMAKNKPTLDFELIKTEAKQPATLRKYCIEAKLTNTTTESRCPTQGEPSLNEEQDKRFICKHSMVDRGWGNGCGLFGKGGIVTCAMFTCKKNMEGKVVLPENLEYTIMITPHSGEEHAVGNDTGKHGKEIKITPQSSITEAELTGYGTVTMECSPRTGLDFNEMVLLQMEEKAWLVHRQWFLDLPLPWLPGADTQGSNWIQKETLVTFKNPHAKKQDVVVLGSQEGAMHTALTGATEIQMSSGNLLFTGHLKCRLRMDKLQLKGMSYSMCTGKFKIVKEIAETQHGTIVIRVQYEGDGSPCKIPFEIMDLEKRHVLGRLITVNPIVTEKDSPVNIEAEPPFGDSYIIIGVEPGQLKLNWFKKGSSIGQMFETTMRGAKRMAILGDTAWDFGSLGGVFTSIGKALHQVFGAIYGAAFSGVSWTMKILIGVIITWIGMNSRSTSLSVSLVLVGVVTLYLGAMVQADSGCVVSWKNKELKCGSGIFITDNVHTWTEQYKFQPESPSKLASAIQKAHEEGICGIRSVTRLENLMWKQITPELNHILSENEVKLTIMTGDIKGIMQAGKRSLRPQPTELKYSWKTWGKAKMLSTESHNQTFLIDGPETAECPNTNRAWNSLEVEDYGFGVFTTNIWLKLREKQDVFCDSKLMSAAIKDNRAVHADMGYWIESALNDTWKMEKASFIEVKSCHWPKSHTLWSNGVLESEMIIPKNFAGPVSQHNYRPGYHTQTAGPWHLGKLEMDFDFCEGTTVVVTEDCGNRGPSLRTTTASGKLITEWCCRSCTLPPLRYRGEDGCWYGMEIRPLKEKEENLVNSLVTAGHGQIDNFSLGVLGMALFLEEMLRTRVGTKHAILLVAVSFVTLITGNMSFRDLGRVMVMVGVTMTDDIGMGVTYLALLAAFKVRPTFAAGLLLRKLTSKELMMATIGIALLSQSTIPETILELTDALALGMMVLRIVRNMEKYQLAVTIMAISCVPNAVILLNAWKVSCTILAAVSVSPLLLTSSQQKTDWIPLALTIKGLNPTAIFLTTLSRTSKKRSWPLNEAVMAVGMVSILASSLLKNDIPMTGPLVAGGLLTVCYVLTGRSADLELERAADVKWEDQAEISGSSPILSITISEDGSMSIKNEEEEQTLTILIRTGLLVISGVFPVSIPITAAAWYLWEVKKQRAGVLWDVPSPPPVEKAELEDGAYRIKQRGILGYSQIGAGVYKEGTFHTMWHVTRGAVLMHRGKRIEPSWADVKKDLISYGGGWKLEGEWKEGEEVQVLALEPGKNPRAVQTKPGLFKTNTGTIGAISLDFSPGTSGSPIVDRKGKVVGLYGNGVVTRSGAYVSAIAQTEKSIEDNPEIEDDIFRKKRLTIMDLHPGAGKTKRYLPAIVREAIKRGLRTLILAPTRVVAAEMEEALRGLPIRYQTPAIRAEHTGREIVDLMCHATFTMRLLSPVRVPNYNLIIMDEAHFTDPASIAARGYISTRVEMGEAAGIFMTATPPGSRDPFPQSNAPIMDEEREIPERSWNSGHEWVTDFKGKTVWFVPSIKAGNDIAACLRKNGKKVIQLSRKTFDSEYVKTRANDWDFVVTTDISEMGANFRAERVIDPRRCMKPVILTDGEERVILAGPMPVTHSSAAQRRGRIGRNPKNENDQYIYMGEPLENDEDCAHWKEAKMLLDNINTPEGIIPSMFEPEREKVDAIDGEYRLRGEARKTFVDLMRRGDLPVWLAYRVAAEGINYADRRWCFDGIKNNQILEENVEVEIWTKEGERKKLKPRWLDARIYSDPLALKEFKEFAAGRKSLTLNLITEMGRLPTFMTQKARDALDNLAVLHTAEAGGRAYKHALSELPETLETLLLLTLLATVTGGIFLFLMSGKGIGKMTLGMCCIITASILLWYAQIQPHWIAASIILEFFLIVLLIPEPEKQRTPQDNQLTYVVIAILTVVAATMANEMGFLEKTKKDLGLGSITTQESESNILDIDLRPASAWTLYAVATTFVTPMLRHSIENSSVNVSLTAIANQATVLMGLGKGWPLSKMDIGVPLLAIGCYSQVNPITLTAALLLLVAHYAIIGPGLQAKATREAQKRAAAGIMKNPTVDGITVIDLEPIPYDPKFEKQLGQVMLLILCVTQVLMMRTTWALCEALTLATGPISTLWEGNPGRFWNTTIAVSMANIFRGSYLAGAGLLFSIMKNTTNTRRGTGNIGETLGEKWKSRLNALGKSEFQIYKKSGIQEVDRTLAKEGIKRGETDHHAVSRGSAKLRWFVERNMVTPEGKVVDLGCGRGGWSYYCGGLKNVREVKGLTKGGPGHEEPIPMSTYGWNLVRLQSGVDVFFTPPEKCDTLLCDIGESSPNPTIEAGRTLRVLNLAENWLNNNTQFCIKVLNPYMPSVIEKMETLQRKYGGALVRNPLSRNSTHEMYWVSNATGNIVSSVNMISRMLINRFTMKHKKATYEPDVDLGSGTRNIGIESEIPNLDIIGKRIEKIKQEHETSWHYDQDHPYKTWAYHGSYETKQTGSASSMVNGVVRLLTKPWDVVPMVTQMAMTDTTPFGQQRVFKEKVDTRTQEPKEGTKKLMRITAEWLWKELGKKKTPRMCTREEFTRKVRSNAALGAIFTDENKWKSAREAVEDSRFWELVDRERNLHLEGKCETCVYNMMGKREKKLGEFGKAKGSRAIWYMWLGARFLEFEALGFLNEDHWFSRGNSLSGVEGEGLHRLGYILRDVSKKEGGAMYADDTAGWDTRITLEDLKNEEMVTNHMKGEHKKLAEAIFKLTYQNKVVRVQRPTPRGTVMDIISRKDQRGSGQVGTYGLNTFTNMEAQLIRQMEGEGIFKSIQHLTVTEEIAVQNWLARVGRERLSRMAISGDDCVVKPLDDRFASALTALNDMGKVRKDIQQWEPSRGWNDWTQVPFCSHHFHELVMKDGRVLVVPCRNQDELIGRARISQGAGWSLKETACLGKSYAQMWTLMYFHRRDLRLAANAICSAVPSHWVPTSRTTWSIHAKHEWMTTEDMLAVWNRVWIQENPWMEDKTPVESWEEVPYLGKREDQWCGSLIGLTSRATWAKNIQTAINQVRSLIGNEEYTDYMPSMKRFRREEEEAGVLW

>DENV2_AGN94890_Brazil

MNNQRKKARSTPFNMLKRERNRVSTVQQLTKRFSLGMLQGRGPLKLFMALVAFLRFLTIPPTAGILKRWGTIKKSKAINVLRGFRKEIGRMLNILNRRRRTAGVIIMLIPTAMAFHLTTRNGEPHMIVGRQEKGKSLLFKTEDGVNMCTLMAIDLGELCEDTITYKCPLLRQNEPEDIDCWCNSTSTWVTYGTCTTTGEHRREKRSVALVPHVGMGLETRTETWMSSEGAWKHVQRIETWILRHPGFAIMAAILAYTIGTTHFQRALIFILLTAVAPSMTMRCIGISNRDFVEGVSGGSWVDIVLEHGSCVTTMAKNKPTLDFELIKTEAKQPATLRKYCIEAKLTNTTTESRCPTQGEPSLNEEQDKRFICKHSMVDRGWGNGCGLFGKGGIVTCAMFTCKKNMEGKVVLPENLEYTIVITPHSGEEHAVGNDTGKHGEEIKITPQSSITEAELTGYGTVTMECSPRTGLDFNEMVLLQMEEKAWLVHRQWFLDLPLPWLPGADTQGSNWIQKETLVTFKNPHAKKQDVVVLGSQEGAMHTALTGATEIQMSSGNLLFTGHLKCRLRMDKLQLKGMSYSMCTGKFKIVKEIAETQHGTIVIRVQYEGDGSPCKIPFEIMDLEKRHVLGRLITVNPIVTEKDSPVNIEAEPPFGDSYIIIGVEPGQLKLNWFKKGSSIGQMFETTMRGAKRMAILGDTAWDFGSLGGVFTSIGKALHQVFGAIYGAAFSGVSWTMKILIGVIITWIGMNSRSTSLSVSLVLVGVVTLYLGAMVQADSGCVVSWKNKELKCGSGIFITDNVHTWTEQYKFQPESPSKLASAIQKAHEEGICGIRSVTRLENLMWKQITPELNHILSENEVKLTIMTGDIKGIMQAGKRSLRPQPTELKYSWKTWGKAKMLSTESHNQTFLIDGPETAECPNTNRAWNSLEVEDYGFGVFTTNIWLKLREKQDVFCDSKLMSAAIKDNRAVHADMGYWIESALNDTWKMEKASFIEVKSCHWPKSHTLWSNGVLESEMIIPKNFAGPVSQHNYRPGYHTQTAGPWHLGKLEMDFDFCEGTTVVVTEDCGNRGPSLRTTTASGKLITEWCCRSCTLPPLRYRGEDGCWYGMEIRPLKEKEENLVNSLVTAGHGQIDNFSLGVLGMALFLEEMLRTRVGTKHAILLVAVSFVTLITGNMSFRDLGRVMVMVGVTMTDDIGMGVTYLALLAAFKVRPTFAAGLLLRKLTSKELMMATIGITLLSQSTTPETILELTDALALGMMVLKIVRNMEKYQLAVTIMTILCVPNAVILLNAWKVSCTILAVVSVSPLLLTSSQQKTDWIPLALTIKGLNPTAIFLTTLSRTSKKRSWPLNEAVMAVGMVSILASSLLKNDIPMTGPLVAGGLLTVCYVLTGRSADLELERAADVKWEDQAEISGSSPILSITISEDGSMSIKNEEEEQTLTILIRTGLLVISGVFPVSIPITAAAWYLWEVKKQRAGVLWDVPSPPPVEKAELEDGAYRIKQRGILGYSQIGAGVYKEGTFHTMWHVTRGAVLMHRGKRIEPSWADVKKDLISYGGGWKLEGEWKEGEEVQVLALEPGKNPRAVQTKPGLFKTNTGTIGAVSLDFSPGTSGSPIVDRKGKVVGLYGNGVVTRSGAYVSAIAQTEKSIEDNPEIEDDIFRKKRLTIMDLHPGAGKTKRYLPAIVREAIKRGLRTLILAPTRVVAAEMEEALRGLPIRYQTPAIRAEHTGREIVDLMCHATFTMRLLSPVRVPNYNLIIMDEAHFTDPASIAARGYISTRVEMGEAAGIFMTATPPGSRDPFPQSNAPIMDEEREIPERSWNSGHEWVTDFKGKTVWFVPSIKAGNDIAACLRKNGKKVIQLSRKTFDSEYVKTRANDWDFVVTTDISEMGANFRAERVIDPRRCMKPVILTDGEERVILAGPMPVTHSSAAQRRGRVGRNPKNENDQYIYMGEPLENDEDCAHWKEAKMLLDNINTPEGIIPSMFEPEREKVDAIDGEYRLRGEARKTFVDLMRRGDLPVWLAYRVAAEGINYADRRWCFDGIKNNQILEENVEVEIWTKEGERKKLKPRWLDARIYSDPLALKEFKEFAAGRKSLTLNLITEMGRLPTFMTQKARNALDNLAVLHTAEAGGRAYNHALSELPETLETLLLLTLLATVTGGIFLFLMSGKGIGKMTLGMCCIITASVLLWYAQIQPHWIAASIILEFFLIVLLIPEPEKQRTPQDNQLTYVVIAILTVVAATMANEMGFLEKTKKDLGLGSITTQESESNILDIDLRPASAWTLYAVATTFVTPMLRHSIENSSVNVSLTAIANQATVLMGLGKGWPLSKMDIGVPLLAIGCYSQVNPITLTAALLLLVAHYAIIGPGLQAKATREAQKRAAAGIMKNPTVDGITVVDLEPIPYDPKFEKQLGQVMLLILCVTQVLMMRTTWALCEALTLATGPISTLWEGNPGRFWNTTIAVSMANIFRGSYLAGAGLLFSIMKNTTNTRRGTGNIGETLGEKWKSRLNALGKSEFQIYKRSGIQEVDRTLAKEGIKRGETDHHAVSRGSAKLRWFVERNMVTPEGKVVDLGCGRGGWSYYCGGLKNVREVKGLTKGGPGHEEPIPMSTYGWNLVRLQSGVDVFFTPPEKCDTLLCDIGESSPNPTIEAGRTLRVLNLVENWLNNNTQFCIKVLNPYMPSVIEKMETLQRKYGGALVRNPLSRNSTHEMYWVSNATGNIVSSVNMISRMLINRFTMKHKKATYEPDVDLGSGTRNIGIESEIPNLDIIGKRIEKIKQEHETSWHYDQDHPYKTWAYHGSYETKQTGSASSMVNGVVRLLTKPWDVVPMVTQMAMTDTTPFGQQRVFKEKVDTRTQEPKEGTKKLMRITAEWLWKELGKKKTPRMCTREEFTRKVRSNAALGAIFTDENKWKSAREAVEDSRFWELVDRERNLHLEGKCETCVYNMMGKREKKLGEFGKAKGSRAIWYMWLGARFLEFEALGFLNEDHWFSRGNSLSGVEGEGLHRLGYILRDVSKKEGGAMYADDTAGWDTRITLEDLKNEEMVTNHMKGEHKKLAEAVFKLTYQNKVVRVQRPTPRGTVMDIISRRDQRGSGQVGTYGLNTFTNMEAQLIRQMEGEGIFKSIQHLTVTEEIAVQNWLARVGRERLSRMAISGDDCVVKPLDDRFASALTALNDMGKVRKDIQQWEPSRGWNDWTQVPFCSHHFHELVMKDGRVLVVPCRNQDELIGRARISQGAGWSLKETACLGKSYAQMWTLMYFHRRDLRLAANAICSAVPSHWVPTSRTTWSIHAKHEWMTTEDMLAVWNRVWIQENPWMEDKTPVESWEEVPYLGKREDQWCGSLIGLTSRATWAKNIQTAINQVRSLIGNEEYTDYMPSMKRFRREEEEAGVLW

>DENV2_AGN94889_Brazil

MNNQRKKARSTPFNMLKRERNRVSTVQQLTKRFSLGMLQGRGPLKLFMALVAFLRFLTIPPTAGILKRWGTIKKSKAINVLRGFRKEIGRMLNILNKRRRTAGVIIMLIPTAMAFHLTTRNGEPHMIVGKQEKGKSLLFKTEDGVNMCTLMAIDLGELCEDTITYKCPLLRQNEPEDIDCWCNSTSTWVTYGTCTTTGEHRREKRSVALVPHVGMGLETRTETWMSSEGAWKHVQRIETWILRHPGFAIMAAILAYTIGTTHFQRALIFILLTAVAPSMTMRCIGISNRDFVEGVSGGSWVDIVLEHGSCVTTMAKNKPTLDFELIKTEAKQPATLRKYCIEAKLTNTTTESRCPTQGEPSLNEEQDKRFICKHSMVDRGWGNGCGLFGKGGIVTCAMFTCKKNMEGKVVLPENLEYTIVITPHSGEEHAVGNDTGKHGKEIKITPQSSITEAELTGYGTVTMECSPRTGLDFNEMVLLQMEEKAWLVHRQWFLDLPLPWLPGADTQGSNWIQKETLVTFKNPHAKKQDVVVLGSQEGAMHTALTGATEIQMSSGNLLFTGHLKCRLRMDKLQLKGMSYSMCTGKFKIVKEIAETQHGTIVIRVQYEGDGSPCKIPFEIMDLEKRHVLGRLITVNPIVTEKDSPVNIEAEPPFGDSYIIIGVEPGQLKLNWFKKGSSIGQMFETTMRGAKRMAILGDTAWDFGSLGGVFTSIGKALHQVFGAIYGAAFSGVSWTMKILIGVIITWIGMNSRSTSLSVSLVLVGVVTLYLGAMVQADSGCVVSWKNKELKCGSGIFITDNVHTWTEQYKFQPESPSKLASAIQKAHEEGICGIRSVTRLENLMWKQITPELNHILSENEVKLTIMTGDIKGIMQAGKRSLRPQPTELKYSWKTWGKAKMLSTESHNQTFLIDGPETAECPNTNRAWNSLEVEDYGFGVFTTNIWLKLREKQDVFCDSKLMSAAIKDNRAVHADMGYWIESALNGTWKMEKASFIEVKSCHWPKSHTLWSNGVLESEMIIPKNFAGPVSQHNYRPGYHTQTAGPWHLGKLEMDFDFCEGTTVVVTEDCGNRGPSLRTTTASGKLITEWCCRSCTLPPLRYRGEDGCWYGMEIRPLKEKEENLVNSLVTAGHGQIDNFSLGVLGMALFLEEMLRTRVGTKHAILLVAVSFVTLITGNMSFRDLGRVIVMVGVTMTDDIGMGVTYLALLAAFKVRPTFAAGLLLRKLTSKELMMATIGITLLSQSTIPETILELTDALALGMMVLKIVRNMEKYQLAVTIMAISCVPNAVILLNAWKVSCTILAVVSVSPLLLTSSQQKTDWIPLALTIKGLNPTAIFLTTLSRTSKKRSWPLNEAVMAVGMVSILASSLLKNDIPMTGPLVAGGLLTVCYVLTGRSADLELERAADVKWEDQAEISGSSPILSITISEDGSMSIKNEEEEQTLTILIRTGLLVISGVFPVSIPITAAAWYLWEVKKQRAGVLWDVPSPPPVEKAELEDGAYRIKQRGILGYSQIGAGVYKEGTFHTMWHVTRGAVLMHRGKRIEPSWADVKKDLISYGGGWKLEGEWKEGEEVQVLALEPGKNPRAVQTKPGLFKTNTGTIGAVSLDFSPGTSGSPIVDRKGKVVGLYGNGVVTRSGAYVSAIAQTEKSIEDNPEIEDDIFRKKRLTIMDLHPGAGKTKRYLPAIVREAIKRGLRTLILAPTRVVAAEMEEALRGLPIRYQTPAIRAEHTGREIVDLMCHATFTMRLLSPVRVPNYNLIIMDEAHFTDPASIAARGYISTRVEMGEAAGIFMTATPPGSRDPFPQSNAPIMDEEREIPERSWNSGHEWVTDFKGKTVWFVPSIKAGNDIAACLRKNGKKVIQLSRKTFDSEYVKTRANDWDFVVTTDISEMGANFKAERVIDPRRCMKPVILTDGEERVILAGPMPVTHSSAAQRRGRVGRNPKNENDQYIYMGEPLENDEDCAHWKEAKMLLDNINTPEGIIPSMFEPEREKVDAIDGEYRLRGEARKTFVDLMRRGDLPVWLAYRVAAEGINYADRRWCFDGIKNNQILEENVEVEIWTKEGERKKLKPRWLDARIYSDPLALKEFKEFAAGRKSLTLNLITEMGRLPTFMTQKARDALDNLAVLHTAEAGGRAYNHALSELPETLETLLLLTLLATVTGGIFLFLMSGKGIGKMTLGMCCIITASILLWYAQIQPHWIAASIILEFFLIVLLIPEPEKQRTPQDNQLTYVVIAILTVVAATMANEMGFLEKTKKDLGLGSITTQESESNILDIDLRPASAWTLYAVATTFVTPMLRHSIENSSVNVSLTAIANQATVLMGLGKGWPLSKMDIGVPLLAIGCYSQVNPITLTAALLLLVAHYAIIGPGLQAKATREAQKRAAAGIMKNPTVDGITVIDLEPIPYDPKFEKQLGQVMLLILCVTQVLMMRTTWALCEALTLATGPISTLWEGNPGRFWNTTIAVSMANIFRGSYLAGAGLLFSIMKNTTNTRRGTGNIGETLGEKWKSRLNALGKSEFQIYKKSGIQEVDRTLAKEGIKRGETDHHAVSRGSAKLRWFVERNMVTPEGKVVDLGCGRGGWSYYCGGLKNVREVKGLTKGGPGHEEPIPMSTYGWNLVRLQSGVDVFFTPPEKCDTLLCDIGESSPNPTIEAGRTLRVLNLVENWLNNNTQFCIKVLNPYMPSVIEKMETLQRKYGGALVRNPLSRNSTHEMYWVSNATGNIVSSVNMISRMLINRFTMKHKKATYELDVDLGSGTRNIGIESETPNLDIIGKRIEKIKQEHETSWHYDQDHPYKTWAYHGSYETKQTGSASSMVNGVVRLLTKPWDVVPMVTQMAMTDTTPFGQQRVFKEKVDTRTQEPKEGTKKLMRITAEWLWKELGKKKTPRMCTREEFTRKVRSNAALGAIFTDENKWKSAREAVEDSRFWELVDRERNLHLEGKCETCVYNMMGKREKKLGEFGKAKGSRAIWYMWLGARFLEFEALGFLNEDHWFSRGNSLSGVEGEGLHRLGYILRDVSKKEGGAMYADDTAGWDTRITLEDLKNEEMVTNHMKGEHKKLAEAIFKLTYQNKVVRVQRPTPRGTVMDIISRRDQRGSGQVGTYGLNTFTNMEAQLIRQMEGEGIFKSIQHLTVTEEIAVQNWLARVGRERLSRMAISGDDCVVKPLDDRFASALTALNDMGKVRKDIQQWEPSRGWNDWTQVPFCSHHFHELVMKDGRVLVVPCRNQDELIGRARISQGAGWSLKETACLGKSYAQMWTLMYFHRRDLRLAANAICSAVPSHWVPTSRTTWSIHAKHEWMTTEDMLAVWNRVWIQENPWMEDKTPVESWEEVPYLGKREDQWCGSLIGLTSRATWAKNIQTAINQVRSLIGNEEYTDYMPSMKRFRREEEEAGVLW

>DENV2_AET72454_USA

MNNQRKKARNTPFNMLKRERNRVSTVQQLTKRFSLGMLQGRGPLKLFMALVAFLRFLTIPPTAGILKRWGTIKKSKAINVLRGFRKEIGRMLNILNRRRRTAGMIIMLIPTVMAFHLTTRNGEPHMIVSRQEKGKSLLFKTGDGVNMCTLMAMDLGELCEDTITYKCPLLRQNEPEDIDCWCNSTSTWVTYGTCTTTGEHRREKRSVALVPHVGMGLETRTETWMSSEGAWKHAQRIETWILRHPGFTIMAAILAYTIGTTHFQRALIFILLTAVAPSMTMRCIGISNRDFVEGVSGGSWVDIVLEHGSCVTTMAKNKPTLDFELIKTEAKQSATLRKYCIEAKLTNTTTESRCPTQGEPSLNEEQDKRFVCKHSMVDRGWGNGCGLFGKGGIVTCAMFTCKKNMKGEVVQPENLEYTIVITPHSGEEHAVGNDTGKHGKEIKITPQSSITEAELTGYGTVTMECSPRTGLDFNEMVLLQMENKAWLVHRQWFLDLPLPWLPGADTQGSNWIQKETLVTFKNPHAKKQDVVVLGSQEGAMHTALTGATEIQMSSGNLLFTGHLKCRLRMDKLQLKGMSYSMCTGKFKVVKEIAETQHGTIVIRVQYEGDGSPCKIPFEIMDLEKRHVLGRLITVNPIVTEKDSPVNIEAEPPFGDSYIIIGVEPGQLKLNWFKKGSSIGQMLETTMRGAKRMAILGDTAWDFGSLGGVFTSIGKALHQVFGAIYGAAFSGVSWTMKILIGVIITWIGMNSRSTSLSVSLVLVGVVTLYLGVMVQADSGCVVSWKNKELKCGSGIFITDNVHTWTEQYKFQPESPSKLASAIQKAHEEGICGIRSVTRLENLMWKQITPELNHILSENEVKLTIMTGDIKGIMQAGKRSLRPQPTELKYSWKTWGKAKMLSTESHNQTFLIDGPETAECPNTNRAWNSLEVEDYGFGVFTTNIWLKLREKQDVFCDSKLMSAAIKDNRAVHADMGYWIESALNDTWKIEKASFIEVKSCHWPKSHTLWSNEVLESEMIIPKNFAGPVSQHNYRPGYHTQTAGPWHLGKLEMDFDFCEGTTVVVTEDCGNRGPSLRTTTASGKLITEWCCRSCTLPPLRYRGEDGCWYGMEIRPLKEKEENLVNSLVTAGHGQIDNFSLGVLGMALFLEEMLRTRVGTKHAILLVAVSFVTLITGNMSFRDLGRVMVMVGATMTDDIGMGVTYLALLAAFKVRPTFAAGLLLRKLTSKELMMTTIGIVLLSQSTIPETILELTDALALGMMVLKMVRKMEKYQLAVTIMAILCVPNAVILQNAWKVSCTILAVVSVSPLFLTSSQQKADWIPLALTIKGLNPTAIFLTTLSRTNKKRSWPLNEAIMAVGMVSILASSLLKNDIPMTGPLVAGGLLTVCYVLTGRSADLELERAADVKWEDQAEISGSSPILSITISEDGSMSIKNEEEEQTLTILIRTGLLVISGLFPVSLPITAAAWYLWEVKKQRAGVLWDVPSPPPVGKAELEDGAYRIKQKGILGYSQIGAGVYKEGTFHTMWHVTRGAVLMHKGKRIEPSWADVKKDLISYGGGWKLEGEWKEGEEVQVLALEPGKNPRAVQTKPGLFKTNAGTIGAVSLDFSPGTSGSPIIDKKGKVVGLYGNGVVTRSGAYVSAIAQTEKSIEDNPEIEDDIFRKRKLTIMDLHPGAGKTKRYLPAIVREAIKRGLRTLILAPTRVVAAEMEEALRGLPIRYQTPAIRAEHTGREIVDLMCHATFTMRLLSPVRVPNYNLIIMDEAHFTDPASIAARGYISTRVEMGEAAGIFMTATPPGSRDPFPQSNAPIMDEEREIPERSWSSGHEWVTDFKGKTVWFVPSIKAGNDIAACLRKNGKKVIQLSRKTFDSEYVKTRTNDWDFVVTTDISEMGANFKAERVIDPRRCMKPVILTDGEERVILAGPMPVTHSSAAQRRGRIGRNPKNENDQYIYMGEPLENDEDCAHWKEAKMLLDNINTPEGIIPSMFEPEREKVDAIDGEYRLRGEARKTFVDLMRRGDLPVWLAYRVAAEGINYADRRWCFDGIKNNQILEENVEVEIWTKEGERKKLKPRWLDARIYSDPLALKEFKEFAAGRKSLTLSLITEMGRLPTFMTQKARDALDNLAVLHTAEAGGRAYNHALSELPETLETLLLLTLLATVTGGIFLFLMSGRGIGKMTLGMCCIITASILLWYAQIQPHWIAASIILEFFLIVLLIPEPEKQRTPQDNQLTYVVIAILTVVAATMANEMGFLEKTKKDLGLGSITTQQPESNILDIDLRPASAWTLYAVATTFVTPMLRHSIENSSVNVSLTAIANQATVLMGLGKGWPLSKMDIGVPLLAIGCYSQVNPITLTAALFLLVAHYAIIGPGLQAKATREAQKRAAAGIMKNPTVDGITVIDLDPIPYDPKFEKQLGQVMLLVLCVTQVLMMRTTWALCEALTLATGPISTLWEGNPGRFWNTTIAVSMANIFRGSYLAGAGLLFSIMKNTTNTRRGTGNIGETLGEKWKSRLNALGKSEFQIYKKSGIQEVDRTLAKEGIKRGETDHHAVSRGSAKLRWFVERNMVTPEGKVVDLGCGRGGWSYYCGGLKNVREVKGLTKGGPGHEEPIPMSTYGWNLVRLQSGVDVFFTPPEKCDTLLCDIGESSPNPTVEAGRTLRVLNLVENWLNNNTQFCIKVLNPYMPSVIEKMEALQRKYGGALVRNPLSRNSTHEMYWVSNASGNIVSSVNMISRMLINRFTMRHKKATYEPDVDLGSGTRNIGIESEIPNLDIIGKRIEKIKQEHETSWHYDQDHPYKTWAYHGSYETKQTGSASSMVNGVVRLLTKPWDVVPMVTQMAMTDTTPFGQQRVFKEKVDTRTQEPKEGTKKLMKITAEWLWKELGKKKTPRMCTREEFTRKVRSNAALGAIFTDENKWKSAREAVEDSRFWELVDKERNLHLEGKCETCVYNMMGKREKKLGEFGKAKGSRAIWYMWLGARFLEFEALGFLNEDHWFSRENSLSGVEGEGLHKLGYILRDVSKKEGGAMYADDTAGWDTRITLEDLKNEEMVTNHMEGEHKKLAEAIFKLTYQNKVVRVQRPTPRGTVMDIISRRDQRGSGQVGTYGLNTFTNMEAQLIRQMEGEGVFKSIQHLTVTEEIAVQNWLARVGRERLSRMAISGDDCVVKPLDDRFASALTALNDMGKVRKDIQQWEPSRGWNDWTQVPFCSHHFHELIMKDGRVLVVPCRNQDELIGRARISQGAGWSLRETACLGKSYAQMWSLMYFHRRDLRLAANAICSAVPSHWVPTSRTTWSIHAKHEWMTAEDMLTVWNRVWIQENPWMEDKTPVESWEEIPYLGKREDQWCGSLIGLTSRATWAKNIQTAINQVRSLIGNEEYTDYMPSMKRFRREEEEAGVLW

>DENV2_AET43238_Venezuela

MNNQRKKARSTPFNMLKRERNRVSTVQQLTKRFSLGMLQGRGPLKLFMALVAFLRFLTIPPTAGILKRWGTIKKSKAINVLRGFRKEIGRMLNILNRRRRTAGVIVMLIPTAMAFHLTTRNGEPHMIVGRQEKGKSLLFKTEDGVNMCTLMAIDLGELCEDTITYKCPLLRQNEPEDIDCWCNSTSTWVTYGTCTTTGEHRREKRSVALVPHVGMGLETRTETWMSSEGAWKHVQRIETWILRHPGFTIMAAILAYTIGTTHFQRALIFILLTAVAPSMTMRCIGISNRDFVEGVSGGSWVDIVLEHGSCVTTMAKNKPTLDFELIKTEAKQPATLRKYCIEAKLTNTTTESRCPTQGEPSLNEEQDKRFICKHSMVDRGWGNGCGLFGKGGIVTCAMFTCKKNMEGKVVQPENLEYTIVITPHSGEEHAVGNDTGKHGKEIKITPQSSITEAELTGYGTVTMECSPRTGLDFNEMVLLQMEDKAWLVHRQWFLDLPLPWLPGADTQGSNWIQKETLVTFKNPHAKKQDVVVLGSQEGAMHTALTGATEIQMSLGNLLFTGHLKCRLRMDKLQLKGMSYSMCTGKFKIVKEIAETQHGTIVIRVQYEGDGSPCKIPFEITDLEKRHVLGRLITVNPIVIEKDSPVNIEAEPPFGDSYIIIGVEPGQLKLNWFKKGSSIGQMFETTMRGAKRMAILGDTAWDFGSLGGVFTSIGKALHQVFGAIYGAAFSGVSWTMKILIGVVITWIGMNSRSTSLSVSLVLVGVVTLYLGAMVQADSGCVVSWKNKELKCGSGIFITDNVHTWTEQYKFQPESPSKLASAIQKAHEEGICGIRSVTRLENLMWKQITPELNHILSENEVKLTIMTGDTKGIMQAGKRSLRPQPTELKYSWKTWGKAKMLFTESHNQTFLIDGPETAECPNTNRAWNSLEVEDYGFGVFSTNIWLKLREKQDVFCDSKLMSAAIKDNRAVHADMGYWIESALNDTWKMEKASFIEVKSCHWPKSHTLWSNGVLESEMIIPKNFAGPVSQHNYRPGYHTQTAGPWHLGKLEMDFDFCEGTTVVVTEDCGNRGPSLRTTTASGKLITEWCCRSCTLPPLRYRGEDGCWYGMEIRPLKEKEENLVNSLVTAGHGQIDNFSLGVLGMALFLEEMLRTRVGTKHAILLVAVSFVTLITGNMSFRDLGRVMVMVGATMTDDIGMGVTYLALLAAFKVRPTFAAGLLLRKLTSKELMMATIGITLLSQSTIPETILELTDALALGMMVLKIVRNMEKYQLAVTIMAISCVPNAVILRNAWKVSCTILAAVSVSPLLLTSSQQKADWIPLALTIKGLNPTAIFLTTLSRTSKKRSWPLNEAIMAVGMVSILASSLLKNDIPMTGPLVAGGLLTVCYVLTGRSADLELERAADVKWEDQAEISGSSPILSITISEDGSMSIKNEEEEQTLTILIRTGLLVISGVFPVSIPITAAAWYLWEVKKQRAGVLWDVPSPPPVGKAELEDGAYRIKQRGILGYSQIGAGVYKEGTFHTMWHVTRGAVLMHRGKRIEPSWADVKKDLISYGGGWKLEGEWKEGEEVQVLALEPGKNPRAVQTKPGIFKTNTGTIGAVSLDFSPGTSGSPIVDRKGKVVGLYGNGVVTRSGAYVSAIAQTEKSIEDNPEIEDDIFRKKRLTIMDLHPGAGKTKRYLPAIVREAIKRGLRTLILAPTRVVAAEMEEALRGLPIRYQTPAIKAEHTGREIVDLMCHATFTMRLLSPVRVPNYNLIIMDEAHFTDPASIAARGYISTRVEMGEAAGIFMTATPPGSRDPFPQSNAPIMDEEREIPERSWNSGHEWVTDFKGKTVWFVPSIKAGNDIAACLRKNGKKVIQLSRKTFDSEYVKTRANDWDFVVTTDISEMGANFKAERVIDPRRCMKPVILTDGEERVILAGPMPVTHSSAAQRRGRIGRNPKNENDQYIYMGEPLENDEDCAHWKEAKMLLDNINTPEGIIPSMFEPEREKVDAIDGEYRLRGEARKTFVDLMRRGDLPVWLAYKVAAEGINYADRKWCFDGIKNNQILEENVEVEIWTKEGERKKLKPRWLDARIYSDPLALKEFKEFAAGRKSLTLNLITEMGRLPTFMTQKARNALDNLAVLHTAEAGGRAYNHALSELPETLETLLLLTLLATVTGGIFLFLMSGKGIGKMTLGMCCIITASVLLWYAQIQPHWIAASIILEFFLIVLLIPEPEKQRTPQDNQLTYVVIAILTVVAATMANEMGFLEKTKKDLGLGSITTQEPESNILDIDLRPASAWTLYAVATTFVTPMLRHSIENSSVNVSLTAIANQATVLMGLGKGWPLSKMDIGVPLLAIGCYSQVNPITLTAALLLLVAHYAIIGPGLQAKATREAQKRAAAGIMKNPTVDGITVIDLEPIPYDPKFEKQLGQVMLLILCVTQVLMMRTTWALCEALTLATGPISTLWEGNPGRFWNTTIAVSMANIFRGSYLAGAGLLFSIMKNTTNTRRGTGNIGETLGEKWKSRLNALGKSEFQIYKKSGIQEVDRTLAKEGIKRGETDHHAVSRGSAKLRWFVERNMVTPEGKVVDLGCGRGGWSYYCGGLKNVREVKGLTKGGPGHEEPIPMSTYGWNLVRLQSGVDVFFTPPEKCDTLLCDIGESSPNPTIEAGRTLRVLNLVENWLNNNTQFCIKVLNPYMPSVIEKMETLQRKYGGALVRNPLSRNSTHEMYWVSNATGNIVSSVNMISRMLINRFTMKHKKATYEPDVDLGSGTRNIGIESEIPNLDIIGKRIEKIKQEHETSWHYDQDHPYKTWAYHGSYETKQTGSASSMVNGVVRLLTKPWDVVPMVTQMAMTDTTPFGQQRVFKEKVDTRTQEPKEGTKKLMRITAEWLWKELGKKKTPRMCTREEFTRKVRSNAALGAIFTDENKWKSAREAVEDGRFWELVDRERNLHLEGKCETCVYNMMGKREKKLGEFGKAKGSRAIWYMWLGARFLEFEALGFLNEDHWFSRGNSLSGVEGEGLHRLGYILRDVGKKEGGAMYADDTAGWDTRITLEDLKNEEMVTNHMKGEHKKLAEAIFKLTYQNKVVRVQRPTPRGTVMDIISRRDQRGSGQVGTYGLNTFTNMEAQLIRQMEGEGIFKSIQHLTVTEEIAVQNWLARVGRERLSRMAISGDDCVVKPIDDRFASALTALNDMGKVRKDIQQWEPSRGWNDWTQVPFCSHHFHELVMKDGRVLVVPCRNQDELIGRARISQGAGWSLKETACLGKSYAQMWTLMYFHRRDLRLAANAICSAVPSHWVPTSRTTWSIHAKHEWMTTEDMLAVWNRVWIQENPWMEDKTPVESWEEVPYLGKREDQWCGSLIGLTSRATWAKNIQTAINQVRSLIGNEEYTDYMPSMKRFRREEEEAGVLW

>DENV2_AER45462_Guatemala

MNNQRKKARSTPFNMLKRERNRVSTVQQLTKRFSLGMLQGRGPLKLFMALVAFLRFLTIPPTAGILKRWGTIKKSKAINVLRGFRKEIGRMLNILNRRRRTAGVIVMLIPTAMAFHLTTRNGEPHMIVGRQEKGKSLLFKTEDGVNMCTLMAIDLGELCEDTITYKCPLLRQNEPEDIDCWCNSTSTWVTYGTCTTTGEHRREKRSVALVPHVGMGLETRTETWMSSEGAWKHIQRIETWILRHPGFTIMAAILAYTIGTTHFQRALIFILLTAVAPSMTMRCIGISNRDFVEGVSGGSWVDIVLEHGSCVTTMAKNKPTLDFELIKTEAKQPATLRKYCIEAKLTNTTTESRCPTQGEPSLNEEQDKRFICKHSMVDRGWGNGCGLFGKGGIVTCAMFTCKKNMEGKVVQPENLEYTIVITPHSGEEHAVGNDTGKHGKEIKITPQSSITEAELTGYGTVTMECSPRTGLDFNEMVLLQMEDKAWLVHRQWFLDLPLPWLPGADTQGSNWIQKETLVTFKNPHAKKQDVVVLGSQEGAMHTALTGATEIQMSSGNLLFTGHLKCRLRMDKLQLKGMSYSMCTGKFKIVKEIAETQHGTIVIRVQYEGDGSPCKIPFEITDLEKRHVLGRLITVNPIVTEKDSPINIEAEPPFGDSYIIIGVEPGQLKLNWFKKGSSIGQMFETTMRGAKRMAILGDTAWDFGSLGGVFTSIGKALHQVFGAIYGAAFSGVSWTMKILIGVIITWIGMNSRSTSLSVSLVLVGVVTLYLGAMVQADSGCVVSWKNKELKCGSGIFITDNVHTWTEQYKFQPESPSKLASAIQKAHEEGICGIRSVTRLENLMWKQITPELNHILSENEVKLTIMTGDIKGIMQAGKRSLRPQPTELKYSWKTWGKAKMLSTESHNQTFLIDGPETAECPNTNRAWNSLEVEDYGFGVFTTNIWLKLKEKQDVFCDSKLMSAAIKDNRAVHADMGYWIESALNDTWKMEKASFIEVKSCHWPKSHTLWSNGVLESEMIIPKNFAGPVSQHNYRPGYHTQTAGPWHLGKLEMDFDLCEGTTVVVTEDCGNRGPSLRTTTASGKLITEWCCRSCTLPPLRYRGEDGCWYGMEIRPLKEKEENLVNSLVTAGHGQIDNFSLGVLGMALFLEEMLRTRIGTKHAILLVAVSFVTLITGNMSFRDLGRVMVMVGATMTDDIGMGVTYLALLAAFKVRPTFAAGLLLRKLTSKELMMATIGIALLSQSTIPETILELTDALALGMMVLKVVRNMEKYQLAVTIMAISCVPNAVILQNAWKVSCTILAAVSVSPLLLTSSQQKADWIPLALTIKGLNPTAIFLTTLSRTSTKRSWPLNEAIMAVGMVSILASSLLKNDIPMTGPLVAGGLLTVCYVLTGRSADLELERAADVKWEDQAEISGSSPILSITISEDGSMSIKNEEEEQTLTILIRTGLLVISGVFPVSIPITAAAWYLWEVKKQRAGVLWDVPSPPPVEKAELEDGAYRIKQRGILGYSQIGAGVYKEGTFHTMWHVTRGAVLMHRGKRIEPSWADVKKDLISYGGGWKLEGEWKEGEEVQVLALEPGKNPRAVQTKPGIFKTNTGTIGAVSLDFSPGTSGSPIVDRKGKVVGLYGNGVVTRSGAYVSAIAQTEKSIEDNPEIEDDIFRKKRLTIMDLHPGAGKTKRYLPAIVREAIKRGLRTLILAPTRVVAAEMEEALRGLPIRYQTPAIKTEHTGREIVDLMCHATFTMRLLSPVRVPNYNLIIMDEAHFTDPASIAARGYISTRVEMGEAAGIFMTATPPGSRDPFPQSNAPIMDEEREIPERSWNSGHEWVTDFKGKTVWFVPSIKAGNDIAACLRKNGKKVIQLSRKTFDSEYVKTRANDWDFVVTTDISEMGANFKAERVIDPRRCMKPVILTDGEERVILAGPMPVTHSSAAQRRGRIGRNPKNENDQYIYMGEPLENDEDCAHWKEAKMLLDNINTPEGIIPSMFEPEREKVDAIDGEYRLRGEARKTFVDLMRRGDLPVWLAYKVAAEGINYADRRWCFDGIKNNQILEENMEVEIWTKEGERKKLKPRWLDARIYSDPLALKEFKEFAAGRKSLTLNLITEMGRLPTFMTQKARNALDNLAVLHTAEAGGRAYNHALSELPETLETLLLLTLLATVTGGIFLFLMSGKGIGKMTLGMCCIITASILLWYAQIQPHWIAASIILEFFLIVLLIPEPEKQRTPQDNQLTYVVIAILTVVAATMANEMGFLEKTKKDLGLGSITTQESESNILDIDLRPASAWTLYAVATTFLTPMLRHSIENSSVNVSLTAIANQATVLMGLGKGWPLSKMDIGAPLLAIGCYSQVNPITLTAALLLLVAHYAIIGPGLQAKATREAQKRAAAGIMKNPTVDGITVIDLDPIPYDPKFEKQLGQVMLLILCVTQVLMMRTTWALCEALTLATGPISTLWEGNPGRFWNTTIAVSMANIFRGSYLAGAGLLFSIMKNTTSTRRGTGNIGETLGEKWKSRLNALGKSEFQIYKKSGIQEVDRTLAKEGIKRGETDHHAVSRGSAKLRWFVERNMVTPEGKVVDLGCGRGGWSYYCGGLKNVREVKGLTKGGPGHEEPIPMSTYGWNLVRLQSGVDVFFTPPEKCDTLLCDIGESSPNPTIEAGRTLRVLNLVENWLNNNTQFCIKVLNPYMPSVIEKMEALQRKYGGALVRNPLSRNSTHEMYWVSNATGNIVSSVNMISRMLINRFTMKHKKATYEPDVDLGSGTRNIGIESEVPNLDIIGKRIEKIKQEHETSWHYDQDHPYKTWAYHGSYETKQTGSASSMVNGVVRLLTKPWDVVPMVTQMAMTDTTPFGQQRVFKEKVDTRTQEPKEGTKKLMKITAEWLWKELGKKKTPRMCTREEFTRKVRSNAALGAIFTDENKWKSAREAVEDGRFWELVDRERNLHLEGKCETCVYNMMGKREKKLGEFGKAKGSRAIWYMWLGARFLEFEALGFLNEDHWFSRGNSLSGVEGEGLHRLGYILREVSKKEGGAMYADDTAGWDTRITLEDLKNEEMVTNHMKGEHKKLAEAIFKLTYQNKVVRVQRPTPRGTVMDIISRRDQRGSGQVGTYGLNTFTNMEAQLIRQMEGEGIFKSIQHLTATEEIAVQNWLARVGRERLSRMAISGDDCVVKPIDDRFASALTALNDMGKVRKDIQQWEPSRGWNDWTQVPFCSHHFHELVMKDGRVLVVPCRNQDELIGRARISQGAGWSLKETACLGKSYAQMWTLMYFHRRDLRLAANAICSAVPSHWVPTSRTTWSIHAKHEWMTTEDMLAVWNRVWIQENPWMEDKTPVESWEEVPYLGKREDQWCGSLIGLTSRATWAKNIQTAINQVRSLIGNEEYTDYMPSMKRFRREEEEAGVLW

>DENV2_AEH59348_USA

MNNQRKKARNTPFNMLKRERNRVSTVQQLTKRFSLGMLQGRGPLKLFMALVAFLRFLTIPPTAGILKRWGTIKKSKAINVLRGFRKEIGRMLNILNRRRRTAGMIIMLIPTVMAFHLTTRNGEPHMIVSRQEKGKSLLFKTGDGVNMCTLMAMDLGELCEDTITYKCPLLRQNEPEDIDCWCNSTSTWVTYGTCTTTGEHRREKRSVALVPHVGMGLETRTETWMSSEGAWKHAQRIETWILRHPGFTIMAAILAYTIGTTHFQRALIFILLTAVAPSMTMRCIGISNRDFVEGVSGGSWVDIVLEHGSCVTTMAKNKPTLDFELIKTEAKQSATLRKYCIEAKLTNTTTESRCPTQGEPSLNEEQDKRFVCKHSMVDRGWGNGCGLFGKGGIVTCAMFTCKKDMKGEVVQPENLEYTIVITPHSGEEHAVGNDTGKHGKEIKITPQSSITEAELTGYGTVTMECSPRTGLDFNEMVLLQMENKAWLVHRQWFLDLPLPWLPGADTQGSNWIQKETLVTFKNPHAKKQDVVVLGSQEGAMHTALTGATEIQMSSGNLLFTGHLKCRLRMDKLQLKGMSYSMCTGKFKVVKEIAETQHGTIVIRVQYEGDGSPCKIPFEIMDLEKRHVLGRLITVNPIVTEKDSPVNIEAEPPFGDSYIIIGVEPGQLKLNWFKKGSSIGQMLETTMRGAKRMAILGDTAWDFGSLGGVFTSIGKALHQVFGAIYGAAFSGVSWTMKILIGVIITWIGMNSRSTSLSVSLVLVGVVTLYLGVMVQADSGCVVSWKNKELKCGSGIFITDNVHTWTEQYKFQPESPSKLASAIQKAHEEGICGIRSVTRLENLMWKQITPELNHILSENEVKLTIMTGDIKGIMQAGKRSLRPQPTELKYSWKTWGKAKMLSTESHNQTFLIDGPETAECPNTNRAWNSLEVEDYGFGVFTTNIWLKLREKQDVFCDSKLMSAAIKDNRAVHADMGYWIESALNDTWKIEKASFIEVKSCHWPKSHTLWSNEVLESEMIIPKNFAGPVSQHNYRPGYHTQTAGPWHLGKLEMDFDFCEGTTVVVTEDCGNRGPSLRTTTASGKLITEWCCRSCTLPPLRYRGEDGCWYGMEIRPLKEKEENLVNSLVTAGHGQIDNFSLGVLGMALFLEEMLRTRVGTKHAILLVAVSFVTLITGNMSFRDLGRVMVMVGATMTDDIGMGVTYLALLAAFKVRPTFAAGLLLRKLTSKELMMTTIGIVLLSQSTIPETILELTDALALGMMVLKMVRKMEKYQLAVTIMAILCVPNAVILQNAWKVSCTILAVVSVSPLFLTSSQQKADWIPLALTIKGLNPTAIFLTTLSRTNKKRSWPLNEAIMAVGMVSILASSLLKNDIPMTGPLVAGGLLTVCYVLTGRSADLELERAADVKWEDQAEISGSSPILSITISEDGSMSIKNEEEEQTLTILIRTGLLVISGLFPVSLPITAAAWYLWEVKKQRAGVLWDVPSPPPVGKAELEDGAYRIKQKGILGYSQIGAGVYKEGTFHTMWHVTRGAVLMHKGKRIEPSWADVKKDLISYGGGWKLEGEWKEGEEVQVLALEPGKNPRAVQTKPGLFKTNAGTIGAVSLDFSPGTSGSPIIDKKGKVVGLYGNGVVTRSGAYVSAIAQTEKSIEDNPEIEDDIFRKRKLTIMDLHPGAGKTKRYLPAIVREAIKRGLRTLILAPTRVVAAEMEEALRGLPIRYQTPAIRAEHTGREIVDLMCHATFTMRLLSPVRVPNYNLIIMDEAHFTDPASIAARGYISTRVEMGEAAGIFMTATPPGSRDPFPQSNAPIMDEEREIPERSWSSGHEWVTDFKGKTVWFVPSIKAGNDIAACLRKNGKKVIQLSRKTFDSEYVKTRTNDWDFVVTTDISEMGANFKAERVIDPRRCMKPVILTDGEERVILAGPMPVTHSSAAQRRGRIGRNPKNENDQYIYMGEPLENDEDCAHWKEAKMLLDNINTPEGIIPSMFEPEREKVDAIDGEYRLRGEARKTFVDLMRRGDLPVWLAYRVAAEGINYADRRWCFDGIKNNQILEENVEVEIWTKEGERKKLKPRWLDARIYSDPLALKEFKEFAAGRKSLTLSLITEMGRLPTFMTQKARDALDNLAVLHTAEAGGRAYNHALSELPETLETLLLLTLLATVTGGIFLFLMSGRGIGKMTLGMCCIITASILLWYAQIQPHWIAASIILEFFLIVLLIPEPEKQRTPQDNQLTYVVIAILTVVAATMANEMGFLEKTKKDLGLGSITTQQPESNILDIDLRPASAWTLYAVATTFVTPMLRHSIENSSVNVSLTAIANQATVLMGLGKGWPLSKMDIGVPLLAIGCYSQVNPITLTAALFLLVAHYAIIGPGLQAKATREAQKRAAAGIMKNPTVDGITVIDLDPIPYDPKFEKQLGQVMLLVLCVTQVLMMRTTWALCEALTLATGPISTLWEGNPGRFWNTTIAVSMANIFRGSYLAGAGLLFSIMKNTTNTRRGTGNIGETLGEKWKSRLNALGKSEFQIYKKSGIQEVDRTLAKEGIKRGETDHHAVSRGSAKLRWFVERNMVTPEGKVVDLGCGRGGWSYYCGGLKNVREVKGLTKGGPGHEEPIPMSTYGWNLVRLQSGVDVFFTPPEKCDTLLCDIGESSPNPTVEAGRTLRVLNLVENWLNNNTQFCIKVLNPYMPSVIEKMEALQRKYGGALVRNPLSRNSTHEMYWVSNASGNIVSSVNMISRMLINRFTMRHKKATYEPDVDLGSGTRNIGIESEIPNLDIIGKRIEKIKQEHETSWHYDQDHPYKTWAYHGSYETKQTGSASSMVNGVVRLLTKPWDVVPMVTQMAMTDTTPFGQQRVFKEKVDTRTQEPKEGTKKLMKITAEWLWKELGKKKTPRMCTREEFTRKVRSNAALGAIFTDENKWKSAREAVEDSRFWELVDKERNLHLEGKCETCVYNMMGKREKKLGEFGKAKGSRAIWYMWLGARFLEFEALGFLNEDHWFSRENSLSGVEGEGLHKLGYILRDVSKKEGGAMYADDTAGWDTRITLEDLKNEEMVTNHMEGEHKKLAEAIFKLTYQNKVVRVQRPTPRGTVMDIISRRDQRGSGQVGTYGLNTFTNMEAQLIRQMEGEGVFKSIQHLTVTEEIAVQNWLARVGRERLSRMAISGDDCVVKPLDDRFASALTALNDMGKVRKDIQQWEPSRGWNDWTQVPFCSHHFHELIMKDGRVLVVPCRNQDELIGRARISQGAGWSLRETACLGKSYAQMWSLMYFHRRDLRLAANAICSAVPSHWVPTSRTTWSIHAKHEWMTAEDMLTVWNRVWIQENPWMEDKTPVESWEEIPYLGKREDQWCGSLIGLTSRATWAKNIQTAINQVRSLIGNEEYTDYMPSMKRFRREEEEAGVLW

>DENV2_AEH59347_USA

MNNQRKKARNTPFNMLKRERNRVSTVQQLTKRFSLGMLQGRGPLKLFMALVAFLRFLTIPPTAGILKRWGTIKKSKAINVLRGFRKEIGRMLNILNRRRRTAGMIIMLIPTVMAFHLTTRNGEPHMIVSRQEKGKSLLFKTGDGVNMCTLMAMDLGELCEDTITYKCPLLRQNEPEDIDCWCNSTSTWVTYGTCTTTGEHRREKRSVALVPHVGMGLETRTETWMSSEGAWKHAQRIETWILRHPGFTIMAAILAYTIGTTHFQRALIFILLTAVAPSMTMRCIGISNRDFVEGVSGGSWVDIVLEHGSCVTTMAKNKPTLDFELIKTEAKQSATLRKYCIEAKLTNTTTESRCPTQGEPSLNEEQDKRFVCKHSMVDRGWGNGCGLFGKGGIVTCAMFTCKKNMKGEVVQPENLEYTIVITPHSGEEHAVGNDTGKHGKEIKITPQSSITEAELTGYGTVTMECSPRTGLDFNEMVLLQMENKAWLVHRQWFLDLPLPWLPGADTQGSNWIQKETLVTFKNPHAKKQDVVVLGSQEGAMHTALTGATEIQMSSGNLLFTGHLKCRLRMDKLQLKGMSYSMCTGKFKVVKEIAETQHGTIVIRVQYEGDGSPCKIPFEIMDLEKRHVLGRLITVNPIVTEKDSPVNIEAEPPFGDSYIIIGVEPGQLKLNWFKKGSSIGQMLETTMRGAKRMAILGDTAWDFGSLGGVFTSIGKALHQVFGAIYGAAFSGVSWTMKILIGVIITWIGMNSRSTSLSVSLVLVGVVTLYLGVMVQADSGCVVSWKNKELKCGSGIFITDNVHTWTEQYKFQPESPSKLASAIQKAHEEGICGIRSVTRLENLMWKQITPELNHILSENEVKLTIMTGDIKGIMQAGKRSLRPQPTELKYSWKTWGKAKMLSTESHNQTFLIDGPETAECPNTNRAWNSLEVEDYGFGVFTTNIWLKLREKQDVFCDSKLMSAAIKDNRAVHADMGYWIESALNDTWKIEKASFIEVKSCHWPKSHTLWSNEVLESEMIIPKNFAGPVSQHNYRPGYHTQTAGPWHLGKLEMDFDFCEGTTVVVTEDCGNRGPSLRTTTASGKLITEWCCRSCTLPPLRYRGEDGCWYGMEIRPLKEKEENLVNSLVTAGHGQIDNFSLGVLGMALFLEEMLRTRVGTKHAILLVAVSFVTLITGNMSFRDLGRVMVMVGATMTDDIGMGVTYLALLAAFKVRPTFAAGLLLRKLTSKELMMTTIGIVLLSQSTIPETILELTDALALGMMVLKMVRKMEKYQLAVTIMAILCVPNAVILQNAWKVSCTILAVVSVSPLFLTSSQQKADWIPLALTIKGLNPTAIFLTTLSRTNKKRSWPLNEAIMAVGMVSILASSLLKNDIPMTGPLVAGGLLTVCYVLTGRSADLELERAADVKWEDQAEISGSSPILSITISEDGSMSIKNEEEEQTLTILIRTGLLVISGLFPVSLPITAAAWYLWEVKKQRAGVLWDVPSPPPVGKAELEDGAYRIKQKGILGYSQIGAGVYKEGTFHTMWHVTRGAVLMHKGKRIEPSWADVKKDLISYGGGWKLEGEWKEGEEVQVLALEPGKNPRAVQTKPGLFKTNAGTIGAVSLDFSPGTSGSPIIDKKGKVVGLYGNGVVTRSGAYVSAIAQTEKSIEDNPEIEDDIFRKRKLTIMDLHPGAGKTKRYLPAIVREAIKRGLRTLILAPTRVVAAEMEEALRGLPIRYQTPAIRAEHTGREIVDLMCHATFTMRLLSPVRVPNYNLIIMDEAHFTDPASIAARGYISTRVEMGEAAGIFMTATPPGSRDPFPQSNAPIMDEEREIPERSWSSGHEWVTDFKGKTVWFVPSIKAGNDIAACLRKNGKKVIQLSRKTFDSEYVKTRTNDWDFVVTTDISEMGANFKAERVIDPRRCMKPVILTDGEERVILAGPMPVTHSSAAQRRGRIGRNPKNENDQYIYMGEPLENDEDCAHWKEAKMLLDNINTPEGIIPSMFEPEREKVDAIDGEYRLRGEARKTFVDLMRRGDLPVWLAYRVAAEGINYADRRWCFDGIKNNQILEENVEVEIWTKEGERKKLKPRWLDARIYSDPLALKEFKEFAAGRKSLTLSLITEMGRLPTFMTQKARDALDNLAVLHTAEAGGRAYNHALSELPETLETLLLLTLLATVTGGIFLFLMSGRGIGKMTLGMCCIITASILLWYAQIQPHWIAASIILEFFLIVLLIPEPEKQRTPQDNQLTYVVIAILTVVAATMANEMGFLEKTKKDLGLGSITTQQPESNILDIDLRPASAWTLYAVATTFVTPMLRHSIENSSVNVSLTAIANQATVLMGLGKGWPLSKMDIGVPLLAIGCYSQVNPITLTAALFLLVAHYAIIGPGLQAKATREAQKRAAAGIMKNPTVDGITVIDLDPIPYDPKFEKQLGQVMLLVLCVTQVLMMRTTWALCEALTLATGPISTLWEGNPGRFWNTTIAVSMANIFRGSYLAGAGLLFSIMKNTTNTRRGTGNIGETLGEKWKSRLNALGKSEFQIYKKSGIQEVDRTLAKEGIKRGETDHHAVSRGSAKLRWFVERNMVTPEGKVVDLGCGRGGWSYYCGGLKNVREVKGLTKGGPGHEEPIPMSTYGWNLVRLQSGVDVFFTPPEKCDTLLCDIGESSPNPTVEAGRTLRVLNLVENWLNNNTQFCIKVLNPYMPSVIEKMEALQRKYGGALVRNPLSRNSTHEMYWVSNASGNIVSSVNMISRMLINRFTMRHKKATYEPDVDLGSGTRNIGIESEIPNLDIIGKRIEKIKQEHETSWHYDQDHPYKTWAYHGSYETKQTGSASSMVNGVVRLLTKPWDVVPMVTQMAMTDTTPFGQQRVFKEKVDTRTQEPKEGTKKLMKITAEWLWKELGKKKTPRMCTREEFTRKVRSNAALGAIFTDENKWKSAREAVEDSRFWELVDKERNLHLEGKCETCVYNMMGKREKKLGEFGKAKGSRAIWYMWLGARFLEFEALGFLNEDHWFSRENSLSGVEGEGLHKLGYILRDVSKKEGGAMYADDTAGWDTRITLEDLKNEEMVTNHMEGEHKKLAEAIFKLTYQNKVVRVQRPTPRGTVMDIISRRDQRGSGQVGTYGLNTFTNMEAQLIRQMEGEGVFKSIQHLTVTEEIAVQNWLARVGRERLSRMAISGDDCVVKPLDDRFASALTALNDMGKVRKDIQQWEPSRGWNDWTQVPFCSHHFHELIMKDGRVLVVPCRNQDELIGRARISQGAGWSLRETACLGKSYAQMWSLMYFHRRDLRLAANAICSAVPSHWVPTSRTTWSIHAKHEWMTAEDMLTVWNRVWIQENPWMEDKTPVESWEEIPYLGKREDQWCGSLIGLTSRATWAKNIQTAINQVRSLIGNEEYTDYMPSMKRFRREEEEAGVLW

>DENV2_AEH59346_USA

MNNQRKKARNTPFNMLKRERNRVSTVQQLTKRFSLGMLQGRGPLKLFMALVAFLRFLTIPPTAGILKRWGTIKKSKAINVLRGFRKEIGRMLNILNRRRRTAGMIIMLIPTVMAFHLTTRNGEPHMIVSRQEKGKSLLFKTGDGVNMCTLMAMDLGELCEDTITYKCPLLRQNEPEDIDCWCNSTSTWVTYGTCTTTGEHRREKRSVALVPHVGMGLETRTETWMSSEGAWKHAQRIETWILRHPGFTIMAAILAYTIGTTHFQRALIFILLTAVAPSMTMRCIGISNRDFVEGVSGGSWVDIVLEHGSCVTTMAKNKPTLDFELIKTEAKQSATLRKYCIEAKLTNTTTESRCPTQGEPSLNEEQDKRFVCKHSMVDRGWGNGCGLFGKGGIVTCAMFTCKKNMKGKVVQPENLEYTIVITPHSGEEHAVGNDTGKHGKEIKITPQSSITEAELTGYGTVTMECSPRTGLDFNEMVLLQMENKAWLVHRQWFLDLPLPWLPGADTQGSNWIQKETLVTFKNPHAKKQDVVVLGSQEGAMHTALTGATEIQMSSGNLLFTGHLKCRLRMDKLQLKGMSYSMCTGKFKVVKEIAETQHGTIVIRVQYEGDGSPCKIPFEIMDLEKRHVLGRLITVNPIVTEKDSPVNIEAEPPFGDSYIIIGVEPGQLKLNWFKKGSSIGQMLETTMRGAKRMAILGDTAWDFGSLGGVFTSIGKALHQVFGAIYGAAFSGVSWTMKILIGVIITWIGMNSRSTSLSVSLVLVGVVTLYLGVMVQADSGCVVSWKNKELKCGSGIFITDNVHTWTEQYKFQPESPSKLASAIQKAHEEGICGIRSVTRLENLMWKQITPELNHILSENEVKLTIMTGDIKGIMQAGKRSLRPQPTELKYSWKTWGKAKMLSTESHNQTFLIDGPETAECPNTNRAWNSLEVEDYGFGVFTTNIWLKLREKQDVFCDSKLMSAAIKDNRAVHADMGYWIESALNDTWKIEKASFIEVKSCHWPKSHTLWSNEVLESEMIIPKNFAGPVSQHNYRPGYHTQTAGPWHLGKLEMDFDFCEGTTVVVTEDCGNRGPSLRTTTASGKLITEWCCRSCTLPPLRYRGEDGCWYGMEIRPLKEKEENLVNSLVTAGHGQIDNFSLGVLGMALFLEEMLRTRVGTKHAILLVAVSFVTLITGNMSFRDLGRVMVMVGATMTDDIGMGVTYLALLAAFKVRPTFAAGLLLRKLTSKELMMTTIGIVLLSQSTIPETILELTDALALGMMVLKMVRKMEKYQLAVTIMAILCVPNAVILQNAWKVSCTILAVVSVSPLFLTSSQQKADWIPLALTIKGLNPTAIFLTTLSRTNKKRSWPLNEAIMAVGMVSILASSLLKNDIPMTGPLVAGGLLTVCYVLTGRSADLELERAADVKWEDQAEISGSSPILSITISEDGSMSIKNEEEEQTLTILIRTGLLVISGLFPVSLPITAAAWYLWEVKKQRAGVLWDVPSPPPVGKAELEDGAYRIKQKGILGYSQIGAGVYKEGTFHTMWHVTRGAVLMHKGKRIEPSWADVKKDLISYGGGWKLEGEWKEGEEVQVLALEPGKNPRAVQTKPGLFKTNAGTIGAVSLDFSPGTSGSPIIDKKGKVVGLYGNGVVTRSGAYVSAIAQTEKSIEDNPEIEDDIFRKRKLTIMDLHPGAGKTKRYLPAIVREAIKRGLRTLILAPTRVVAAEMEEALRGLPIRYQTPAIRAEHTGREIVDLMCHATFTMRLLSPVRVPNYNLIIMDEAHFTDPASIAARGYISTRVEMGEAAGIFMTATPPGSRDPFPQSNAPIMDEEREIPERSWSSGHEWVTDFKGKTVWFVPSIKAGNDIAACLRKNGKKVIQLSRKTFDSEYVKTRTNDWDFVVTTDISEMGANFKAERVIDPRRCMKPVILTDGEERVILAGPMPVTHSSAAQRRGRIGRNPKNENDQYIYMGEPLENDEDCAHWKEAKMLLDNINTPEGIIPSMFEPEREKVDAIDGEYRLRGEARKTFVDLMRRGDLPVWLAYRVAAEGINYADRRWCFDGIKNNQILEENVEVEIWTKEGERKKLKPRWLDARIYSDPLALKEFKEFAAGRKSLTLSLITEMGRLPTFMTQKARDALDNLAVLHTAEAGGRAYNHALSELPETLETLLLLTLLATVTGGIFLFLMSGRGIGKMTLGMCCIITASILLWYAQIQPHWIAASIILEFFLIVLLIPEPEKQRTPQDNQLTYVVIAILTVVAATMANEMGFLEKTKKDLGLGSITTQQPESNILDIDLRPASAWTLYAVATTFVTPMLRHSIENSSVNVSLTAIANQATVLMGLGKGWPLSKMDIGVPLLAIGCYSQVNPITLTAALFLLVAHYAIIGPGLQAKATREAQKRAAAGIMKNPTVDGITVIDLDPIPYDPKFEKQLGQVMLLVLCVTQVLMMRTTWALCEALTLATGPISTLWEGNPGRFWNTTIAVSMANIFRGSYLAGAGLLFSIMKNTTNTRRGTGNIGETLGEKWKSRLNALGKSEFQIYKKSGIQEVDRTLAKEGIKRGETDHHAVSRGSAKLRWFVERNMVTPEGKVVDLGCGRGGWSYYCGGLKNVREVKGLTKGGPGHEEPIPMSTYGWNLVRLQSGVDVFFTPPEKCDTLLCDIGESSPNPTVEAGRTLRVLNLVENWLNNNTQFCIKVLNPYMPSVIEKMEALQRKYGGALVRNPLSRNSTHEMYWVSNASGNIVSSVNMISRMLINRFTMRHKKATYEPDVDLGSGTRNIGIESEIPNLDIIGKRIEKIKQEHETSWHYDQDHPYKTWAYHGSYETKQTGSASSMVNGVVRLLTKPWDVVPMVTQMAMTDTTPFGQQRVFKEKVDTRTQEPKEGTKKLMKITAEWLWKELGKKKTPRMCTREEFTRKVRSNAALGAIFTDENKWKSAREAVEDSRFWELVDKERNLHLEGKCETCVYNMMGKREKKLGEFGKAKGSRAIWYMWLGARFLEFEALGFLNEDHWFSRENSLSGVEGEGLHKLGYILRDVSKKEGGAMYADDTAGWDTRITLEDLKNEEMVTNHMEGEHKKLAEAIFKLTYQNKVVRVQRPTPRGTVMDIISRRDQRGSGQVGTYGLNTFTNMEAQLIRQMEGEGVFKSIQHLTVTEEIAVQNWLARVGRERLSRMAISGDDCVVKPLDDRFASALTALNDMGKVRKDIQQWEPSRGWNDWTQVPFCSHHFHELIMKDGRVLVVPCRNQDELIGRARISQGAGWSLRETACLGKSYAQMWSLMYFHRRDLRLAANAICSAVPSHWVPTSRTTWSIHAKHEWMTAEDMLTVWNRVWIQENPWMEDKTPVESWEEIPYLGKREDQWCGSLIGLTSRATWAKNIQTAINQVRSLIGNEEYTDYMPSMKRFRREEEEAGVLW

>DENV2_AEH59342_USA

MNNQRKKARNTPFNMLKRERNRVSTVQQLTKRFSLGMLQGRGPLKLFMALVAFLRFLTIPPTAGILKRWGTIKKSKAINVLRGFRKEIGRMLNILNRRRRTAGMIFMLIPTVMAFHLTTRNGEPHMIVSRQEKGKSLLFKTGDGVNMCTLMAMDLGELCEDTITYKCPLLRQNEPEDIDCWCNSTSTWVTYGTCTTTGEHRREKRSVALVPHVGMGLETRTETWMSSEGAWKHAQRIETWILRHPGFTIMAAILAYTIGTTHFQRALIFILLTAVAPSMTMRCIGISNRDFVEGVSGGSWVDIVLEHGSCVTTMAKNKPTLDFELIKTEAKQSATLRKYCIEAKLTNTTTESRCPTQGEPSLNEEQDKRFVCKHSMVDRGWGNGCGLFGKGGIVTCAMFTCIKDMKGEVVQPENLEYTIVITPHSGEEHAVGNDTGKHGKEIKITPQSSITEAELTGYGTVTMECSPRTGLDFNEMVLLQMENKAWLVHRQWFLDLPLPWLPGADTQGSNWIQKETLVTFKNPHAKKQDVVVLGSQEGAMHTALTGATEIQMSSGNLLFTGHLKCRLRMDKLQLKGMSYSMCTGKFKVVKEIAETQHGTIVIRVQYEGDGSPCKIPFEIMDLEKRHVLGRLITVNPIVTEKDSPVNIEAEPPFGDSYIIIGVEPGQLKLNWFKKGSSIGQMLETTMRGAKRMAILGDTAWDFGSLGGVFTSIGKALHQVFGAIYGAAFSGVSWTMKILIGVIITWIGMNSRSTSLSVSLVLVGVVTLYLGVMVQADSGCVVSWKNKELKCGSGIFITDNVHTWTEQYKFQPESPSKLASAIQKAHEEGICGIRSVTRLENLMWKQITPELNHILSENEVKLTIMTGDIKGIMQAGKRSLRPQPTELKYSWKTWGKAKMLSTESHNQTFLIDGPETAECPNTNRAWNSLEVEDYGFGVFTTNIWLKLREKQDVFCDSKLMSAAIKDNRAVHADMGYWIESALNDTWKIEKASFIEVKSCHWPKPHTLWSNEVLESEMIIPKNFAGPVSQHNYRPGYHTQTAGPWHLGKLEMDFDFCEGTTVVVTEDCGNRGPSLRTTTASGKLITEWCCRSCTLPPLRYRGEDGCWYGMEIRPLKEKEENLVNSLVTAGHGQIDNFSLGVLGMALFLEEMLRTRVGTKHAILLVAVSFVTLITGNMSFRDLGRVMVMVGATMTDDIGMGVTYLALLAAFKVRPTFAAGLLLRKLTSKELMMTTIGIVLLSQSTIPETILELTDALALGMMVLKMVRKMEKYQLAVTIMAILCVPNAVILQNAWKVSCTILAVVSVSPLFLTSSQQKADWIPLALTIKGLNPTAIFLTTLSRTNKKRSWPLNEAIMAVGMVSILASSLLKNDIPMTGPLVAGGLLTVCYVLTGRSADLELERAADVKWEDQAEISGSSPILSITISEDGSMSIKNEEEEQTLTILIRTGLLVISGLFPVSLPITAAAWYLWEVKKQRAGVLWDVPSPPPVGKAELEDGAYRIKQKGILGYSQIGAGVYKEGTFHTMWHVTRGAVLMHKGKRIEPSWADVKKDLISYGGGWKLEGEWKEGEEVQVLALEPGKNPRAVQTKPGLFKTNAGTIGAVSLDFSPGTSGSPIIDKKGKVVGLYGNGVVTRSGAYVSAIAQTEKSIEDNPEIEDDIFRKRKLTIMDLHPGAGKTKRYLPAIVREAIKRGLRTLILAPTRVVAAEMEEALRGLPIRYQTPAIRAEHTGREIVDLMCHATFTMRLLSPVRVPNYNLIIMDEAHFTDPASIAARGYISTRVEMGEAAGIFMTATPPGSRDPFPQSNAPIMDEEREIPERSWSSGHEWVTDFKGKTVWFVPSIKAGNDIAACLRKNGKKVIQLSRKTFDSEYVKTRTNDWDFVVTTDISEMGANFKAERVIDPRRCMKPVILTDGEERVILAGPMPVTHSSAAQRRGRIGRNPKNENDQYIYMGEPLENDEDCAHWKEAKMLLDNINTPEGIIPSMFEPEREKVDAIDGEYRLRGEARKTFVDLMRRGDLPVWLAYRVAAEGINYADRRWCFDGIKNNQILEENVEVEIWTKEGERKKLKPRWLDARIYSDPLALKEFKEFAAGRKSLTLSLITEMGRLPTFMTQKARDALDNLAVLHTAEAGGRAYNHALSELPETLETLLLLTLLATVTGGIFLFLMSGRGIGKMTLGMCCIITASILLWYAQIQPHWIAASIILEFFLIVLLIPEPEKQRTPQDNQLTYVVIAILTVVAATMANEMGFLEKTKKDLGLGSITTQQPESNILDIDLRPASAWTLYAVATTFVTPMLRHSIENSSVNVSLTAIANQATVLMGLGKGWPLSKMDIGVPLLAIGCYSQVNPITLTAALFLLAAHYAIIGPGLQAKATREAQKRAAAGIMKNPTVDGITVIDLDPIPYDPKFEKQLGQVMLLVLCVTQVLMMRTTWALCEALTLATGPISTLWEGNPGRFWNTTIAVSMANIFRGSYLAGAGLLFSIMKNTTNTRRGTGNIGETLGEKWKSRLNALGKSEFQIYKKSGIQEVDRTLAKEGIKRGETDHHAVSRGSAKLRWFVERNMVTPEGKVVDLGCGRGGWSYYCGGLKNVREVKGLTKGGPGHEEPIPMSTYGWNLVRLQSGVDVFFTPPEKCDTLLCDIGESSPNPTVEAGRTLRVLNLVENWLNNNTQFCIKVLNPYMPSVIEKMEALQRKYGGALVRNPLSRNSTHEMYWVSNASGNIVSSVNMISRMLINRFTMRHKKATYEPDVDLGSGTRNIGIESEIPNLDIIGKRIEKIKQEHETSWHYDQDHPYKTWAYHGSYETKQTGSASSMVNGVVRLLTKPWDVVPMVTQMAMTDTTPFGQQRVFKEKVDTRTQEPKEGTKKLMKITAEWLWKELGKKKTPRMCTREEFTRKVRSNAALGAIFTDENKWKSAREAVEDSRFWELVDKERNLHLEGKCETCVYNMMGKREKKLGEFGKAKGSRAIWYMWLGARFLEFEALGFLNEDHWFSRENSLSGVEGEGLHKLGYILRDVSKKEGGAMYADDTAGWDTRITLEDLKNEEMVTNHMEGEHKKLAEAIFKLTYQNKVVRVQRPTPRGTVMDIISRRDQRGSGQVGTYGLNTFTNMEAQLIRQMEGEGVFKSIQHLTVTEEIAVQNWLARVGRERLSRMAISGDDCVVKPLDDRFASALTALNDMGKVRKDIQQWEPSRGWNDWTQVPFCSHHFHELIMKDGRVLVVPCRNQDELIGRARISQGAGWSLRETACLGKSYAQMWSLMYFHRRDLRLAANAICSAVPSHWVPTSRTTWSIHAKHEWMTAEDMLTVWNRVWIQENPWMEDKTPVESWEEIPYLGKREDQWCGSLIGLTSRATWAKNIQTAINQVRSLIGNEEYTDYMPSMKRFRREEEEAGVLW

>DENV2_ACS32039_Sri_Lanka

MNNQRKKARNTPFNMLKRERNRVSTVQQLTKRFSLGMLQGRGPLKLFMALVAFLRFLTIPPTAGILKRWGTIKKSKAINVLRGFRKEIGRMLNILNRRRRTAGVIIMLIPTAMAFHLTTRNGEPHMIVSKQEKGKSLLFKTEEGVNMCTLMAMDLGELCEDTITYNCPLLRQNEPEDIDCWCNSTSTWVTYGTCTATGEHRREKRSVALVPHVGMGLETRTETWMSSEGAWKHAQRIETWILRHPGFTIMAAILAYTIGTTYFQRVLIFILLTAVAPSMTMRCIGISNRDFVEGVSGGSWVDIVLEHGSCVTTMAKNKPTLDFELIKTEAKQPATLRKYCIEAKLTNTTTASRCPTQGEPSLNEEQDKRFVCKHSMVDRGWGNGCGLFGKGGIVTCAMFTCKKNMEGKIVQPENLEYTIVVTPHSGEENAVGNDTGKHGKEIKVTPQSSITEAELTGYGTVTMECSPRTGLDFNEMVLLQMENKAWLVHRQWFLDLPLPWLPGADIQGSNWIQKETLVTFKNPHAKKQDVVVLGSQEGAMHTALTGATEIQMSSGNLLFTGHLKCRLRMDKLQLKGMSYSMCTGKFKVVKEIAETQHGTIVVRVQYEGDGSPCKIPFEIMDLEKRHVLGRLITVNPIVTEKDSPVNIEAEPPFGDSYIIIGVEPGQLKLSWFKKGSSIGQMFETTMRGAKRMAILGDTAWDFGSLGGVFTSIGKALHQVFGAIYGAAFSGVSWTMKILIGVVITWIGMNSRSTSLSVSLVLVGVVTLYLGVMVQADSGCVVSWKNKELKCGSGIFITDNVHTWTEQYKFQPESPSKLASAIQKAHEEGICGIRSVTRLENLMWKQITPELNHILSENEVKLSIMTGDIKGIMQAGKRSLRPQPTELKYSWKTWGKAKMLSTEPHNQTFLIDGPETAECPNTNRAWNSLEVEDYGFGVFTTNIWLKLKERQDVFCDSKLMSAAIKDNRAVHADMGYWIESALNDTWKIEKASFIEVKSCHWPKSHTLWSNGVLESEMIIPKNFAGPVSQHNYRPGYHTQTAGPWHLGKLEMDFDFCEGTTVVVTEDCGNRGPSLRTTTASGKLITEWCCRSCTLPPLRYRGEDGCWYGMEIRPLKEKEENLVNSLVTAGHGQIDNFSLGVLGMALFLEEMLRTRVGTKHAVLLVAVSFMTLITGNMSFRDLGRVMVMVGAAMTDDIGMGVTYLALLAAFKVRPTFAAGLLLRKLTSKELMMTTIGIVLLSQSTIPETILELTDALALGMMILKIVRNMEKYQLAVTIMAILCVPNAVILQNAWKVSCTILAAVSVSPLLLTSSQQKADWIPLALTIKGLNPTAIFLTTLSRTNKKRSWPLNEAIMAVGMVSILASSLLKNDIPMTGPLVAGGLLTVCYVLTGRSADLELERAADVRWEDQAEISGSSPILSITISEDGSMSIKNEEEEQTLTILIRTGLLVISGLFPVSIPITAAAWYLWEVKKQRAGVLWDVPSPPPVGKAELEDGAYRIKQKGILGYSQIGAGVYKEGTFHTMWHVTRGAVLMHKGKRIEPSWADVRKDLISYGGGWKLEGEWKEGEEVQVLALEPGKNPRAVQTKPGLFKTNTGTIGAVSLDFSPGTSGSPIVDKKGKVVGLYGNGVVTRSGTYVSAIAQTEKSIEDNPEIEDDIFRKKRLTIMDLHPGAGKTKRYLPAIVREAIKRGLRTLILAPTRVVAAEMEEALRGLPIRYQTPAIRAEHTGREIVDLMCHATFTMRLLSPVRVPNYNLIIMDEAHFTDPASIAARGYISTRVEMGEAAGIFMTATPPGSRDPFPQSNAPIMDEEREIPERSWNSGHEWVTDFKGKTVWFVPSIKAGNDIAACLRKNGKKVIQLSRKTFDSEYIKTRTNDWDFVVTTDISEMGANFKAERVIDPRRCMKPVILTDGEERVILAGPMPVTHSSAAQRRGRIGRNPKNENDQYIYMGEPLENDEDCAHWKEAKMLLDNINTPEGIIPSMFEPEREKVDAIDGEYRLRGEARKTFVDLMRRGDLPVWLAYRVAAEGINYADRRWCFDGIKNNQILEENVEVEIWTKEGERKKLKPRWLDARIYSDPLALKEFKEFAAGRKSLTLNLITEMGRLPTFMTQKARNALDNLAVLHTAEAGGRAYNHALSELPETLETLLLLTLLATVTGGIFLFLMSGKGIGKMTLGMCCIITASILLWYAQIQPHWIAASIILEFFLIVLLIPEPEKQRTPQDNQLTYVVIAILTVVAATMANEMGFLEKTKKDFGLGSIATQQPESNILDIDLRPASAWTLYAVATTFITPMLRHSIENSSVNVSLTAIANQATVLMGLGKGWPLSKMDIGVPLLAIGCYSQVNPITLTAALLLLVAHYAIIGPGLQAKATREAQKRAAAGIMKNPTVDGITVIDLDPIPYDPKFEKQLGQVMLLVLCVTQVLMMRTTWALCEALTLATGPISTLWEGNPGRFWNTTIAVSMANIFRGSYLAGAGLLFSIMKNTTNTRRGTGNTGETLGEKWKNRLNSLGKSEFQIYKRSGIQEVDRTLAKEGIKRGETDHHAVSRGSAKLRWFVERNLVTPEGKVVDLGCGRGGWSYYCGGLKNVREVKGLTKGGPGHEEPIPMSTYGWNLVRLQSGVDVFFTPPEKCDTLLCDIGESSPNPTVEAGRTLRVLNLVENWLNNNTQFCIKVLNPYMPSVIEKMETLQRKYGGALVRNPLSRNSTHEMYWVSNASGNIVSSVNMISRMLINRFTMRHKKATYEPDVDLGSGTRNIGIESETPNLDIIGKRIEKIKQEHETSWHYDQDHPYKTWAYHGSYETKQTGSASSMVNGVVRLLTKPWDVIPMVTQMAMTDTTPFGQQRVFKEKVDTRTQEPKEGTKKLMKITAEWLWKELRKKKTPRMCTREEFARKVRSNAALGAIFTDENKWKSAREAVEDSRFWELVDKERNLHLEGKCETCVYNMMGKREKKIGEFGKAKGSRAIWYMWLGARFLEFEALGFLNEDHWFSRENSLSGVEGEGLHKLGYILREVSKKEGGAMYADDTAGWDTRITLEDLKNEEMVTNHMEGEHKKLAEAIFKLTYQNKVVRVQRPTPRGTVMDIISRRDQRGSGQVGTYGLNTFTNMGAQLIRQMEGEGVFKGIQHLTATEEVAVQDWLARVGRERLSRMAISGDDCVVKPLDDRFASALTALNDMGKVRKDIQQWEPSRGWNDWTQVPFCSHHFHELIMKDGRVLVVPCRNQDELIGRARISQGAGWSLRETACLGKSYAQMWSLMYFHRRDLRLAANAICSAVPSHWVPTSRTTWSIHARHEWMTTEDMLTVWNRVWIQENPWMEDKTPVESWEEIPYLGKREDQWCGSLIGLTSRATWAKNIQTAINQVRSLIGSEEYTDYMPSMKRFRREEEEAGVLW

>DENV2_ACS32038_Sri_Lanka

MNNQRKKARNTPFNMLKRERNRVSTVQQLTKRFSLGMLQGRGPLKLFMALVAFLRFLTIPPTAGILKRWGTIKKSKAINVLRGFRKEIGRMLNILNRRRRTAGVIIMLIPTAMAFHLTTRNGEPHMIVSKQEKGKSLLFKTEEGVNMCTLMAMDLGELCEDTITYNCPLLRQNEPEDIDCWCNSTSTWVTYGTCTATGEHRREKRSVALVPHVGMGLETRTETWMSSEGAWKHAQRIETWILRHPGFTIMAAILAYTIGTTYFQRVLIFILLTAVAPSMTMRCIGISNRDFVEGVSGGSWVDIVLEHGSCVTTMAKNKPTLDFELIKTEAKQPATLRKYCIEAKLTNTTTASRCPTQGEPSLNEEQDKRFVCKHSMVDRGWGNGCGLFGKGGIVTCAMFTCKKNMEGKIVQPENLEYTIVVTPHSGEENAVGNDTGKHGKEIKVTPQSSITEAELTGYGTVTMECSPRTGLDFNEMVLLQMENKAWLVHRQWFLDLPLPWLPGADIQGSNWIQKETLVTFKNPHAKKQDVVVLGSQEGAMHTALTGATEIQMSSGNLLFTGHLKCRLRMDKLQLKGMSYSMCTGKFKVVKEIAETQHGTIVVRVQYEGDGSPCKIPFEIMDLEKRHVLGRLITVNPIVTEKDSPVNIEAEPPFGDSYIIIGVEPGQLKLSWFKKGSSIGQMFETTMRGAKRMAILGDTAWDFGSLGGVFTSIGKALHQVFGAIYGAAFSGVSWTMKILIGVVITWIGMNSRSTSLSVSLVLVGVVTLYLGVMVQADSGCVVSWKNKELKCGSGIFITDNVHTWTEQYKFQPESPSKLASAIQKAHEEGICGIRSVTRLENLMWKQITPELNHILSENEVKLSIMTGDIKGIMQAGKRSLRPQPTELKYSWKTWGKAKMLSTEPHNQTFLIDGPETAECPNTNRAWNSLEVEDYGFGVFTTNIWLKLKERQDVFCDSKLMSAAIKDNRAVHADMGYWIESALNDTWKIEKASFIEVKSCHWPKSHTLWSNGVLESEMIIPKNFAGPVSQHNYRPGYHTQTAGPWHLGKLEMDFDFCEGTTVVVTEDCGNRGPSLRTTTASGKLITEWCCRSCTLPPLRYRGEDGCWYGMEIRPLKEKEENLVNSLVTAGHGQIDNFSLGVLGMALFLEEMLRTRVGTKHAVLLVAVSFMTLITGNMSFRDLGRVMVMVGAAMTDDIGMGVTYLALLAAFKVRPTFAAGLLLRKLTSKELMMTTIGIVLLSQSTIPETILELTDALALGMMILKIVRNMEKYQLAVTIMAILCVPNAVILQNAWKVSCTILAAVSVSPLLLTSSQQKADWIPLALTIKGLNPTAIFLTTLSRTNKKRSWPLNEAIMAVGMVSILASSLLKNDIPMTGPLVAGGLLTVCYVLTGRSADLELERAADVRWEDQAEISGSSPILSITISEDGSMSIKNEEEEQTLTILIRTGLLVISGLFPVSIPITAAAWYLWEVKKQRAGVLWDVPSPPPVGKAELEDGAYRIKQKGILGYSQIGAGVYKEGTFHTMWHVTRGAVLMHKGKRIEPSWADVRKDLISYGGGWKLEGEWKEGEEVQVLALEPGKNPRAVQTKPGLFKTNTGTIGAVSLDFSPGTSGSPIVDKKGKVVGLYGNGVVTRSGTYVSAIAQTEKSIEDNPEIEDDIFRKKRLTIMDLHPGAGKTKRYLPAIVREAIKRGLRTLILAPTRVVAAEMEEALRGLPIRYQTPAIRAEHTGREIVDLMCHATFTMRLLSPVRVPNYNLIIMDEAHFTDPASIAARGYISTRVEMGEAAGIFMTATPPGSRDPFPQSNAPIMDEEREIPERSWNSGHEWVTDFKGKTVWFVPSIKAGNDIAACLRKNGKKVIQLSRKTFDSEYIKTRTNDWDFVVTTDISEMGANFKAERVIDPRRCMKPVILTDGEERVILAGPMPVTHSSAAQRRGRIGRNPKNENDQYIYMGEPLENDEDCAHWKEAKMLLDNINTPEGIIPSMFEPEREKVDAIDGEYRLRGEARKTFVDLMRRGDLPVWLAYRVAAEGINYADRRWCFDGIKNNQILEENVEVEIWTKEGERKKLKPRWLDARIYSDPLALKEFKEFAAGRKSLTLNLITEMGRLPTFMTQKARNALDNLAVLHTAEAGGRAYNHALSELPETLETLLLLTLLATVTGGIFLFLMSGKGIGKMTLGMCCIITASILLWYAQIQPHWIAASIILEFFLIVLLIPEPEKQRTPQDNQLTYVVIAILTVVAATMANEMGFLEKTKKDFGLGSIATQQPESNILDIDLRPASAWTLYAVATTFITPMLRHSIENSSVNVSLTAIANQATVLMGLGKGWPLSKMDIGVPLLAIGCYSQVNPITLTAALLLLVAHYAIIGPGLQAKATREAQKRAAAGIMKNPTVDGITVIDLDPIPYDPKFEKQLGQVMLLVLCVTQVLMMRTTWALCEALTLATGPISTLWEGNPGRFWNTTIAVSMANIFRGSYLAGAGLLFSIMKNTTNTRRGTGNTGETLGEKWKNRLNSLGKSEFQIYKRSGIQEVDRTLAKEGIKRGETDHHAVSRGSAKLRWFVERNLVTPEGKVVDLGCGRGGWSYYCGGLKNVREVKGLTKGGPGHEEPIPMSTYGWNLVRLQSGVDVFFTPPEKCDTLLCDIGESSPNPTVEAGRTLRVLNLVENWLNNNTQFCIKVLNPYMPSVIEKMETLQRKYGGALVRNPLSRNSTHEMYWVSNASGNIVSSVNMISRMLINRFTMRHKKATYEPDVDLGSGTRNIGIESETPNLDIIGKRIEKIKQEHETSWHYDQDHPYKTWAYHGSYETKQTGSASSMVNGVVRLLTKPWDVIPMVTQMAMTDTTPFGQQRVFKEKVDTRTQEPKEGTKKLMKITAEWLWKELGKKKTPRMCTREEFARKVRSNAALGAIFTDENKWKSAREAVEDSRFWELVDKERNLHLEGKCETCVYNMMGKREKKIGEFGKAKGSRAIWYMWLGARFLEFEALGFLNEDHWFSRENSLSGVEGEGLHKLGYILREVSKKEGGAMYADDTAGWDTRITLEDLKNEEMVTNHMEGEHKKLAEAIFKLTYQNKVVRVQRPTPRGTVMDIISRRDQRGSGQVGTYGLNTFTNMGAQLIRQMEGEGVFKGIQHLTATEEVAVQDWLARVGRERLSRMAISGDDCVVKPLDDRFASALTALNDMGKVRKDIQQWEPSRGWNDWTQVPFCSHHFHELIMKDGRVLVVPCRNQDELIGRARISQGAGWSLRETACLGKSYAQMWSLMYFHRRDLRLAANAICSAVPSHWVPTSRTTWSIHARHEWMTTEDMLTVWNRVWIQENPWMEDKTPVESWEEIPYLGKREDQWCGSLIGLTSRATWAKNIQTAINQVRSLIGSEEYTDYMPSMKRFRREEEEAGVLW

>DENV2_ACQ44517__Papua_New_Guinea

MNNQRKKARNTPFNMLKRERNRVSTVQQLTKRFSLGMLQGRGPLKLFMALVAFLRFLTIPPTAGILKRWGTIKKSKAINVLRGFRKEIGRMLNILNRRRRTAGMIIMLIPTVMAFHLTTRNGEPHMIVSRQEKGKSLLFKTEDGVNMCTLMAMDLGELCEDTITYKCPFLKQNEPEDIDCWCNSTSTWVTYGTCTTTGEHRREKRSVALVPHVGMGLETRTETWMSSEGAWKHAQRIETWILRHPGFTIMAAILAYTIGTTHFQRALIFILLTAVAPSMTMRCIGISNRDFVEGVSGGSWVDIVLEHGSCVTTMAKNKPTLDFELIKTEAKQPATLRKYCIEAKLTNTTTDSRCPTQGEPSLNEEQDKRFVCKHSMVDRGWGNGCGLFGKGGIVTCAMFTCKKNMKGKVVQPENLEYTIVITPHSGEEHAVGNDTGKHGKEIKITPQSSITEAELTGYGTVTMECSPRTGLDFNEMVLLQMENKAWLVHRQWFLDLPLPWLPGADTQGSNWIQKETLVTFKNPHAKKQDVVVLGSQEGAMHTALTGATEIQMSSGNLLFTGHLKCRLRMDKLQLKGMSYSMCTGKFKVVKEIAETQHGTIVIRVQYEGDGSPCKIPFEIMDLEKRHVLGRLITVNPIVTEKDSPVNIEAEPPFGDSYIIIGVEPGQLKLNWFKKGSSIGQMIETTMRGAKRMAILGDTAWDFGSLGGVFTSIGKALHQVFGAIYGAAFSGVSWIMKILIGVIITWIGMNSRSTSLSVSLVLVGVVTLYLGVMVQADSGCVVSWKNKELKCGSGIFITDNVHTWTEQYKFQPESPSKLASAIQKAHEEGICGIRSVTRLENLMWKQITPELNHILSENEVKLTIMTGDIKGIMQAGKRSLQPQPTELKYSWKTWGKAKMLSTESHNQTFLIDGPETAECPNTNRAWNSLEVEDYGFGVFTTNIWLKLREKQDVFCDSKLMSAAIKDNRAVHADMGYWIESALNDTWKIEKASFIEVKSCHWPKSHTLWSNGVLESEMIIPKNFAGPVSQHNYRPGYHTQTAGPWHLGKLEMDFDFCEGTTVVVTEDCGNRGPSLRTTTASGKLITEWCCRSCTLPPLRYRGEDGCWYGMEIRPLKEKEENLVNSLVTAGHGQIDNFSLGVLGMALFLEEMLRTRVGTKHAILLVAVSFVTLITGNMSFRDLGRVMVMVGATMTDDIGMGVTYLALLAAFKVRPTFAAGLLLRKLTSKELMMTTIGIVLLSQSTIPETILELTDALALGMMVLKMVRKMEKYQLAVTIMAILCVPNAVILQNAWKVSCTILAVVSVSPLFLTSSQQKADWIPLALTIKGLNPTAIFLTTLSRTNKKRSWPLNEAIMAVGMVSILASSLLKNDIPMTGPLVAGGLLTVCYVLTGRSADLELERAADVKWEDQAEISGSSPILSITISEDGSMSIKNEEEEQTLTILIRTGLLVISGLFPVSIPITAAAWYLWEVKKQRAGVLWDVPSPPPVGKAELEDGAYRIKQKGILGYSQIGAGVYKEGTFHTMWHVTRGAVLMHKGKRIEPSWADVKKDLISYGGGWKLEGEWKEGEEVQVLALEPGKNPRAVQTKPGLFKTNAGTIGAVSLDFSPGTSGSPIIDKKGKVVGLYGNGVVTRSGAYVSAIAQTEKSIEDNPEIEDDIFRKRKLTIMDLHPGAGKTKRYLPAIVREAIKRGLRTLILAPTRVVAAEMEEALRGLPIRYQTPAIRAEHTGREIVDLMCHATFTMRLLSPVRVPNYNLIIMDEAHFTDPASIAARGYISTRVEMGEAAGIFMTATPPGSRDPFPQSNAPIMDEEREIPERSWSSGHEWVTDFKGKTVWFVPSIKAGNDIAACLRKNGKKVIQLSRKTFDSEYVKTRTNDWDFVVTTDISEMGANFKAERVIDPRRCMKPVILTDGEERVILAGPMPVTHSSAAQRRGRIGRNPKNENDQYIYMGEPLENDEDCAHWKEAKMLLDNINTPEGIIPSMFEPEREKVDAIDGEYRLRGEARKTFVDLMRRGDLPVWLAYRVAAEGINYADRRWCFDGIKNNQILEENVEVEIWTKEGERKKLKPRWLDARIYSDPLALKEFKEFAAGRKSLTLNLITEMGRLPTFMTQKARDALDNLAVLHTAEAGGRAYNHALSELPETLETLLLLTLLATVTGGIFLFLMSGRGIGKMTLGMCCIITASILLWYAQIQPHWIAASIILEFFLIVLLIPEPEKQRTPQDNQLTYVVIAILTVVAATMANEMGFLEKTKKDLGLGSITTQQPESNILDIDLRPASAWTLYAVATTFVTPMLRHSIENSSVNVSLTAIANQATVLMGLGKGWPLSKMDIGVPLLAIGCYSQVNPITLTAALFLLVAHYAIIGPGLQAKATREAQKRAAAGIMKNPTVDGITVIDLDPIPYDPKFEKQLGQVMLLVLCVTQVLMMRTTWALCEALTLATGPISTLWEGNPGRFWNTTIAVSMANIFRGSYLAGAGLLFSIMKNTTNTRRGTGNIGETLGEKWKSRLNALGKSEFQIYKKSGIQEVDRTLAKEGIKRGETDHHAVSRGSAKLRWFVERNMVTPEGKVVDLGCGRGGWSYYCGGLKNVREVKGLTKGGPGHEEPIPMSTYGWNLVRLQSGVDVFFTPPEKCDTLLCDIGESSPNPTVEAGRTLRVLNLVENWLNNNTQFCIKVLNPYMPSVIEKMEALQRKYGGALVRNPLSRNSTHEMYWVSNASGNIVSSVNMISRMLINRFTMRHKKATYEPDVDLGSGTRNIGIESEIPNLDIIGKRIEKIKQEHETSWHYDQDHPYKTWAYHGSYETKQTGSASSMVNGVVRLLTKPWDVVPMVTQMAMTDTTPFGQQRVFKEKVDTRTQEPKEGTKKLMKITAEWLWKELGKKKTPRMCTREEFTRKVRSNAALGAIFTDENKWKSAREAVEDSRFWELVDKERNLHLEGKCETCVYNMMGKREKKLGEFGKAKGSRAIWYMWLGARFLEFEALGFLNEDHWFSRENSLSGVEGEGLHKLGYILRDVSKKEGGAMYADDTAGWDTRITLEDLKNEEMVTNHMEGEHKKLAEAIFKLTYQNKVVRVQRPTPRGTVMDIISRRDQRGSGQVGTYGLNTFTNMEAQLIRQMEGEGVFKSIQHLTVTEEIAVQNWLARVGRERLSRMAISGDDCVVKPLDDRFASALTALNDMGKVRKDIQQWEPSRGWNDWTQVPFCSHHFHELIMKDGRVLVVPCRNQDELIGRARISQGAGWSLRETACLGKSYAQMWSLMYFHRRDLRLAANAICSAVPSHWVPTSRTTWSIHAKHEWMTTEDMLTVWNRVWIQENPWMEDKTPVESWEEIPYLGKREDQWCGSLIGLTSRATWAKNIQTAINQVRSLIGNEEYTDYMPSMKRFRREEEEAGVLW

>DENV2_ACQ44488_Honduras

MNNQRKKARNTPFNMLKRERNRVSTVQQLTKRFSLGMLQGRGPLKLFMALVAFLRFLTIPPTAGILKRWGTIKKSKAINVLRGFRKEIGRMLNILNRRRRTAGMIIMLIPTVMAFHLTTRNGEPHMIVSRQEKGKSLLFKTKDGTNMCTLMAMDLGELCEDTITYKCPFLKQNEPEDIDCWCNSTSTWVTYGTCTTTGEHRREKRSVALVPHVGMGLETRTETWMSSEGAWKHAQRIETWILRHPGFTIMAAILAYTIGTTHFQRVLIFILLTAIAPSMTMRCIGISNRDFVEGVSGGSWVDIVLEHGSCVTTMAKNKPTLDFELIKTEAKQPATLRKYCIEAKLTNTTTDSRCPTQGEPTLNEEQDKRFVCKHSMVDRGWGNGCGLFGKGGIVTCAMFTCKKNMEGKIVQPENLEYTVVITPHSGEEHAVGNDTGKHGKEVKITPQSSITEAELTGYGTVTMECSPRTGLDFNEMVLLQMEDKAWLVHRQWFLDLPLPWLPGADTQGSNWIQKETLVTFKNPHAKKQDVVVLGSQEGAMHTALTGATEIQMSSGNLLFTGHLKCRLRMDKLQLKGMSYSMCTGKFKIVKEIAETQHGTIVIRVQYEGDGSPCKIPFEIMDLEKRHVLGRLITVNPIVTEKDSPVNIEAEPPFGDSYIIIGVEPGQLKLDWFKKGSSIGQMFETTMRGAKRMAILGDTAWDFGSLGGVFTSIGKALHQVFGAIYGAAFSGVSWTMKILIGVIITWIGMNSRSTSLSVSLVLVGIVTLYLGVMVQADSGCVVSWKNKELKCGSGIFVTDNVHTWTEQYKFQPESPSKLASAIQKAHEEGICGIRSVTRLENLMWKQITSELNHILSENEVKLTIMTGDIKGIMQVGKRSLRPQPTELRYSWKTWGKAKMLSTELHNQTFLIDGPETAECPNTNRAWNSLEVEDYGFGVFTTNIWLRLREKQDVFCDSKLMSAAIKDNRAVHADMGYWIESALNDTWKIEKASFIEVKSCHWPKSHTLWSNGVLESEMIIPKNFAGPVSQHNNRPGYHTQTAGPWHLGKLEMDFDFCEGTTVVVTEECGNRGPSLRTTTASGKLITEWCCRSCTLPPLRYRGEDGCWYGMEIRPLKEKEENLVSSLVTAGHGQIDNFSLGILGMALFLEEMLRTRVGTKHAILLVAVSFVTLITGNMSFRDLGRVMVMVGATMTDDIGMGVTYLALLAAFKVRPTFAAGLLLRKLTSKELMMTTIGIVLLSQSSIPETILELTDALALGMMVLKMVRNMEKYQLAVTIMAILCVPNAVILQNAWKVSCTILAVVSVSPLLLTSSQQKADWIPLALTIKGLNPTAIFLTTLSRTSKKRSWPLNEAIMAVGMVSILASSLLKNDIPMTGPLVAGGLLTVCYVLTGRSADLELERATDVKWDDQAEISGSSPILSITISEDGSMSIKNEEEEQTLTILIRTGLLVISGLFPVSIPITAAAWYLWEVKKQRAGVLWDVPSPPPVGKAELEDGAYRIKQKGILGYSQIGAGVYKEGTFHTMWHVTRGAVLMHKGKRIEPSWADVKKDLISYGGGWKLEGEWKEGEEVQVLALEPGKNPRAVQTKPGLFRTNTGTIGAVSLDFSPGTSGSPIVDKKGKVVGLYGNGVVTRSGAYVSAIAQTEKSIEDNPEIEDDIFRKRRLTIMDLHPGAGKTKKYLPAIVREAIKRGLRTLILAPTRVVAAEMEEALRGLPIRYQTPAIRAEHTGREIVDLMCHATFTMRLLSPIRVPNYNLIIMDEAHFTDPASIAARGYISTRVEMGEAAGIFMTATPPGSRDPFPQSNAPIMDEEREIPERSWNSGHEWVTDFKGKTVWFVPSIKTGNDIAACLRKNGKRVIQLSRKTFDSEYVKTRTNDWDFVVTTDISEMGANFKAERVIDPRRCMKPVILTDGEERVILAGPMPVTHSSAAQRRGRIGRNPRNENDQYIYMGEPLENDEDCAHWKEAKMLLDNINTPEGIIPSMFEPEREKVDAIDGEYRLRGEARKTFVDLMRRGDLPVWLAYKVASEGINYADRRWCFDGTRNNQILEENVEVEIWTKEGERKKLKPRWLDARIYSDPLALKEFKEFAAGRKSLTLNLITEMGRLPTFMTQKARDALDNLAVLHTAEAGGKAYNHALSELPETLETLLLLTLLATVTGGIFLFLMSGRGIGKMTLGMCCIITASILLWYAQIQPHWIAASIILEFFLIVLLIPEPEKQRTPQDNQLTYVIIAILTVVAATMANEMGFLEKTKKDLGLGHIATQQPESNILDIDLRPASAWTLYAVATTFITPMLRHSIENSSVNVSLTAIANQATVLMGLGKGWPLSKMDIGVPLLAIGCYSQVNPITLTAALLLLVAHYAIIGPGLQAKATREAQKRAAAGIMKNPTVDGITVIDLDPIPYDPKFEKQLGQVMLLVLCVTQVLMMRTTWALCEALTLATGPVSTLWEGNPGRFWNTTIAVSMANIFRGSYLAGAGLLFSIMKNTTSTRRGTGNIGETLGEKWKSRLNALGKSEFQIYKKSGIQEVDRTLAKEGIKRGETDHHAVSRGSAKLRWFVERNLVTPEGKVVDLGCGRGGWSYYCGGLKNVREVKGLTKGGPGHEEPIPMSTYGWNLVRLQSGVDVFFVPPERCDTLLCDIGESSPNPTVEAGRTLRVLNLVENWLNNNTQFCVKVLNPYMPSVIERMETLQRKYGGALVRNPLSRNSTHEMYWVSNASGNIVSSVNMISRMLINRFTMRHKKATYEPDVDLGSGTRNIGIESETPNLDIIGKRIEKIKQEHEASWHYDQDHPYKTWAYHGSYETKQTGSASSMVNGVVRLLTKPWDVVPMVTQMAMTDTTPFGQQRVFKEKVDTRTQEPKEGTKKLMKITAEWLWKELGKEKTPRMCTREEFTKKVRSNAALGAIFTDENKWKSAREAVEDNRFWELVDKERNLHLEGKCETCVYNMMGKREKKLGEFGKAKGSRAIWYMWLGARFLEFEALGFLNEDHWFSRENSLSGVEGEGLHKLGYILREVSKKEGGAMYADDTAGWDTRITIEDLKNEEMITNHMAGEHKKLAEAIFKLTYQNKVVRVQRPTPRGTVMDIISRRDQRGSGQVGTYGLNTFTNMEAQLIRQMEGEGIFKSIQHLTASEEIAVQDWLVRVGRERLSRMAISGDDCVVKPLDDRFARALTALNDMGKVRKDIQQWEPSRGWNDWTQVPFCSHHFHELIMKDGRTLVVPCRNQDELIGRARISQGAGWSLRETACLGKSYAQMWSLMYFHRRDLRLAANAICSAVPSHWVPTSRTTWSIHASHEWMTTEDMLTVWNRVWILENPWMEDKTPVESWEEIPYLGKREDQWCGSLIGLTSRATWAKNIQTAINQVRSLIGNEEYTDYMPSMKRFRREEEEAGVLW

>DENV2_ACJ04201_Papua_New_Guinea

MNNQRKKARNTPFNMLKRERNRVSTVQQLTKRFSLGMLQGRGPLKLFMALVAFLRFLTIPPTAGILKRWGTIKKSKAINVLRGFRKEIGRMLNILNRRRRTAGMIIMLIPTVMAFHLTTRNGEPHMIVSRQEKGKSLLFKTGDGVNMCTLMAMDLGELCEDTITYKCPLLRQNEPEDIDCWCNSTSTWVTYGTCTTTGEHRREKRSVALVPHVGMGLETRTETWMSSEGAWKHAQRIETWILRHPGFTIMAAILAYTIGTTHFQRALIFILLTAVAPSMTMRCIGISNRDFVEGVSGGSWVDIVLEHGSCVTTMAKNKPTLDFELIKTEAKQSATLRKYCIEAKLTNTTTESRCPTQGEPSLNEEQDKRFVCKHSMVDRGWGNGCGLFGKGGIVTCAMFTCKKNMKGKVVQPENLEYTIVITPHSGEEHAVGNDTGKHGKEIKITPQSSITEAELTGYGTVTMECSPRTGLDFNEMVLLQMENKAWLVHRQWFLDLPLPWLPGADTQGSNWIQKETLVTFKNPHAKKQDVVVLGSQEGAMHTALTGATEIQMSSGNLLFTGHLKCRLRMDKLQLKGMSYSMCTGKFKVVKEIAETQHGTIVIRVQYEGDGSPCKIPFEIMDLEKRHVLGRLITVNPIVTEKDSPVNIEAEPPFGDSYIIIGVEPGQLKLNWFKKGSSIGQMFETTMRGAKRMAILGDTAWDFGSLGGVFTSIGKALHQVFGAIYGAAFSGVSWTMKILIGVIITWIGMNSRSTSLSVSLVLVGVVTLYLGVMVQADSGCVVSWKNKELKCGSGIFITDNVHTWTEQYKFQPESPSKLASAIQKAHEEGICGIRSVTRLENLMWKQITPELNHILSENEVKLTIMTGDIKGIMQAGKRSLRPQPTELKYSWKTWGKAKMLSTESHNQTFLIDGPETAECPNTNRAWNSLEVEDYGFGVFTTNIWLKLREKQDVFCDSKLMSAAIKDNRAVHADMGYWIESALNDTWKIEKASFIEVKSCHWPKSHTLWSNEVLESEMIIPKNFAGPVSQHNYRPGYHTQTAGPWHLGKLEMDFDFCEGTTVVVTEDCGNRGPSLRTTTASGKLITEWCCRSCTLPPLRYRGEDGCWYGMEIRPLKEKEENLVNSLVTAGHGQIDNFSLGVLGMALFLEEMLRTRVGTKHAILLVAVSFVTLITGNMSFRDLGRVMVMVGATMTDDIGMGVTYLALLAAFKVRPTFAAGLLLRKLTSKELMMTTIGIVLLSQSTIPETILELTDALALGMMVLKMVRKMEKYQLAVTIMAILCVPNAVILQNAWKVSCTILAVVSVSPLFLTSSQQKADWIPLALTIKGLNPTAIFLTTLSRTNKKRSWPLNEAIMAVGMVSILASSLLKNDIPMTGPLVAGGLLTVCYVLTGRSADLELERAADVKWEDQAEISGSSPILSITISEDGSMSIKNEEEEQILTILIRTGLLVISGLFPVSMPITAAAWYLWEVKKQRAGVLWDVPSPPPVGKAELEDGAYRIKQKGILGYSQIGAGVYKEGTFHTMWHVTRGAVLMHKGKRIEPSWADVKKDLISYGGGWKLEGEWKEGEEVQVLALEPGKNPRAVQTKPGLFKTNAGTIGAVSLDFSPGTSGSPIIDKKGKVVGLYGNGVVTRSGAYVSAIAQTEKSIEDNPEIEDDIFRKRKLTIMDLHPGAGKTKRYLPAIVREAIKRGLRTLILAPTRVVAAEMEEALRGLPIRYQTPAIRAEHTGREIVDLMCHATFTMRLLSPVRVPNYNLIIMDEAHFTDPASIAARGYISTRVEMGEAAGIFMTATPPGSRDPFPQSNAPIMDEEREIPERSWSSGHEWVTDFKGKTVWFVPSIKAGNDIAACLRKNGKKVIQLSRKTFDSEYVKTRTNDWDFVVTTDISEMGANFKAERVIDPRRCMKPVILTDGEERVILAGPMPVTHSSAAQRRGRIGRNPKNENDQYIYMGEPLENDEDCAHWKEAKMLLDNINTPEGIIPSMFEPEREKVDAIDGEYRLRGEARKTFVDLMRRGDLPVWLAYRVAAEGINYADRRWCFDGIKNNQILEENVEVEIWTKEGERKKLKPRWLDARIYSDPLALKEFKEFAAGRKSLTLNLITEMGRLPTFMTQKARDALDNLAVLHTAEAGGRAYNHALSELPETLETLLLLTLLATVTGGIFLFLMSGRGIGKMTLGMCCIITASILLWYAQIQPHWIAASIILEFFLIVLLIPEPEKQRTPQDNQLTYVVIAILTVVAATMANEMGFLEKTKKDLGLGSITTQQPESNILDIDLRPASAWTLYAVATTFVTPMLRHSIENSSVNVSLTAIANQATVLMGLGKGWPLSKMDIGVPLLAIGCYSQVNPITLTAALFLLVAHYAIIGPGLQAKATREAQKRAAAGIMKNPTVDGITVIDLDPIPYDPKFEKQLGQVMLLVLCVTQVLMMRTTWALCEALTLATGPISTLWEGNPGRFWNTTIAVSMANIFRGSYLAGAGLLFSIMKNTTNTRRGTGNIGETLGEKWKSRLNALGKSEFQIYKKSGIQEVDRTLAKEGIKRGETDHHAVSRGSAKLRWFVERNMVTPEGKVVDLGCGRGGWSYYCGGLKNVREVKGLTKGGPGHEEPIPMSTYGWNLVRLQSGVDVFFTPPEKCDTLLCDIGESSPNPTVEAGRTLRVLNLVENWLNNNTQFCIKVLNPYMPSVIEKMEALQRKYGGALVRNPLSRNSTHEMYWVSNASGNIVSSVNMISRMLINRFTMRHKKATYEPDVDLGSGTRNIGIESEIPNLDIIGKRIEKIKQEHETSWHYDQDHPYKTWAYHGSYETKQTGSASSMVNGVVRLLTKPWDVVPMVTQMAMTDTTPFGQQRVFKEKVDTRTQEPKEGTKKLMKITAEWLWKELGKKKTPRMCTREEFTRKVRSNAALGAIFTDENKWKSAREAVEDSRFWELVDKERNLHLEGKCETCVYNMMGKREKKLGEFGKAKGSRAIWYMWLGARFLEFEALGFLNEDHWFSRENSLSGVEGEGLHKLGYILRDVSKKEGGAMYADDTAGWDTRITLEDLKNEEMVTNHMEGEHKKLAEAIFKLTYQNKVVRVQRPTPRGTVMDIISRRDQRGSGQVGTYGLNTFTNMEAQLIRQMEGEGVFKSIQHLTVTEEIAVQNWLARVGRERLSRMAISGDDCVVKPLDDRFASALTALNDMGKVRKDIQQWEPSRGWNDWTQVPFCSHHFHELIMKDGRVLVVPCRNQDELIGRARISQGAGWSLRETACLGKSYAQMWSLMYFHRRDLRLAANAICSAVPSHWVPTSRTTWSIHAKHEWMTTEDMLTVWNRVWIQENPWMEDKTPVESWEEIPYLGKREDQWCGSLIGLTSRATWAKNIQTAINQVRSLIGNEEYTDYMPSMKRFRREEEEAGVLW

>DENV2_ACH61726_Colombia

MNNQRKKARSTPFNMLKRERNRVSTVQQLTKRFSLGMLQGRGPLKLFMALVAFLRFLTIPPTAGILKRWGTIKKSKAINVLRGFRKEIGRMLNILNRRRRTAGVIVMLIPTAMAFHLTTRNGEPHMIVGRQEKGKSLLFKTEDGVNMCTLMAIDLGELCEDTITYKCPLLRQNEPEDIDCWCNSTSTWVTYGTCTTTGEHRREKRSVALVPHVGMGLETRTETWMSSEGAWKHVQRIETWILRHPGFTIMAAILAYTIGTTHFQRALIFILLTAVAPSMTMRCIGISNRDFVEGVSGGSWVDIVLEHGSCVTTMAKNKPTLDFELIKTEAKQPATLRKYCIEAKLTNTTTESRCPTQGEPSLNEEQDKRFICKHSMVDRGWGNGCGLFGKGGIVTCAMFTCKKNMEGKVVQPENLEYTIVITPHSGEEHAVGNDTGKHGKEIKITPQSSITEAELTGYGTVTMECSPRTGLDFNEMVLLQMEDKAWLVHRQWFLDLPLPWLPGADTQGSNWIQKETLVTFKNPHAKKQDVVVLGSQEGAMHTALTGATEIQMSSGNLLFTGHLKCRLRMDKLQLKGMSYSMCTGKFKIVKEIAETQHGTIVIRVQYEGDGSPCKIPFEITDLEKRHVLGRLITVNPIVTEKDSPVNIEAEPPFGDSYIIIGVEPGQLKLNWFKKGSSIGQMFETTMRGAKRMAILGDTAWDFGSLGGVFTSIGKALHQVFGAIYGAAFSGVSWTMKILIGVIITWIGMNSRSTSLSVSLVLVGVVTLYLGAMVQADSGCVVSWKNKELKCGSGIFITDNVHTWTEQYKFQPESPSKLASAIQKAHEEGICGIRSATRLENLMWKQITPELNHILSENEVKLTIMTGDIKGIMQAGKRSLRPQPTELKYSWKTWGKAKMLSTESHNQTFLIDGPETAECPNTNRAWNSLEVEDYGFGVFTTNIWLKLREKQDVFCDSKLMSAAIKDNRAVHADMGYWIESALNDTWKMEKASFIEVKSCHWPKSHTLWSNGVLESEMIIPKNFAGPVSQHNYRPGYHTQTAGPWHLGKLEMDFDFCEGTTVVVTEDCGNRGPSLRTTTASGKLITEWCCRSCTLPPLRYRGEDGCWYGMEIRPLKEKEENLVNSLVTAGHGQIDNFSLGVLGMALFLEEMLRTRVGTKHAILLAAVSFVTLITGNMSFRDLGRVMVMVGATMTDDIGMGVTYLALLAAFKVRPTFAAGLLLRKLTSKELMMATIGIALLSQSTIPETILELTDALALGMMVLKIVRNMEKYQLAVTIMAISCVPNAVILQNAWKVSCTILAVVSVSPLLLTSSQQKADWIPLALTIKGLNPTAIFLTTLSRTSKKRSWPLNEAIMAVGMVSILASSLLKNDIPMTGPLVAGGLLTVCYVLTGRSADLELERAADVKWEDQAEISGSSPILSITISEDGSMSIKNEEEEQTLTILIRTGLLVISGVFPVSIPITAAAWYLWEVKKQRAGVLWDVPSPPPVGKAELEDGAYRIKQRGILGYSQIGAGVYKEGTFHTMWHVTRGAVLMHRGKRIEPSWADVKKDLISYGGGWKLEGEWKEGEEVQVLALEPGKNPRAVQTKPGIFKTNTGTIGAVSLDFSPGTSGSPIVDRKGKVVGLYGNGVVTRSGAYVSAIAQTEKSIEDNPEIEDDIFRKKRLTIMDLHPGAGKTKRYLPAIVREAIKRGLRTLILAPTRVVAAEMEEALRGLPIRYQTPAIKAEHTGREIVDLMCHATFTMRLLSPVRVPNYNLIIMDEAHFTDPASIAARGYISTRVEMGEAAGIFMTATPPGSRDPFPQSNAPIMDEEREIPERSWNSGHEWVTDFKGKTVWFVPSIKAGNDIAACLRKNGKKVIQLSRKTFDSEYVKTRANDWDFVVTTDISEMGANFKAERVIDPRRCMKPVILTDGEERVILAGPMPVTHSSAAQRRGRIGRNPKNENDQYIYMGEPLENDEDCAHWKEAKMLLDNINTPEGIIPSMFEPEREKVDAIDGEYRLRGEARKTFVDLMRRGDLPVWLAYKVAAEGINYADRKWCFDGIKNNQILEENVEVEIWTKEGERKKLKPRWLDARIYSDPLALKEFKEFAAGRKSLTLNLITEMGRLPTFMTQKARNALDNLAVLHTAEAGGRAYNHALSELPETLETLLLLTLLATVTGGIFLFLMSGKGIGKMTLGMCCIITASVLLWYAQIQPHWIAASIILEFFLIVLLIPEPEKQRTPQDNQLTYVVIAILTVVAATMANEMGFLEKTKKDLGLGSITTQESESNILDIDLRPASAWTLYAVATTFVTPMLRHSIENSSVNVSLTAIANQATVLMGLGKGWPLSKMDIGVPLLAIGCYSQVNPITLTAALLLLVAHYAIIGPGLQAKATREAQKRAAAGIMKNPTVDGITVIDLEPIPYDPKFEKQLGQVMLLILCVTQVLMMRTTWALCEALTLATGPISTLWEGNPGRFWNTTIAVSMANIFRGSYLAGAGLLFSIMKNTTNTRRGTGNIGETLGEKWKSRLNALGKSEFQIYKKSGIQEVDRTLAKEGIKRGETDHHAVSRGSAKLRWFVERNMVTPEGKVVDLGCGRGGWSYYCGGLKNVREVKGLTKGGPGHEEPIPMSTYGWNLVRLQSGVDVFFTPPEKCDTLLCDIGESSPNPTIEAGRTLRVLNLVENWLNNNTQFCIKVLNPYMPSVIEKMETLQRKYGGALVRNPLSRNSTHEMYWVSNATGNIVSSVNMISRMLINRFTMKHKKATYEPDVDLGSGTRNIGIESEIPNLDIIGKRIEKIKQEHETSWHYDQDHPYKTWAYHGSYETKQTGSASSMVNGVVRLLTKPWDVVPMVTQMAMTDTTPFGQQRVFKEKVDTRTQEPKEGTKKLMKITAEWLWKELGKKKTPRMCTREEFTRKVRSNAALGAIFTDENKWKSAREAVEDGRFWELVDRERNLHLEGKCETCVYNMMGKREKKLGEFGKAKGSRAIWYMWLGARFLEFEALGFLNEDHWFSRGNSLSGVEGEGLHRLGYILRDVSKKEGGAMYADDTAGWDTRITLEDLKNEEMVTNHMKGEHKKLAEAIFKLTYQNKVVRVQRPTPRGTVMDIISRRDQRGSGQVGTYGLNTFTNMEAQLIRQMEGEGIFKSIQHLTATEEIAVQNWLARVGRERLSRMAISGDDCVVKPIDDRFASALTALNDMGKVRKDIQQWEPSRGWNDWTQVPFCSHHFHELVMKDGRVLVVPCRNQDELIGRARISQGAGWSLKETACLGKSYAQMWTLMYFHRRDLRLAANAICSAVPSHWVPTSRTTWSIHAKHEWMTTEDMLAVWNRVWIQENPWMEDKTPVESWEEVPYLGKREDQWCGSLIGLTSRATWAKNIQTAINQVRSLIGNEEYTDYMPSMKRFRREEEEAGVLW

>DENV2_ACH61724_Colombia

MNNQRKKARSTPFNMLKRERNRVSTVQQLTKRFSLGMLQGRGPLKLFMALVAFLRFLTIPPTAGILKRWGTIKKSKAINVLRGFRKEIGRMLNILNRRRRTAGVIVMLIPTAMAFHLTTRNGEPHMIVGRQEKGKSLLFKTEDGVNMCTLMAIDLGELCEDTITYKCPLLRQNEPEDIDCWCNSTSTWVTYGTCTTTGEHRREKRSVALVPHVGMGLETRTETWMSSEGAWKHVQRIETWILRHPGFTIMAAILAYTIGTTHFQRALIFILLTAVAPSMTMRCIGISNRDFVEGVSGGSWVDIVLEHGSCVTTMAKNKPTLDFELIKTEAKQPATLRKYCIEAKLTNTTTESRCPTQGEPSLNEEQDKRFICKHSMVDRGWGNGCGLFGKGGIVTCAMFTCKKNMEGKVVQPENLEYTIVITPHSGEEHAVGDDTGKHGKEIKITPQSSITEAELTGYGTVTMECSPRTGLDFNEMVLLQMEDKAWLVHRQWFLDLPLPWLPGADTQGSNWIQKETLVTFKNPHAKKQDVVVLGSQEGAMHTALTGATEIQMSSGNLLFTGHLKCRLRMDKLQLKGMSYSMCTGKFKIVKEIAETQHGTIVIRVQYEGDGSPCKIPFEITDLEKRHVLGRLITVNPIVIEKDSPVNIEAEPPFGDSYIIIGVEPGQLKLNWFKKGSSIGQMFETTMRGAKRMAILGDTAWDFGSLGGVFTSIGKALHQVFGAIYGAAFSGVSWTMKILIGVVITWIGMNSRSTSLSVSLVLVGVVTLYLGAMVQADSGCVVSWKNKELKCGSGIFITDNVHTWTEQYKFQPESPSKLASAIQKAHEEGICGIRSVTRLENLMWKQITPELNHILSENEVKLTIMTGDTKGIMQAGKRYLRPQPTELKYSWKTWGKAKMLSTESHNQTFLIDGPETAECPNTNRAWNSLEVEDYGFGVFTTNIWLKLREKQDVFCDSKLMSAAIKDNRAVHADMGYWIESALNDTWKMEKASFIEVKSCHWPKSHTLWSNGVLESEMIIPKNFAGPVSQHNYRPGYHTQTAGPWHLGKLEMDFDFCEGTTVVVTEDCGNRGPSLRTTTASGKLITEWCCRSCTLPPLRYRGEDGCWYGMEIRPLKEKEENLVNSLVTAGHGQIDNFSLGVLGMALFLEEMLRTRVGTKHAILLVAVSFVTLITGNMSFRDLGRVMVMVGATMTDDIGMGVTYLALLAAFKVRPTFAAGLLLRKLTSKELMMATIGITLLSQSTIPETILELTDALALGMMVLKIVRNMEKYQLAVTIMAISCVPNAVILRNAWKVSCTILAAVSVSPLLLTSSQQKADWIPLALTIKGLNPTAIFLTTLSRTSKKRSWPLNEAIMAVGMVSILASSLLKNDIPMTGPLVAGGLLTVCYVLTGRSADLELERAADVKWEDQAEISGSSPILSITISEDGSMSIKNEEEETTLTILIRTGLLVISGVFPVSIPITAAAWYLWEVKKQRAGVLWDVPSPPPVGKAELEDGAYRIKQRGILGYSQIGAGVYKEGTFHTMWHVTRGAVLMHRGKRIEPSWADVKKDLISYGGGWKLEGEWKEGEEVQVLALEPGKNPRAVQTKPGIFKTNTGTIGAVSLDFSPGTSGSPIVDRKGKVVGLYGNGVVTRSGAYVSAIAQTEKSIEDNPEIEDDIFRKKRLTIMDLHPGAGKTKRYLPAIVREAIKRGLRTLILAPTRVVAAEMEEALRGLPIRYQTPAIKAEHTGREIVDLMCHATFTMRLLSPVRVPNYNLIIMDEAHFTDPASIAARGYISTRVEMGEAAGIFMTATPPGSRDPFPQSNAPIMDEEREIPERSWNSGHEWVTDFKGKTVWFVPSIKAGNDIAACLRKNGKKVIQLSRKTFDSEYVKTRANDWDFVVTTDISEMGANFKAERVIDPRRCMKPVILTDGEERVILAGPMPVTHSSAAQRRGRIGRNPKNENDQYIYMGEPLENDEDCAHWKEAKMLLDNINTPEGIIPSMFEPEREKVDAVDGEYRLRGEARKTFVDLMRRGDLPVWLAYKVAAEGINYADRKWCFDGIKNNQILEENVEVEIWTKEGERKKLKPRWLDARIYSDPLALKEFKEFAAGRKSLTLNLITEMGRLPTFMTQKARNALDNLAVLHTAEAGGRAYNHALSELPETLETLLLLTLLATVTGGIFLFLMSGKGIGKMTLGMCCIITASVLLWYAQIQPHWIAASIILEFFLIVLLIPEPEKQRTPQDNQLTYVVIAILTVVAATMANEMGFLEKTKKDLGLGSITTQESESNILDIDLRPASAWTLYAVATTFVTPMLRHSIENSSVNVSLTAIANQATVLMGLGKGWPLSKMDIGVPLLAIGCYSQVNPMTLTAALLLLVAHYAIIGPGLQAKATREAQKRAAAGIMKNPTVDGITVIDLEPIPYDPKFEKQLGQVMLLILCVTQVLMMRTTWALCEALTLATGPISTLWEGNPGRFWNTTIAVSMANIFRGSYLAGAGLLFSIMKNTTNTRRGTGNIGETLGEKWKSRLNALGKSEFQIYKKSGIQEVDRTLAKEGIKRGETDHHAVSRGSAKLRWFVERNMVTPEGKVVDLGCGRGGWSYYCGGLKNVREVKGLTKGGPGHEEPIPMSTYGWNLVRLQSGVDVFFTPPEKCDTLLCDIGESSPNPTIEAGRTLRVLNLVENWLNNNTQFCIKVLNPYMPSVIEKMETLQRKYGGALVRNPLSRNSTHEMYWVSNATGNIVSSVNMISRMLINRFTMKHKKATYEPDVDLGSGTRNIGIESEIPNLDIIGKRIEKIKQEHETSWHYDQDHPYKTWAYHGSYETKQTGSASSMVNGVVRLLTKPWDVVPMVTQMAMTDTTPFGQQRVFKEKVDTRTQEPKEGTKKLMRITAEWLWKELGKKKTPRMCTREEFTRKVRSNAALGAIFTDENKWKSAREAVEDGRFWELVDRERNLHLEGKCETCVYNMMGKREKKLGEFGKAKGSRAIWYMWLGARFLEFEALGFLNEDHWFSRGNSLSGVEGEGLHRLGYILRDVGKKEGGAMYADDTAGWDTRITLEDLKNEEMVTNHMKGEHKKLAEAIFKLTYQNKVVRVQRPTPRGTVMDIISRRDQRGSGQVGTYGLNTFTNMEAQLIRQMEGEGIFKSIQHLTATEEIAAQNWLARVGRERLSRMAISGDDCVVKPIDDRFASALTALNDMGKVRKDIQQWEPSRGWNDWTQVPFCSHHFHELVMKDGRVLVVPCRNQDELIGRARISQGAGWSLKETACLGKSYAQMWTLMYFHRRDLRLAANAICSAVPSHWVPTSRTTWSIHAKHEWMTTEDMLAVWNRVWIQENPWMEDKTPVESWEEVPYLGKREDQWCGSLIGLTSRATWAKNIQTAINQVRSLIGNEEYTDYMPSMKRFRREEEEAGVLW

>DENV2_AAV70829_Tonga

MNNQRKKARNTPFNMLKRERNRVSTVQQLTKRFSLGMLQGRGPLKLFMALVAFLRFLTIPPTAGILKRWGTIKKSKAINVLRGFRKEIGRMLNILNRRRRTVGMIIMLTPTVMAFHLTTRNGEPHMIVSRQEKGKSLLFKTKDGTNMCTLMAMDLGELCEDTITYKCPFLKQNEPEDIDCWCNSTSTWVTYGTCTTTGEHRREKRSVALVPHVGMGLETRTETWMSSEGAWKHAQRIETWILRHPGFTIMAAILAYTIGTTHFQRVLIFILLTAIAPSMTMRCIGISNRDFVEGVSGGSWVDIVLEHGSCVTTMAKNKPTLDFELIKTEAKQPATLRKYCIEAKLTNTTTDSRCPTQGEPTLNEEQDKRFVCKHSMVDRGWGNGCGLFGKGGIVTCAMFTCKKNMEGKIVQPENLEYTVVITPHSGEEHAVGNDTGKHGKEVKITPQSSITEAELTGYGTVTMECSPRTGLDFNEMVLLQMEDKAWLVHRQWFLDLPLPWLPGADTQGSNWIQKETLVTFKNPHAKKQDVVVLGSQEGAMHTALTGATEIQMSSGNLLFTGHLKCRLRMDKLQLKGMSYSMCTGKFKIVKEIAETQHGTIVIRVQYEGDGSPCKIPFEIMDLEKRHVLGRLITVNPIVTEKDSPVNIEAEPPFGDSYIIIGVEPGQLKLDWFKKGSSIGQMFETTMRGAKRMAILGDTAWDFGSLGGVFTSIGKALHQVFGAIYGAAFSGVSWTMKILIGVIITWIGMNSRSTSLSVSLVLVGIVTLYLGVMVQADSGCVVSWKNKELKCGSGIFVTDNVHTWTEQYKFQPESPSKLASAIQKAHEEGICGIRSVTRLENLMWKQITSELNHILSENEVKLTIMTGDIKGIMQVGKRSLRPQPTELRYSWKTWGKAKMLSTELHNQTFLIDGPETAECPNTNRAWNSLEVEDYGFGVFTTNIWLRLREKQDVFCDSKLMSAAIKDNRAVHADMGYWIESALNDTWKIEKASFIEVKSCHWPKSHTLWSNGVLESEMVIPKNFAGPVSQHNNRPGYYTQTAGPWHLGKLEMDFDFCEGTTVVVTENCGNRGPSLRTTTASGKLITEWCCRSCTLPPLRYRGEDGCWYGMEIRPLKEKEENLVSSLVTAGHGQIDNFSLGILGMALFLEEMLRTRVGTKHAILLVAVSFVTLITGNMSFRDLGRVMVMVGATMTDDIGMGVTYLALLAAFRVRPTFAAGLLLRKLTSKELMMTTIGIVLLSQSSIPETILELTDALALGMMVLKMVRNMEKYQLAVTIMAILCVPNAVILQNAWKVSCTILAVVSVSPLLLTSSQQKADWIPLALTIKGLNPTAIFLTTLSRTNKKRSWPLNEAIMAVGMVSILASSLLKNDIPMTGPLVAGGLLTVCYVLTGRSADLELERATDVKWDDQAEISGSSPILSITISEDGSMSIKNEEEEQTLTILIRTGLLVISGLFPVSIPITAAAWYLWEVKKQRAGVLWDVPSPPPVGKAELEDGAYRIKQKGILGYSQIGAGVYKEGTFHTMWHVTRGAVLMHKGKRIEPSWADVKKDLISYGGGWKLEGEWKEGEEVQVLALEPGKNPRAVQTKPGLFRTNTGTIGAVSLDFSPGTSGSPIVDKKGKVVGLYGNGVVTRSGAYVSAIAQTEKSIEDNPEIEDDIFRKRRLTIMDLHPGAGKTKRYLPAIVREAIKRGLRTLILAPTRVVAAEMEEALRGLPIRYQTPAIRAEHTGREIVDLMCHATFTMRLLSPIRVPNYNLIIMDEAHFTDPASIAARGYISTRVEMGEAAGIFMTATPPGSRDPFPQSNAPIMDEEREIPERSWNSGHEWVTDFKGKTVWFVPSIKTGNDIAACLRKNGKRVIQLSRKTFDSEYVKTRTNDWDFVVTTDISEMGANFKAERVIDPRRCMKPVILTDGEERVILAGPMPVTHSSAAQRRGRIGRNPRNENDQYIYMGEPLENDEDCAHWKEAKMLLDNINTPEGIIPSLFEPEREKVDAIDGEYRLRGEARKTFVDLMRRGDLPVWLAYKVAAEGINYADRRWCFDGTRNNQILEENVEVEIWTKEGERKKLKPRWLDARIYSDPLALKEFKEFAAGRKSLTLNLITEMGRLPTFMTQKARDALDNLAVLHTAEAGGKAYNHALSELPETLETLLLLTLLATVTGGIFLFLMSGRGMGKMTLGMCCIITASILLWYAQIQPHWIAASIILEFFLIVLLIPEPEKQRTPQDNQLTYVIIAILTVVAATMANEMGFLEKTKKDLGLGNIATQQPESNILDIDLRPASAWTLYAVATTFITPMLRHSIENSSVNVSLTAIANQATVLMGLGKGWPLSKMDIGVPLLAIGCYSQVNPITLTAALLLLVAHYAIIGPGLQAKATREAQKRAAAGIMKNPTVDGITVIDLDPIPYDPKFEKQLGQVMLLVLCVTQVLMMRTTWALCEALTLATGPVSTLWEGNPGRFWNTTIAVSMANIFRGSYLAGAGLLFSIMKNTTSTRRGTGNIGETLGEKWKSRLNALGKSEFQIYKKSGIQEVDRTLAKEGIKRGETDHHAVSRGSAKLRWFVERNLVTPEGKVVDLGCGRGGWSYYCGGLKNVREVKGLTKGGPGHEEPIPMSTYGWNLVRLQSGVDVFFVPPEKCDTLLCDIGESSPNPTVEAGRTLRVLNLVENWLNNNTQFCVKVLNPYMPSVIERMETLQRKYGGALVRNPLSRNSTHEMYWVSNASGNIVSSVNMISRMLINRFTMRHKKATYEPDVDLGSGTRNIGIESETPNLDIIGKRIEKIKQEHETSWHYDQDHPYKTWAYHGSYETKQTGSASSMVNGVVRLLTKPWDVVPMVTQMAMTDTTPFGQQRVFKEKVDTRTQEPKEGTKKLMKITAEWLWKELGKKKTPRMCTREEFTKKVRSNAALGAIFTDENKWKSAREAVEDSRFWELVDKERNLHLEGKCETCVYNMMGKREKKLGEFGKAKGSRAIWYMWLGARFLEFEALGFLNEDHWFSRENSLSGVEGEGLHKLGYILREVSKKEGGAMYADDTAGWDTRITIEDLKNEEMITNHMAGEHKKLAEAIFKLTYQNKVVRVQRPTPRGTVMDIISRRDQRGSGQVGTYGLNTFTNMEAQLIRQMEGEGIFKSIQHLTASEEIAVQDWLVRVGRERLSRMAISGDDCVVKPLDDRFARALTALNDMGKVRKDIQQWEPSRGWNDWTQVPFCSHHFHELIMKDGRTLVVPCRNQDELIGRARISQGAGWSLRETACLGKSYAQMWSLMYFHRRDLRLAANAICSAVPSHWIPTSRTTWSIHASHEWMTTEDMLTVWNRVWILENPWMEDKTPVESWEEIPYLGKREDQWCGSLIGLTSRATWAKNIQTAINQVRSLIGNEEYTDYMPSMKRFRREEEEAGVLW

>DENV2_AAK67712_Australia

MNNQRKKARNTPFNMLKRERNRVSTVQQLTKRFSLGMLQGRGPLKLFMALVAFLRFLTIPPTAGILKRWGTIKKSKAINVLRGFRKEIGRMLNILNRRRRTAGIIIMMIPTVMAFHLTTRNGEPHMIVSRQEKGKSLLFKTENGVNMCTLMAMDLGELCEDTITYNCPLLRQNEPEDIDCGCHSTSTWVTYGTCTATGEHRREKRSVALVPHVGMGLETRTETWMSSEGAWKHAQRIETWVLRHPGFTIMAAILAYTIGTTYFQRVLIFILLTAVTPSMTMRCIGISNRDFVEGVSGGSWVDIVLEHGSCVTTMAKNKPTLDFELVKTEAKHPATLRKYCIEAKLTNTTTASRCPTQGEPSLNEEQDKRFVCKHSMVDRGWGNGCGLFGKGGIVTCAMFTCKKNMEGKVVQPENLEYTIVITPHSGEENAVGNDTGKHGKEIKVTPQSSITEAELTGYGTVTMECSPRTGLDFNEMVLLQMENKAWLVHRQWFLDLPLPWLPGADTQGSNWIQKETLVTFKNPHAKKQDVVVLGSQEGAMHTALTGATEIQMSSGNLLFTGHLKCRLRMDKLQLKGMSYSMCTGKFKVVKEIAETQHGTIVIRVQYEGDGSPCKIPFEIMDLEKRHVLGRLITVNPIVTEKDSPVNIEAEPPFGDSYIIIGVEPGQLKLSWFKKGSSIGQMFETTMRGAKRMAILGDTAWDFGSLGGVFTSIGKALHQVFGAIYGAAFSGVSWTMKILIGVVITWIGMNSRSTSLSVSLVLVGVVTLYLGVMVQADSGCVVSWKNKELKCGSGIFITDNVHTWTEQYKFQPESPSKLASAIQKAHEEGICGIRSVTRLENLMWKQITPELNHILSENEVKLTIMTGDIKGIMQAGKRSLRPQPTELKYSWKAWGKAKMLSTELHNHTFLIDGPETAECPNTNRAWNSLEVEDYGFGVFTTNIWLKLKERQDVFCDSKLMSAAIKDNRAVHADMGYWIESALNDTWKIEKASFIEVKSCHWPKSHTLWSNGVLESEMIIPKNFAGPVSQHNYRPGYHTQTAGPWHLGRLEMDFDFCEGTTVVVTEDCGNRGPSLRTTTASGKLITEWCCRSCTLPPLRYRGEDGCWYGMEIRPLKEKEENLVNSLVTAGHGQIDNFSLGVLGMALFLEEMLRTRVGTKHAILLVAVSFVTLITGNMSFRDLGRVMVMVGATMTDDIGMGVTYLALLAAFKVRPTFAAGLLLRKLTSKELMMTTIGIVLLSQSTIPETILELTDAWALGMMVLKIVRNMEKYQLAVTIMAILCVPNAVILQNAWKVSCTTLAVVSVSPLLLTSSQQKADWIPLALTIKGLNPTAIFLTTLSRTSKKRSWPLNEAIMAVGMVSILASSLLKNDIPMTGPLVAGGLLTVCYVLTGRSADLELERAADVRWEEQAEISGSSPILSITISEDGSMSIKNEEEEQTLTILIRTGLLVISGLFPASIPITAAAWYLWEVKKQRAGVLWDVPSPPPVGKAELEDGAYRIKQKGILGYSQIGAGVYKEGTFHTMWHVTRGAVLMHKGKRIEPSWADVKKDLISYGGGWKLEGEWKEGEEVQVLALEPGKNPRAVQTKPGLFKTNTGTIGAVSLDFSPGTSGSPIVDKKGKVVGLYGNGVVTRSGAYVSAIAQTEKSIEDNPEIEDDIFRKKRLTIMDLHPGAGKTKRYLPAIVREAIKRGLRTLILAPTRVVAAEMEEALRGLPIRYQTPAIRAEHTGREIVDLMCHATFTMRLLSPIRVPNYNLIIMDEAHFTDPASIAARGYISTRVEMGEAAGIFMTATPPGSRDPFPQSNAPIMDEEREIPERSWNSGHEWVTDFKGKTVWFVPSIKAGNDIAACLRKNGKKVIQLSRKTFDSEYIKTRTNDWDFVVTTDISEMGANFKAERVIDPRRCMKPVILTDGEERVILAGPMPVTHSSAAQRRGRVGRNPKNENDQYIYMGEPLENDEDCAHWKEAKMLLDNINTPEGIIPSMFEPEREKVDAIDGEYRLRGEARKTFVDLMRRGDLPVWLAYRVAAEGINYADRRWCFDGVKNNQILEENVEVEIWTKEGERKKLKPRWLDARIYSDPLALKEFKEFAAGRKSLTLNLITEMGRLPTFMTQKARNALDNLAVLHTAEAGGRAYNHALSELPETLETLLLLTLLATVTGGIFLFLMSGKGIGKMTLGMCCIITASILLWYAQIQPHWIAASIILEFFLIVLLIPEPEKQRTPQDNQLTYVVIAILTVVAATMANEMGFLEKTKKDFGLGSIATQQPESNILDIDLRPASAWTLYAVATTFITPMLRHSIENSSVNVSLTAIANQATVLMGLGKGWPLSKMDIGVPLLAIGCYSQVNPITLTAALLLLVAHYAIIGPGLQAKATREAQKRAAAGIMKNPTVDGITVIDLDPIPYDPKFEKQLGQVMLLVLCVTQVLMMRTTWALCEALTLATGPISTLWEGNPGRFWNTTIAVSMANIFRGSYLAGAGLLFSIMKNTANTRRGTGNTGETLGEKWKNRLNALGKSEFQIYKKSGIQEVDRTLAKEGIKRGETDHHAVSRGSAKLRWFVERNLVTPEGKVVDLGCGRGGWSYYCGGLKNVKEVKGLTKGGPGHEEPIPMSTYGWNLVRLQSGVDVFFTPPEKCDTLLCDIGESSPNPTVEAGRTLRVLNLVENWLNNNTQFCIKVLNPYMPSVIEKMEALQRKYGGALVRNPLSRNSTHEMYWVSNASGNIVSSVNMISRMLINRFTMRHKKATYEPDVDLGSGTRNIGIESETPNLDIIGKRIEKIKQEHETSWHYDQDHPYKTWAYHGSYETKQTGSASSMVNGVVRLLTKPWDIIPMVTQMAMTDTTPFGQQRVFKEKVDTRTQEPKEGTKKLMKITAEWLWKELGKKKTPRMCTREEFTRKVRSNAALGAIFTDENKWKSAREAVEDSGFWELVDKERNLHLEGKCETCVYNMMGKREKKLGEFGKAKGSRAIWYMWLGARFLEFEALGFLNEDHWFSRENSLSGVEGEGLHKLGYILRDVSKKEGGAMYADDTAGWDTRITLEDLKNEEMVTNHMEGEHKKLAEAIFKLTYQNKVVRVQRPTPRGTVMDIISRRDQRGSGQVVTYGLNTFTNMEAQLIRQMEGEGVFKSIQHLTVTEEIAVKNWLVRVGRERLSRMAISGDDCVVKPLDDRFASALTALNDMGKVRKDIQQWEPSRGWNDWTQVPFCSHHFHELIMKDGRVLVVPCRNQDELIGRARISQGAGWSLRETACLGKSYAQMWSLMYFHRRDLRLAANAICSAVPSHWVPTSRTTWSIHATHEWMTTEDMLTVWNRVWIQENPWMEDKTPVESWEEIPYLGKREDQWCGSLIGLTSRATWAKNIQTAINQVRSLIGNEEYTDYMPSMKRFRREEEEAGVLW

>DENV2_AAG30730_Martinique

MNNQRKKARSTPFNMLKRERNRVSTVQQLTKRFSLGMLQGRGPLKLFMALVAFLRFLTIPPTAGILKRWGTIKKSKAINVLRGFRKEIGRMLNILNRRRRTAGVIVMLIPTAMAFHLTTRNGEPHMIVGRQEKGKSLLFKTEDGVNMCTLMAIDLGELCEDTITYKCPFLRQNEPEDIDCWCNFTSTWVTYGTCTTTGEHRREKRSVALVPHVGMGLETRTETWMSSEGAWKHVQRIETWILRHPGFTIMAAILAYTIGTTHFQRALIFILLTAVAPSMTMRCIGISNRDFVEGVSGGSWVDIVLEHGSCVTTMAKNKPTLDFELIKTEAKQPATLRKYCIEAKLTNTTTESRCPTQGEPSLNEEQDKRFICKHSMVDRGWGNGCGLFGKGGIVTCAMFTCKKNMEGKVVQPENLEYTIVITPHSGEEHAVGNDTGKHGKEIKITPQSSITEAELTGYGTVTMECSPRTGLDFNEMVLLQMEDKAWLVHRQWFLDLPLPWLPGADTQGSNWIQKETLVTFKNPHAKKQDVVVLGSQEGAMHTALTGATEIQMSSGNLLFTGHLKCRLRMDKLQLKGMSYSMCTGKFKIVKEIAETQHGTIVIRVQYEGDGSPCKIPFEITDLEKRHVLGRLITVNPIVTEKDSPVNIEAEPPFGDSYIIIGVEPGQLKLNWFKKGSSIGQMFETTMRGAKRMAILGDTAWDFGSLGGVFTSIGKALHQVFGAIYGAAFSGVSWTMKILIGVIITWIGMNSRSTSLSVSLVLVGVVTLYLGAMVQADSGCVVSWKNKELKCGSGIFITDNVHTWTEQYKFQPESPSKLASAIQKAHEEGICGIRSVTRLENLMWKQITPELNHILSENEVKLTIMTGDIKGIMQAGKRSLRPQPTELKYSWKTWGKAKMLSTESHNQTFLIDGPETAECPNTNRAWNSLEVEDYGFGVFTTNIWLKLREKQDVFCDSKLMSAAIKDNRAVHADMGYWIESALNDTWKMEKASFIEVKSCHWPKSHTLWSNGVLESEMIIPKNFAGPVSQHNYRPGYHTQTAGPWHLGKLEMDFDFCEGTTVVVTEDCGNRGPSLRTTTASGKLITEWCCRSCTLPPLRYRGEDGCWYGMEIRPLKEKEENLVNSLVTAGHGQIDNFSLGVLGMALFLEEMLRTRVGTKHAILLVAVSFVTLITGNMSFRDLGRVMVMVGATMTDDIGMGVTYLALLAAFKVRPTFAAGLLLRKLTSKELMMATIGIALLSQSTMPETILELTDALALGMMVLKIVRNMEKYQLAVTIMAISCVPNAVILQNAWKVSCTILAAVSVSPLLLTSSQQKADWIPLALTIKGLNPTAIFLTTLSRTSKKRSWPLNEAIMAVGMVSILASSLLKNDIPMTGPLVAGGLLTVCYVLTGRSADLELERAADVKWEDQAEISGSSPILSITISEDGSMSIKNEEEEQTLTILIRTGLLVISGVFPVSIPITAAAWYLWEVKKQRAGVLWDVPSPPPVGKAELEDGAYRIKQRGILGYSQIGAGVYKEGTFHTMWHVTRGAVLMHRGKRIEPSWADVKKDLISYGGGWKLEGEWKEGEEVQVLALEPGKNPRAVQTKPGIFKTNTGTIGAVSLDFSPGTSGSPIVDRKGKVVGLYGNGVVTRSGAYVSAIAQTEKSIEDNPEIEDDIFRKKRLTIMDLHPGAGKTKRYLPAIVREAIKRGLRTLILAPTRVVAAEMEEALRGLPIRYQTPAIRAEHTGREIVDLMCHATFTMRLLSPVRVPNYNLIIMDEAHFTDPASIAARGYISTRVEMGEAAGIFMTATPPGSRDPFPQSNAPIMDEEREIPERSWNSGHEWVTDFKGKTVWFVPSIKAGNDIAACLRKNGKKVIQLSRKTFDSEYVKTRANDWDFVVTTDISEMGANFKAERVIDPRRCMKPVILTDGEERVILAGPMPVTHSSAAQRRGRIGRNPKNENDQYIYMGEPLENDEDCAHWKEAKMLLDNINTPEGIIPSMFEPEREKVDAIDGEYRLRGEARKTFVDLMRRGDLPVWLAYKVAAEGINYADRKWCFDGIKNNQILEENVEVEIWTKEGERKKLKPRWLDARIYSDPLALKEFKEFAAGRKSLTLNLITEMGRLPTFMTQKARNALDNLAVLHTAEAGGRAYNHALSELPETLETLLLLTLLATVTGGIFLFLMSGKGIGKMTLGMCCIITASSLLWYAQIQPHWIAASIILEFFLIVLLIPEPEKQRTPQDNQLTYVVIAILTVVAATMANEMGFLEKTKKDLGLGSIATQESESNILDIDLRPASAWTLYAVATTFVTPMLRHSIENSSVNVSLTAIANQATVLMGLGKGWPLSKMDIGVPLLAIGCYSQVNPITLTAALLLLVAHYAIIGPGLQAKATREAQKRAAAGIMKNPTVDGITVIDLEPIPYDPKFEKQLGQVMLLILCVTQVLMMRTTWALCEALTLATGPISTLWEGNPGRFWNTTIAVSMANIFRGSYLAGAGLLFSIMKNTTNTRRGTGNIGETLGEKWKSRLNALGKSEFQIYKKSGIQEVDRTLAKEGIKRGETDHHAVSRGSAKLRWFVERNMVTPEGKVVDLGCGRGGWSYYCGGLKNVREVKGLTKGGPGHEEPIPMSTYGWNLVRLQSGVDVFFTPPEKCDTLLCDIGESSPNPTIEAGRTLRVLNLVENWLNNNTQFCIKVLNPYMPSVIEKMETLQRKYGGALVRNPLSRNSTHEMYWVSNATGNIVSSVNMISRMLINRFTMKHKKATYEPDVDLGSGTRNIGIESEIPNLDIIGKRIEKIKQEHETSWHYDQDHPYKTWAYHGSYETKQTGSASSMVNGVVRLLTKPWDVVPMVTQMAMTDTTPFGQQRVFKEKVDTRTQEPKEGTKKLMKITAEWLWKELGKKKTPRMCTREEFTRKVRSNAALGAIFTDENKWKSAREAVEDGRFWELVDRERNLHLEGKCETCVYNMMGKREKKLGEFGKAKGSRAIWYMWLGARFLEFEALGFLNEDHWFSRGNSLSGVEGEGLHRLGYILREVGKKEGGAMYADDTAGWDTRITLEDLKNEEMVTNHMKGEHKKLAEAIFKLTYQNKVVRVQRPTPRGTVMDIISRRDQRGSGQVGTYGLNTFTNMEAQLIRQMEGEGIFKSIQHLTATEEIAVQNWLARVGRERLSRMAISGDDCVVKPIDDRFASALTALNDMGKVRKDIQQWEPSRGWNDWTQVPFCSHHFHELVMKDGRVLVVPCRNQDELIGRARISQGAGWSLKETACLGKSYAQMWTLMYFHKRDLRLAANAICSAVPSHWVPTSRTTWSIHAKHEWMTTEDMLAVWNRVWIQENPWMEDKTPVESWEEVPYLGKREDQWCGSLIGLTSRATWAKNIQTAINQVRSLIGNEEYTDYMPSMKRFRREEEEAGVLW

>DENV2_ASN77915_Myanmar

MNNQRKKAKNTPFNMLKRERNRVSTVQQLTKRFSLGMLQGRGPLKLFMALVAFLRFLTIPPTAGILKRWGTIKKSKAINVLRGFRKEIGRMLNILNRRRRSAGMIIMLIPTVMAFHLTTRNGEPHMIVGIQEKGKSLLFKTEDGVNMCTLMAMDLGELCEDTITYKCPLLRQNEPEDIDCWCNSTSTWVTYGTCTTTGEHRREKRSVALVPHVGMGLETRTETWMSSEGAWKHAQRIETWILRHPGFTIMAAILAYTIGTTHFQRVLIFILLTAVAPSMTMRCIGISNRDFVEGVSGGSWVDIVLEHGSCVTTMAKNKPTLDFELIKTEAKQPATLRKYCIEAKLTNTTTESRCPTQGEPSLKEEQDKRFVCKHSMVDRGWGNGCGLFGKGGIVTCAMFTCKKNMEGKIVQPENLEYTIVVTPHSGEEHAVGNDTGKHGKEIKVTPQSSITEAELAGYGTVTMECSPRTGLDFNEMVLLQMENKAWLVHRQWFLDLPLPWLPGADKQESNWIQKETLVTFKNPHAKKQDVVVLGSQEGAMHTALTGATEIQMSSGNLLFTGHLKCRLRMDKLQLKGMSYSMCTGKFKVVKEIAETQHGTIVIRVQYEGDGSPCKIPFEIMDLEKRYVLGRLITVNPIVTEKDSPVNIEAEPPFGDSYIIIGVEPGQLKLNWFKKGSSIGQMFETTMRGAKRMAILGDTAWDFGSLGGVFTSIGKALHQVFGAIYGAAFSGVSWTMKILIGVIITWIGMNSRSTSLSVSLVLVGIVTLYLGVMVQADSGCVVSWKNKELKCGSGIFITDNVHTWTEQYKFQPESPSKLASAIQKAQEEGICGIRSVTRLENLMWKQITPELNHILAENEVKLTIMTGDIKGIMQAGKRSLRPQPTELKYSWKTWGKAKMLSTESYNQTFLIDGPETAECPNTNRAWNSLEVEDYGFGVFTTNIWLKLKEKQDAFCDSKLMSAAIKDNRAVHADMGYWIESALNDTWKIEKASFIEVKNCHWPKSHTLWSNGVLESEMIIPKNLAGPVSQHNYRPGYYTQIAGPWHLGKLEMDFDFCDGTTVIVTEDCGNRGPSLRTTTASGKLITEWCCRSCTLPPLRYRGEDGCWYGMEIRPLKEKEENLVNSLVTAGHGQVDNFSLGVLGMALFLEEMLRTRVGTKHAILLVAVSFVTLITGNMSFKDLGRVVVMVGATMADDIGMGVTYLALLAAFKVRPTFAAGLLLRKLTSKELMMTTIGIVLLSQSTIPETVLELTDALALGMMVLKIVRNMEKYQLAVTIMAILCVPNTVILQNAWKVSCTILAVVSVSPLLLTSSQQKTDWIPLALTIKGLNPTAIFLTTLSRTNKKRSWPLNEAIMAVGMVSILASSLLKNDIPMTGPLVAGGLLTVCYVLTGRSADLELERATDVKWEDQAEISGSSPILSITISEDGSMSIKNEEEEQTLTILIRTGLLVISGLFPISIPITAAAWYLWEVKKQRAGVLWDVPSPPPMGKAELEDGAYRIKQKGILGYSQIGAGVYKEGTFHTMWHVTRGAVLMHKGKRIEPSWADVKKDLISYGGGWKLEGEWKEGEEVQVLALEPGKNPRAVQTKPGLFKTNTGTIGAVSLDFSPGTSGSPIIDKKGKVVGLYGNGVVTRSGAYVSAIAQTEKSIEDNPEIEDDIFRKRRLTIMDLHPGAGKTKRYLPAIVREAIKRGLRTLILAPTRVVAAEMEEALRGLPIRYQTPAIRAEHTGREIVDLMCHATFTMRLLSPVRVPNYNLIIMDEAHFTDPASIAARGYISTRVEMGEAAGIFMTATPPGSRDPFPQSNAPIIDEEREIPERSWNSGHEWVTDFKGKTVWFVPSIKAGNDIAACLRKNGKKVIQLSRKTFDSEYVKTRTNDWDFVVTTDISEMGANFKAERVIDPRRCMKPVILTDGEERVILAGPMPVTHSSAAQRRGRIGRNPKNENDQYIYMGEPLENDEDCAHWKEAKMLLDNINTPEGIIPSMFEPEREKVDAIDGEYRLRGEARKTFVDLMRRGDLPVWLAYKVAAEGINYADRRWCFDGIKNNQILEENVEVEIWTKEGERKKLKPRWLDARIYSDPLALKEFKEFAAGRKSLTLNLITEMGRLPTFMTQKTRDALDNLAVLHTAEAGGRAYNHALSELPETLETLLLLTLLATVTGGIFLFLMSGRGIGKMTLGMCCIITASILLWYAQIQPHWIAASIILEFFLIVLLIPEPEKQRTPQDNQLTYVVIAILTVVAATMANEMGFLEKTKKDLGLGSIATQQPESNILDIDLRPASAWTLYAVATTFVTPMLRHSIENSSVNVSLTAIANQATVLMGLGKGWPLSKMDIGVPLLAIGCYSQVNPITLTAALLLLVAHYAIIGPGLQAKATREAQKRAAAGIMKNPTVDGITVIDLDPIPYDPKFEKQLGQVMLLILCVTQVLMMRTTWALCEALTLATGPISTLWEGNPGRFWNTTIAVSMANIFRGSYLAGAGLLFSIMKNTTNTRRGTGNIGETLGEKWKSRLNALGKSEFQIYKKSGIQEVDRTLAKEGIKRGETDHHAVSRGSAKLRWFVERNMVTPEGKVVDLGCGRGGWSYYCGGLKNVREVKGLTKGGPGHEEPIPMSTYGWNLVRLQSGVDVFFIPPEKCDTLLCDIGESSPNPTVEAGRTLRVLNLVENWLNNNTQFCIKVLNPYMPSVIEKMEALQRKYGGALVRNPLSRNSTHEMYWVSNASGNIVSSVNMISRMLINRFTMRHKKATYEPDVDLGSGTRNIGIESEIPNLDIIGKRIEKIKQEHETSWHYDQDHPYRTWAYHGSYETKQTGSASSMVNGVVRLLTKPWDVLPTVTQMAMTDTTPFGQQRVFKEKVDTRTQEPKEGTKKLMKITAEWLWKELGKKKTPRMCTREEFTRKVRSNAALGAIFTDENKWKSAREAVEDGRFWELVDKERNLHLEGKCETCVYNMMGKREKKLGEFGKAKGSRAIWYMWLGARFLEFEALGFLNEDHWFSRENSLSGVEGEGLHKLGYILRDVSKKEGGAMYADDTAGWDTRITLEDLKNEEMVTNHMEGEHKKLAEAIFKLTYQNKVVRVQRPTPRGTVMDIISRRDQRGSGQVGTYGLNTFTNMEAQLIRQMEGEGVFKNIQHLTVTEEVAVQNWLARVGRERLSRMAISGDDCVVKPLDDRFAHALTALNDMGKIRKDIQQWEPSKGWNDWTQVPFCSHHFHELIMKDGRVLVVPCRNQDELIGRARISQGAGWSLRETACLGKSYAQMWSLMYFHRRDLRLAANAICSAVPSHWVPTSRTTWSIHAKHEWMTTEDMLTVWNRVWIRENPWMEDKTPVESWEEIPYLGKREDQWCGSLIGLTSRATWAKNIQAAINQVRSLIGNEEYTDYMPSMKRFRREEEEAGVLW

>DENV2_AHB51029_China

MNNQRKKAKTTPFNMLKRERNRVSTVQQLTKRFSLGMLQGRGPLKLFMALVAFLRFLTIPPTAGILKRWGTIKKSKAINVLRGFRKEIGRMLNILNRRRRSAGMIIMLIPTVMAFHLTTRNGEPHMIVSIQEKGKSLLFKTEDGVNMCTLMAMDLGELCEDTITYKCPLLRQNEPEDIDRWCNSTSTWVTYGTCTTTGEHRREKRSVALVPHVGMGLETRTETWMSSEGAWKHAQRIETWILRHPGFTIMAAILAYTIGTTHFQRALIFILLTAVAPPMTMRCIGISNRDFVEGVSGGSWVDIVLEHGSCVTTMAKNKPTLDFELIKTEAKQPATLRKYCIEAKLTNTTTESRCPTQGEPSLKEEQDKRFVCKHSIVDRGWGNGCGSFGKGGIVTCAMFTCKKNMEGKIVQPENLEYTIVVTPHSGEEHAIGNDTGKHGKEIKVTPQSSVTEAELTGYGTVTIECSPRTGLDFNEMVLLQMGNKAWLVHRQWFLDLPLPWLPGADTQGSNWIQKETLVTFKNPHAKKQDVVVLGSQEGAMHTALTGATEIQMSSGNLLFTGHLKCGLRMDKLQLKGMSYSMCTGKFKVVKEIAETQHGTIVIRVQYEGDGSPCKIPFEIMDLEKRYVLGRLITVNPIVTEKDSPVNIEAEPPFGDSYIIIGVEPGQLKLNWFKKGSSIGQMFETTMRGAKRMAILGDTAWDFGSLGGVFTSIGKALHQVFGAIYGAAFSGVSWTMKILIGVIITWIGMNSRSTSLSVSLVLVGIVTLYLGVMVQADSGCVVSWKNKELKCGSGIFITDNVRTWTEQYKFQPESPSKLASAIQKAQEEGICGIRSVTRLENLMWKQITPELNHILAENEVKLTIMTGDIKGTMQAGKRSLRPQPTELKYSWKTWGKAKMLSTESHNQTFLIDGPETAECPNTNRAWNSLEVEDYGFGMFTTNIWLQLKEKQDAFCDSKLMSAAIKDNRAVHADMGYWIESALNDTWKIEKASFIEVKNCHWPKSHTLWSNGVLESEMIIPKNLAGPVSQHNYRPGYHTQIAGPWHQGKPEMDFDFCDGTTVVVTEDCGNRGPSLRTTTASGKLITEWCCRSCTLPPLRYRGEDGCWYGMEIRPLKEKEENLVNSLVTAGHGQIDNFSLGVLGMALFLEEMLRTRVGTKHAILLVAVSFVTLITGNMSFKDLGRVVVMVGATMTDDIGMGVTYLALLAAFKVRPTFAAGLLLRKLTSKELMMTTIGIVLLSQSTIPETVLELTDALALGMMALKIVRDMEKYQLAVTIMAILCVPNAVILQNAWKVSCTILAVVSVSPLLLTSSQQKTDWIPLALTIKGLNPTAIFLTTLSRTNKKRSWPLNEAIMAVGMVSILASSLLKNDIPMTGPLVAGGLLTVCYVLTGRSADLELERATDVNWEDQAEISGSSPILSITISEDGSMSIKNEEEEQTLTILIRTGLLVISGLFPVSIPITAAAWYLWEVKKQRAGVLWDVPSPPPMGKAELEDGAYRIKQKGVLGYSQIGAGVYKEGTFHTMWHVTRGAVLMHKGKRIEPSWADVKKDLISYGGGWKLGGEWKEGEEVQVLALEPGKNPRAVQTKPGLFKTNTGTIGAVSLDFSPGTSGSPIIDKKGKVEGLYGNGVVTRSGTYVSAIAQTEKSIEDNPEIEDDIFRKKRLTIMDLHPGAGKTKRYLPAIVREAIKRGLRTLILAPTRVVAAEMEEALRGLPIRYQTPAIRAEHTGREIVDLMCHATFTMRLLSPVRVPNYNLIIMDEAHFTDPASIAARGYISTRVEMGEAAGIFMTATPPGSRDPFPQSNAPIIDEEREIPERSWNSGHEWVTDFKGKTVWFVPSIKAGNDIAACLRKNGKKVIQLSRKTFDSEYVKTRTNDWDFVVTTDISEMGANFKAERVIDPRRCMKPVILTDGEERVILAGPMPVTHSSAAQRRGRIGRNPKNENDQYIYMGEPLENDEDCAHWKEAKMLLDNINTPEGIIPSMFEPEREKVDAIDGEYRLRGEARKTFVDLMRRGDLPVWLAYKVAAEGINYADRRWCFDGIKNNQILEENVEVEIWTKEGERKKLKPRWLDARIYSDPLALKEFKEFAAGRKSLTLNLITEMGRLPTFMTQKTRDALDNLAVLHTAEAGGRAYNHALSELPETLETLLLLTLLATVTGGIFLFLMSGRGIGKMTLGMCCIITASILLWYAQIQPHWIAASIILEFFLIVLLIPEPEKQRTPQDNQLTYVVIAILTVVAATMANEMGFLEKTKKDLGLGSITTQQPESNILDIDLPPASAWTLYAVATTFVTPMLRHSIENSSVNVSLTAIANQATVLMGLGKGWPLSKMDIGVPLLAIGCYSQVNPITLTAALLLLVAHYAIIGPGLQAKATREAQKRAAAGIMKNPTVDGITVIDLDPIPYDPKFEKQLGQVMLLVLCVTQVLMMRTTWALCEALTLATGPISTLWEGNPGRFWNTTIAVSMANIFRGSYLAGAGLLFSIMKNTTNTRRGTGNIGETLGEKWKSRLNALGKSEFQIYKKSGIQEVDRTLAKEGIKRGETDHHAVSRGSAKLRWFVERNMVTPEGKVVDLGCGRGGWSYYCGGLKNVREVKGLTKGGPGHEEPIPMSTYGWNLVRLQSGVDVFFIPPEKCDTLLCDIGEWSPNPTVEAGRTLRVLNLVENWLNNNTQFCIKVLNPYMPSVIEKMETLQRKYGGALVRNPLSRNSTHEMYWVSNASGNIVSSVNMISRMLINRFTMRHKKATYEPDVDLGSGTRNIGIESETPNLDIIGKRIGKIKQEHETSWHYDQDHPYKTWAYHGSYETKQTGSASSMVDGVVRLLTKPWDVIPMVTQMAMTDTTPFGQQRVFKEKVDTRTQEPKEGTKKLMKITAEWLWKELGKKETPRMCTREEFTRKVRSNAALGAIFTDENKWKSAREAVEDSGFWELVDKERNLHLEGKCETCVYNMMGKREKKLGEFGKAKGSRAIWYMWLGARFLEFEALGFLNEDHWFSRENSLSGVEGEGLHKLGYILRDVSKKEGGAMYADDTAGWDTRITLEDLKNEEMVTNHMEGEHKKLAEAIFKLTYQNKVVRVQRPPPRGTVMDIISRRDQRGSGQVVTYGLNTFTNMEAQLIRQMEGEGVFKNIQHLTVTEEIAVQNWLARVGRERLSRMAISGDDCVVKPLDDRFASALTALNDVGKIRKDIQQWEPSRGWDDWTQVPFCSHHSHELIMKDGRVLVVPCRNQDELIGRARISQGAGWSLRETACLGKSYAQMWSLMYFHRRDLRLAANAICSAVPSHWVPTSRTTWSIHAKHEWMTTEDMLTVWNRVWIQENPWMEDKTPVESWEEIPYLGKREGQWCGSLIGLTSRATWAKNIQTAINQVRSLIGNEEYTDYMPSMKRFRREEEEAGVLW

>DENV2_AIE17400_Pakistan

MNNQRKKARNTPFNMLKRERNRVSTVQQLTKRFSLGMLQGRGPLKLFMALVAFLRFLTIPPTAGILKRWGTIKKSKAINVLRGFRKEIGRMLNILNRRRRTAGVIIMLIPTAMAFHLTTRNGEPHMIVSKQEKGKSLLFKTEDGVNMCTLMAMDLGELCEDTITYNCPLLRQNEPEDIDCWCNATSTWVTYGTCTATGEHRREKRSVALVPHVGMGLETRTETWMSSEGAWKHAQRIETWILRHPGFTIMAAILAYTIGTTYFQRVLIFILLTAVAPSMTMRCIGISNRDFVEGVSGGSWVDIVLEHGSCVTTMAKNKPTLDFELIKTEAKQPATLRKYCIEAKLTNTTTASRCPTQGEPSLNEEQDKRFVCKHSMVDRGWGNGCGLFGKGGIVTCAMFTCKKNMEGKIVQPENLEYTIVVTPHSGEENAVGNDTGKHGKEIKVTPQSSITEAELTGYGTVTMECSPRTGLDFNEMVLLQMENKAWLVHRQWFLDLPLPWLPGADIQGSNWIQKETLVTFKNPHAKKQDVVVLGSQEGAMHTALTGATEIQMSSGNPLFTGHLKCRLRMDKLQLKGMSYSMCTGKFKVVKEIAETQHGTIVVRIQYEGDGSPCKIPFEIMDLEKRHVLGRLITVNPIVTEKDSPVNIEAEPPFGDSYIIIGVEPGQLKLSWFKKGSSIGQMFETTMRGAKRMAILGDTAWDFGSLGGVFTSIGKALHQVFGAIYGAAFSGVSWTMKILIGVVITWIGMNSRSTSLSVSLVLVGVVTLYLGVMVQADSGCVVSWKNKELKCGSGIFFTDNVHTWTEQYKFQPESPSKLASAIQKAHEEGICGIRSITRLDNLMWKQITPELNHILSENEVKLSIMTGDIKGIMQAGKRSLRPQPTELKYSWKTWGKAKMLSTEPHNQTFLIDGPETAQCPNTNRAWNSLEVEDYGFGVFTTNIWLKLKERQDVFCDSKLMSAAIKDNRAVHADMGYWIESALNDTWKIEKASFIEVKSCHWPKSHTLWSNGVLESEMIIPKNFAGPVSQHNYRPGYHTQTAGPWHLGKLEMDFDFCEGTTVVVTEDCGNRGPSLRTTTASGKLITEWCCRSCTLPPLRYRGEDGCWYGMEIRPLKEKEENLVNSLVTAGHGQIDNFSLGVLGMALFLEEMLRTRVGTKHAVLLVAVSFMTLITGNMSFRDLGRVMVMVGAAMTDDIGMGVTYLALLAAFKVRPTFAAGLLLRKLTSKELMMTTIGIVLLSQSTTPETILELTDALALGMMILKIVRNMEKYQLAVTIMAILCVPNAVILQNAWKVSCTILAAVSVSPLLLTSSQQKADWIPLALTIKGLNPTAIFLTTLSRTNKKRSWPLNEAIMAVGMVSILASSLLKNDIPMTGPLVAGGLLTVCYVLTGRSADLELERAADVRWEDQAEISGSSPILSITISEDGSMSIKNEEEEQTLTILIRTGLLVISGLFPVSIPITAAAWYLWEVKKQRAGVLWDVPSPPPVGKAELEDGAYRIKQKGILGYSQIGAGVYKEGTFHTMWHVTRGAVLMHKGKRIEPSWADVRKDLISYGGGWKLEGEWKEGEEVQVLALEPGKNPRAVQTKPGLFKTNTGTIGAVSLDFSPGTSGSPIVDKKGKVVGLYGNGVVTRSGTYVSAIAQTEKSIEDNPEIEDDIFRKKRLTIMDLHPGAGKTKRYLPAIVREAIKRGLRTLILAPTRVVAAEMEEALRGLPIRYQTPAIRAEHTGREIVDLMCHATFTMRLLSPVRVPNYNLIIMDEAHFTDPASIAARGYISTRVEMGEAAGIFMTATPPGSRDPFPQSNAPIMDEEREIPERSWNSGHEWVTDFKGKTVWFVPSIKAGNDIAACLRKNGKKVIQLSRKTFDSEYIKTRTNDWDFVVTTDISEMGANFKAERVIDPRRCMKPVILTDGEERVILAGPMPVTHSSAAQRRGRIGRNPKNENDQYIYMGEPLENDEDCAHWKEAKMLLDNINTPEGIIPSMFEPEREKVDAIDGEYRLRGEARKTFVDLMRRGDLPVWLAYRVAAEGINYADRRWCFDGIKNNQILEENVEVEIWTKEGERKKLKPRWLDARIYSDPLALKEFKEFAAGRKSLTLNLITEMGRLPTFMTQKARNALDNLAVLHTAEAGGRAYNHALSELPETLETLLLLTLLATVTGGIFLFLMSGKGIGKMTLGMCCIITASILLWYAQIQPHWIAASIILEFFLIVLLIPEPEKQRTPQDNQLTYVVIAILTVVAATMANEMGFLEKTKKDFGLGSIATQQPESNILDIDLRPASAWTLYAVATTFITPMLRHSIENSSVNVSLTAIANQATVLMGLGKGWPLSKMDIGVPLLAIGCYSQVNPITLTAALLLLVAHYAIIGPGLQAKASREAQKRAAAGIMKNPTVDGITVIDLDPIPYDPKFEKQLGQVMLLILCVTQVLMMRTTWALCEALTLATGPISTLWEGNPGRFWNTTIAVSMANIFRGSYLAGAGLLFSIMKNTTNTRRGTGNTGETLGEKWKNRVSLLGKSEFQIYKRSGIQEVDRTLAKEGIKRGETDHHAVSRGSAKLRWFVERNLVTPEGKVVDLGCGRGGWSYYCGGLKNVREVKGLTKGGPGHEEPIPMSTYGWNLVRLLTGADVFFTPPEKCDTLLCDIGESSPNPTVEAGRTLRVLNLVENWLNNNTQFCIKVLNPYMPSVIEKMETLQRKYGGALVRNPLSRNSTHEMYWVSNASGNIVSSVNMISRMLINRFTMRHKKATYDPDVDLGSGTRNIGIESETPNLDIIGKRIEKIKQEHETSRHYDQDHPYKTWAYHGSYETKQTGSASSTVNGVVRLLTKPWDVIPMVTQMAMTDTTPFGQQRVFKEKVDTRTQEPKEGTKKLMKITAEWLWKELGKKKTPRMCTREEFARKVRSNAALGAIFTDENKWKSAREAVEDSRFWELVDKERNLHLEGKCETCVYNMMGKREKKIGEFGKAKGSRAIWYMWLGARFLEFEALGFLNEDHWFSRENSLSGVEGEGLHKLGYILREVSKKEGGAMYADDTAGWDTRITLEDLKNEEMVTNHMEGEHKKLAEAIFKLTYQNKVVRVQRPTPRGTVMDIISRRDQRGSVQVGTYGLGTFTNMGAQLIRQMEGEGVFKGIQHLTATEEVAVQDWLARVGRERLSRMAISGDDCVVKPLDDRFASALTALNDMGKVRKDIQQWEPSRGWNDWTQVPFCSHHFHELIMKDGRVLVVPCRNQDELIGRARISQGAGWSLRETACLGKSYAQMWSLMYFHRRDLRLAANAICSAVPSHWVPTSRTTWSIHAGHEWMTTEDMLTVWNRVWIQENPWMEDKTPVESWEEIPYLGKREDQWCGSLIGLTSRATWARNIQTAINQVRSLIGSEEYTDYMPSMKRFGREEEEAGVLW

>DENV2_AAF18447_China

MNNQRKKARNTPFNMLKRERNRVSTVQQLTKRFSLGMLQGRGPLKLFMALVAFLRFLTIPPTAGILKRWGTIKKSKAINVLRGFRKEIGRMLNILNRRRRTAGMIIMLIPTVMAFHLTTRNGEPHMIVSRQEKGKSLLFKTGDGVNMCTLMAMDPGELCEDTITYKCPLLKQNEPEDIDWWCNSTSTWVTYGTCTTTGEHRREKRSVALVPHVGMGLETRTETWMSSEGAWKHAQRIETWILRHPGFTMMAAILAYTIGTTHFQRALIFILLTAVAPSMTMRCIGISNRDFVEGVSGGSWVDIVLEHGSCVTTMAKNKPTLDFELIKTEAKQPATLRKYCIEAKLTNTTTDSRCPTQGEPSLNEEQDKRFVCKHSMVDRGWGNGCGLFGKGGIVTFAMFTCKKNMKGKVVQPENLEYTIVITPHSGEEHAVGNDTGKHGKEIKITPQSSITEAELTGYGTVTMKCSPRTGLDFNEMVLLQMENKAWLVHRQWFLDLPLPWLPGADTQGSNWIQKETLVTFKNPHAKKQDVVVLGSQEGAMHTALTGATEIQMSSGNLLFTGHLKCRLRMDKLQLKGMSYSMCTGKFKVVKEIAETQHGTIVIRVQYEGDGSPCKIPFEIMDLEKRHVLGRLITVNPIVTEKDSPVNIEAEPPFGDSYIIIGVEPGQLKLNWFKKGSSIGQMFETTMRGAKRMAILGDTAWDFGSLGGVFTSIGKALHQVFGAIYGAAFSGVSWTMKILIGVIITWIGMNSRSTSLSVSLVLVGVVTLYLGVMVQADSGCVVSWKNKELKCGSGIFITDDVHTWTEQYKFQPESPSKLASAIQKAHEEGICGIRSVTRLENLMWKQITPELNHILSENEVKLTIMTGDIKGIMQAGKRSLRPQPTELKYSWKTWGKAKMLSTESHNQTFLIDGPETAECPNTNRAWNSLEVEDYGFGVFTTNIWLKLREKQDVFCDSKLMSAVIKDNRAAHADMGYWIESALNDTWKIEKASFIEVKSCHWPKSHTLWSNGVLESEMIIPKNFAGPVSQHNYRPGYHTQTAGPWHLGKLEMDFDFCEGTTVVVTEDCGNRGPSLRTTTASGKLITEWCCRSCTLPPLRYRGEDGCWYGMEIRPLKEKEENLVNSLVTAGHGQIDNFSLGVLGMALFLEEMLRTRVGTKHAILLVAVSFVTLITGNMSFRDLGRVMVMVGATMTDDIGMGVTYLALLAAFKVRPTFAAGLLLRKLTSKELMMTTIGIVLLSQSTIPETILELTDALALGMMVLKMVRKMEKYQLAVTIMAILCVPNAVILQNAWKVSCTILAVVSVSPLFITSSQQKADWIPLALTIKGLNPTAIFLTTLSRTNKKRSWPLNEAIMAVGMVSILASSLLKNDIPMTGPLVAGGLLTVCYVLTGRSADLELERAADVKWEDQAEISGSSPILSITISEDGSMSIKNEEEEQILTILIRTGLLVISGLFPVSIPITAAAWYLWEVKKQRAGVLWDVPSPPPVGKAELEDGAYRIKQKGILGYSQIGAGVYKEGTFHTMWHVTRGAVLMHKGKRIEPSWADVKKDLISYGGGWKLEGEWKEGEEVQVLALEPGKNPRAVQTKPGLFKTNAGTIGAVSLDFSPGTSGSPIIDKKGKVVGLYGNGVVTRSGAYVSAIAQTEKSIEDNPEIEDDIFRKRKLTIMDLHPGAGKTKRYLPAIVREAIKRGLRTLILAPTRVVAAEMEEALRGLPIRYQTPAIRAEHTGREIVDLMCHATFTMRLLSPVRVPNYNLIIMDEAHFTDPASIAARGYISTRVEMGEAAGIFMTATPPGSRDPFPQSNAPIMDEEREIPERSWSSGHEWVTDFKGKTVWFVPSIKAGNDIAACLRKNGKKVIQLSRKTFDSEYVKTRTNDWDFVVTTDISEMGANFKAERVIDPRRCMKPVILTDGEERVILAGPMPVTHSSAAQRRGRIGRNPKNENDQYIYMGEPLENDEDCAHWKEAKMLLDNINTPEGIIPSMFEPEREKVDAIDGEYRLRGEARKTFVDLMRRGDLPVWLAYRVAAEGINYADRRWCFDGIKNNQILEENVEVEIWTKEGERKKLKPRWLDAKIYSDPLALKEFKEFAAGRKSLTLNLITEMGRLPTFMTQKARDALDNLAVLHTAEAGGRAYNHALSELPETLETLLLLTLLATVTGGIFLFLMSGRGIGKMTLGMCCIITASILLWYAQIQPHWIAASIILEFFLIVLLIPEPEKQRTPQDNQLTYVVIAILTVVAATMANEMGFLEKTKKDLGLGSITTQQPESNILDIDLRPASAWTLYAVATTFVTPMLRHSIENSSVNVSLTAIANQATVLMGLGKGWPLSKMDIGVPLLAIGCYSQVNPITLTAALFLLVAHYAIIGPGLQAKATREAQKRAAAGIMKNPTVDGITVIDLDPIPYDPKFEKQLGQVMLLVLCVTQVLMMRTTWALCEALTLATGPISTLWEGNPGRFWNTTIAVSMANIFRGSYLAGAGLLFSIMKNTTNTRRGTGNIGETLGEKWKIRLNALGKSEFQIYKKSGIQEVDRTLAKEGIKRGETDHHAVSRGSAKLRWFVERNMVTPEGKVVDLGCGRGGWSYYCGGLKNVREVKGLTKGGPGHEEPIPMSTYGWNLVRLQSGVDVFFTPPEKCDTLLCDIGESSPNPTVEAGRTLRVLNLVENWLNNNTQFCIKVLNPYMPSVIEKMETLQRKYGGALVGNPLSRNSTHEMYWVSNASGNIVSSVNMISRMLINRFTMRHKKATYEPDVDLGSGTRNIGIESEIPNLDIIGKRIEKIKQEHETSWHYDQDHPYKTWAYHGSYETKQTGSASSMVNGVVRLLTKPWDVVPMVTQMAMTDTTPFGQQRVFKEKVDTRTQEPKEGTKKLMKITAEWLWKELGKKKTPRMCTREEFTRKVRSNAALGAIFTDENKWKSAREAVEDSRFWELVDKERNLHLEGKCETCVYNMMGKREKKLGEFGKAKGSRAIWYMWLGARFLEFEALGFLNEDHWFSRENSLSGVEGEGLYKLGYILRDVSKKEGGAMYADDTAGWDTRITLEDLKNEEMVTNHMEGEHKKLAEAIFKLTYQNKVVRVQRPTPRGTVMDIISRRDQRGSGQVGTYGLNTFTNMEAQLIRQMEGEGVFKSIQHLTVTEEIAVQNWLARVRRERLSRMAISGDDCVVKPLDDRFASALTALNDMGKVRKDIQQWEPSRGWNDWTQVPFCSHHFHELIMKDGRVLVVPCRNQDELIGRARISQGAGWSLRETACLGKSYAQMWSLMYFHRRDLRLAANAICSAVPSHWVPTSRTTWSIHAKHEWMTTEDMLTVWNRVWIQENPWMEDKTPVESWEEIPYLGKREDQWCGSLIGLTSRATWAKNIQTAINQVRSLIGNEEYTDYMPSMKRFRREEEEAGVLW

>DENV2_ ATO98049_Tanzania

MNNQRKKARNTPFNMLKRERNRVSTVQQLTKRFSLGMLQGRGPLKLFMALVAFLRFLTIPPTAGILKRWGTIKKSKAINVLRGFRKEIGRMLNILNRRRRTAGIIIMMIPTVMAFHLTTRNGEPHMIVSRQEKGKSLLFKTENGVNMCTLMAMDLGELCEDTITYNCPLLRQNEPEDIDCWCNSTSTWVTYGTCTATGEHRREKRSVALVPHVGMGLETRTETWMSSEGAWKHAQRIETWVLRHPGFTIMAAILAYTIGTTYFQRVLIFILLTAVAPSMTMRCIGISNRDFVEGVSGGSWVDIVLEHGSCVTTMAKNKPTLDFELIKTEAKHPATLRKYCIEAKLTNTTTASRCPTQGEPSLNEEQDKRFVCKHSMVDRGWGNGCGLFGKGGIVTCAMFTCKKNMEGKIVQPENLEYTIVITPHSGEENAVGNDTGKHGKEIKVTPQSSITEAELTGYGTVTMECSPRTGLDFNEMVLLQMENKAWLVHRQWFLDLPLPWLPGADTQGSNWIQKETLVTFKNPHAKKQDVVVLGSQEGAMHTALTGATEIQMSSGNLLFTGHLKCRLRMDKLQLKGMSYSMCTGKFKVVKEIAETQHGTIVIRVQYEGDGSPCKIPFEIMDLEKRHVLGRLITVNPIVTEKDSPVNIEAEPPFGDSYIIIGVEPGQLKLSWFKKGSSIGQMFETTMRGAKRMAILGDTAWDFGSLGGVFTSIGKALHQVFGAIYGAAFSGVSWTMKILIGVVITWIGMNSRSTSLSVSLVLVGVVTLYLGVMVQADSGCVVSWKNKELKCGSGIFITDNVHTWTEQYKFQPESPSKLASAIQKAHEEGICGIRSVTRLENLMWKQITPELNHILSENEVKLTIMTGDIKGIMQAGKRSLRPQPTELKYSWKTWGKAKMLSTELHNHTFLIDGPETAECPNTNRAWNSLEVEDYGFGVFTTNIWLKLKERQDVFCDSKLMSAAIKDNRAVHADMGYWIESALNDTWKIEKASFIEVKSCHWPKSHTLWSNGVLESEMIIPKNFAGPVSQHNYRPGYHTQTAGPWHLGRLEMDFDFCEGTTVVVTEDCGNRGPSLRTTTASGKLITEWCCRSCTLPPLRYRGEDGCWYGMEIRPLKEKEENLVNSLVTAGHGQIDNFSLGVLGMALFLEEMLRTRVGTKHAILLVAVSFVTLITGNMSFRDLGRVMVMVGTAMTDDIGMGVTYLALLAAFKVRPTFAAGLLLRKLTSKELMMTTIGIVLLSQSTMPETILELTDALALGMMVLKIVRNMEKYQLAVTIMAILCVPNAVILQNAWKVSCTTLAVVSVSPLLLTSSQQKADWIPLALTIKGLNPTAIFLTTLSRTSKKRSWPLNEAIMAVGMVSILASSLLKNDISMTGPLVAGGLLTVCYVLTGRSADLELERAADVRWEEQAEISGSSPILSITISEDGSMSIKNEEEEQTLTILIRTGLLVISGLFPVSIPITAAAWYLWEVKKQRAGVLWDVPSPPPVGKAELEDGAYRIKQKGILGYSQIGAGVYKEGTFHTMWHVTRGAVLMHKGKRIEPSWADVKKDLISYGGGWKLEGEWKEGEEVQVLALEPGKNPRAVQTKPGLFKTNTGTIGAVSLDFSPGTSGSPIVDKKGKVVGLYGNGVVTRSGTYVSAIAQTEKSIEDNPEIEDDIFRKKRLTIMDLHPGAGKTKRYLPAIVREAIKRGLRTLILAPTRVVAAEMEEALRGLPIRYQTPAIRAEHTGREIVDLMCHATFTMRLLSPIRVPNYNLIIMDEAHFTDPASIAARGYISTRVEMGEAAGIFMTATPPGSRDPFPQSNAPIMDEEREIPERSWNSGHEWVTDFKGKTVWFVPSIKAGNDIAACLRKNGKKVIQLSRKTFDSEYIKTRTNDWDFVVTTDISEMGANFKAERVIDPRRCMKPVILTDGEERVILAGPMPVTHSSAAQRRGRVGRNPKNENDQYIYMGEPLENDEDCAHWKEAKMLLDNINTPEGIIPSMFEPEREKVDAIDGEYRLRGEARKTFVDLMRRGDLPVWLAYRVAAEGINYADRRWCFDGVKNNQILEENVEVEIWTKEGERKKLKPRWLDARIYSDPLALKEFKEFAAGRKSLTLNLITEMGRLPTFMTQKARNALDNLAVLHTAEAGGRAYNHALSELPETLETLLLLTLLAAVTGGIFLFLMSGKGIGKMTLGMCCIITASILLWYAQIQPHWIAASIILEFFLIVLLIPEPEKQRTPQDNQLTYVVIAILTVVAATMANEMGFLEKTKKDFGLGSTTTQQPESNILDIDLRPASAWTLYAVATTFITPMLRHSIENSSVNVSLTAIANQATVLMGLGKGWPLSKMDIGVPLLAIGCYSQVNPITLIAALLLLVAHYAIIGPGLQAKATREAQKRAAAGIMKNPTVDGITVIDLDPIPYDPKFEKQLGQVMLLVLCVTQVLMMRTTWALCEALTLATGPISTLWEGNPGRFWNTTIAVSMANIFRGSYLAGAGLLFSIMKNTANTRRGTGNTGETLGEKWKNRLNALGKSEFQIYKKSGIQEVDRTLAKEGIKRGETDHHAVSRGSAKLRWFVERNLVTPEGKVVDLGCGRGGWSYYCGGLKNVKEVKGLTKGGPGHEEPIPMSTYGWNLVRLQSGVDVFFTPPEKCDTLLCDIGVSSPNPTVEAGRTLRVLNLVENWLNNNTQFCIKVLNPYMPSVIEKMEALQRKYGGALVRNPLSRNSTHEMYWVSNASGNIVSSVNMISRMLINRFTMRHKKATYEPDVDLGSGTRNIGIESETPNLDIIGKRIEKIKQEHETSWHYDQDHPYKTWAYHGSYETKQTGSASSMVNGVVRLLTKPWDVIPMVTQMAMTDTTPFGQQRVFKEKVDTRTQEPKEGTKKLMKITAEWLWKELGKKKTPRMCTREEFTRKVRSNAALGAIFTDENKWKSAREAVEDSGFWELVDKERNLHLEGKCETCVYNMMGKREKKLGEFGKAKGSRAIWYMWLGARFLEFEALGFLNEDHWFSRENSLSGVEGEGLHKLGYILRDVSKKEGGAMYADDTAGWDTRITLEDLKNEEMVTNHMEGEHKKLAEAIFKLTYQNKVVRVQRPTPRGTVMDIISRRDQRGSGQVVTYGLNTFTNMEAQLIRQMEGEGVFKSIQQLTVTEEIAVKNWLARVGRERLSRMAISGDDCVVKPLDDRFASALTALNDMGKVRKDIQQWEPSRGWNDWTQVPFCSHHFHELIMKDGRVLVVPCRNQDELIGRARISQGAGWSLRETACLGKSYAQMWSLMYFHRRDLRLAANAICSAVPSHWVPTSRTTWSIHATHEWMTTEDMLTVWNRVWIQENPWMEDKTPVESWEEIPYLGKREDQWCGSLIGLTSRATWAKNIQTAINQVRSLIGNEEYTDYMPSMKRFRREEEEAGVLW

>DENV2_ARM59246_Singapore

MNNQRKKARNTPFNMLKRERNRVSTVQQLTKRFSLGMLQGRGPLKLFMALVAFLRFLTIPPTAGILKRWGTIKKSKAINVLRGFRKEIGRMLNILNRRRRTAGIIIMMIPTVMAFHLTTRNGEPHMIVSRQEKGKSLLFKTENGVNMCTLMAMDLGELCEDTITYNCPLLRQNEPEDIDCWCNSTSTWVTYGTCTATGEHRREKRSVALVPHVGMGLETRTETWMSSEGAWKHAQRIETWVLRHPGFTIMAAILAYTIGTTYFQRVLIFILLTAVAPSMTMRCIGISNRDFVEGVSGGSWVDIVLEHGSCVTTMAKNKPTLDFELIKTEAKHPATLRKYCIEAKLTNTTTASRCPTQGEPSLNEEQDKRFVCKHSMVDRGWGNGCGLFGKGGIVTCAMFTCKKNMEGKIVQPENLEYTIVITPHSGEENAVGNDTGKHGKEIKVTPQSSITEAELTGYGTVTMECSPRTGLDFNEMVLLQMENKAWLVHRQWFLDLPLPWLPGADTQGSNWIQKETLVTFKNPHAKKQDVVVLGSQEGAMHTALTGATEIQMSSGNLLFTGHLKCRLRMDKLQLKGMSYSMCTGKFKVVKEIAETQHGTIVIRVQYEGDGSPCKIPFEIMDLEKRHVLGRLITVNPIVTEKDSPVNIEAEPPFGDSYIIIGVEPGQLKLSWFKKGSSIGQMFETTMRGAKRMAILGDTAWDFGSLGGVFTSIGKALHQVFGAIYGAAFSGVSWTMKILIGVVITWIGMNSRSTSLSVSLVLVGVVTLYLGVMVQADSGCVVSWKNKELKCGSGIFITDNVHTWTEQYKFQPESPSKLASAIQKAHEEGICGIRSVTRLENLMWKQITPELNHILTENEVKLTIMTGDIKGIMQAGKRSLRPQPTELKYSWKAWGKAKMLSTELHNHTFLIDGPETAECPNTNRAWNSLEVEDYGFGVFTTNIWLKLKERQDVSCDSKLMSAAIKDNRAVHADMGYWIESALNDTWKIEKASFIEVKSCHWPKSHTLWSNGVLESEMIIPKNFAGPVSQHNYRPGYHTQTAGPWHLGRLEMDFDFCEGTTVVVTEDCGNRGPSLRTTTASGKLITEWCCRSCTLPPLRYRGEDGCWYGMEIRPLKEKEENLVNSLVTAGHGQIDNFSLGVLGMALFLEEMLRTRVGTKHAILLVAVSFVTLITGNMSFRDLGRVMVMVGATMTDDIGMGVTYLALLAAFKVRPTFAAGLLLRKLTSKELMMTTIGIVLLSQSTIPETILELTDALALGMMVLKIVRNMEKYQLAVTIMAILCVPNAVILQNAWKVSCTALAVVSVSPLLLTSSQQKTDWIPLALTIKGLNPTAIFLTTLSRTSKKRSWPLNEAIMAVGMVSILASSFLKNDIPMTGPLVAGGLLTVCYVLTGRSADLELERAADIRWEEQAEISGSSPILSITISEDGSMSIKNEEEEQTLTILIRTGLLVISGLFPVSIPITAAAWYLWEVKKQRAGVLWDVPSPPPVGRAELEDGAYRIKQKGILGYSQIGAGVYKEGTFHTMWHVTRGAVLMHKGKRIEPSWADVKKDLISYGGGWKLEGEWKEGEEVQVLALEPGKNPRAVQTKPGLFKTNTGTIGAVSLDFSPGTSGSPIVDKKGKVVGLYGNGVVTRSGTYVSAIAQTEKSIEDNPEIEDDIFRKKRLTIMDLHPGAGKTKRYLPAIVREAIKRGLRTLILAPTRVVAAEMEEALRGLPIRYQTPAIRAEHTGREIVDLMCHATFTMRLLSPIRVPNYNLIIMDEAHFTDPASIAARGYISTRVEMGEAAGIFMTATPPGSRDPFPQSNAPIMDEEREIPERSWNSGHEWVTDFKGKTVWFVPSIKAGNDIAACLRKNGKKVIQLSRKTFDSEYIKTRTNDWDFVVTTDISEMGANFKAERVIDPRRCMKPVILTDGEERVILAGPMPVTHSSAAQRRGRVGRNPKNENDQYIYMGEPLENDEDCAHWKEAKMLLDNINTPEGIIPSMFEPEREKVDAIDGEYRLRGEARKTFVDLMRRGDLPVWLAYRVAAEGINYADRRWCFDGVKNNQILEENVEVEIWTKEGERKKLKPRWLDARIYSDPLALKEFKEFAAGRKSLTLNLITEMGRLPTFMTQKARNALDNLAVLHTAEAGGRAYNHALSELPETLETLLLLTLLATVTGGIFLFLMSGKGIGKMTLGMCCIVTASILLWYAQIQPHWIAASIILEFFLIVLLIPEPEKQRTPQDNQLTYVVIAILTVVAATMANEMGFLEKTKKDFGLGSITTQQPESNILDIDLRPASAWTLYAVATTFITPMLRHSIENSSVNVSLTAIANQATVLMGLGKGWPLSKMDIGVPLLAIGCYSQVNPITLTAALLLLVAHYAIIGPGLQAKATREAQKRAAAGIMKNPTVDGITVIDLDPIPYDPKFEKQLGQVMLLVLCVTQVLMMRTTWALCEALTLATGPISTLWEGNPGRFWNTTIAVSMANIFRGSYLAGAGLLFSIMKNTANTRRGTGNTGETLGEKWKNRLNALGKSEFQIYKKSGIQEVDRTLAKEGIKRGETDHHAVSRGSAKLRWFVERNLVTPEGKVVDLGCGRGGWSYYCGGLKNVKEVKGLTKGGPGHEEPIPMSTYGWNLVRLQSGVDVFFTPPEKCDTLLCDIGESSPNPTVEAGRTLRVLNLVENWLNNNTQFCIKVLNPYMPSVIEKMEALQRKYGGALVRNPLSRNSTHEMYWVSNASGNIVSSVNMISRMLINRFTMRHKKATYEPDVDLGSGTRNIGIESEVPNLDIIGKRIEKIKQEHETSWHYDQDHPYKTWAYHGSYETKQTGSASSMVNGVVRLLTKPWDVIPMVTQMAMTDTTPFGQQRVFKEKVDTRTQEPKEGTKKLMKITAEWLWKELGKKKTPRMCTREEFTRKVRSNAALGAIFTDENKWKSAREAVEDSGFWELVDKERNLHLEGKCETCVYNMMGKREKKLGEFGKAKGSRAIWYMWLGARFLEFEALGFLNEDHWFSRENSLSGVEGEGLHKLGYILRDVSKKEGGAMYADDTAGWDTRITLEDLKNEEMVTNHMEGEHKKLAEAIFRLTYQNKVVRVQRPTSRGTVMDIISRRDQRGSGQVVTYGLNTFTNMEAQLIRQMEGEGVFKSIQHLTATEEIAVKNWLVRVGRERLSRMAISGDDCVVKPLDDRFANALTALNDMGKVRKDIQQWEPSRGWNDWTQVPFCSHHFHELIMKDGRVLVVPCRNQDELIGRARISQGAGWSLRETACLGKSYAQMWSLMYFHRRDLRLAANAICSAVPSHWVPTSRTTWSIHATHEWMTTEDMLTVWNRVWIQENPWMEDKTPVESWEEIPYLGKREDQWCGSLIGLTSRATWAKNIQTAINQVRSLIGNEEYTDYMPSMKRFRREEEEAGVLW

>DENV2_AQV12084_Burkina_Faso

MNNQRKKARSTPFNMLKRERNRVSTVQQLTKRFSLGMLQGRGPLKLFMALVAFLRFLTIPPTAGILKRWGTIKKSKAINVLRGFRKEIGRMLNILNRRRRTAGIIIMLIPTVMTFHLTTRNGEPHMIVSRQEKGKSLLFKTEDGVNMCTLMAMDLGELCEDTITYNCPLLRQNEPEDIDCWCNSTSTWVTYGTCTATGEHRREKRSVALVPHVGMGLETRTETWMSSEGAWKHAQRIETWILRHPGFTIMAAILAYTIGTTHFQRVLIFTLLTAVAPSMTMRCIGISNRDFVEGVSGGSWVDIVLEHGSCVTTMAKNKPTLDFELIKTEAKQPATLRKYCIEAKLTNTTTASRCPTQGEPSLNEEQDKRFVCKHSMVDRGWGNGCGLFGKGGIVTCAMFTCKKNMEGKVVQPENLEYTIVITPHSGEENAVGNDTGKHGKEIKITPQSSITEAELTGYGTVTMECSPRTGLDFNEMVLLQMENKAWLVHRQWFLDLPLPWLPGADTQGSNWIQKETLVTFKNPHAKKQDVVVLGSQEGAMHTALTGATEIQMSSGNLLFTGHLKCRLRMDKLQLKGMSYSMCTGKFKVVKEIAETQHGTIVVRIQYEGDGSPCKIPFEIMDLEKRHVLGRLITVNPIVTEKDSPVNIEAEPPFGDSYIIIGVEPGQLKLNWFKKGSSIGQVFETTMRGAKRMAILGDTAWDFGSLGGVFTSIGKALHQVFGAIYGAAFSGVSWTMKILIGVVITWIGMNSRSTSLSVSLVLVGVVTLYLGVVVQADSGCVVSWKNKELKCGSGIFVTDNVHTWTEQYKFQPESPSKLASAIQKAHEEGICGIRSVTRLENLMWKQITPELNHILSENEVKLTIMTGDIKGIMQTGKRSLRPQPTELKYSWKTWGKAKMLSTELHNQTFLIDGPETAECPNINRAWNSLEVEDYGFGVFTTNIWLKLKERQDVFCDSKLMSAAIKDNRAVHADMGYWIESALNDTWKIEKASFIEIKSCHWPKSHTLWSNGVLESEMIIPKNLAGPVSQHNYRPGYHTQTAGPWHLGKLEVDFNFCEGTTVVVTEDCGNRGPSLRTTTASGKLITEWCCRSCTLPPLRYRGEDGCWYGMEIRPLKEKEENLVNSLVTAGHGQIDNFSLGVLGMALFLEEMLRTRVGTKHAILLVAVSFVTLITGNMSFRDLGRVMVMVGATMTDDIGMGVTYLALLAAFKVRPTFAAGLLLKKLTSRELMMTTIGIVLLSQSTIPETILELTDALALGMMVLKIVRNMEKYQLAATIMAILCVPNAVILQNAWKVSCTILAVVSVSPLLLTSSQQKADWIPLALTIKGLNPTAIFLTTLSRTSKKRSWPLNEAIMAVGMVSILASSLLKNDIPMTGPLVAGGLLTVCYVLTGRSADLELERAADVRWEDQAEISGSSPILSITISEDGSMSIKNEEEEQTLTILIRTGLLVISGLFPVSIPITAAAWYLWEVKKQRAGVLWDVPSPPPVGKAELEDGAYRIKQKGILGYSQIGAGVYKEGTFHTMWHVTRGAVLMHKGKRIEPSWADVKKDLISYGGGWKLEGEWKEGEEVQVLALEPGKNPRAVQTKPGLFKTNTGTIGAVSLDFSPGTSGSPIVDKKGKVVGLYGNGVVTRSGTYVSAIAQTEKSIEDNPEIEDDIFRKKRLTIMDLHPGAGKTKRYLPAIVREAIKRGLRTLILAPTRVVAAEMEEALRGLPIRYQTPAIRAEHTGREIVDLMCHATFTMRLLSPVRVPNYNLIIMDEAHFTDPASIAARGYISTRVEMGEAAGIFMTATPPGSRDPFPQSNAPIMDEEREIPERSWNSGHEWVMDFKGKTVWFVPSIKAGNDIAACLRKNGKKVIQLSRKTFDSEYIKTRTNDWDFVVTTDISEMGANFKAERVIDPRRCMKPVILTDGEERVILAGPMPVTHSSAAQRRGRIGRNPKNENDQYIYMGEPLENDEDCAHWKEAKMLLDNINTPEGIIPSMFEPEREKVDAIDGEYRLRGEARKTFVDLMRRGDLPVWLAYRVAAEGINYADRRWCFDGIKNNQILEENVEVEIWTKEGERKKLKPRWLDARIYSDPLALKEFKEFAAGRKSLTLNLITEMGRLPTFMTQKARNALDNLAVLHTAEAGGRAYNHALSELPETLETLLLLTLLATVTGGIFLFLMSGKGIGKMTLGMCCIITASILLWYAQIQPHWIAASIILEFFLIVLLIPEPEKQRTPQDNQLTYVVIAILTMVAATMANEMGFLEKTKKDFGLGSVATQQPESNILDIDLRPASAWTLYAVATTFITPMLRHSIENSSVNVSLTAIANQATVLMGLGKGWPLSKMDIGVPLLAIGCYSQVNPITLTAALFLLVAHYAIIGPGLQAKATREAQKRAAAGIMKNPTVDGITVIDLDPIPYDPKFEKQLGQVMLLVLCVTQVLMMRTTWALCEALTLATGPISTLWEGNPGRFWNTTIAVSMANIFRGSYLAGAGLLFSIMKNTANTRRGTGNTGETLGEKWKNRLNTLGKSEFQAYKKSGIQEVDRTLAKEGIKRGETDHHAVSRGSAKLRWFVERNLVTPEGKVVDLGCGRGGWSYYCGGLKNVREVKGLTKGGPGHEEPIPMSTYGWNLVRLQSGVDVFFTPPEKCDTLLCDIGESSPNPTIEAGRTLRVLNLVENWLNNNTQFCIKVLNPYMPSVIEKMEALQRKYGGALVRNPLSRNSTHEMYWVSNASGNIVSSVNMISRMLINRFTMRHKKATYEPDVDLGSGTRNIGIENETPNLDIIGKRIEKIKQEHETSWHYDQDHPYKTWAYHGSYETKQTGSASSMVNGVVRLLTKPWDVIPTVTQMAMTDTTPFGQQRVFKEKVDTRTQEPKEGTKKLMKITAEWLWKELGKKKTPRMCTREEFTRKVRSNAALGAIFTDENKWKSAREAVEDSRFWELVDKERNLHLEGKCETCVYNMMGKREKKLGEFGKAKGSRAIWYMWLGARFLEFEALGFLNEDHWFSRENSLSGVEGEGLHKLGYILRDVSKKEGGAMYADDTAGWDTRITLEDLKNEEMVTNHMEGEHRKLAEAIFRLTYQNKVVRVQRPTPRGTVMDIISRRDQRGSGQVGTYGLNTFTNMGAQLIRQMEGEGVFKSIQHLTVTEEITVQNWLARVGRERLSRMAISGDDCVVKPLDDRFASALTALNDMGKVRKDIQQWEPSKGWNDWTQVPFCSHHFHELIMKDGRVLVVPCRNQDELIGRARISQGAGWSLRETACLGKSYAQMWSLMYFHRRDLRLAANAICSAVPSHWVPTSRTTWSIHARHEWMTTEDMLTVWNRVWIQENPWMEDKTPVESWEEIPYLGKREDQWCGSLIGLTSRATWARNIQTAINQVRSLIGNEEYTDYMPSMKRFRREEEEAGVLW

>DENV2_AAW51407_Indonesia

MNNQRKKARNTPFNMLKRERNRVSTVQQLTKRFSLGMLQGRGPLKLFMALVAFLRFLTIPPTAGILKRWGTIKKSKAINVLRGFRKEIGRMLNILNRRRRTAGIIIMMIPTVMAFHLTTRNGEPHMIVSRQEKGKSLLFKTENGVNMCTLMAMDLGELCEDTITYNCPLLRQNEPEDIDCWCNSTSTWVTYGTCTATGEHRREKRSVALVPHVGMGLETRTETWMSSEGAWKHAQRIETWVLRHPGFTIMAAILAYTIGTTYFQRVLIFILLTAVAPSMTMRCIGISNRDFVEGVSGGSWVDIVLEHGSCVTTMAKNKPTLDFELIKTEAKHPATLRKYCIEAKLTNTTTASRCPTQGEPSLNEEQDKRFVCKHSMVDRGWGNGCGLFGKGGIVTCAMFTCKKNMEGKIVQPENLEYTIVITPHSGEENAVGNDTGKHGKEIKVTPQSSITEAELTGYGTVTMECSPRTGLDFNEMVLLQMENKAWLVHRQWFLDLPLPWLPGADTQGSNWIQKETLVTFKNPHAKKQDVVVLGSQEGAMHTALTGATEIQMSSGNLLFTGHLKCRLRMDKLQLKGMSYSMCTGKFKVVKEIAETQHGTIVIRVQYEGDGSPCKIPFEIMDLEKRHVLGRLITVNPIVTEKDSPVNIEAEPPFGDSYIIIGVEPGQLKLSWFKKGSSIGQMFETTMRGAKRMAILGDTAWDFGSLGGVFTSIGKALHQVFGAIYGAAFSGVSWTMKILIGVVITWIGMNSRSTSLSVSLVLVGVVTLYLGVMVQADSGCVVSWKNKELKCGSGIFVTDNVHTWTEQYKFQPESPSKLASAIQKAHEEGICGIRSVTRLENLMWKQITPELNHILSENEVKLTIMTGDIKGIMQAGKRSLRPQPTELKYSWKTWGKAKMLSTELHNHTFLIDGPETAECPNTNRAWNSLEVEDYGFGVFTTNIWLKLKERQDVFCDSKLMSAAIKDNRAVHADMGYWIESALNDTWKIEKASFIEVKSCHWPKSHTLWSNGVLESEMIIPKNFAGPVSQHNYRPGYHTQTAGPWHLGRLEMDFDFCEGTTVVVTEDCGNRGPSLRTTTASGKLITEWCCRSCTLPPLRYRGEDGCWYGMEIRPLKEKEENLVNSLVTAGHGQIDNFSLGVLGMALFLEEMLRTRVGTKHAILLVAVSFVTLITGNMSFRDLGRVMVMVGATMTDDIGMGVTYLALLAAFKVRPTFAAGLLLRKLTSKELMMTTIGIVLLSQSTIPETILELTDALALGMMVLKIVRNMEKYQLAVTIMAILCVPNAVILQNAWKVSCTTLAVVSVSPLLLTSSQQKADWIPLALTIKGLNPTAIFLTTLARTSKKRSWPLNEAIMAVGMVSILASSLLKNDIPMTGPLVAGGLLTVCYVLTGRSADLELERAADVRWEEQAEISGSSPILSITISEDGSMSIKNEEEEQTLTILIRTGLLVISGLFPVSIPITAAAWYLWEVKKQRAGVLWDVPSPPPVGKAELEDGAYRIKQKGILGYSQIGAGVYKEGTFHTMWHVTRGAVLMHKGKRIEPSWADVKKDLISYGGGWKLEGEWKEGEEVQVLALEPGKNPRAVQTKPGLFKTNTGTIGAVSLDFSPGTSGSPIVDKKGKVVGLYGNGVVTRSGTYVSAIAQTEKSIEDNPEIEDDIFRKKRLTIMDLHPGAGKTKRYLPAIVREAIKRGLRTLILAPTRVVAAEMEEALRGLPIRYQTPAIRAEHTGREIVDLMCHATFTMRLLSPIRVPNYNLIIMDEAHFTDPASIAARGYISTRVEMGEAAGIFMTATPPGSRDPFPQSNAPIMDEEREIPERSWNSGHEWVTDFKGKTVWFVPSIKAGNDIAACLRKNGKKVIQLSRKTFDSEYIKTRTNDWDFVVTTDISEMGANFKAERVIDPRRCMKPVILTDGEERVILAGPMPVTHSSAAQRRGRVGRNPKNENDQYIYMGEPLENDEDCAHWKEAKMLLDNINTPEGIIPSMFEPEREKVDAIDGEYRLRGEARKTFVDLMRRGDLPVWLAYRVAAEGINYADRRWCFDGVKNNQILEENVEVEIWTKEGERKKLKPRWLDARIYSDPLALKEFKEFAAGRKSLTLNLITEMGRLPTFMTQKARNALDNLAVLHTAETGGRAYNHALSELPETLETLLLLTLLATVTGGIFLFLMSGKGIGKMTLGMCCIITASILLWYAQIQPHWIAASIILEFFLIVLLIPEPEKQRTPQDNQLTYVVIAILTVVAATMANEMGFLEKTKKDFGLGSITTQQPESNILDIDLRPASAWTLYAVATTFITPMLRHSIENSSVNVSLTAIANQATVLMGLGKGWPLSKMDIGVPLLAIGCYSQVNPITLTAALLLLVAHYAIIGPGLQAKATREAQKRAAAGIMKNPTVDGITVIDLDPIPYDPKFEKQLGQVMLLVLCVTQVLMMRTTWALCEALTLATGPISTLWEGNPGRFWNTTIAVSMANIFRGSYLAGAGLLFSIMKNTANTRRGTGNTGETLGEKWKNRLNALGKSEFQIYKKSGIQEVDRTLAKEGIKRGETDHHAVSRGSAKLRWFVERNLVTPEGKVVDLGCGRGGWSYYCGGLKNVKEVKGLTKGGPGHEEPIPMSTYGWNLVRLQSGVDVFFTPPEKCDTLLCDIGESSPNPTVEAGRTLRVLNLVENWLNNNTQFCIKVLNPYMPSVIEKMEALQRKYGGALVRNPLSRNSTHEMYWVSNASGNIVSSVNMISRMLINRFTMRHKKATYEPDVDLGSGTRNIGIESETPNLDIIGKRIEKIKQEHETSWHYDQDHPYKTWAYHGSYETKQTGSASSMVNGVVRLLTKPWDVIPMVTQMAMTDTTPFGQQRVFKEKVDTRTQEPKEGTKKLMKITAEWLWKELGKKKTPRMCTREEFTRKVRSNAALGAIFTDENKWKSAREAVEDSEFWELVDKERNLHLEGKCETCVYNMMGKREKKLGEFGKAKGSRAIWYMWLGARFLEFEALGFLNEDHWFSRENSLSGVEGEGLHKLGYILRDVSKKEGGAMYADDTAGWDTRITLEDLKNEEMVTNHMEGEHKKLAEAIFRLTYQNKVVRVQRPTPRGTVMDIISRRDQRGSGQVVTYGLNTFTNMEAQLIRQMEGEGVFKSIQHLTVTEEIAVKNWLVRVGRERLSRMAISGDDCVVKPLDDRFASALTALNDMGKVRKDIQQWEPSRGWNDWTQVPFCSHHFHELIMKDGRVLVVPCRNQDELIGRARISQGAGWSLRETACLGKSYAQMWSLMYFHRRDLRLAANAICSAVPSHWVPTSRTTWSIHATHEWMTTEDMLTVWNRVWIQENPWMEDKTPVESWEEIPYLGKREDQWCGSLIGLTSRATWAKNIQTAINQVRSLIGNEEYTDYMPSMKRFRREEEEAGVLW

>DENV2_BAU45374_East_Africa

MNNQRKKARNTPFNMLKRERNRVSTVQQLTKRFSLGMLQGRGPLKLFMALVAFLRFLTIPPTAGILKRWGTIKKSKAINVLRGFRKEIGRMLNILNRRRRTVGMIIMLIPTAMAFHLTTRNGEPHMIVSRQEKGKSLLFKTEDGVNMCTLMAMDLGELCEDTVTYNCPLLRQNEPEDIDCWCNSTSTWVTYGTCTATGEHRREKRSVALVPHVGMGLETRTETWMSSEGAWKHAQRIETWILRHPGFTIMAAILAHTIGTTYFQRVLIFILLTAVAPSMTMRCIGISNRDFVEGVSGGSWVDIVLEHGSCVTTMAKNKPTLDFELIKTEAKQPATLRKYCIEAKLTNTTTASRCPTQGEPSLNEEQDKRFVCKHSMVDRGWGNGCGLFGKGGIVTCAMFTCKKNMEGKIVQPENLEYTIVITPHSGEENAVGNDTGKHGKEVKVTPQSSITEAELTGYGTVTMECSPRTGLDFNEMVLLQMENKAWLVHRQWFLDLPLPWLPGADTQGSNWIQKETLVTFKNPHAKKQDVVVLGSQEGAMHTALTGATEIQMSSGNLLFTGHLKCRLRMDKLQLKGMSYSMCTGKFKVVKEIAETQHGTIVIRVQYEGDGSPCKIPFEIMDLEKRHVLGRLITVNPIVTERDSPVNIEAEPPFGDSYIIIGVEPGQLKLSWFKKGSSIGQMFETTMRGAKRMAILGDTAWDFGSLGGVFTSIGKALHQVFGAIYGAAFSGVSWTMKILIGVVITWIGMNSRSTSLSVSLVLVGVVTLYLGVMVQADSGCVVSWKNKELKCGSGIFITDNVHTWTEQYKFQPESPSKLASAIQKAYEEGICGIRSVTRLENLMWKQITPELNHILSENEVKLTIMTGDIKGIMQAGKRSLRPQPTELKYSWKTWGKAKMLSTEHHNQTLLIDGPETAECPNTNRAWNSLEVEDYGFGVFTTNIWLKLKERQDVFCDSKLMSAAIKDNRAVHADMGYWIESALNDTWKIEKASFIEVKSCHWPKSHTLWSNGVLESEMIIPKNFAGPVSQHNYRPGYHTQTAGPWHLGKLEMDFDFCEGTTVVVTEDCGNRGPSLRTTTASGKLITEWCCRSCTLPPLRYRGEDGCWYGMEIRPLKEKEENLVNSLVTAGHGQIDNFSLGVLGMALFLEEMLRTRVGTKHALLLVAVSFMTLITGNMSFRDLGRVMVMVGAAMTDDIGMGVTYLALLAAFKVRPTFAAGLLLRKLTSKELMMTTIGIVLLSQSTIPETILELTDALALGMMILKIVRNMEKYQLAVTIMAILCVPNAVILQNAWKVSCTILAVVSVTPLLLTSSRQKADWIPLALTIKGLNPTAIFLTTLSRTNKKRSWPLNEAIMAVGMVSILASSLLKNDIPMTGPLVAGGLLTVCYVLTGRSADLELERAADVRWEDQAEISGSSPILSITISEDGSMSIKNEEEEQTLTILIRTGLLVISGLFPVSIPITAAAWYLWEVKKQRAGVLWDVPSPPPVGKAELEDGAYRIKQKGILGYSQIGAGVYKEGTFHTMWHVTRGAVLMHKGKRIEPSWADVRKDLISYGGGWKLEGEWKEGEEVQVLALEPGKNPRAVQTKPGLFKTDTGTIGAVSLDFSPGTSGSPIVDKKGKVVGLYGNGVVTRSGTYVSAIAQTEKSIEDNPEIEDDIFRKRRLTIMDLHPGAGKTKRYLPAIVREAIKRGLRTLILAPTRVVAAEMEEALRGLPIRYQTPAIRAEHTGREIVDLMCHATFTMRLLSPVRVPNYNLIIMDEAHFTDPASIAARGYISTRVEMGEAAGIFMTATPPGSRDPFPQSNAPIMDEEREIPERSWNSGHEWVTDFKGKTVWFVPSIKAGNDIAACLRKNGKKVIQLSRKTFDSEYIKTRTNDWDFVVTTDISEMGANFKAERVIDPRRCMKPVILTDGEERVILAGPMPVTHSSAAQRRGRIGRNPKNENDQYIYMGEPLENDEDCAHWKEAKMLLDNINTPEGIIPSMFEPEREKVDAIDGEYRLRGEARKTFVDLMRRGDLPVWLAYRVAAEGINYADRRWCFDGVKNNQILEENVEVEIWTKEGERKKLKPRWLDARIYSDPLALKEFKEFAAGRKSLTLNLITEMGRLPTFMTQKARNALDNLAVLHTAEAGGRAYNHALSELPETLETLLLLTLLATVTGGIFLFLMSGKGIGKMTLGMCCIITASILLWYAQIQPHWIAASIILEFFLIVLLIPEPEKQRTPQDNQLTYVVIAILTVVAATMANEMGFLEKTKKDFGLGSIATQQPESNILDIDLRPASAWTLYAVATTFITPMLRHSIENSSVNVSLTAIANQATVLMGLGKGWPLSKMDIGVPLLAIGCYSQVNPITLTAALLLLVAHYAIIGPGLQAKATREAQKRAAAGIMKNPTVDGITVIDLDPIPYDPKFEKQLGQVMLLVLCVTQVLMMRTTWALCEALTLATGPISTLWEGNPGRFWNTTIAVSMANIFRGSYLAGAGLLFSIMKNTTNTRRGTGNTGETLGEKWKNRLNALGKSEFQTYKKSGIQEVDRTLAKEGIKRGETDHHAVSRGSAKLRWFVERNLVTPEGKVVDLGCGRGGWSYYCGGLKNVREVKGLTKGGPGHEEPIPMSTYGWNLVRLQSGVDVFFTPPEKCDTLLCDIGESSPNPTVEAGRTLRVLNLVENWLNNNTQFCIKVLNPYMPSVIEKMEALQRKYGGALVRNPLSRNSTHEMYWVSNASGNIVSSVNMISRMLINRFTMRHKKATYEPDVDLGSGTRNIGIESETPNLDIIGKRIEKIRQEHETSWHYDQDHPYKTWAYHGSYETKQTGSASSMVNGVVRLLTKPWDVIPMVTQMAMTDTTPFGQQRVFKEKVDTRTQEPKEGTKKLMKITAKWLWKELGKKKTPRMCTREEFTRKVRSNAALGAIFTDENKWKSAREAVEDSRFWELVDKERNLHLEGKCETCVYNMMGKREKKLGEFGKAKGSRAIWYMWLGARFLEFEALGFLNEDHWFSRENSLSGVEGEGLHKLGYILRDVSKKEGGAMYADDTAGWDTRITLEDLKNEEMVTNHMEGEHKKLAEAIFKLTYQNKVVRVQRPTPRGTVMDIISRRDQRGSGQVGTYGLNTFTNMGAQLIRQMEGEGVFKSIQHLTATEEIAVQDWLARVGRERLSRMAISGDDCVVKPLDDRFASALTALNDMGKVRKDIQQWEPSRGWNDWTQVPFCSHHFHELIMKDGRVLVVPCRNQDELIGRARISQGAGWSLRETACLGKSYAQMWSLMYFHRRDLRLAANAICSAVPSHWVPTSRTTWSIHAKHEWMTTEDMLTVWNRVWIQENPWMEDKTPVESWEEIPYLGKREDQWCGSLIGLTSRATWAKNIQTAINQVRSLIGNEEYTDYMPSMKRFRREEEEAGVLW

>DENV2_AIH13924_Saudi_Arabia

MNNQRKKARNTPFNMLKRERNRVSTVQQLTKRFSLGMLQGRGPLKLFMALVAFLRFLTIPPTAGILKRWGIIKKSKAINVLRGFRKEIGRMMNILNRRRRTAGVIIMLIPTAMAFHLTTRNGEPHMIVSKQEKGKSLLFKTEDGVNMCTLMAMDLGELCEDTITYNCPLLRQNEPEDIDCWCNSTSTWVTYGTCTATGEHRREKRSVALVPHVGMGLETRTETWMSSEGAWKHAQRIETWILRHPGFTIMAAILAYTIGTTYFQRVLIFILLTAVAPSMTMRCIGISNRDFVEGVSGGSWVDIVLEHGSCVTTMAKNKPTLDFELIKTEAKQPATLRKYCIEAKLTNTTTASRCPTQGEPSLNEEQDKRFVCKHSMVDRGWGNGCGLFGKGGIVTCAMFTCKKNMEGKIVQPENLEYTIVVTPHSGEENAVGNDTGKHGKEIKVTPQSSITEAELTGYGTVTMECSPRTGLDFNEMVLLQMENKAWLVHRQWFLDLPLPWLPGADIQGSNWIQKETLVTFKNPHAKKQDVVVLGSQEGAMHTALTGATEIQMSSGNLLFTGHLKCRLRMDKLQLKGMSYSMCTGKFKVVKEIAETQHGTIVVRVQYEGDGSPCKIPFEIMDLEKRHVLGRLITVNPIVTGKDSPVNIEAEPPFGDSYIIIGVEPGQLKLSWFKKGSSIGQMFETTMRGAKRMAILGDTAWDFGSLGGVFTSIGKALHQVFGAIYGAAFSGVSWTMKILIGVVITWIGMNSRSTSLSVSLVLVGVVTLYLGVMVQADSGCVVSWKNKELKCGSGIFITDNVHTWTEQYKFQPESPSKLASAIQKAHEEGICGIRSVTRLENLMWKQITPELNHILSENEVKLSIMTGDIKGIMQAGKRSLRPQPTELKYSWKTWGKAKMLSTEPHNQTFLIDGPETAECPNTNRAWNSLEVEDYGFGVFTTNIWLKLKERQDVSCDSKLMSAAIKDNRAVHADMGYWIESALNDTWKIEKASFIEVKSCHWPKSHTLWSNGVLESEMIIPKNFAGPVSQHNYRPGYHTQTAGPWHLGKLEMDFDFCEGTTVVVTEDCGNRGPSLRTTTASGKLITEWCCRSCTLPPLRYRGEDGCWYGMEIRPLKEKEENLVNSLVTAGHGQIDNFSLGVLGMALFLEEMLRTRVGTKHAVLLVAVSFMTLITGNMSFRDLGRVMVMVGAAMTDDIGMGVTYLALLAAFKVRPTFAAGLLLRKLTSKELMMTTIGIVLLSQSTTPESILELTDALALGMMILKIVRNMEKYQLAVTIMAILCVPNAVILQNAWKVSCTILAAVSVSPLLLTSSQQKADWIPLALTIKGLNPTAIFLTTLSRTNKKRSWPLNEAIMAVGMVSILASSLLKNDIPMTGPLVAGGLLTVCYVLTGRSADLELEKAADVRWEDQAEISGSSPILSITISEDGSMSIKNEEEEQTLTILIRTGLLVISGLFPVSIPITAAAWYLWEVKKQRAGVLWDVPSPPPVGKAELEDGAYRIKQKGILGYSQIGAGVYKEGTFHTMWHVTRGAVLMHKGKRIEPSWADVRKDLISYGGGWKLEGEWKEGEEVQVLALEPGKNPRAVQTKPGLFKTNTGTIGAVSLDFSPGTSGSPIVDKKGKVVGLYGNGVVTRSGTYVSAIAQTEKSIEDNPEIEDDIFRKKRLTIMDLHPGAGKTKRYLPAIVREAIKRGLRTLILAPTRVVAAEMEEALRGLPIRYQTPAIRAEHTGREIVDLMCHATFTMRLLSPVRVPNYNLIIMDEAHFTDPASIAARGYISTRVEMGEAAGIFMTATPPGSRDPFPQSNAPIMDEEREIPERSWNSGHEWVTDFKGKTVWFVPSIKAGNDIAACLRKNGKKVIQLSRKTFDSEYIKTRTNDWDFVVTTDISEMGANFKAERVIDPRRCMKPVILTDGEERVILAGPMPVTHSSAAQRRGRIGRNPKNENDQYIYMGEPLENDEDCAHWKEAKMLLDNINTPEGIIPSMFEPEREKVDAIDGEYRLRGEARKTFVDLMRRGDLPVWLAYRVAAEGINYADRRWCFDGIKNNQILEENVEVEIWTKEGERKKLKPRWLDARIYSDPLALKEFKEFAAGRKSLTLNLITEMGRLPTFMTQKARNALDNLAVLHTAEAGGRAYNHALSELPETLETLLLLTLLATVTGGIFLFLMSGKGIGKMTLGMCCIITASILLWYAQIQPHWIAASIILEFFLIVLLIPEPEKQRTPQDNQLTYVVIAILTVVAATMANEMGFLEKTKKDFGLGSIAIQQPESNILDIDLRPASAWTLYAVATTFITPMLRHSIENSSVNVSLTAIANQATVLMGLGKGWPLSKMDIGVPLLAIGCYSQVNPITLTAALLLLVAHYAIIGPGLQAKATREAQKRAAAGIMKNPTVDGVTVIDLDPIPYDPKFEKQLGQVMLLVLCVTQVLMMRTTWALCEALTLATGPISTLWEGNPGRFWNTTIAVSMANIFRGSYLAGAGLLFSIMKNTTNTRRGTGNTGETLGEKWKNRLNSLGKSEFQIYKRSGIQEVDRTLAKEGIKRGETDHHAVSRGSAKLRWFVERNLVTPEGKVVDLGCGRGGWSYYCGGLKNVREVKGLTKGGPGHEEPIPMSTYGWNLVRLQSGVDVFFTPPEKCDTLLCDIGESSPNPTVEAGRTLRVLNLVENWLNNNTQFCIKVLNPYMPSVIEKMETLQRKYGGALVRNPLSRNSTHEMYWVSNASGNIVSSVNMISRMLINRFTMRHKKATYEPDVDLGSGTRNIGIESETPNLDIIGKRIEKIKQEHETSWHYDQDHPYKTWAYHGSYETKQTGSASSMVNGVVRLLTKPWDVIPMVTQMAMTDTTPFGQQRVFKEKVDTRTQEPKEGTKKLMKITAEWLWKELGKKKTPRMCTREEFARKVRSNAALGAIFTDENKWKSAREAVEDSRFWELVDKERNLHLEGKCETCVYNMMGKREKKIGEFGKAKGSRAIWYMWLGARFLEFEALGFLNEDHWFSRENSLSGVEGEGLHKLGYILREVSKKEGGAMYADDTAGWDTRITLEDLKNEEMVTNHMEGEHKKLAEAIFKLTYQNKVVRVQRPTPRGTVMDIISRRDQRGSGQVGTYGLNTFTNMGAQLIRQMEGEGVFKGIQHLTATEEVAVQDWLARVGRERLSRMAISGDDCVVKPLDDRFASALTALNDMGKVRKDIQQWEPSRGWNDWTQVPFCSHHFHELIMKDGRVLVVPCRNQDELIGRARISQGAGWSLRETACLGKSYAQMWSLMYFHRRDLRLAANAICSAVPSHWVPTSRTTWSIHAGHEWMTTEDMLTVWNRVWIQENPWMEDKTPVESWEEIPYLGKREDQWCGSLIGLTSRATWARNIQTAINQVRSLIGSEEYTDYMPSMKRFRREEEEAGVLW

>DENV2_AGX15388_Peru

MNNQRKKARSTPFNMLKRERNRVSTVQQLTKRFSLGMLQGRGPLKLFMALVAFLRFLTIPPTAGILKRWGTIKKSKAINVLRGFRKEIGRMLNILNRRRRTAGVIVMLIPTAMAFHLTTRNGEPHMIVGRQEKGKSLLFKTEDGVNMCTLMAIDLGELCEDTITYKCPLLRQNEPEDIDCWCNSTSTWVTYGTCTTTGEHRREKRSVALVPHVGMGLETRTETWMSSEGAWKHVQRIETWILRHPGFTIMAAILAYTIGTTHFQRALIFILLTAVAPSMTMRCIGISNRDFVEGVSGGSWVDIVLEHGSCVTTMAKNKPTLDFELIKTEAKQPATLRKYCIEAKLTNTTTESRCPTQGEPSLNEEQDKRFICKHSMVDRGWGNGCGLFGKGGIVTCAMFTCKKNMEGKIVQPENLEYTIVITPHSGEEHAVGNDTGKHGKEIKITPQSSTTEAELTGYGTVTMECSPRTGLDFNEMVLLQMEDKAWLVHRQWFLDLPLPWLPGADTQGSNWIQKETLVTFKNPHAKKQDVVVLGSQEGAMHTALTGATEIQMSSGNLLFTGHLKCRLRMDKLQLKGMSYSMCTGKFKIVKEIAETQHGTIVIRVQYEGDGSPCKIPFEITDLEKRHVLGRLITVNPIVTEKDSPVNIEAEPPFGDSYIIVGVEPGQLKLNWFKKGSSIGQMFETTMRGAKRMAILGDTAWDFGSLGGVFTSIGKALHQVFGAIYGAAFSGVSWTMKILIGVIITWIGMNSRSTSLSVSLVLVGVVTLYLGAMVQADSGCIVSWKNKELKCGSGIFITDNVHTWTEQYKFQPESPSKLASAIQKAHEEGICGIRSVTRLENLMWKQITPELNHILSENEVKLTIMTGDIKGIMQAGKRSLRPQPTELKYSWKTWGKAKMLSTESHNQTFLIDGPETAECPNTNRAWNSLEVEDYGFGVFTTNIWLKLREKQDVFCDSKLMSAAIKDNRAVHADMGYWIESALNDTWKMEKASFIEVKSCHWPKSHTLWSNGVLESEMIIPKNFAGPVSQHNYRPGYHTQTAGPWHLGKLEMDFDFCEGTTVVVTEDCGNRGPSLRTTTASGKLITEWCCRSCTLPPLRYRGEDGCWYGMEIRPLKEKEENLVNSLVTAGHGQIDNFSLGVLGMALFLEEMLRTRVGTKHAILLVALSFVTLITGNMSFRDLGRVMVMVGATMTDDIGMGVTYLALLAAFKVRPTFAAGLLLRKLTSKELMMATIGIALLSQSTLPETILELTDALALGMMALKIVRNMEKYQLAVTIMAISCVPNAVILQNAWKVSCTILAAVSVSPLLLTSSQQKADWIPLALTIKGLNPTAIFLTTLSRTSKKRSWPLNEAIMAVGMVSILASSLLKNDIPMTGPLVAGGLLTVCYVLTGRSADLELERAADVKWEDQAEISGSSPILSITISEDGSMSIKNEEEEQTLTILIRTGLLVISGVFPVSIPITAAAWYLWEVKKQRAGVLWDVPSPPPVGKAELEDGAYRIKQRGILGYSQIGAGVYKEGTFHTMWHVTRGAVLMHRGKRIEPSWADVKKDLISYGGGWKLEGEWKEGEEVQVLALEPGKNPRAVQTKPGIFKTNTGTIGAVSLDFSPGTSGSPIVDRKGKVVGLYGNGVVTRSGAYVSAIAQTEKSIEDNPEIEDDIFRKKRLTIMDLHPGAGKTKRYLPAIVREAIKRGLRTLILAPTRVVAAEMEEALRGLPIRYQTPAIRAEHTGREIVDLMCHATFTMRLLSPVRVPNYNLIIMDEAHFTDPASIAARGYISTRVEMGEAAGIFMTATPPGSRDPFPQSNAPIMDEEREIPERSWNSGHEWVTDFKGKTVWFVPSIKAGNDIAACLRKNGKKVIQLSRKTFDSEYVKTRANDWDFVVTTDISEMGANFKAERVIDPRRCMKPVILTDGEERVILAGPMPVTHSSAAQRRGRIGRNPKNENDQYIYMGEPLENDEDCAHWKEAKMLLDNINTPEGIIPSMFEPEREKVDAIDGEYRLRGEARKTFVDLMRRGDLPVWLAYKVAAEGINYADRKWCFDGIKNNQILEENVEVEIWTKEGERKKLKPRWLDARIYSDPLALKEFKEFAAGRKSLTLNLITEMGRLPTFMTQKARNALDNLAVLHTAEVGGKAYTHALSELPETLETLLLLTLLAAVTGGIFLFLMSGKGIGKMTLGMCCIITASILLWYAQIQPHWIAASIILEFFLIVLLIPEPEKQRTPQDNQLTYVVIAILTVVAATMANEMGFLEKTKKDLGFGSITTQESESNILDIDLRPASAWTLYAVATTFVTPMLRHSIENSSVNVSLTAIANQATVLMGLGKGWPLSKMDIGVPLLAIGCYSQVNPITLTAALLLLVAHYAIIGPGLQAKATREAQKRAAAGIMKNPTVDGITVIDLEPIPYDPKFEKQLGQVMLLILCVTQVLMMRTTWALCEALTLATGPISTLWEGNPGRFWNTTIAVSMANIFRGSYLAGAGLLFSIMKNTTNTRRGTGNVGETLGEKWKSRLNALGKSEFQIYKKSGIQEVDRTLAKEGIKRGETDHHAVSRGSAKLRWFVERNMVTPEGKVVDLGCGRGGWSYYCGGLKNVREVKGLTKGGPGHEEPIPMSTYGWNLVRLQSGVDVFFTPPEKCDTLLCDIGESSPNPTIEAGRTLRVLNLVENWLNNNTQFCIKVLNPYMPSVIEKMETLQRKYGGALVRNPLSRNSTHEMYWVSNATGNIVSSVNMISRMLINRFTMKHKKATYEPDVDLGSGTRNIGIESEIPNLDIVGKRIEKIKQEHETSWHYDQDHPYKTWAYHGSYETKQTGSASSMVNGVVRLLTKPWDVVPMVTQMAMTDTTPFGQQRVFKEKVDTRTQEPKEGTKKLMKITAEWLWKELGKEKTPRMCTREEFTRKVRSNAALGAVFTDENKWKSAREAVEDGRFWELVDRERNLHLEGKCETCVYNMMGKREKKLGEFGKAKGSRAIWYMWLGARFLEFEALGFLNEDHWFSRGNSLSGVEGEGLHRLGYILREVGKKEGGAMYADDTAGWDTRITLEDLKNEEMVTNHMKGEHKKLAEAIFKLTYQNKVVRVQRPTPRGTVMDIISRKDQRGSGQVGTYGLNTFTNMEAQLIRQMEGEGIFKSIQHLTATEEIAVQNWLARVGRERLSRMAISGDDCVVKPIDDRFASALTALNDMGKVRKDIQQWEPSRGWNDWTQVPFCSHHFHELVMKDGRVLVVPCRNQDELIGRARISQGAGWSLKETACLGKSYAQMWTLMYFHRRDLRLAANAICSAVPSHWVPTSRTTWSIHAKHEWMTTEDMLVVWNRVWIQENPWMEDKTPVESWEEVPYLGKREDQWCGSLIGLTSRATWAKNIQTAINQVRSLIGNEGYTDYMPSMKRFRREEEEAGVLW

>DENV2_AIK23226._Cuba

MNNQRKKARNTPFNMLKRERNRVSTVQQLTKRFSLGMLQGRGPLKLFMALVAFLRFLTIPPTAGILKRWGTIKKSKAINVLRGFRKEIGRMLHILHRRRRTAGMIIMLIPTVMAFHLTTRNGEPHMIVSRQEKGKSLLFNTGDGVHMCTLMAMHLGELCEDTITYKCPLLRQNEPEDIDCWCNSTSTWVTYGTCTTTGEHRREKRSVALVPHVGMGLETRTETWMSSEGAWKHAQRIETWILRHPGFTIMAAILAYTIGTTHFQRALIFILLTAVAPSMTMRCIGISNRDFVEGVSGGSWVDIVLEHGSCVTTMAKNKPTLDLELIKTEAKQPATLRKYCIEAKLTNTTTESRCPTQGEPSLNEEQDKRFVCKHSMVDRGWGNGCGLFGKGSIVTCAMFTCKKIMKGKVVQPENLEYTIVITPHSGEEHAVGNDTGKHGKEIKITPQSSITEAELTGYGTVTMECSPRTGLDFNEMVLLQMEDKAWLVHRQWFLDLPLPWLPGADTQGSNWIQKETLVTFKNPHAKKQDVVVLGSQEGAMHTALTGATEIQMSSGNVLFTGHLKCRLRMDKLQLKGMSYSMCTGKFKVVKEIAETQHGTIVIRVQYEGDGSPCKIPFEIMDLEKRHVLGRLITVNPIVTGKDSPVNIEAEPPFGDSYIIIGVEPGQLKLNWFKKGSSIGQMFATTMRGAKRMAILGDTAWDFGSLGGVFTSIGKALHQVFGAIYGAAFSGVSWTMKILTGVIITWIGMNSRSTSLSVSLVLVGVVTLYLGVMVQADSGCVVSWKNKELKCGSGIFITDNVHTWTEQYKFQPESPSKLASAIQKAHEEGICGIRSVTRLENLMWKQITPELNHILSENEVKLTIMTGDIKGIMQAGKRSLRPQPTELKYSWKTWGKAKMLSTESHNQTFLIDGPETAECPNTNRAWNSLEVEDYGFGVFTTNIWLKLREKQDVFCDSKLMSAAIKDNRAVHADMGYWIESALNDTWKIEKASFIEVKSCHWPKSHTLWSNGVLESEMIIPKNFAGPVSQHNYRPGYHTQTAGPWHLGKLEMDFDFCEGTTVVVTEDCGNRGPSLRTTTASGKLITEWCCRSCTLPPLRYRGEDGCWYGMEIRPLKEKEENLVNSLVTAGHGQIDNFSLGVLGMALFLEEMLRTRVGTKHAILLVAVSFVTLITGNMSFRDLGRVMVMVGATMTDDIGMGVTYLALLAAFKVRPTFAAGLLLRKLTSKELMMTTIGIVLLSQSTIPETILELTDALALGMMVLKMVRKMEKYQLAVTIMAILCVPNAVILQNTWKVSCTILAVVSVSPLFLTSSQQKADWIPLALTIKGLNPTAIFLTTLSRTNKKRSWPLNEAIMAVGMVSILASSLLKNDIPMTGPLVAGGLLTVCYVLTGRSADLELERAADVKWEDQAEISGSSPILSITISEDGSMSIKNEEEEQTLTILIRTGLLVISGLFPVSIPITAAAWYLWEVKKQRAGVLWDVPSPPPVGKAELEDGAYRIKQKGILGYSQIGAGVYKEGTFHTMWHVTRGAVLMHKGKRIEPSWADVKKDLISYGGGWKLEGEWKEGEEVQVLALEPGKNPRAVQTKPGLFKTNAGTIGAVSLDFSPGTSGSPIIDKKGKVVGLYGNGVVTRSGAYVSAIAQTEKSIEDNPEIEDDIFRKRKLTIMDLHPGAGKTKRYLPAIVREAIKRGLRTLILAPTRVVAAEMEEALRGLPIRYQTPAIRAEHTGREIVDLMCHATFTMRLLSPVRVPNYNLIIMDEAHFTDPASIAARGYISTRVEMGEAAGIFMTATPPGSRDPFPQSNAPIMDEEREIPERSWSSGHEWVTDFKGKTVWFVPSIRAGNDIAACLRKNGKKVIQLSRKTFDSEYVKTRTNDWDFVVTTDISEMGANFKAERVIDPRRCMKPVILTDGEERVILAGPMPVTHSSAAQRRGRIGRNPKNENDQYIYMGEPLENDEDCAHWKEAKMLLDNINTPEGIIPSMFEPEREKVDAIDGEYRLRGEARKTFVDLMRRGDLPVWLAYRVAAEGINYADRRWCFDGIKNNQILEENVEVEIWTKEGERKKLKPRWLDARIYSDPLALKEFKEFAAGRKSLTLNLITEMGRLPTFMTQKARDALDNLAVLHTAEAGGRAYNHALSELPETLETLLLLTLLATVTGGIFLFLMSGRGIGKMTLGMCCIITASILLWYAQIQPHWIAASIILEFFLIVLLIPEPEKQRTPQDNQLTYVVIAILTVVAATMANEMGFLEKTKKDLGLGSITTQQPESNILDIDLRPASAWTLYAVATTFVTPMLRHSIENSSVNVSLTAIANQATVLMGLGKGWPLSKMDIGVPLLAIGCYSQVNPITLTAALFLLVAHYAIIGPGLQAKATREAQKRAAAGIMKNPTVDGITVIDLDPIPYDPKFEKQLGQVMLLVLCVTQVLMMRTTWALCEALTLATGPISTLWEGNPGRFWNTTIAVSMANIFRGSYLAGAGLLFSIMKNTTNTRRGTGNMGETLGEKWKSRLNALGKSEFQIYKKSGIQEVDRTLAKEGIKRGETDHHAVSRGSAKLRWFVERNMVTPEGKVVDLGCGRGGWSYYCGGLKNVREVKGLTKGGPGHEEPIPMSTYGWNLVRLQSGVDVFFTPPEKCDTLLCDIGESSPNPTVEAGRTLRVLNLVENWLNNNTQFCIKVLNPYMPSVIEKMEALQRKYGGALVRNPLSRNSTHEMYWVSNASGNIVSSVNMISRMLINRFTMRHKKATFEPDVDLGSGTRNIGIESEIPNLDIIGKRIEKIKQEYETSWHYDQDHPYKTWAYHGSYETKQTGSASSMVNGVVRLLTKPWDVVPMVTQMAMTDTTPFGQQRVFKEKVDTRTQEPKEGTKKLMKITAEWLWKELGKKKTPRMCTREEFTRKVRSNAALGAIFTDENKWKSAREAVEDSRFWELVDKERNLHLEGKCETCVYNMMGKREKKLGEFGKAKGSRAIWYMWLGARFLEFEALGFLNEDHWFSRENSLSGVEGEGLHKLGYILRDVSKKEGGAMYADDTAGWDTRITLEDLKNEEMVTNHMEGEHKKLAEAIFKLTYQNKVVRVQRPTPRGTVMDIISRRDQRGSGQVGTYGLNTFTNMEAQLIRQMEGEGVFKNIQHLTVTEEIAVQNWLARVGRERLSRMAISGDDCVVKPLDDRFASALTALNDMGKVRKDIQQWEPSRGWNDWTQVPFCSHHFHELIMKDGRVLVVPCRNQDELIGRARISQGAGWSLRETACLGKSYAQMWSLMYFHRRDLRLAANAICSAVPSHWVPTSRTTWSIHAKHEWMTTEDMLTVWNRVWIQENPWMEDKTPVESWEEIPYLGKREDQWCGSLIGLTSRATWAKNIQTAINQVRSLIGNEEYTDYMPSMKRFRREEEEAGVLW

>DENV2_AHZ61501_Taiwan

MNNQRKKARNTPFNMLKRERNRVSTVQQLTKRFSLGMLQGRGPLRLFMALVAFLRFLTIPPTAGILKRWGTIKKSKAINVLRGFRKEIGRMLNILNRRRRTAGMIIMLIPTVMAFHLTTRNGEPHMIVSRQEKGKSLLFKTEDGVNMCTLMAMDLGELCEDTITYKCPLLRQNEPEDIDCWCNSTSTWVTYGTCTTTGEHRREKRSVALVPHVGMGLETRTETWMSSEGAWKHAQRIETWILRHPGFTLMAAILAYTIGTTHFQRALIFILLTAVAPSMTMRCIGISNRDFVEGVSGGSWVDIVLEHGSCVTTMAKNKPTLDFELIKTEAKQLATLRKYCIEAKLTNTTTESRCPTQGEPSLNEEQDKRFVCKHSMVDRGWGNGCGLFGKGGIVTCAMFTCKKNMEGKIVQPENLEYTIVITPHSGEEHAVGNDTGKHGKEIKITPQSSITEAELTGYGTVTMECSPRTGLDFNEMVLLQMENKAWLVHRQWFLDLPLPWLPGADTQGSNWIQKETLVTFKNPHAKKQDVVVLGSQEGAMHTALTGATEIQMSSGNLLFTGHLKCRLRMDKLQLKGTSYSMCTGKFKVVKEIAETQHGTIVVRVQYEGDGSPCKIPFEIMDLEKRHVLGRLITVNPIVTEKDSPVNIEAEPPFGDSYVIIGVEPGQLKLNWFKKGSSIGQMFETTMRGAKRMAILGDTAWDFGSLGGVFTSIGKALHQVFGAIYGAAFSGVSWTMKILIGVIITWIGMNSRSTSLSVSLVLVGVVTLYLGVVVQADSGCVVSWKNKELKCGSGIFITDNVHTWTEQYKFQPESPSKLASAIQKAHQEGICGIRSVTRLENLMWKQITPELNHILSENEVKLTIMTGDIKGIMHAGKRSLRPQPTELRYSWKTWGKAKILSTESHNQTFLIDGPETAECPNTNRAWNSLEVEDYGFGVFTTNIWLKLREKQDVFCDSKLMSAAIKDNRAVHADMGYWIESALNDTWKIEKASFIEVKSCHWPKSHTLWSNGVLESEMIIPKNFAGPVSQHNYRPGYHTQTAGPWHLGKLEMDFDFCEGTTVVVTEDCGNRGPSLRTTTASGKLITEWCCRSCTLPPLRYRGEDGCWYGMEIRPLKEKEENLVNSLVTAGHGQIDNFSLGVLGMALFLEEMLRTRVGTKHATLLVAVSFVTLITGNMSFRDLGRVMVMVGATMTDDIGMGVTYLALLAAFKVRPTFAAGLLLRKLTSKELMMTTIGVVLLSQSTIPETILELTDALALGMMVLKIVRKMERYQLAVTITAILCVPNAVILQNAWKVSCTILAVVSVSPLLLTSSQQKADWIPLALTIKGLNPTAIFLTTLSRTSKKRSWPLNEAIMAVGMVSILASSLLKNDIPMTGPLVAGGLLTVCYVLTGRSADLELEKAADVRWEDQAEISGSSPILSITISEDGSMSIKNEEEEQTLTILIRTGLLVISGLFPVSIPITAAAWYLWEVKKQRAGVLWDVPSPPPVGKAELEDGAYRIKQKGILGYSQIGAGVYKEGTFHTMWHVTRGAVLMHKGKRIEPSWADVKKDLISYGGGWKLEGEWKEGEEVQVLALEPGKNPRAVQTKPGLFKTNTGTIGAVSLDFSPGTSGSPIIDKKGKVVGLYGNGVVTRNGAYVSAIAQTEKSIEDNPEIEDDIFRKKRLTIMDLHPGAGKTKRYLPAIVREAIKRGLRTLILAPTRVVAAEMEEALRGLPIRYQTPAIRAEHTGREIVDLMCHATFTMRLLSPVRVPNYNLIIMDEAHFTDPASIAARGYISTRVEMGEAAGIFMTATPPGSRDPFPQSNAPIMDEEREIPERSWNSGHEWVTDFKGKTVWFVPSIKAGNDIAACLRKNGKKVIQLSRKTFDSEYVKTRTNDWDFVVTTDISEMGANFKAERVIDPRRCMKPVILTDGEERVILAGPMPVTHSSAAQRRGRIGRNPKNENDQYIYMGEPLENDEDCAHWKEAKMLLDNINTPEGIIPSMFEPEREKVDAIDGEYRLRGEARKTFVDLMRRGDLPVWLAYKVAAEGINYADRRWCFDGIRNNQILEENVEVEIWTKEGERKKLKPRWLDARIYSDPLALKEFKEFAAGRKSLTLNLITEMGRLPTFMTQKARDALDNLAVLHTAEAGGRAYNHALSELPETLETLLLLTLLATVTGGIFLFLMSGRGIGRMPLGMCCIITASILLWYAQIQPHWIAASIILEFFLIVLLIPEPEKQRTPQDNQLTYVVIAILTVVAATMANEMGFLEKTKKDLGLGSITTQQPESNILDIDLRPASAWTLYAVATTFVTPMLRHSIENSSVNVSLTAIANQATVLMGLGKGWPLSKMDIGVPLLAIGCYSQVNPITLTAALLLLVAHYAIIGPGLQAKATREAQKRAAAGIMKNPTVDGITVIDLDPIPYDPKFEKQLGQVMLLVLCVTQVLMMRTTWALCEALTLATGPISTLWEGNPGRFWNTTIAVSMANIFRGSYLAGAGLLFSIMKNTTNTRRGTGNIGETLGEKWKNRLNALGKSEFQIYKKSGIQEVDRTLAKEGIKRGETDHHAVSRGSAKLRWFVERNMVTPEGKVVDLGCGRGGWSYYCGGLKNVREVKGLTKGGPGHEEPIPMSTYGWNLVRLQSGVDVFFTPPEKCDTLLCDIGESSPNPTVEAGRTLRVLNLVENWLNNNIQFCIKVLNPYMPSVIEKMEALQRKYGGALVRNPLSRNSTHEMYWVSNASGNIVSSVNMISRMLINRFTMKHKKATYEPDVDLGSGTRNIGIESEVPNLDIIGKRIEKIKQEHETSWHYDQDHPYKTWAYHGSYETKQTGSASSMVNGVVRLLTKPWDVVPMVTQMAMTDTTPFGQQRVFKEKVDTRTQEPKEGTKKLMKITAEWLWKELGKKKTPRMCTREEFTRKVRSNAALGAIFTDENKWKSAREAVEDSRFWGLVDKERNLHLEGKCETCVYNMMGKREKKLGEFGKAKGSRAIWYMWLGARFLEFEALGFLNEDHWFSRENSLSGVEGEGLHKLGYILRDVSKKEGGAMYADDTAGWDTRITLEDLKNEEMVTNHMEGEHKKLAEAIFKLTYQNKVVRVQRPTPRGTVMDIISRRDQRGSGQVGTYGLNTFTNMEAQLIRQMEGEGVFKSIQHLTVTEEIAVQNWLARVGRERLSRMAISGDDCVVKPLDDRFASALTALNDMGKVRKDIQQWEPSRGWSDWTQVPFCSHHFHELIMKDGRVLVVPCRNQDELIGRARISQGAGWSLRETACLGKSYAQMWSLMYFHRRDLRLAANAICSAVPSHWVPTSRTTWSIHAKHEWMTTEDMLTVWNRVWIQENPWMEDKTPVESWEEIPYLGKREDQWCGSLIGLTSRATWAKNIQTAINQVRSLIGNEEYTDYMPSMKRFRREEEEAGVLW

>DENV2_ACK57817_Guadeloupe

MNNQRKKARSTPFNMLKRERNRVSTVQQLTKRFSLGMLQGRGPLKLFMALVAFLRFLTIPPTAGILKRWGTIKKSKAINVLRGFRKEIGRMLNILNRRRRTAGVIVMLIPTAMAFHLTTRNGEPHMIVGRQEKGKSLLFKTEDGVNMCTLMAIDLGELCEDTITYKCPLLRQNEPEDIDCWCNSTSTWVTYGTCTTTGEHRREKRSVALVPHVGMGLETRTETWMSSEGAWKHVQRIETWILRHPGFTIMAAILAYTIGTTHFQRALIFILLTAVAPSMTMRCIGISNRDFVEGVSGGSWVDIVLEHGSCVTTMAKNKPTLDFELIKTEAKQPATLRKYCIEAKLTNTTTESRCPTQGEPSLNEEQDKRFICKHSMVDRGWGNGCGLFGKGGIVTCAMFTCKKNMEGKIVQPENLEYTIVITPHSGEEHAVGNDTGKHGKEIKITPQSSTTEAELTGYGTVTMECSPRTGLDFNEMVLLQMEDKAWLVHRQWFLDLPLPWLPGADTQGSNWIQKETLVTFKNPHAKKQDVVVLGSQEGAMHTALTGATEIQMSSGNLLFTGHLKCRLRMDKLQLKGMSYSMCTGKFKIMKEIAETQHGTIVIRVQYEGDGSPCKIPFEITDLEKRHVLGRLITVNPIVTEKDSPVNIEAEPPFGDSYIIVGVEPGQLKLNWFKKGSSIGQMFETTMRGAKRMAILGDTAWDFGSLGGVFTSIGKALHQVFGAIYGAAFSGVSWTMKILIGVIITWIGMNSRSTSLSVSLVLVGVVTLYLGAMVQADSGCIVSWKNKELKCGSGIFITDNVHTWTEQYKFQPESPSKLASAIQKAHEEGICGIRSVTRLENLMWKQITPELNHILSENEVKLTIMTGDIKGIMQAGKRSLRPQPTELKYSWKTWGKAKMLSTESHNQTFLIDGPETAECPNTNRAWNSLEVEDYGFGVFTTNIWLKLREKQDVFCDSKLMSAAIKDNRAVHADMGYWIESALNDTWKMEKASFIEVKSCHWPKSHTLWSNGVLESEMIIPKNFAGPVSQHNYRPGYHTQTAGPWHLGKLEMDFDFCEGTTVVVTEDCGNRGPSLRTTTASGKLITEWCCRSCTLPPLRYRGEDGCWYGMEIRPLKEKEENLVNSLVTAGHGQIDNFSLGVLGMALFLEEMLRTRVGTKHAILLVALSFVTLITGNMSFRDLGRVMVMVGATMTDDIGMGVTYLALLAAFKVRPTFAAGLLLRKLTSKELMMATIGIALLSQSTLPETILELTDALALGMMALKIVRNMEKYQLAVTIMAISCVPNAVILQNAWKVSCTILAAVSVSPLLLTSSQQKADWIPLALTIKGLNPTAIFLTTLSRTSKKRSWPLNEAIMAVGMVSILASSLLKNDIPMTGPLVAGGLLTVCYVLTGRSADLELERAADVKWEDQAEISGSSPILSITISEDGSMSIKNEEEEQTLTILIRTGLLVISGVFPVSIPITAAAWYLWEVKKQRAGVLWDVPSPPPVGKAELEDGAYRIKQRGILGYSQIGAGVYKEGTFHTMWHVTRGAVLMHRGKRIEPSWADVKKDLISYGGGWKLEGEWKEGEEVQVLALEPGKNPRAVQTKPGIFKTNTGTIGAVSLDFSPGTSGSPIVDRKGKVVGLYGNGVVTRSGAYVSAIAQTEKSIEDNPEIEDDIFRKKRLTIMDLHPGAGKTKRYLPAIVREAIKRGLRTLILAPTRVVAAEMEEALRGLPIRYQTPAIRAEHTGREIVDLMCHATFTMRLLSPVRVPNYNLIIMDEAHFTDPASIAARGYISTRVEMGEAAGIFMTATPPGSRDPFPQSNAPIMDEEREIPERSWNSGHEWVTDFKGKTVWFVPSIKAGNDIAACLRKNGKKVIQLSRKTFDSEYVKTRANDWDFVVTTDISEMGANFKAERVIDPRRCMKPVILTDGEERVILAGPMPVTHSSAAQRRGRIGRNPKNENDQYIYMGEPLENDEDCAHWKEAKMLLDNINTPEGIIPSMFEPEREKVDAIDGEYRLRGEARKTFVDLMRRGDLPVWLAYKVAAEGINYADRKWCFDGIKNNQILEENVEVEIWTKEGERKKLKPRWLDARIYSDPLALKEFKEFAAGRKSLTLNLITEMGRLPTFMTQKARNALDNLAVLHTAEVGGRAYTHALSELPETLETLLLLTLLATVTGGIFLFLMSGKGIGKMTLGMCCIITASILLWYAQIQPHWIAASIILEFFLIVLLIPEPEKQRTPQDNQLTYVVIAILTVVAATMANEMGFLEKTKKDLGFGSITTQESESNILDIDLRPASAWTLYAVATTFVTPMLRHSIENSSVNVSLTAIANQATVLMGLGKGWPLSKMDIGVPLLAIGCYSQVNPITLTAALLLLVAHYAIIGPGLQAKATREAQKRAAAGIMKNPTVDGITVIDLEPIPYDPKFEKQLGQVMLLILCVTQVLMMRTTWALCEALTLATGPISTLWEGNPGRFWNTTIAVSMANIFRGSYLAGAGLLFSIMKNTTNTRRGTGNVGETLGEKWKSRLNALGKSEFQIYKKSGIQEVDRTLAKEGIKRGETDHHAVSRGSAKLRWFVERNMVTPEGKVVDLGCGRGGWSYYCGGLKNVREVKGLTKGGPGHEEPIPMSTYGWNLVRLQSGVDVFFTPPEKCDTLLCDIGESSPNPTIEAGRTLRVLNLVENWLNNNTQFCIKVLNPYMPSVIEKMETLQRKYGGALVRNPLSRNSTHEMYWVSNATGNIVSSVNMISRMLINRFTMKHKKATYEPDVDLGSGTRNIGIESEIPNLDIIGKRIEKIKQEHETSWHYDQDHPYKTWAYHGSYETKQTGSASSMVNGVVRLLTKPWDVVPMVTQMAMTDTTPFGQQRVFKEKVDTRTQEPKEGTKKLMKITAEWLWKELGKEKTPRMCTREEFTRKVRSNAALGAIFTDENKWKSAREAVEDGRFWELVDRERNLHLEGKCETCVYNMMGKREKKLGEFGKAKGSRAIWYMWLGARFLEFEALGFLNEDHWFSRGNSLSGVEGEGLHRLGYILREVGKKEGGAMYADDTAGWDTRITLEDLKNEEMVTNHMKGEHKKLAEAIFKLTYQNKVVRVQRPTPRGTVMDIISRKDQRGSGQVGTYGLNTFTNMEAQLIRQMEGEGIFKSIQHLTATEEIAVQNWLARVGRERLSRMAISGDDCVVKPIDDRFASALTALNDMGKVRKDIQQWEPSRGWNDWTQVPFCSHHFHELVMKDGRVLVVPCRNQDELIGRARISQGAGWSLKETACLGKSYAQMWTLMYFHRRDLRLAANAICSAVPSHWVPTSRTTWSIHAKHEWMTTEDMLAVWNRVWIQENPWMEDKAPVESWEEVPYLGKREDQWCGSLIGLTSRATWAKNIQTAINQVRSLIGNEGYTDYMPSMKRFRREEEEAGVLW

>DENV2_AGO67249_Vietnam

MNNQRKKARSTPFNMLKRERNRVSTVQQLTKRFSLGMLQGRGPLKLFMALVAFLRFLTIPPTTGILKRWGTIKKSKAINVLRGFRKEIGRMLNILNRRRRTAGVIIMLIPTAMAFHLTTRNGEPHMIVGRQEKGKSLLFKTEDGVNMCTLMAIDLGELCEDTITYKCPLLRQSEPEDIDCWCNSTSTWVTYGTCTTTGEHRREKRSVALVPHVGMGLETRTETWMSSEGAWKHAQRIETWILRHPGFTIMAAILAYTIGTTHFQKALIFILLTAVAPSMTMRCIGISNRDFVEGVSGGSWVDIVLEHGSCVTTMAKNKPTLDFELIKTEAKEPATLRKYCIEAKLTNTTTESRCPTQGEPSLNEEQDKRFVCKHSMVDRGWGNGCGLFGKGGIVTCAMFTCKKNMEGKIVQPENLEYTIVITPHSGEEHAVGNDTGKHGKEIKITPQSSITEAELTGYGTVTMECSPRTGLDFNEMVLLQMEDKAWLVHRQWFLDLPLPWLPGADTQGSNWIQKETLVTFKNPHAKKQDVVVLGSQEGAMHTALTGATEIQMSSGNLLFTGHLKCRLRMDKLQLKGMSYSMCTGKFKIVKEIAETQHGTIVIRVQYEGDGSPCKIPFEIMDLEKRHVLGRLITVNPIVTEKDSPVNIEAEPPFGDSYIIIGVEPGQLKLNWFKKGSSIGQMFETTMRGAKRMAILGDTAWDFGSLGGVFTSIGKALHQVFGAIYGAAFSGVSWTMKILIGVIITWIGMNSRSTSLSVSLVLVGVITLYLGAMVQADSGCVVSWKNKELKCGSGIFITDNVHTWTEQYKFQPESPSKLASAIQKAHEEGICGIRSVTRLENLMWKQITPELNHILSENEVKLTIMTGDIKGIMQAGKRSLRPQPTELKYSWKTWGKAKMLSTESHNQTFLIDGPETAECPNKNRAWNSLEVEDYGFGVFTTNIWLKLREKQDVFCDSKLMSAAIKDNRAVHADMGYWIESALNDTWKMEKASFIEVKSCHWPKSHTLWSNGVLESEMIIPKSFAGPVSQHNYRPGYYTQTAGPWHLGKLEMDFDFCEGTTVVVTEDCGNRGPSLRTTTASGKLITEWCCRSCTLPPLRYRGEDGCWYGMEIRPLKEKEENLVNSLVTAGHGQIDNFSLGVLGMALFLEEMLRTRVGTKHAILLVAVSFVTLITGNMSFRDLGRVMVMVGATMTDDIGMGVTYLALLAAFKVRPTFAAGLLLKKLTSKELMMATIGIALLSQSTIPETILELTDALALGMMVLKIVRNMEKYQLAVTIMAISCVPNAVILQNAWKVSCTILAAVSVSPLLLTSSQQKADWIPLALTIKGLNPTAIFLTTLSRTSKKRSWPLNEAIMAVGMVSILASSLLKNDIPMTGPLVAGGLLTVCYVLTGRSADLELERATDVKWEDQAEISGSSPILSITISEDGSMSIKNEEEEQTLTILIRTGLLVISGLFPMSIPITAAAWYLWEVKKQRAGVLWDVPSPPPVGKAELEDGAYRIKQKGILGYSQIGAGVYKEGTFHTMWHVTRGAVLMHRGKRIEPSWADVKKDLISYGGGWKLEGEWKEGEEVQVLALEPGKNPRAVQTKPGLFKTNTGTIGAVSLDFSPGTSGSPIVDRKGKVVGLYGNGVVTRSGAYVSAIAQTEKSIEDNPEIEDDIFRKKRLTIMDLHPGAGKTKRYLPAIVREAIKRGLRTLILAPTRVVAAEMEEALRGLPIRYQTPAIRAEHTGREIVDLMCHATFTMRLLSPVRVPNYNLIIMDEAHFTDPASIAARGYISTRVEMGEAAGIFMTATPPGSRDPFPQSNAPIMDEEREIPERSWNSGHEWVTDFKGKTVWFVPSIKAGNDIAACLRKNGKKVIQLSRKTFDSEYVKTRTNDWDFVVTTDISEMGANFKAERVIDPRRCMKPVILTDGEERVILAGPMPVTHSSAAQRRGRIGRNPKNENDQYIYMGEPLENDEDCAHWKEAKMLLDNINTPEGIIPSMFEPEREKVDAIDGEYRLRGEARKTFVDLMRRGDLPVWLAYRVAAEGINYADRRWCFDGVKNNQILEENVEVEIWTKEGERKKLKPRWLDARIYSDPLALKEFKEFAAGRKSLTLNLITEMGRLPTFMTQKARDALDNLAVLHTAEAGGRAYNHALSELPETLETLLLLTLLATVTGGIFLFLMSGKGVGKMTLGMCCIITASILLWYAQIQPHWIAASIILEFFLIVLLIPEPEKQRTPQDNQLTYVVIAILTVVAATMANEMGFLEKTKKDLGLGSIATQEPESNILDIDLRPASAWTLYAVATTFVTPMLRHSIENSSVNVSLTAIANQATVLMGLGKGWPLSKMDIGVPLLAIGCYSQVNPITLTAALLLLIAHYAIIGPGLQAKATREAQKRAAAGIMKNPTVDGITVIDLDPIPYDPKFEKQLGQVMLLILCVTQVLMMRTTWALCEALTLATGPISTLWEGNPGRFWNTTIAVSMANIFRGSYLAGAGLLFSIMKNTTNTRRGTGNIGETLGEKWKSRLNALGKSEFQIYKKSGIQEVDRTLAKEGIKRGETDHHAVSRGSAKLRWFVERNMVTPEGKVVDLGCGRGGWSYYCGGLKNVREVKGLTKGGPGHEEPIPMSTYGWNLVRLQSGVDVFFTPPEKCDTLLCDIGESSPNPTIEAGRTLRVLSLVENWLNNDTQFCIKVLNPYMPSVIEKMETLQRKYGGALVRNPLSRNSTHEMYWVSNATGNIVSSVNMISRMLINRFTMKHKKATYEPDVDLGSGTRNIGIESEIPNLDIIGKRIEKIKQEHETSWHYDQDHPYKTWAYHGSYETKQTGSASSMVNGVVRLLTKPWDVVPMVTQMAMTDTTPFGQQRVFKEKVDTRTQEPKEGTKKLMKITAEWLWKELGKKKTPRMCTREEFTRKVRSNAALGAIFTDENKWKSAREAVEDSRFWELVDKERNLHLEGKCETCVYNMMGKREKKLGEFGKAKGSRAIWYMWLGARFLEFEALGFLNEDHWFSRENSLSGVEGEGLHKLGYILRDVSKKEGGAMYADDTAGWDTRITLEDLKNEEMVTNHMEGEHKKLAEAIFKLTYQNKVVRVQRPTPRGTVMDIISRRDQRGSGQVGTYGLNTFTNMEAQLIRQMEGEGIFKSIQHLTVTEEIAVQDWLARVGRERLSRMAISGDDCVVKPLDDRFASALTALNDMGKVRKDIQQWEPSRGWNDWTQVPFCSHHFHELIMKDGRVLVVPCRNQDELIGRARISQGAGWSLKETACLGKSYAQMWSLMYFHRRDLRLAANAICSAVPSHWVPTSRTTWSIHAKHEWMTTEDMLTVWNRVWIQENPWMEDKTPVESWEEVPYLGKREDQWCGSLIGLTSRATWAKNIQTAINQVRSLIGNEEYTDYMPSMKRFRREEEEAGVLW

>DENV2_ADK26435_Guam

MNNQRKKARNTPFNMLKRERNRVSTVQQLTKRFSLGMLQGRGPLKLFMALVAFLRFLTIPPTAGILKRWGTIKKSKAINVLRGFRKEIGRMLNILNRRRRTAGIIIMMIPTVMAFHLTTRNGEPHMIVSRQEKGKSLLFKTENGVNMCTLMAMDLGELCEDTITYNCPLLRQNEPEDIDCWCNSTSTWVTYGTCTVTGEHRREKRSVALVPHVGMGLETRTETWMSSEGAWKHAQRIETWVLRHPGFTIMAAILAYTIGTTYFQRVLIFILLTAVAPSMTMRCIGISNRDFVEGVSGGSWVDIVLEHGSCVTTMAKNKPTLDFELIKTEAKHPATLRKYCIEAKLTNTTTASRCPTQGEPSLNEEQDKRFVCKHSMVDRGWGNGCGLFGKGGIVTCAMFTCKKNMEGKIVQPENLEYTIVITPHSGEENAVGNDTGKHGKEIKVTPQSSITEAELTGYGTVTMECSPRTGLDFNEMVLLQMENKAWLVHRQWFLDLPLPWLPGADTQGSNWIQKETLVTFKNPHAKKQDVVVLGSQEGAMHTALTGATEIQMSSGNLLFTGHLKCRLRMDKLQLKGMSYSMCTGKFKVVKEIAETQHGTIVIRVQYEGDGSPCKIPFEIMDLEKRHVLGRLITVNPIVTEKDSPVNIEAEPPFGDSYIIVGVEPGQLKLSWFKKGSSIGQMFETTMRGAKRMAILGDTAWDFGSLGGVFTSIGKALHQVFGAIYGAAFSGVSWTMKILIGVVITWIGMNSRSTSLSVSLVLVGVVTLYLGVMVQADSGCVVSWKNKELKCGSGIFITDNVHTWTEQYKFQPESPSKLASAIQKAHEEGICGIRSVTRLENLMWKQITPELNHILSENEVKLTIMTGDIKGIMQAGKRSLRPQPTELKYSWKAWGKAKMLSTELHNHTFLIDGPETAECPNTNRAWNSLEVEDYGFGVFTTNIWLKLKERQDVFCDSKLMSAAIKDNRAVHADMGYWIESALNDTWKIEKASFIEVKSCHWPKSHTLWSNGVLESEMIIPKNFAGPVSQHNYRPGYHTQTAGPWHLGRLEMDFDFCEGTTVVVTEDCGNRGPSLRTTTASGKLITEWCCRSCTLPPLRYRGEDGCWYGMEIRPLKEKEENLVNSLVTAGHGQIDNFSLGVLGMALFLEEMLRTRVGTKHAILLVAVSFVTLITGNMSFRDLGRVMVMVGATMTDDIGMGVTYLALLAAFKVRPTYAAGLLLRKLTSKELMMTTIGIVLLSQSTIPETILELTDALALGMMVLKIVRNMEKYQLAVTIMAILCVPNAVILQNAWKVSCTTLAMVSVSPLLLTSSQQKADWIPLALTIKGLNPTAIFLTTLSRTSKKRSWPLNEAIMAVGMVSILASSLLKNDIPMTGPLVAGGLLTVCYVLTGRSADLELERAADVRWEEQAEISGSSPILSITISEDGSMSIKNEEEEQTLTILIRTGLLVISGLFPVSIPITAAAWYLWEVKKQRAGVLWDVPSPPPVGKAELEDGAYRIKQKGILGYSQIGAGVYKEGTFHTMWHVTRGAVLMHKGKRIEPSWADVKKDLISYGGGWKLEGEWKEGEEVQVLALEPGKNPRAVQTKPGLFKTNTGTIGAVSLDFSPGTSGSPIVDKKGKVVGLYGNGVVTRSGTYVSAIAQTEKSIEDNPEIEDDIFRKKRLTIMDLHPGAGKTKRYLPAIVREAIKRGLRTLILAPTRVVAAEMEEALRGLPIRYQTPAIRAEHTGREIVDLMCHATFTMRLLSPIRVPNYNLIIMDEAHFTDPASIAARGYISTRVEMGEAAGIFMTATPPGSRDPFPQSNAPIMDEEREIPERSWNSGHEWVTDFKGKTVWFVPSIKAGNDIAACLRKNGKKVIQLSRKTFDSEYIKTRTNDWDFVVTTDISEMGANFKAERVIDPRRCMKPVILTDGEERVILAGPMPVTHSSAAQRRGRVGRNPKNENDQYIYMGEPLENDEDCAHWKEAKMLLDNINTPEGIIPSMFEPEREKVDAIDGEYRLRGEARKTFVDLMRRGDLPVWLAYRVAAEGINYADRRWCFDGVKNNQILEENVEVEIWTKEGERKKLKPRWLDARIYSDPLALKEFKEFAAGRKSLTLNLITEMGRLPTFMTQKARNALDNLAVLHTAEAGGRAYNHALSELPETLETLLLLTLLATVTGGIFLFLMSGKGIGKMTLGMCCIITASILLWYAQIQPHWIAASIILEFFLIVLLIPEPEKQRTPQDNQLTYVVIAILTVVAATMANEMGFLEKTKKDFGLGSITTQQPESNILDIDLRPASAWTLYAVATTFITPMLRHSIENSSVNVSLTAIANQATVLMGLGKGWPLSKMDIGVPLLAIGCYSQVNPITLTAALLLLVAHYAIIGPGLQAKATREAQKRAAAGIMKNPTVDGITVIDLDPIPYDPKFEKQLGQVMLLVLCVTQVLMMRTTWALCEALTLATGPISTLWEGNPGRFWNTTIAVSMANIFRGSYLAGAGLLFSIMKNTANTRRGTGNTGETLGEKWKHRLNALGKSEFQIYKKSGIQEVDRTLAKEGIKRGETDHHAVSRGSAKLRWFVERNLVTPEGKVVDLGCGRGGWSYYCGGLKNVKEVKGLTKGGPGHEEPIPMSTYGWNLVRLQSGVDVFFTPPEKCDTLLCDIGESSPNPTVEAGRTLRVLNLVENWLNNNTQFCIKVLNPYMPSVIEKMEALQRKYGGALVRNPLSRNSTHEMYWVSNASGNIVSSVNMISRMLINRFTMRHKKATYEPDVDLGSGTRNIGIESETPNLDIIGKRIEKIKQEHETSWHYDQDHPYKTWAYHGSYETKQTGSASSMVNGVVRLLTKPWDVIPMVTQMAMTDTTPFGQQRVFKEKVDTRTQEPKEGTKKLMKITAEWLWKELGKKKTPRMCTREEFTRKVRSNAALGAIFTDENKWKSAREAVEDSGFWELVDKERNLHLEGKCETCVYNMMGKREKKLGEFGKAKGSRAIWYMWLGARFLEFEALGFLNEDHWFSRKNSLSGVEGEGLHKLGYILRDVSKKEGGAMYADDTAGWDTRITLEDLKNEEMVTNHMEGEHKKLAEAIFRLTYQNKVVRVQRPTPRGTVMDIISRRDQRGSGQVVTYGLNTFTNMEAQLIRQMEGEGVFKSIQHLTATEEIAVKTWLVRVGRERLSRMAISGDDCVVKPLDDRFASALTALNDMGKVRKDIQQWEPSRGWNDWTQVPFCSHHFHELIMKDGRVLVVPCRNQDELIGRARISQGAGWSLRETACLGKSYAQMWSLMYFHRRDLRLAANAICSAVPSHWVPTSRTTWSIHATHEWMTTEDMLTVWNRVWIQENPWMEDKTPVESWEEIPYLGKREDQWCGSLIGLTSRATWAKNIQTAINQVRSLIGNEEYTDYMPSMKRFRREEEEAGVLW

>DENV2_ABR13877_Senegal
[truncated: 718,939 more chars]
